# Staking out the frontiers: The limits of the metazoan shallow marine fossil record

**DOI:** 10.1126/sciadv.aeh3920

**Published:** 2026-09-23

**Authors:** Thomas A. Neubauer, Mathias Harzhauser

**Affiliations:** ^1^ SNSB–Bavarian State Collection for Palaeontology and Geology, Munich 80333, Germany.; ^2^ Department of Earth and Environmental Sciences, Palaeontology and Geobiology, Ludwig-Maximilians-Universität München, Munich 80333, Germany.; ^3^ Geological-Paleontological Department, Natural History Museum Vienna, Vienna 1010, Austria.; ^4^ Institut für Erdwissenschaften, NAWI Graz Geocenter, Universität Graz, Graz 8010, Austria.

## Abstract

The fossil record is inherently incomplete, yet its physical limits remain largely uncharted. Here, we quantify the spatiotemporal coverage of the global shallow-marine metazoan fossil record across the Phanerozoic, using a large dataset of georeferenced collections intersected with global geological data. Modeling historical sampling trajectories, we show that for most time intervals, future data accumulation is expected to be negligible, and geographic coverage is approaching saturation. For intervals with remaining potential, we reconstruct the present-day extent of unexplored but geologically accessible strata, providing spatially explicit guidance on where undetected fossil information is most likely to await discovery. Our results constitute a quantitative roadmap for prioritizing fieldwork, completing occurrence databases, and mobilizing unpublished museum collections.

## INTRODUCTION

The fossil record offers a unique window into the evolution of life on our planet. Over the centuries, fossil discoveries have produced a wealth of data, allowing us to reconstruct the rise and demise of diversity on various taxonomic and ecologic scales, understand the roles of evolutionary innovations and mass extinctions, or unravel biogeographic relationships, to name a few (*1*, *2*). A prime focus over the past decades has been on the development of marine metazoan diversity through the Phanerozoic. Starting off with the milestone works of J.J. Sepkoski in the 1980s (*3*, *4*), numerous studies have analyzed diversity dynamics over long timescales and the factors driving its temporal and spatial variability (*5*–*15*).

However, the fossil record is inherently incomplete (*16*, *17*). Any reconstruction of past life and biotic dynamics is inevitably skewed by rock record availability and accessibility, sampling intensity, preservation variability across time and with respect to intrinsic factors, such as body size, skeletal mineralogy, and mode of life, as well as varying research focus (*18*–*20*). Many works have pinpointed the effects of or accounting for such variation in reconstructions of past diversity dynamics (*5*, *21*–*28*). Others have specifically quantified the (in)completeness of the fossil record itself (*18*, *29*). A milestone work in this regard was published recently by Ye and Peters (*30*) who predicted expected fossil occurrences worldwide based on global bedrock data.

Our knowledge of geological history is intrinsically positivist and focuses on published data from defined regions. Despite all analytical advances, our understanding of the physical limits of the fossil record, and especially the uncharted territories that remain yet to be explored, is still inaccurate on a global scale. To date, there is no geographical overview of the terrae incognitae of the various time intervals of the geological past, nor have such “black maps” been merged with paleogeography. However, such information is crucial to allow for more robust reconstructions of past biotic patterns and processes and, in the long run, also facilitate predictions of future biodiversity change by providing a more profound data basis.

Here, we establish a pipeline to quantify spatiotemporal coverage and its limits of the global marine shallow-water metazoan fossil record of the entire Phanerozoic. Based on a large dataset retrieved from the Paleobiology Database (https://paleobiodb.org/), the most comprehensive database for macrofossil occurrences, we quantify the geographic coverage of the marine fossil record (or the lack thereof) across 98 stratigraphic stages. For each interval, we evaluate whether current research trends indicate future geographic expansion or rather point to saturation, i.e., that known regions are repeatedly sampled. By reconstructing the present-day position of the shallow-water regions for which the Paleobiology Database hosts no data and intersecting these areas with global spatial geological data available through the Macrostrat Database (*31*), we pinpoint the regions and sedimentary strata for a given time period that may hold undetected fossil information. This paper focuses on shallow-water regions as these ecosystems host a majority of metazoan diversity throughout the fossil record, but the approach is readily extendable to any type of environment.

## RESULTS

### The paucity of the marine fossil record

**Fig. 1.**
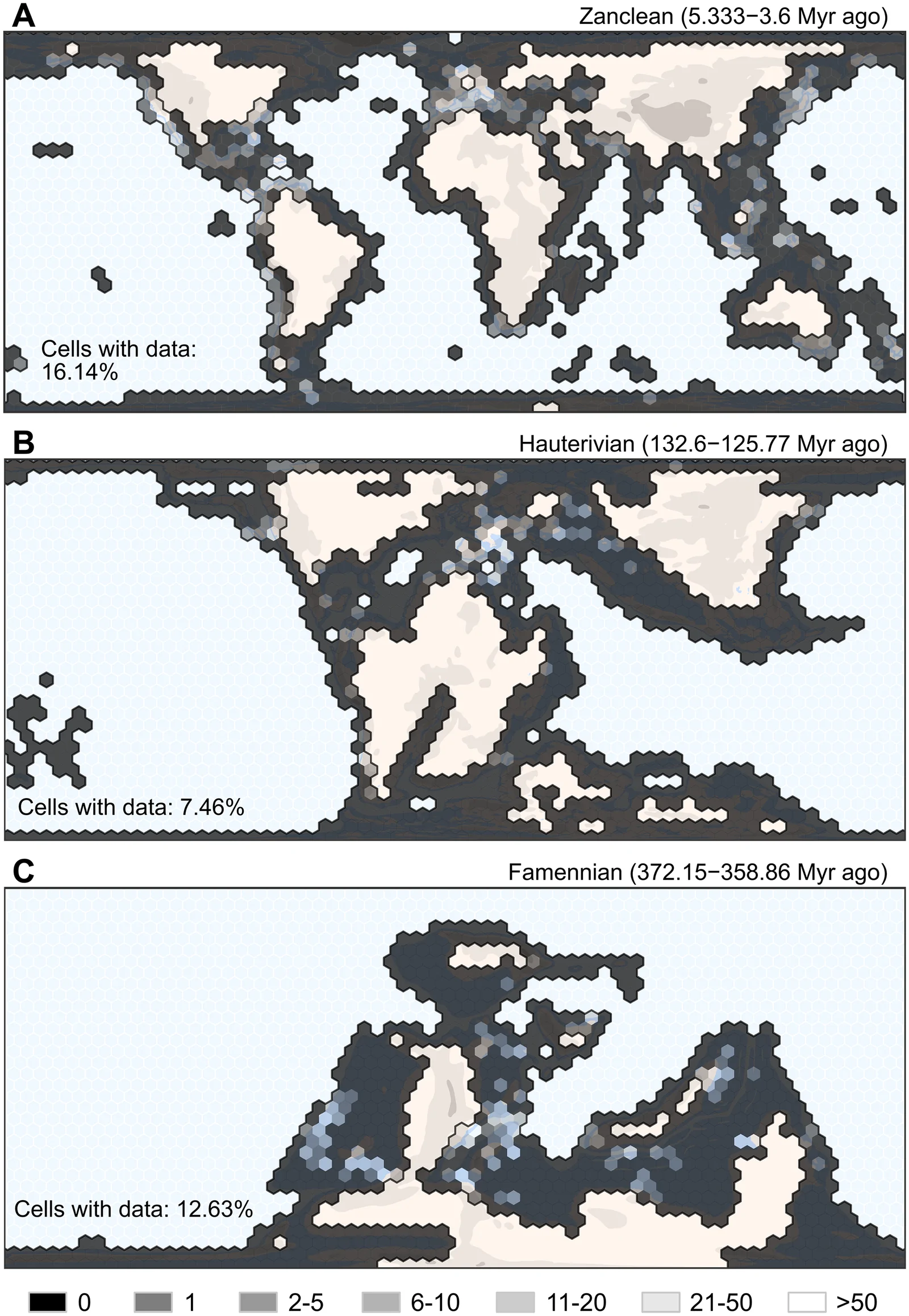
Example paleogeographic maps showing information density on shallow-marine metazoans as light hexagons. The number of fossil collections per grid cell is displayed as relative transparency. Note the high number of dark hexagons lacking any data. (**A**) Zanclean (Early Pliocene, 5.333 to 3.6 Myr ago), as an example for a time interval with a relatively well sampled metazoan fossil record in shallow waters (16.14%) and a high potential for future expansion (compare Fig. 2). (**B**) Hauterivian (Early Cretaceous, 132.6 to 125.77 Myr ago), as a representative of a comparatively poorly sampled shallow-water record (7.46%) and hardly any tendency for expansion. (**C**) Famennian (Late Devonian, 372.15 to 358.86 Myr ago), with a moderate coverage of shallow-marine regions (12.63%) and predicted slight expansion. All figures are based on the 500-km, pointy-topped grid; see figs. S1 to S6 for the full set of maps across all grid types and figs. S24 to S29 for the results of the 49-bin approach.

Our maps binning fossil collections into equal-area hexagonal grids reveal strong variation of the geographic coverage of shallow marine regions through time (Fig. 1, figs. S1 to S6, and table S3). Despite the vast number of fossil collections in our dataset (148,960), coverage is extraordinarily thin for most time bins throughout the Phanerozoic. Across all stratigraphic stages and types of grids chosen (see the Supplementary Materials), only 10.7% of the cells overlapping with shallow marine waters contain fossil information on average. Stages with the highest coverages include the Norian (Late Triassic, 227.3 to 205.7 Myr ago; mean of 23.9% across all grid types), Katian (Late Ordovician, 452.8 to 445.2 Myr ago; 21.5%), Cambrian Stage 4 (514.5 to 506.5 Myr ago; 18.6%), and Carnian (Late Triassic, 237 to 227.3 Myr ago; 18.6%). Lowest coverages were retrieved for the Fortunian (early Cambrian, 538.8 to 529.0 Myr ago; 0.6%), Wuliuan (Miaolingian, 506.5 to 504.5 Myr ago; 2.1%), Selandian (Middle Paleocene, 61.66 to 59.24 Myr ago; 2.9%), and Gelasian (Early Pleistocene, 2.58 to 1.8 Myr ago; 2.9%).

When binned into longer stratigraphic units, average data coverage rises to 16.1%. Moreover, the combination of data across stages results in different bins having highest and lowest coverage. The youngest time bin (11.63 to 0 Myr ago, Tortonian–Pleistocene) has the highest coverage (mean of 32.5% across all grid types), followed by the Late Triassic (227.3 to 201.4 Myr ago, Norian–Rhaetian; 31.1%) and mid-Permian (274.4 to 259.51 Myr ago, Guadalupian; 27.0%) datasets. Lowest coverages were retrieved for the early Cambrian (538.8 to 521.0 Myr ago, Terreneuvian; 5.9%), the earliest Jurassic (201.4 to 199.5 Myr ago, Hettangian; 7.0%), and late Mississippian (330.3 to 323.4 Myr ago, Serpukhovian; 7.7%). For both stages and bins, no trend through time is detectable. To some extent, the variable geographic coverage is likely linked to the different durations of the time bins, ranging from 0.774 Myr (Chibanian–Late Pleistocene) to 25.9 Myr (Norian–Rhaetian). A Pearson’s correlation test revealed only a weak positive correlation though (*r* = 0.555, *P* < 0.001; *r* = 0.412, *P* = 0.0033 for the 49-bin approach).

**Fig. 2.**
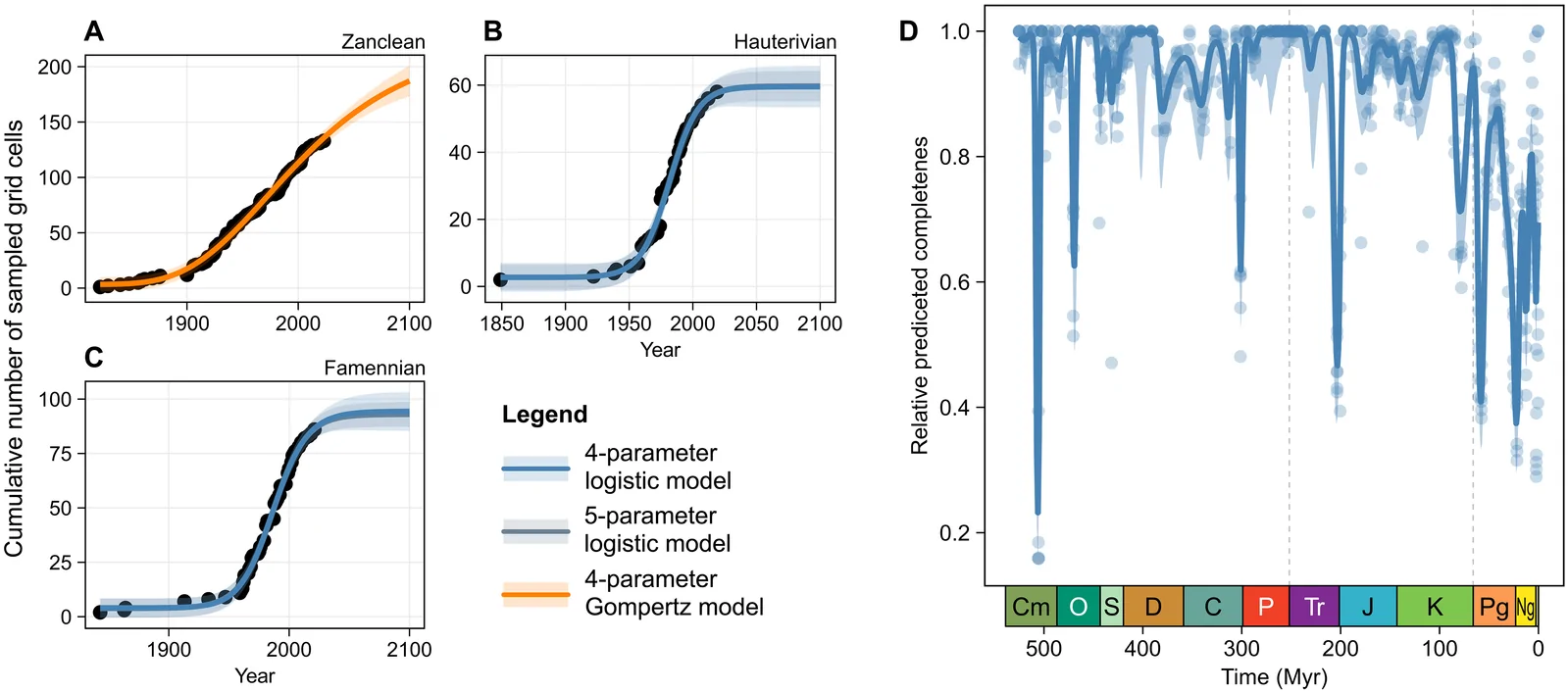
Historical sampling trends and (in)completeness of shallow-water areas. (**A** to **C**) Sampling trends and future prediction for 2100 for the time bins in Fig. 1, indicating various degrees of completeness in shallow-water grid cells, estimated based on historical sampling effort. For some periods, several models rank among the best. (**D**) Summary graph based on predictions across the 98 stages and six grid types analyzed. Completeness is quantified as the difference between the observed maximum number of grid cells and the predicted number based on logistic or logistic-like models. The temporal trend was visualized using a quasi-binomial generalized additive model. All figures are based on the 500-km, pointy-topped grid; see figs. S7 to S12 for all models across grid types and figs. S30 to S36 for the results of the 49-bin approach.

A clearer picture is drawn by the analyses of future projections on geographic coverage (i.e., sampled grid cells). Based on historical sampling effort, we modeled the most probable sampling trend until the year 2100 per time bin. Sampling effort trends suggest that the geographic coverage of the known fossil record is saturated for over a fourth of all time intervals (27.9%; 36.4% for the 49-bin approach; Fig. 2, figs. S7 to S12 and S30 to S35, and tables S4 and S10). Another 40.6% (36.7%) are more than 90% complete, and only 22.2% (15.6%) have less than 80% of the data that can be expected if sampling tendencies persist. Across all grid sizes and types, we see an increase in average estimated coverage from the latest Cretaceous to the Pleistocene (Fig. 2D and figs. S7 to S12 and S30 to S35). In turn, our models indicate that sampling for most of the time bins from the Cambrian to the Jurassic approach an asymptote, or nearly so, and are unlikely to deliver a substantial amount of new data in the future. Most of the Cenozoic and latest Cretaceous time bins (up to the Coniacian, 89.8 to 85.7 Myr ago), as well as notable outliers in the Wuliuan (Miaolingian), Dapingian (Middle Ordovician, 471.3 to 469.4 Myr ago), Gzhelian (Late Pennsylvanian, 303.7 to 298.9 Myr ago), and Rhaetian (Late Triassic, 205.7 to 201.4 Myr ago) are more promising for future expansions of the geographic scope (Fig. 2D, figs. S7 to S12, and table S5; see figs. S30 to S36 and table S11 for the 49-bin approach). We noted variation of the estimated grid coverage across grid types, but differences were statistically not significant [analysis of variance (ANOVA): *P* = 0.945 for stages, *P* = 0.894 for bins; Tukey post hoc test for pairwise comparison: smallest *P* = 0.941 for stages, *P* = 0.864 for bins].

To evaluate whether our results are a particular feature of shallow-water regions or otherwise biased from the data filters applied, we used the same models also for the full marine dataset (including collections mapped as deep-marine or terrestrial). Results varied slightly but drew a similar overall picture; 19.8% of all stages (29.1% for bins) indicated saturation of collection effort on average, and 30.0% (20.3%) would have less than 80% coverage if trends persisted (figs. S13 to S18 and S37 to S42 and tables S6 and S12). On a larger scale, a model of the total collection accumulation curve, disregarding grid type or time bin and including all marine assemblages, also points to saturation already by the mid-21st century (figs. S19 and S43).

### Terra incognita of deep time

Projecting the reconstructed extent of shallow-water regions of our paleomaps onto a present-day map, the intersection of underexplored regions with available global geological data highlighted areas that may yet hold undiscovered fossil information. The total extent of shallow-water areas varied hugely, ranging from 7,845,876 km^2^ (241.464 to 237 Myr ago, Ladinian) to 95,156,457 km^2^ (422.7 to 419.62 Myr ago, Pridoli) (table S7). For comparison, the modern analog has an extent of 35,953,832 km^2^.

**Fig. 3.**
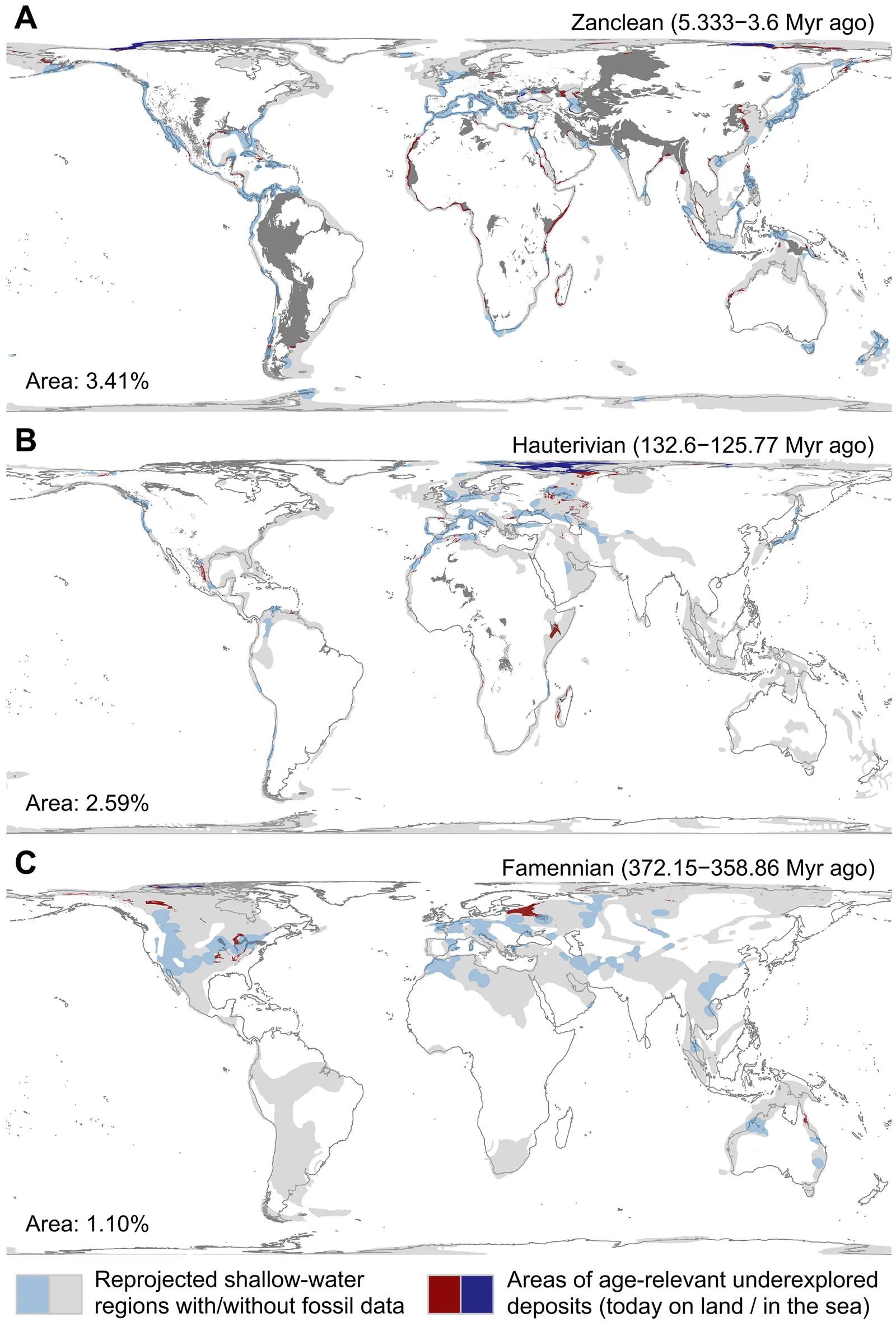
Reconstructed extents of past shallow-water areas and corresponding unexplored deposits. The maps correspond to the paleogeographic reconstructions in Fig. 1. (**A**) Zanclean (Early Pliocene, 5.333 to 3.6 Myr ago), with 3.41% of global marine shallow-water deposits potentially unexplored. (**B**) Hauterivian (Early Cretaceous, 132.6 to 125.77 Myr ago), with 2.59% unexplored deposits, with large areas in Russia and Africa. (**C**) Famennian (Late Devonian, 372.15 to 358.86 Myr ago), with 1.10% unsampled shallow-marine deposits, with notable gaps in North America and eastern Europe. All maps are based on the moderate filter; see figs. S20 and S21 for strict filter and full set of maps and figs. S44 and S45 for the results of the 49-bin approach. Note the gray curved lines in the southern hemisphere in (C) are a result of the reverse reconstruction of coordinates near the paleo-equator.

**Fig. 4.**
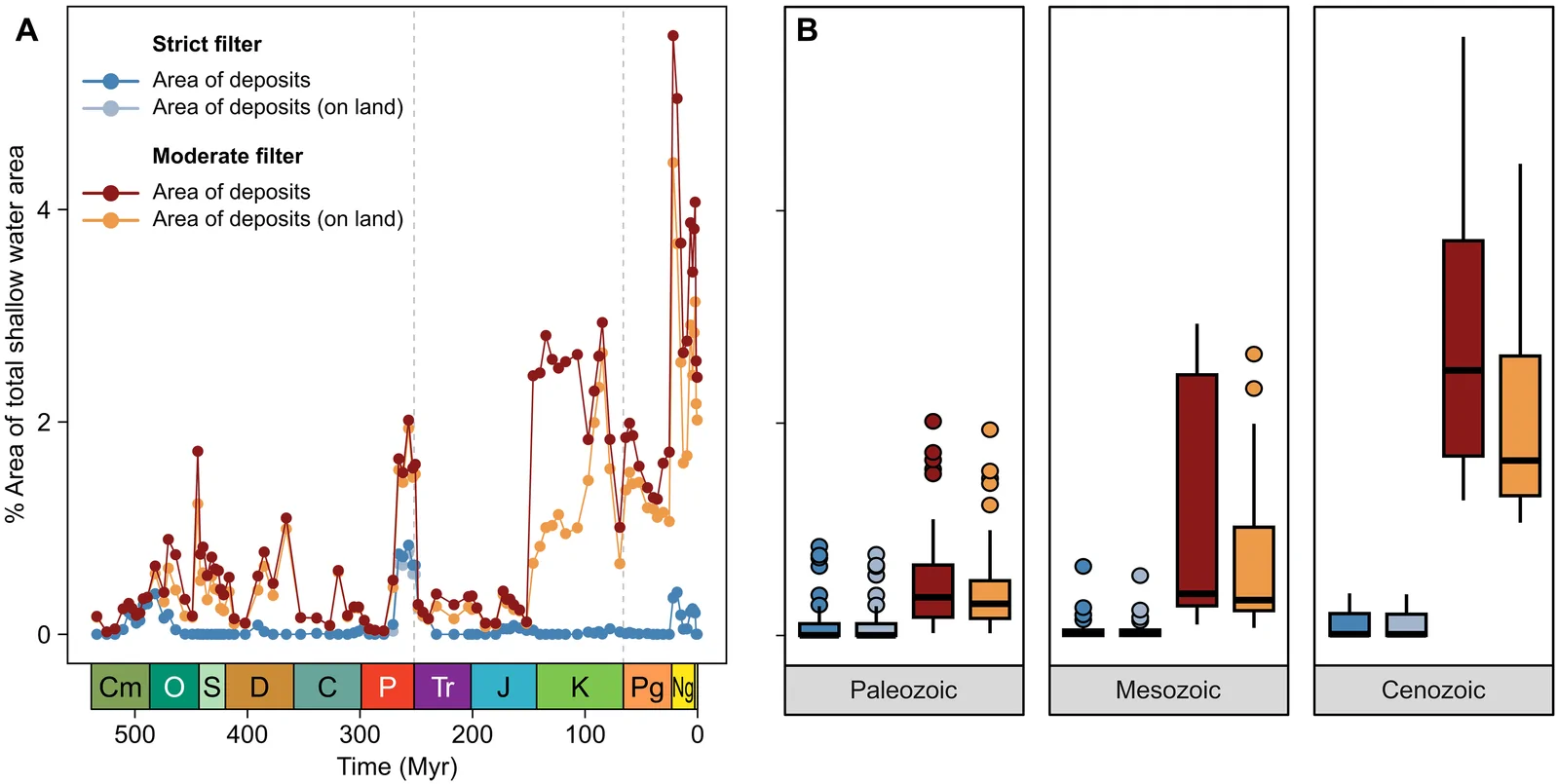
Areas of unexplored shallow-water deposits per stratigraphic stage. (**A**) Areas are given in percent relative to the reconstructed area of shallow-water regions, for two data filters and distinguishing total area and deposits on land. Absolute areas are summarized in fig. S22 and tables S8 and S9; see figs. S44 to S46 and tables S14 and S15 for the results of the 49-bin approach. (**B**) Areas aggregated by era, showing an average increase of unexplored areas from the Paleozoic to the Cenozoic.

Rock availability for undersampled shallow-water regions varied considerably across time bins and generally decreases with stratigraphic age (Figs. 3 and 4, figs. S20 to S22, and tables S8 and S9; see figs. S44 to S46 and tables S14 and S15 for the 49-bin approach), also depending on the filters we applied (see the Materials and Methods and Supplementary Materials for details). When using a strict filter, considering only strata that are of ascertained marine origin, only an average of 0.09% (0.13% for bins) of the unsampled shallow-water regions across all time intervals matched mapped strata of relevant stratigraphic age. With a more moderate filter, including deposits of potentially marine origin and broader age uncertainty, this value rises to 1.18% (0.99%). However, only 0.09% (0.11%) and 0.85% (0.69%) of the respective deposits are on land today. These comparatively low percentage values still translate to mean areas of 44,363 km^2^ (41,780 km^2^ on land) and 647,640 km^2^ (474,160 km^2^ on land) for undersampled shallow-marine deposits.

Most promising for further exploration are the Aquitanian (23.04 to 20.45 Myr ago; 0.35 and 5.64%, respectively, for the two different filters) and Burdigalian (20.45 to 15.98 Myr ago; 0.40 and 5.05%), followed by stages in Neogene, Quaternary, and Cretaceous. Most notably for the Paleozoic are the mid-Permian to earliest Triassic, for which even the strict filter yielded values of up to 0.84% (Fig. 4A), as well as the latest Ordovician (Hirnantian; 1.72% with the moderate filter; table S9). Comparatively little remains to be explored for the Cambrian Stage 2 (529.0 to 521.0 Myr ago; 0 and 0.2%), the early Permian stages Kungurian (283.3 to 274.4 Myr ago; 0 and 0.03%), Artinskian (290.1 to 283.3 Myr ago; 0 and 0.04%), and Sakmarian (293.52 to 290.1 Myr ago; 0.002 and 0.05%), the Cambrian Stage 3 (521.0 to 514.5 Myr ago; 0 and 0.05%), and the Serpukhovian (0 and 0.08%).

When binning into longer time bins, the two youngest intervals, i.e., the Tortonian–Pleistocene (0.49 and 6.23%) and Aquitanian–Serravallian (23.04 to 11.63 Myr ago; 0.41 and 4.21%) bins show the highest unexplored deposit areas, followed by time bins in the latest Jurassic to Early Cretaceous (figs. S44 to S46 and tables S14 and S15). In addition, the terminal Permian (259.51 to 251.902 Myr ago, Wuchiapingian–Changhsingian) may yield considerable added information with enhanced sampling. Similar to the stage-level analyses, apparently little remains unexplored for the Cisuralian (290.1 to 274.4 Myr ago, Artinskian–Kungurian, 0 and 0.01%; 298.9 to 290.1 Myr ago, Asselian–Sakmarian, 0.02 and 0.04%), the late Mississippian (Serpukhovian; 0 and 0.08%), the Early Devonian (410.62 to 393.47 Myr ago, Emsian; 0.03 and 0.10%), and the early Cambrian (Terreneuvian; 0 and 0.07%).

We found a significant positive correlation between unexplored areas and available deposit areas, which was moderately high when applying the strict filter (*r* = 0.718, *P* < 0.001 for stages; 0.484, *P* < 0.001 for bins) and more pronounced for the moderate filter (*r* = 0.828, *P* < 0.001 for stages; *r* = 0.784, *P* < 0.001 for bins).

## DISCUSSION

### Choice of paleogeographic model

We found that, for a few intervals, an elevated number of paleocoordinates reconstructed for apparently marine assemblages were plotting on dry land. To some extent, this is an unavoidable consequence resulting either from inaccurate stratigraphic or geographic information assigned to fossil collections or from variation in paleogeographic configurations in time and associated misplacement of points. We already minimized the effect of geographic biases, first by developing a method to buffer fossil collection points depending on tectonic movement speed and difference between collection and map age, and second by distinguishing inland depressions from marine embayments and epicontinental seas. However, there are still discrepancies in configurations. An example is the Western Interior Seaway, which is mapped as land from the latest Cretaceous (Maastrichtian) onward in the “Paleomap” model used here [following Salles *et al.* (*32*)] but was still open until the early Paleocene according to latest reconstructions (*33*).

For some time intervals, a number of fossil collections yielded no paleocoordinates at all, most clearly seen for the Pleistocene. The relatively high number of missing paleocoordinates (35.9% for the Chibanian–Late Pleistocene and 22.6% for the Calabrian) likely results from the high frequency of near-shore and island records for the Pleistocene with no equivalent in the paleo-rotation models.

These issues potentially influence our estimated coverages of shallow-water areas, similar to what was discussed by a recent study on the impact of different paleogeographic models on paleobiological studies (*34*). With the constant advance of paleogeographic models, specifically the higher temporal and spatial resolution, informed also by the integration of fossil information, as well as improved dating of geological strata, such biases could be minimized in the future. Nevertheless, our models run on the entire marine dataset give comparable results compared to those based on the shallow-water collections (figs. S13 to S18 and S37 to S42 and tables S6 and S12), precluding a major bias of our predictions and interpretations.

### Toward terra cognita

Our sampling effort models indicate that over the past decades, little or no data have been collected from previously unsampled regions for the majority of time periods, or in other words, sampling occurred predominantly in already-known areas. While improving sampling in known areas certainly contributes to our understanding of fossil composition, abundance, and population structure, considerable effort is still needed to push the geographic frontiers.

Our analytical pipeline provides spatially explicit guidance on where these frontiers lie and how tractable they may be. For several stages, our logistic or logistic-like models predict continuously increasing future collection effort toward a larger geographic coverage of shallow-marine areas (Fig. 2 and fig. S6 to S12). Expectedly, these mostly concern younger time intervals, which are represented by a larger rock area globally (*35*), much of which still remains to be explored (Figs. 3 and 4 and figs. S20 to S22). For older intervals, particularly spanning the Cambrian to the Jurassic, the models indicate near or full saturation of sampling effort. In those cases, targeted fieldwork in the specific frontier regions identified here is especially warranted, alongside careful investigation of regional geological maps to account for potentially inexact mapping, stratigraphically coarse coverage, or imprecisely constrained depositional environments.

The reconstruction of undersampled shallow-water areas revealed considerable spatial and temporal variability in the potentially explorable rock record. In part, this likely reflects the uneven spatial resolution of available geological databases. Mapped deposits for the North American continent, for instance, are accessible at a substantially higher spatial and temporal resolution via Macrostrat compared to other continents. We expect that an increased spatial and or temporal resolution for other continents would affect the available area of undersampled regions. Finer spatial resolution is likely to decrease the area due to a more detailed subdivision of geological units, while enhanced stratigraphic resolution will likely increase the reconstructed areas, particularly when applying a strict filter.

The paleontological sampling completeness of North American sedimentary rocks was previously assessed by Peters and Heim (*29*), who, using the same combination of Paleobiology Database and Macrostrat data, showed that, on average, 23% of sediment coverage area across the continent has yielded fossil information. A subsequent global extension of this approach, using bedrock geological map data from Macrostrat to predict expected fossil occurrence yields, estimated a shortfall of ∼639,000 occurrences from outside North America and Europe in the Paleobiology Database alone (*30*). Our results are consistent with this picture and extend it by offering a time-resolved, spatially explicit reconstruction of where the most tractable frontier regions to obtain shallow-water marine fossil assemblages are situated.

Particularly low proportions of geologically accessible, unexplored deposits were identified for the Paleozoic through early Mesozoic. Most notably are intervals in the early to mid-Cambrian, latest Ordovician, Early Devonian, most of the Carboniferous through early Permian, Early Triassic, and parts of the Early Jurassic, as well as the mid-Late Jurassic, all of which range below 0.2% of explorable shallow-marine deposits, even when including a larger range of uncertainty as to environmental origin and age range (Fig. 4A, fig. S22, and table S15). This somewhat counterintuitive finding reflects in part the limited global preservation of older shallow-marine deposits, which is shown by the correlation between available outcrop area and unexplored area. Especially for the abovementioned intervals, the total mapped areas are comparatively small throughout (tables S8 and S9). Similarly, the jump to larger areas to be explored in the latest Jurassic parallels an increase in available outcrop area (fig. S23). In addition, varied research focus for selected intervals, such as the Cambrian radiation, may contribute to time bins being differently well-sampled. The available shallow-marine area per time interval and, accordingly, the inferred extent of matching unsampled deposits can also be affected by the more liberal mapping due to higher uncertainties in paleogeographic reconstructions, particularly in the distant past (*36*). Additionally, strata may be disproportionately represented in global geological databases given economic interest; for example, the Upper Jurassic and mid-Cretaceous have yielded the majority of hydrocarbon reservoir rocks worldwide (*37*). Even in the Paleobiology Database, a more dedicated focus on certain regions or time intervals by individual data providers (e.g., around major extinction intervals) potentially affects the reconstructed areas.

### Where to look again

Areas determined as underexplored in our study may relate to formations that have in fact been sampled, but either not in the specific region under consideration or with results not yet indexed in the Paleobiology Database. Our sampling curves show historical research effort, not intrinsic completeness of the fossil record. In part, this discrepancy may reflect the ongoing and, by necessity, incremental effort to populate this comprehensive database. More broadly, however, the database itself, and our global view of the fossil record in general, is subject to well-documented structural biases. Already, Allison and Briggs (*38*) stated that paleobiogeographic sampling is skewed toward Europe and North America, which is reflected also in the Paleobiology Database (*25*). This spatial bias has a deep historical and socioeconomic dimension: Researchers in high- or upper middle–income countries contribute ∼97% of all fossil occurrence data, and the top-contributing countries have disproportionately conducted fieldwork abroad (*39*). The resulting heterogeneous spatial sampling can create spatiotemporal shifts in reconstructed richness and biogeographic affinities that may substantially over- or underestimate changes on a global scale (*25*). The broader structural inequalities in global paleontology, encompassing field access, institutional infrastructure, and publishing means, constitute a major and still largely unresolved challenge to obtaining an unbiased view of the fossil record (*40*). While our reconstructions are certainly biased by these aspects, they also highlight the blank spots on the map and the need to complete them, either by assembling published data so far unavailable through datasets or by producing new data through fieldwork.

Beyond the published literature, a substantial volume of fossil data resides in unpublished museum collections worldwide, representing a major untapped resource for expanding the spatial and taxonomic scope of the known record (*41*). These considerations suggest that the unexplored areas reconstructed here may be more limited in extent than our models indicate—not because the geology is unfavorable, but because relevant collections exist, yet remain invisible to global databases.

The geographic distribution of underexplored areas identified here has a strong practical implication: It points beyond North America and Europe, which dominate existing databases (*25*, *39*), toward regions of the Global South where both the geological and paleontological records remain comparatively understudied. Ongoing international initiatives such as Deep-Time Digital Earth (https://ddeworld.org/) (*42*), which aims to harmonize and expand existing deep-time Earth databases following FAIR data principles (*43*) and to integrate previously inaccessible geological data from underrepresented regions, hold considerable promise in this regard. As data quality and quantity advances, and fossil occurrences become more comprehensive databased, the frontier regions identified here are likely to shrink.

Our study contributes to addressing these challenges by providing spatially and temporally explicit maps of where the frontiers of the fossil record lie, translating geological potential into actionable research targets. These can guide effort on multiple levels: systematically mining the existing literature to complete occurrence databases, surveying and digitizing unpublished museum collections, or prioritizing geographic areas for fieldwork on genuinely unsampled deposits. We found that sampling is more saturated in older rocks, while the fossil record for younger intervals for which sampling is already good, i.e., for the latest Jurassic through Cenozoic, still has potential for geographic expansion. On the other hand, our data illustrate that for many time bins, especially in the Paleozoic through early Mesozoic, the limits of what is possible in terms of exploring new sites in previously unknown regions have been exhausted. This is illustrated by both the low area coverage of age-relevant strata recovered through Macrostrat as well as the historical sampling trajectories indicating stagnation in many cases. Future efforts should therefore target essentially underrepresented regions and time bins. This observation could also be of great relevance to funding strategies and the design of new studies. Given the geographic distribution of the identified frontier regions and those not identified yet, investment in research capacity in currently underrepresented countries will be essential to realizing this potential.

## MATERIALS AND METHODS

### Dataset

We downloaded all available data on the metazoan fossil record from the early Cambrian to the latest Pleistocene from the Paleobiology Database (https://paleobiodb.org/) on 5 May 2026. All data handling, map processing, and analytical steps were carried out in the statistical environment R v.4.4.2 (*44*). A full list of R packages used in this study (including references) is provided in table S1. All spatial operations were performed in Behrmann equal-area projection.

Data were filtered for marine, published occurrence records. Because nonmarine taxa are occasionally preserved in marine environments, we applied taxonomic filters following Bush and Bambach (*27*) and Close *et al.* (*6*); for Mollusca, we used own lists following Neubauer (*45*) for gastropods and MolluscaBase (*46*) for bivalves. Using the R package “CoordinateCleaner,” we filtered for spatial issues, excluding collections with invalid coordinates (i.e., nonnumeric or outside bounds) and with identical latitude and longitude values. Records at the resolution of “basin” were excluded following Close *et al.* (*6*). Collections without stratigraphic ages and those from Holocene deposits were omitted as well. To avoid too strong temporal biases, records with uncertainty ranges higher than 10 Myr were omitted (*15*), except for records that exactly match stratigraphic stages (otherwise, all records for, e.g., the Norian with an age range of 21.6 Myr would be dropped).

### Recalibrating stratigraphy, time-binning, and paleogeographic maps

To ascertain sound age data, stratigraphic information retrieved from the Paleobiology Database was updated where necessary using the R package “fossilbrush” (*47*), with a few specific changes to the default settings: While substage boundaries of international chronostratigraphic units as well as regional units were updated with the in-built default settings, stage boundaries were recalibrated following the latest international chart (December 2024; https://stratigraphy.org/chart/). This was necessary because the default settings referred to an outdated version of the international chronostratigraphic chart.

Analyses were carried out on the level of stratigraphic stages, whereas the Chibanian (Middle Pleistocene) and Late Pleistocene were combined into a single unit given their comparatively short duration (0.1173 and 0.645 Myr, respectively), resulting in a total of 98 intervals (table S2). Fossil collections were allocated to the time bin with the highest overlap of their age range.

In addition, the same set of analyses was run for 49 intervals of roughly equal duration while retaining stage boundaries, in order to test for the influence of a coarser temporal resolution on the spatial distribution of data and reconstructed unexplored areas. We largely followed the concept of Kiessling (*48*) while adapting the bin boundaries where necessary: (i) We accommodated recent changes in age boundaries, and (ii) we used international stage boundaries throughout, while Kiessling (*48*) had partly divided bins after regional stages (e.g., for the Carboniferous and Ordovician). The time bins defined here are summarized in table S3.

Each bin was assigned a paleogeographic map from the collection of reconstructions of Salles *et al.* (*32*). Generally, the “nearest” map in terms of a bin’s mean age was chosen; only for the Middle Triassic for the 49-bin approach, we used the map for 245 Myr (Anisian) instead of 240 Myr (Ladinian) when a major regression drastically reduced the extent of shallow-water regions globally (*32*), causing multiple collections of marine fossil assemblages to plot on dry land.

### Dealing with geographic uncertainty

Paleocoordinates were calculated with the function “palaeorotate()” of the R package “palaeoverse” (*49*). We used the Paleomap global plate model, also to match the paleogeographic maps of Salles *et al.* (*32*), based on the mean of a fossil collection’s age range.

Uncertainties in geographic coordinates and stratigraphic age of fossil collections as well as paleogeographic configurations can lead to an incorrect reconstruction of paleocoordinates and, accordingly, a wrong interpretation of the depositional environment. To account for this, we defined a buffer around each point on a paleomap. Considering plate tectonic movement, the correct reconstruction of a fossil locality’s paleocoordinates is affected by the difference between a collection’s stratigraphic age and the map’s age used for reconstruction. Therefore, we chose a buffer depending on this age difference, applying a median plate movement speed of 4 cm/year (+20% uncertainty range) as estimated from deep-time plate kinematic reconstructions (*50*). To incorporate general geographic and stratigraphic uncertainty, we set a minimum buffer of 250 km, matching the cell diameter of the smallest grid defined above.

### A method to determine shallow-water regions

Shallow-water regions were defined as marine areas down to a depth of −600 m following Close *et al.* (*6*) and using the high-resolution (0.1° × 0.1°) digital elevation models of Salles *et al.* (*32*). These models, however, identified a series of inland depressions below sea level, inflating the actual area of shallow-water regions when strictly applying a threshold by bathymetry. Therefore, we developed a method to distinguish between actual marine regions and inland depressions:

1) Digital elevation models were cropped to the interval −600 to 0 m and smoothed using the function “focal()” of R package “terra” with a moving window size of three cells choosing the most frequent value (mode) and omitting NA values.

2) We then identified connected regions with the function “patches(),” considering cells as adjacent if occurring in a 3 × 3 neighborhood (including diagonals). Patches smaller than 5000 km^2^ were discarded. This threshold corresponds approximately to the size of a 1° × 1° grid cell at mid-latitude (c. 71 × 71 km at 50°) and is still considerably smaller than major inland water bodies. To test for the impact of that choice, we ran additional test analyses with 1000, 2500, 10,000, and 25,000 km^2^ for all 82 maps used in the analyses. The summed areas of the patches marked as “isolated, nonmarine” vary slightly and only for a few time intervals but remain in the same order of magnitude (fig. S47). Similarly, the total shallow-marine areas are only slightly affected. Area variation (quantified as standard error) is on average higher for Mesozoic and Cenozoic maps, but variation still remains in the order of 10^4^ km^2^ compared to an overall range of shallow-marine areas of 7.5 × 10^6^ to 97.2 × 10^6^ km^2^ (fig. S48 and table S16).

3) To account for inexactness inherent to any paleoelevation model, we determined whether a patch should be considered as connected to the ocean if a patch buffered by five cells touched the global ocean polygon. This way, we avoid discarding large marine areas that are cut off from the global ocean (or only apparently so).

4) Patches were marked as inland, nonmarine if (i) the abovementioned versions were not connected to the ocean, (ii) when in a surrounding ring of 10 cells, the average elevation was at least 10 m, and (iii) the minimum distance of the closest point to a marine patch (including those included under point 3) was more than 1000 km. That way, marginal seas not directly connected to the open ocean were still considered marine.

By applying this method, we accounted for major epicontinental seas (e.g., the Caspian Sea) but discarded depressions further inland. The method involves a certain degree of subjectiveness concerning the thresholds chosen for patch size and distance, but visual inspection of the resulting maps showed distinctly improved results compared to those created with a classical bathymetric cutoff.

### Spatial grids

The (buffered) fossil collections were binned into equal-area hexagonal grids. Because results based on spatial grids tend to vary depending on the grid type, we used three different grid sizes (maximum diameter of grid cell: 250, 500, and 750 km) and two grid types (pointy-topped versus flat-topped hexagons). Thereby, we minimize a bias of our estimates of shallow-water coverage and discovery rate (see below) from sticking to a predefined grid. For each time bin and grid type, we calculated the total number of cells containing fossil data and the number of cells with data overlapping shallow-water regions.

### Modeling region discovery rate

For each time bin and grid type, we modeled historical sampling effort as a cumulative function of the number of grid cells gained over the years. To assess whether sampling area increases over time, and to evaluate whether future sampling is expected to access new geographic regions—and thus increase the accessible fossil record—only newly sampled cells in each year are accounted for, i.e., the first occurrence of any fossil collection in that cell within a given time bin. Collections from the years 2024 to 2026 were excluded prior to the analyses to avoid an artificial saturation simply resulting from delayed data entry.

The best-fitting model for each time bin and grid type was chosen from a set of 11 models of five types (linear, exponential, asymptotic, logistic, and Gompertz), following the recent approach of Li *et al.* (*51*) with the function “drm()” of the package “drc.” No model was calculated if fewer than 10 years were sampled. Models were compared using the Akaike information criterion (AIC). All models within ΔAIC = 2 of the best model were treated as equally likely; hence, in some cases, more than one model had to be considered. Based on these models, we predicted future sampling effort until the year 2100.

We estimated sampling completeness for a given time bin and grid type, based on the difference between the maximum observed number of grid cells and the one predicted for 2100. Given the uncertainty inherent to such models, the average predicted value was in some cases slightly below the observed one; in that case, the difference was treated as 0. To model the overall trend of the coverage through time, we fitted a generalized additive model (GAM) computed with the package “mgcv” (*52*, *53*). As we deal with percentages, we chose a quasibinomial model under a restricted maximum likelihood (REML). The smooth basis dimension *k* was set to 97 (98-stage dataset) and 45 (49-bin dataset) after assessing the model via “gam.check()” to ascertain a *k* index close to 1 (*53*).

While we focus on collections in shallow-water regions, which are expected to hold the majority of fossil diversity, we also ran these analyses with the entire marine fossil record. In addition, for completeness, we ran the same 11 models for the total collection accumulation curve (irrespective of any grid or time bin and including all marine assemblages) (figs. S13 to S18 and S37 to S42).

### Estimating the extent of unexplored shallow-water regions

We reverse-reconstructed the extent of shallow-marine regions for each time bin on the present-day map. This step was necessary to be able to compute the relative areas of total shallow-marine regions and unexplored deposits per bin.

Shallow-marine regions (buffered by 100 km for uncertainty, compare discussion above) were converted into a dense raster of equally spaced points placed at a distance of 25 km. Points that intersected shallow-marine regions but not areas defined by the buffered points containing fossil data were marked as unexplored, shallow-marine. For each of these points, we reconstructed its modern location using the function “reconstruct()” of the R package “rgplates.” The same procedure was done for points overlapping regions that contain fossil data for comparison.

The reconstructed point clouds (both unexplored and explored) were converted back into polygons using the following routine, which was tested in several iterations to obtain most reasonable representation of areas, i.e., finding the optimal middle course between interpolating and oversplitting. Points were first buffered each by a 100-km radius to connect points stretched by the reverse reconstructions; overlapping circles were merged, and the resulting polygon buffered inversely by a 75-km radius to not overestimate the outer boundary; polygon geometry was simplified with a tolerance of 6250 km (i.e., a fourth of the original point distance) and subsequently smoothed using Chaikin’s corner cutting algorithm and three iterations with the package “smoothr” (*54*) to remove edges; a final buffer of 6250 km ascertained that neighboring polygons of explored and unexplored reconstructed regions touched.

Resulting polygons for unexplored shallow-marine regions were intersected with data on global bedrock geology as derived from the Macrostrat Database (*55*) available through the work of Ye and Peters (*30*). Unfortunately, the dataset comes with certain limitations, such as unknown or unspecified environmental origin or long age uncertainty ranges. We first retrieved all available information for sedimentary units on lithology and environment via the command “get_map_outcrop()” from the R package “rmacrostrat” (*55*). Output was screened using a text filter for keywords marking marine and nonmarine environments in any descriptive and name columns. For further analyses, we applied two filters: (i) In a stricter approach, we considered only units that were ascertained of marine origin and have a maximum age range of 25.9 Myr (i.e., the maximum duration of the used time bins). (ii) Using a moderate filter, we also included deposits of uncertain origin (many of which are likely to be marine) as well as a longer age uncertainty range, i.e., the mean duration of Phanerozoic periods (44.9 Myr). Especially in regions that are stratigraphically poorly resolved in the dataset, this coarser filter is essential to highlight possible areas for more detailed exploration.

For each time bin, we calculated the areas of (i) shallow-water regions in the original paleogeographic map, (ii) the reconstructed extent thereof on the present-day map (which may differ as a result of plate tectonics), (iii) the shallow-water regions that contain fossil data, (iv) the shallow-water regions that overlap with deposits of relevant age (for both filters), as well as the percentage thereof compared to the total reconstructed extent [see (ii)], (v) the regions overlapping with deposits of relevant age that are currently on land or in the sea, as well as (vi) the total area covered by Macrostrat data for a given time bin (fig. S23). For comparison, we calculated the area of the analog modern shallow-water extent (−600 to 0 m) based on the GEBCO_2023 global elevation model (*56*).

### AI usage statement

During the preparation of the code underlying this study, the authors used Anthropic’s Claude Sonnet 4.5 and 4.6 and OpenAI’s ChatGPT 4.o as a coding assistant for R-based spatial and statistical analyses. The AI was interrogated through iterative dialog in which specific coding problems were described, error messages and nonfunctioning code were shared, and proposed solutions were tested and refined across multiple exchanges.

Key areas where Claude’s assistance was sought are (i) spatial operations in R, including the calculation of polygon areas using the “sf” and “terra” packages, map projections, and the intersection of fossil occurrence collections with georeferenced geological layers; (ii) distinguishing inland topographic depressions and lakes from shallow epicontinental seas in raster and vector-based workflows; (iii) handling and processing API requests to external databases (e.g., GPlates), including solutions to timeout errors and rate-limiting issues; (iv) handling and filtering Macrostrat data; and (v) formatting of figures and map layouts, including legend design, color scale adjustments, and plot annotation in “ggplot2.” ChatGPT was queried concerning (i) initial concepts on how to distinguish inland depressions; (ii) formatting the input dataset for the models on historical sampling effort; and (iii) buffering collection points to account for geographic uncertainty.

In each case, the scientific framing and objectives were provided, the correctness and appropriateness of suggested code was evaluated, and final decisions about implementation were made. The AI did not contribute to study design or interpretation of results.

## Supplementary Materials

### The PDF file includes:

Figs. S1 to S48 Tables S1 to S16 Legend for data S1 References
