# Supplementary material for: Staking out the frontiers: The limits of the metazoan shallow marine fossil record
Source: Sci Adv. 2026 Sep 23;12(39):eaeh3920. doi: 10.1126/sciadv.aeh3920 (PMC13600244; doi:10.1126/sciadv.aeh3920)
Supplement: Supplementary file 1 [file sciadv.aeh3920_sm.pdf]

Science Advances
AAAS

Supplementary Materials for

Staking out the frontiers: The limits of the metazoan shallow marine fossil record

Thomas A. Neubauer and Mathias Harzhauser

Corresponding author: Thomas A. Neubauer, neubauer@snsb.de

Sci. Adv. **12**, eaeh3920 (2026)
DOI: 10.1126/sciadv.aeh3920

The PDF file includes:

Figs. S1 to S48
Tables S1 to S16
Legend for data S1
References

Other Supplementary Material for this manuscript includes the following:

Data S1

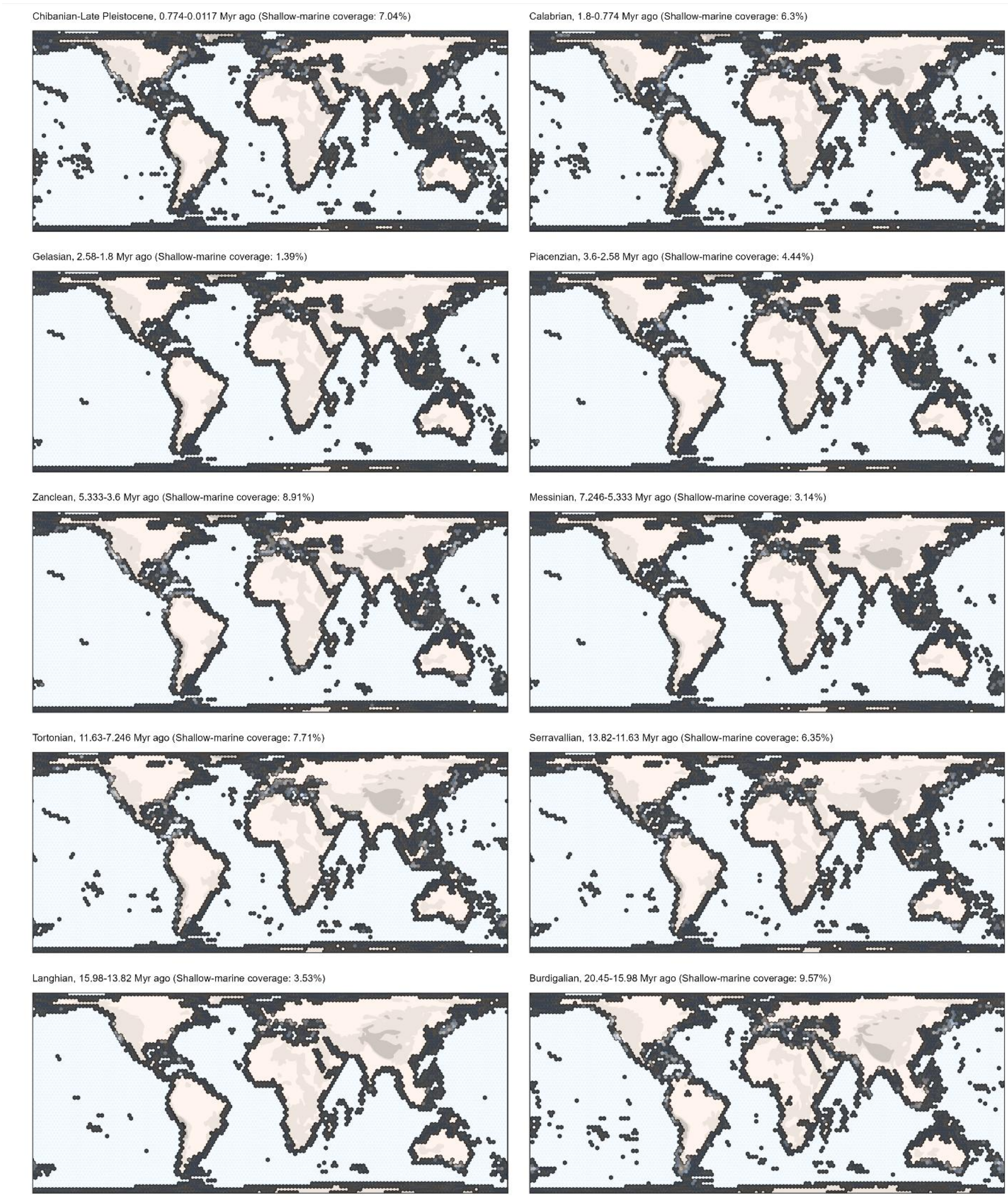

Fig. S1.

Paleogeographic maps showing information density on shallow-marine metazoans as light hexagons for the 250 km pointy-topped grid. The number of fossil collections per grid cell is displayed as relative transparency (see Figure 1 in main text for legend). Corresponding data on coverage is summarized in table S4.

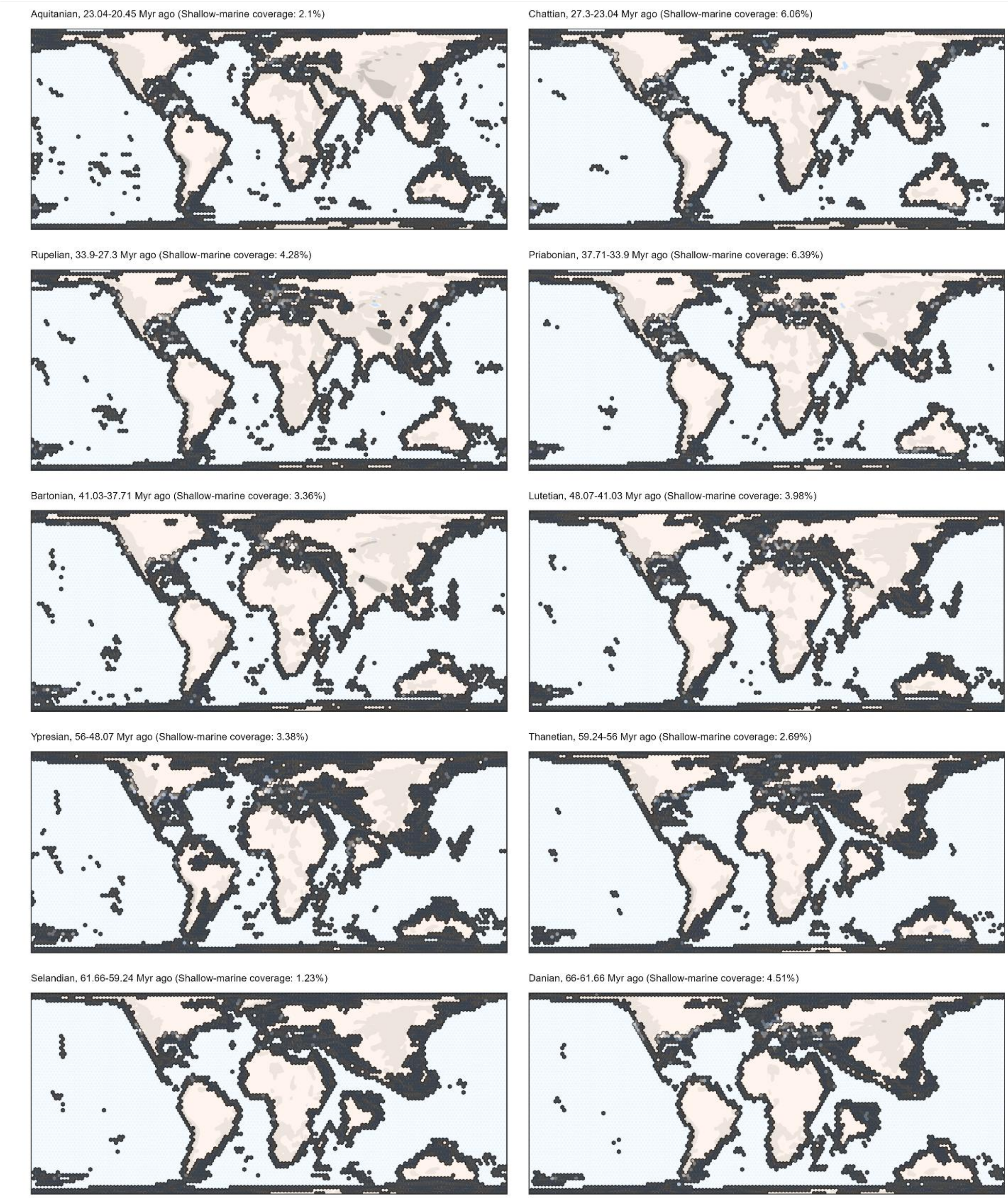

Fig. S1. Continued.

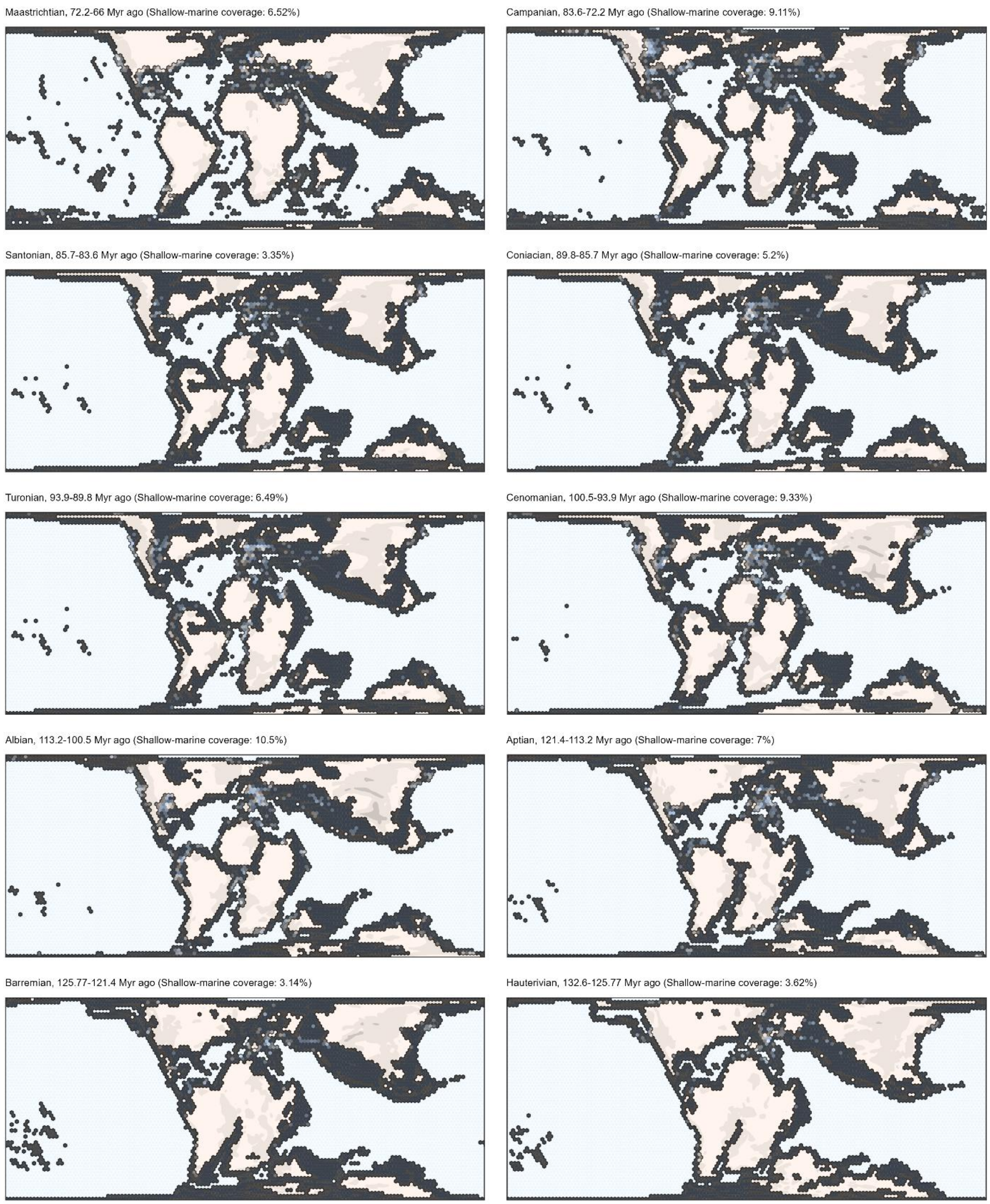

Fig. S1. Continued.

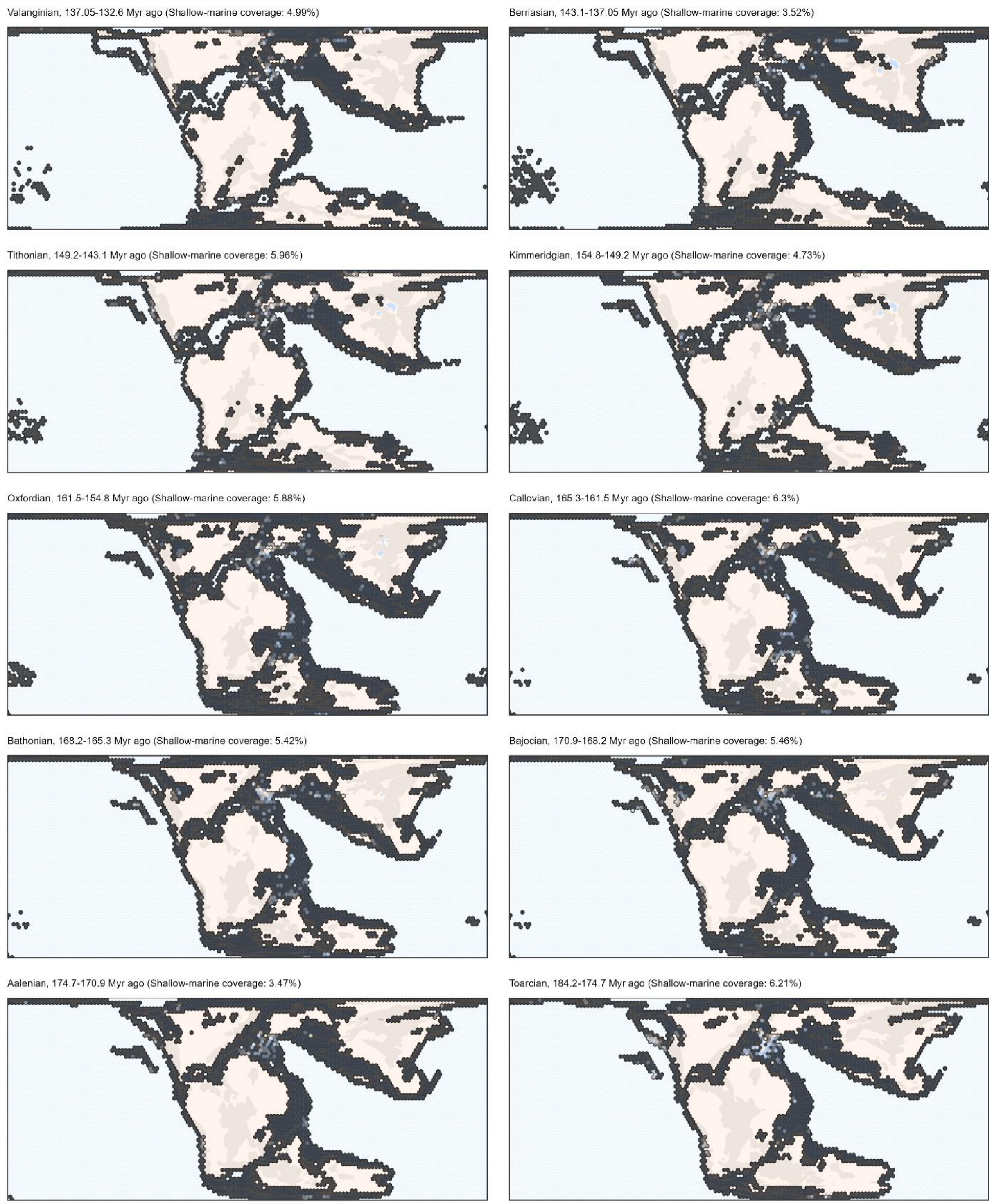

Fig. S1. Continued.

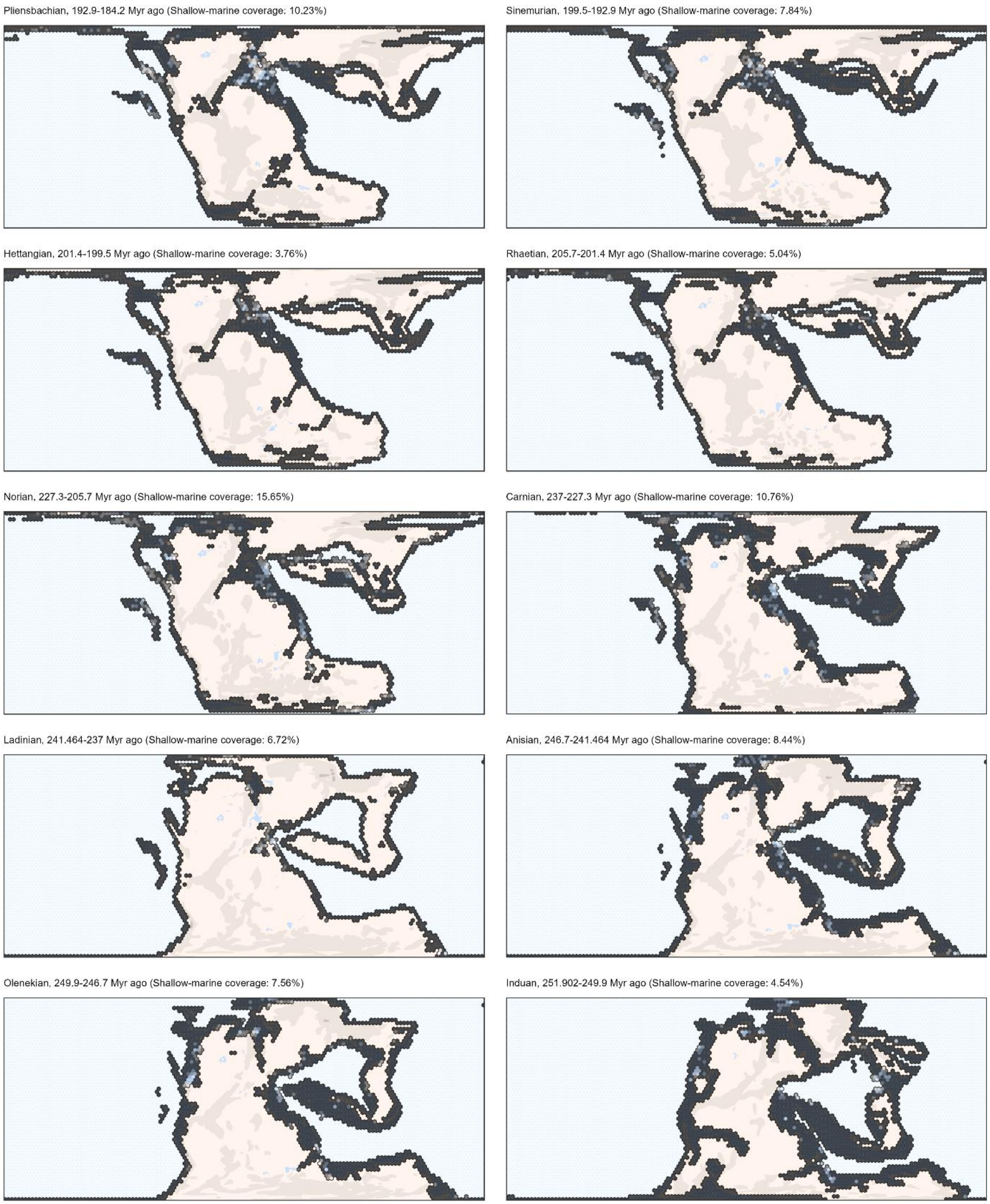

Fig. S1. Continued.

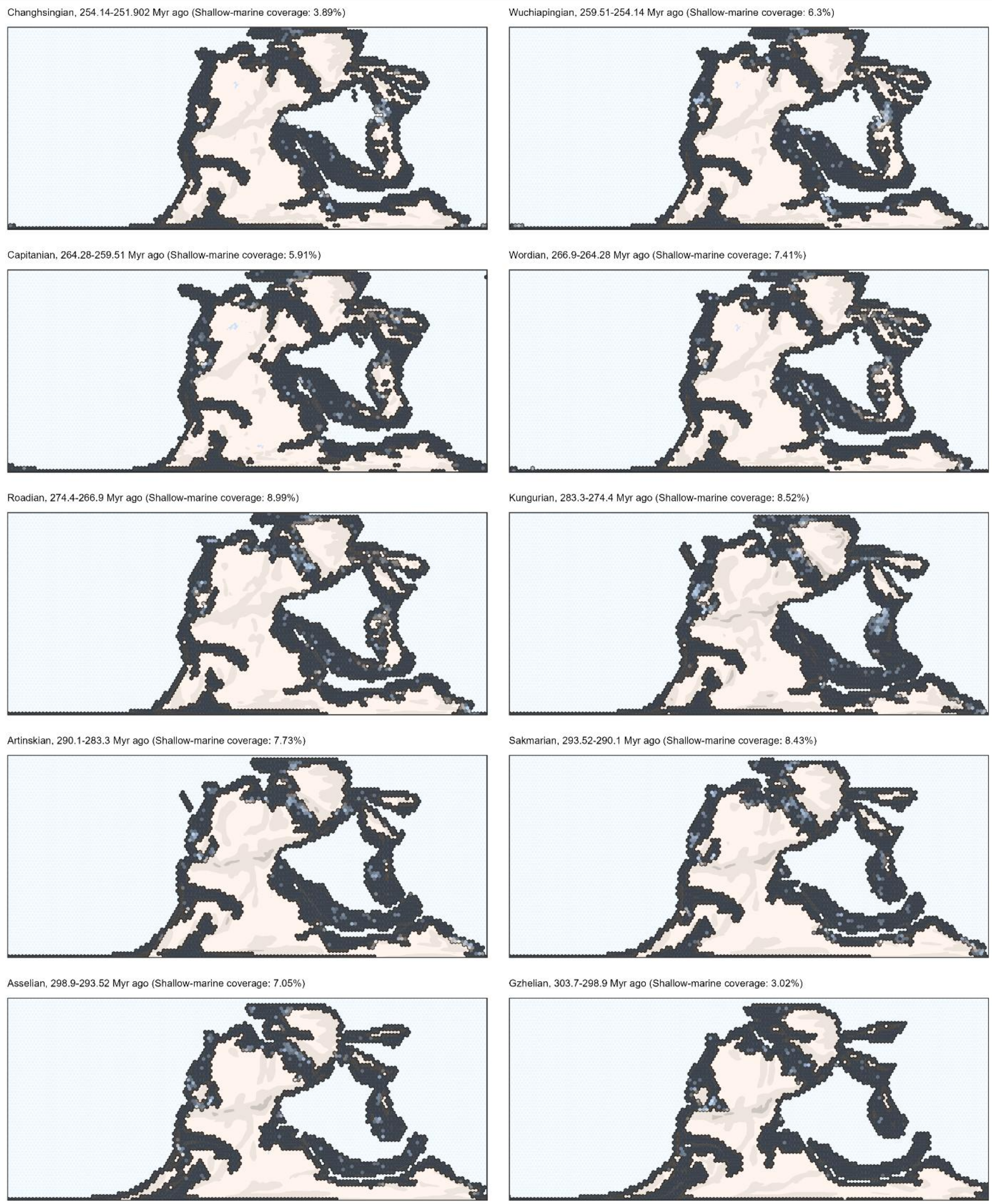

Fig. S1. Continued.

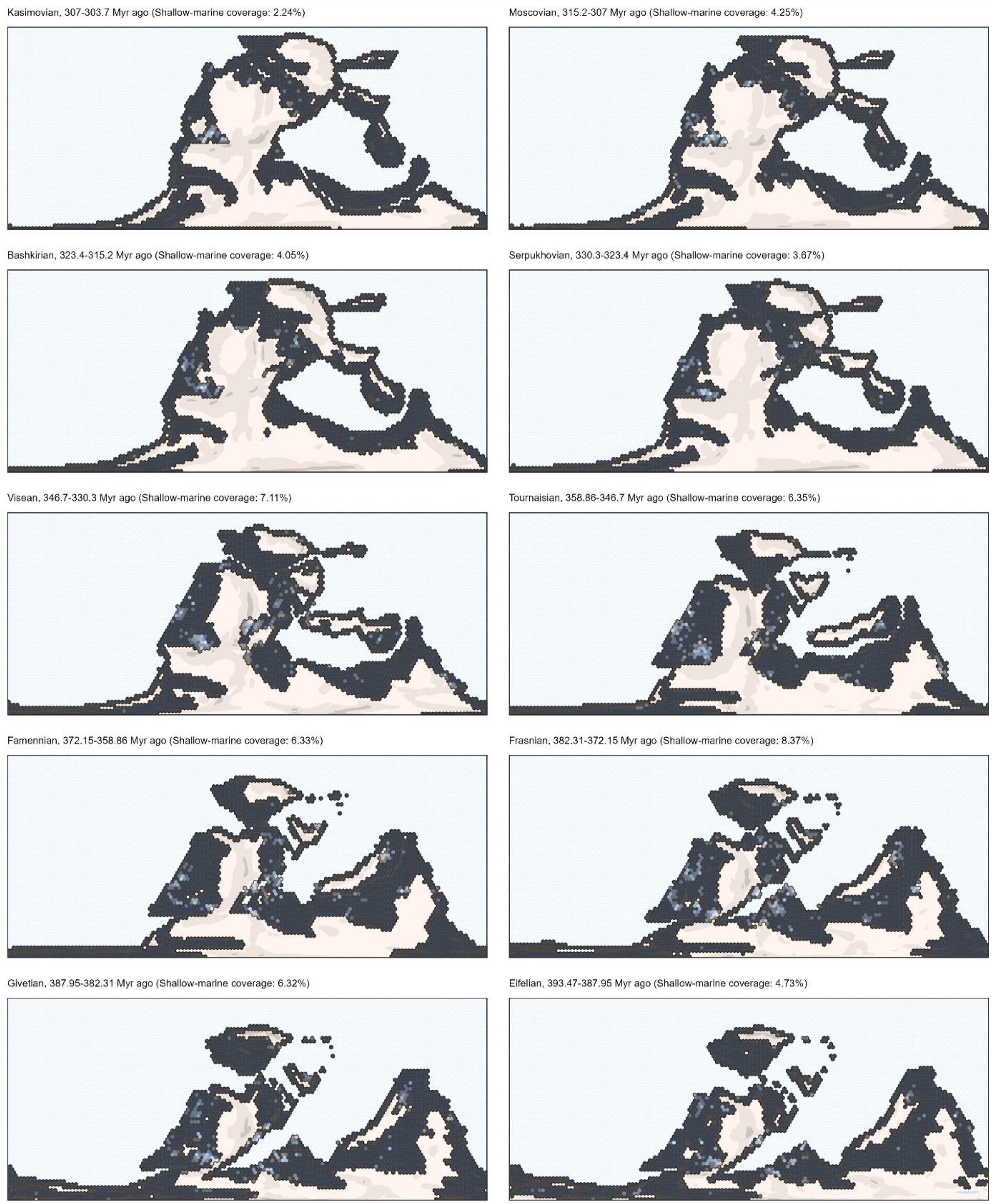

Fig. S1. Continued.

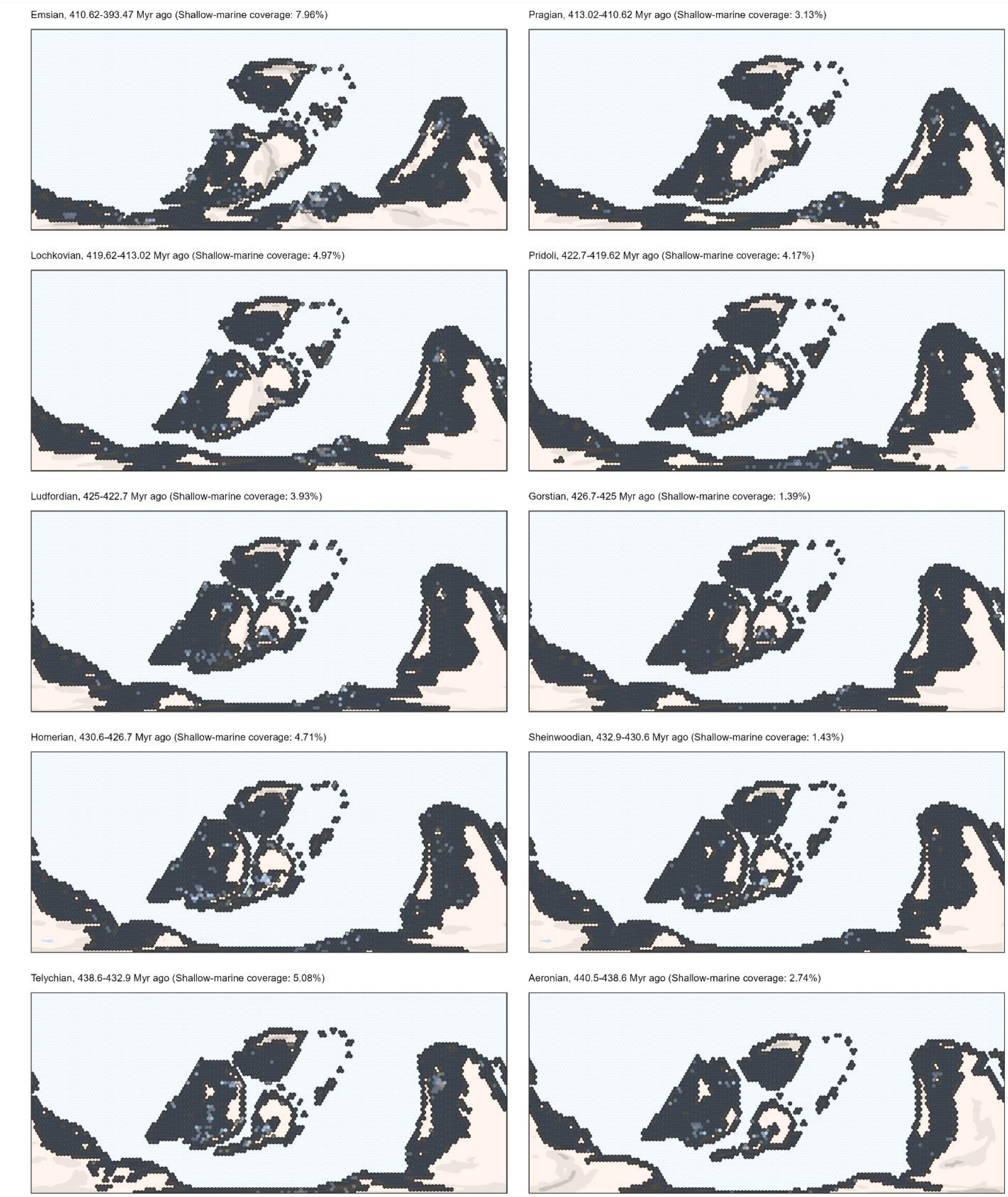

Fig. S1. Continued.

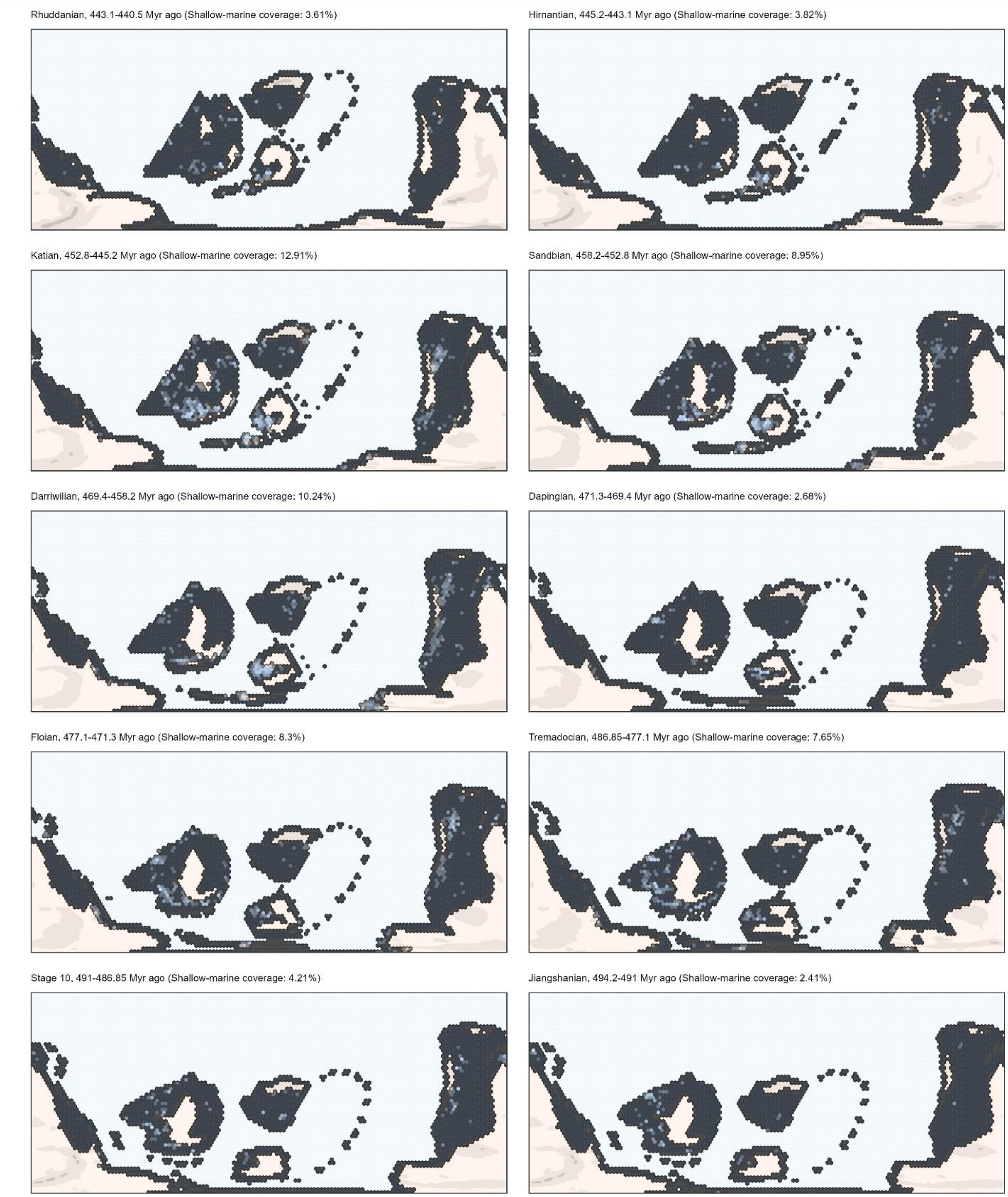

Fig. S1. Continued.

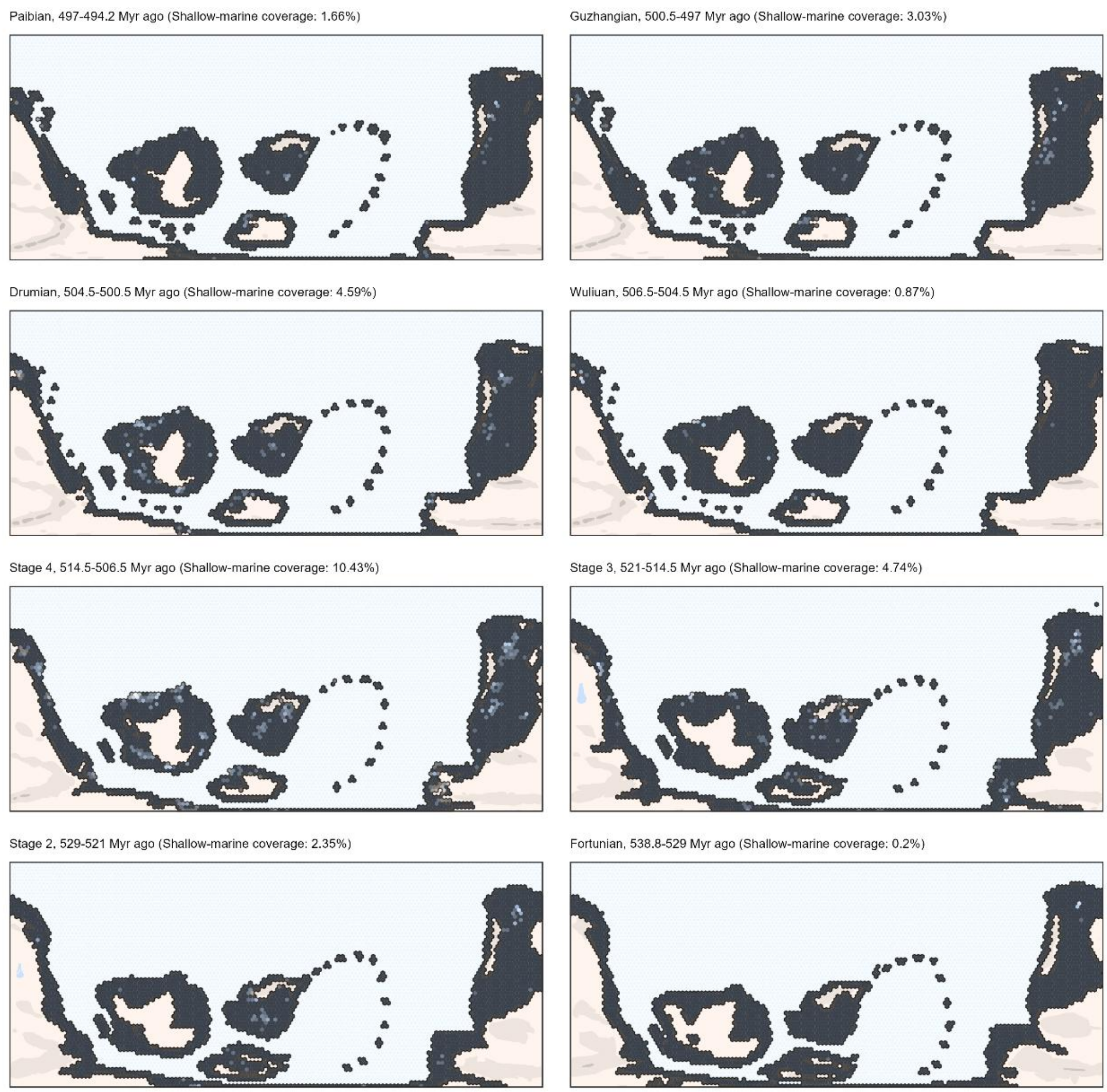

Fig. S1. Continued.

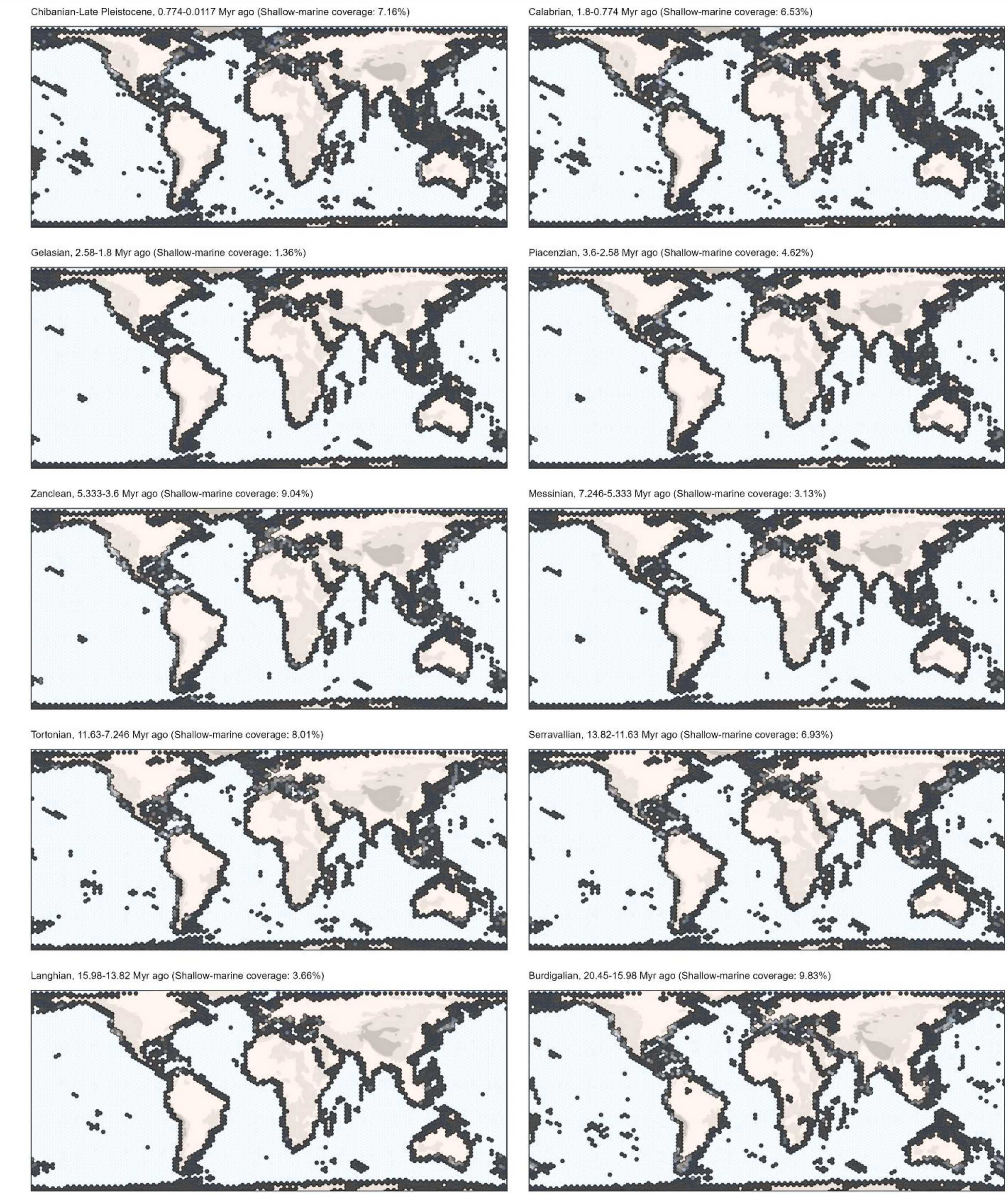

Fig. S2.

Paleogeographic maps showing information density on shallow-marine metazoans as light hexagons for the 250 km flat-topped grid. The number of fossil collections per grid cell is displayed as relative transparency (see Figure 1 in main text for legend). Corresponding data on coverage is summarized in table S4.

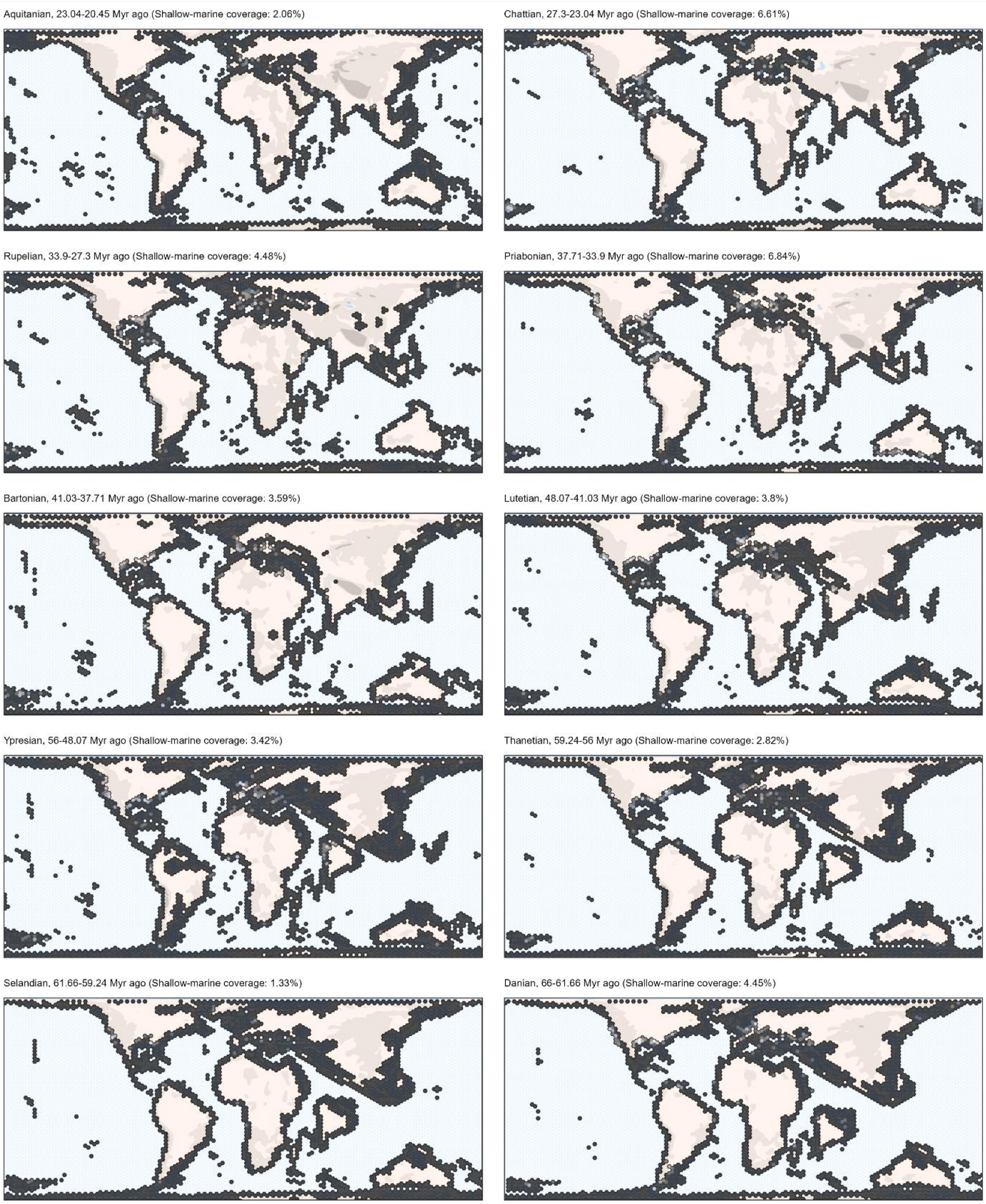

Fig. S2. Continued.

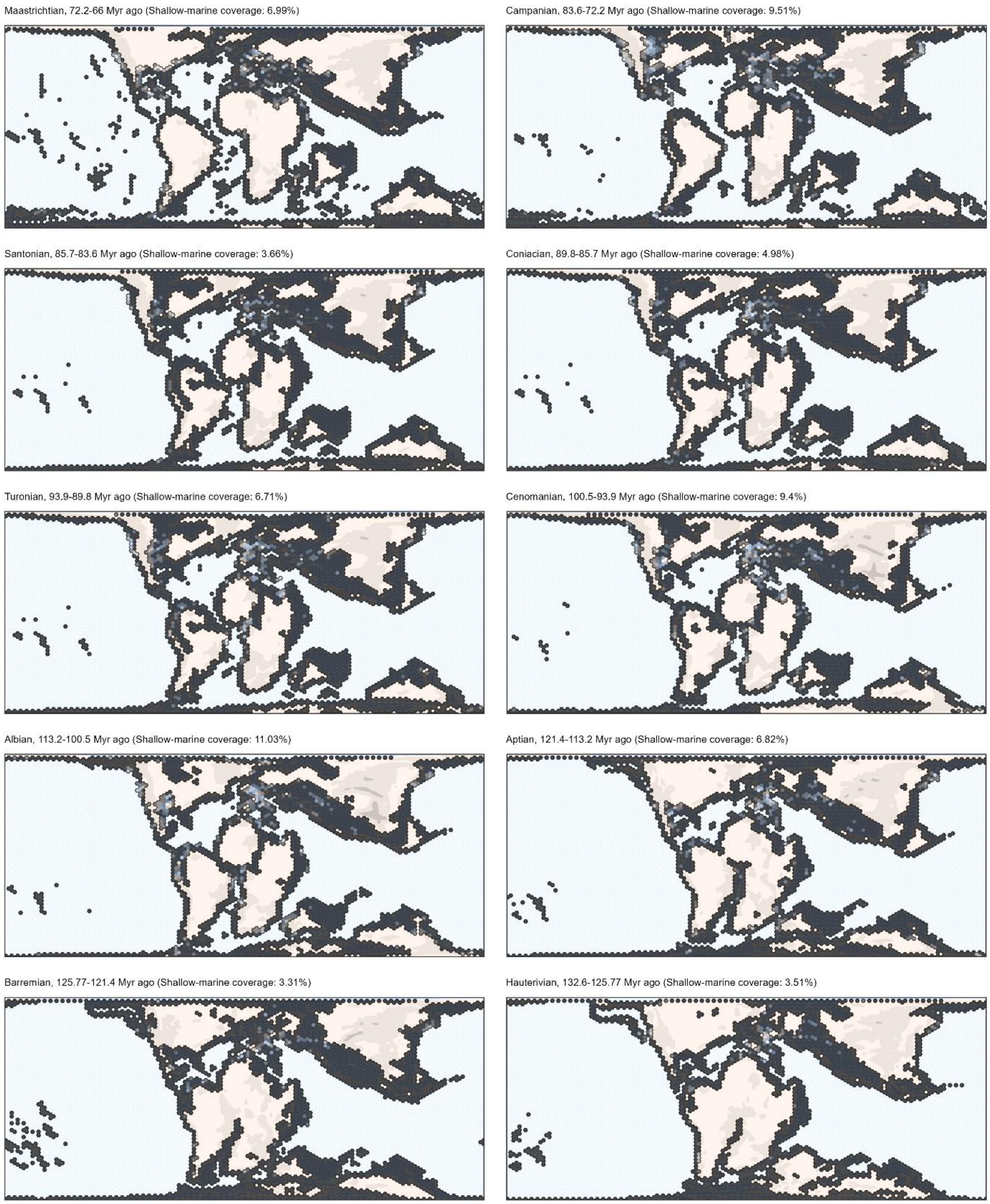

Fig. S2. Continued.

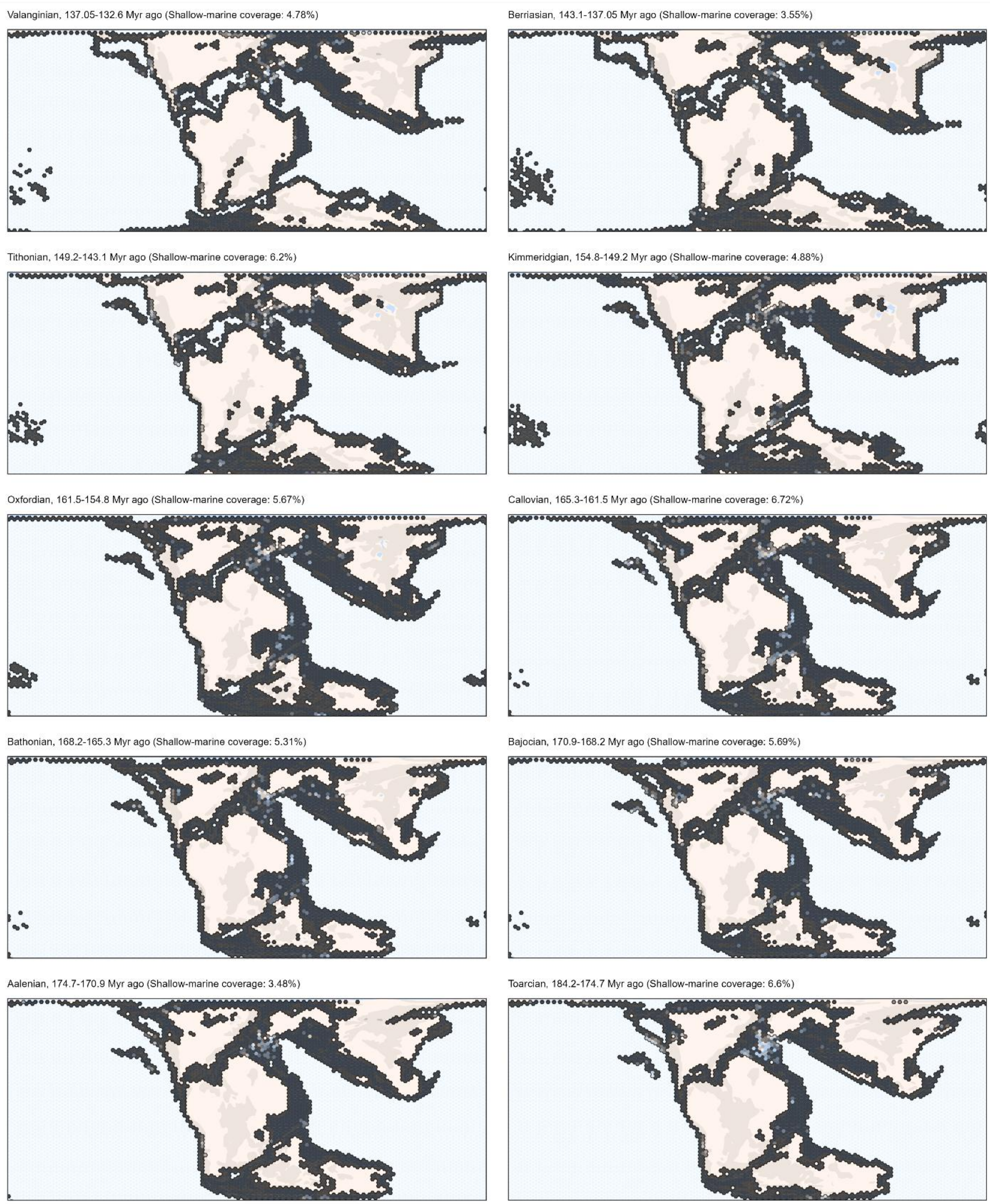

Fig. S2. Continued.

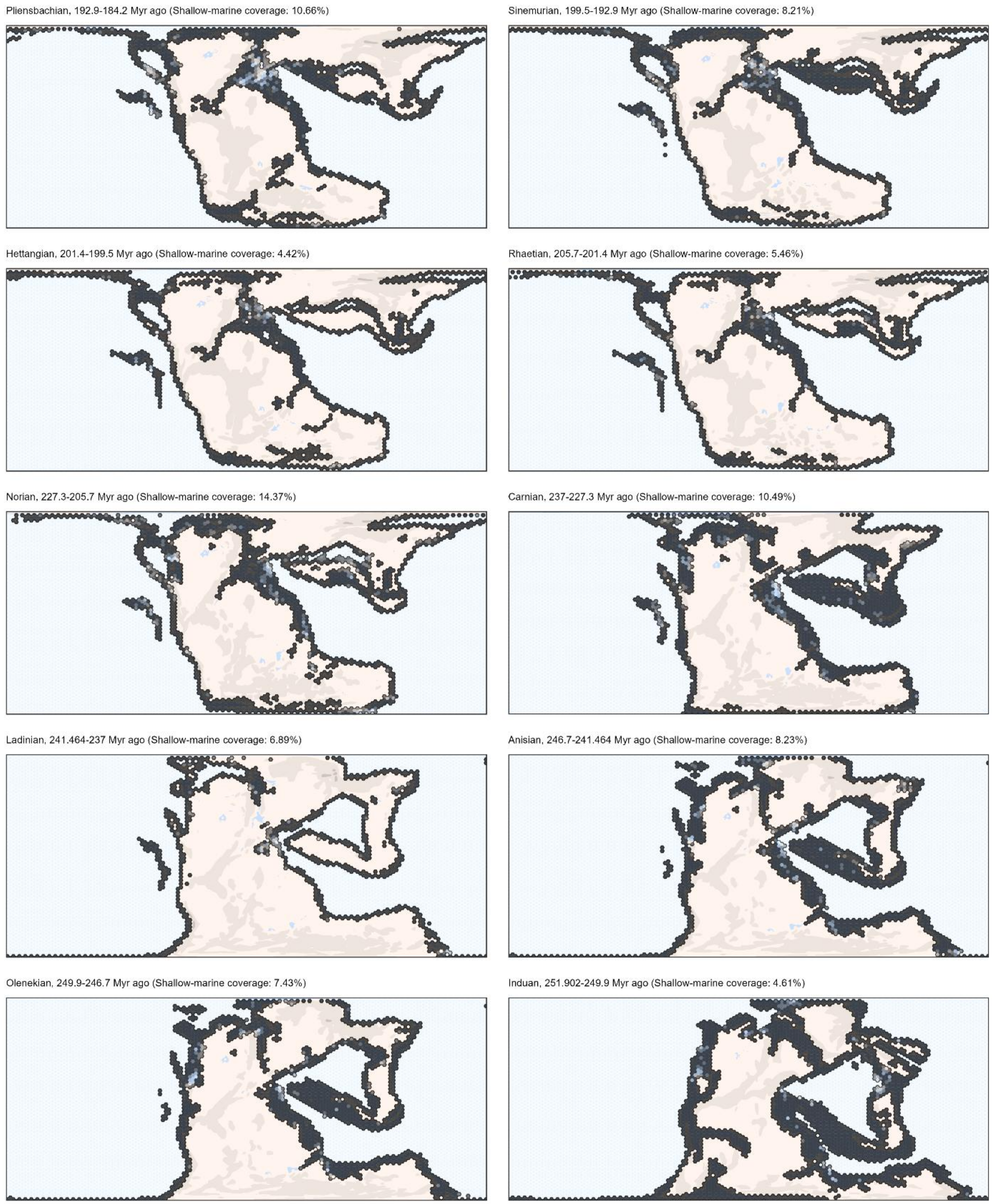

Fig. S2. Continued.

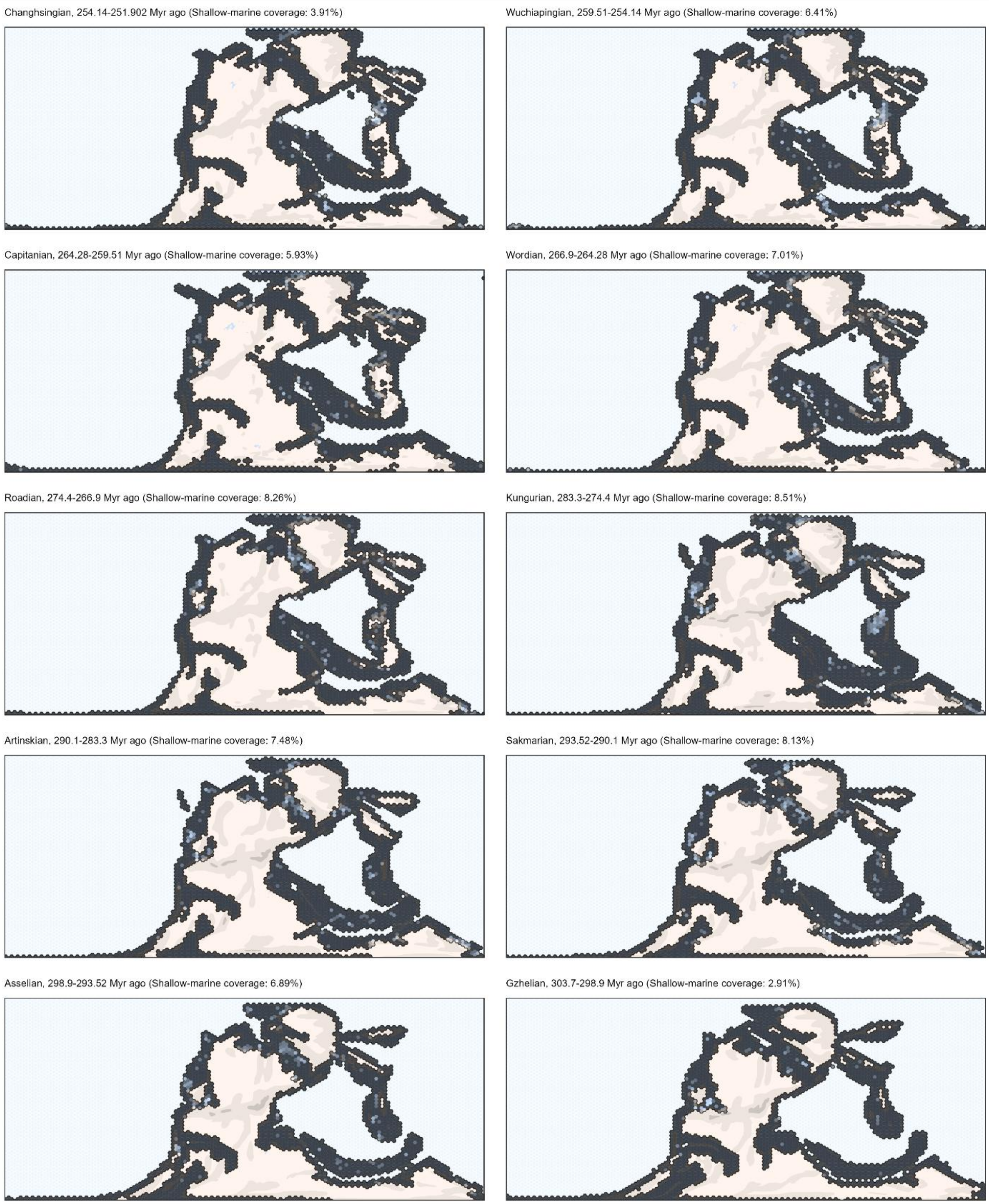

Fig. S2. Continued.

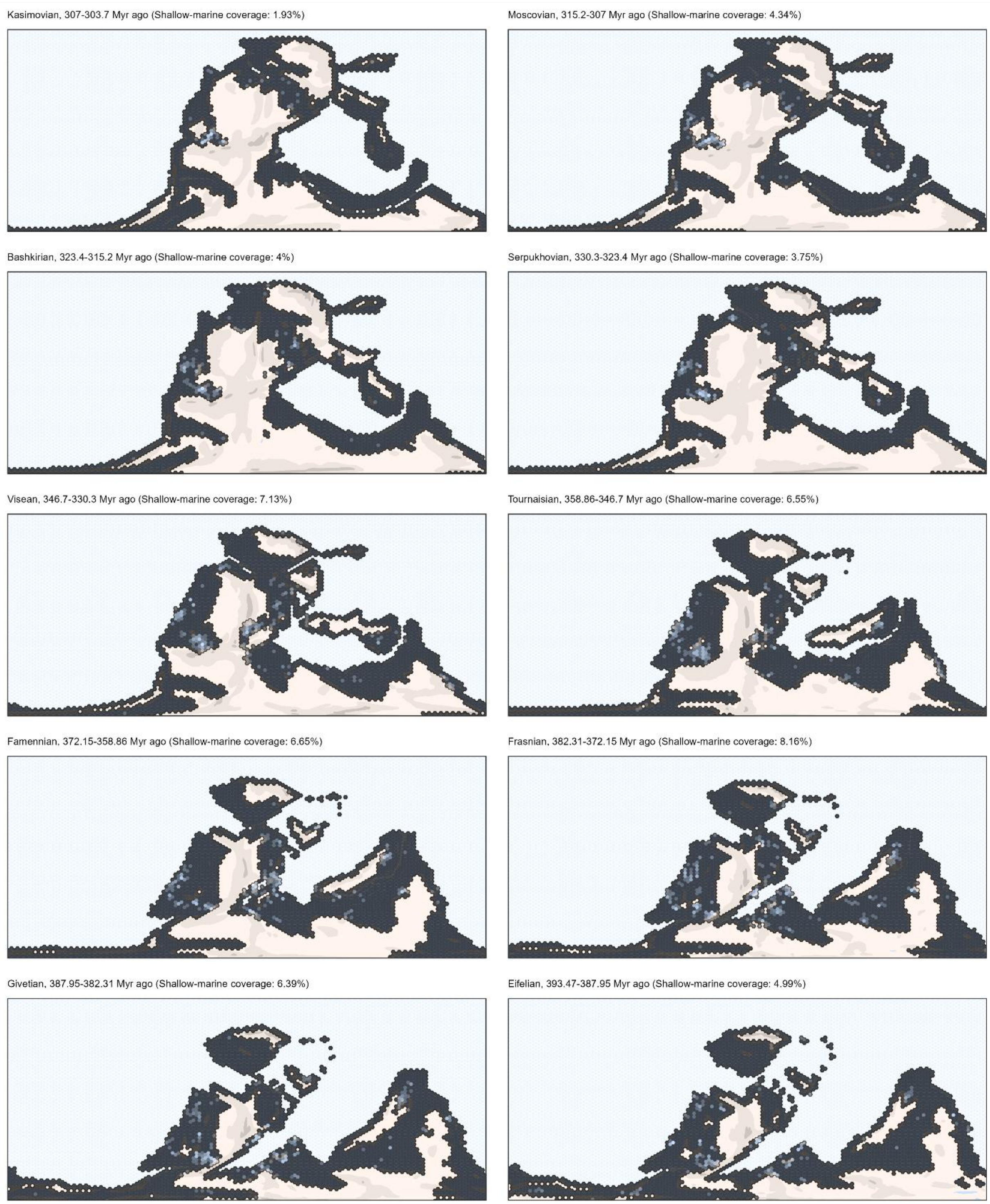

Fig. S2. Continued.

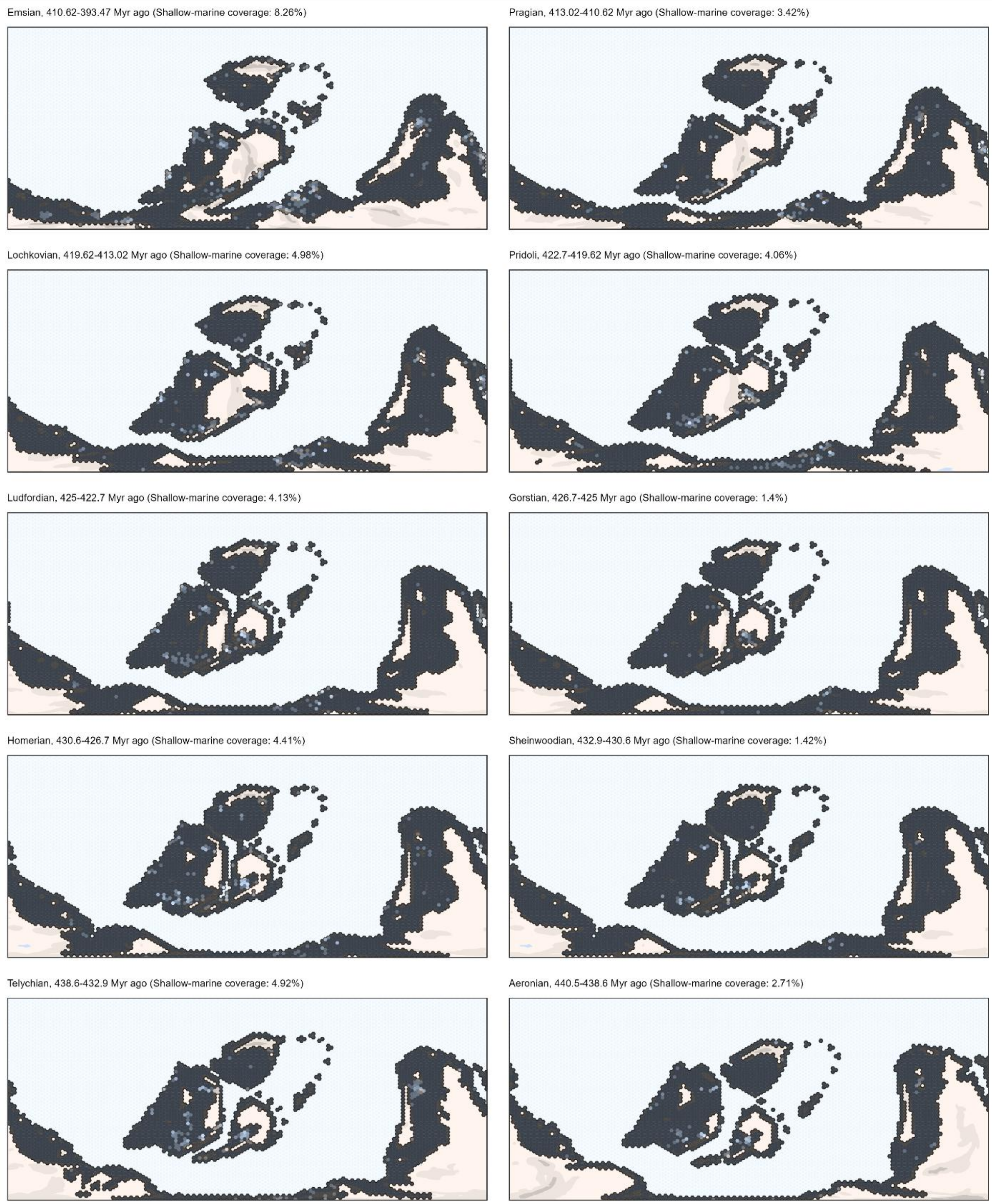

Fig. S2. Continued.

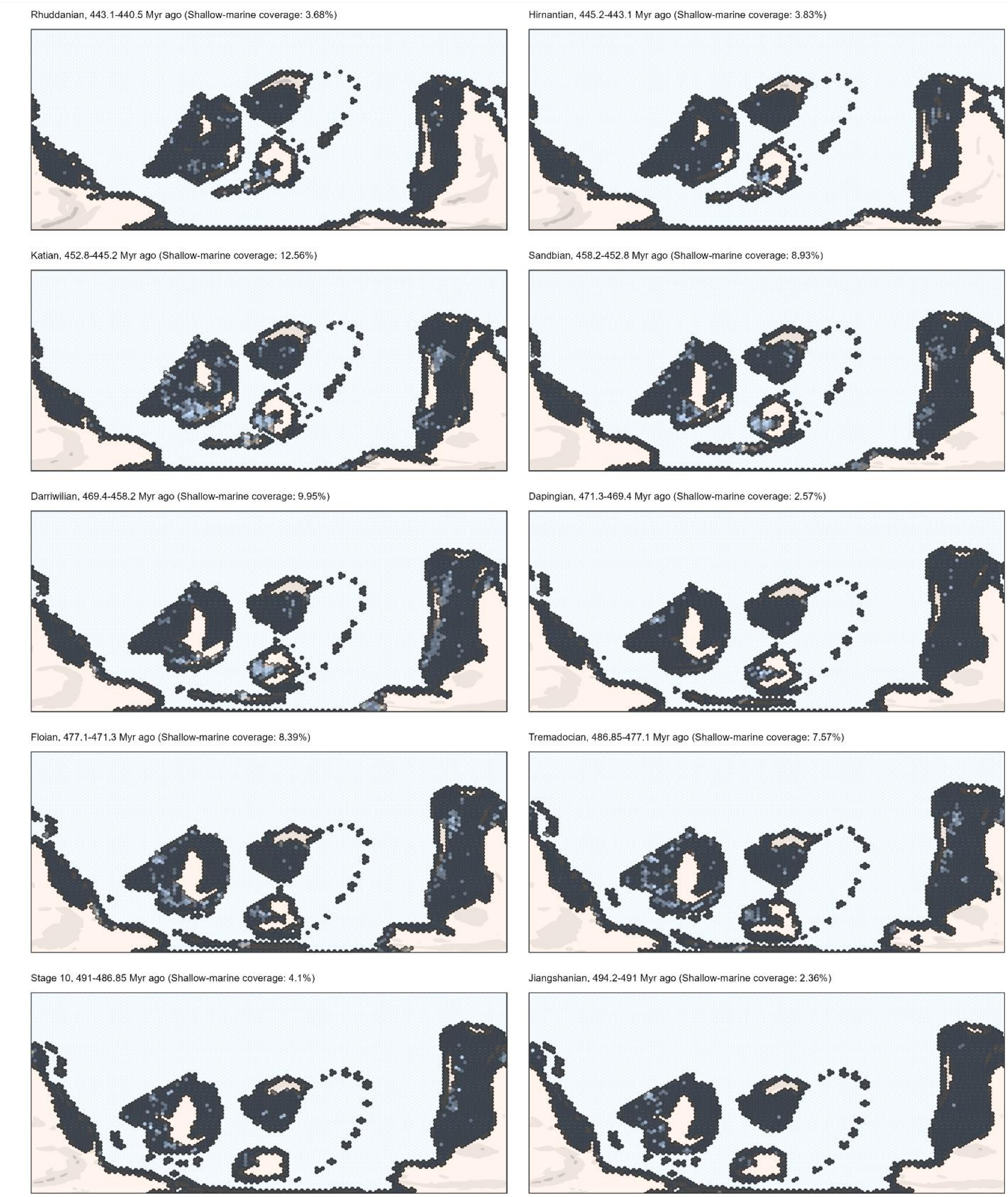

Fig. S2. Continued.

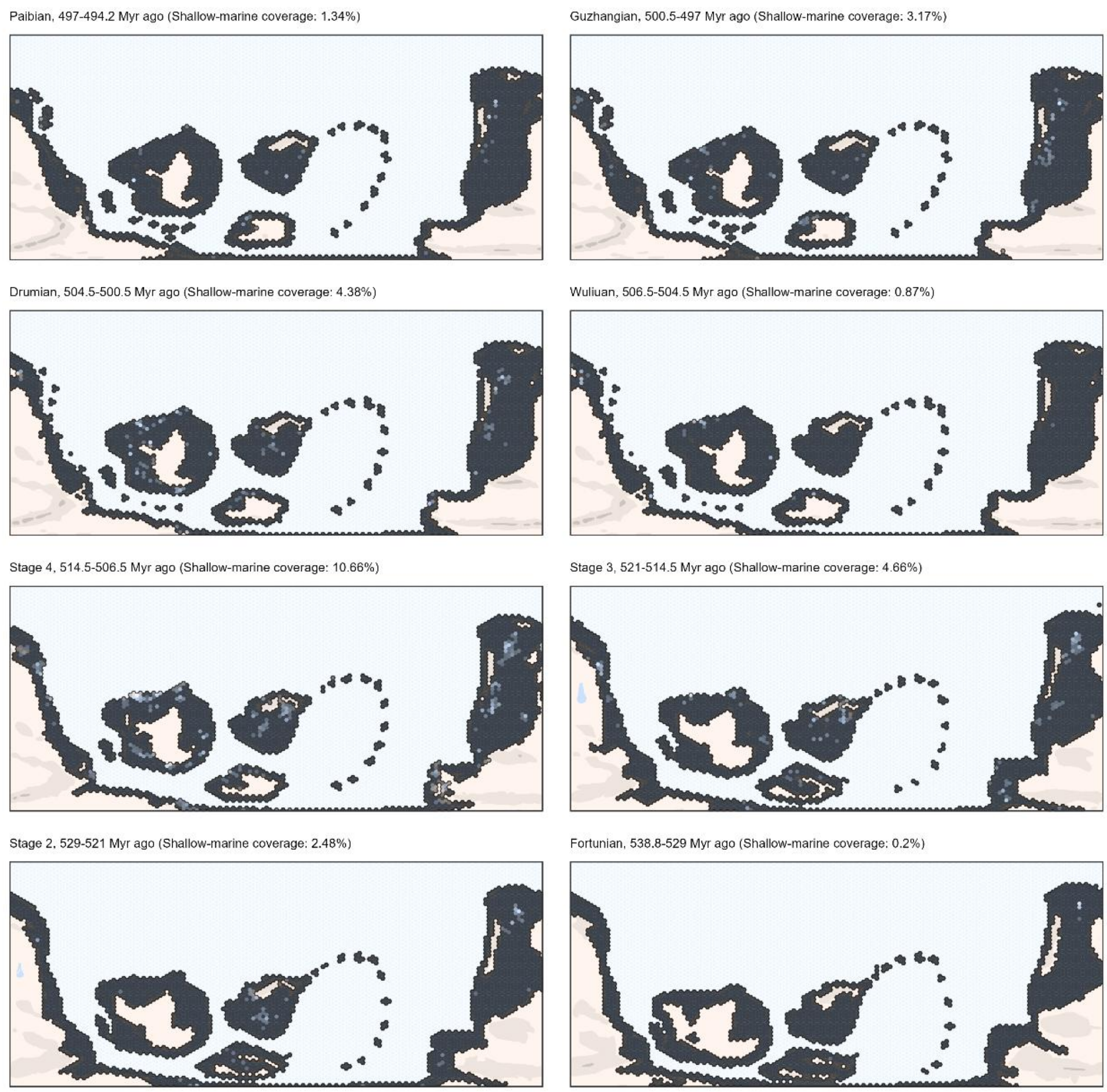

Fig. S2. Continued.

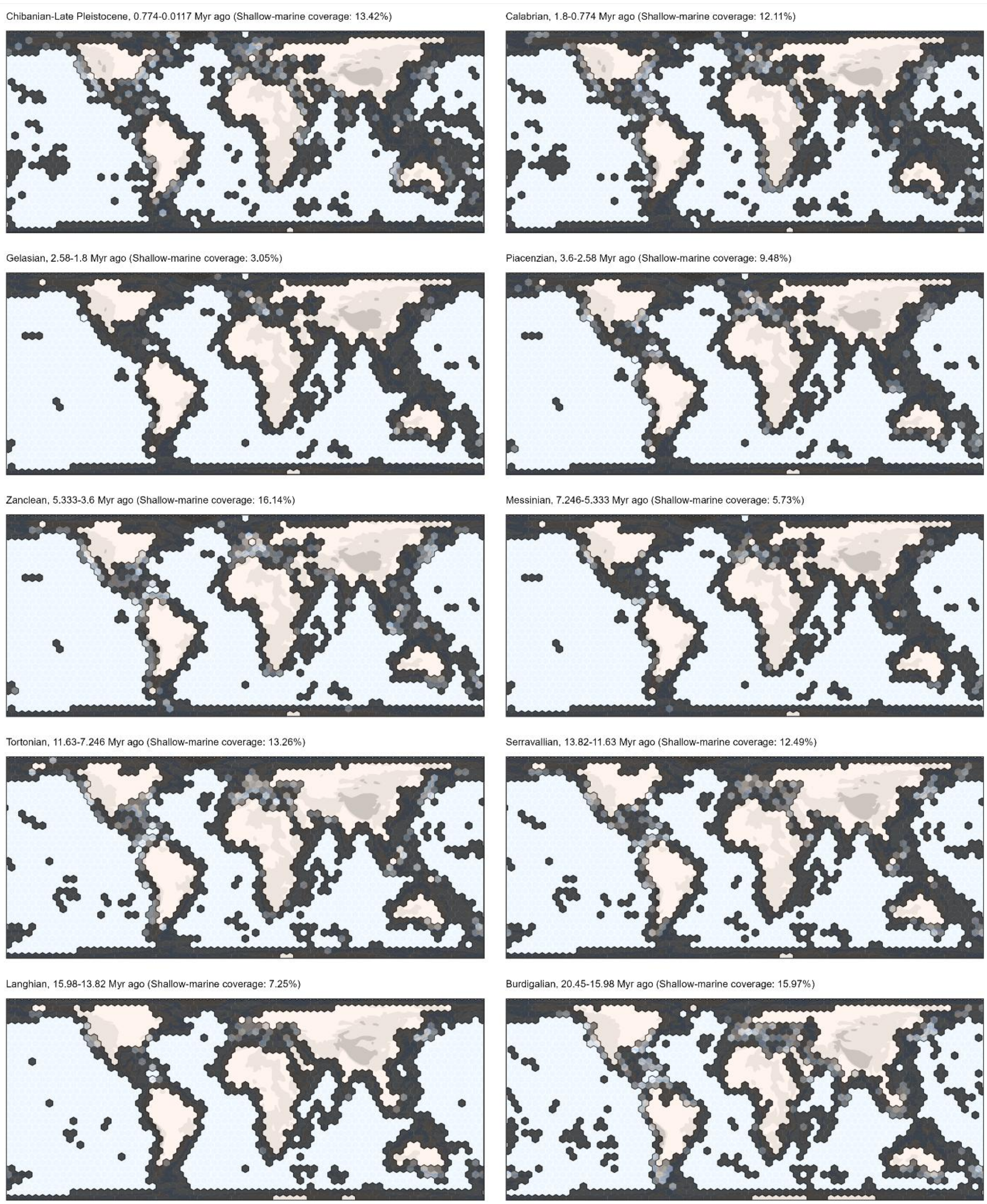

Fig. S3.

Paleogeographic maps showing information density on shallow-marine metazoans as light hexagons for the 500 km pointy-topped grid. The number of fossil collections per grid cell is displayed as relative transparency (see Figure 1 in main text for legend). Corresponding data on coverage is summarized in table S4.

Aquitanian, 23.04-20.45 Myr ago (Shallow-marine coverage: 4.62%)

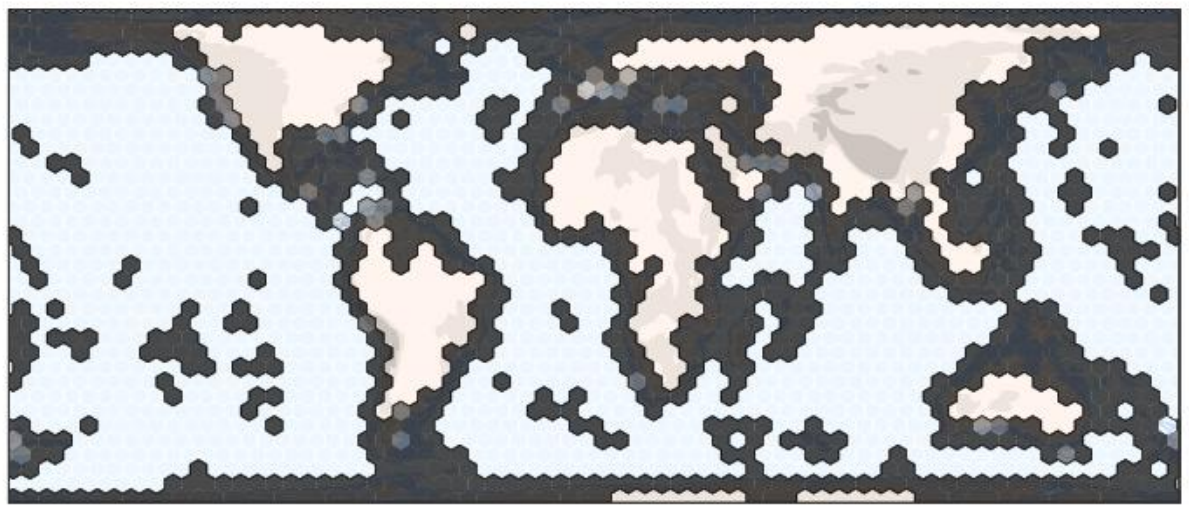

Chattian, 27.3-23.04 Myr ago (Shallow-marine coverage: 11.59%)

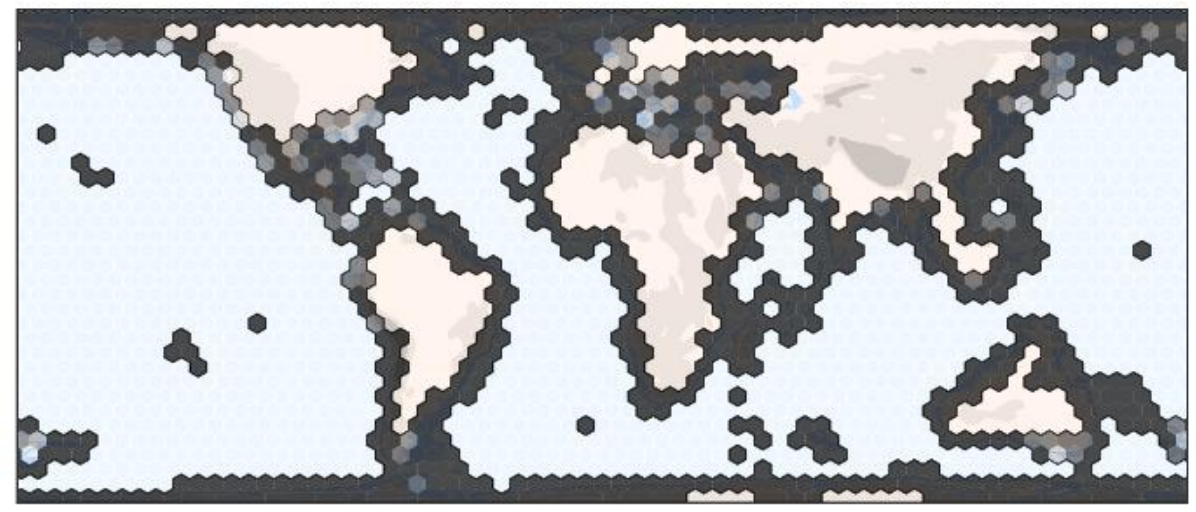

Rupelian, 33.9-27.3 Myr ago (Shallow-marine coverage: 8.65%)

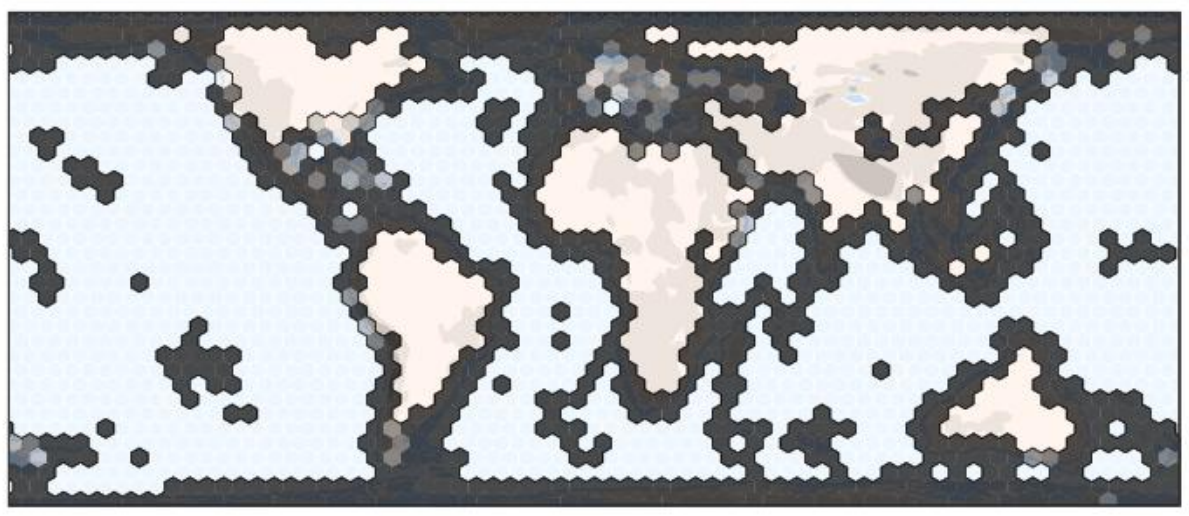

Priabonian, 37.71-33.9 Myr ago (Shallow-marine coverage: 11.79%)

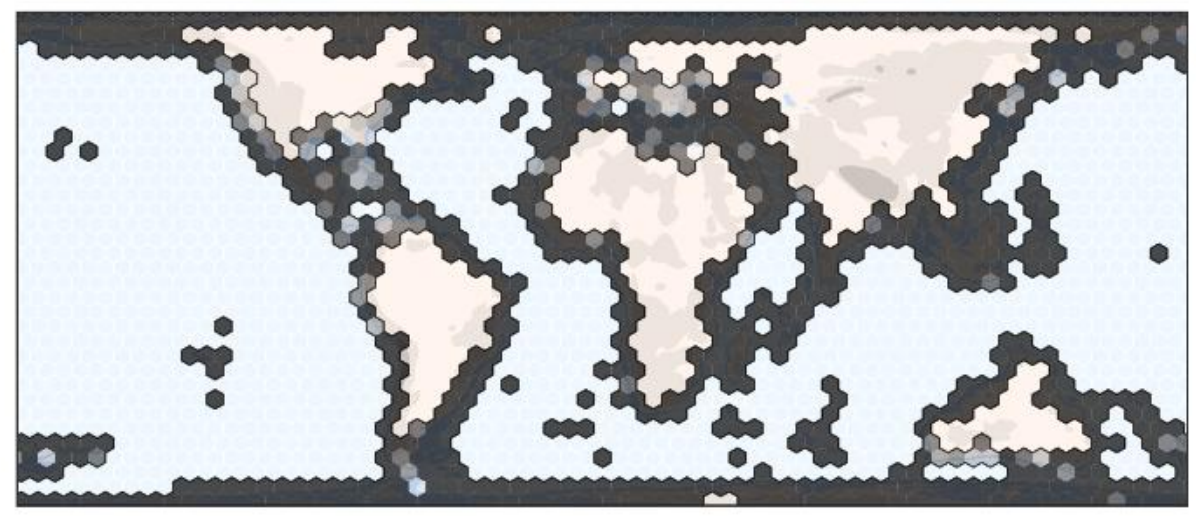

Bartonian, 41.03-37.71 Myr ago (Shallow-marine coverage: 6.84%)

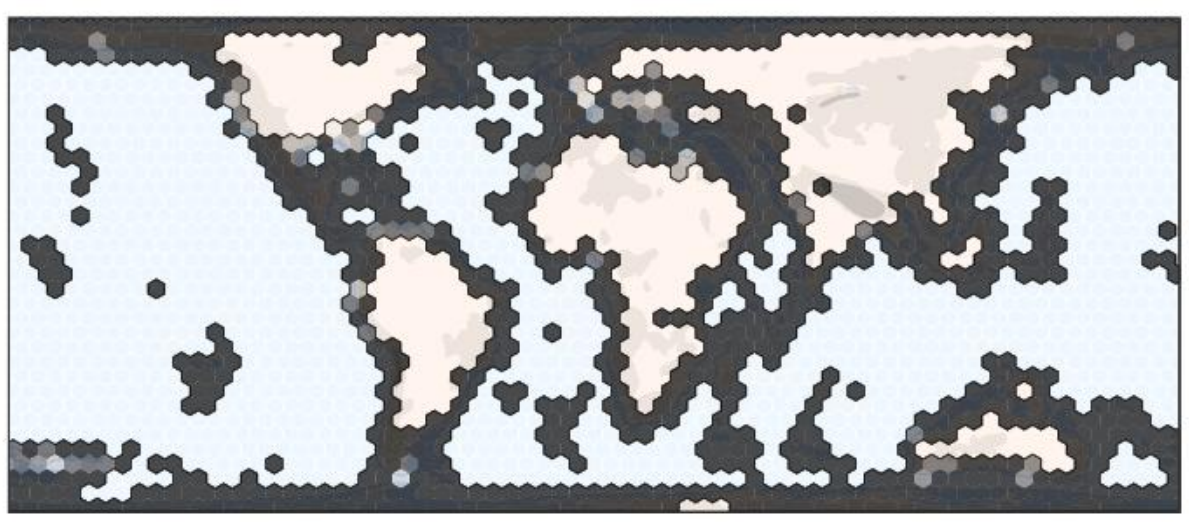

Lutetian, 48.07-41.03 Myr ago (Shallow-marine coverage: 7.45%)

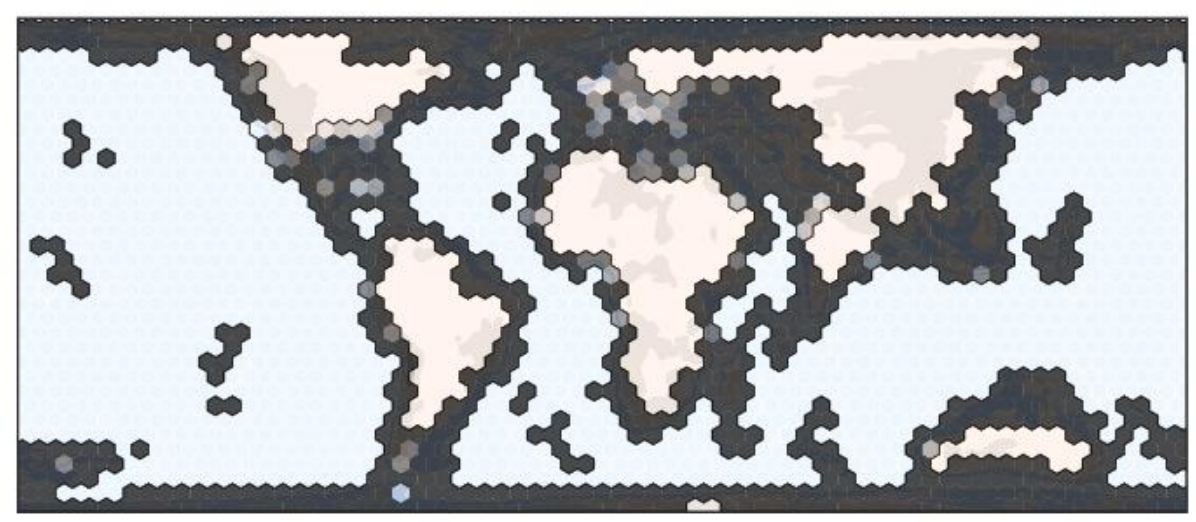

Ypresian, 56-48.07 Myr ago (Shallow-marine coverage: 7.29%)

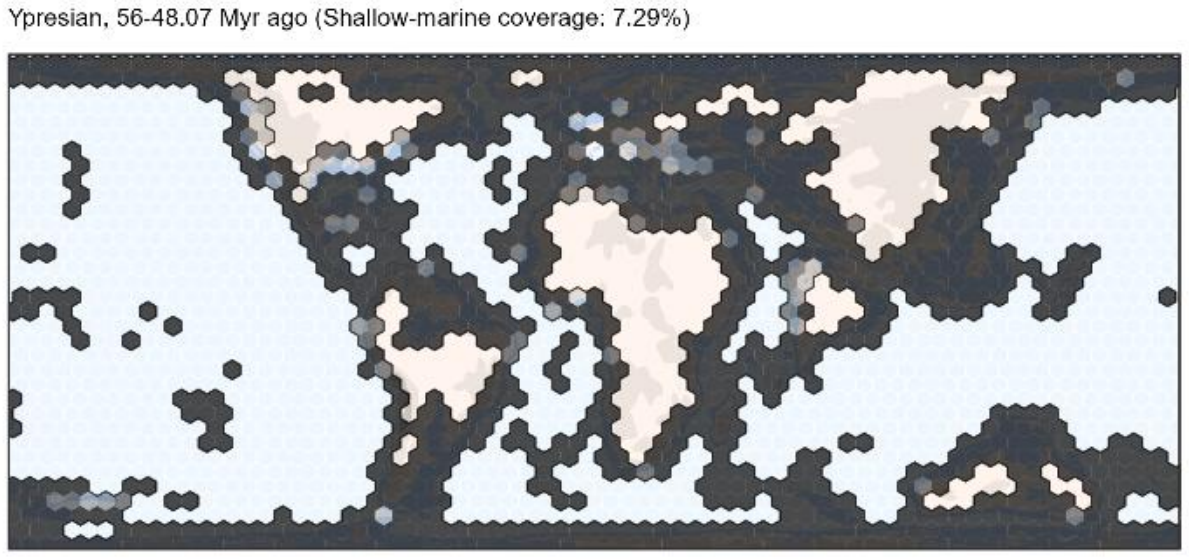

Thanetian, 59.24-56 Myr ago (Shallow-marine coverage: 5.69%)

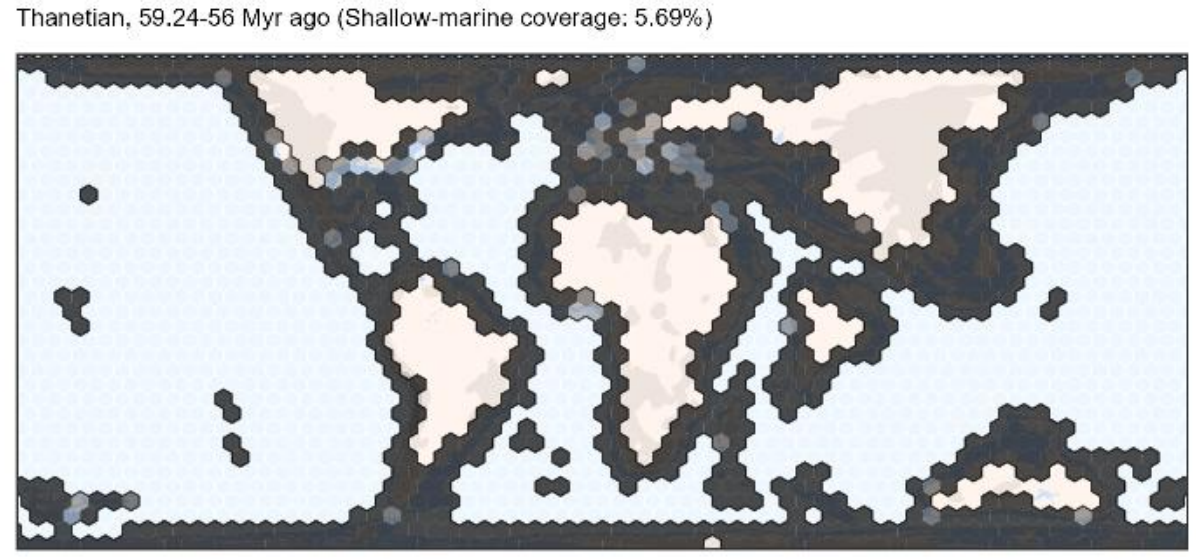

Selandian, 61.66-59.24 Myr ago (Shallow-marine coverage: 2.57%)

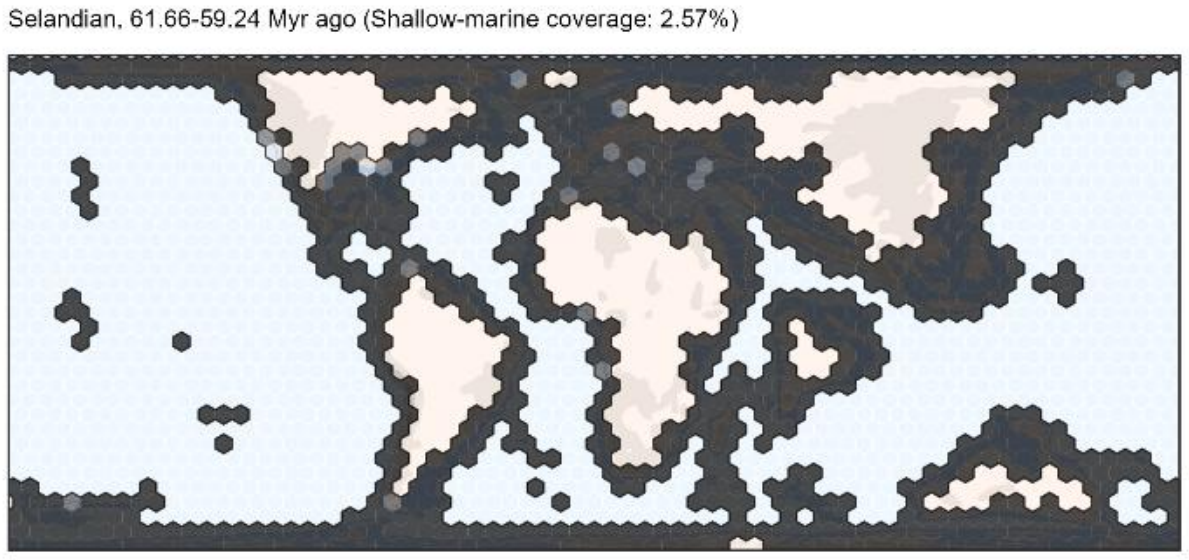

Danian, 66-61.66 Myr ago (Shallow-marine coverage: 9.74%)

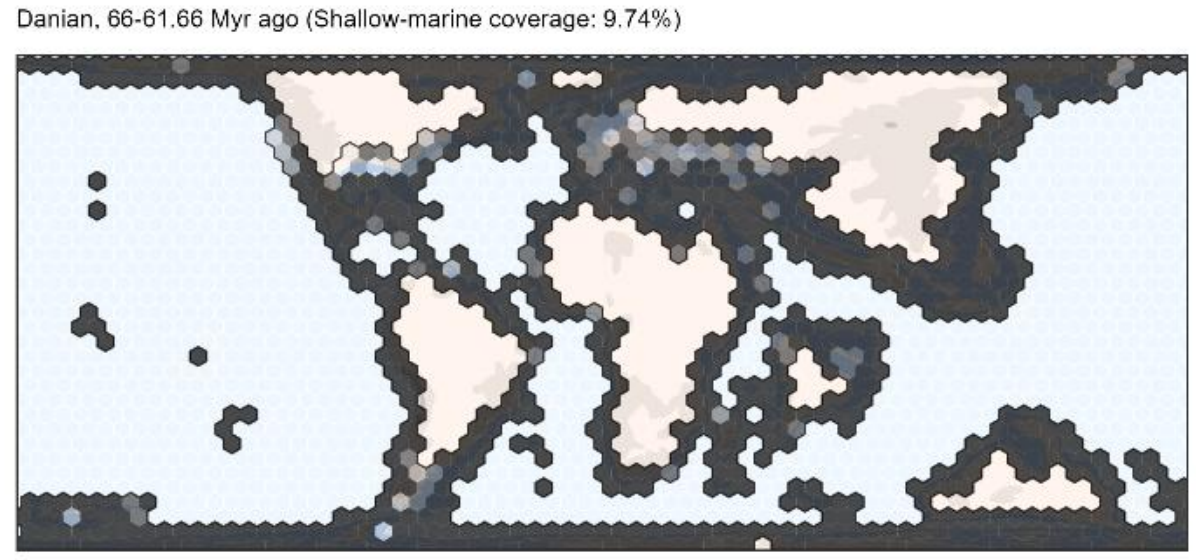

Fig. S3. Continued.

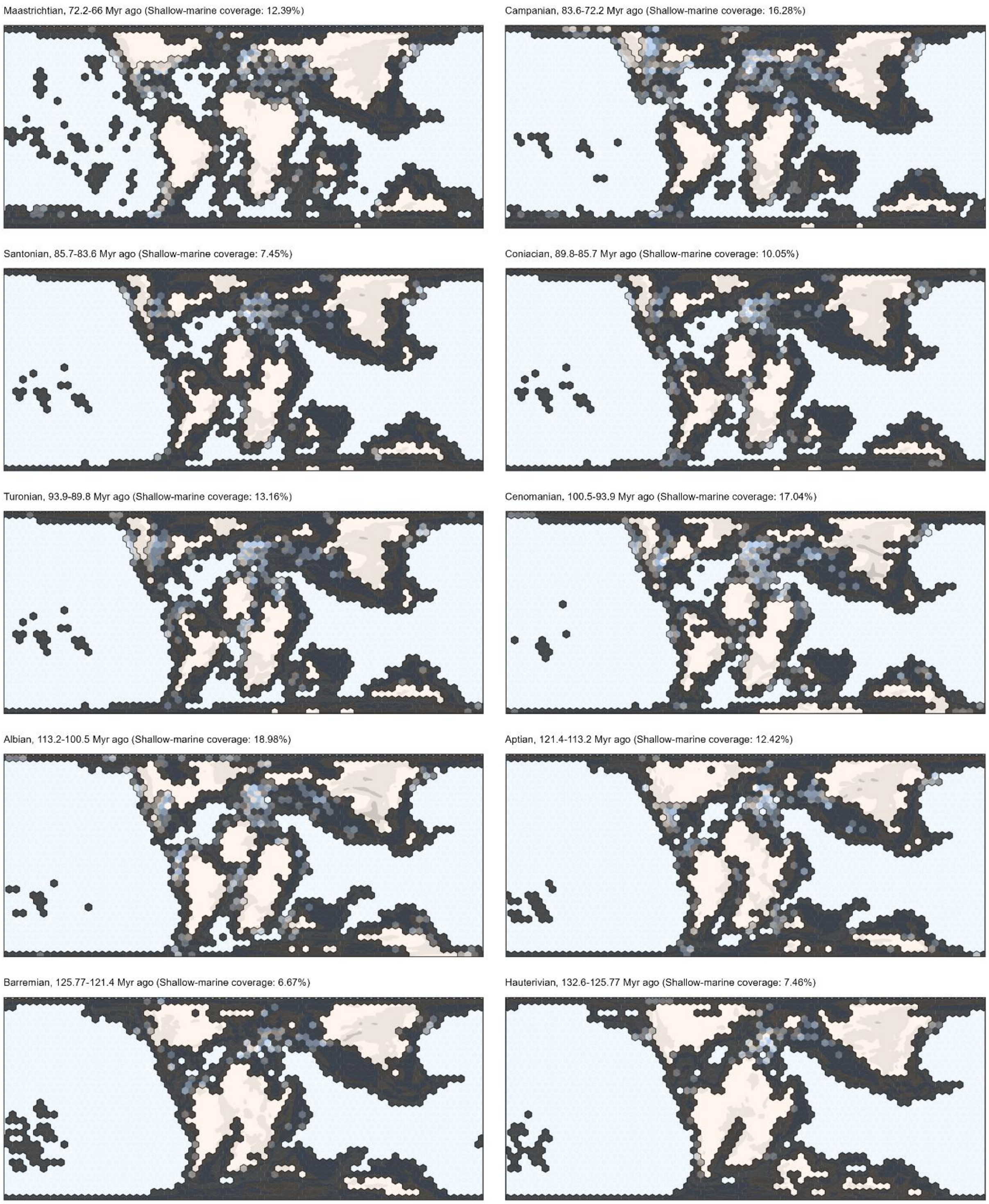

Fig. S3. Continued.

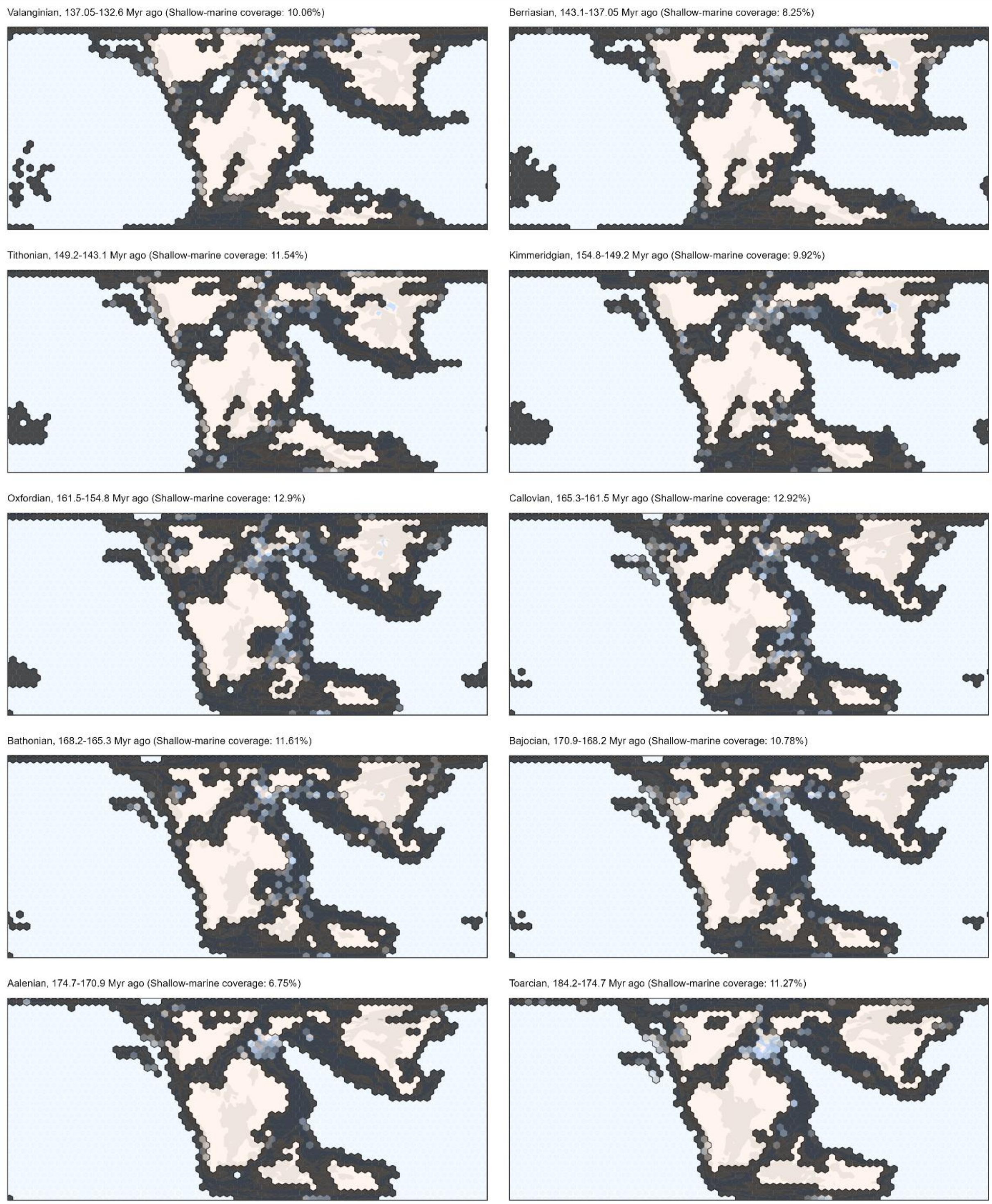

Fig. S3. Continued.

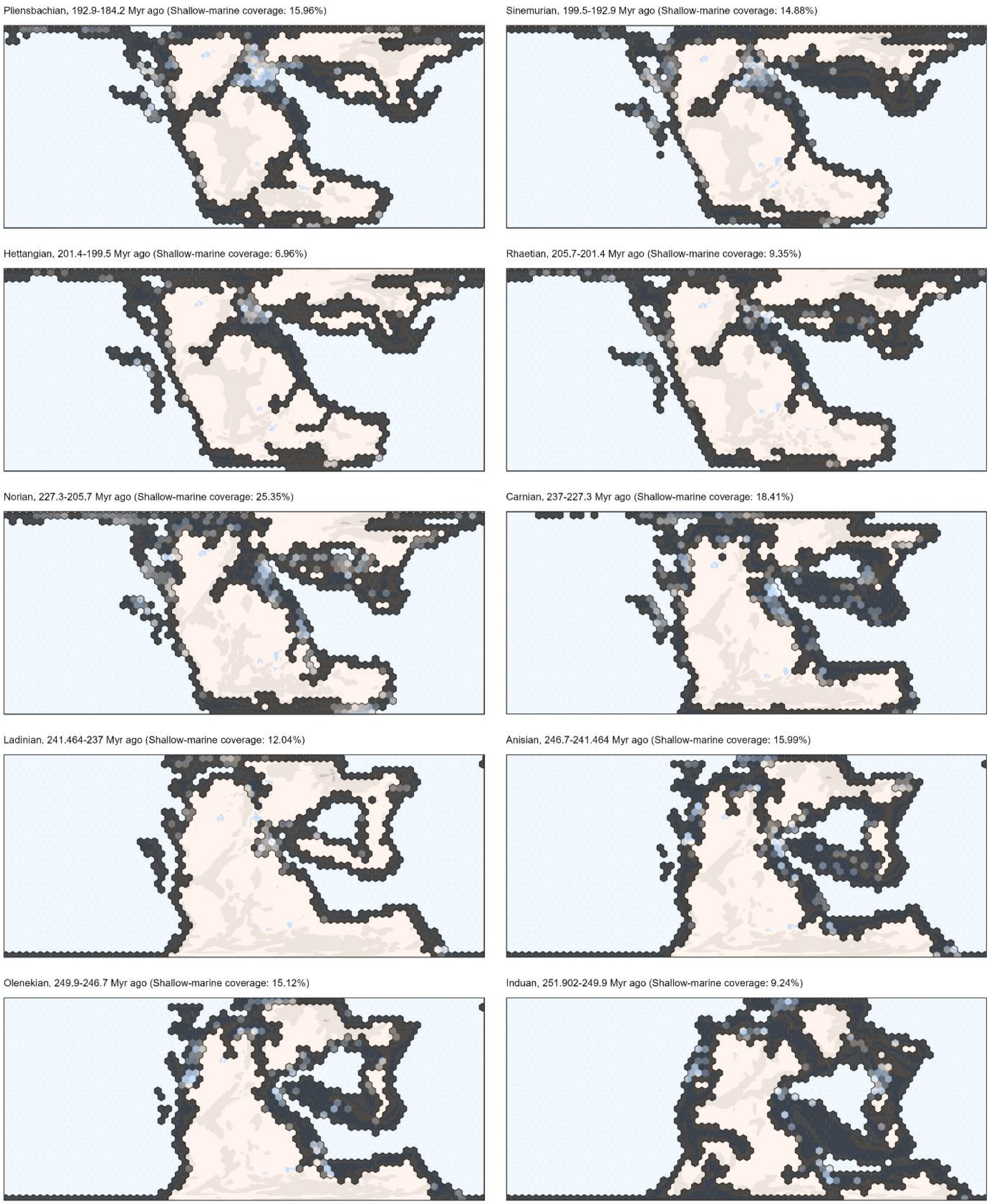

Fig. S3. Continued.

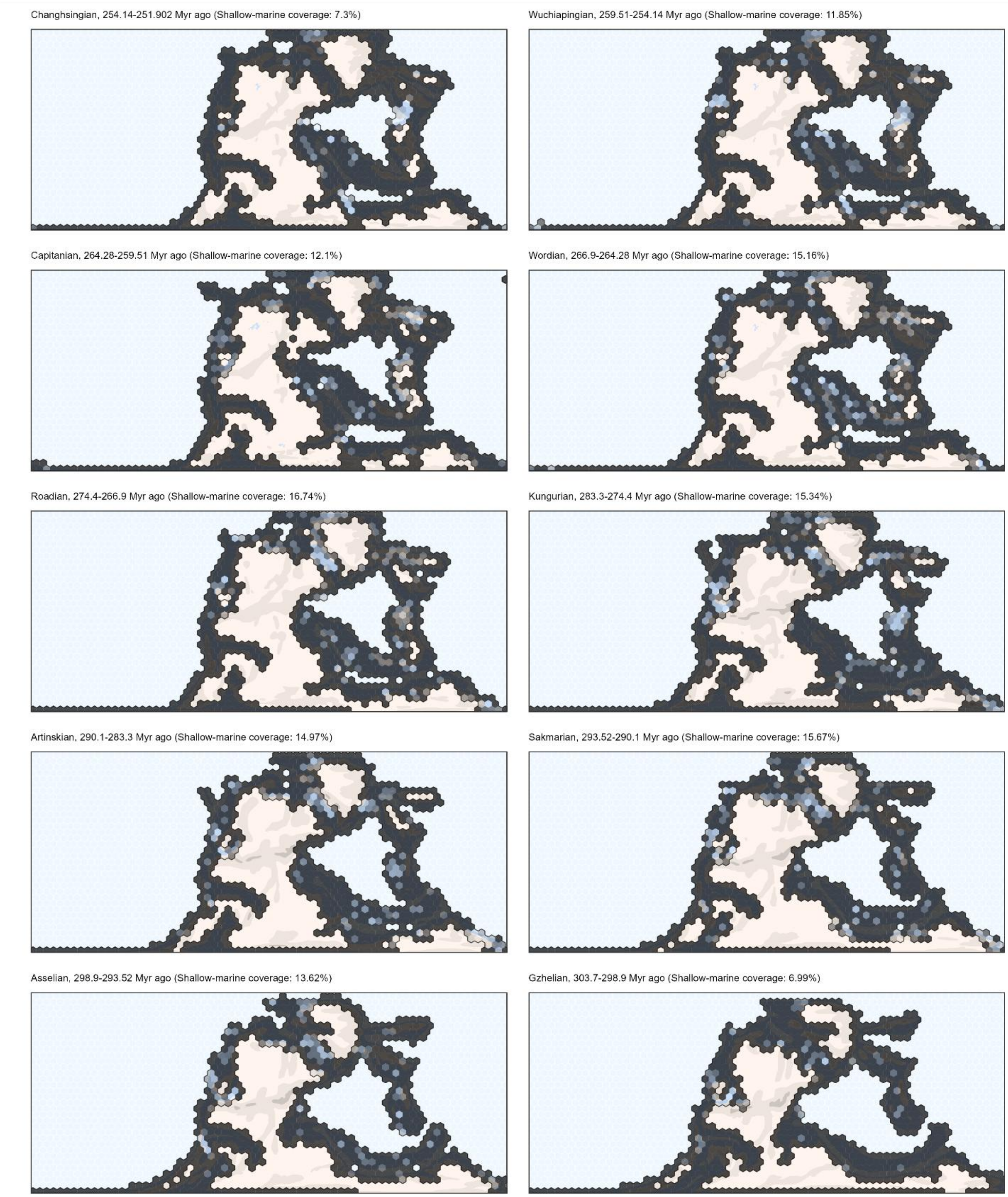

Fig. S3. Continued.

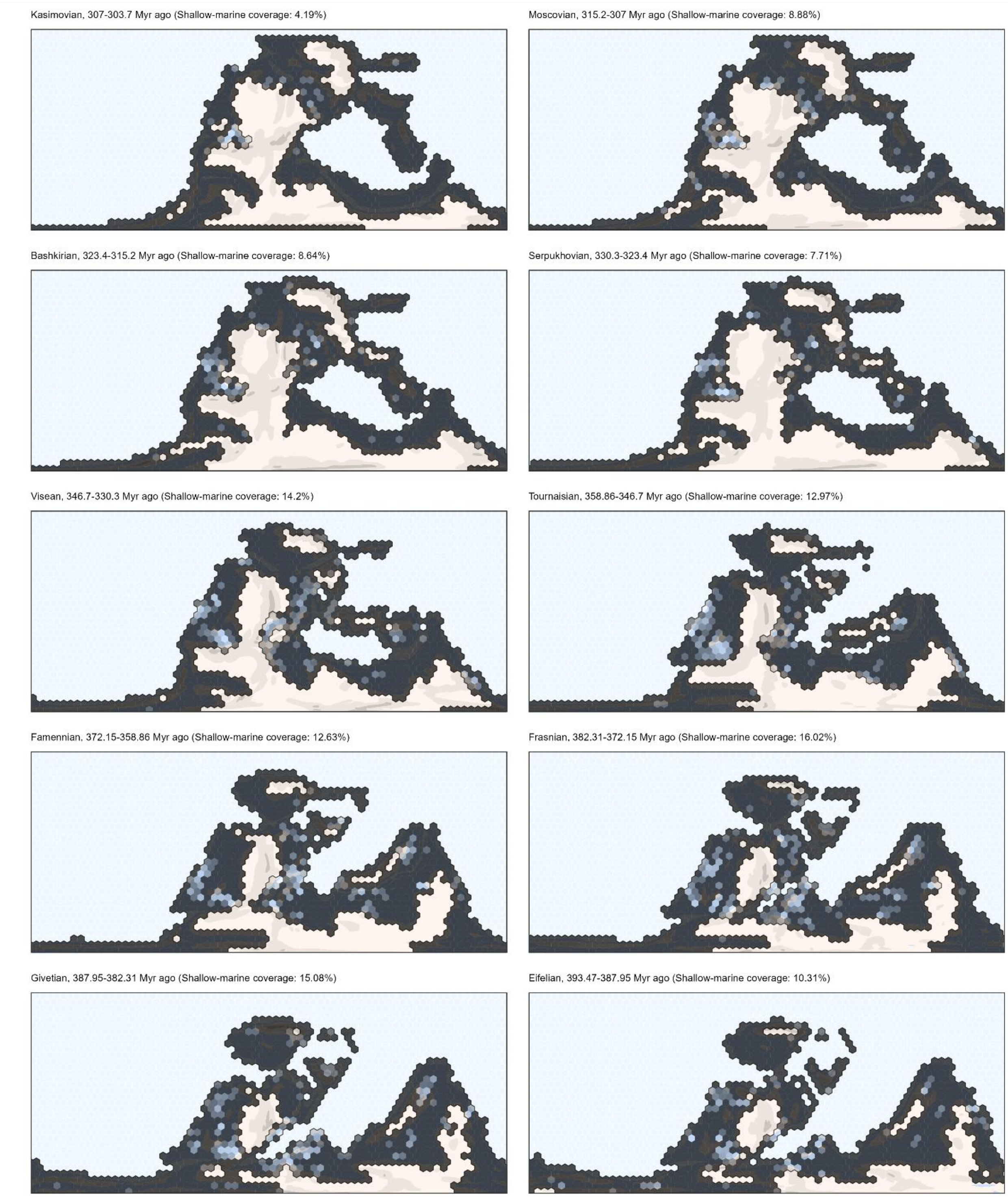

Fig. S3. Continued.

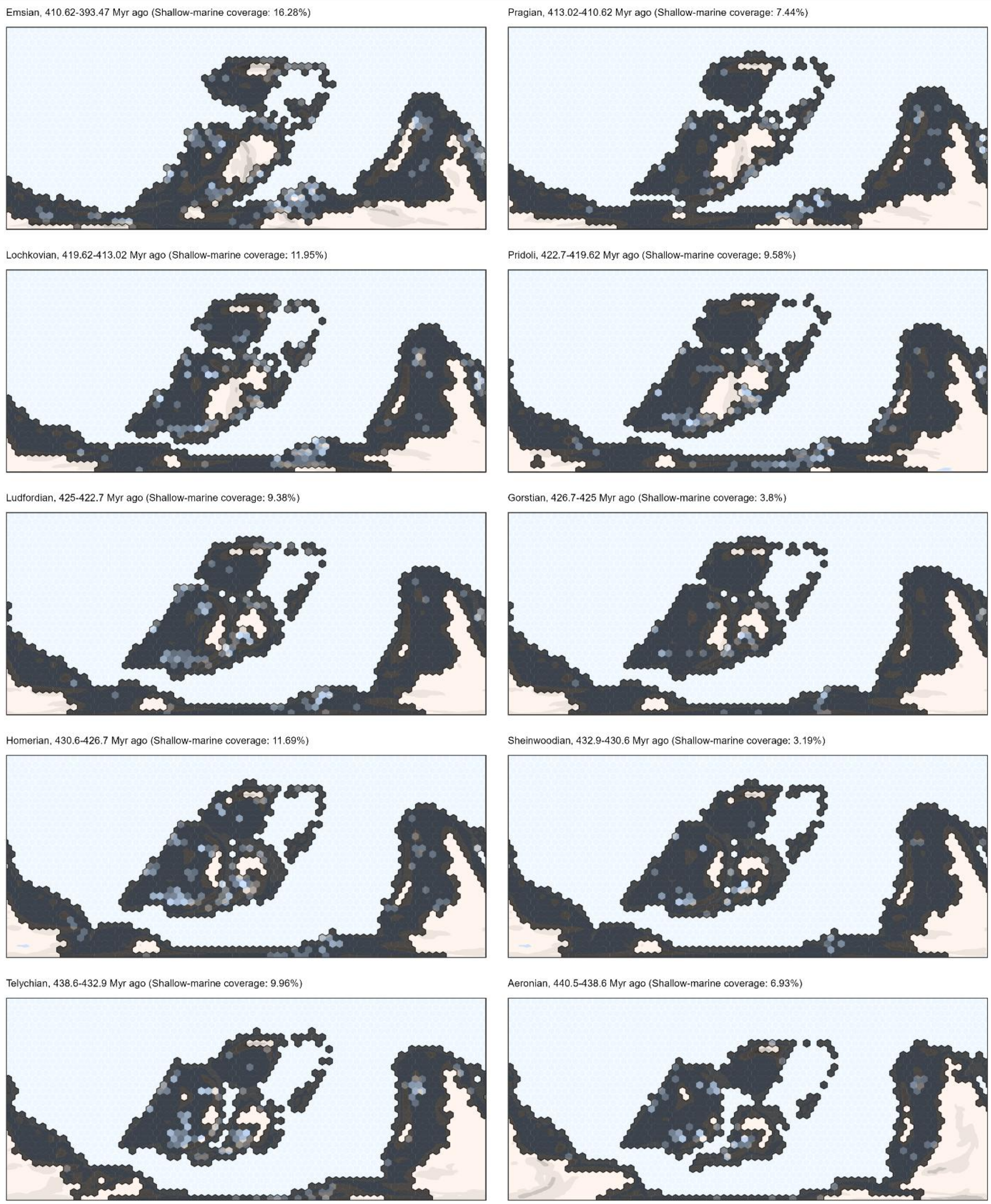

Fig. S3. Continued.

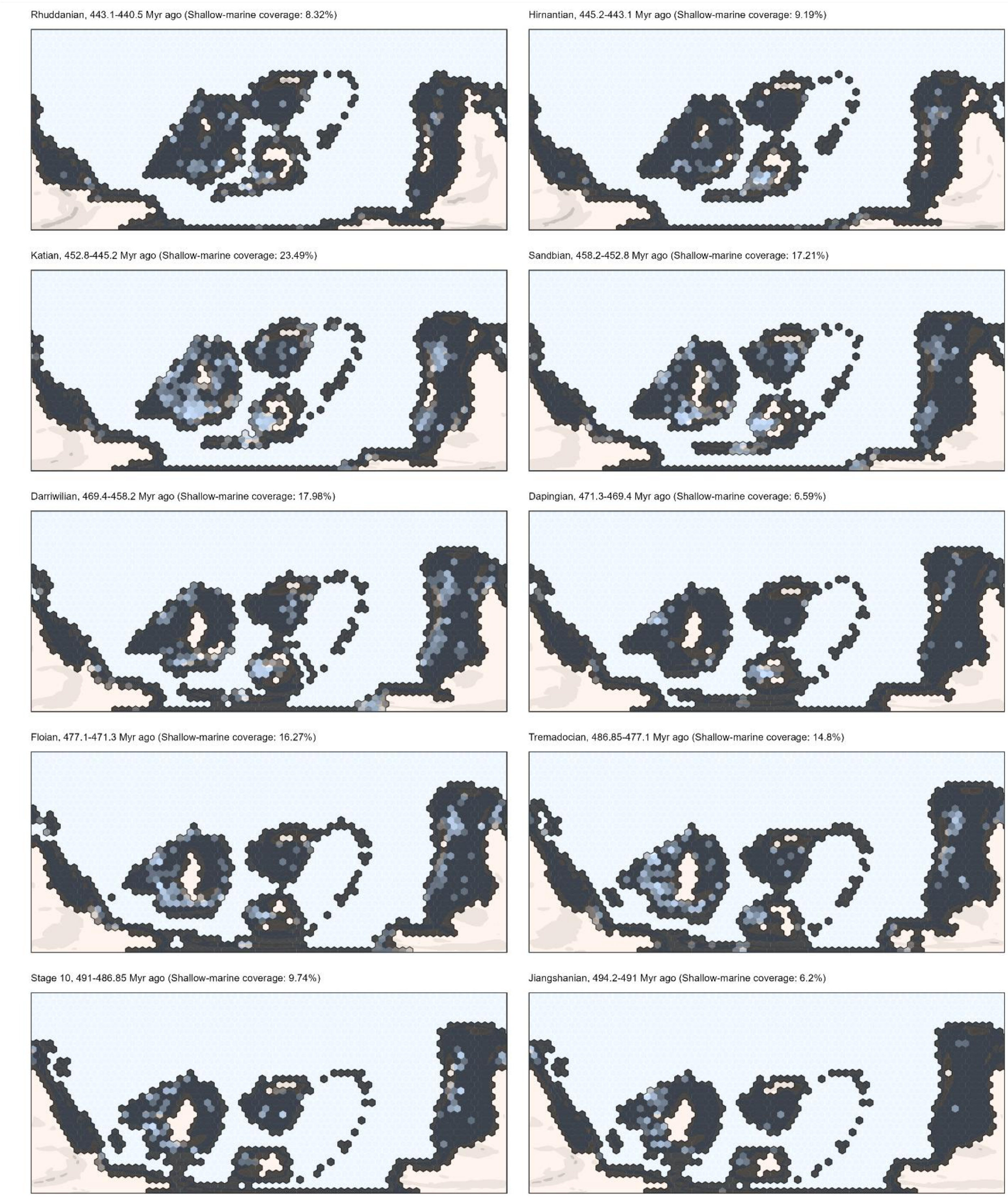

Fig. S3. Continued.

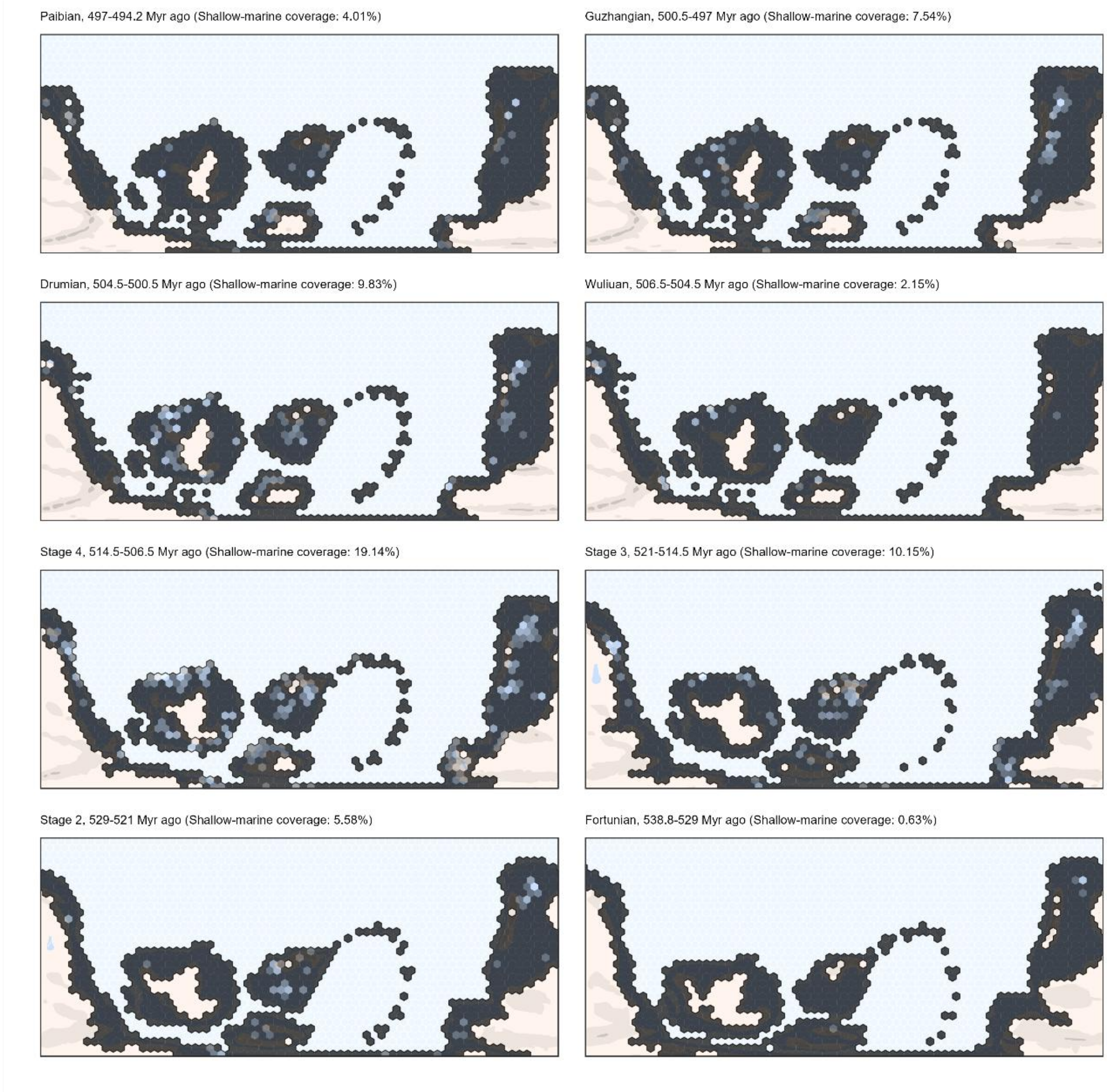

Fig. S3. Continued.

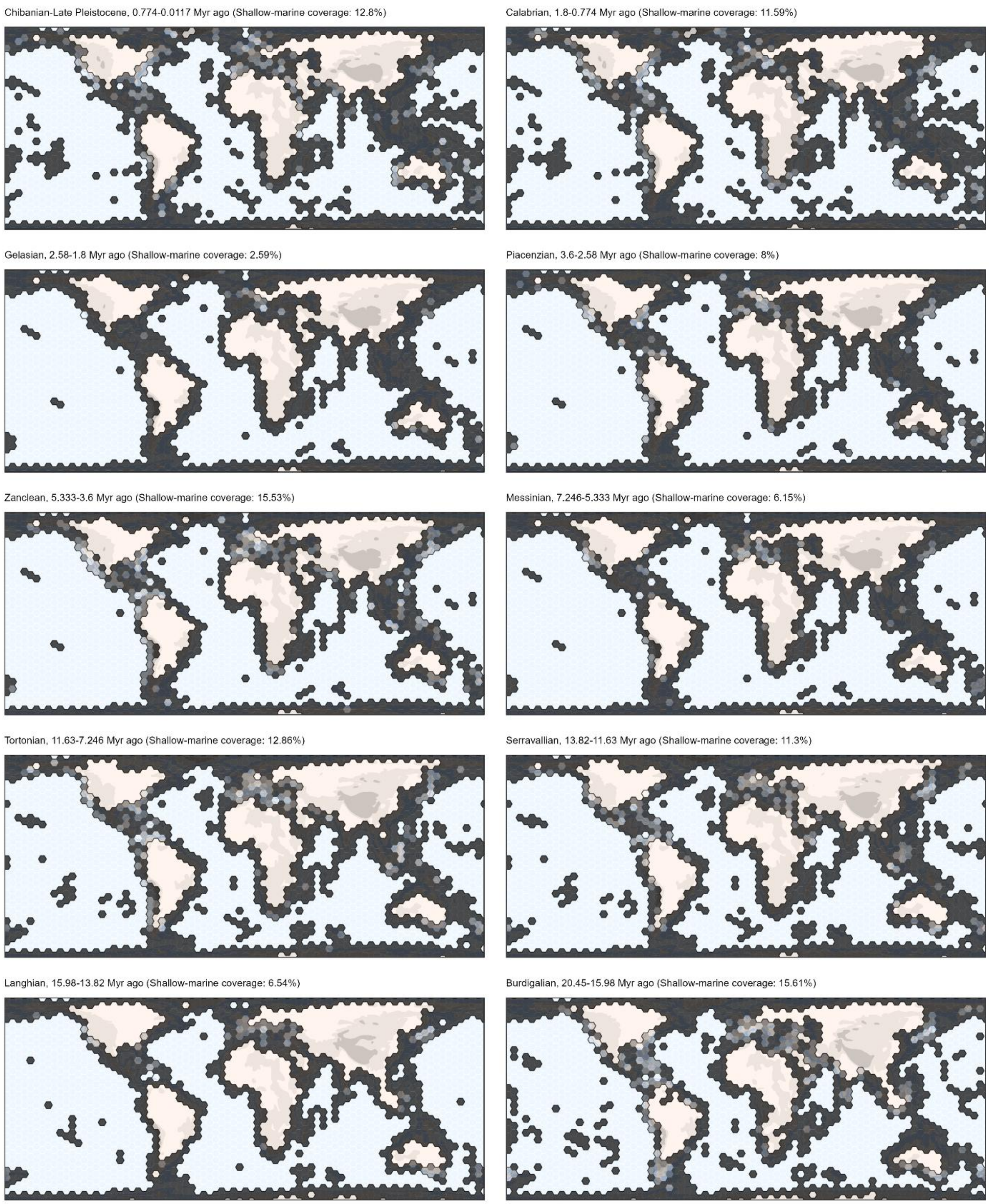

Fig. S4.

Paleogeographic maps showing information density on shallow-marine metazoans as light hexagons for the 500 km flat-topped grid. The number of fossil collections per grid cell is displayed as relative transparency (see Figure 1 in main text for legend). Corresponding data on coverage is summarized in table S4.

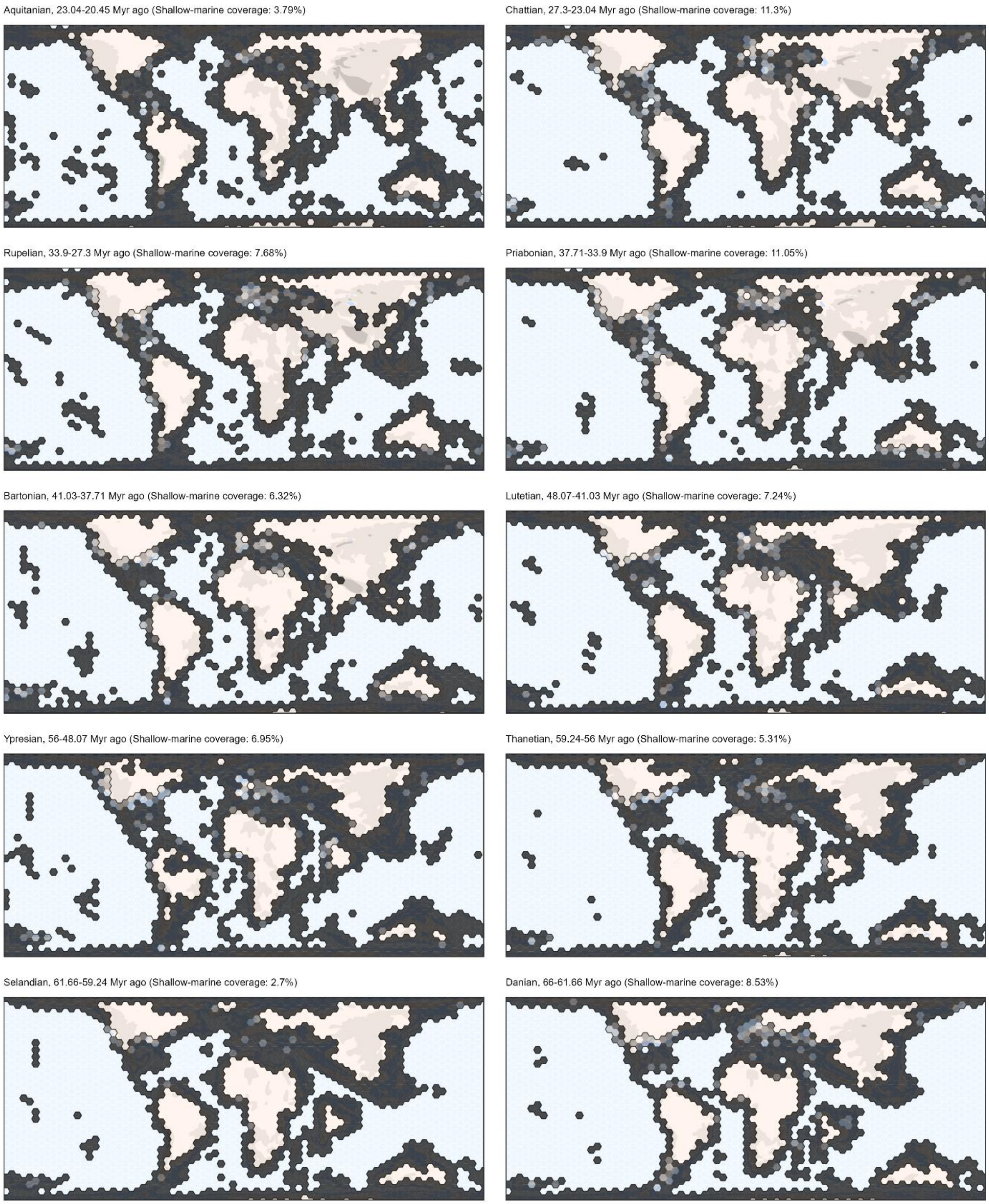

Fig. S4. Continued.

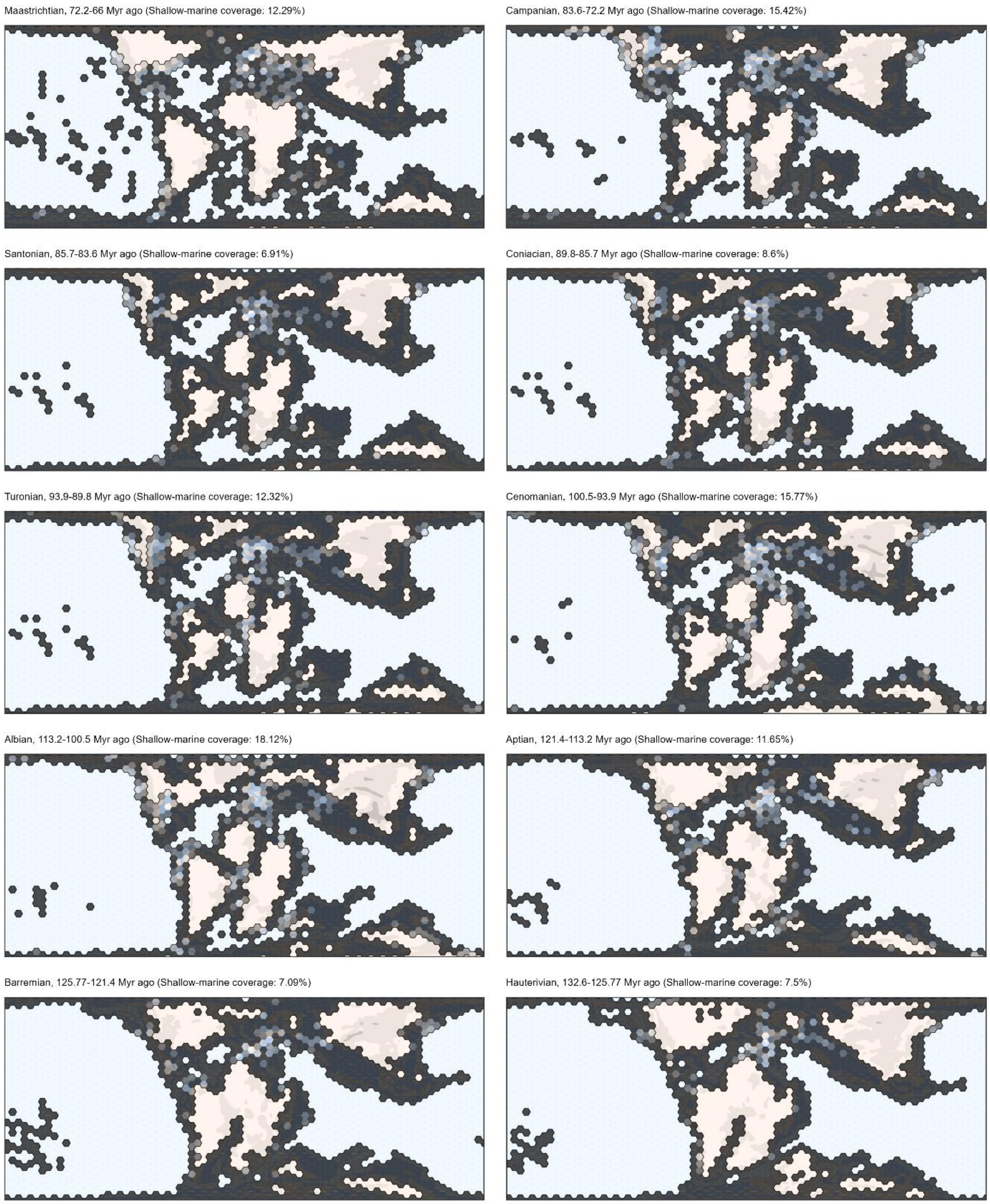

Fig. S4. Continued.

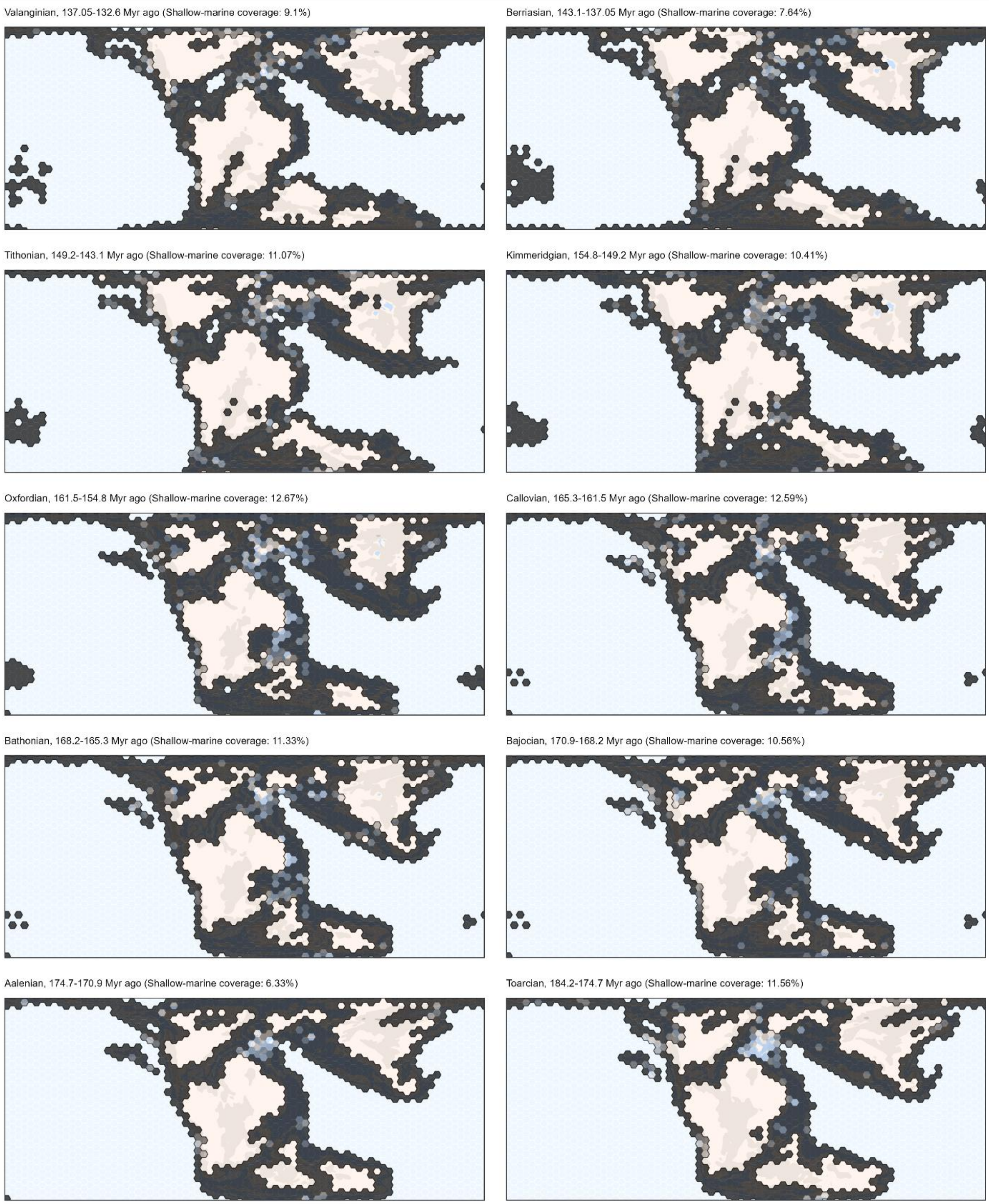

Fig. S4. Continued.

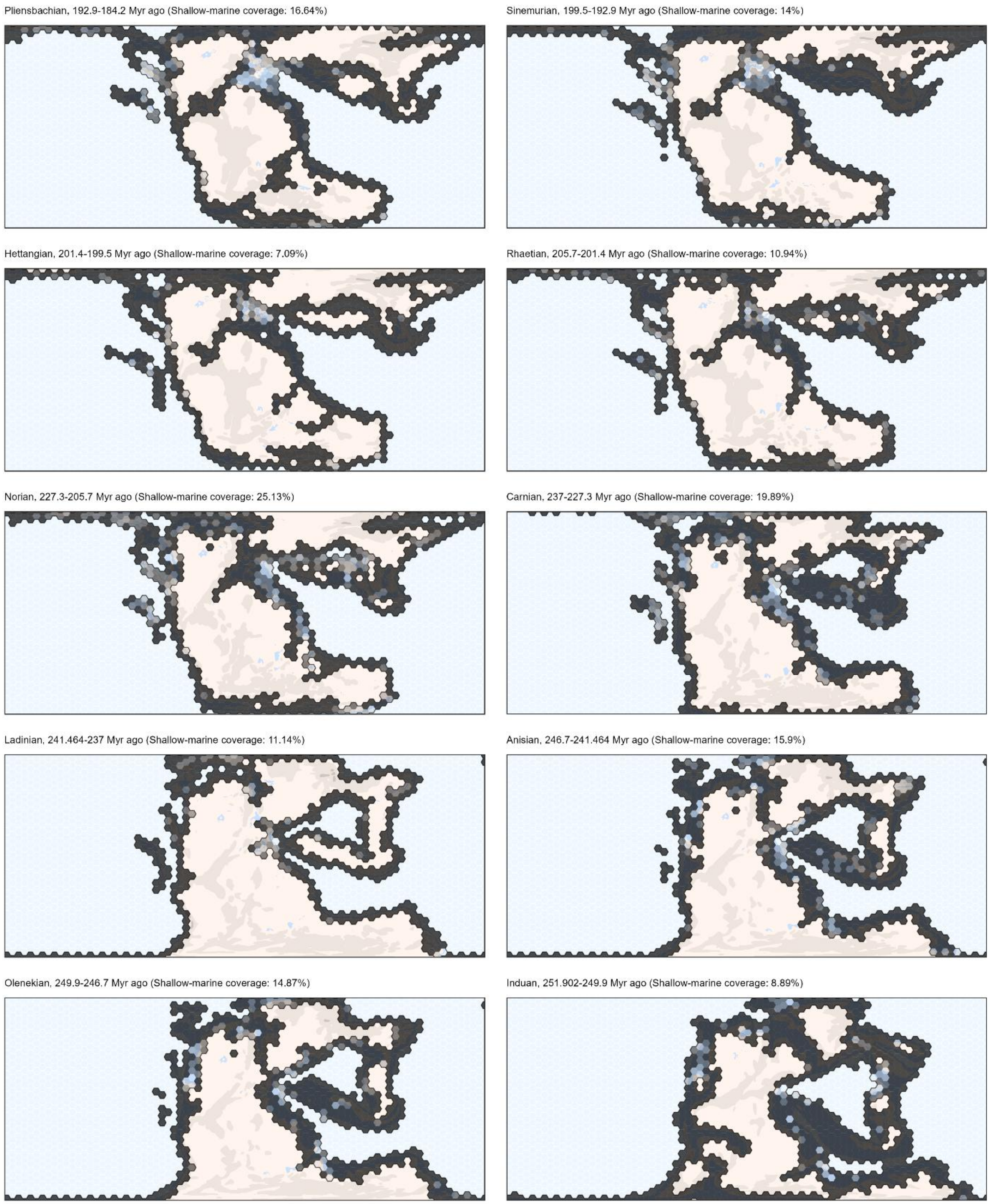

Fig. S4. Continued.

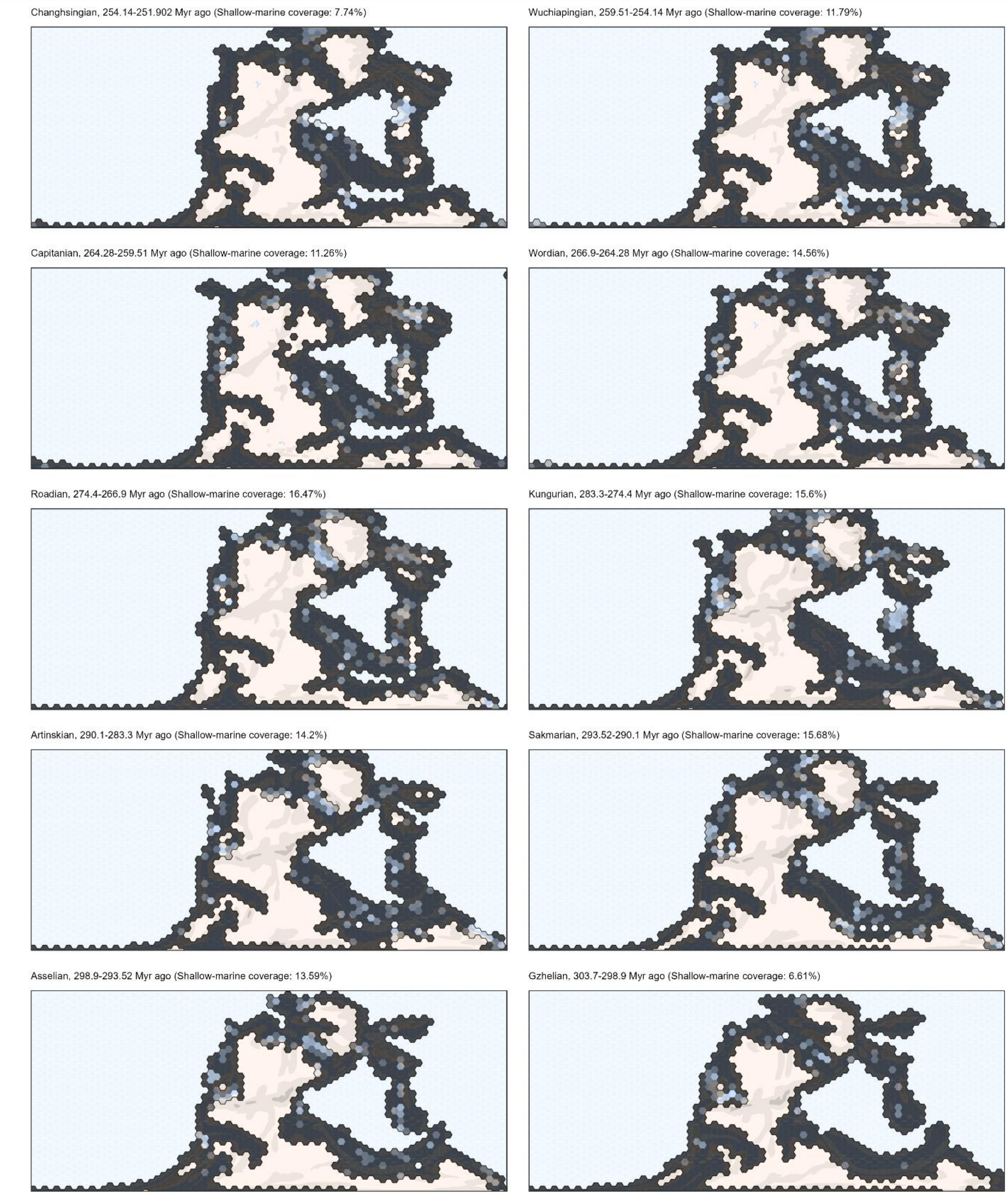

Fig. S4. Continued.

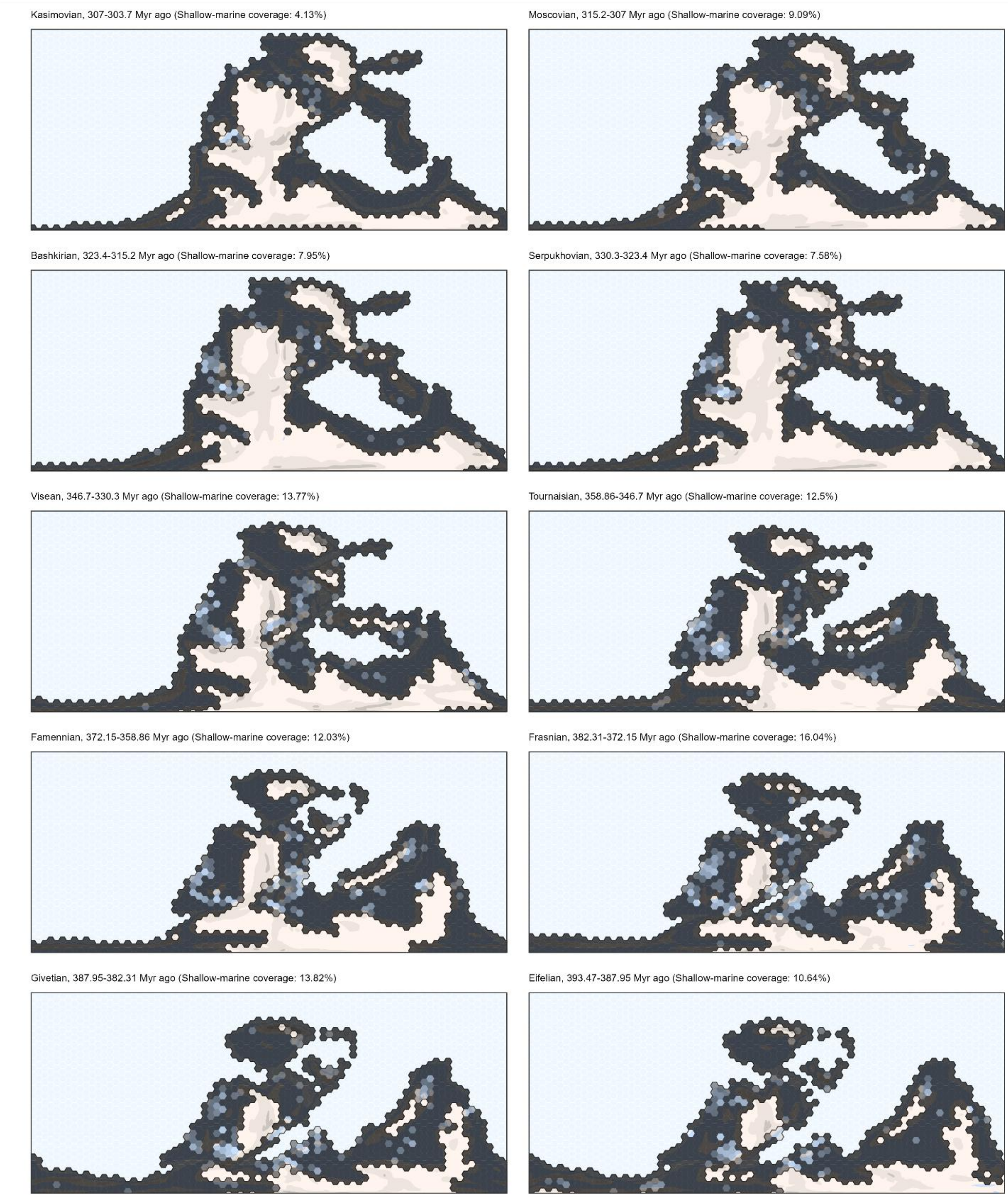

Fig. S4. Continued.

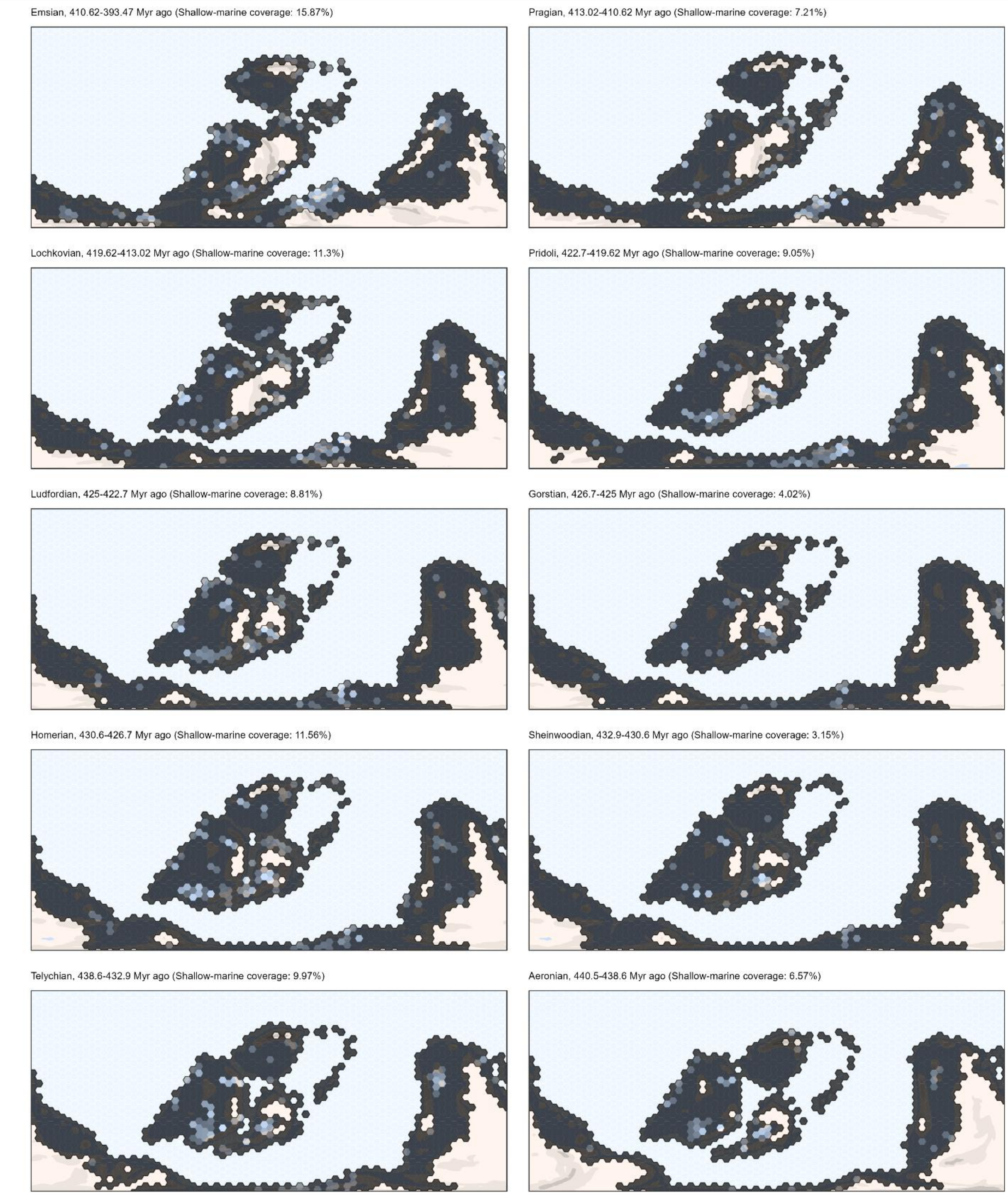

Fig. S4. Continued.

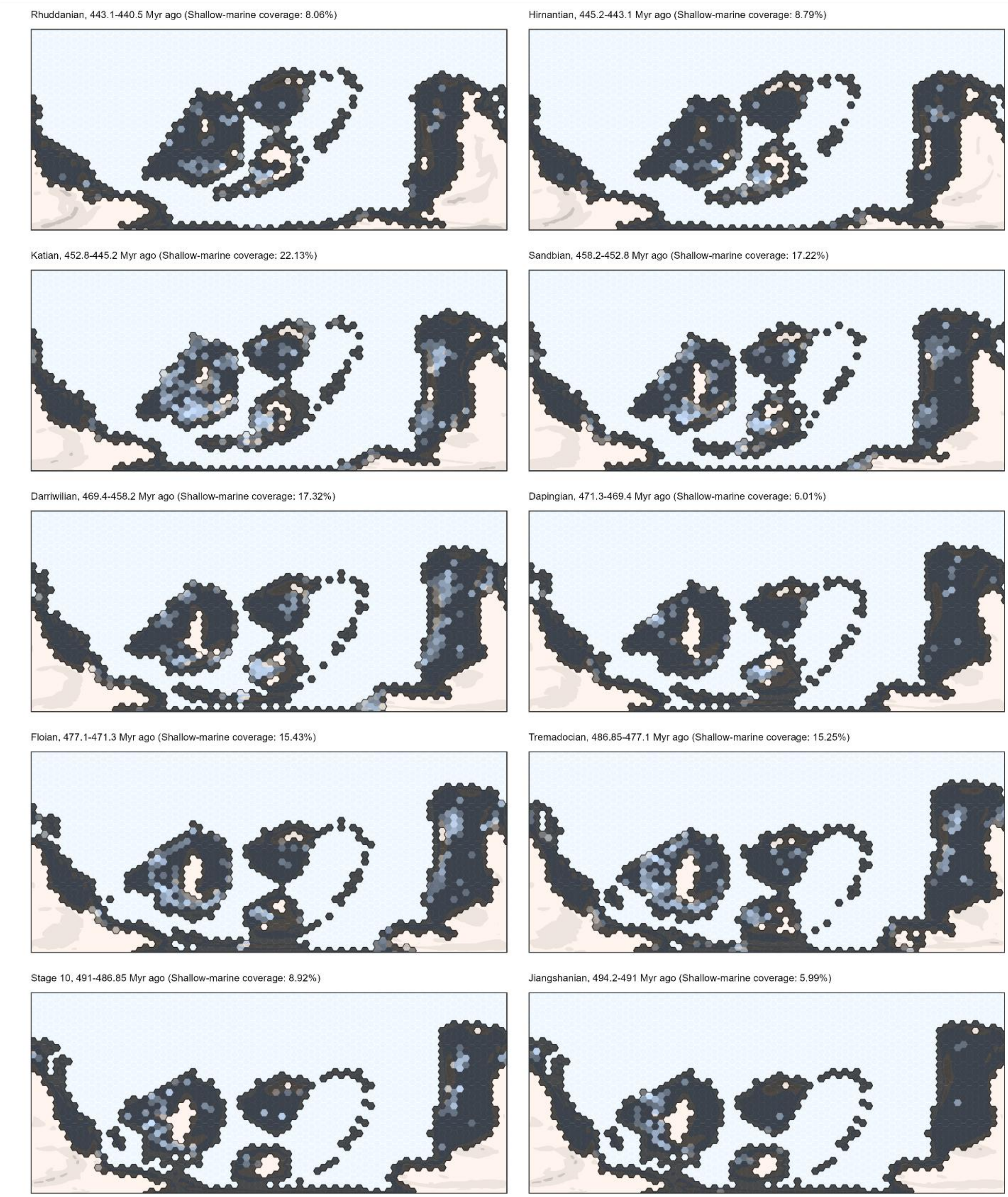

Fig. S4. Continued.

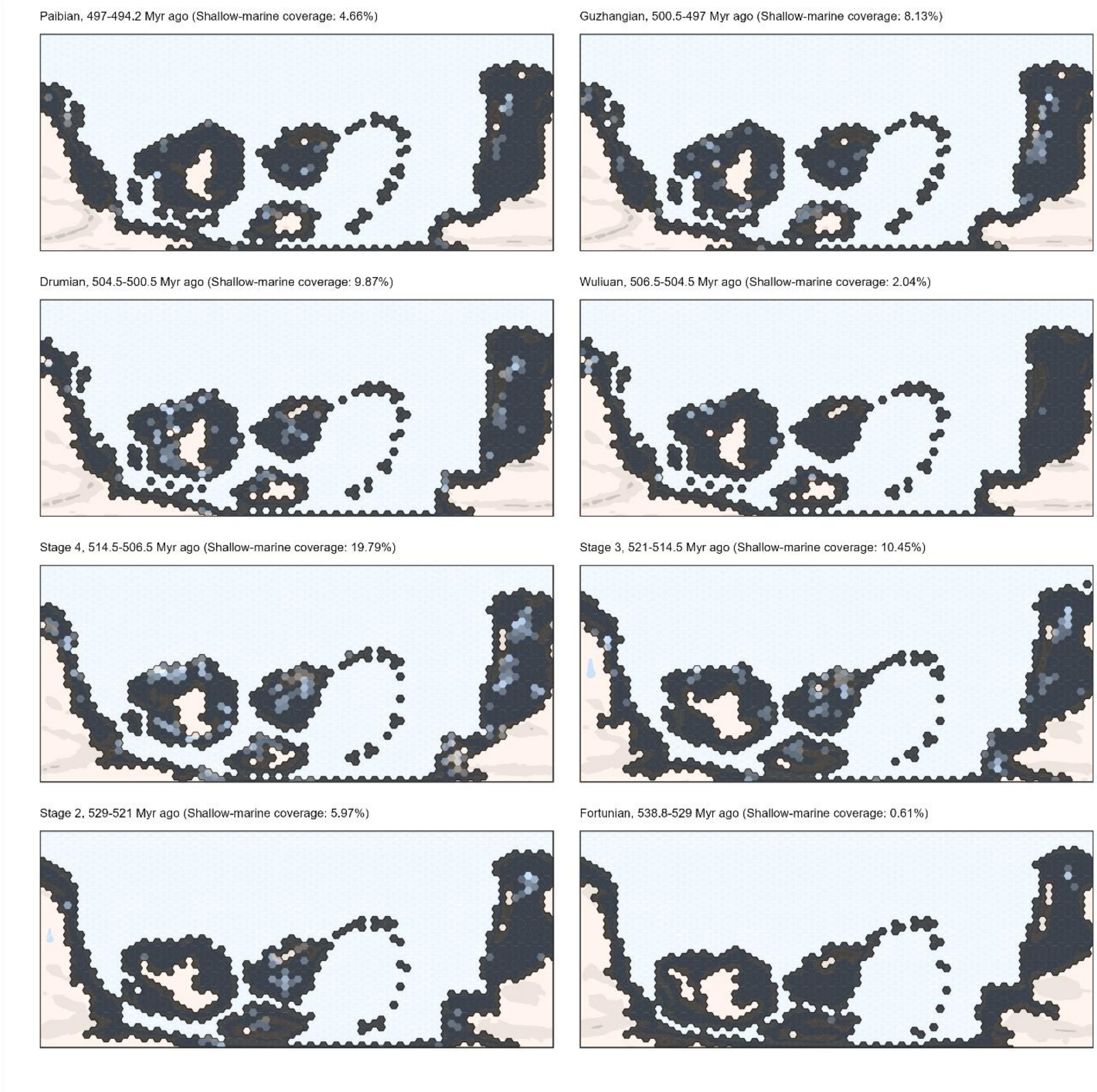

Fig. S4. Continued.

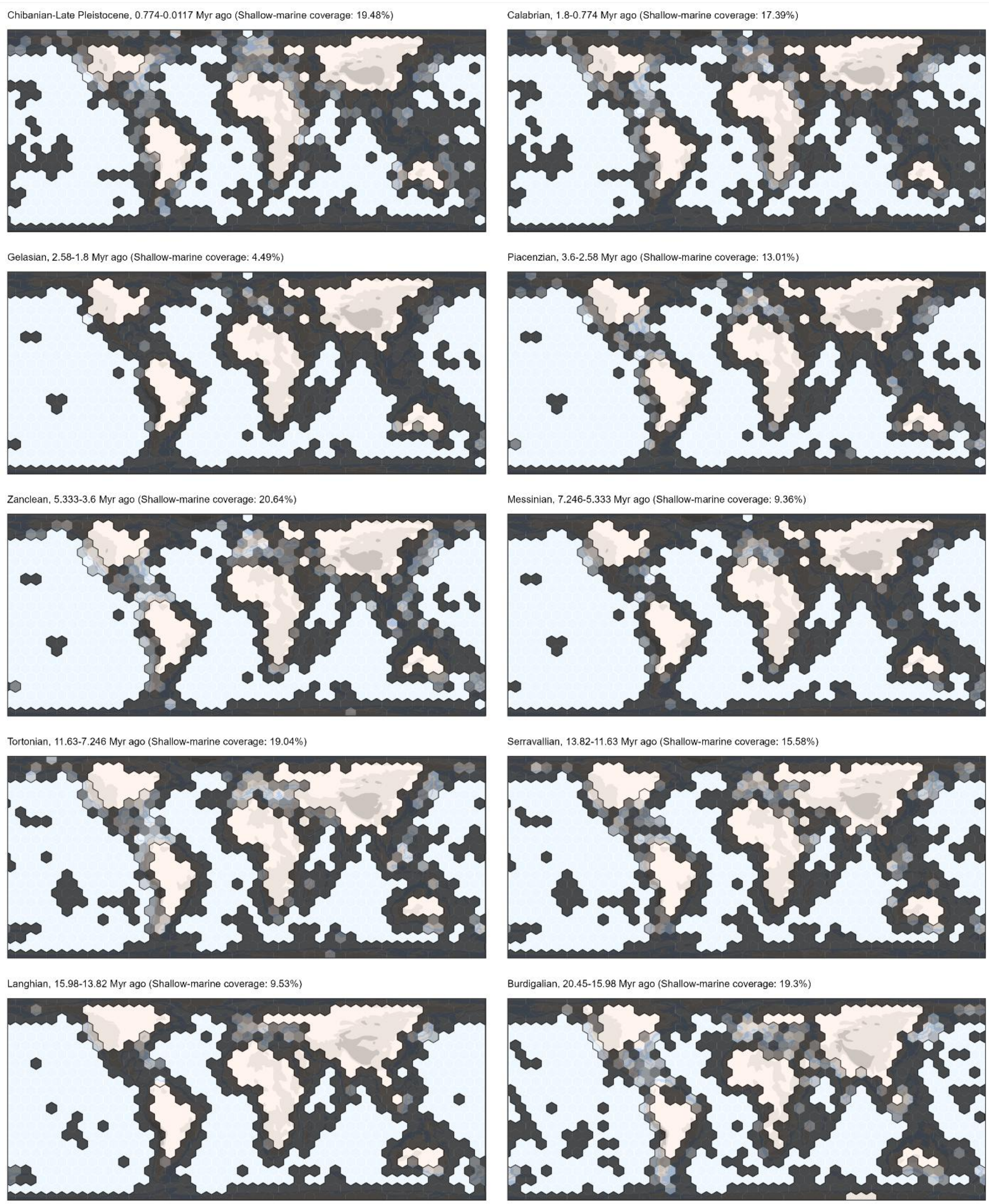

Fig. S5.

Paleogeographic maps showing information density on shallow-marine metazoans as light hexagons for the 750 km pointy-topped grid. The number of fossil collections per grid cell is displayed as relative transparency (see Figure 1 in main text for legend). Corresponding data on coverage is summarized in table S4.

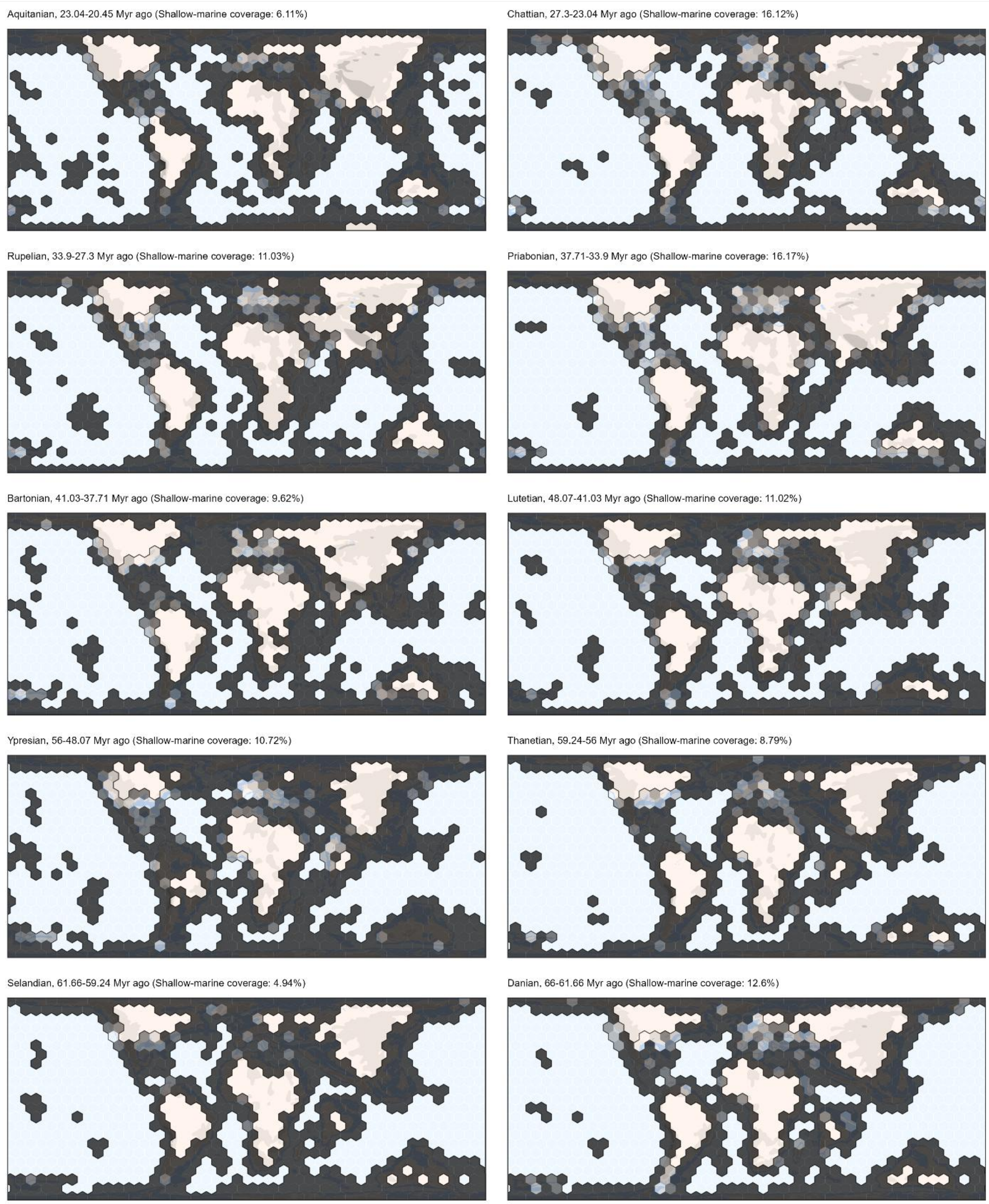

Fig. S5. Continued.

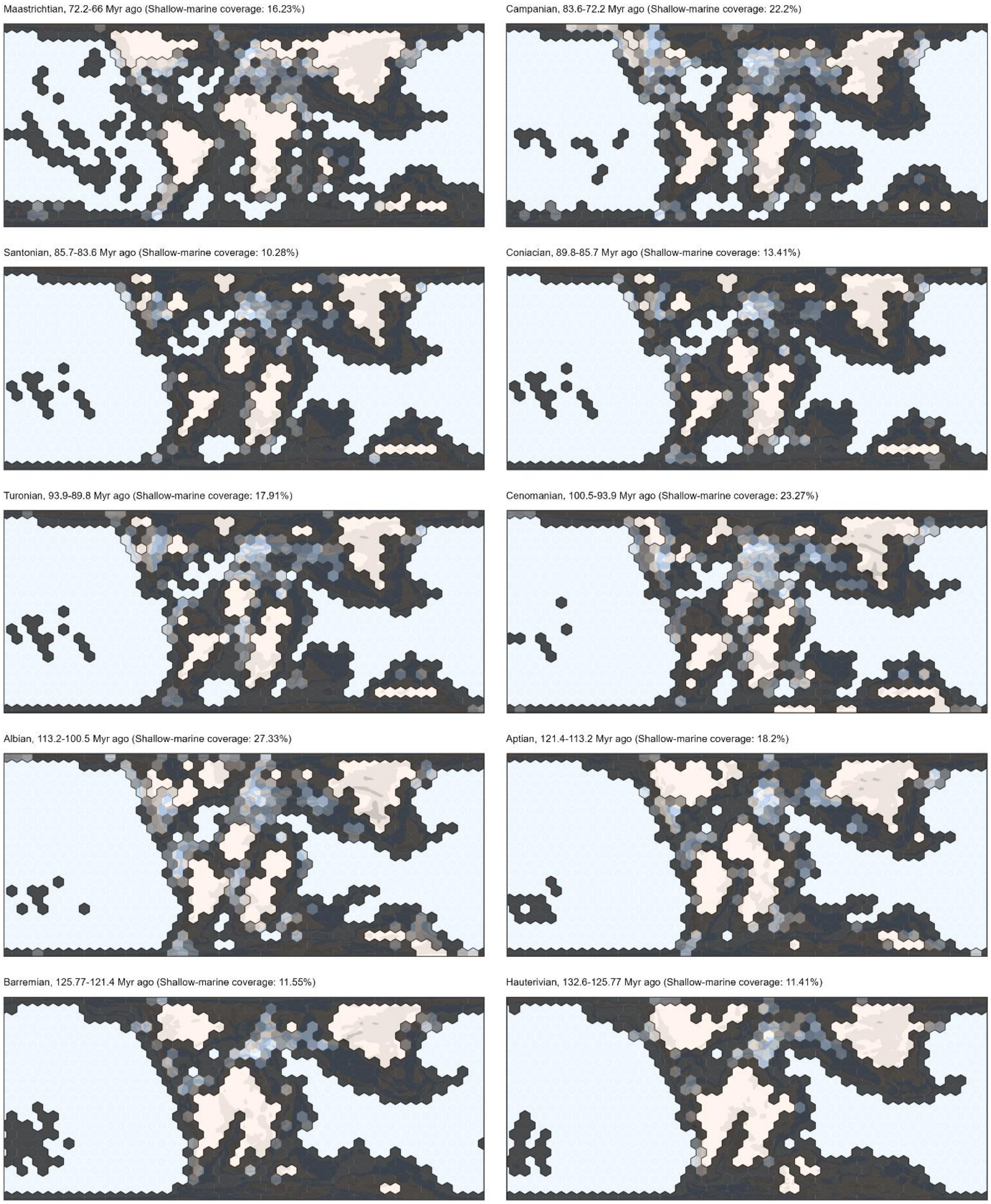

Fig. S5. Continued.

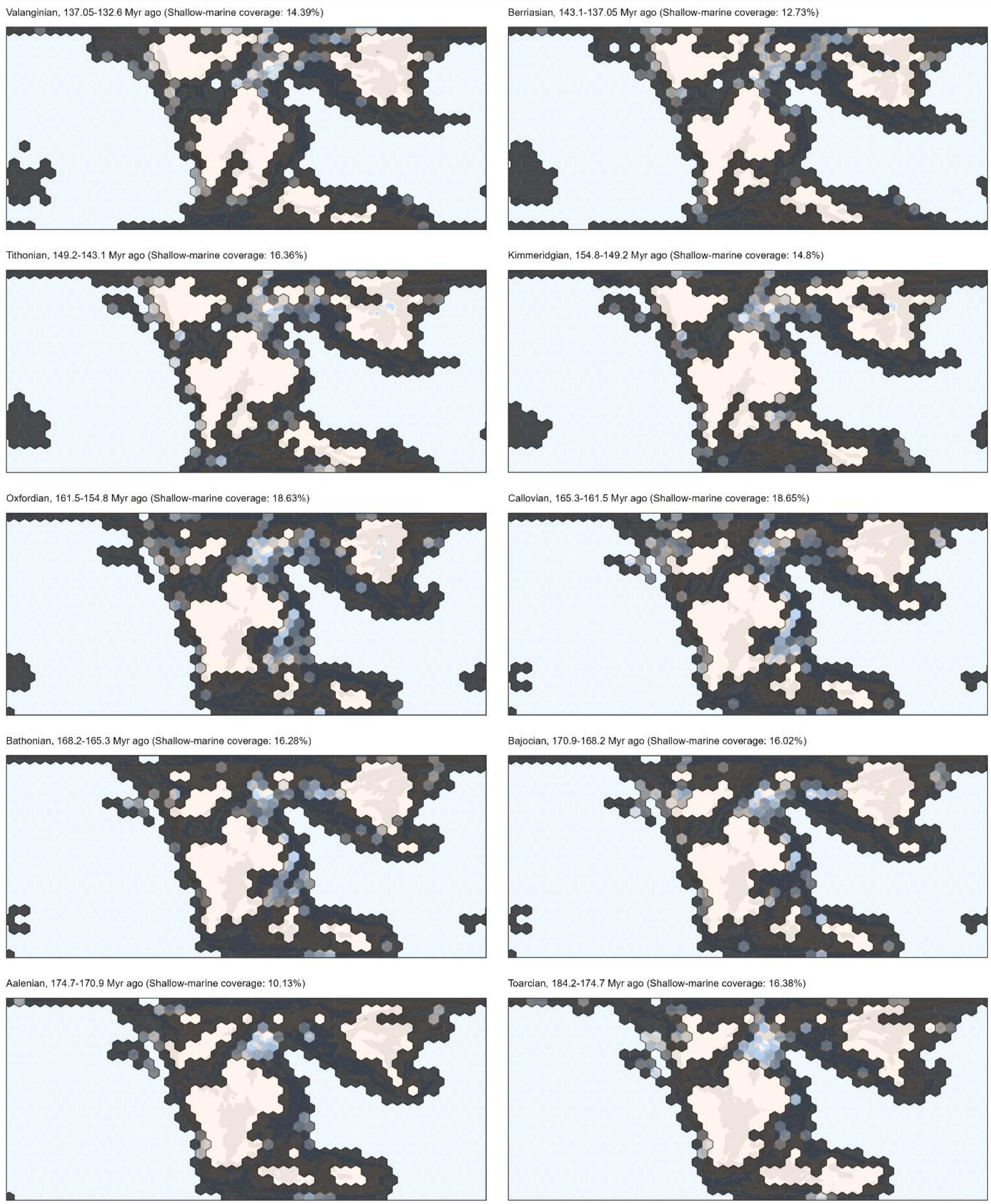

Fig. S5. Continued.

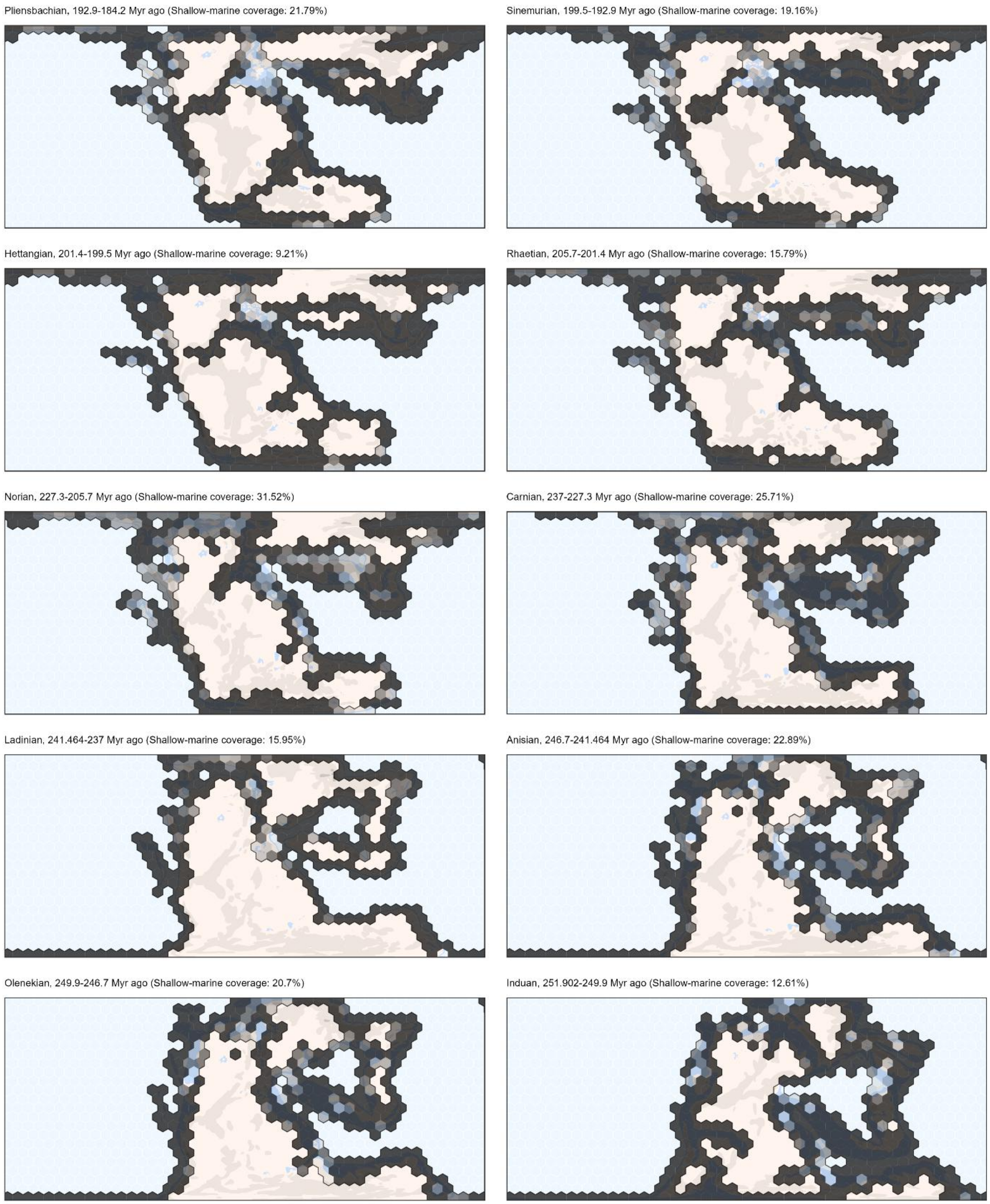

Fig. S5. Continued.

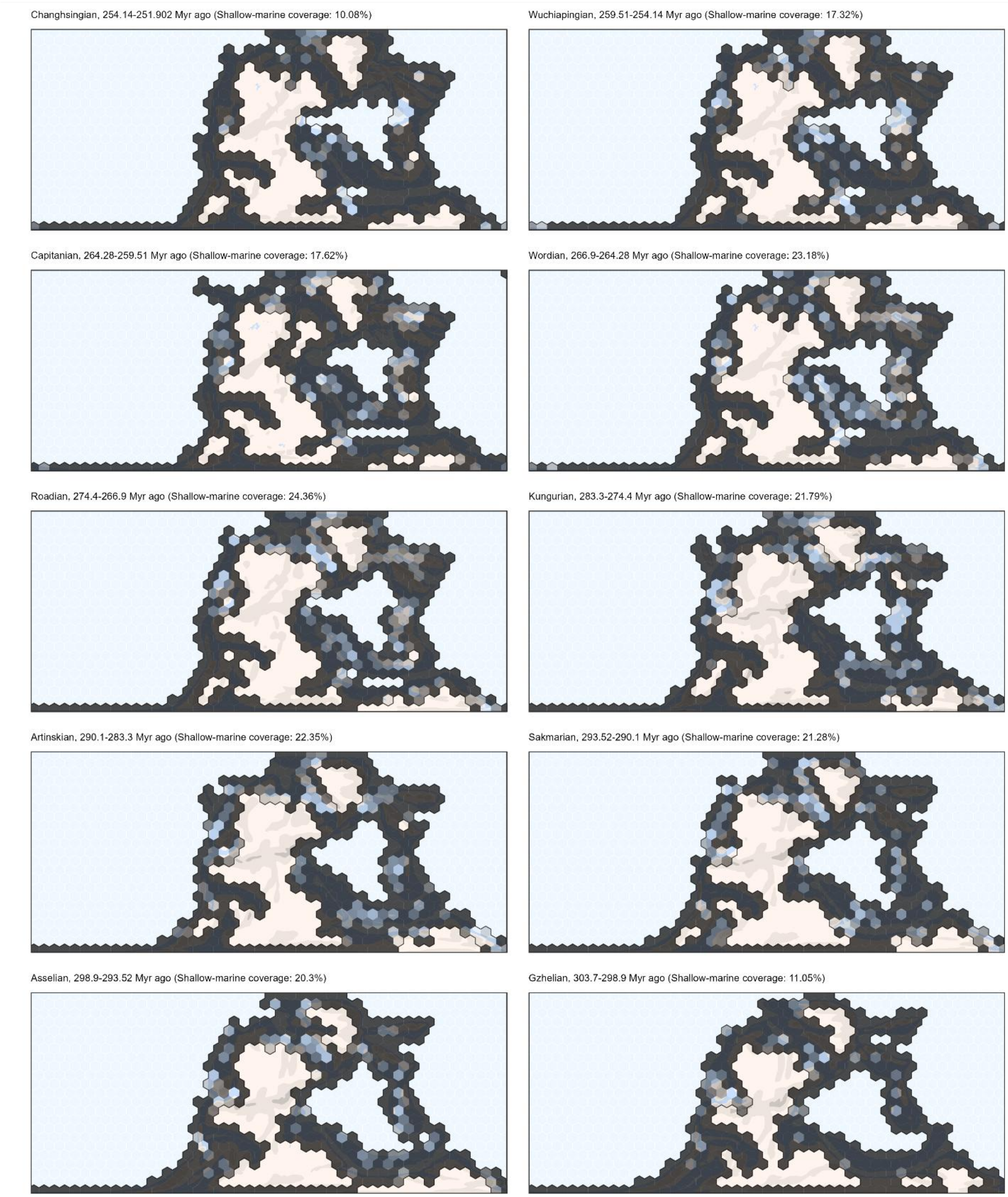

Fig. S5. Continued.

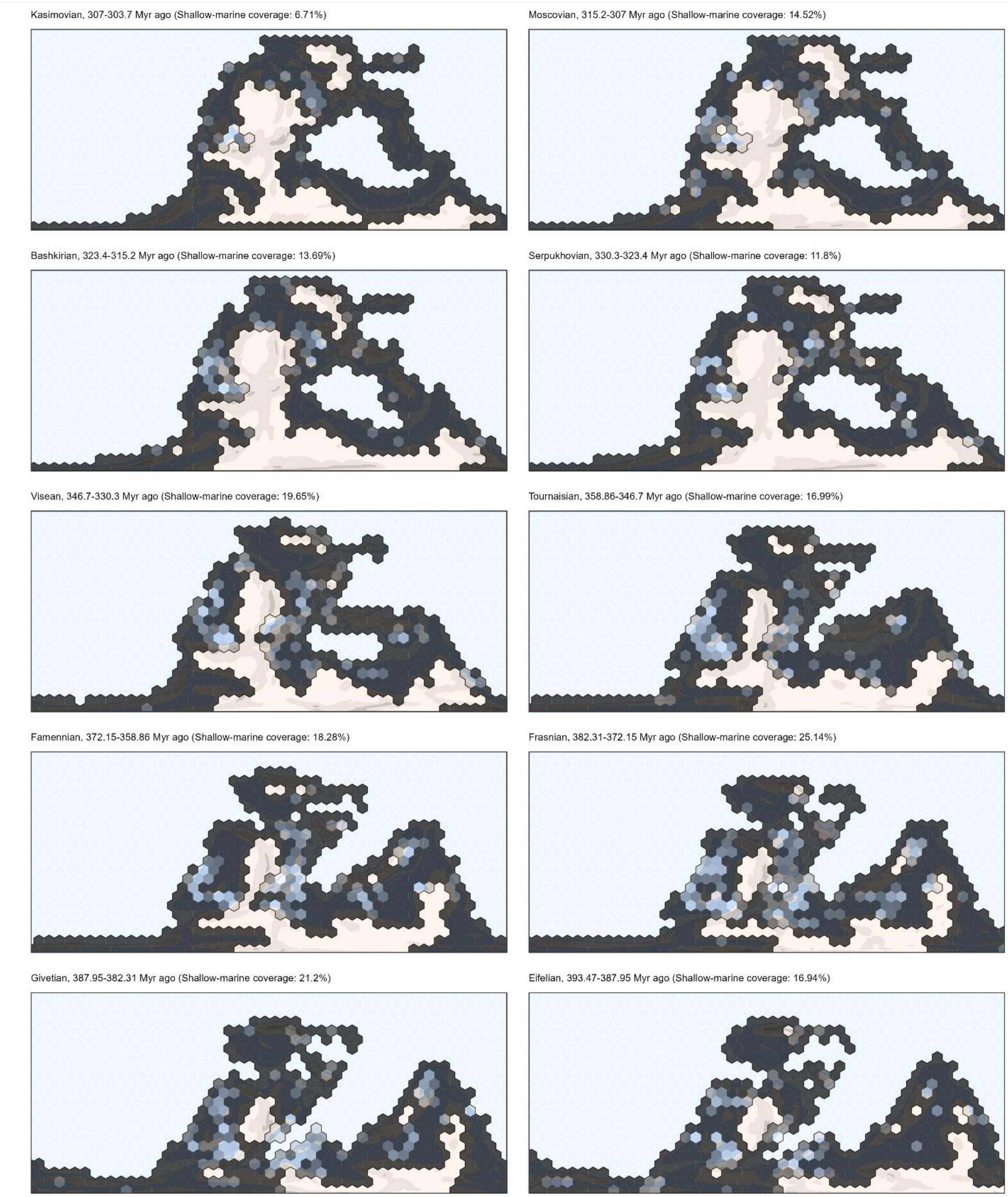

Fig. S5. Continued.

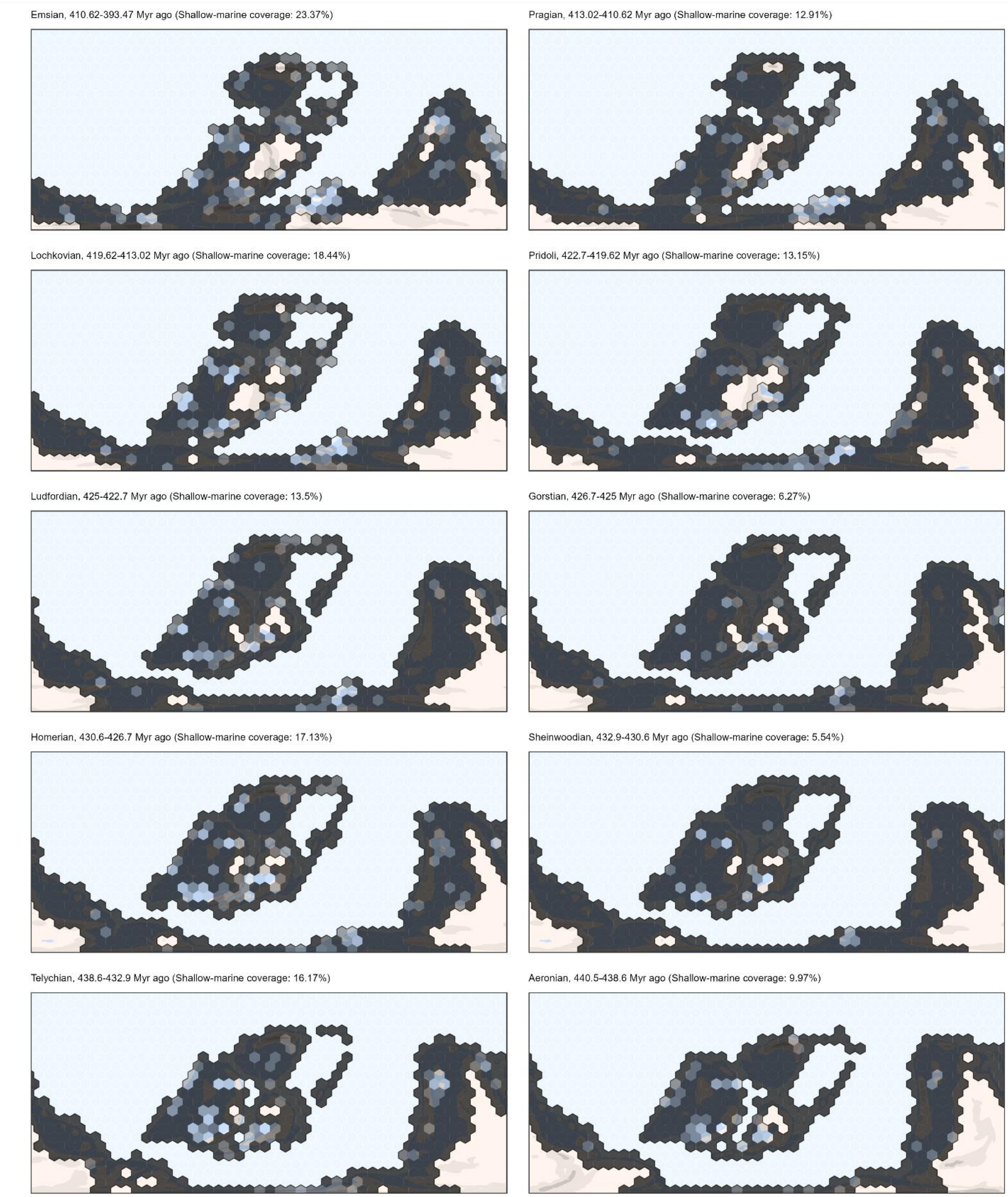

Fig. S5. Continued.

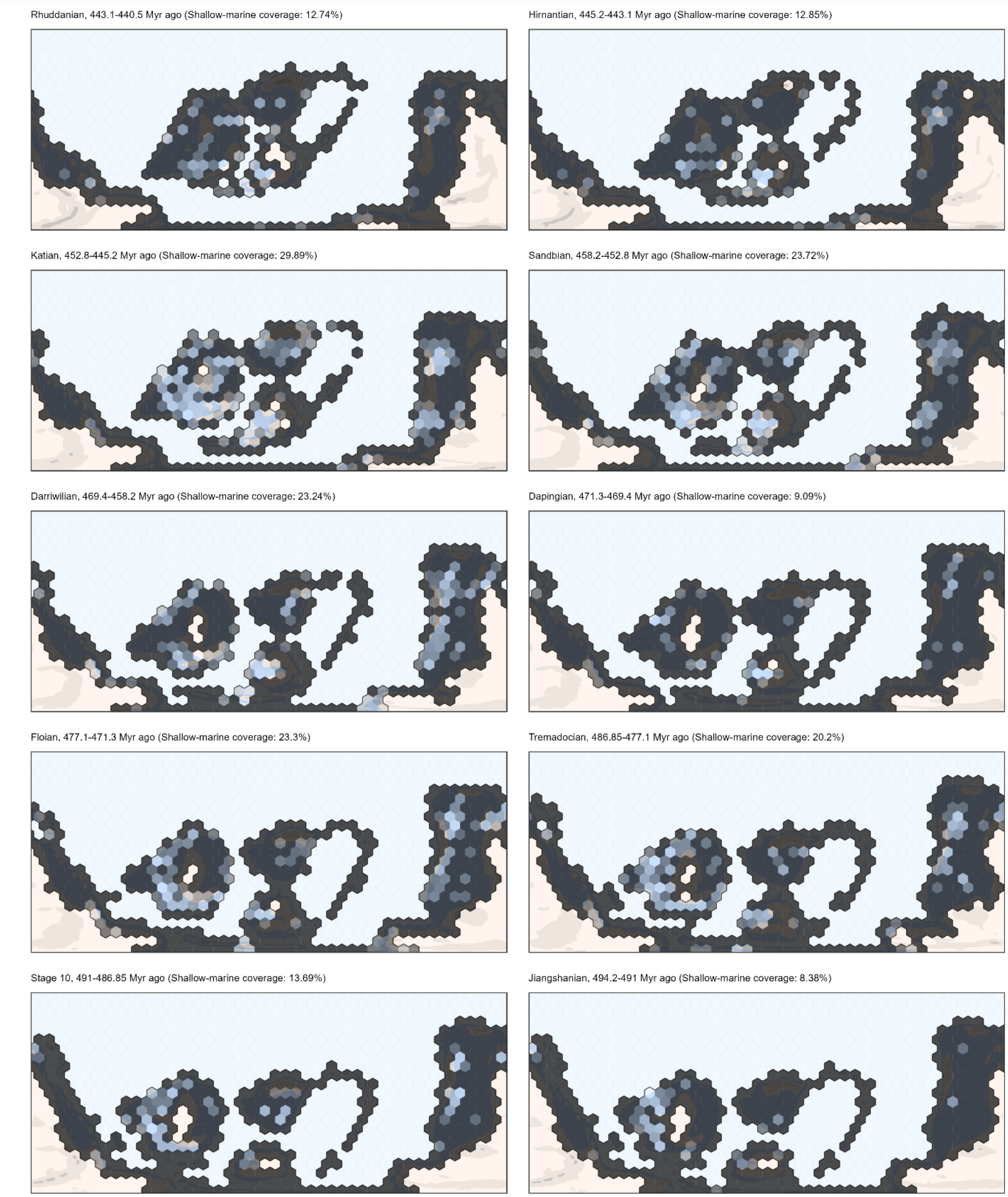

Fig. S5. Continued.

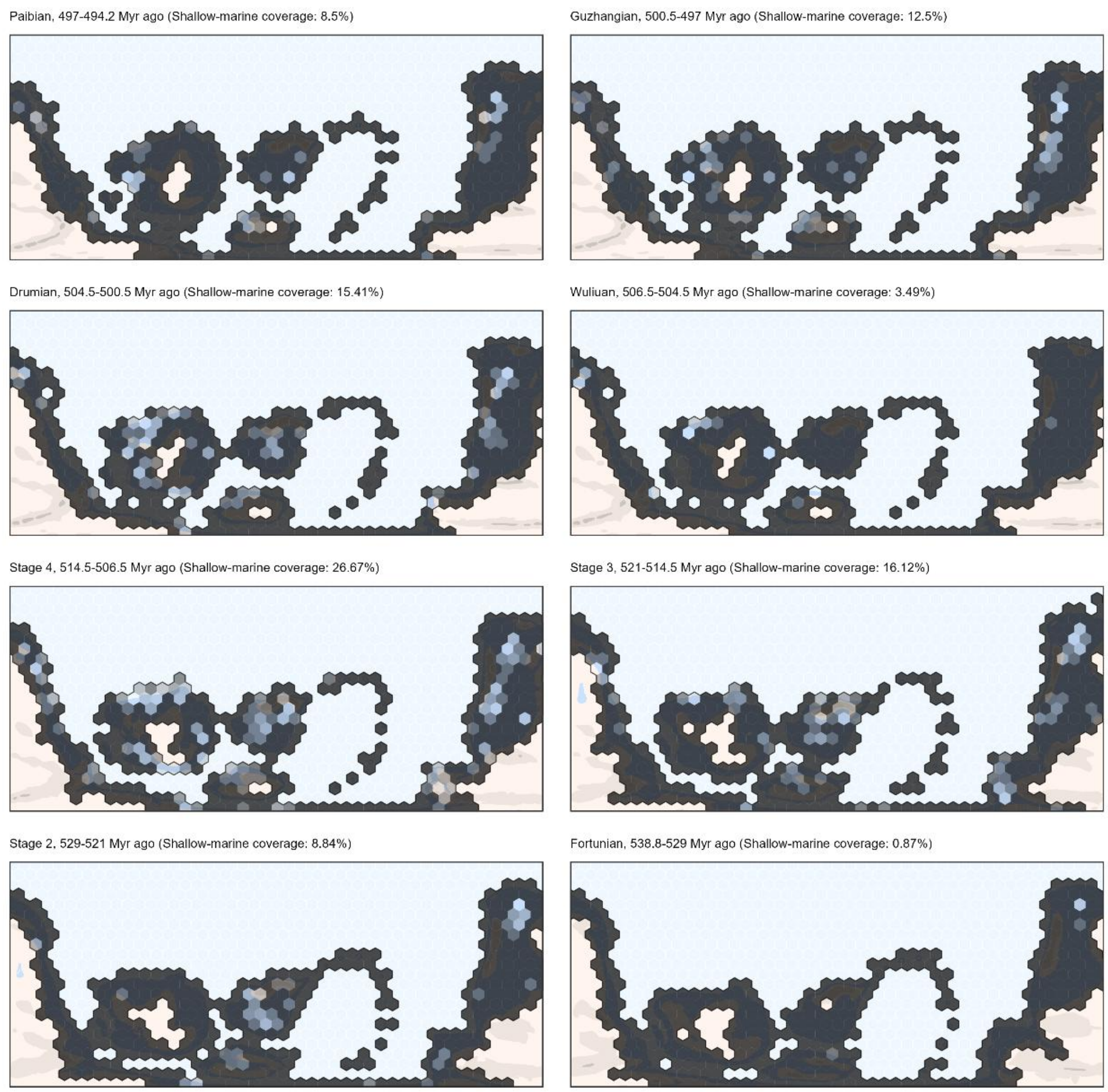

Fig. S5. Continued.

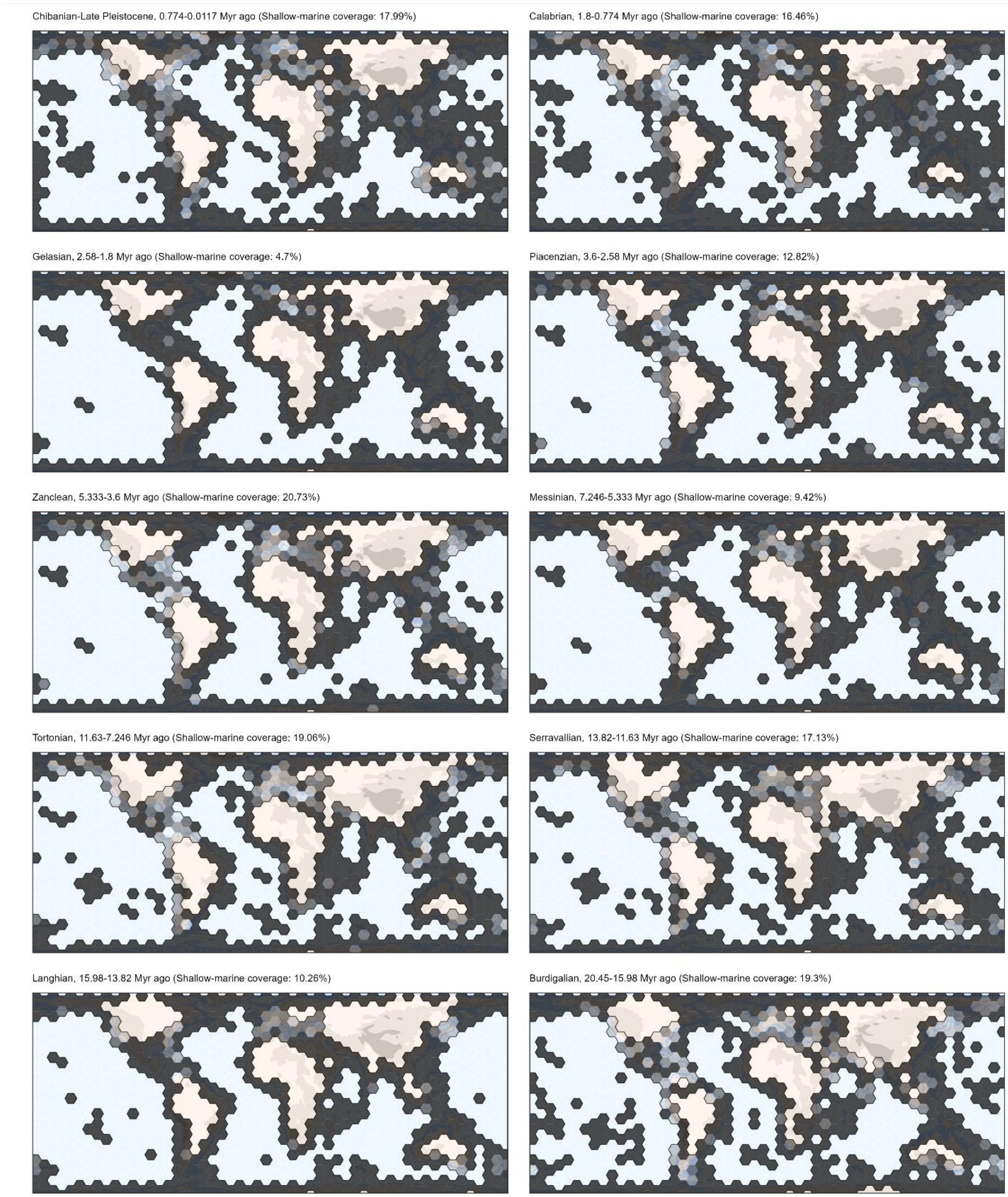

Fig. S6.

Paleogeographic maps showing information density on shallow-marine metazoans as light hexagons for the 750 km flat-topped grid. The number of fossil collections per grid cell is displayed as relative transparency (see Figure 1 in main text for legend). Corresponding data on coverage is summarized in table S4.

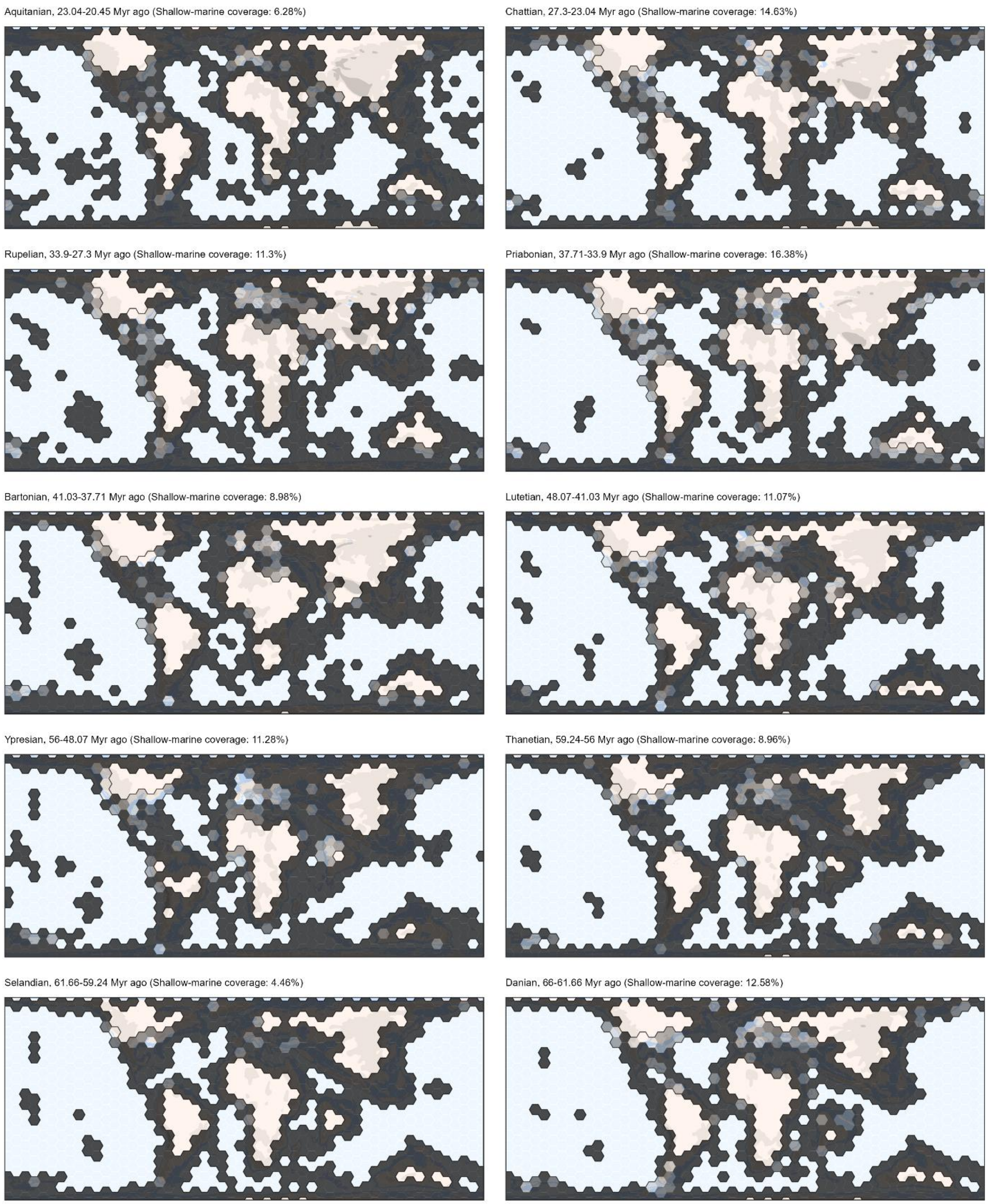

Fig. S6. Continued.

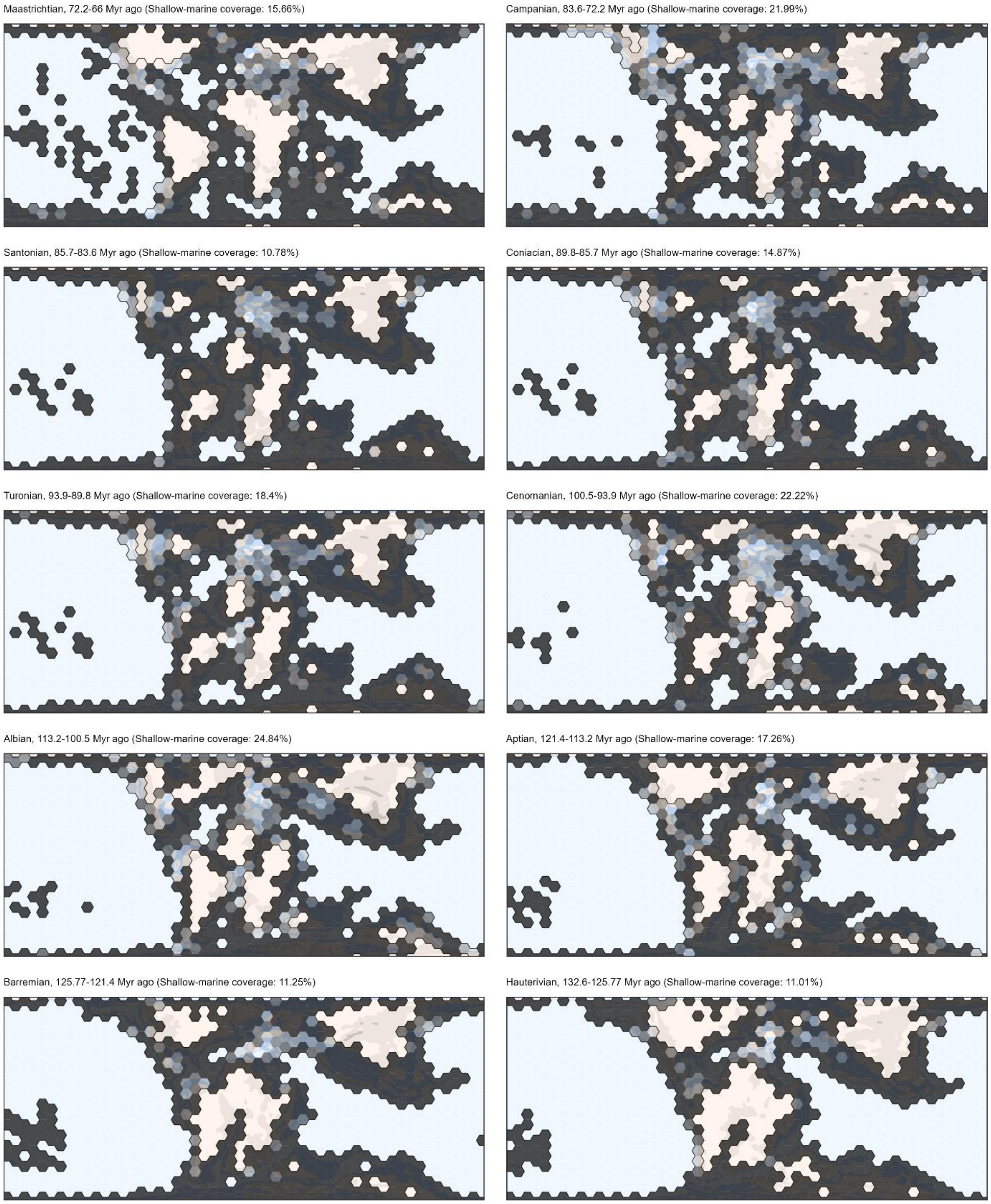

Fig. S6. Continued.

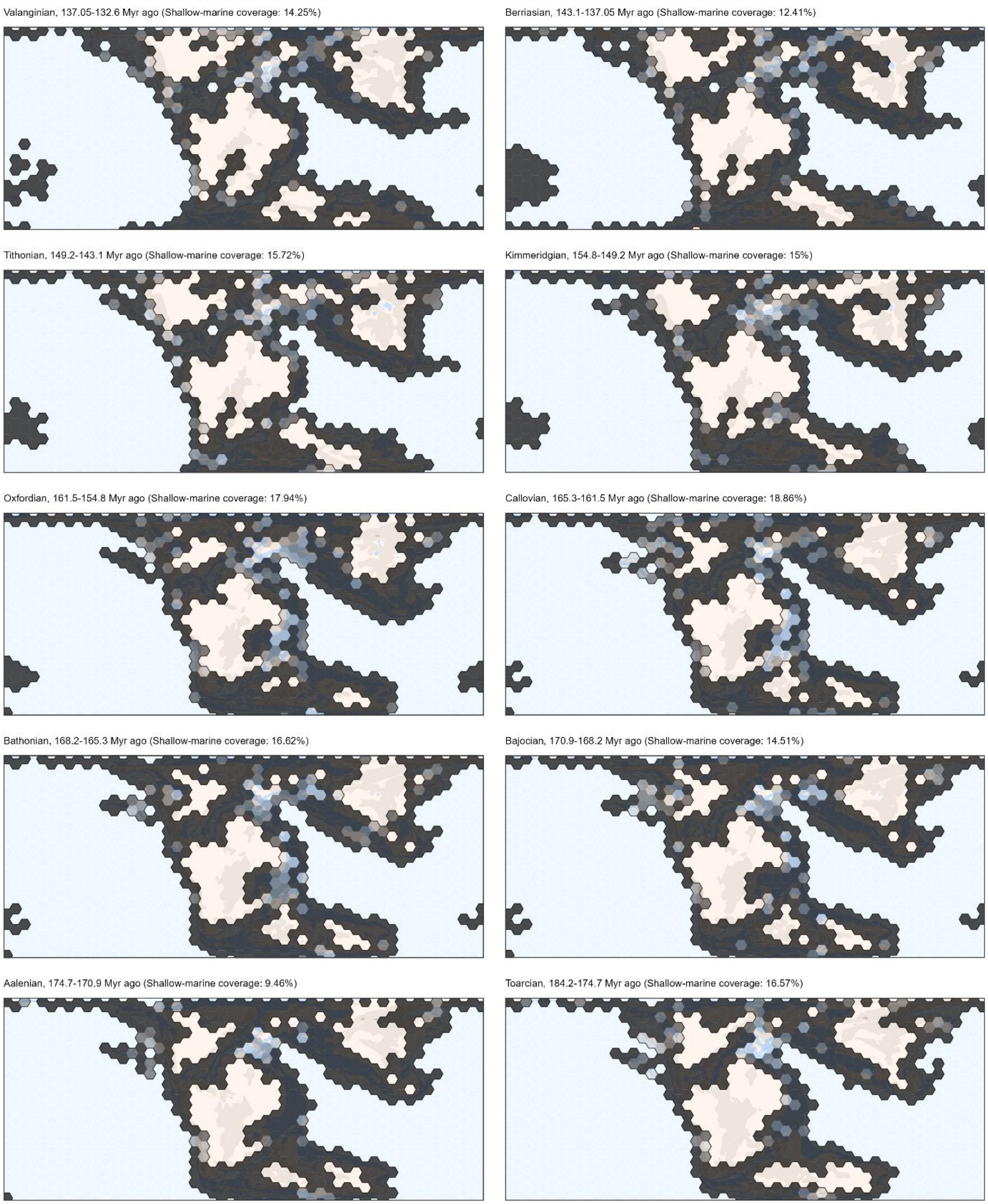

Fig. S6. Continued.

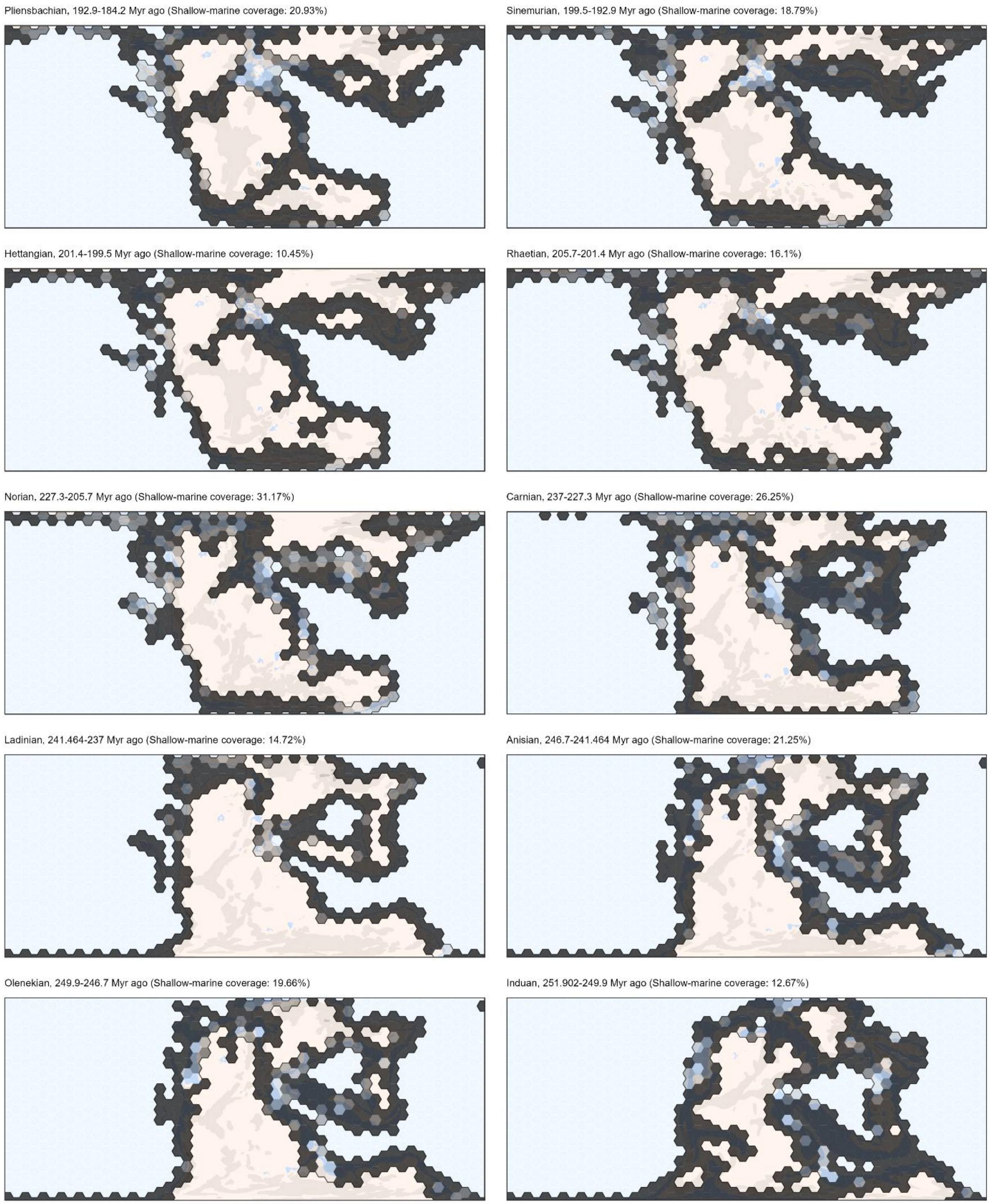

Fig. S6. Continued.

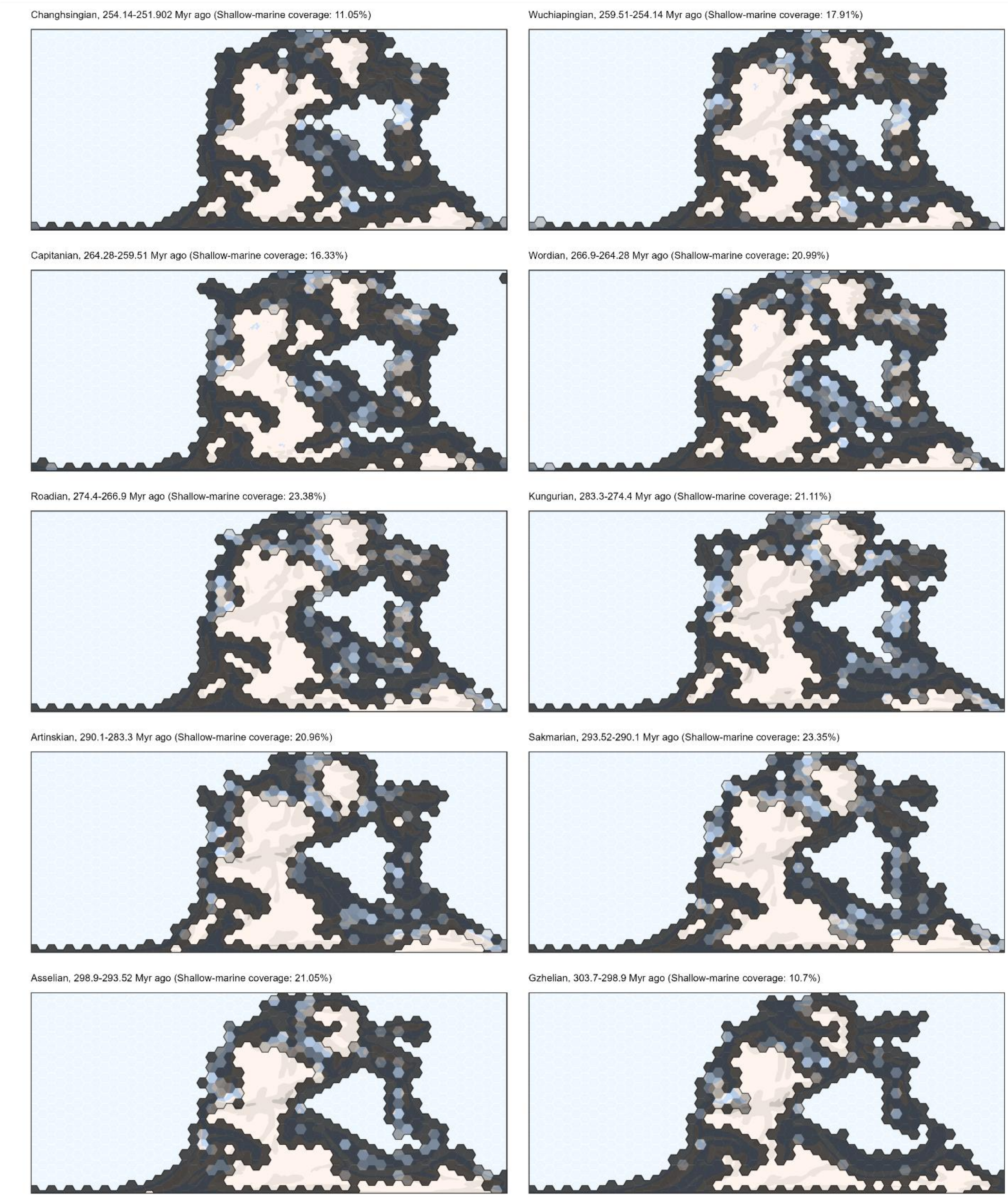

Fig. S6. Continued.

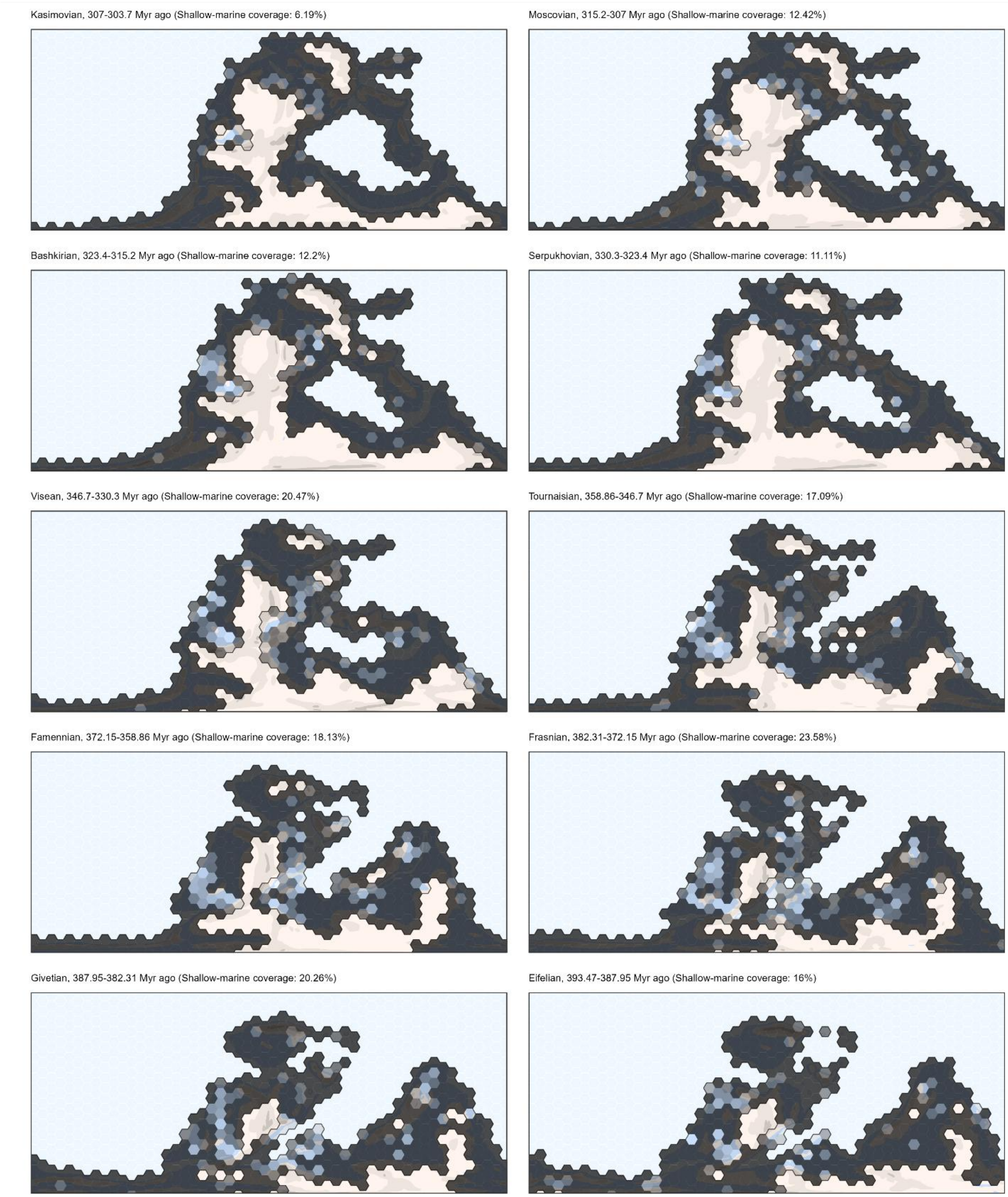

Fig. S6. Continued.

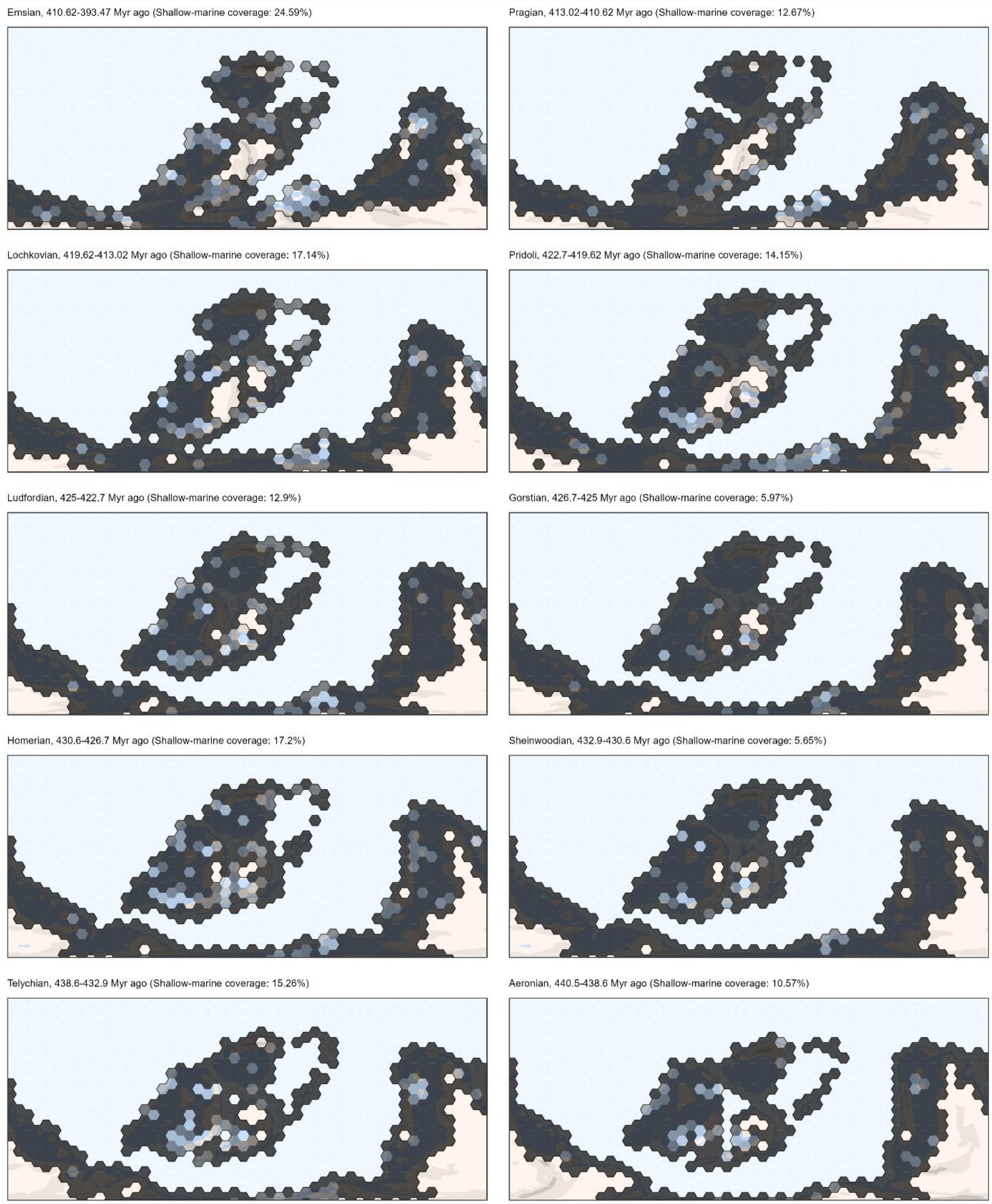

Fig. S6. Continued.

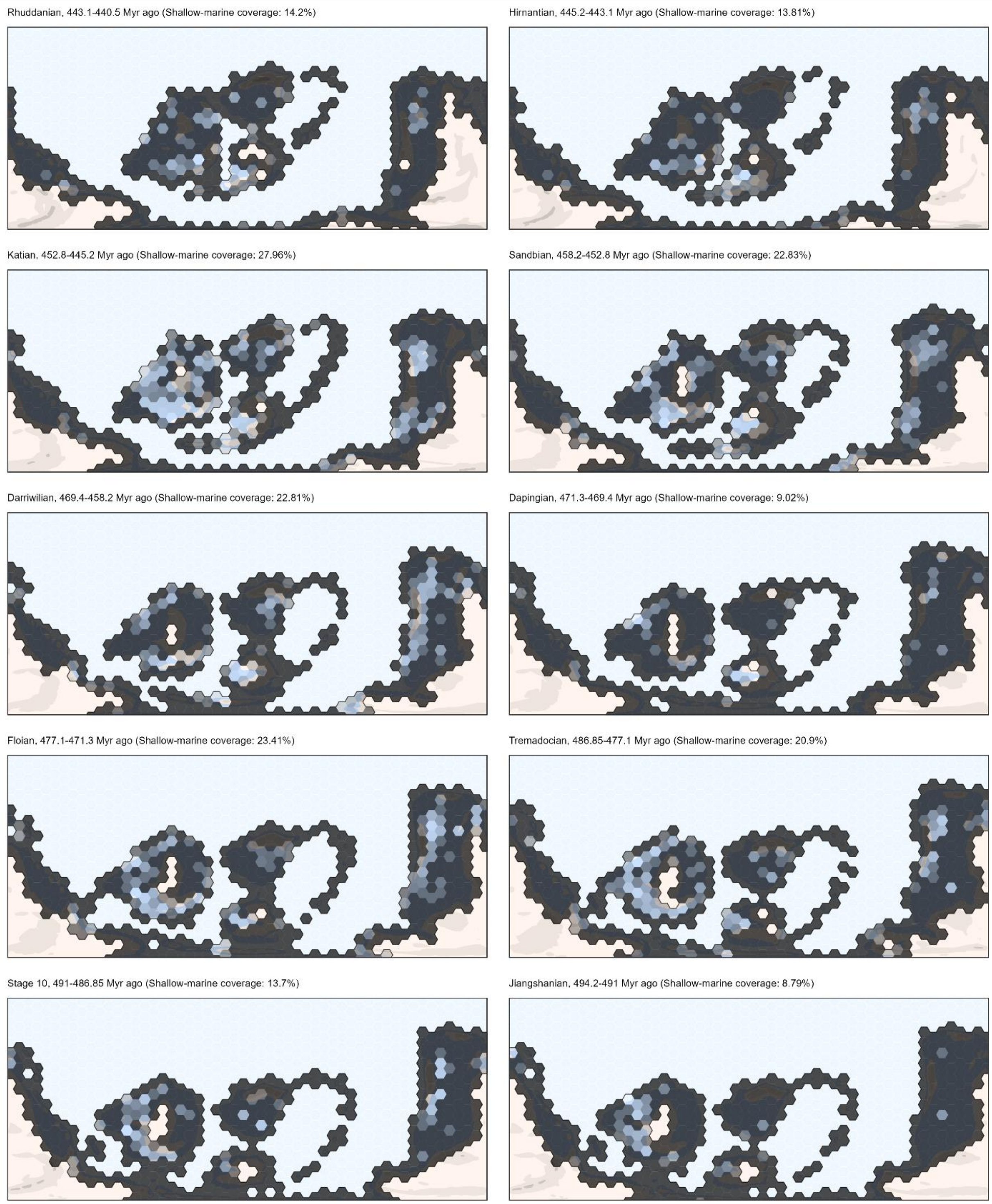

Fig. S6. Continued.

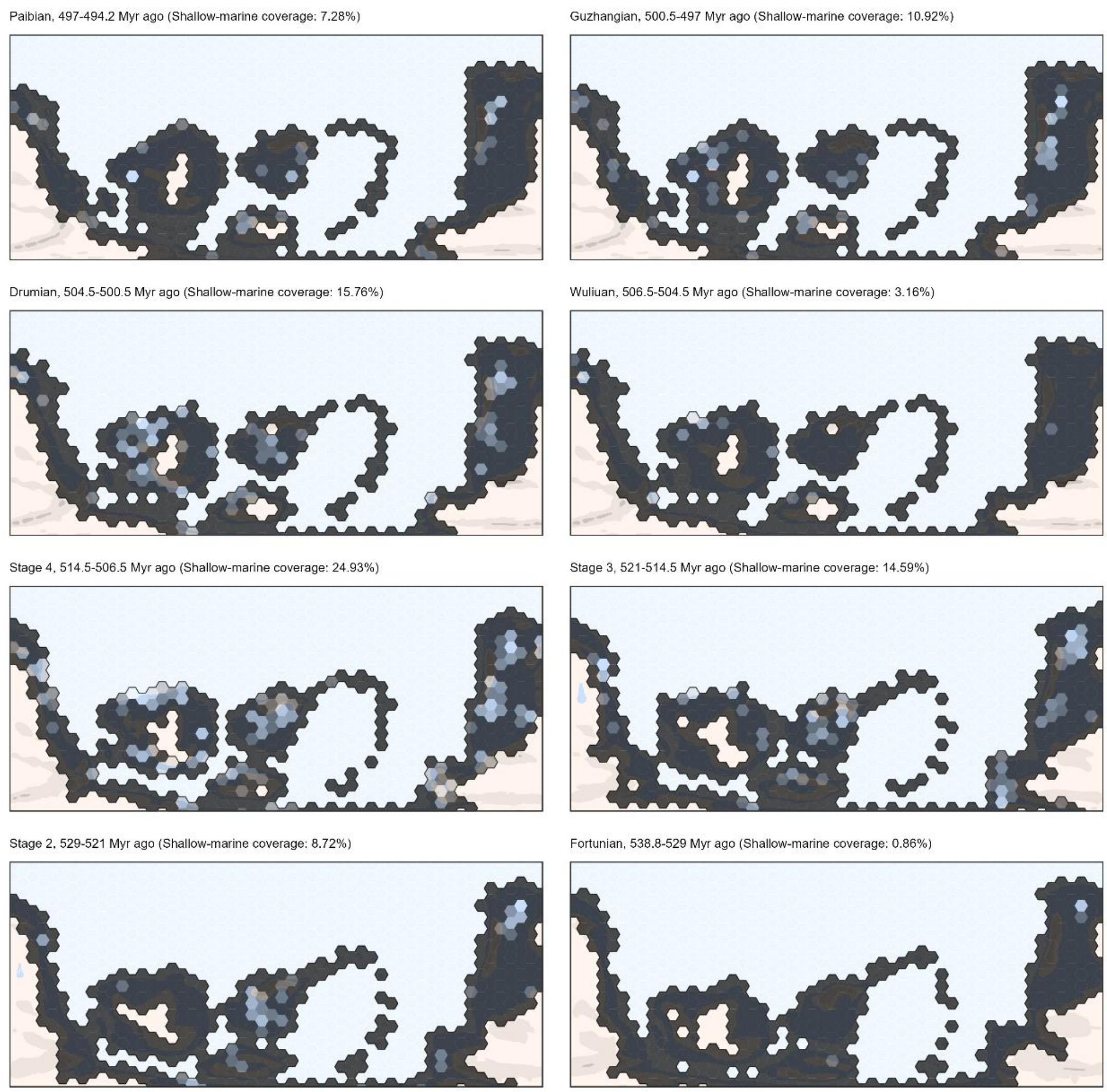

Fig. S6. Continued.

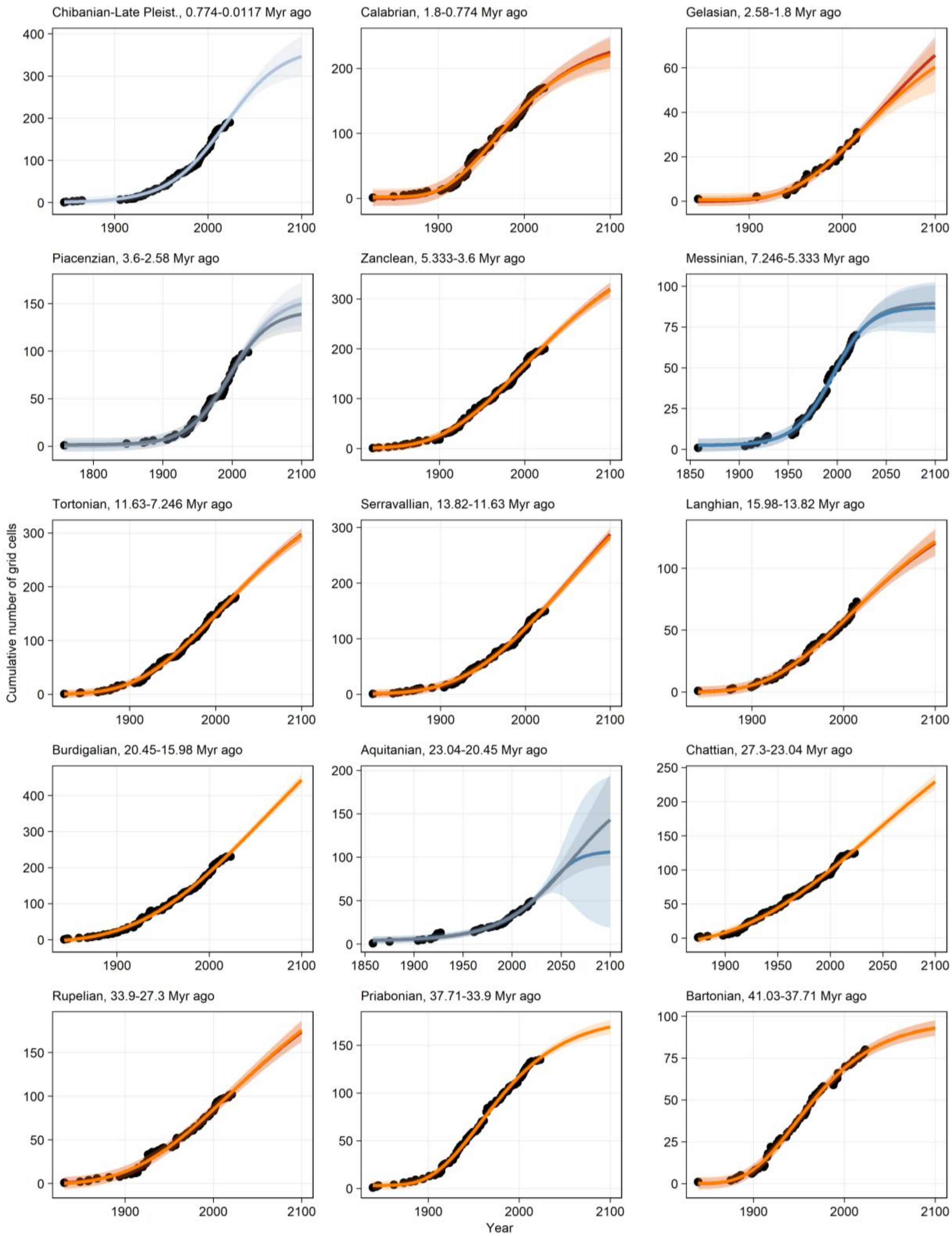

Fig. S7.

Growth models predicting future collection effort across grid cells until the year 2100 for each stage for the 250 km pointy-topped grid. Note that for some stages, more than one model qualified as the best (i.e., within ΔAIC = 2). Underlying data is summarized in table S5.

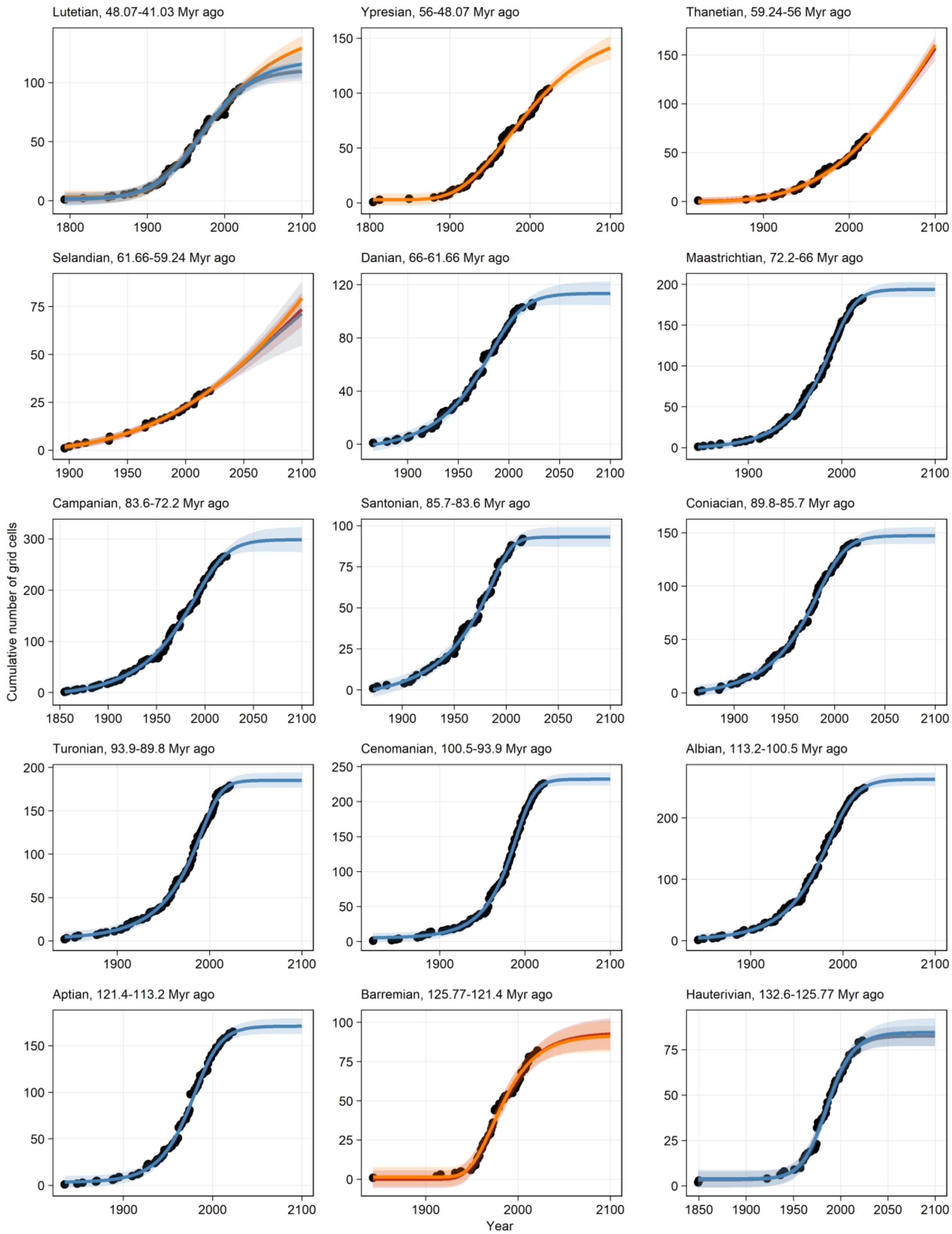

Fig. S7. Continued.

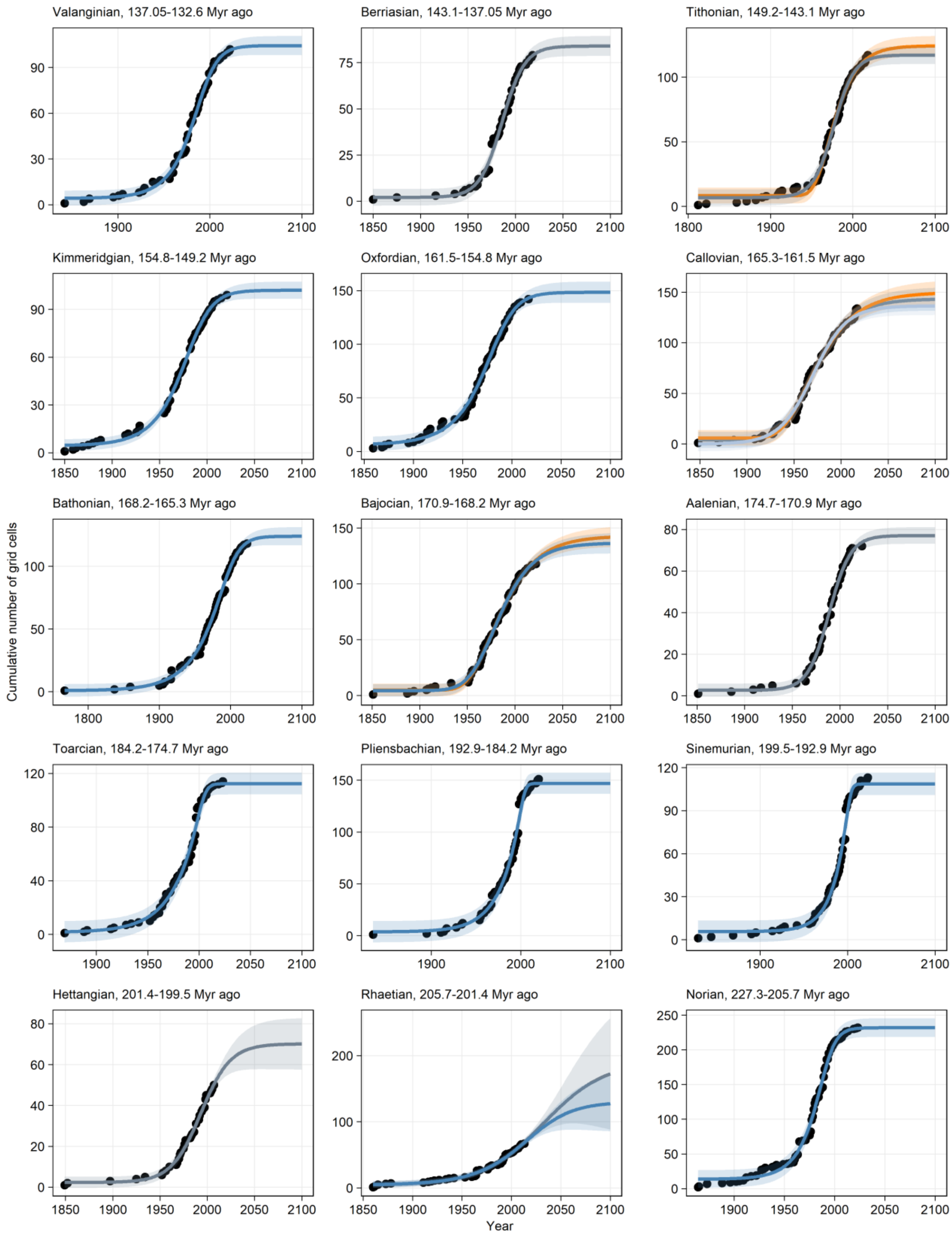

Fig. S7. Continued.

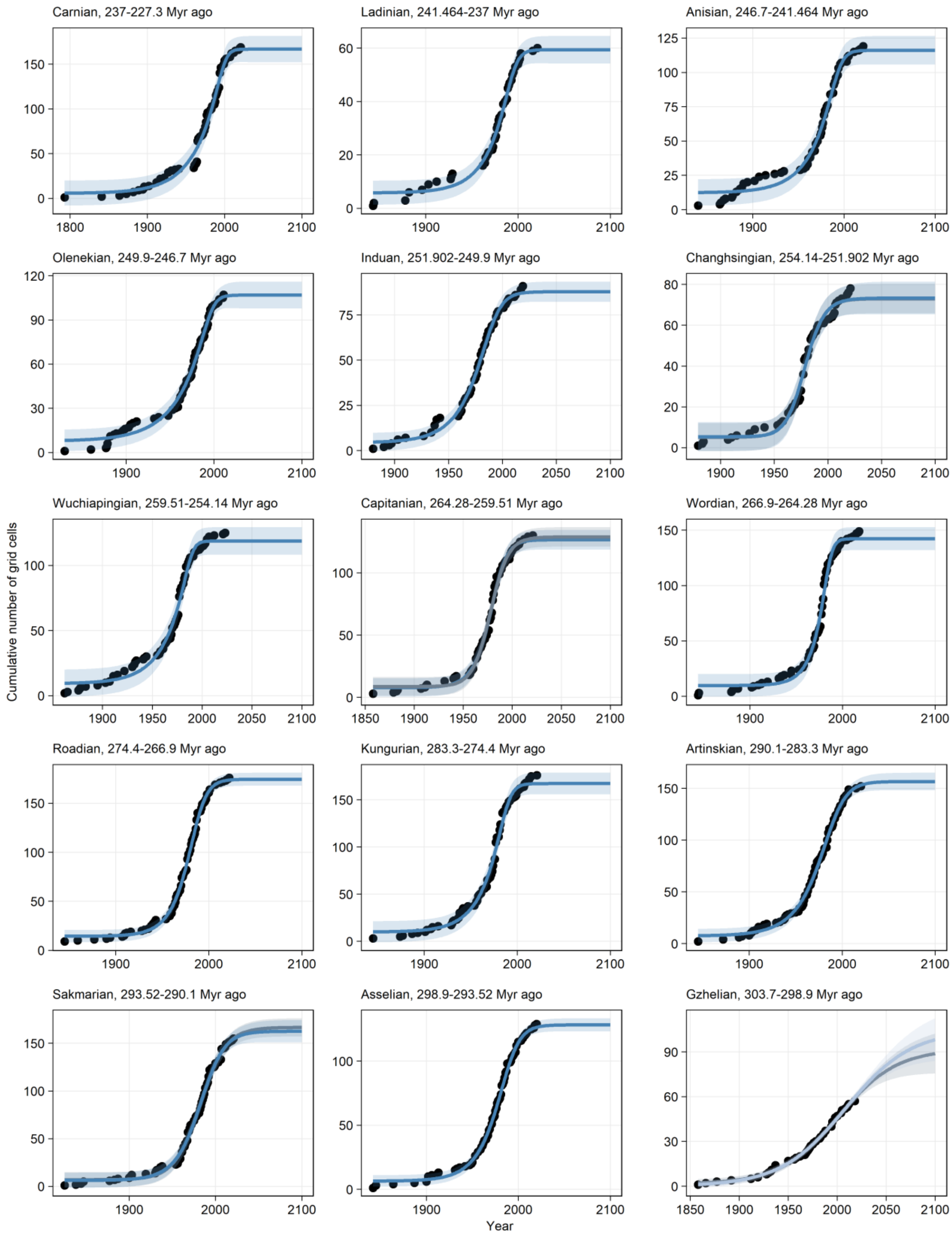

Fig. S7. Continued.

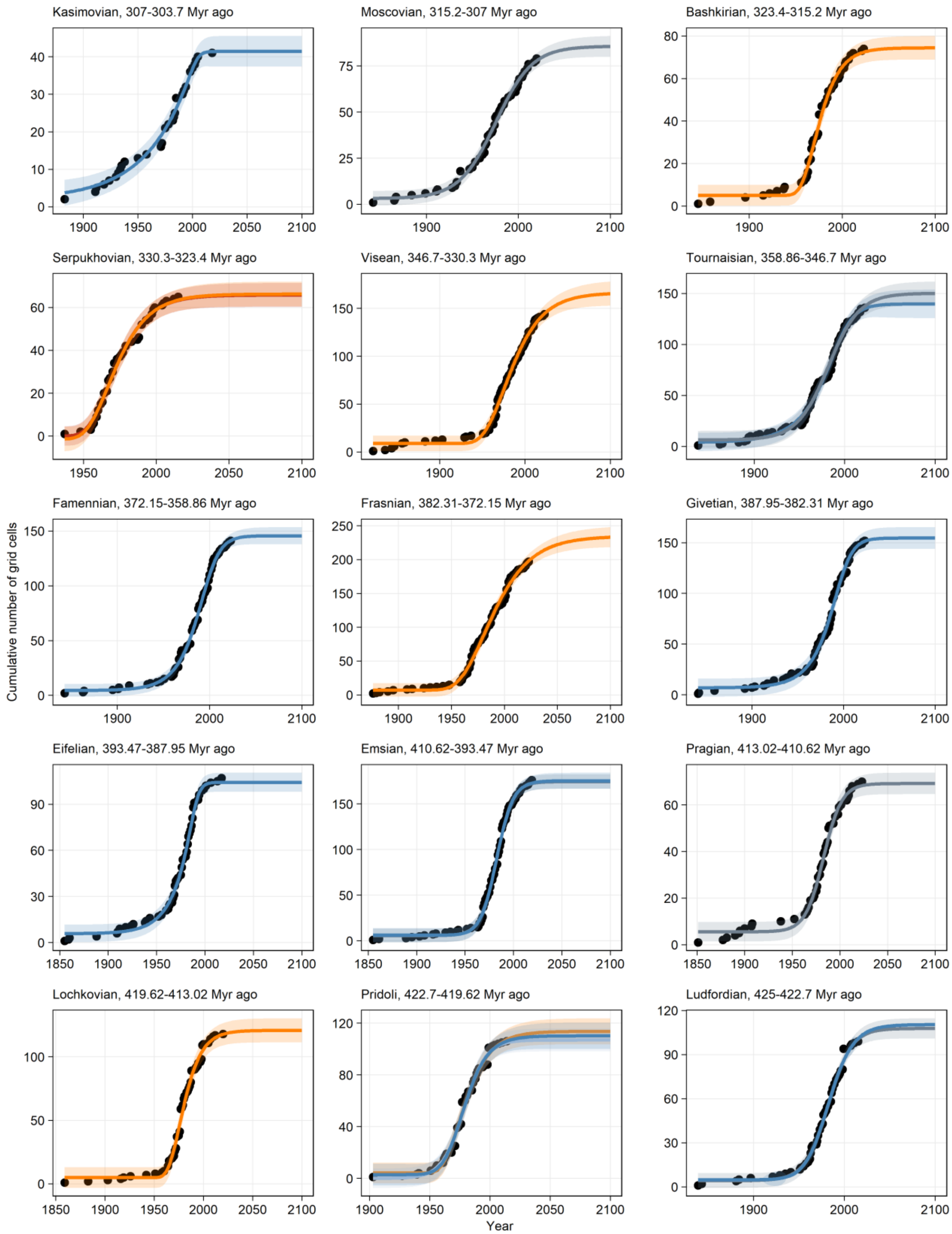

Fig. S7. Continued.

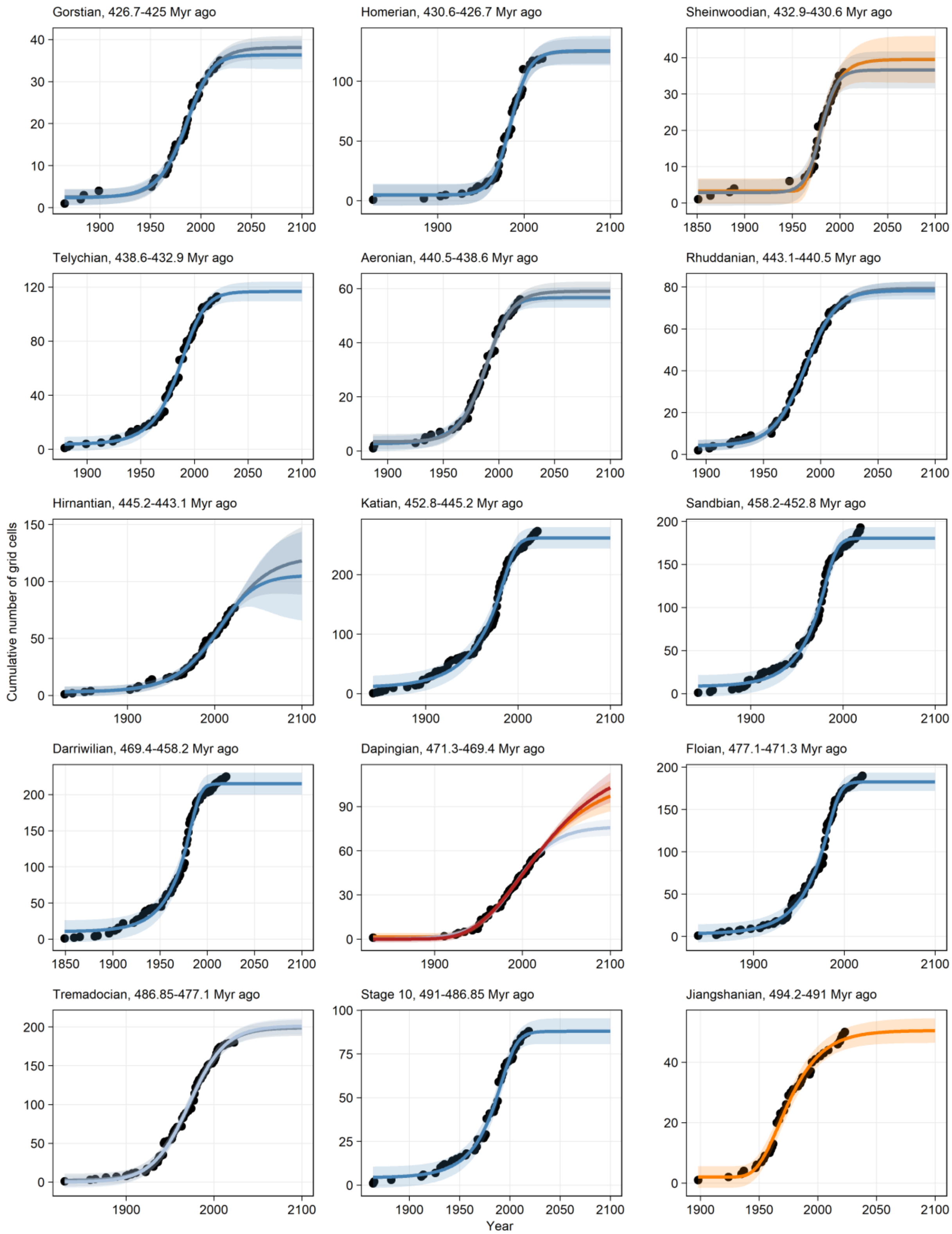

Fig. S7. Continued.

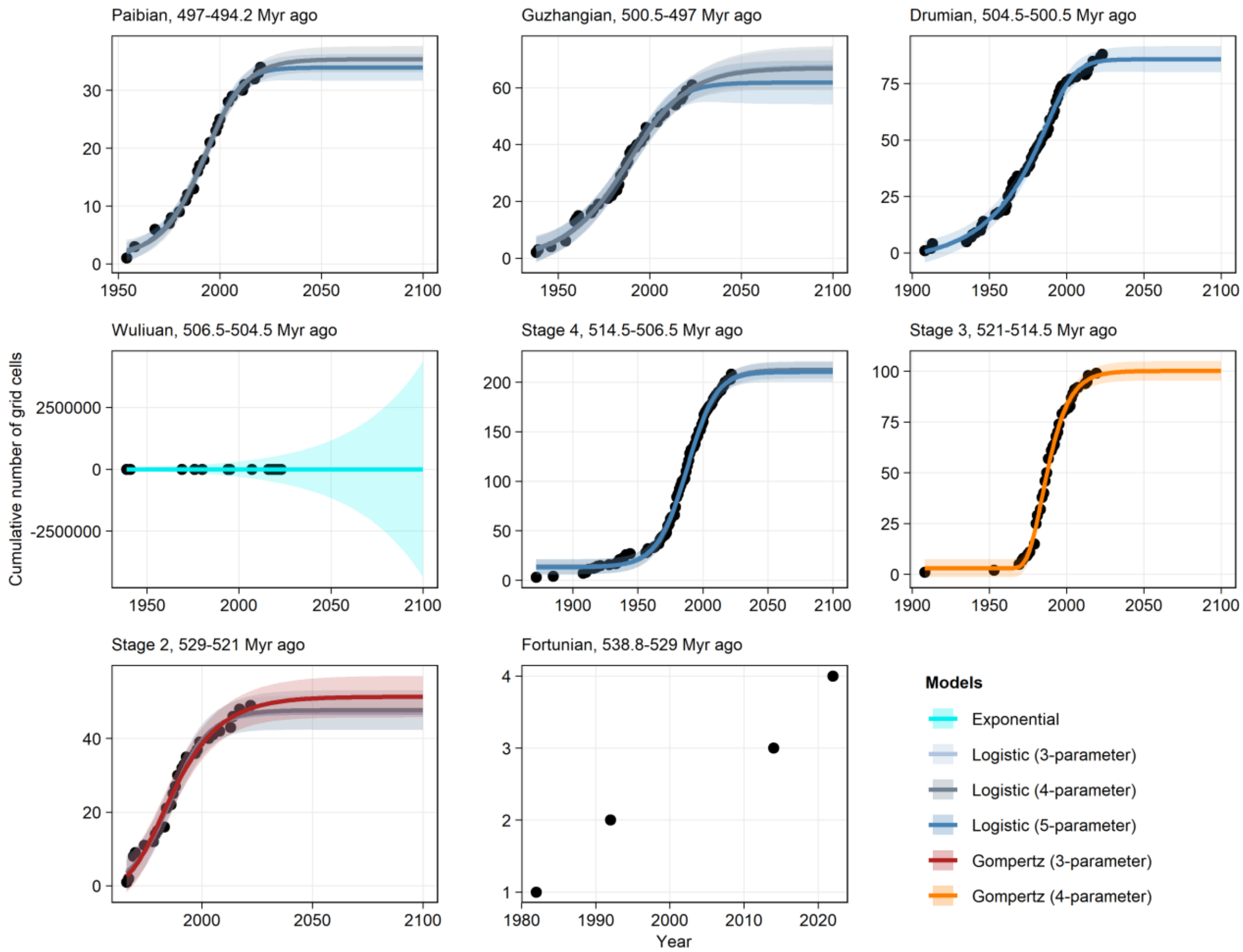

Fig. S7. Continued.

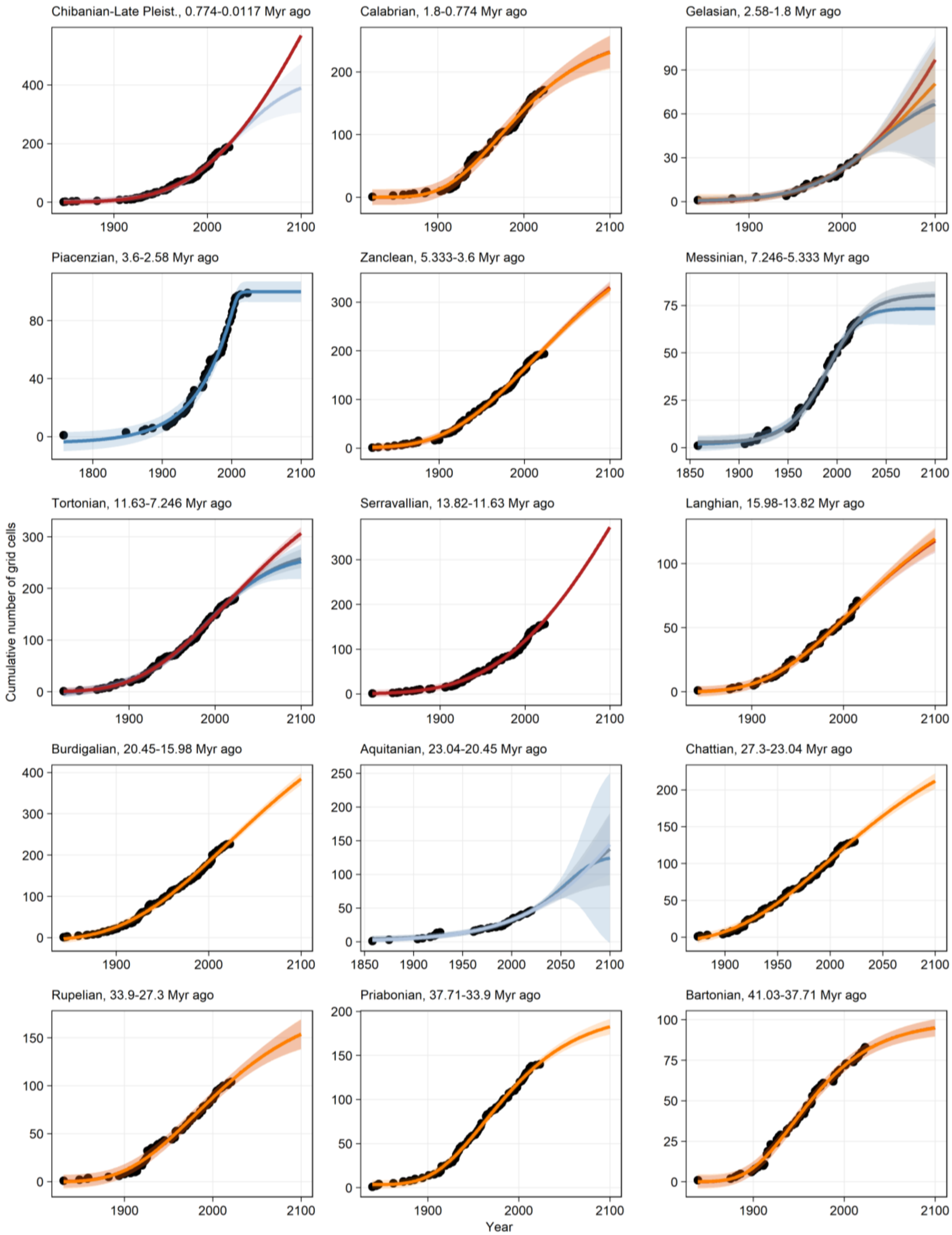

Fig. S8.

Growth models predicting future collection effort across grid cells until the year 2100 for each stage for the 250 km flat-topped grid. Note that for some stages, more than one model qualified as the best (i.e., within ΔAIC = 2). Underlying data is summarized in table S5.

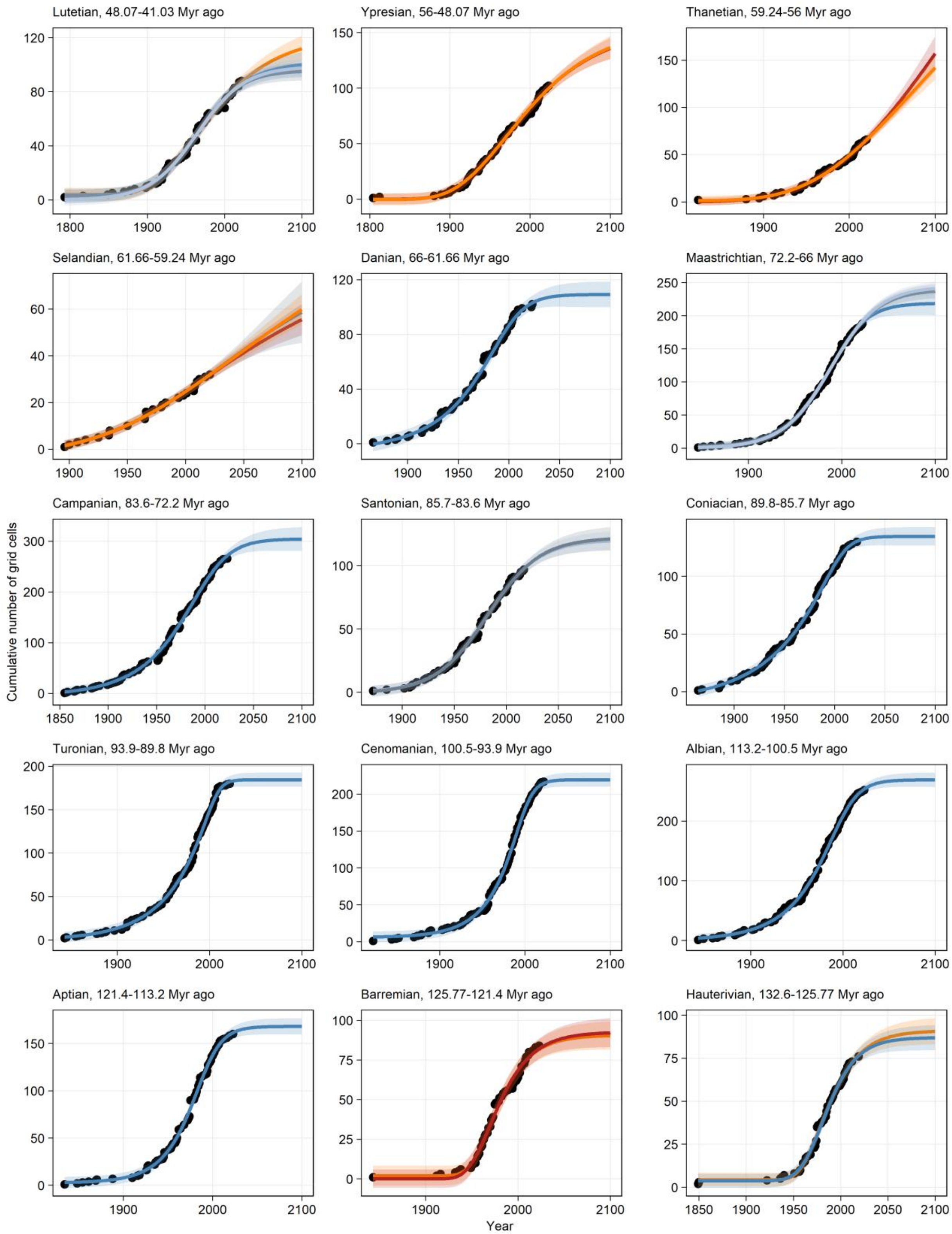

Fig. S8. Continued.

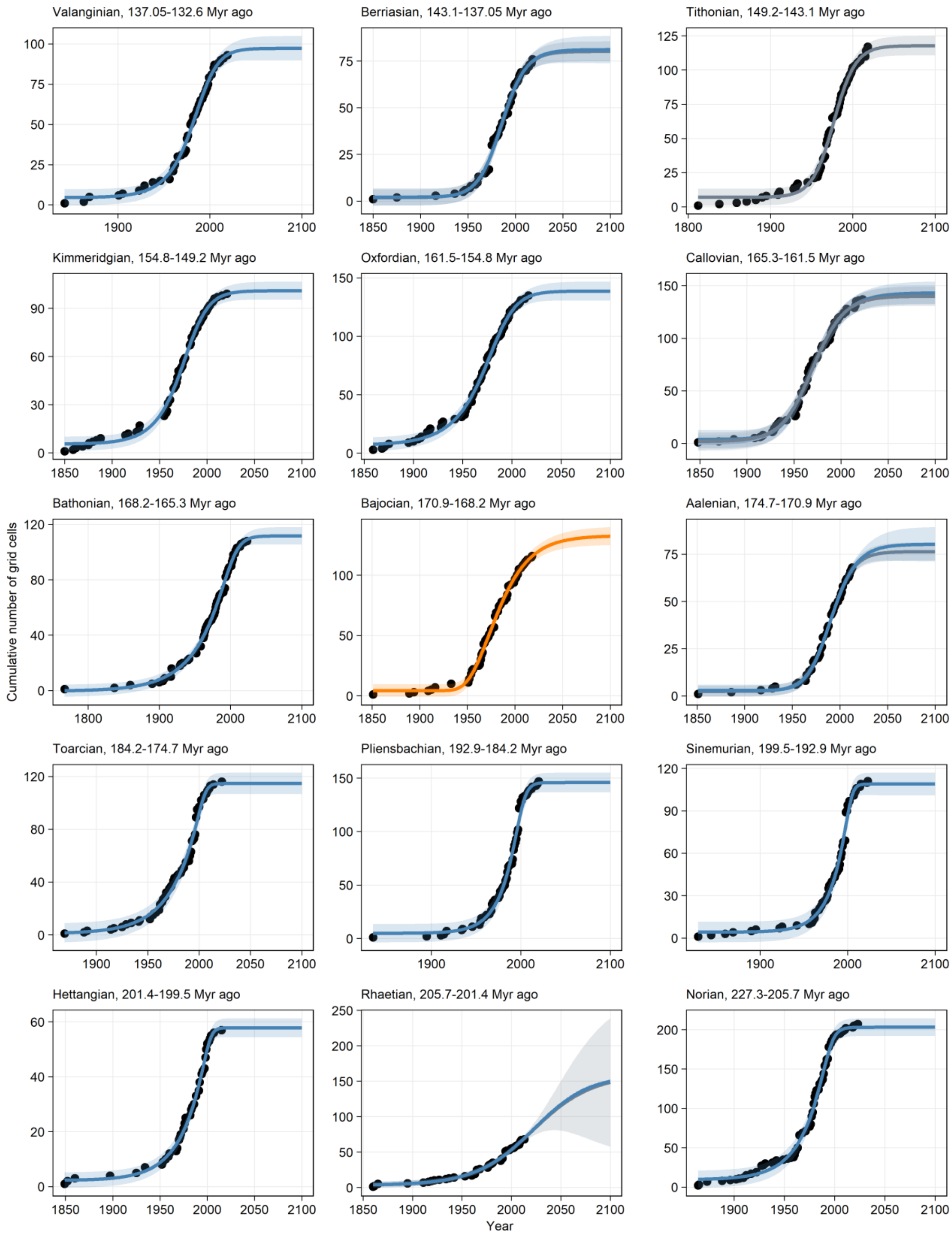

Fig. S8. Continued.

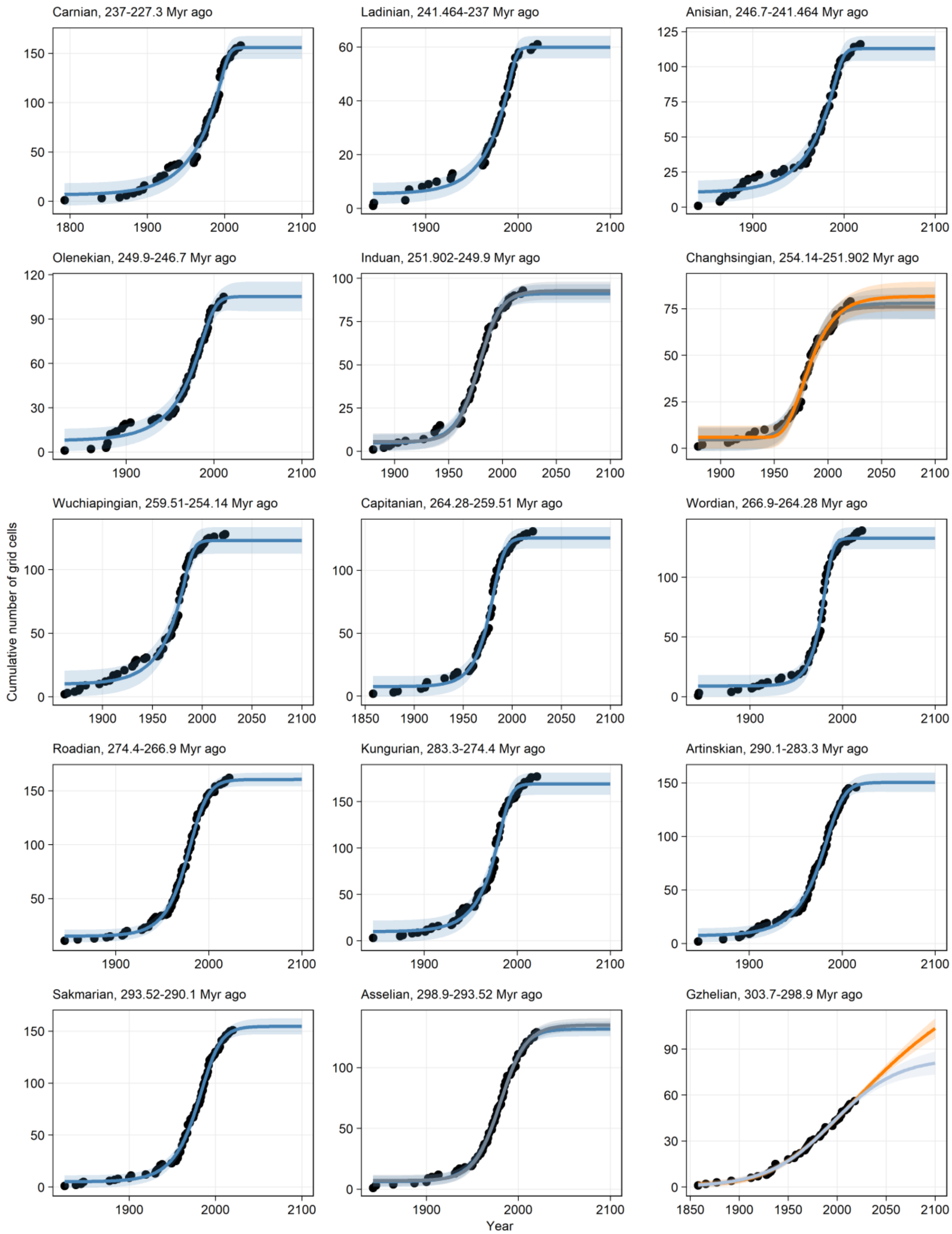

Fig. S8. Continued.

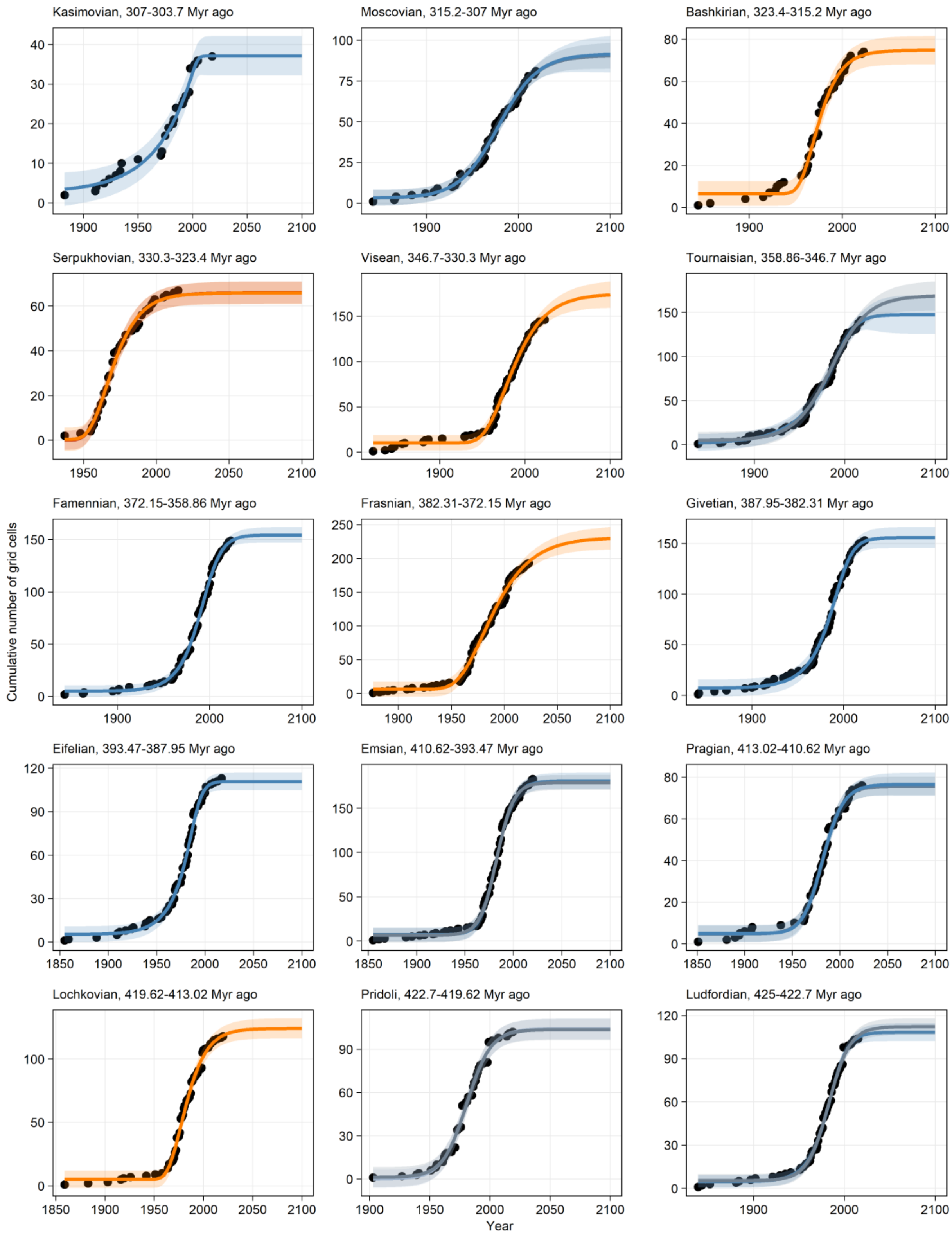

Fig. S8. Continued.

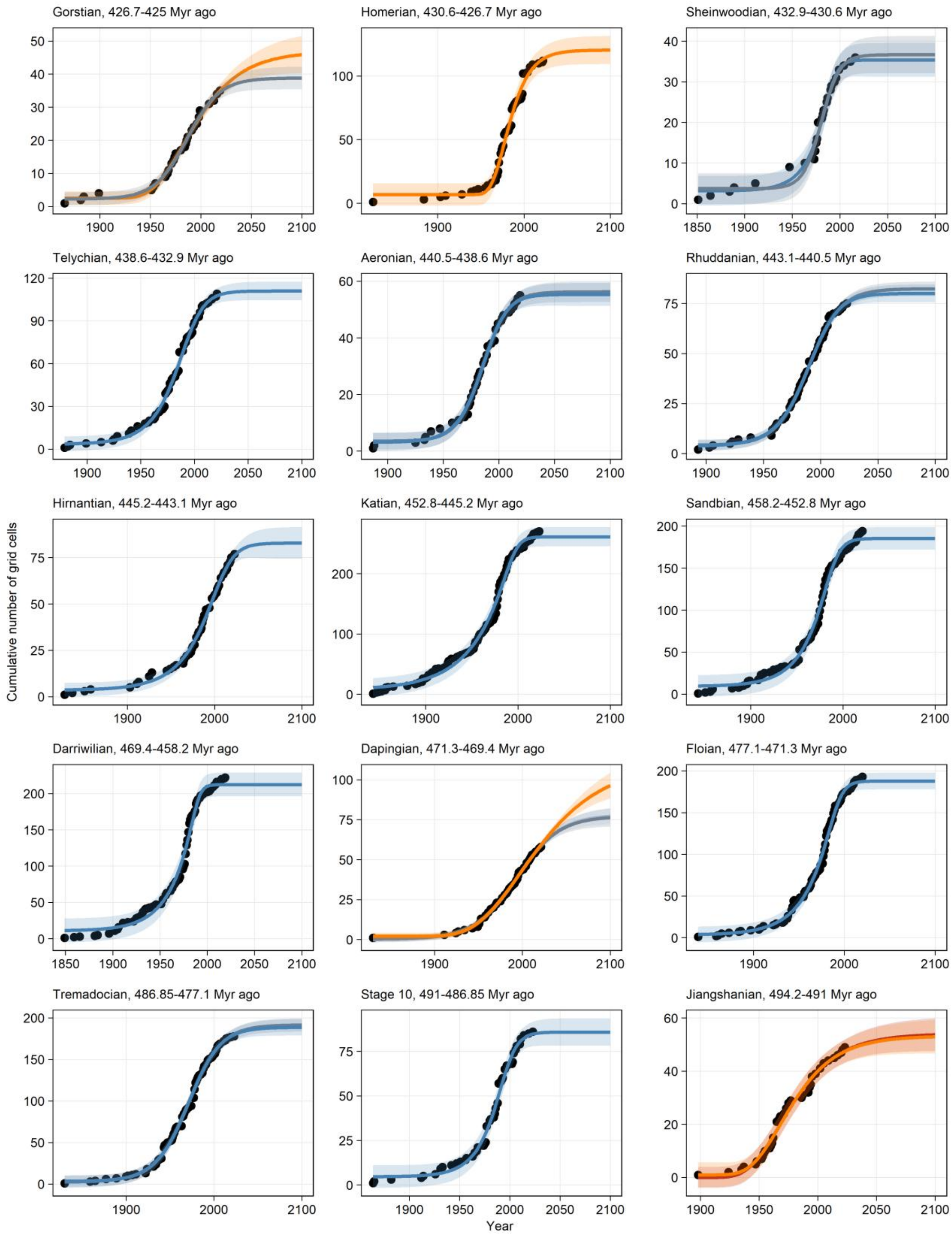

Fig. S8. Continued.

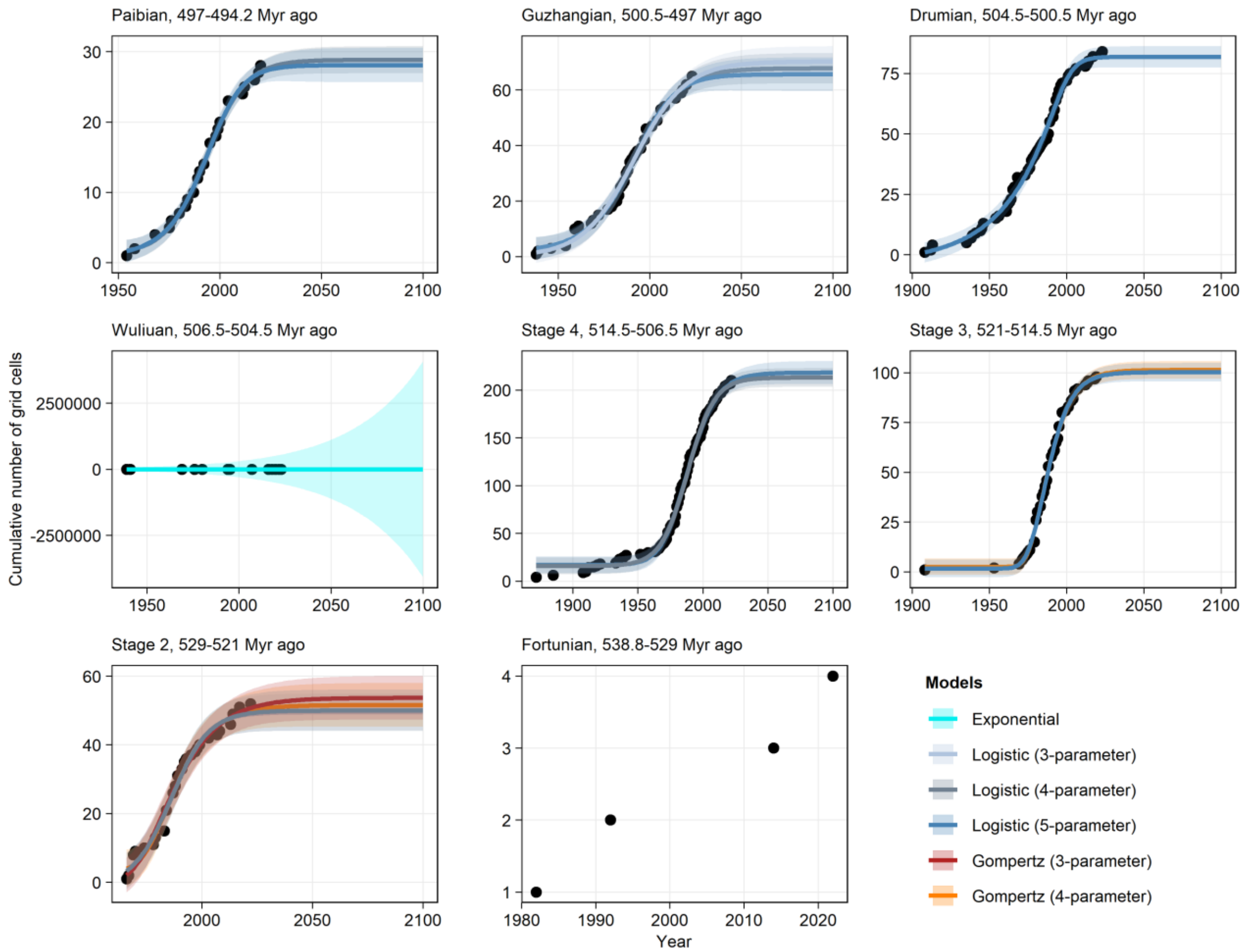

Fig. S8. Continued.

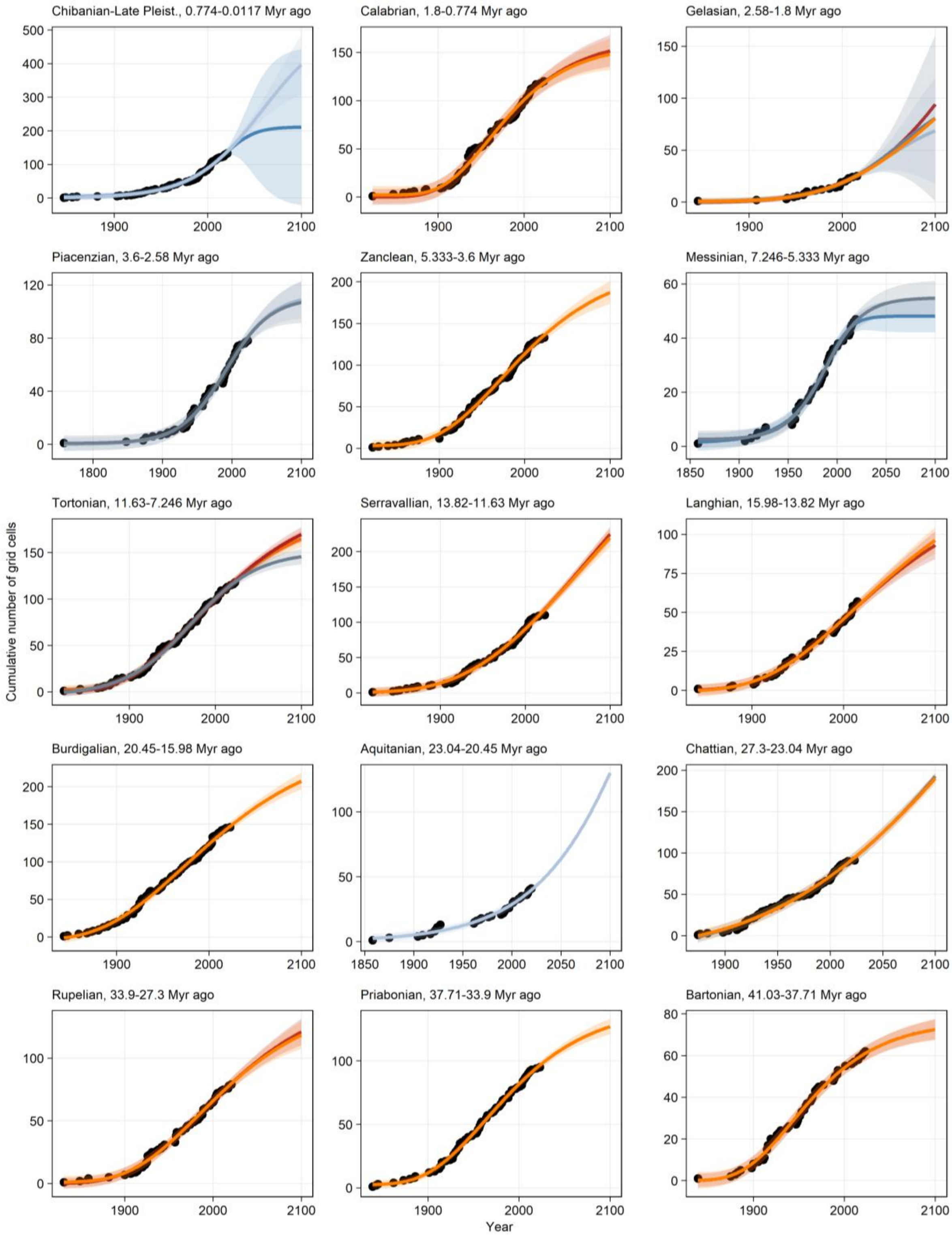

Fig. S9.

Growth models predicting future collection effort across grid cells until the year 2100 for each stage for the 500 km pointy-topped grid. Note that for some stages, more than one model qualified as the best (i.e., within ΔAIC = 2). Underlying data is summarized in table S5.

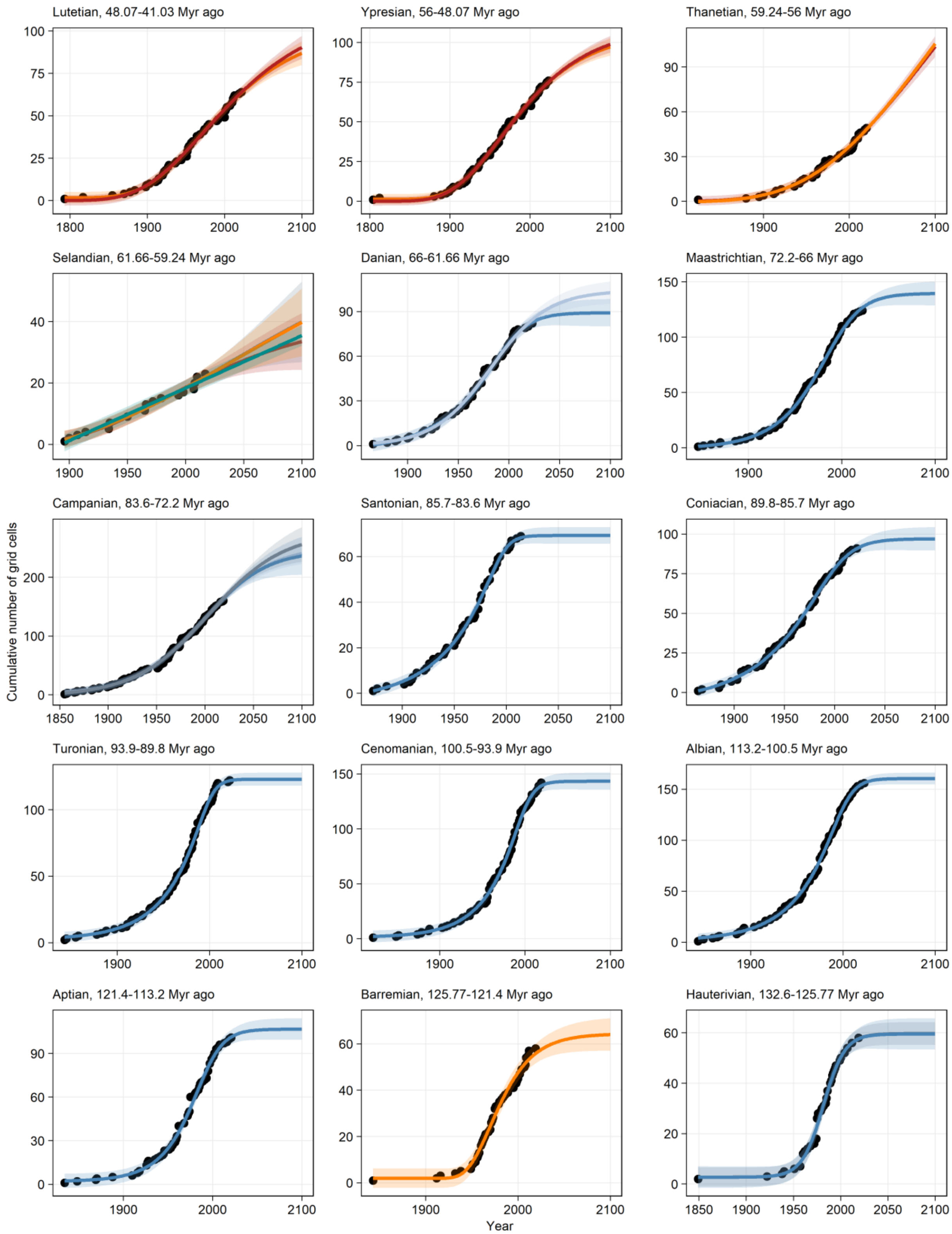

Fig. S9. Continued.

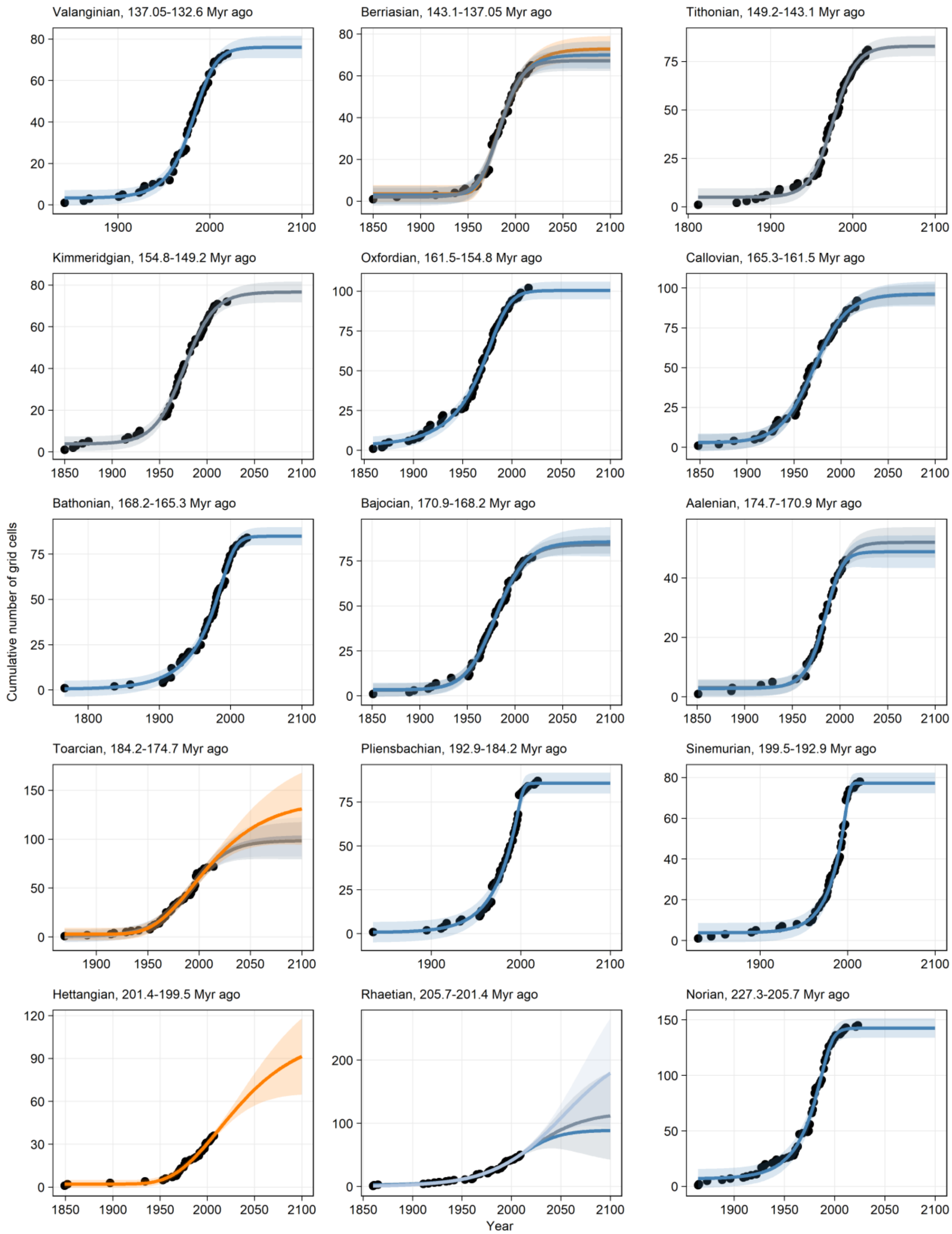

Fig. S9. Continued.

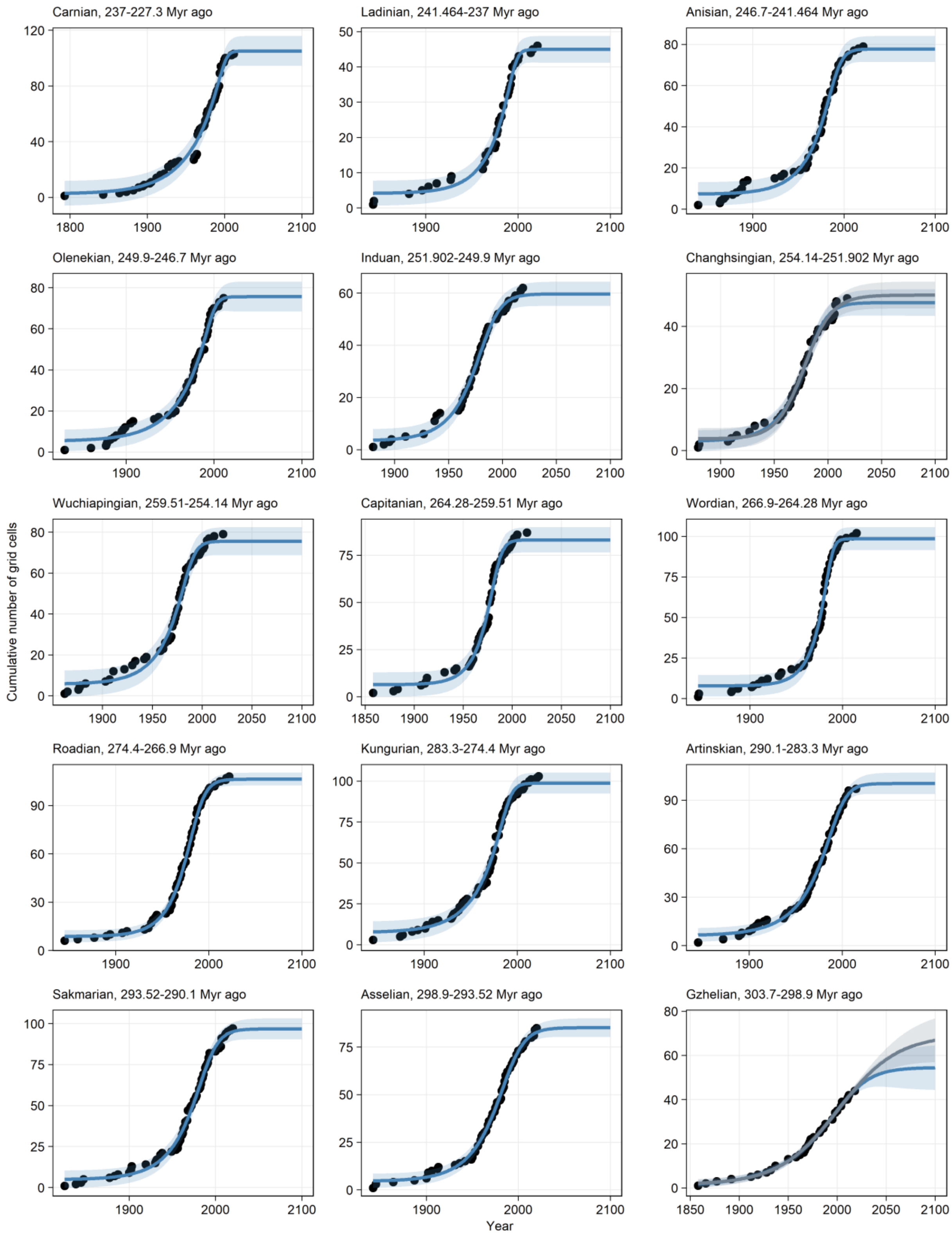

Fig. S9. Continued.

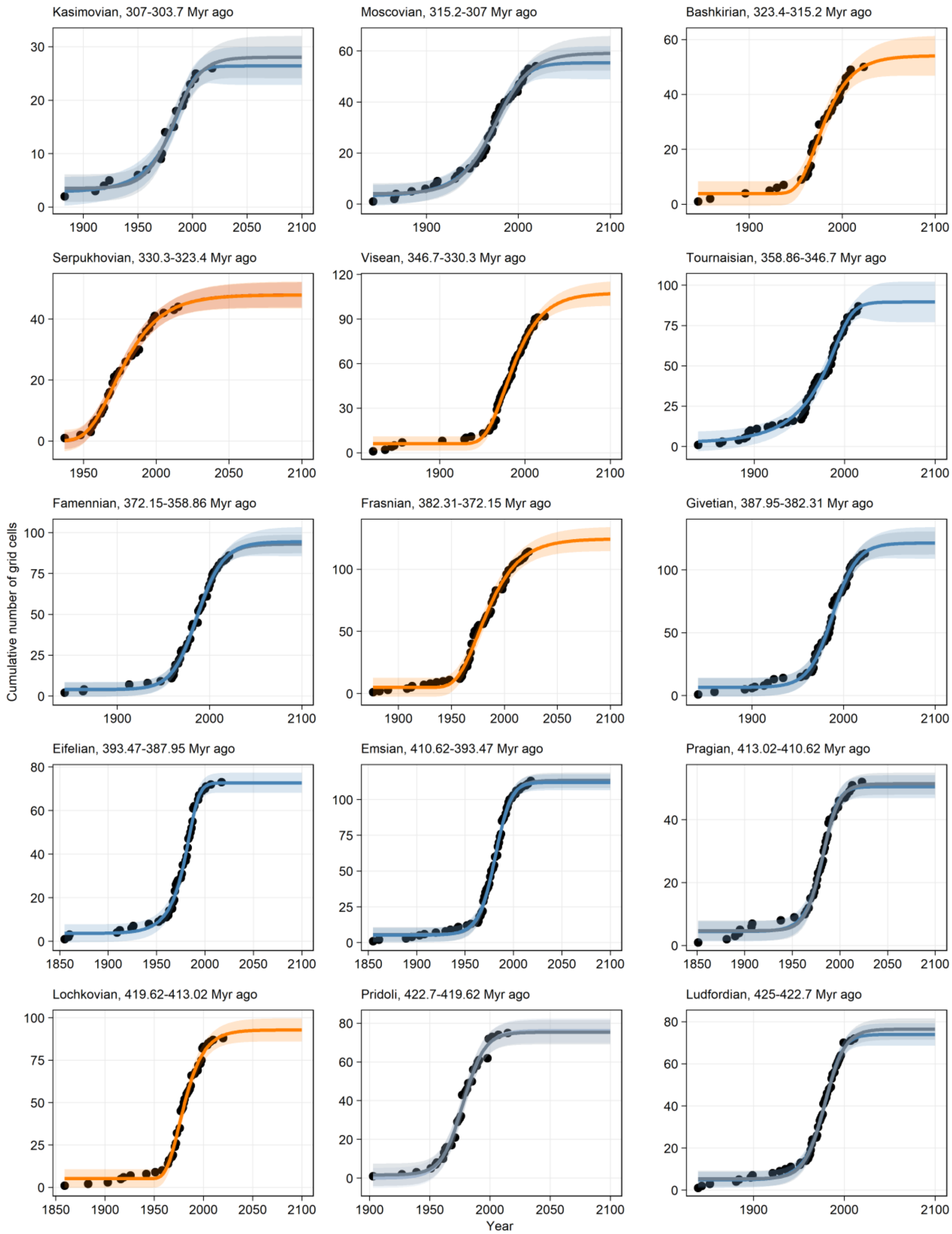

Fig. S9. Continued.

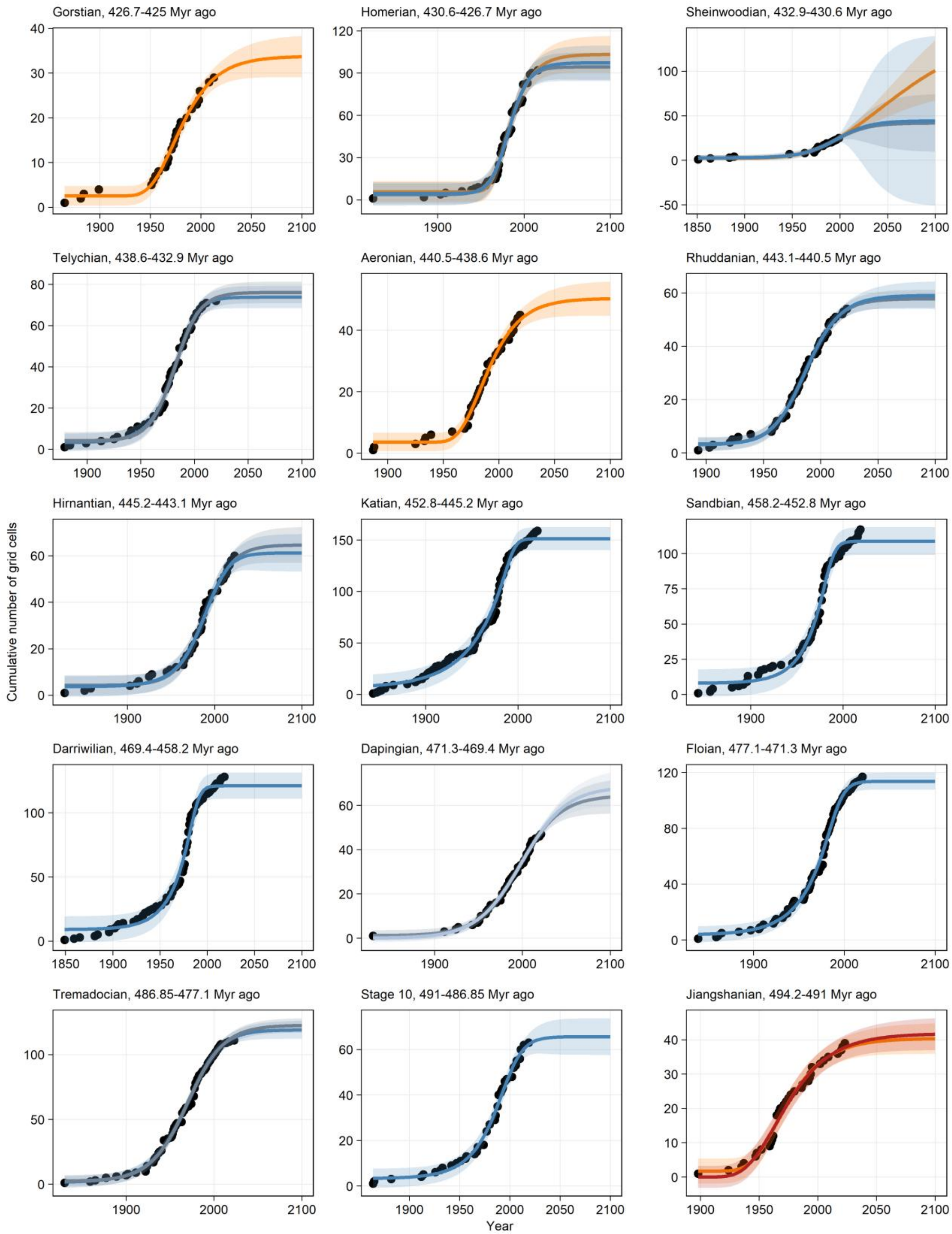

Fig. S9. Continued.

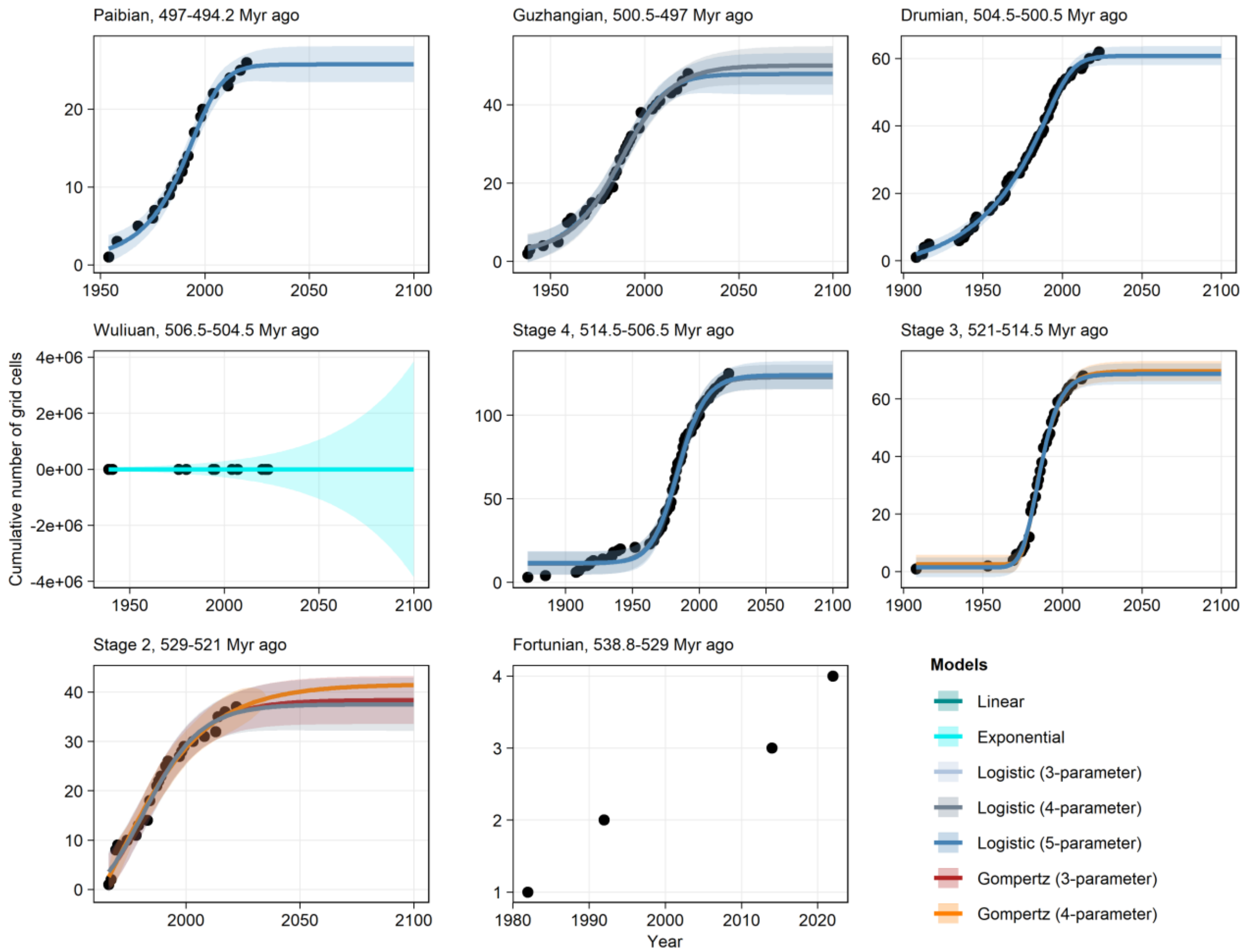

Fig. S9. Continued.

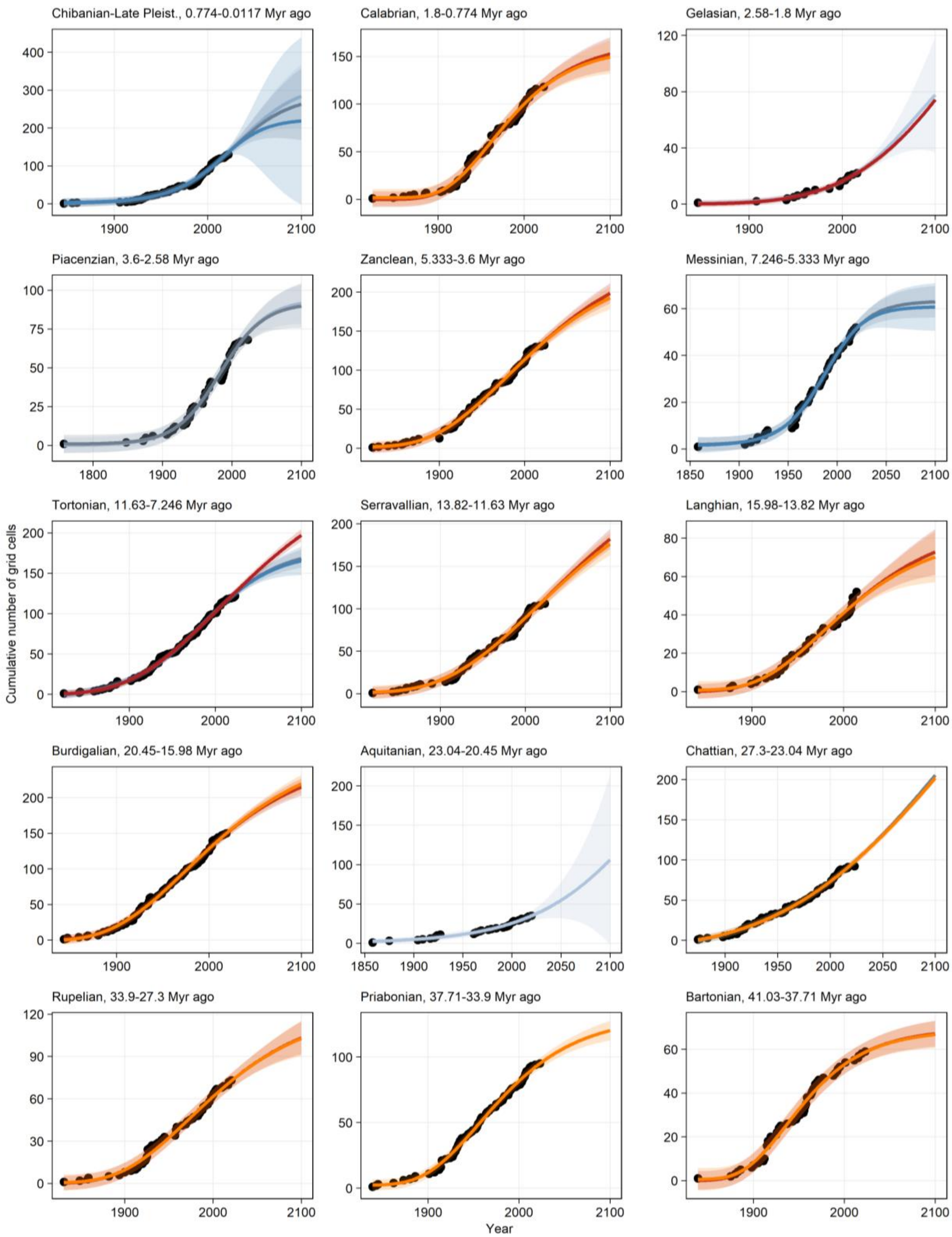

Fig. S10.

Growth models predicting future collection effort across grid cells until the year 2100 for each stage for the 500 km flat-topped grid. Note that for some stages, more than one model qualified as the best (i.e., within ΔAIC = 2). Underlying data is summarized in table S5.

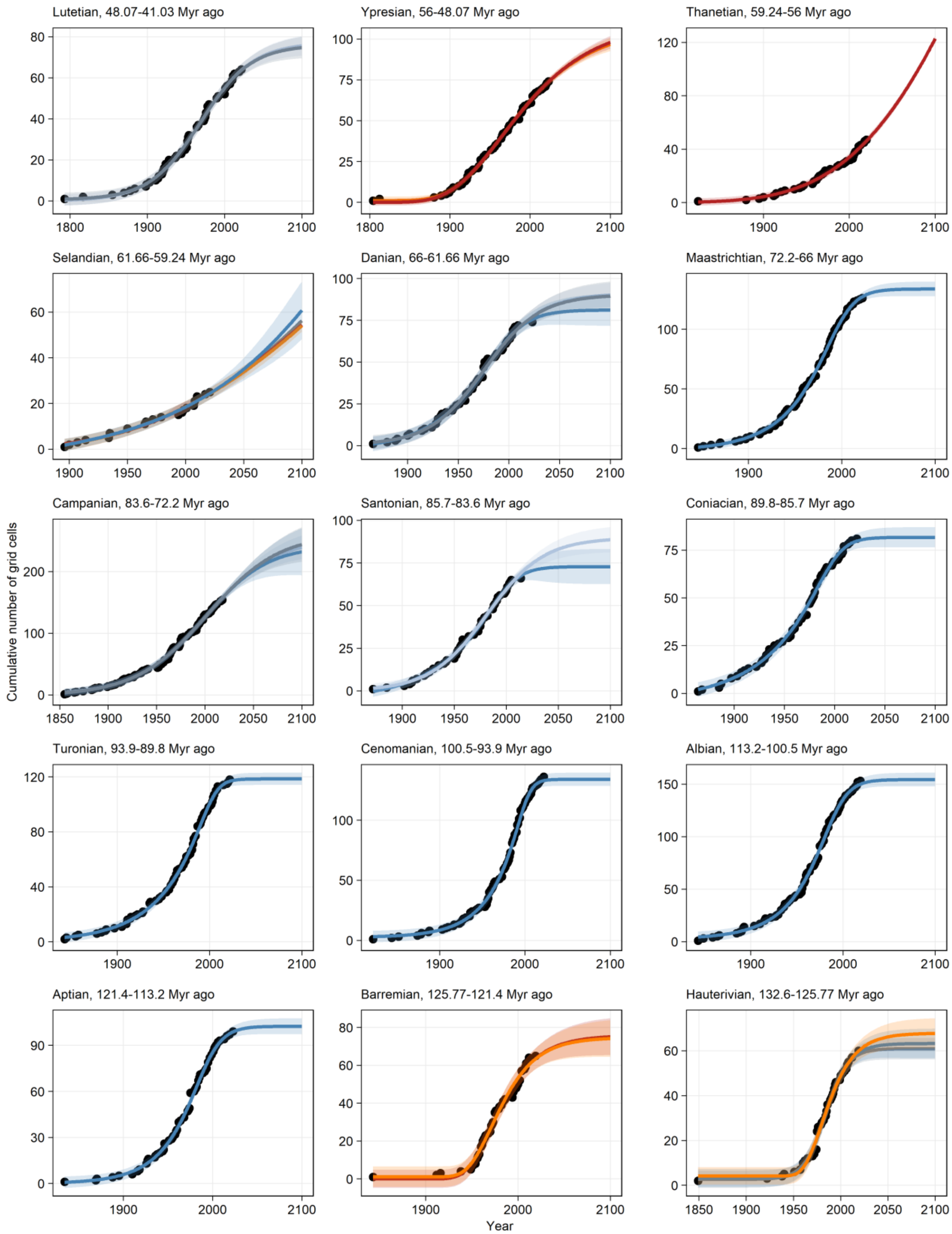

Fig. S10. Continued.

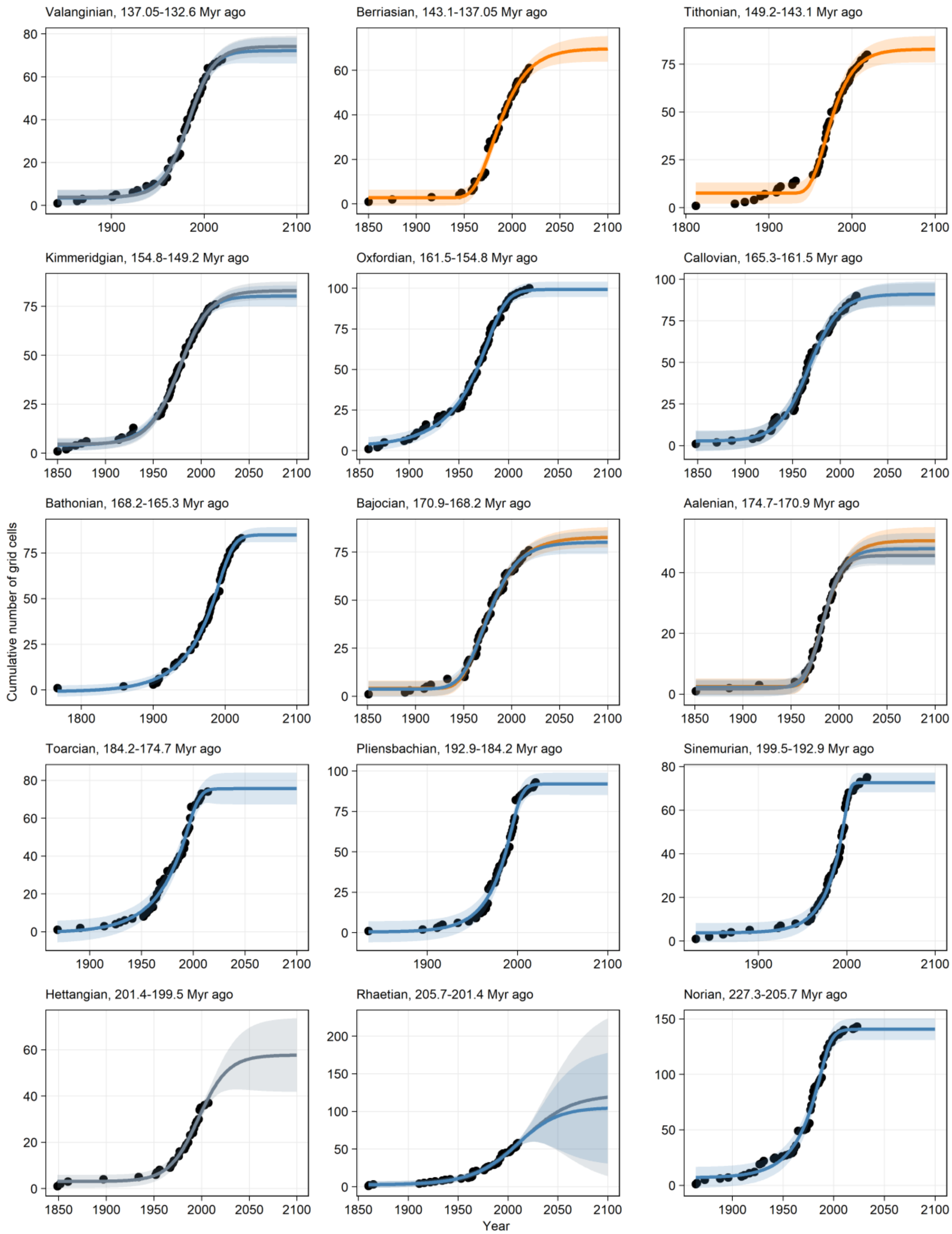

Fig. S10. Continued.

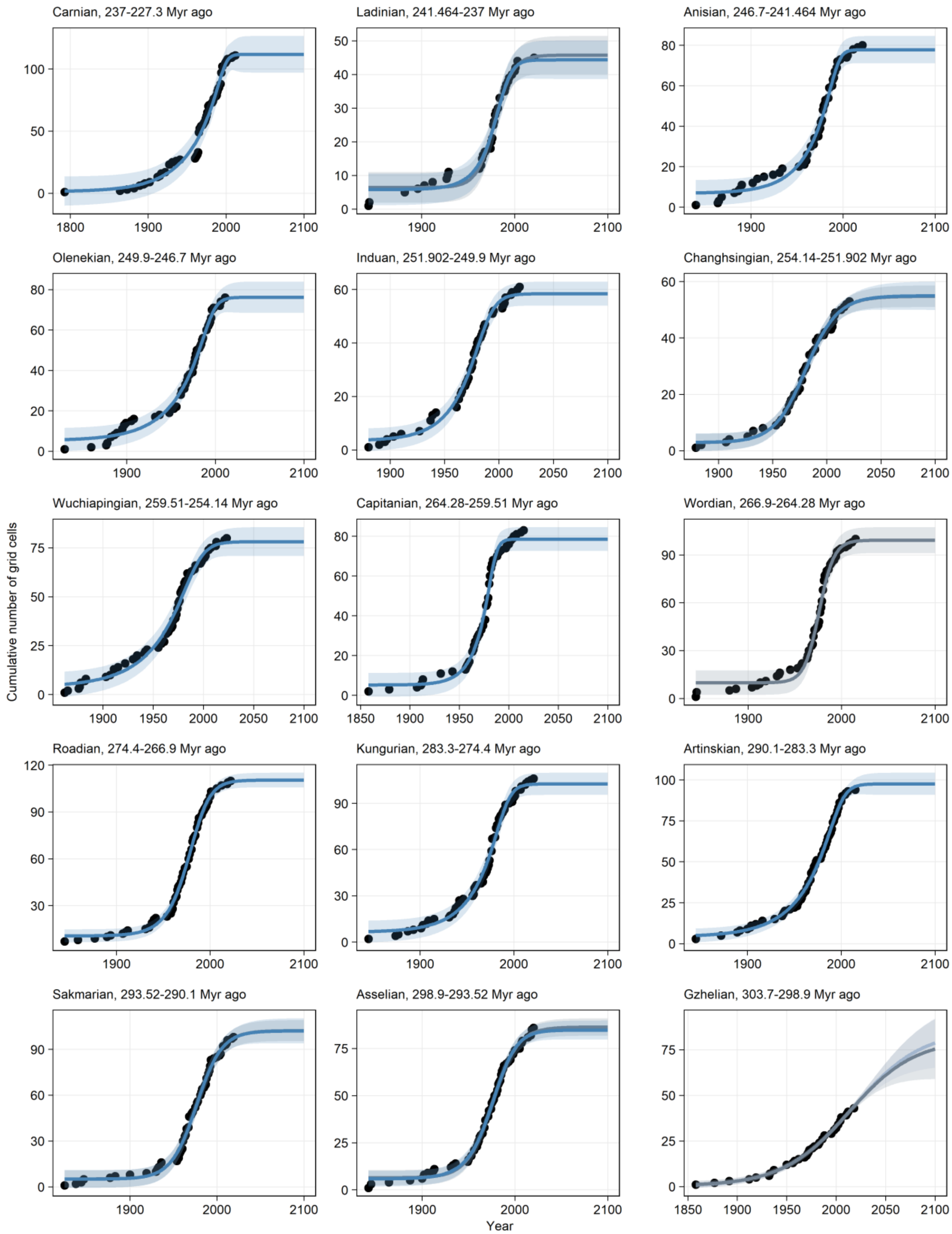

Fig. S10. Continued.

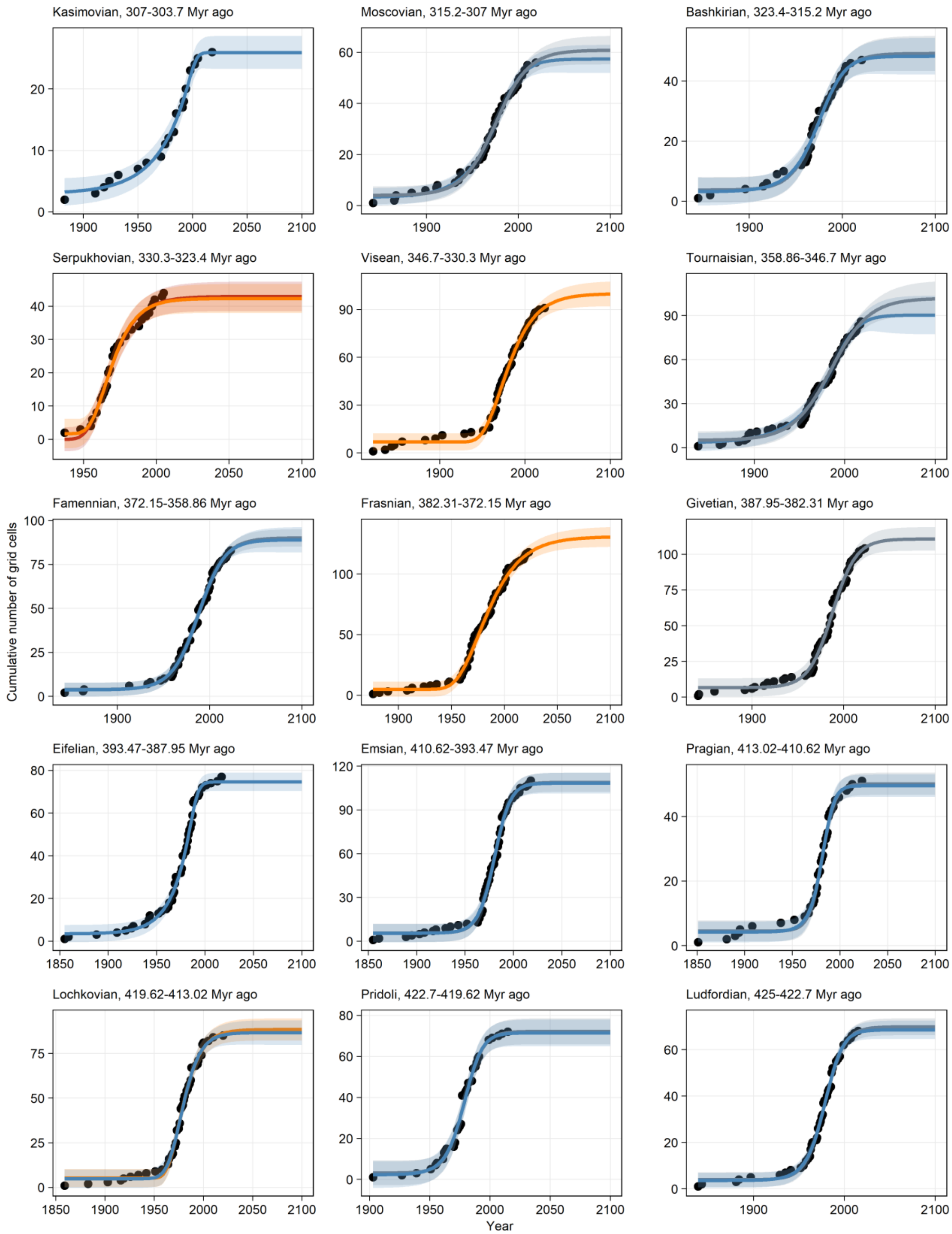

Fig. S10. Continued.

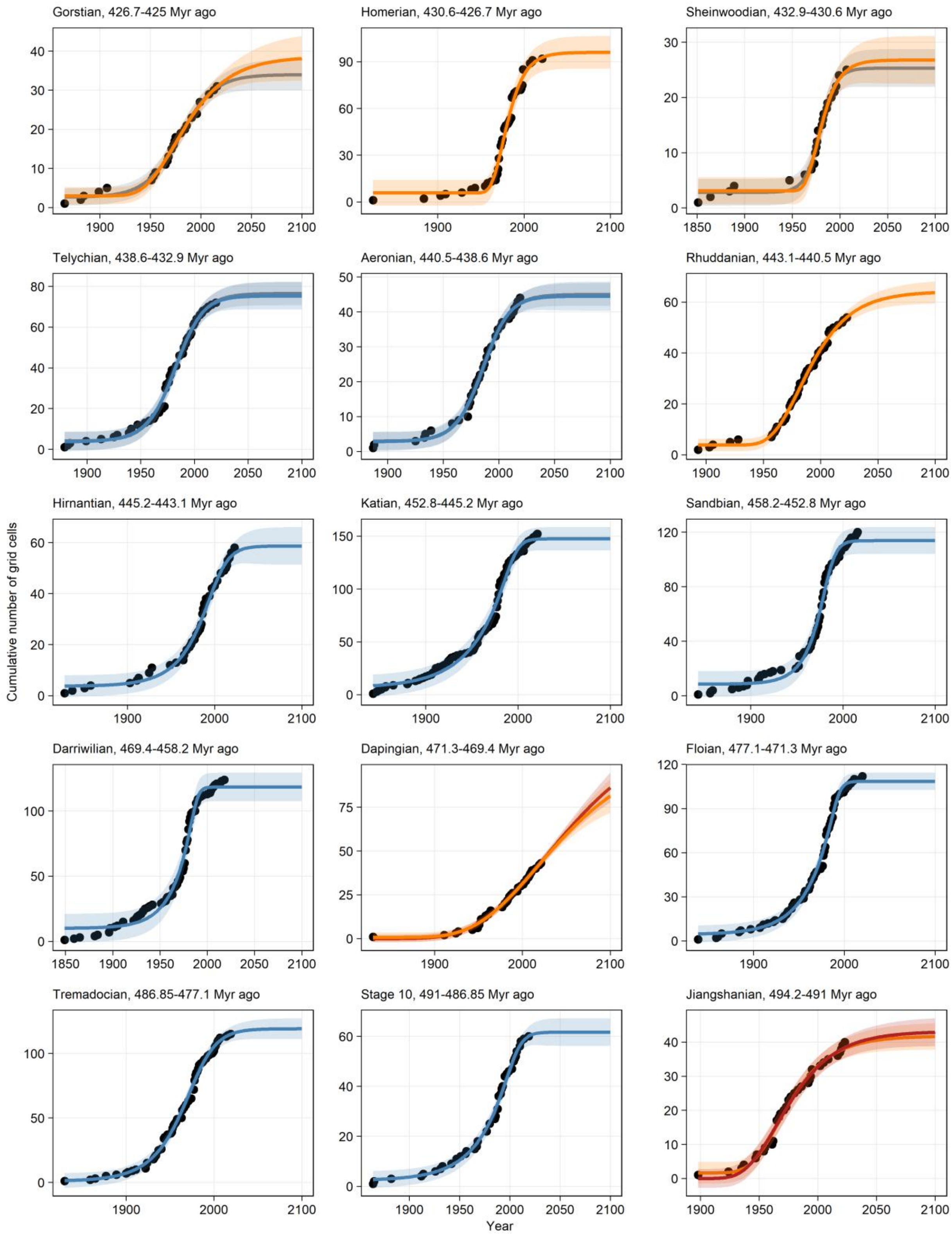

Fig. S10. Continued.

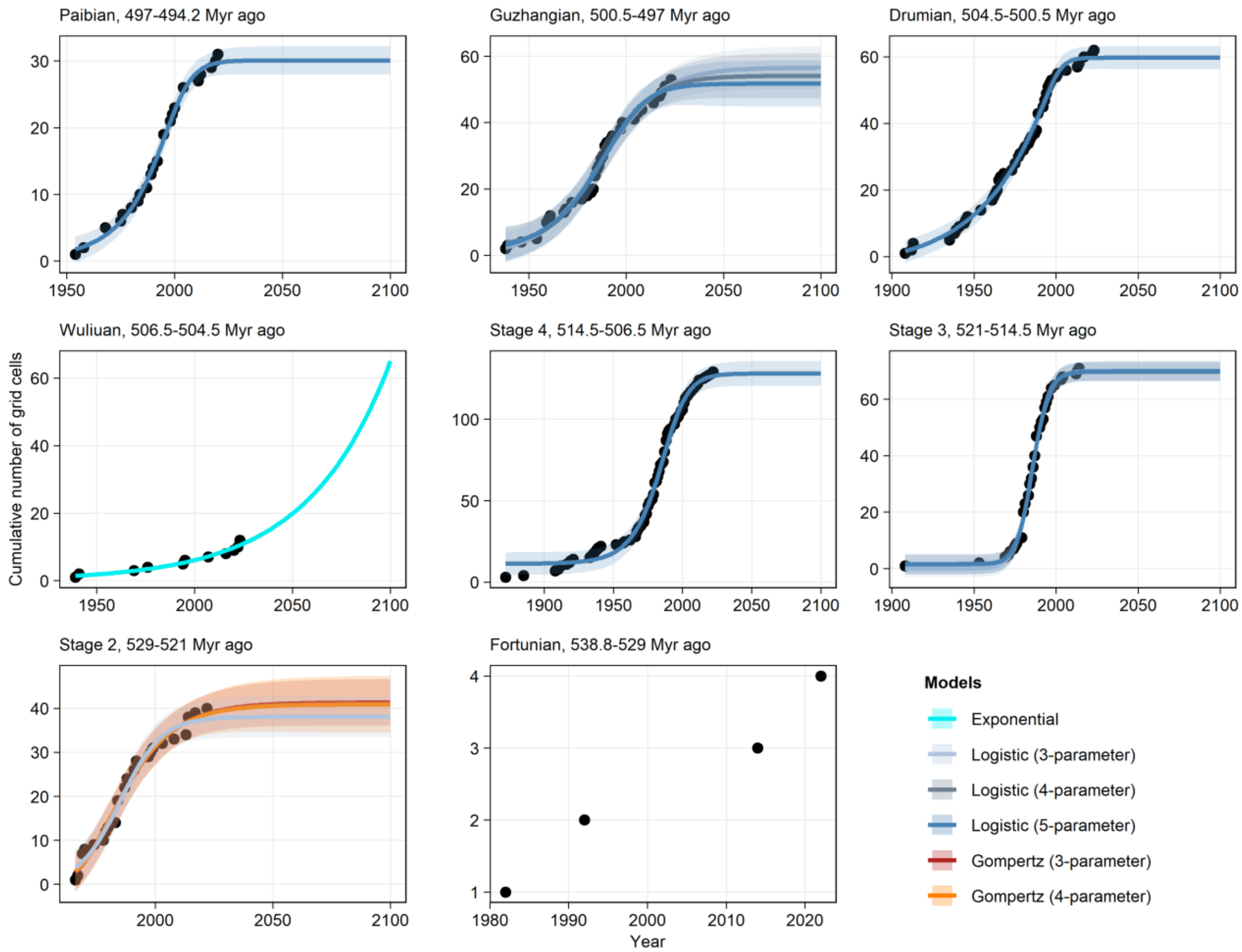

Fig. S10. Continued.

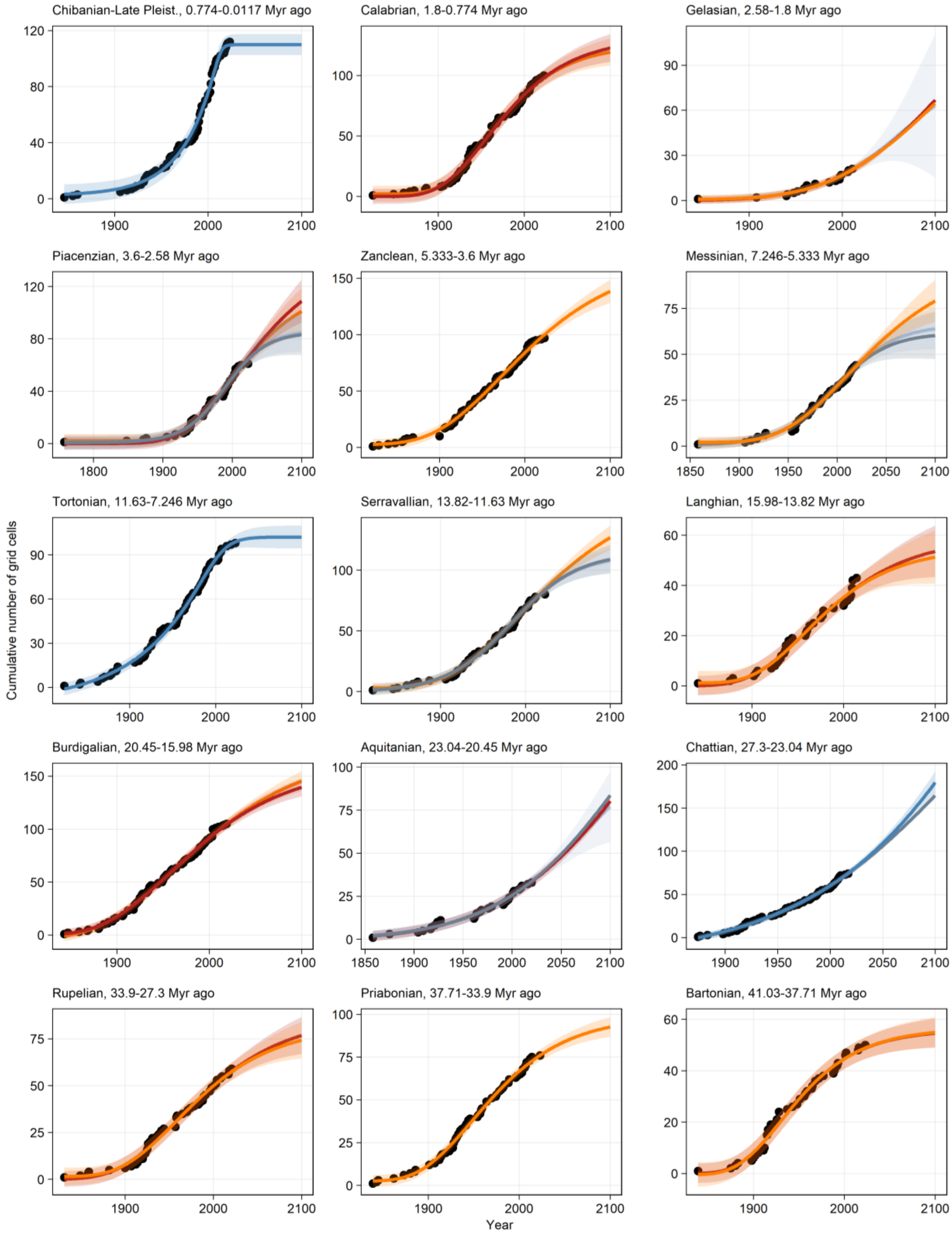

Fig. S11.

Growth models predicting future collection effort across grid cells until the year 2100 for each stage for the 750 km pointy-topped grid. Note that for some stages, more than one model qualified as the best (i.e., within ΔAIC = 2). Underlying data is summarized in table S5.

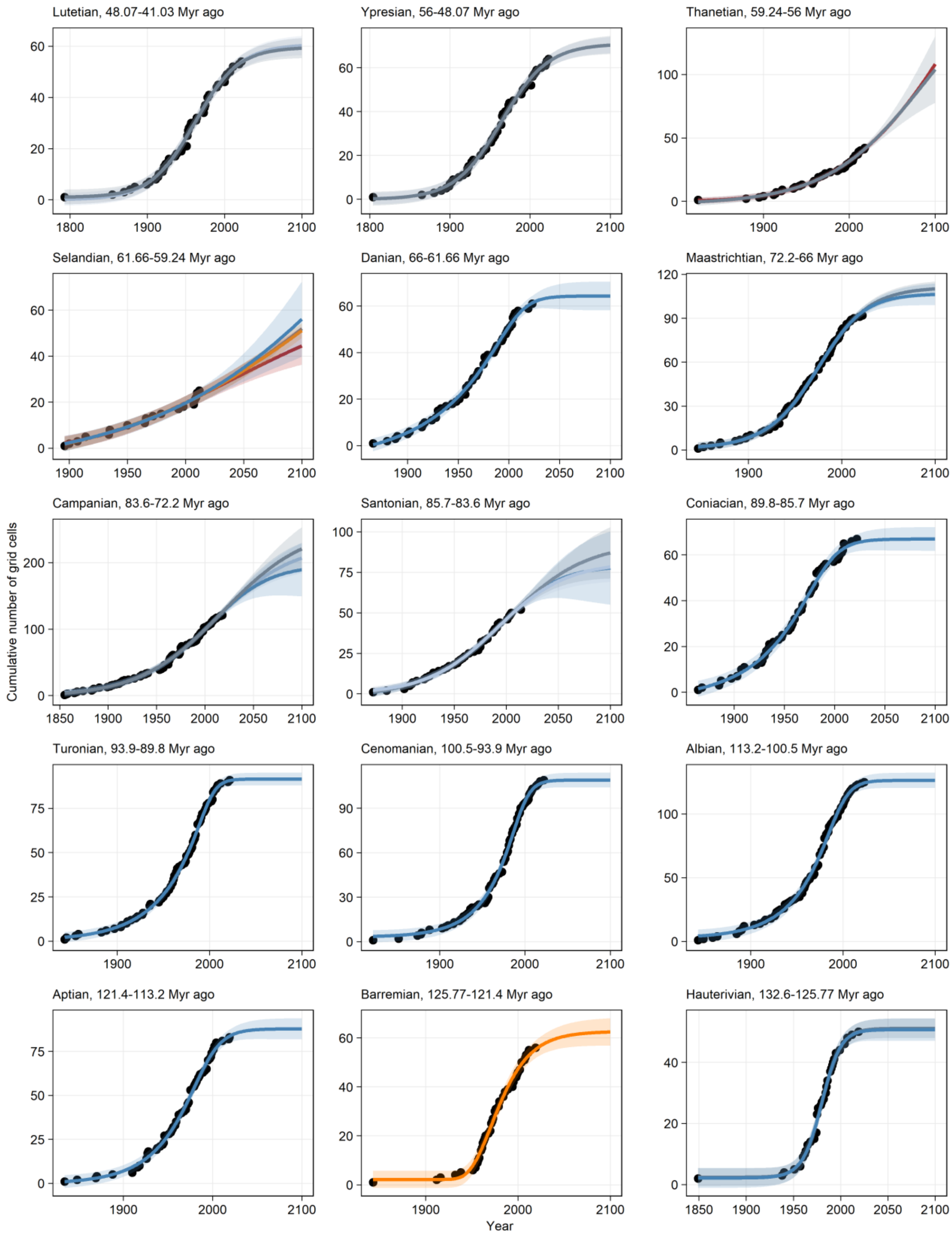

Fig. S11. Continued.

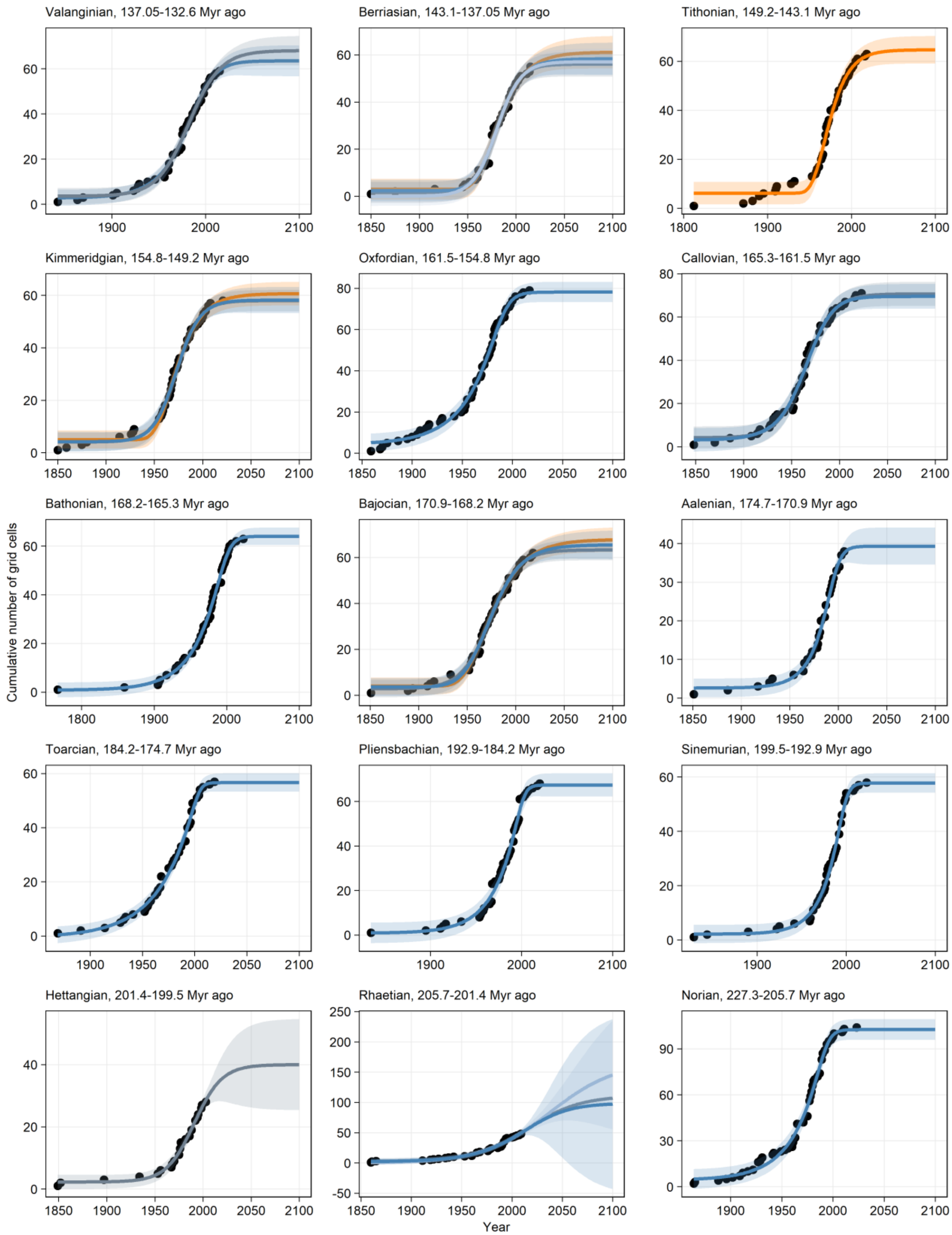

Fig. S11. Continued.

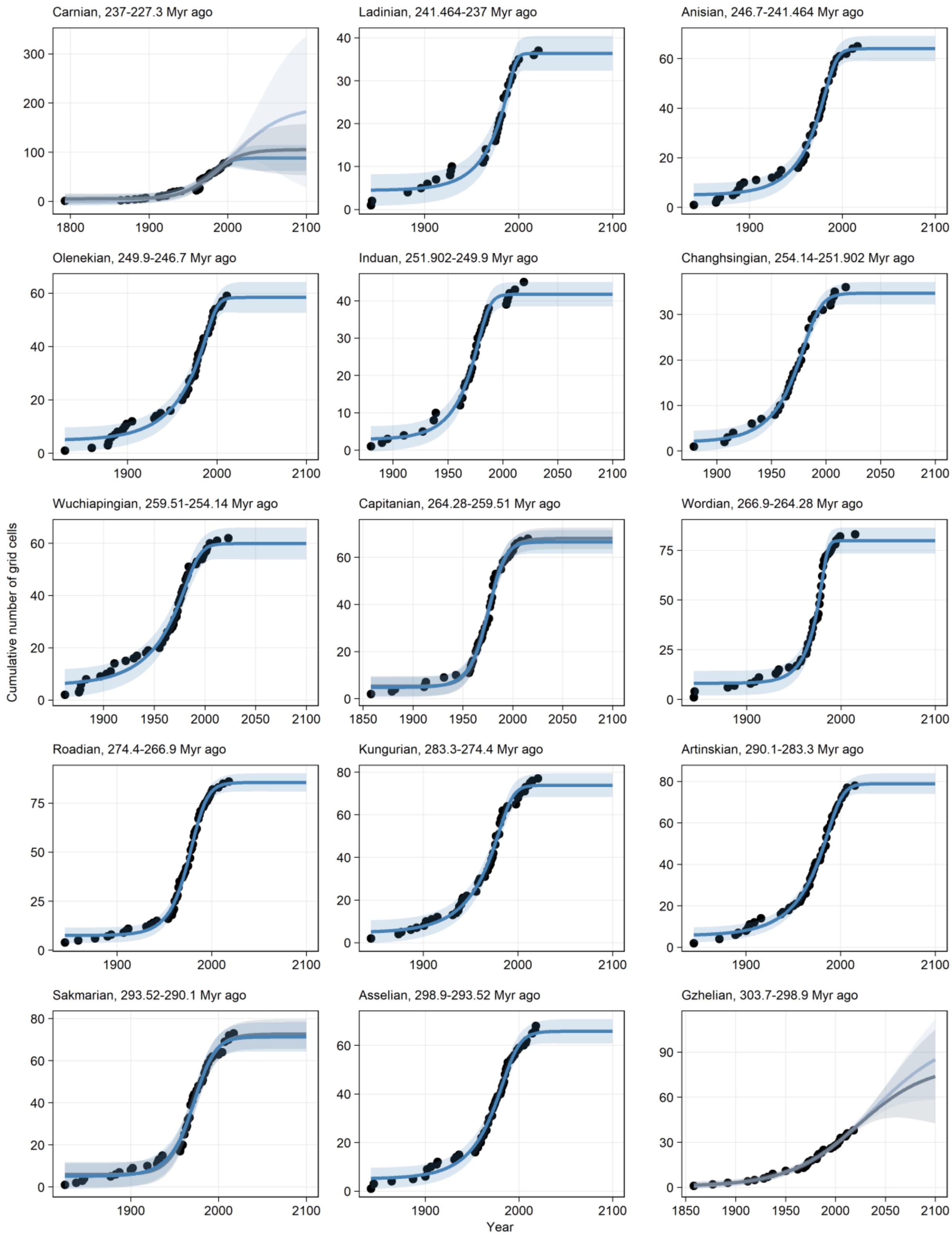

Fig. S11. Continued.

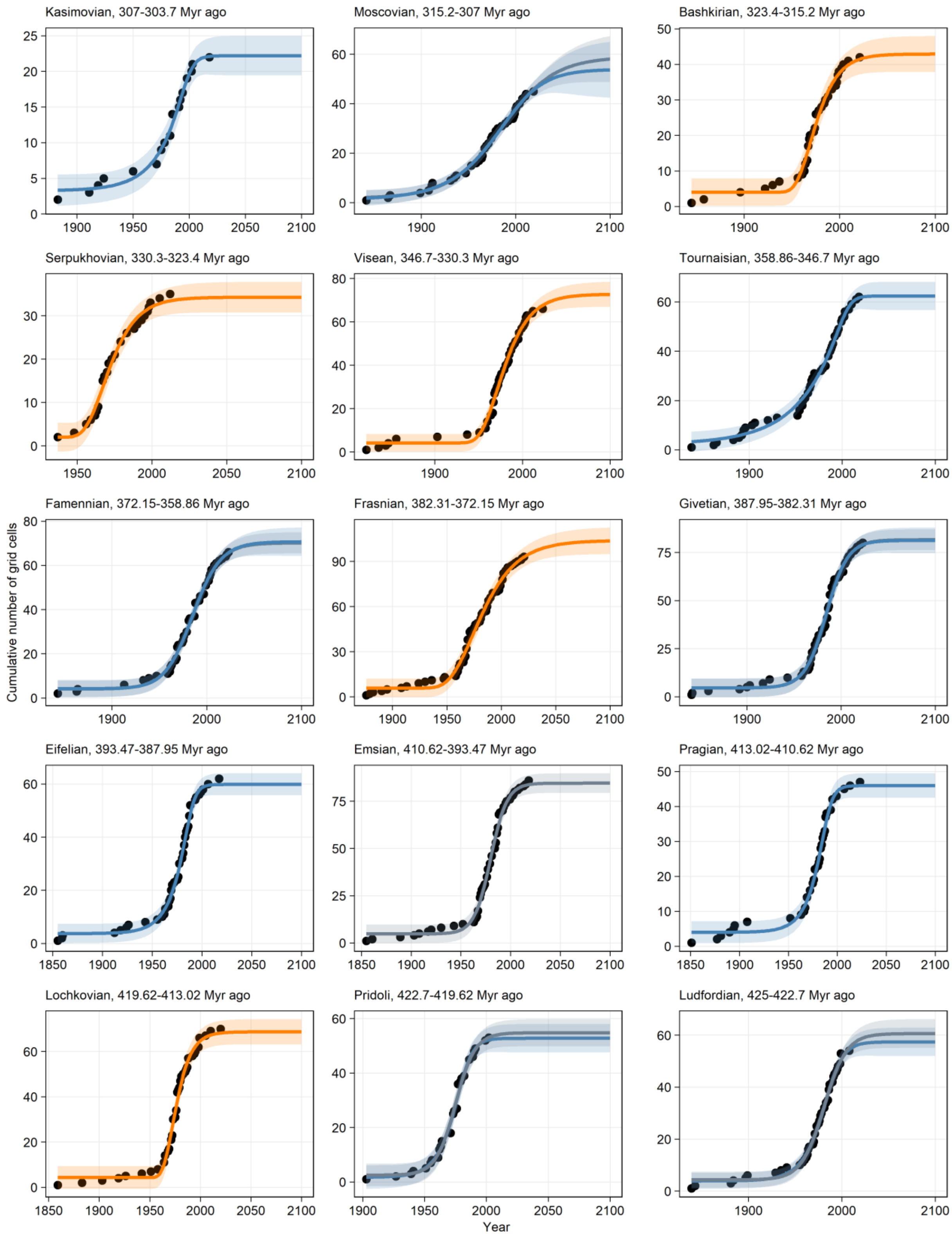

Fig. S11. Continued.

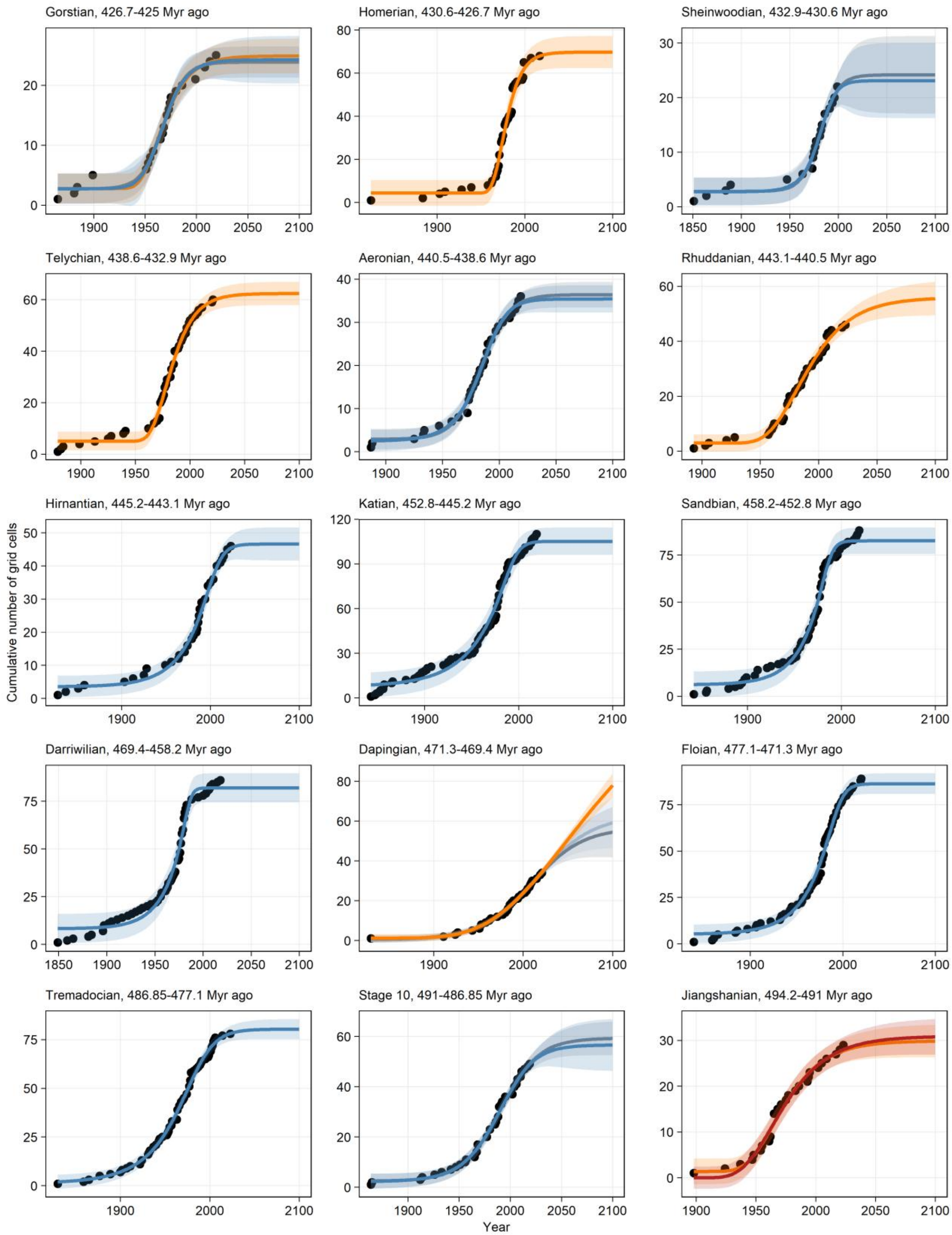

Fig. S11. Continued.

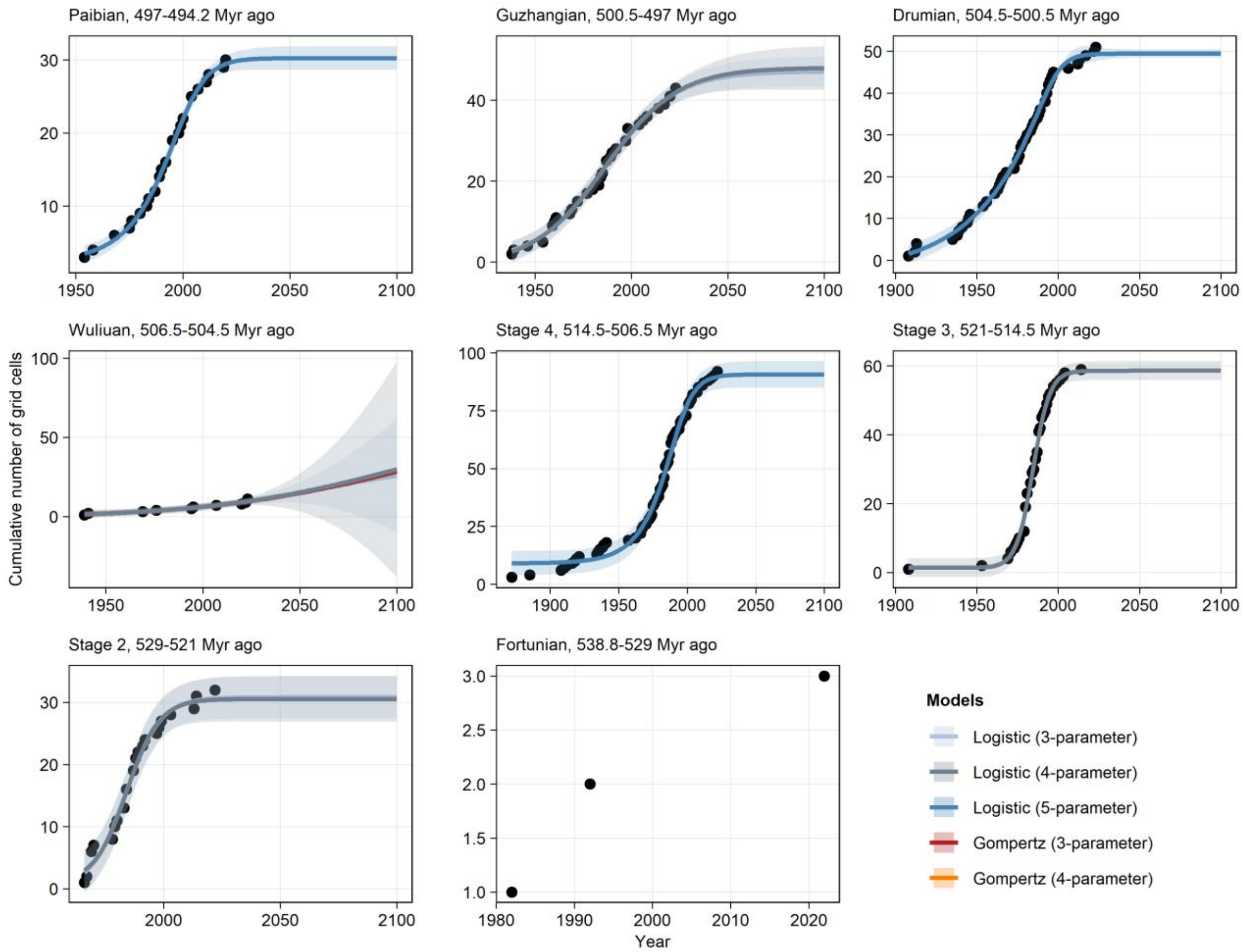

Fig. S11. Continued.

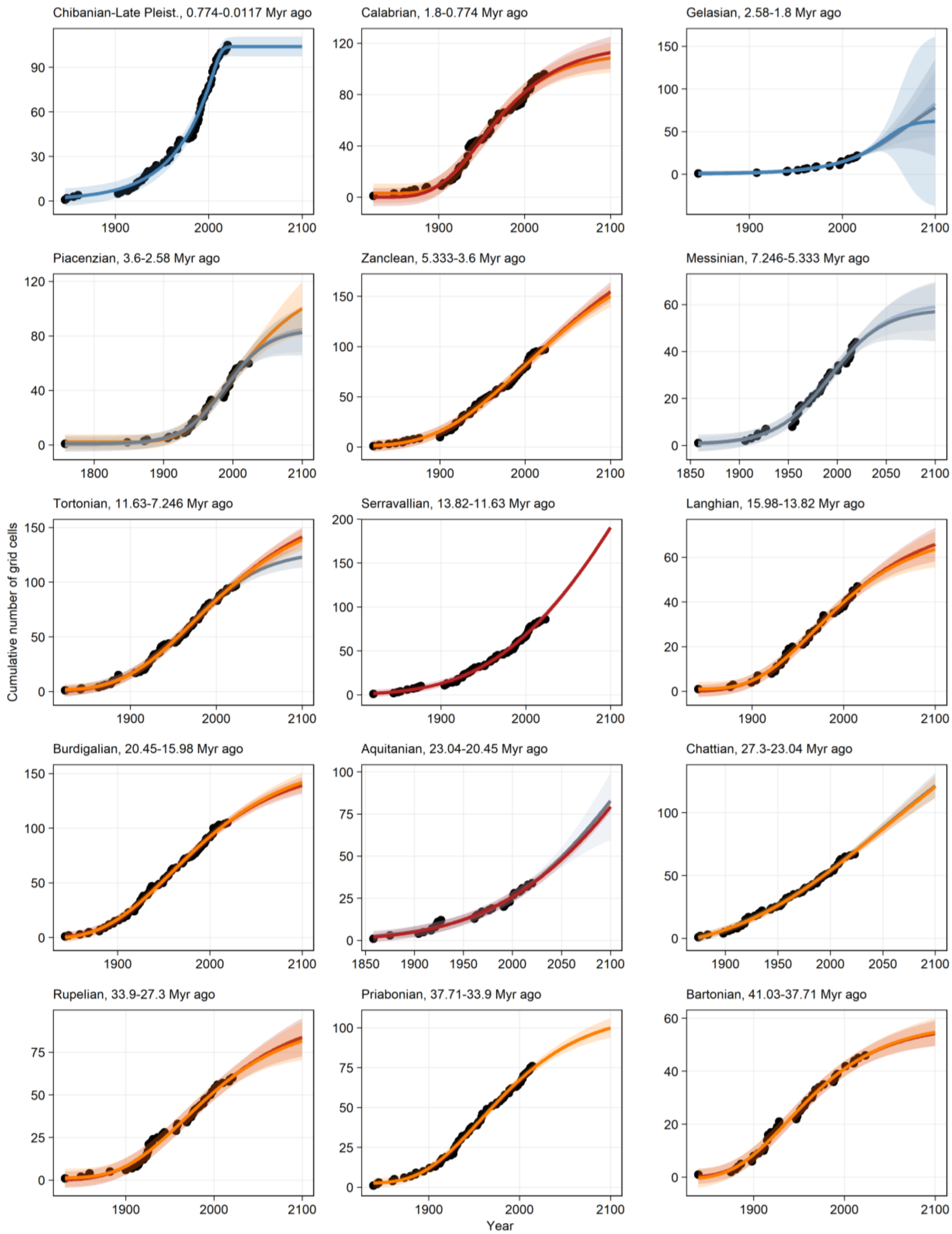

Fig. S12.

Growth models predicting future collection effort across grid cells until the year 2100 for each stage for the 750 km flat-topped grid. Note that for some stages, more than one model qualified as the best (i.e., within ΔAIC = 2). Underlying data is summarized in table S5.

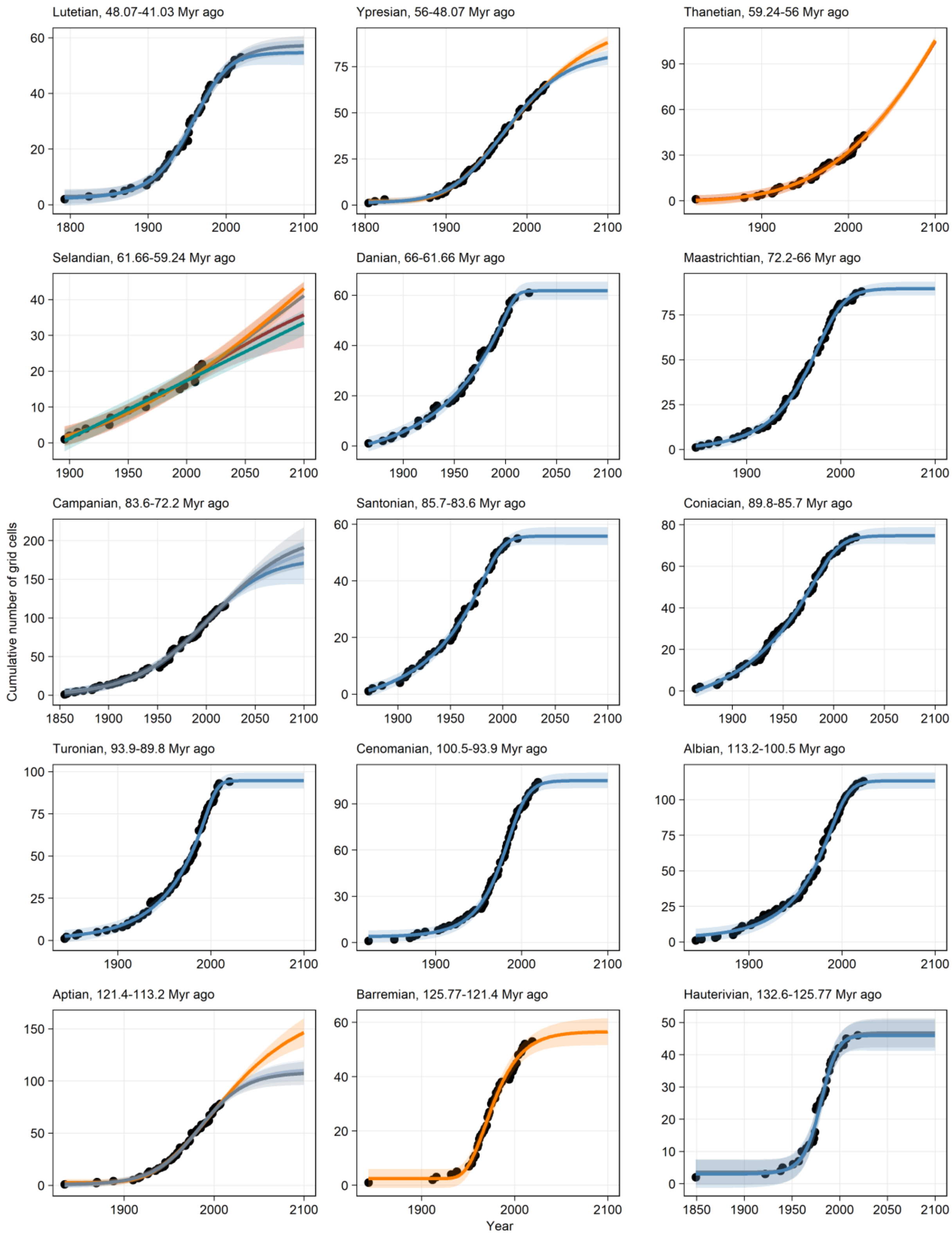

Fig. S12. Continued.

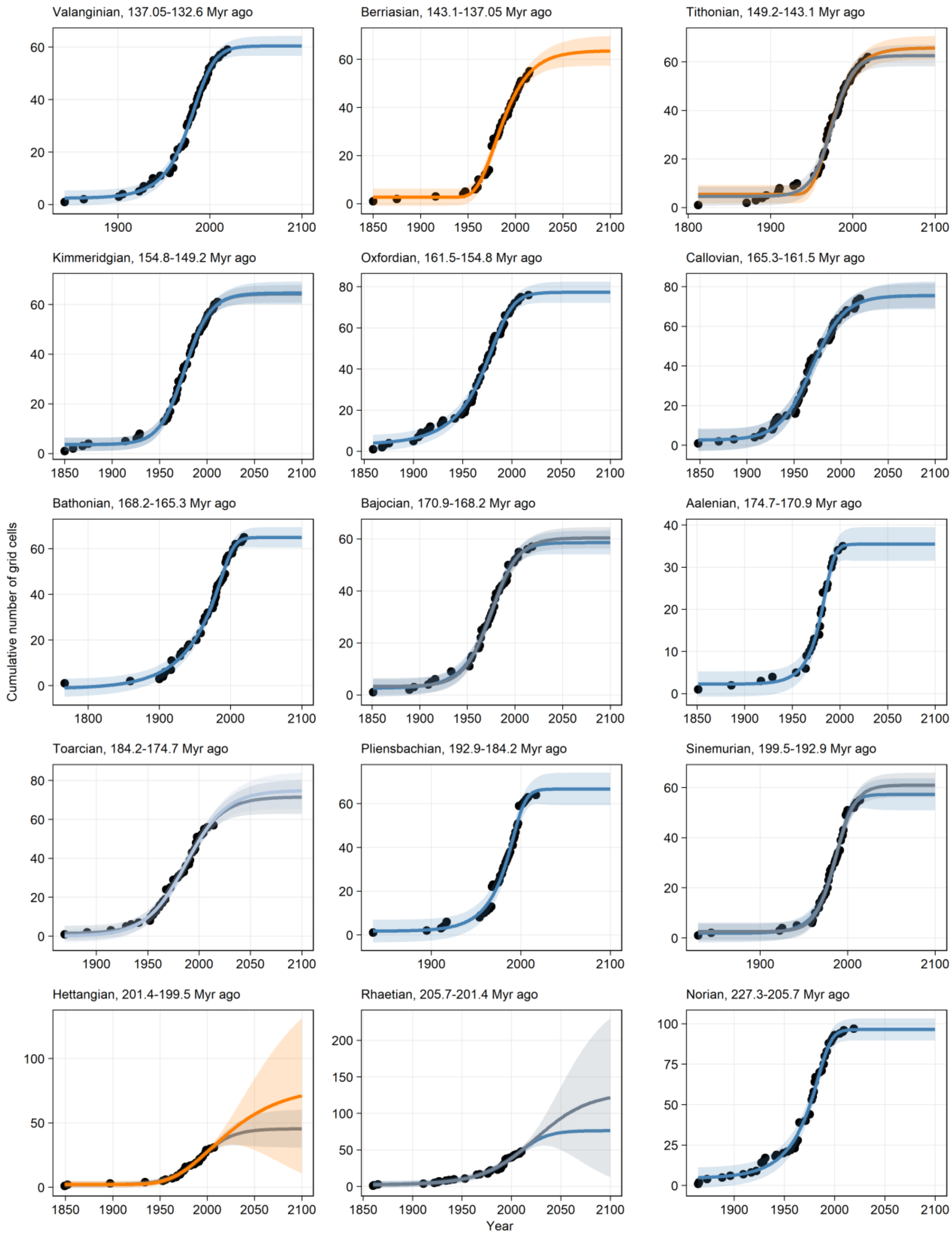

Fig. S12. Continued.

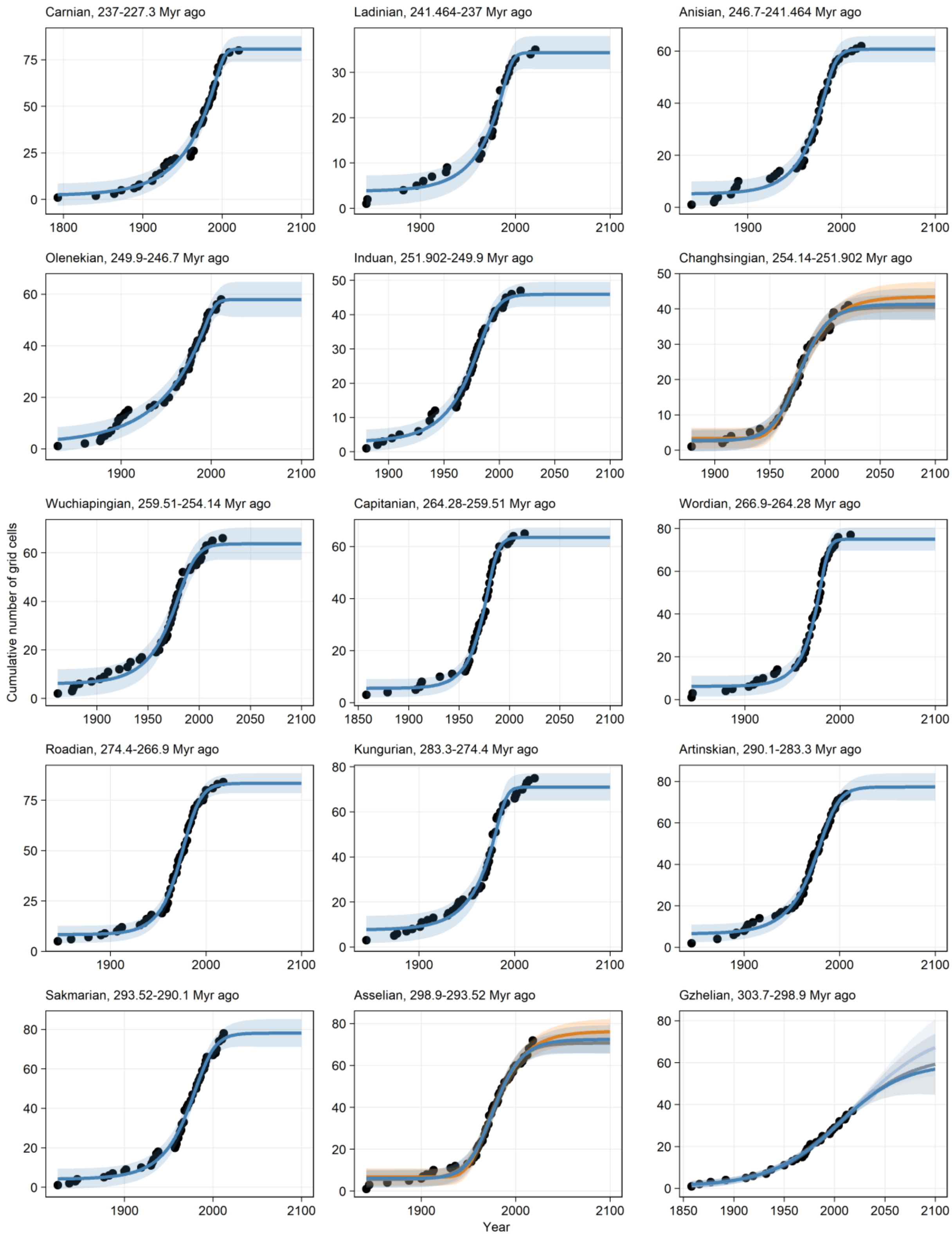

Fig. S12. Continued.

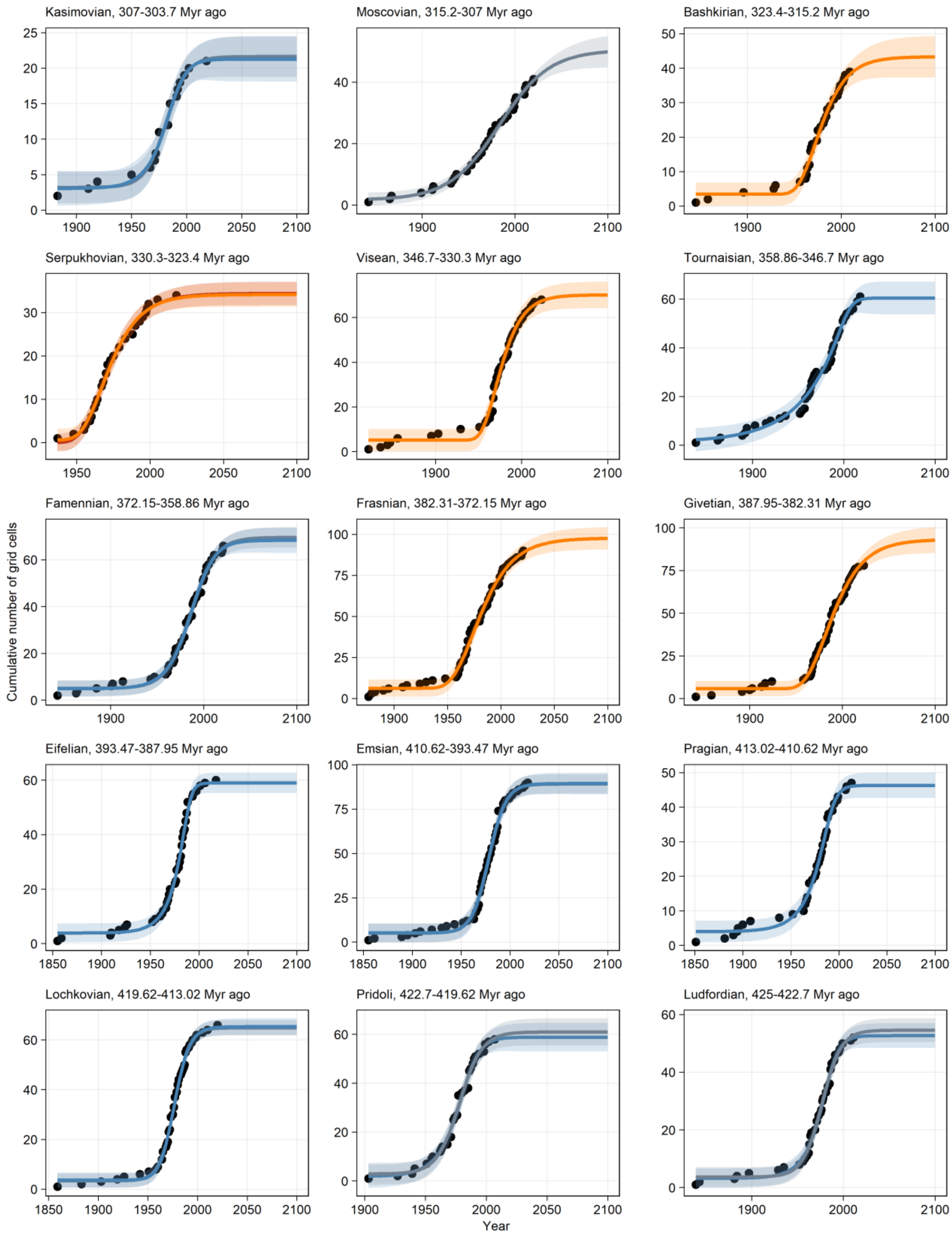

Fig. S12. Continued.

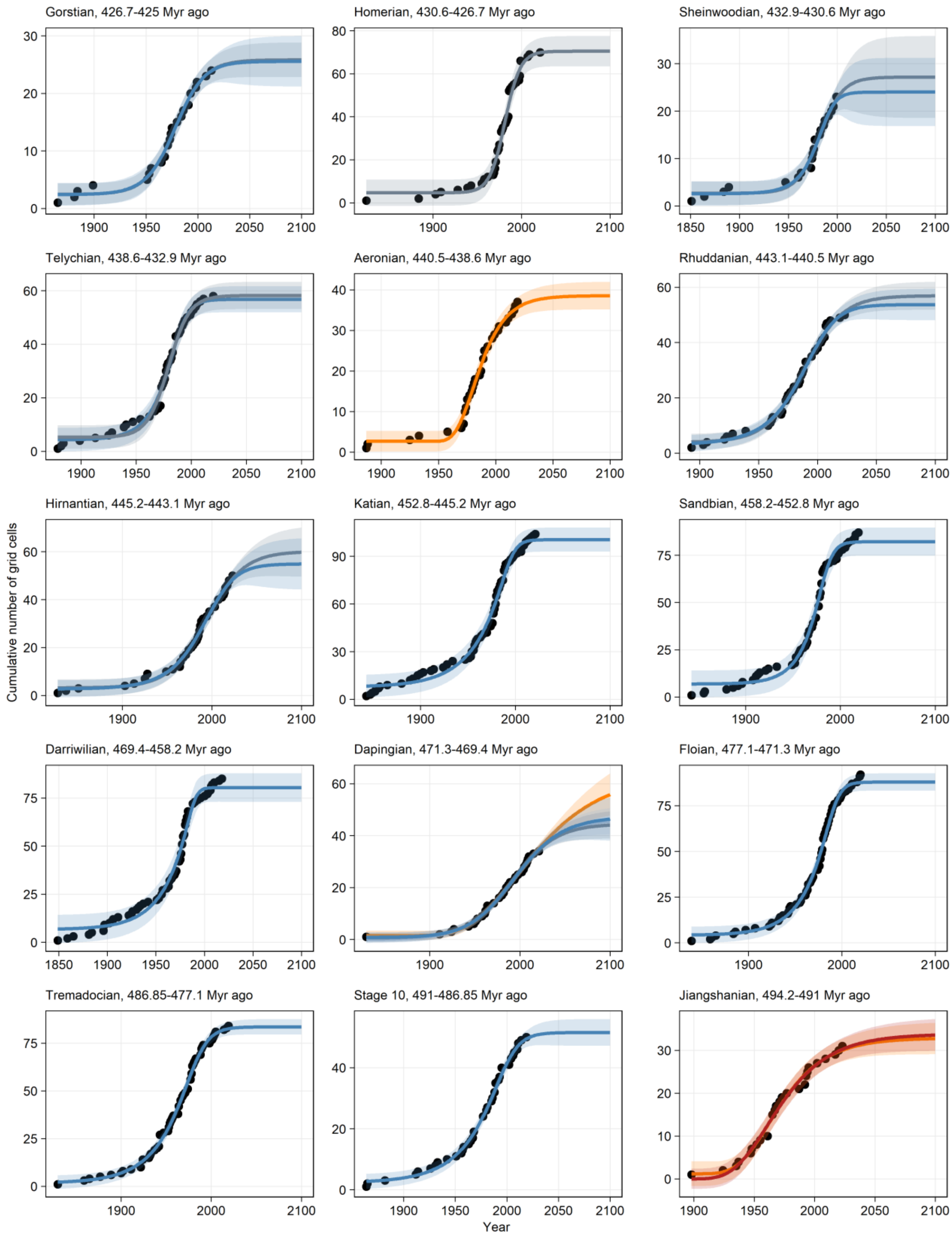

Fig. S12. Continued.

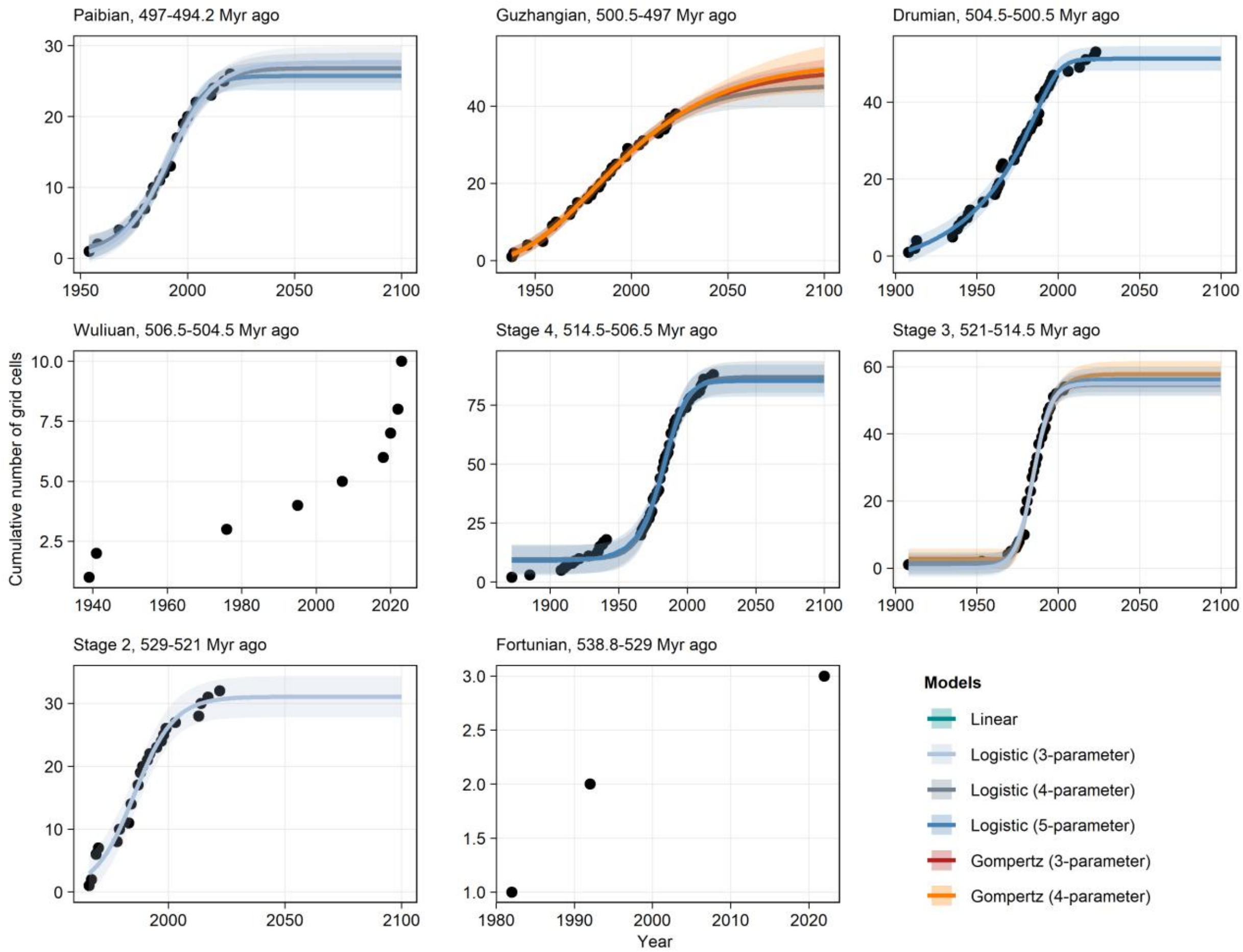

Fig. S12. Continued.

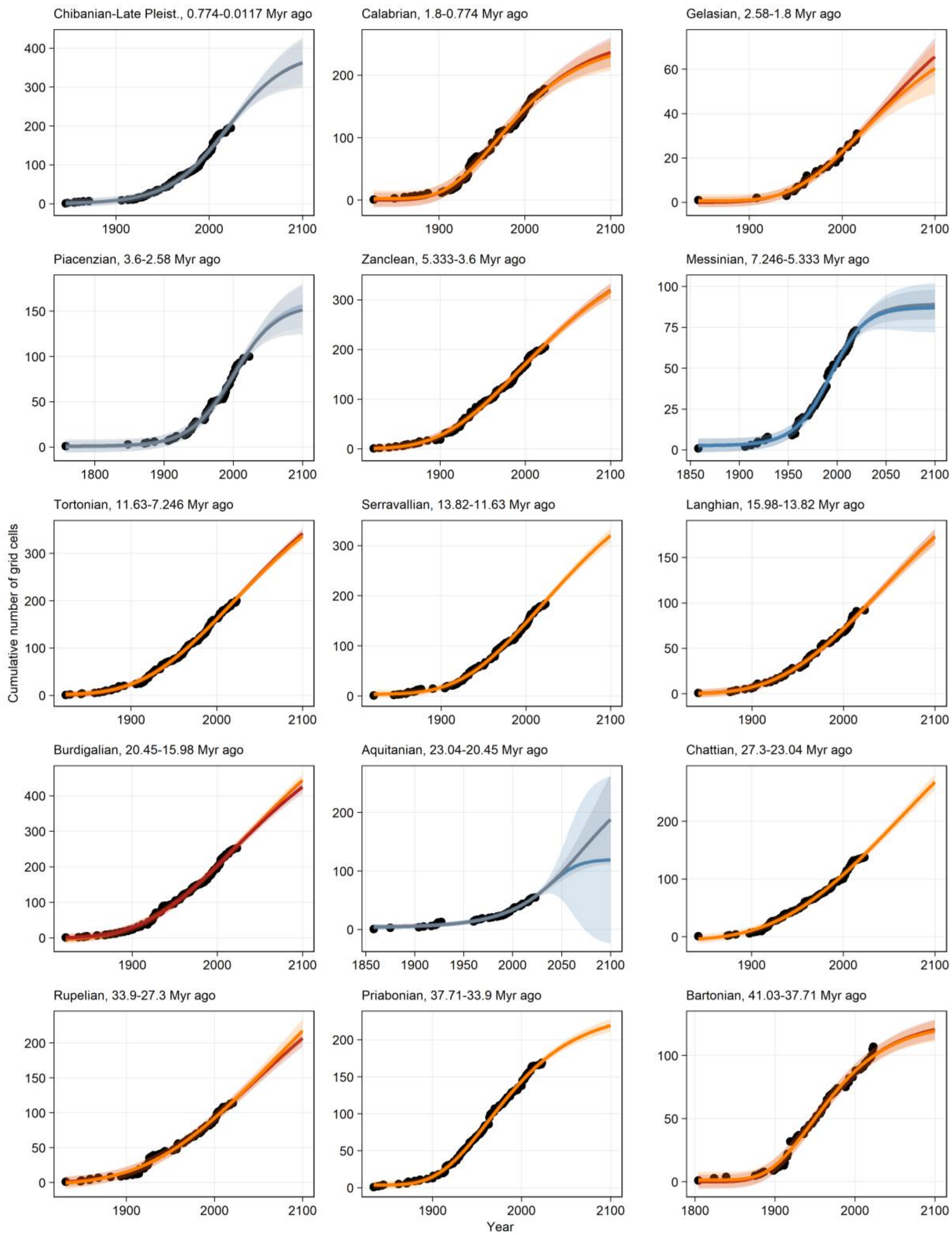

Fig. S13.

Growth models predicting future collection effort across grid cells until the year 2100 for each stage for the 250 km pointy-topped grid (all marine data, not shallow-water only). Note that for some stages, more than one model qualified as the best (i.e., within ΔAIC = 2). Underlying data is summarized in table S6.

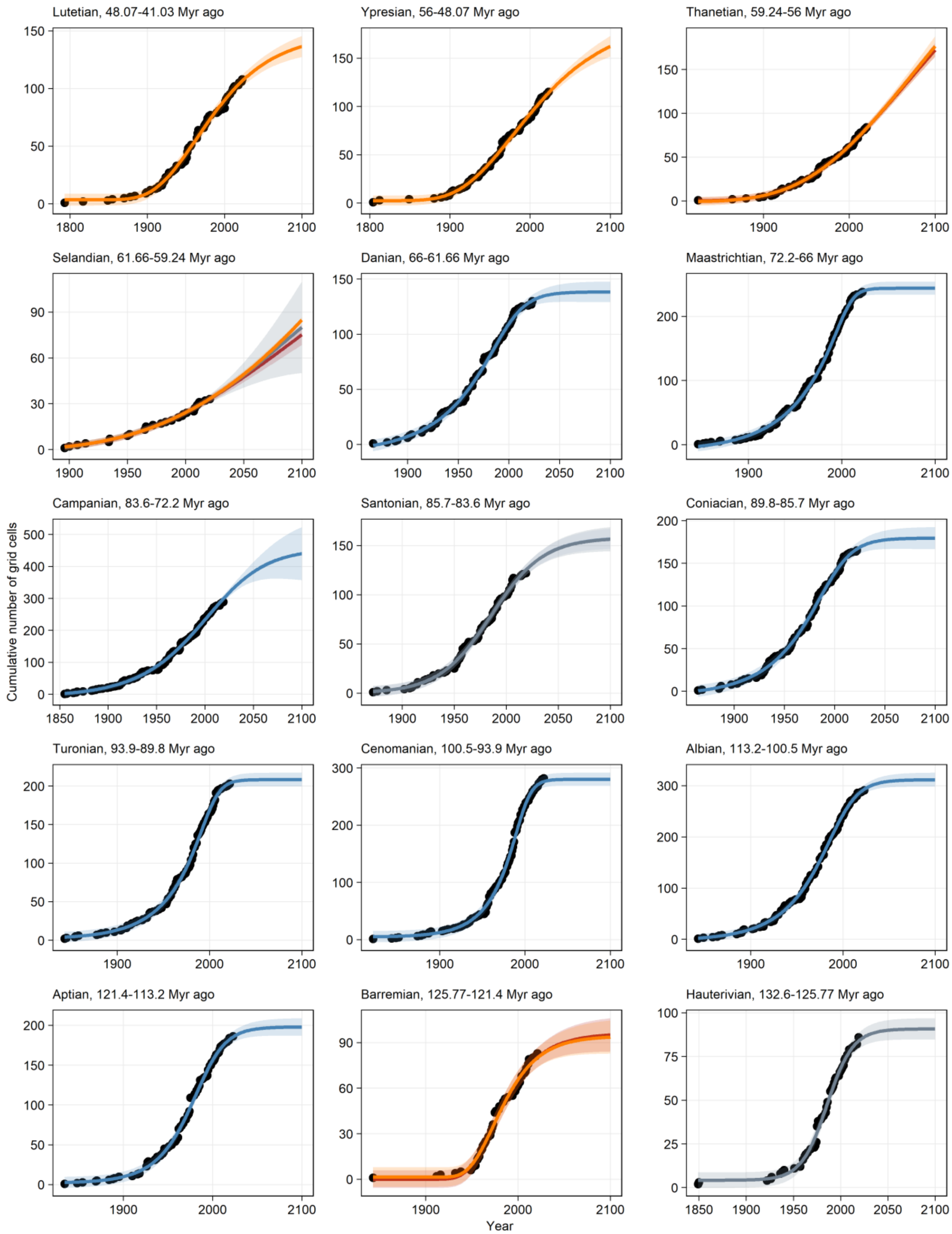

Fig. S13. Continued.

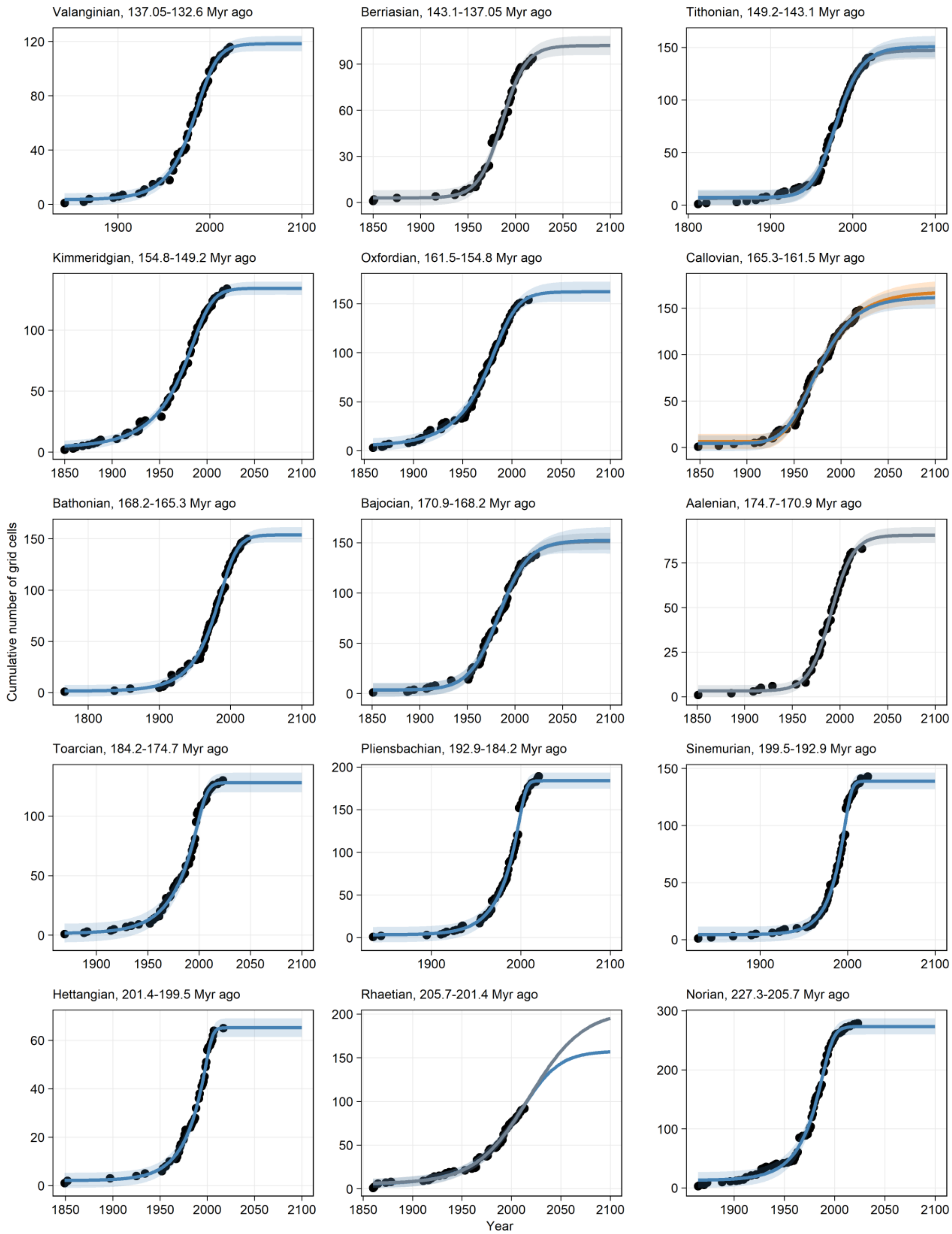

Fig. S13. Continued.

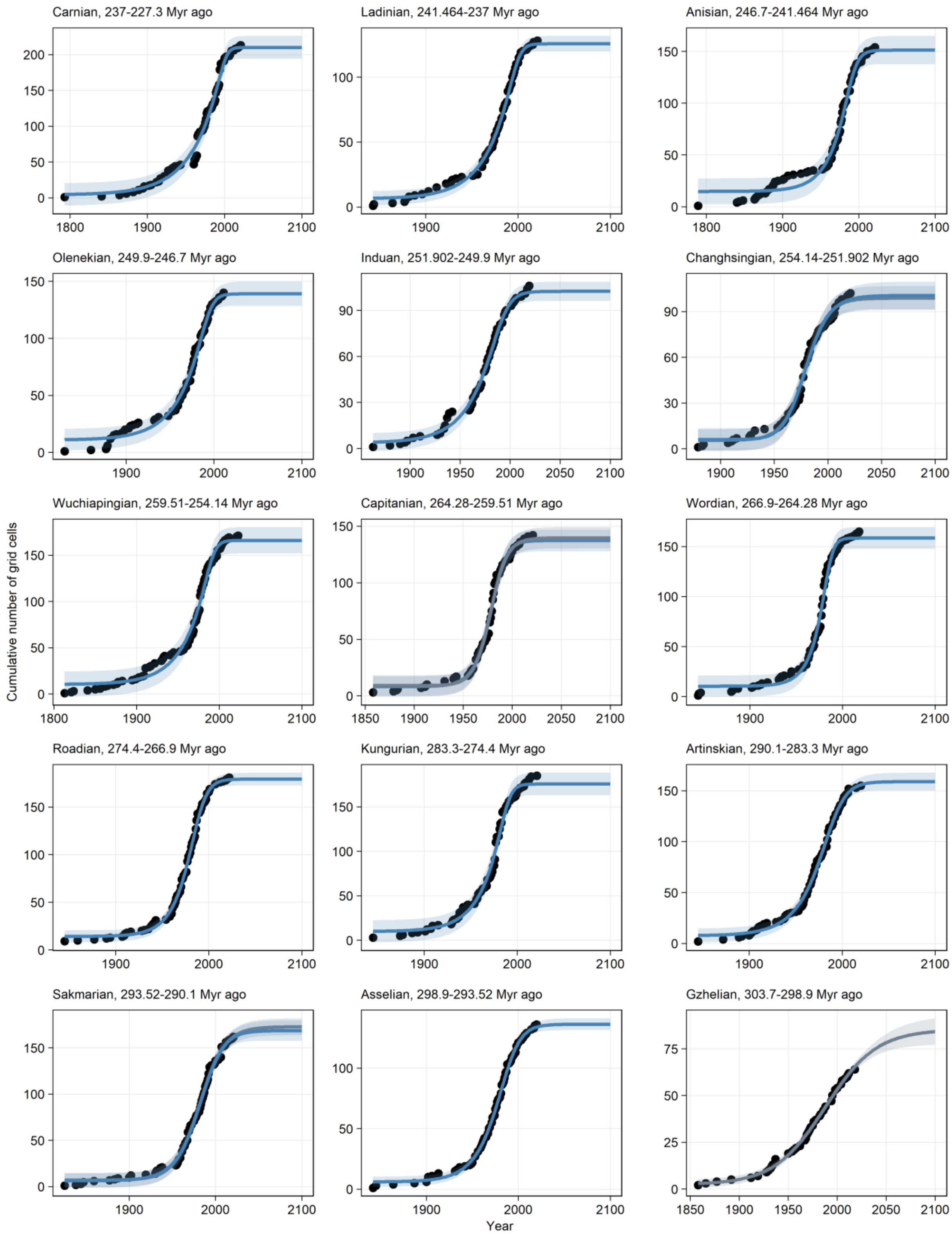

Fig. S13. Continued.

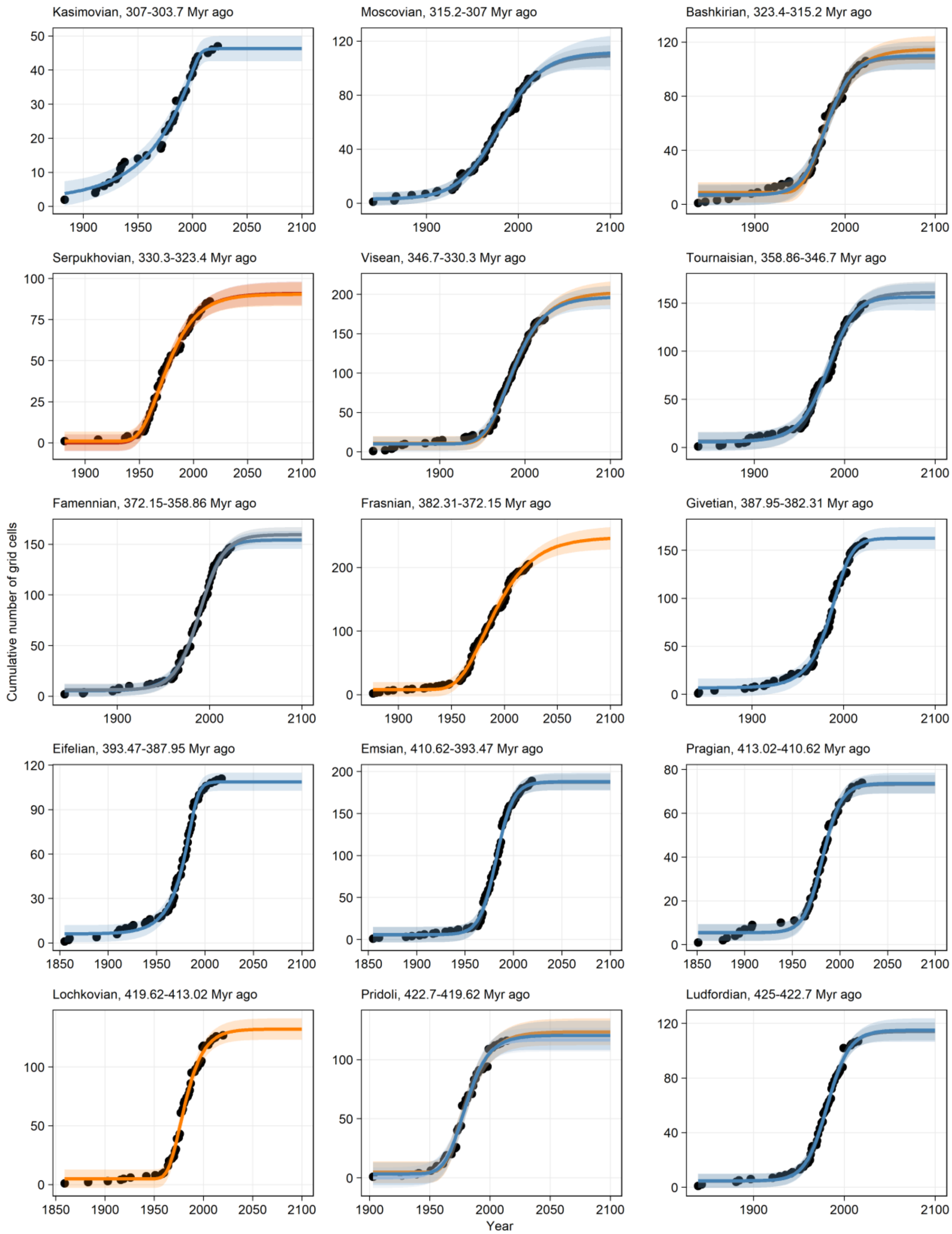

Fig. S13. Continued.

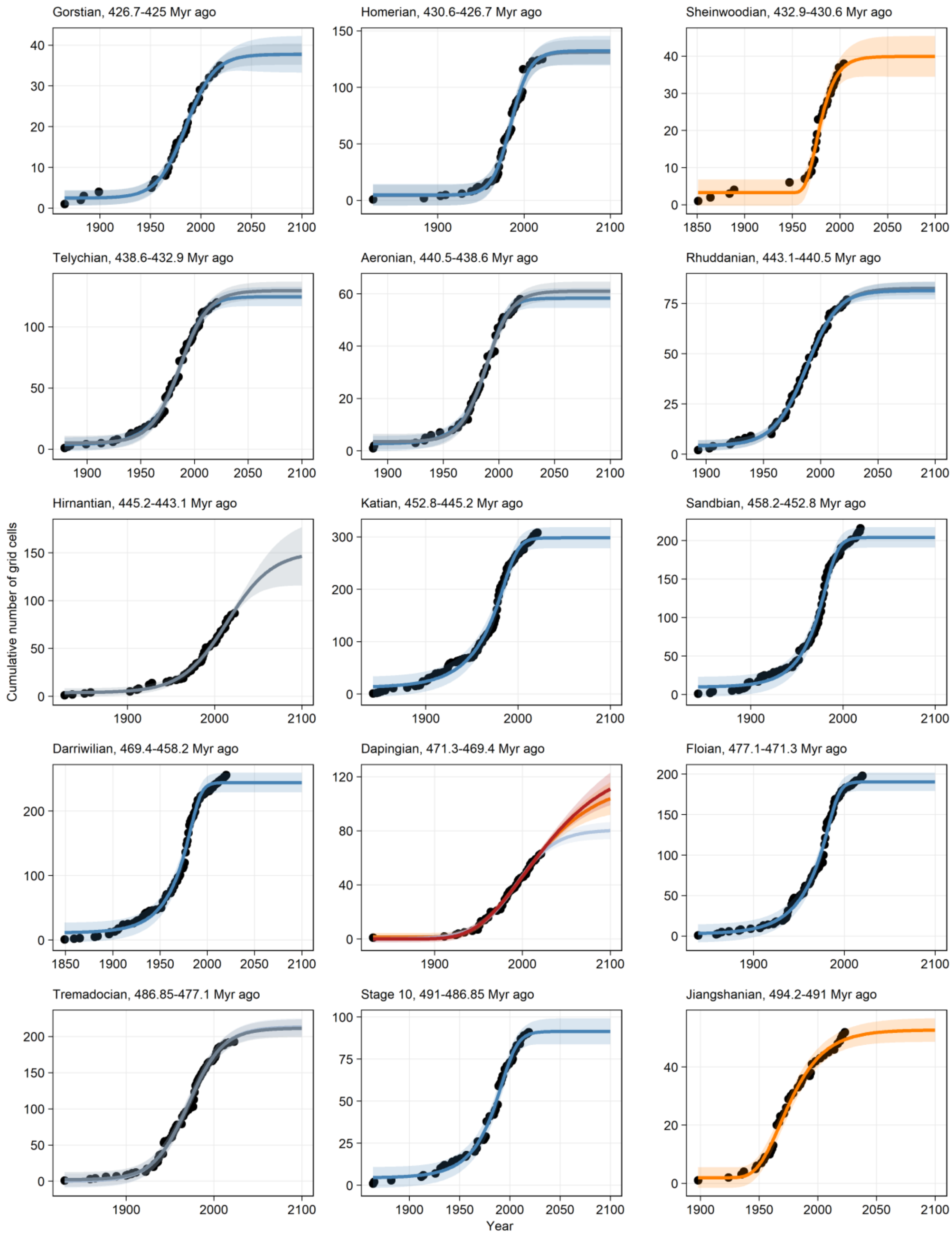

Fig. S13. Continued.

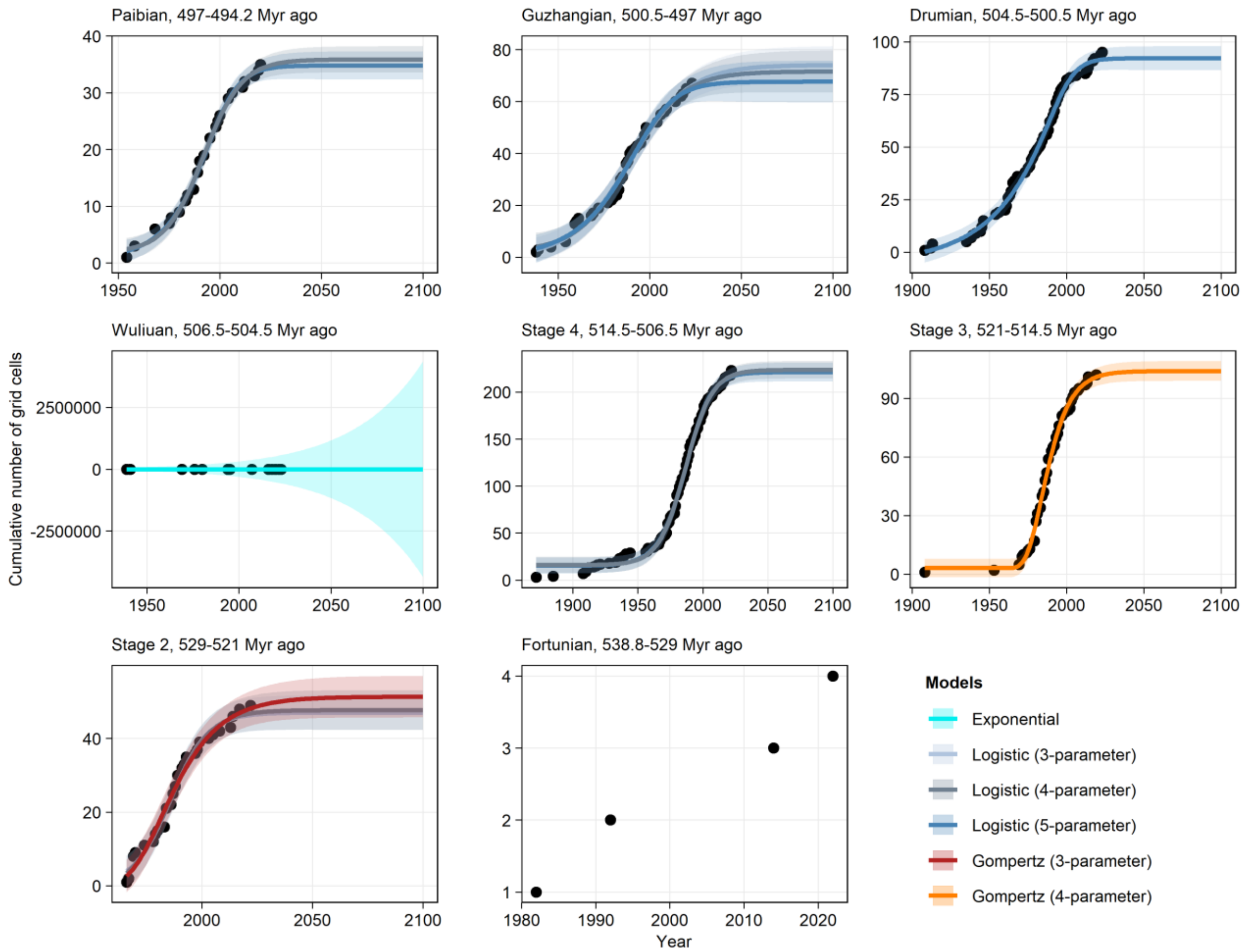

Fig. S13. Continued.

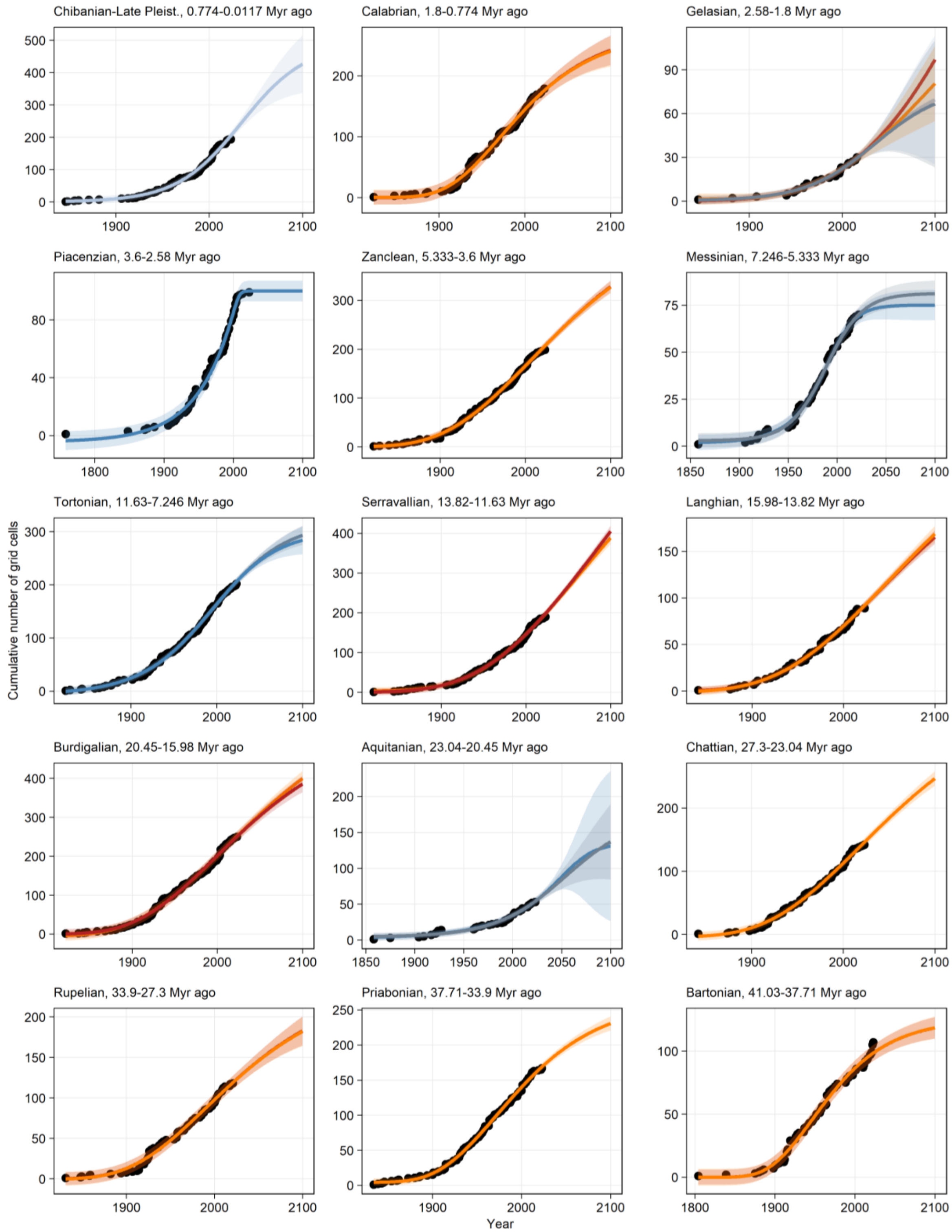

Fig. S14.

Growth models predicting future collection effort across grid cells until the year 2100 for each stage for the 250 km flat-topped grid (all marine data, not shallow-water only). Note that for some stages, more than one model qualified as the best (i.e., within ΔAIC = 2). Underlying data is summarized in table S6.

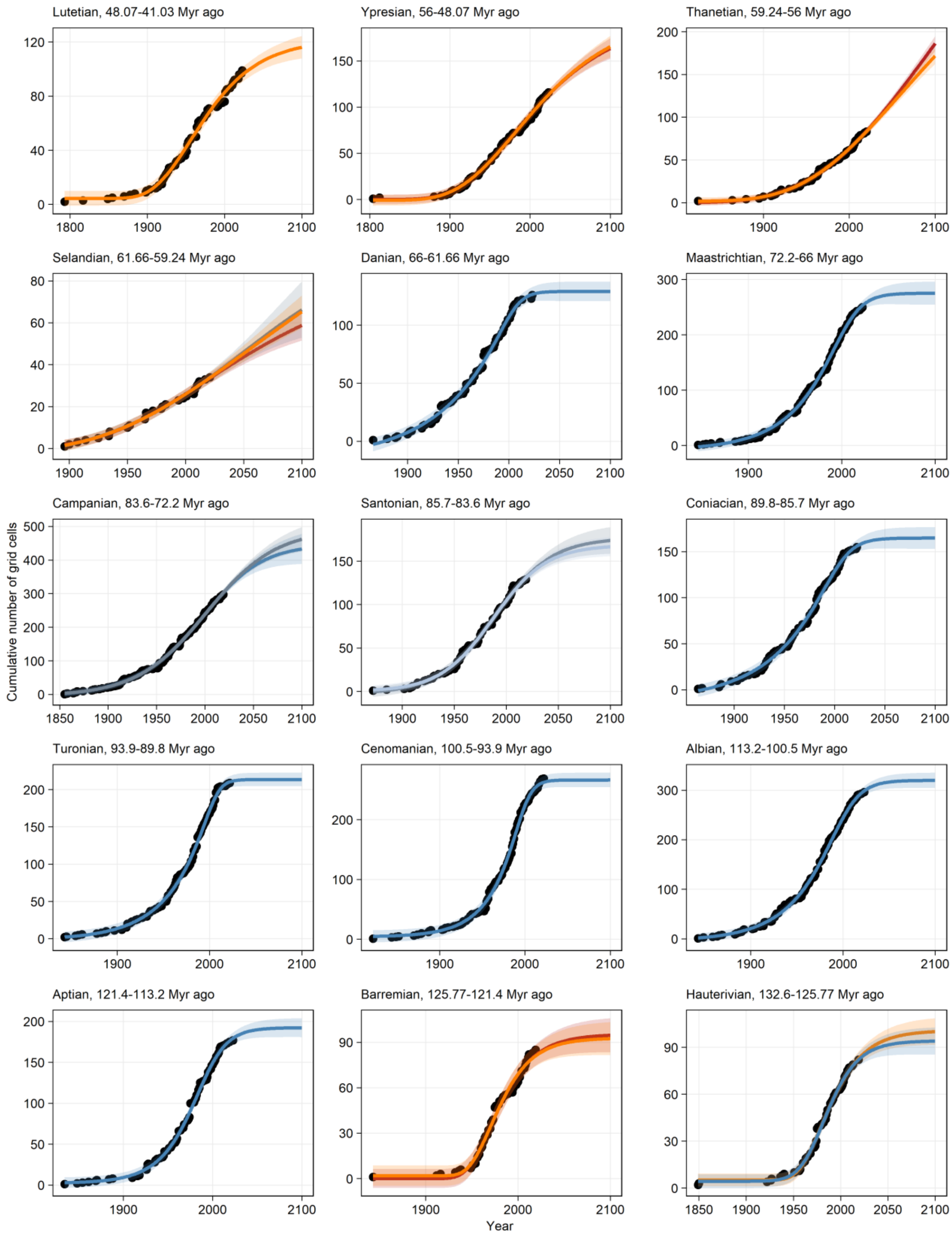

Fig. S14. Continued.

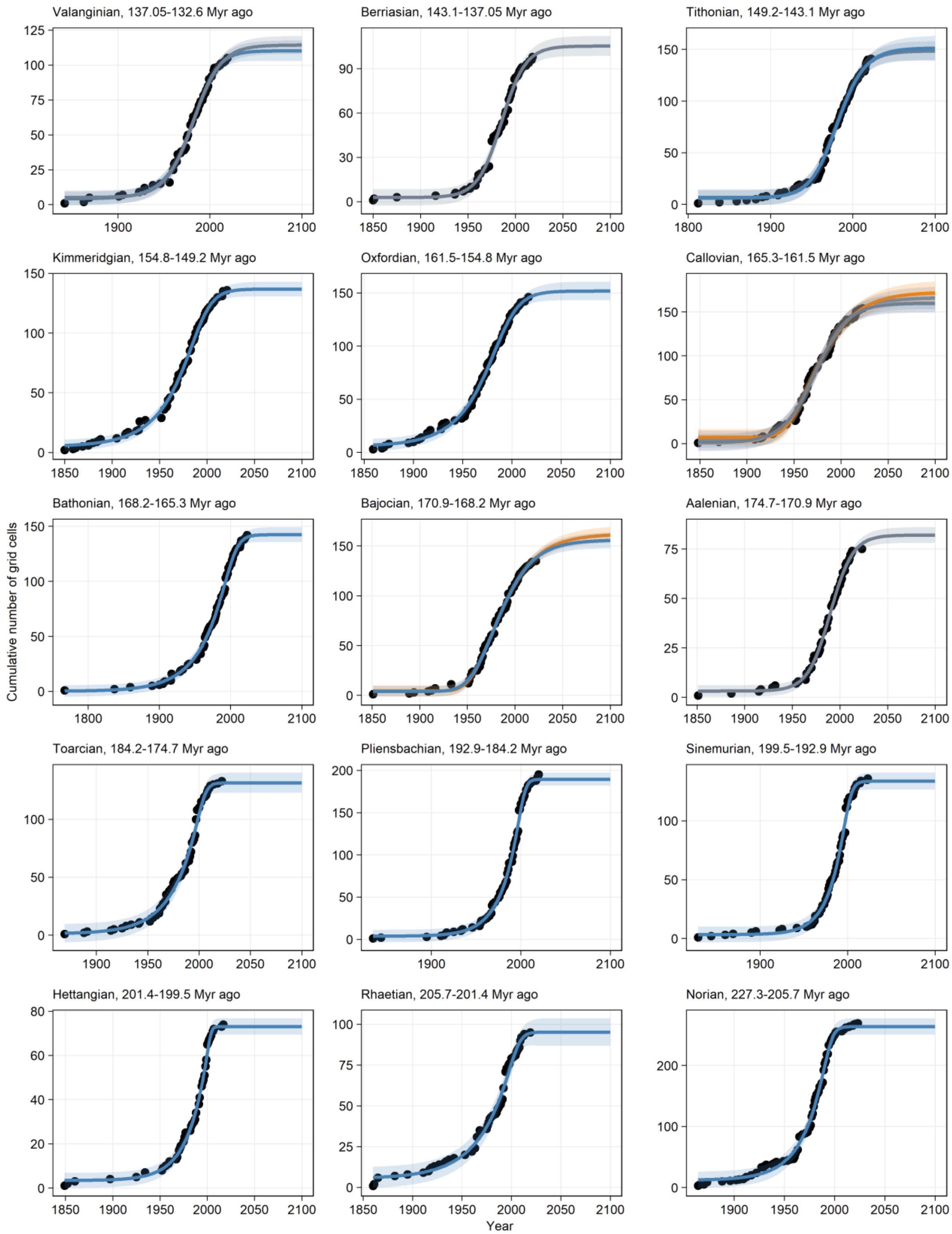

Fig. S14. Continued.

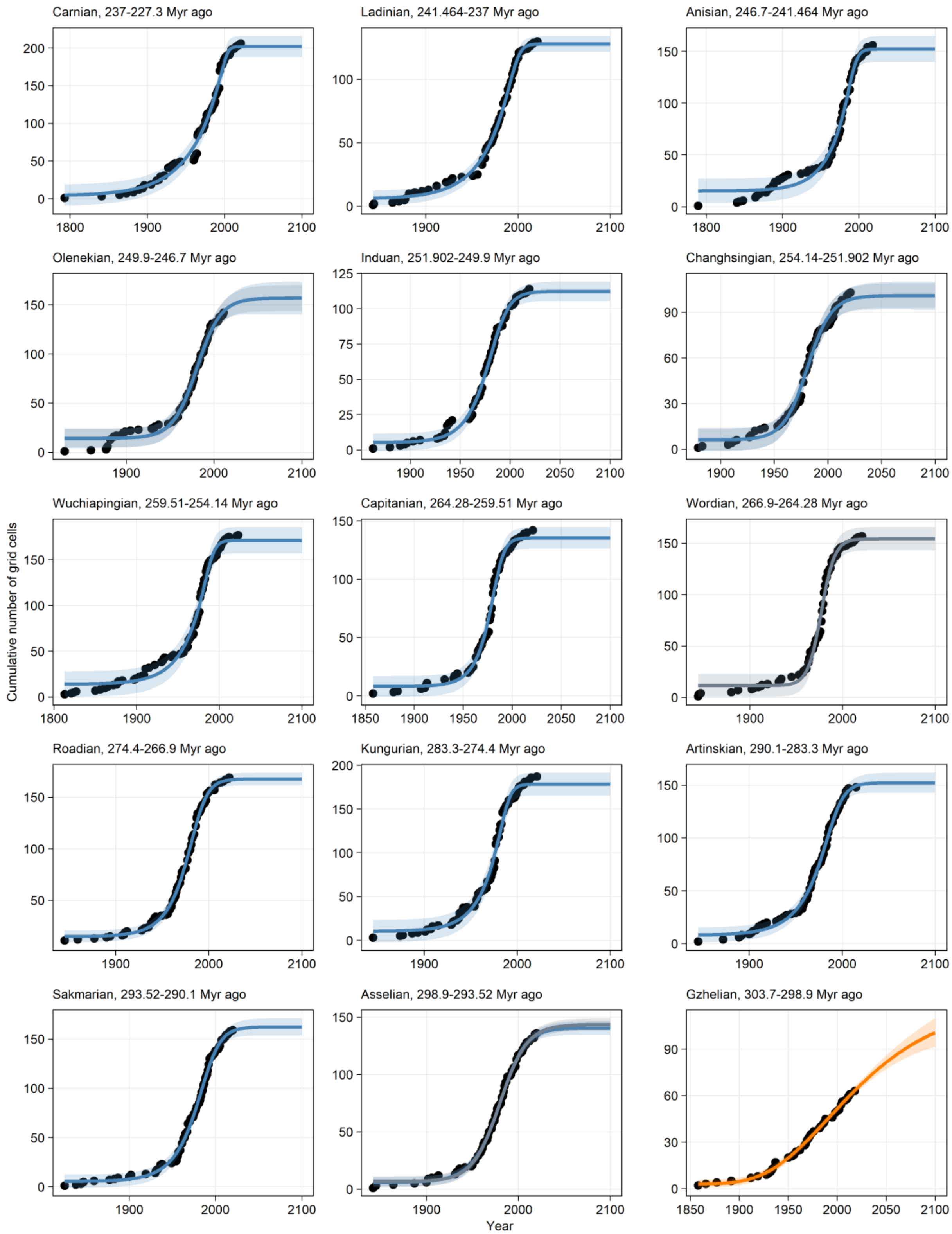

Fig. S14. Continued.

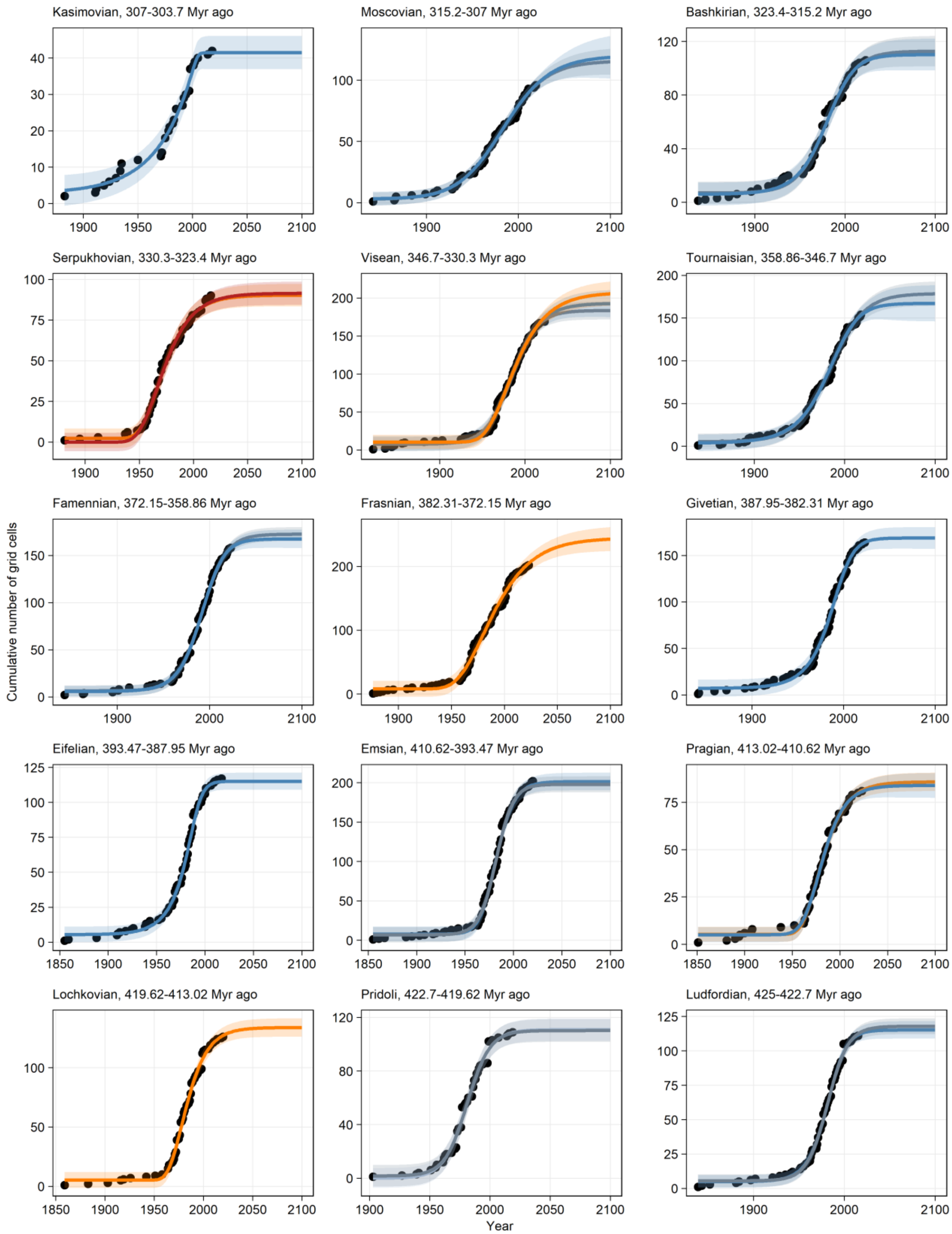

Fig. S14. Continued.

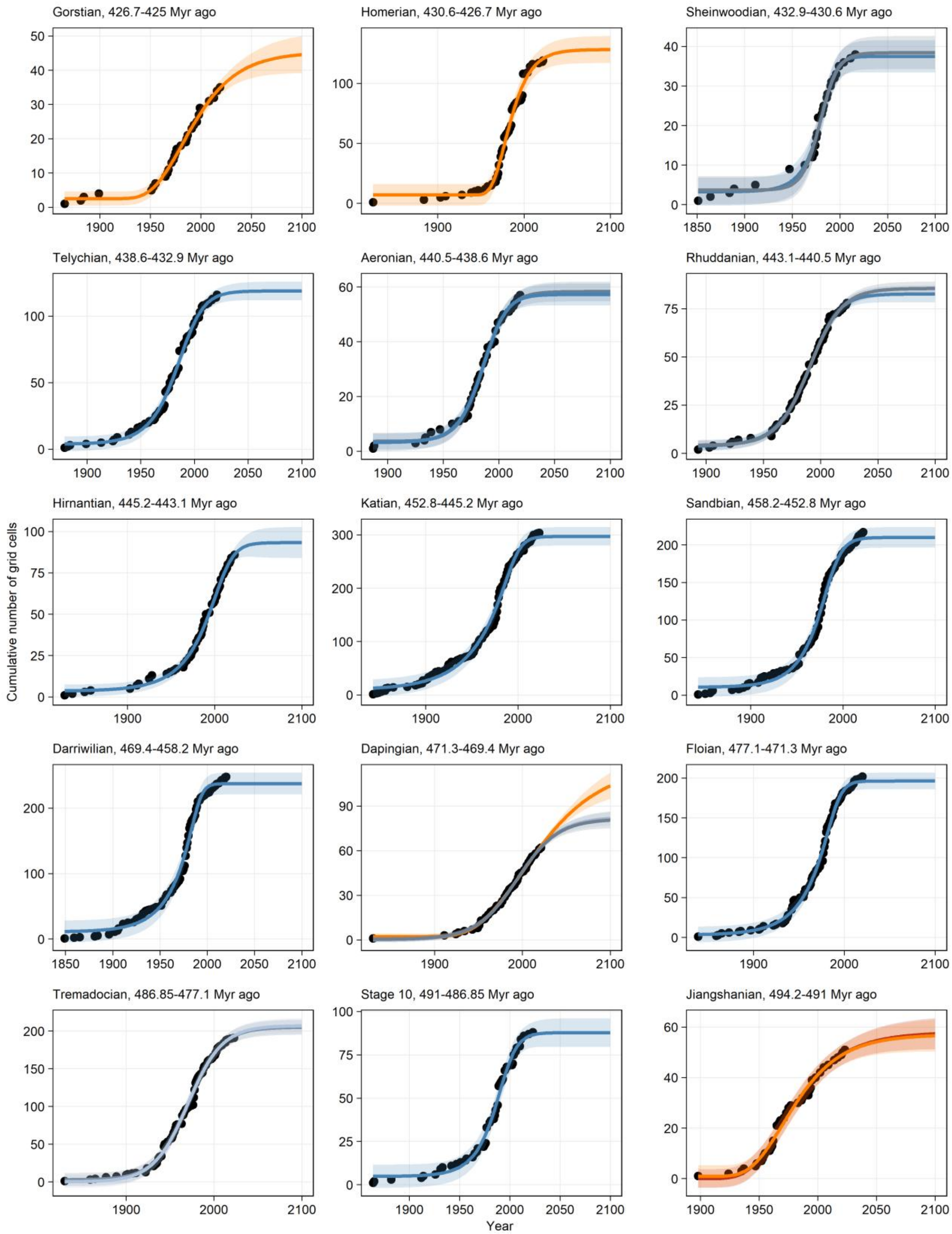

Fig. S14. Continued.

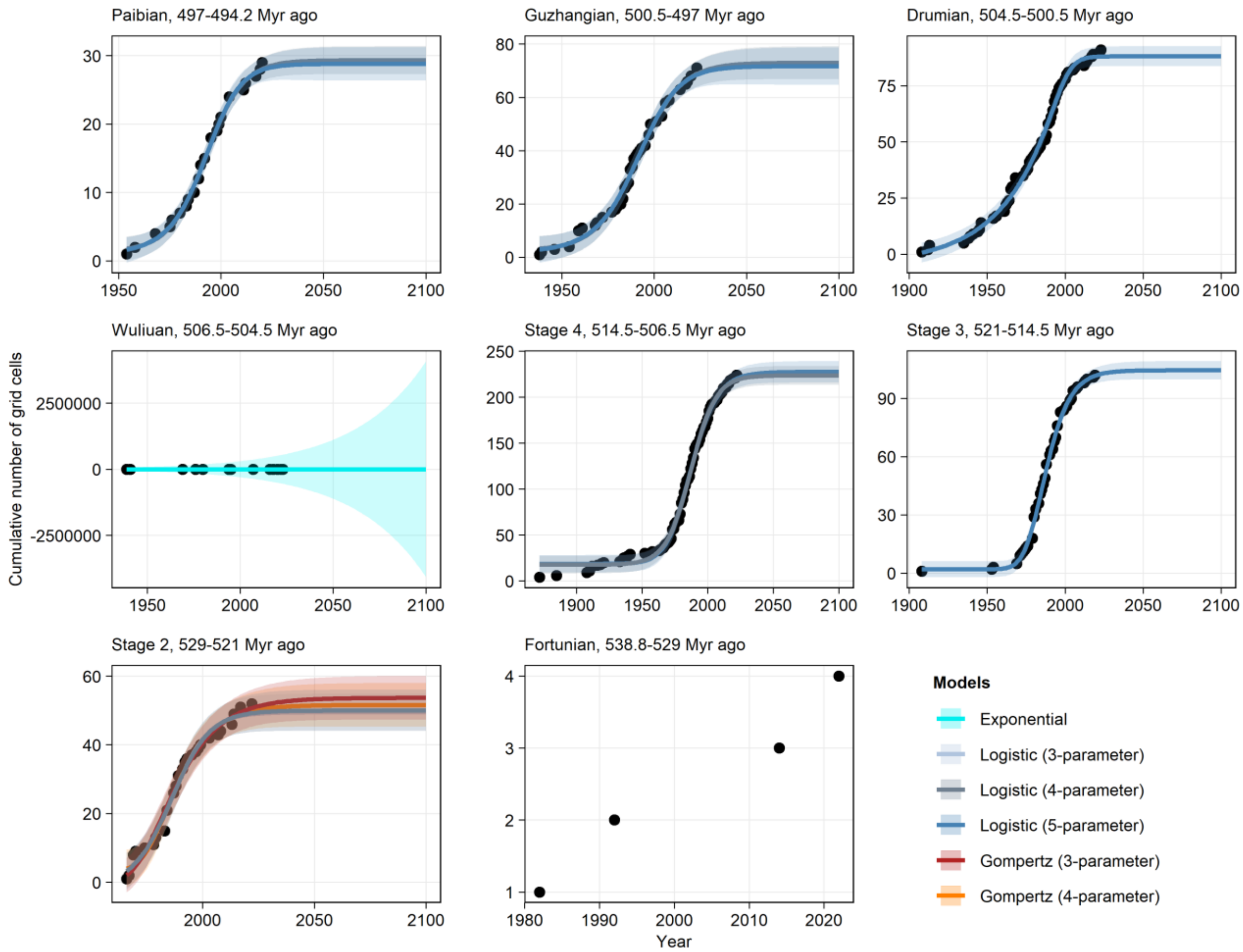

Fig. S14. Continued.

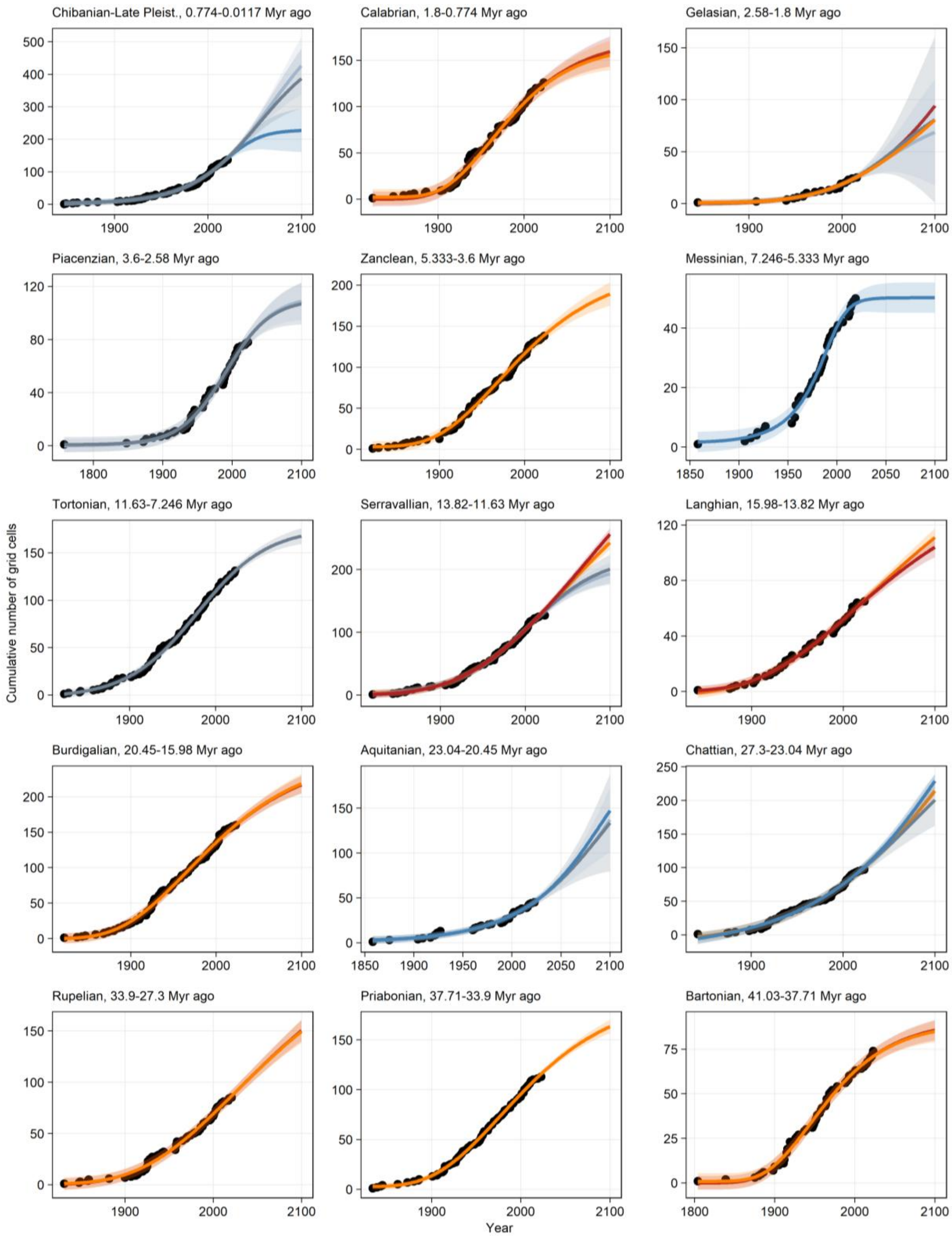

Fig. S15.

Growth models predicting future collection effort across grid cells until the year 2100 for each stage for the 500 km pointy-topped grid (all marine data, not shallow-water only). Note that for some stages, more than one model qualified as the best (i.e., within ΔAIC = 2). Underlying data is summarized in table S6.

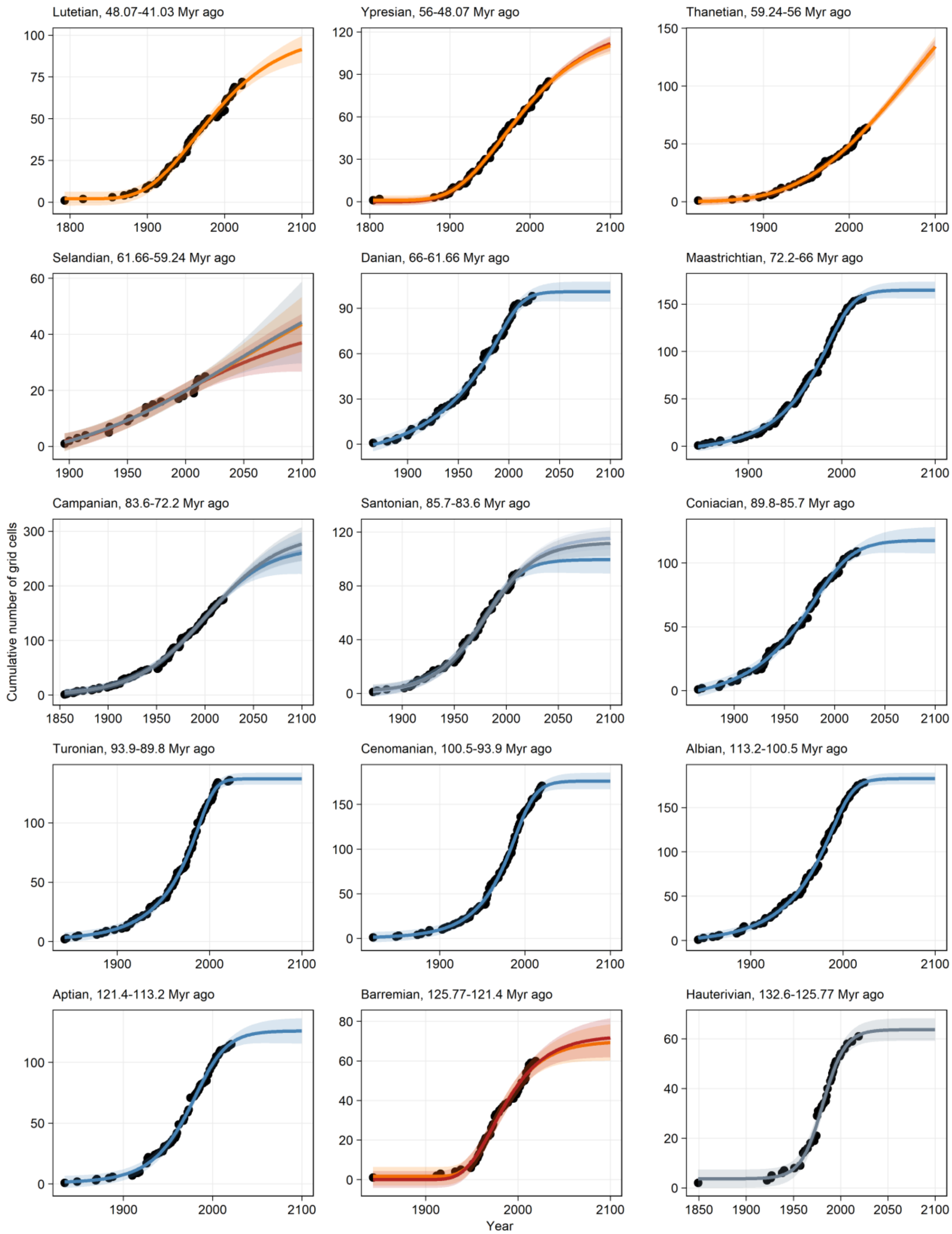

Fig. S15. Continued.

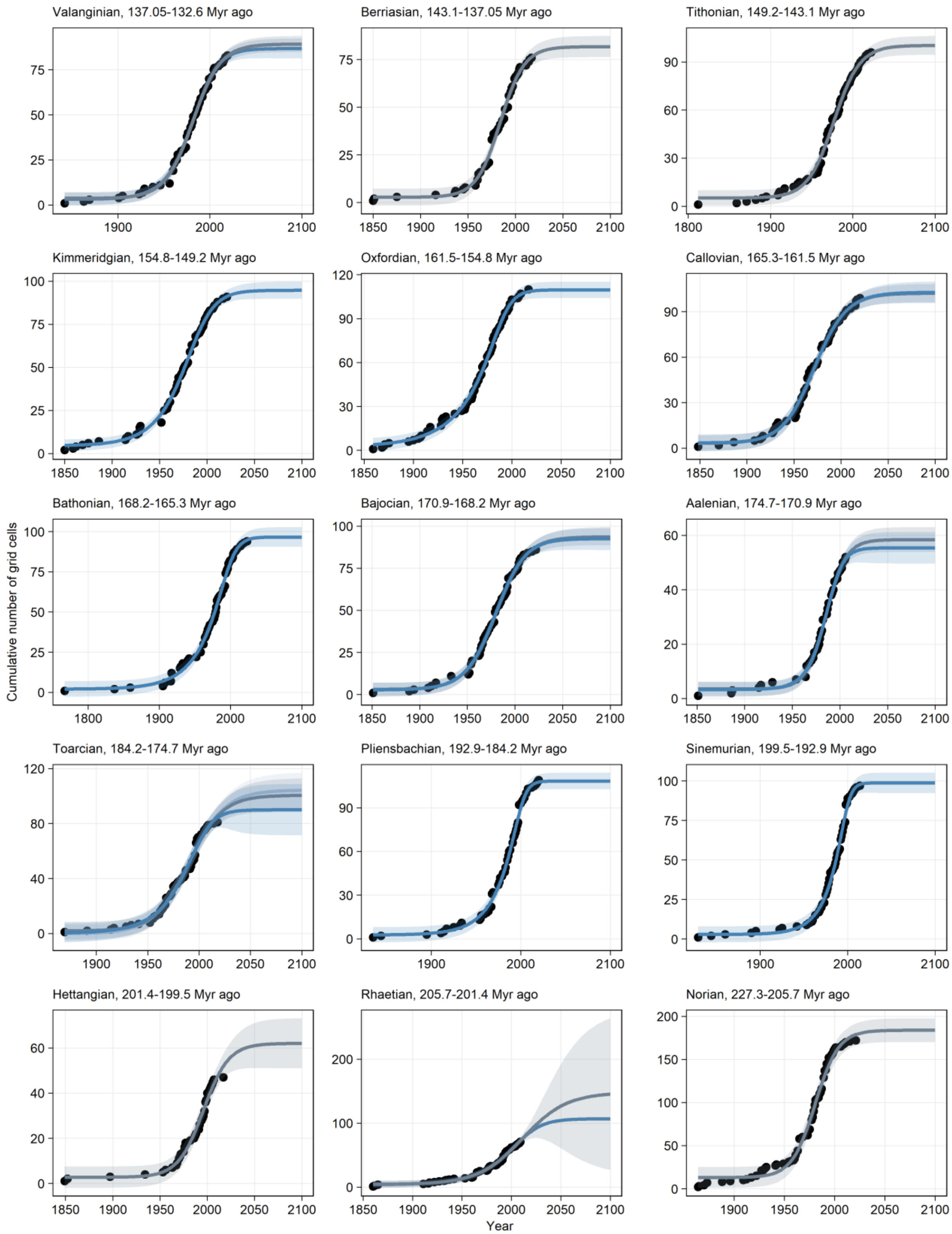

Fig. S15. Continued.

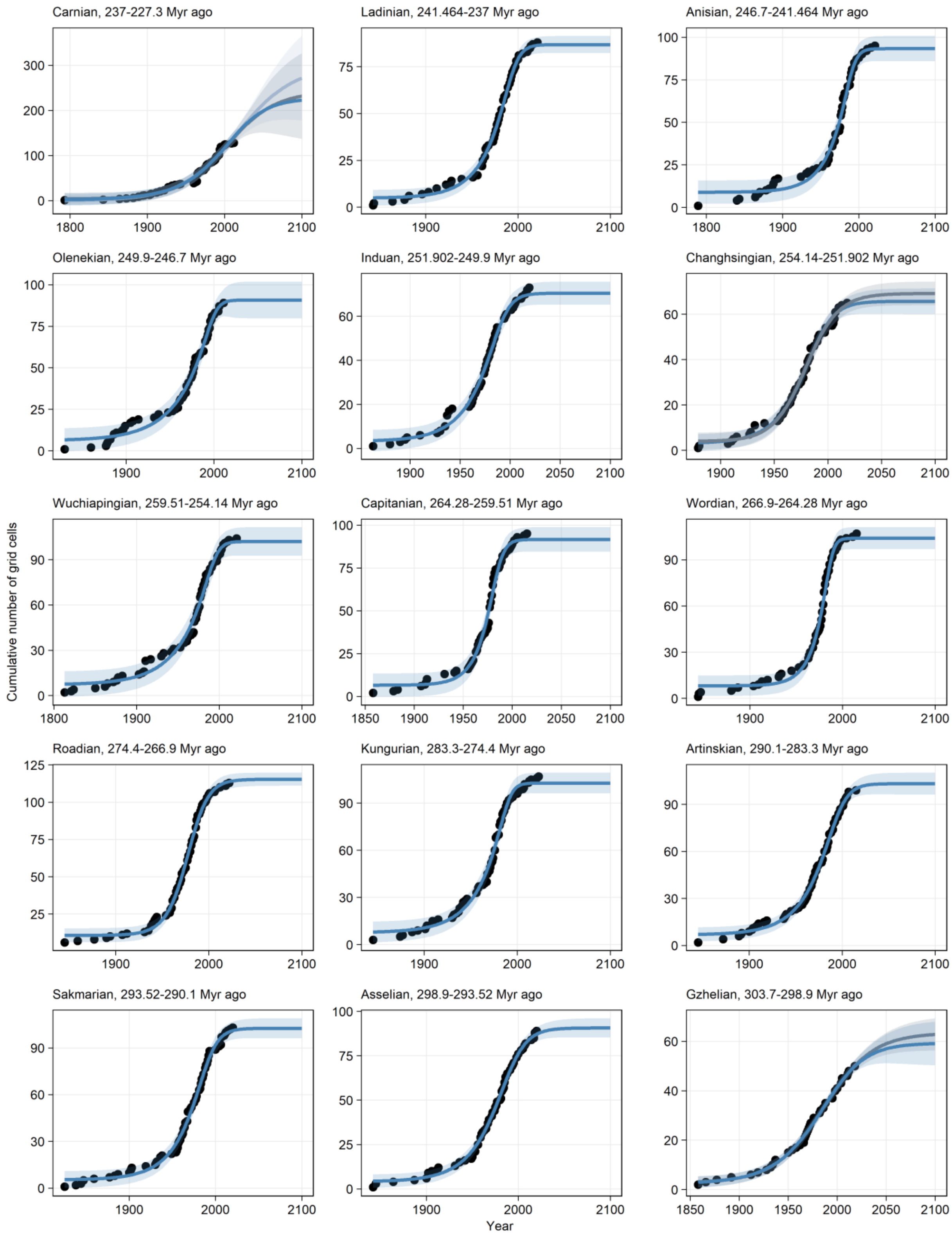

Fig. S15. Continued.

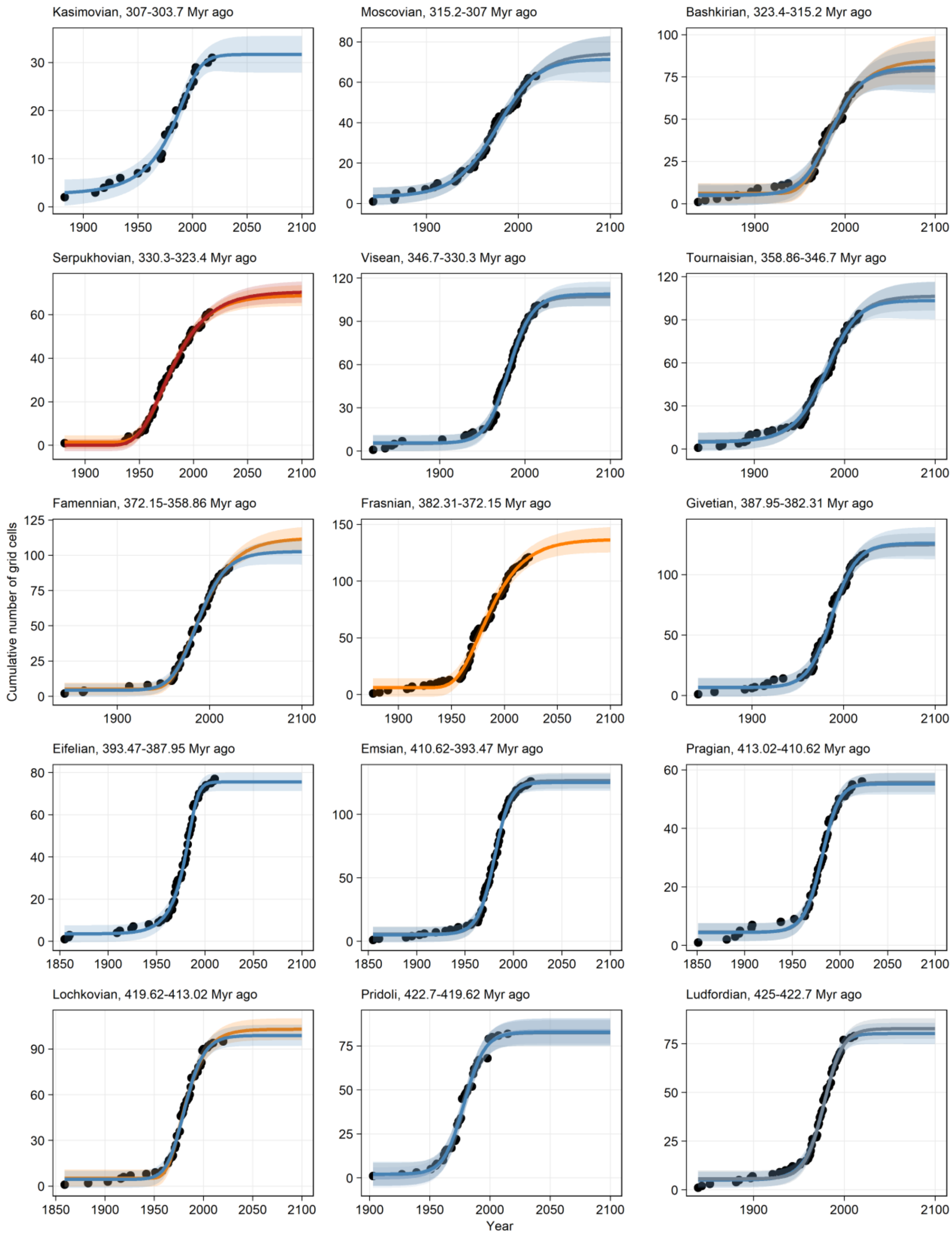

Fig. S15. Continued.

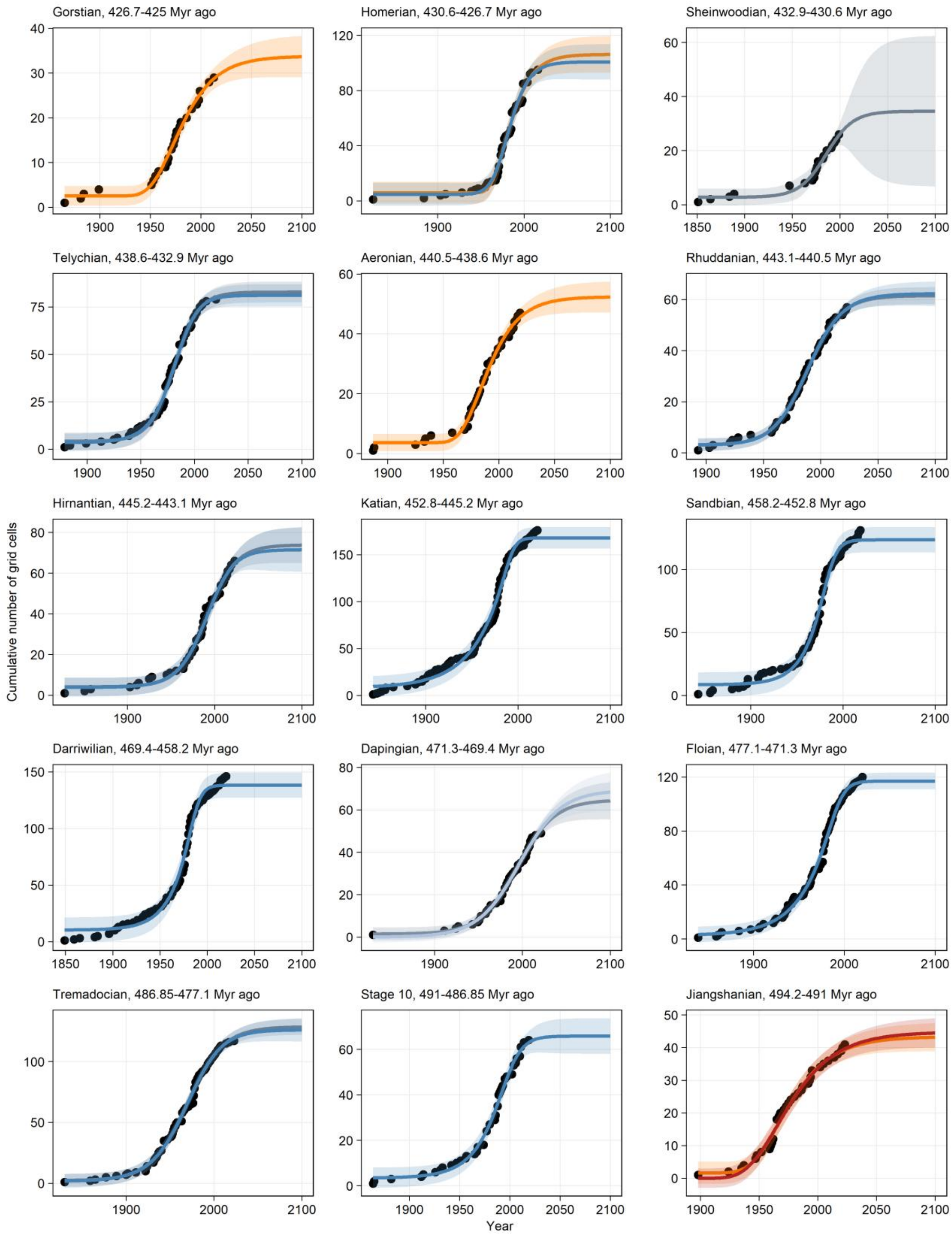

Fig. S15. Continued.

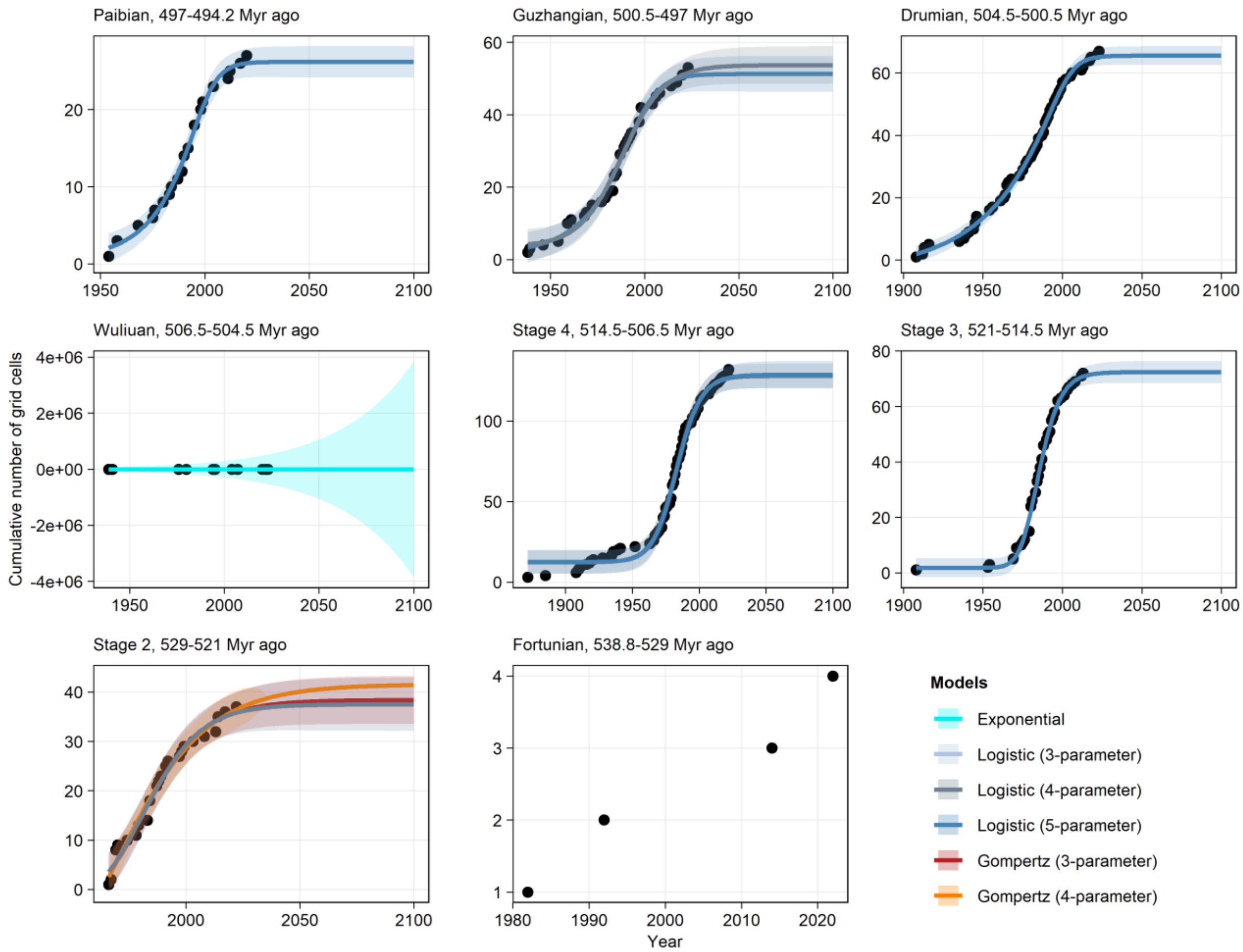

Fig. S15. Continued.

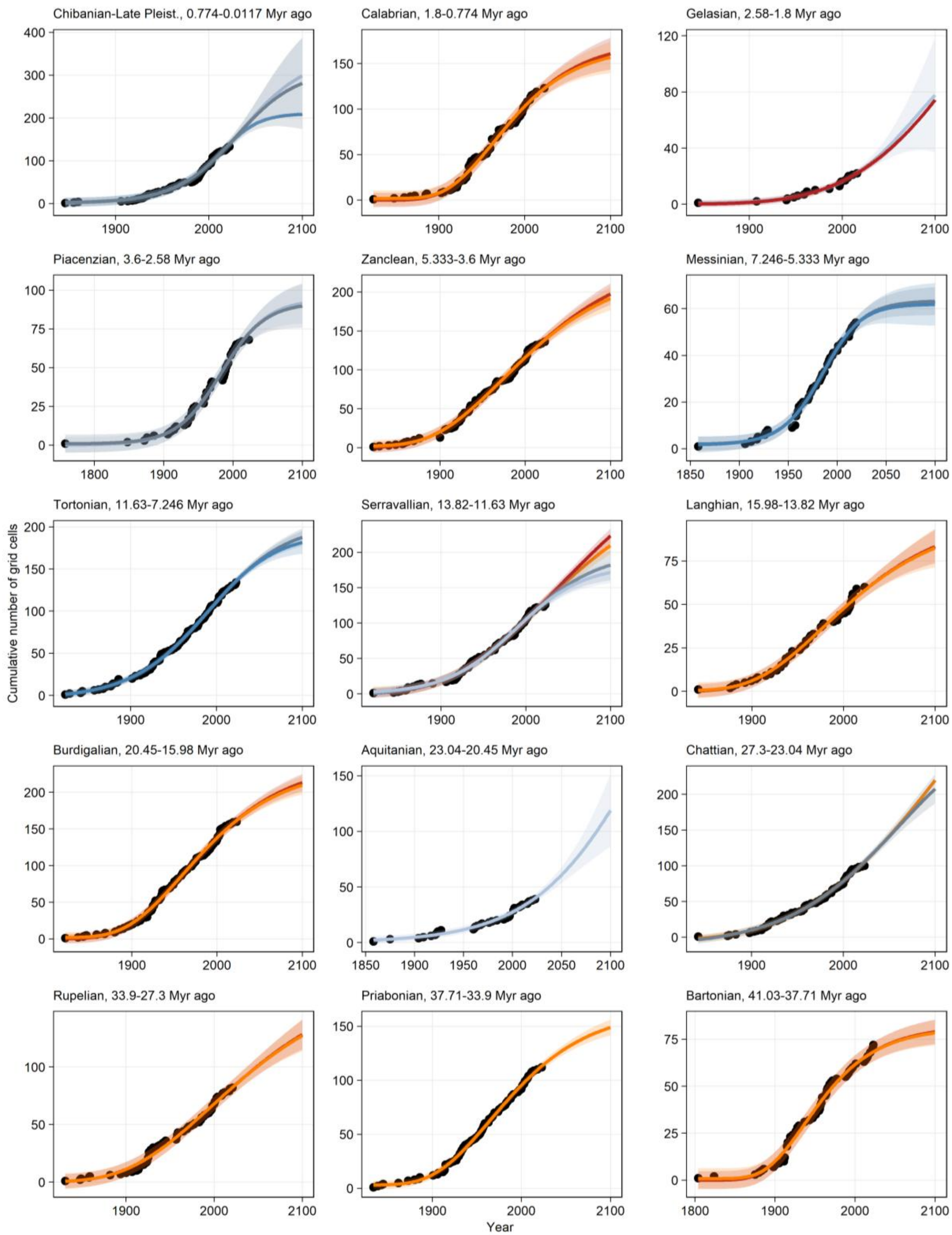

Fig. S16.

Growth models predicting future collection effort across grid cells until the year 2100 for each stage for the 500 km flat-topped grid (all marine data, not shallow-water only). Note that for some stages, more than one model qualified as the best (i.e., within ΔAIC = 2). Underlying data is summarized in table S6.

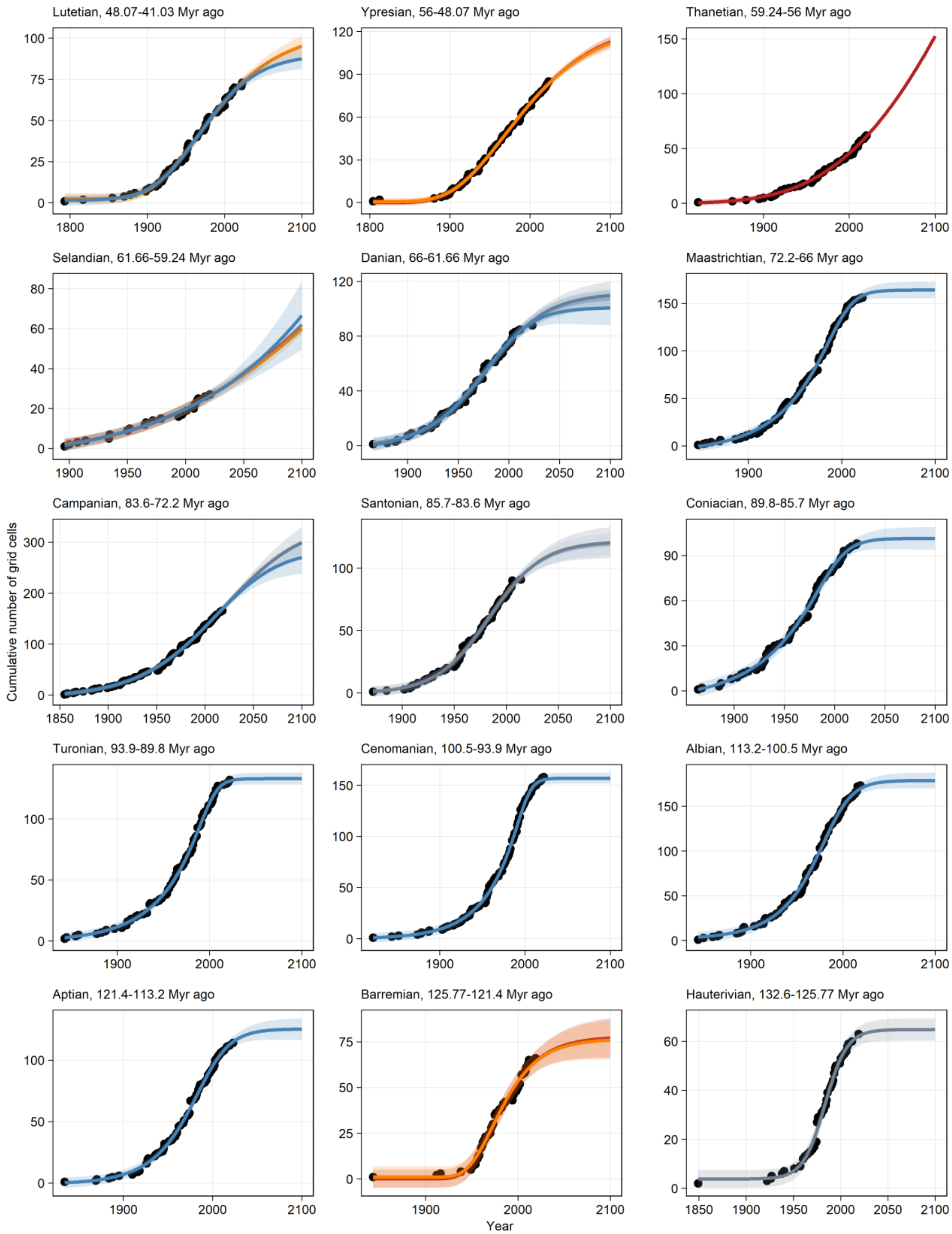

Fig. S16. Continued.

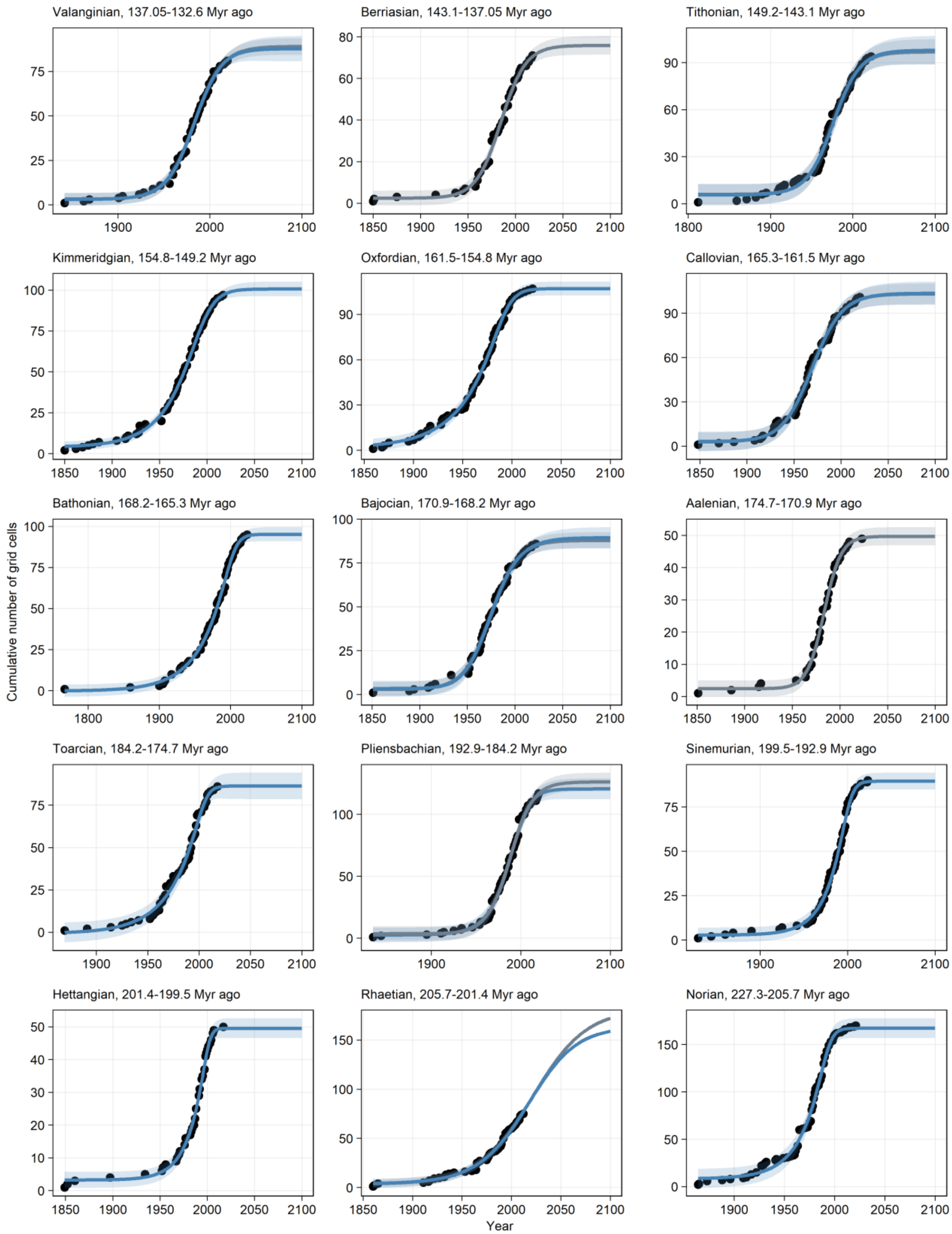

Fig. S16. Continued.

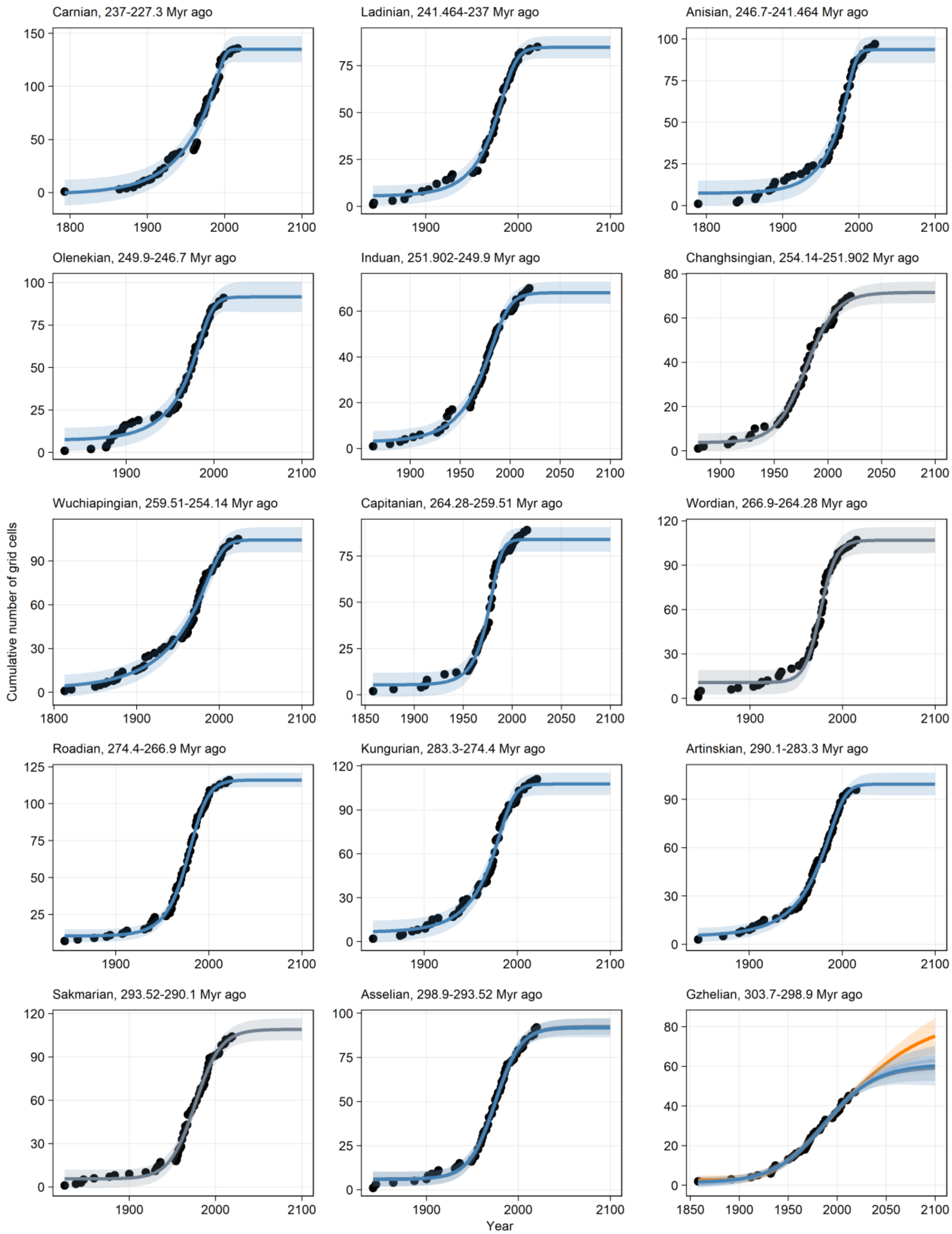

Fig. S16. Continued.

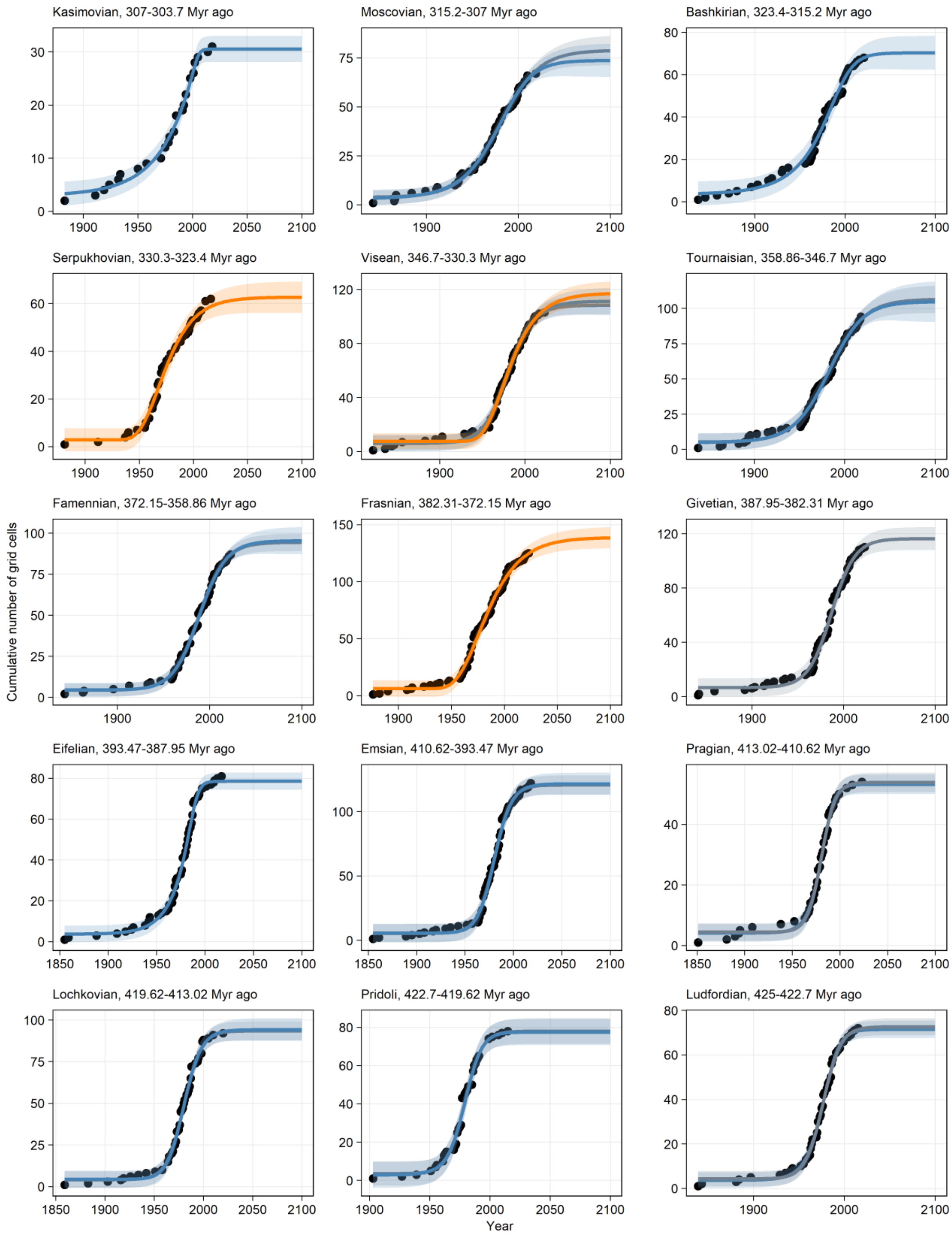

Fig. S16. Continued.

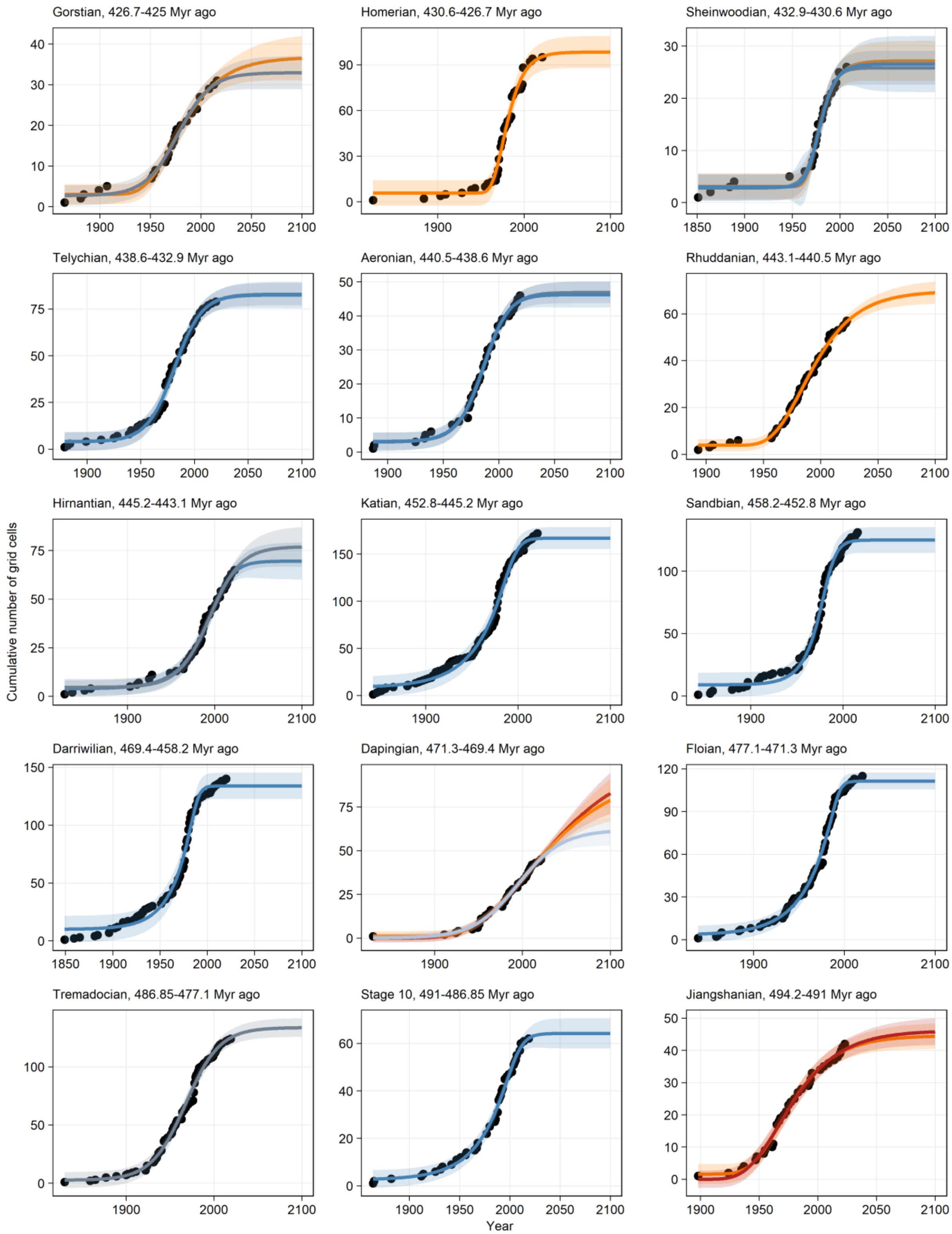

Fig. S16. Continued.

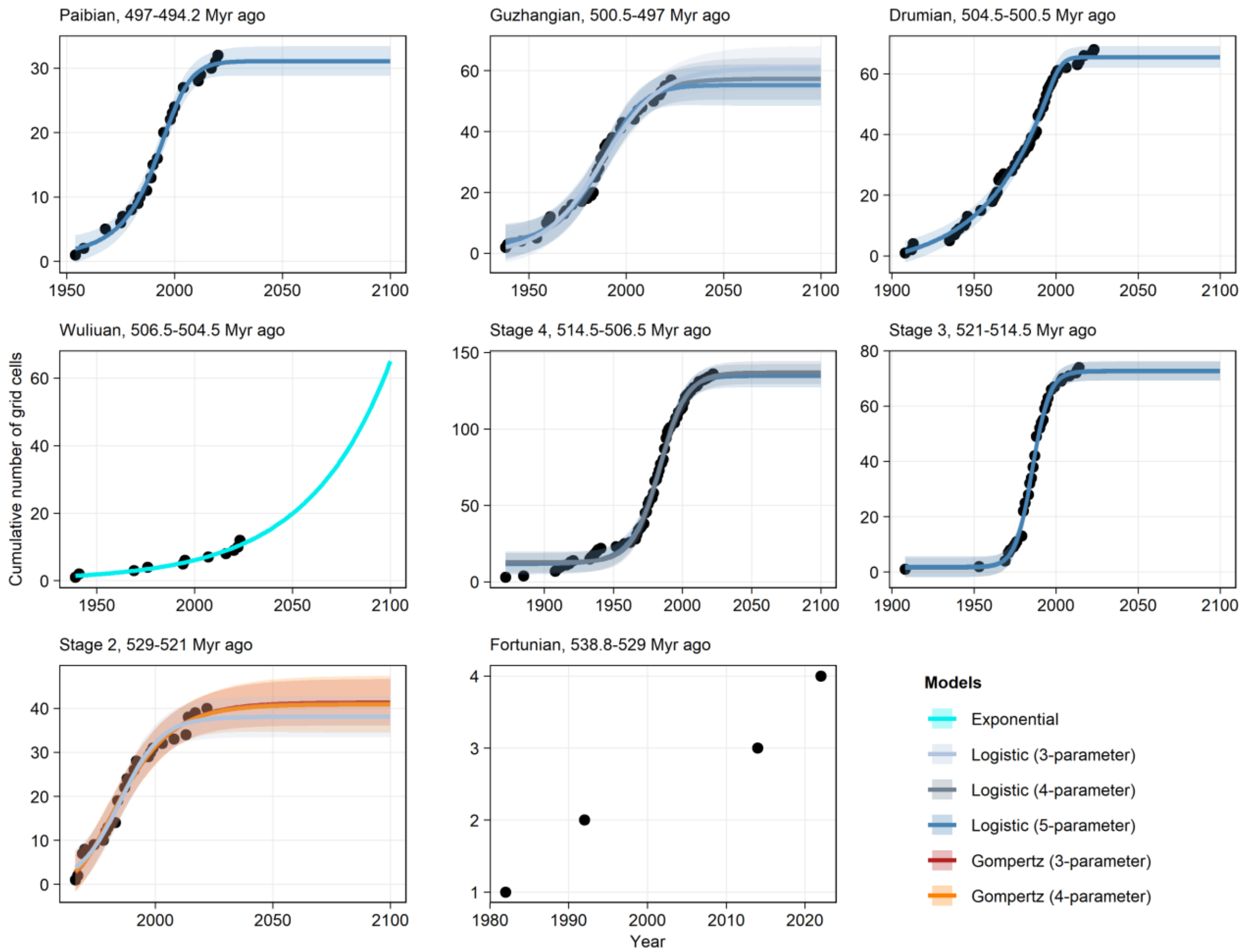

Fig. S16. Continued.

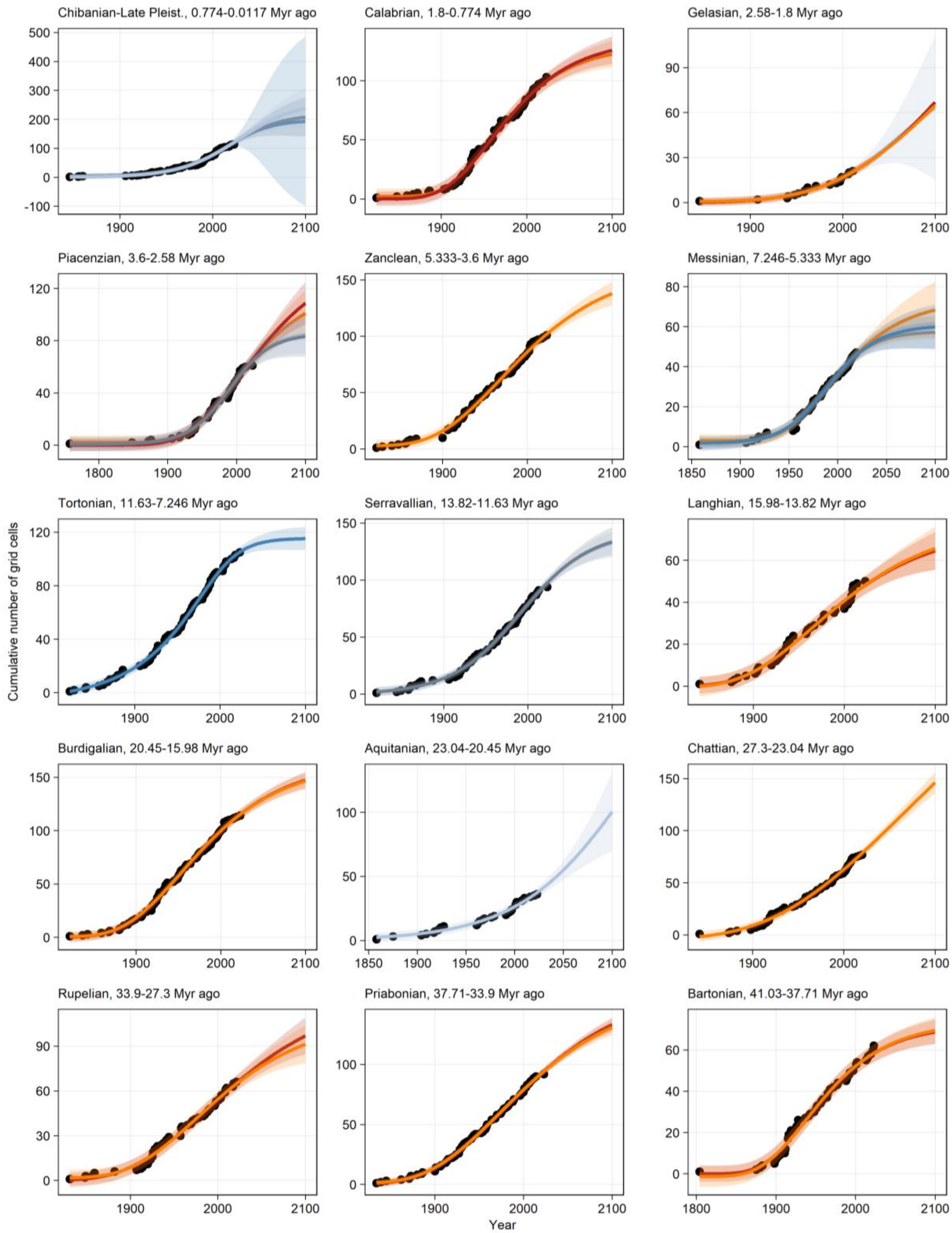

Fig. S17.

Growth models predicting future collection effort across grid cells until the year 2100 for each stage for the 750 km pointy-topped grid (all marine data, not shallow-water only). Note that for some stages, more than one model qualified as the best (i.e., within ΔAIC = 2). Underlying data is summarized in table S6.

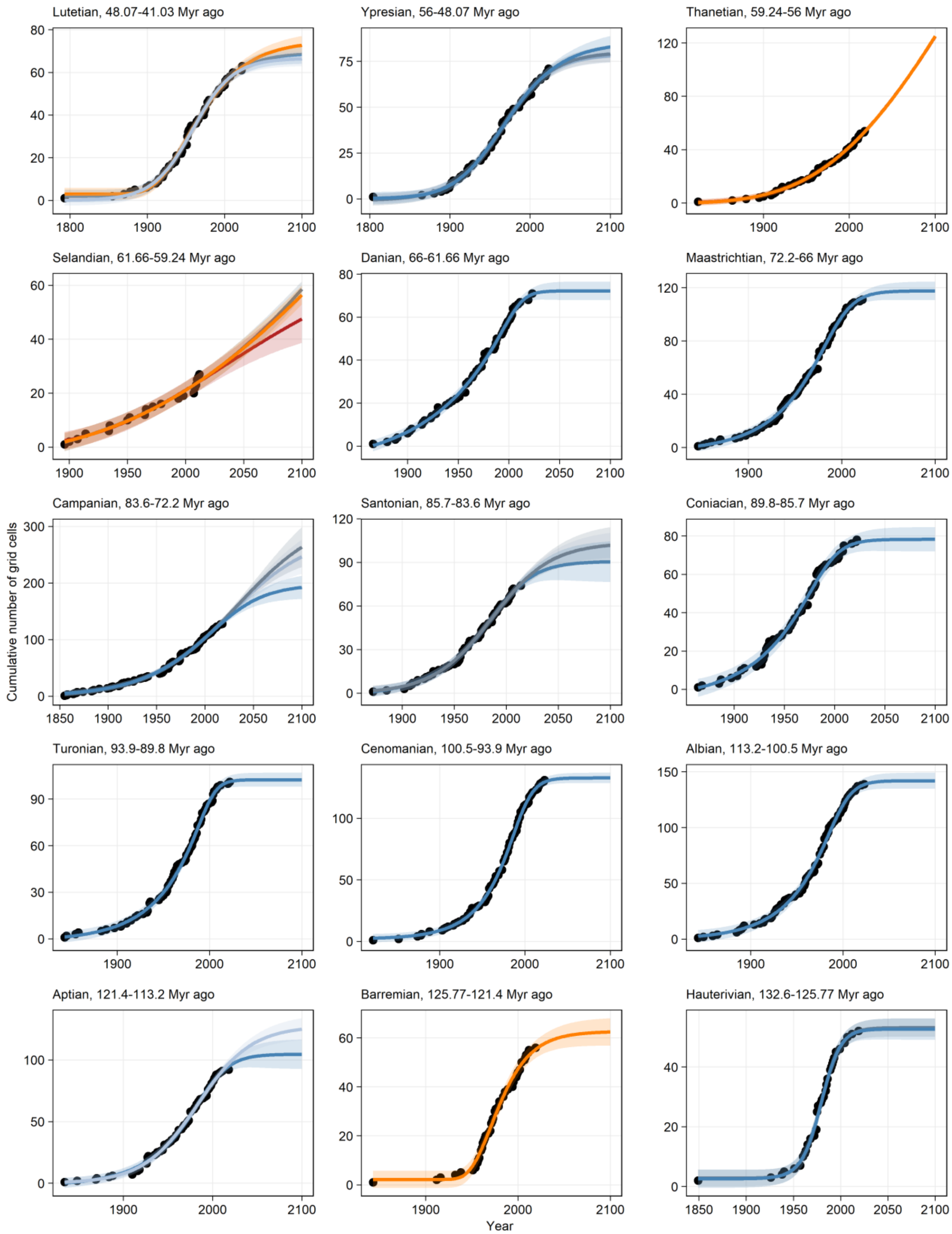

Fig. S17. Continued.

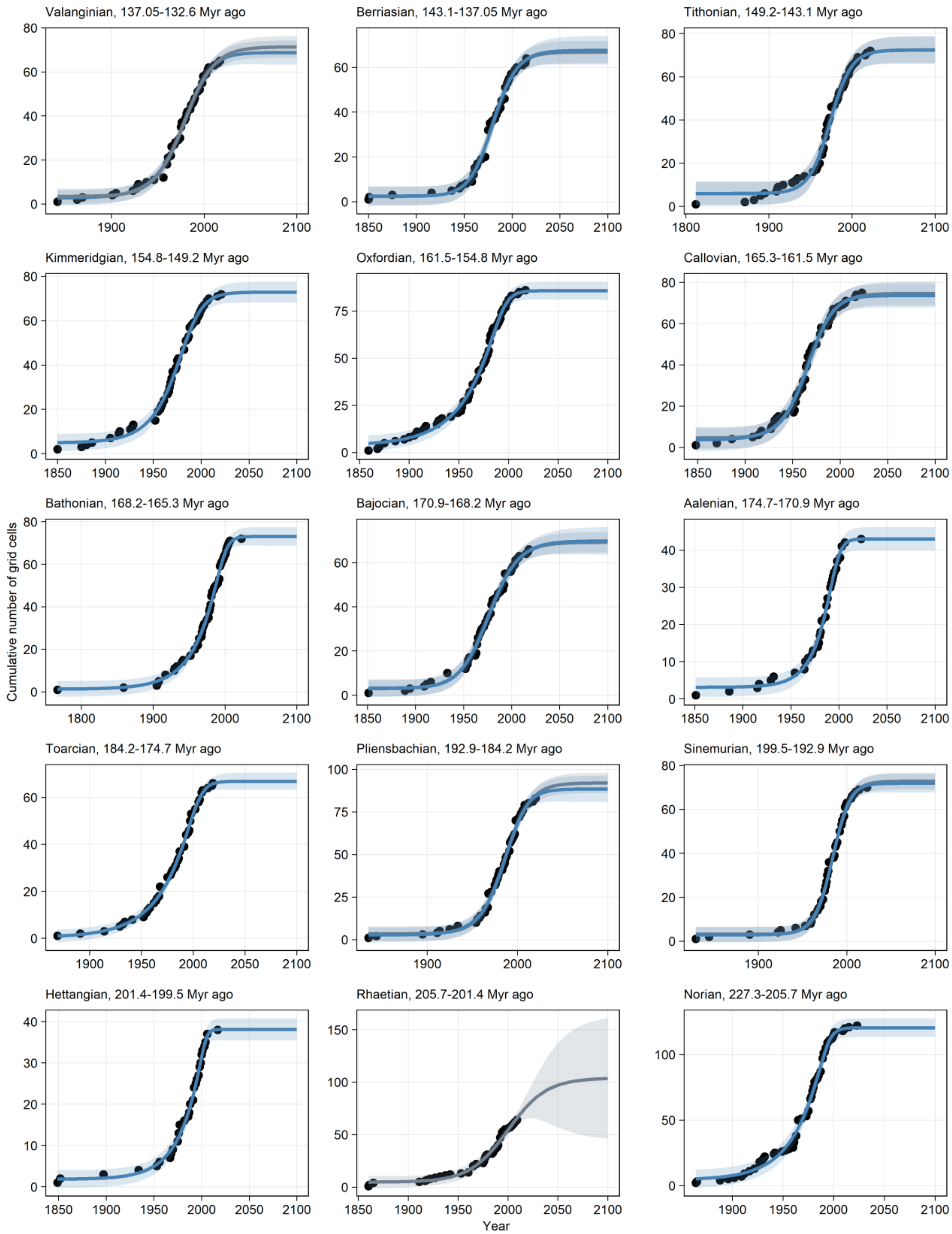

Fig. S17. Continued.

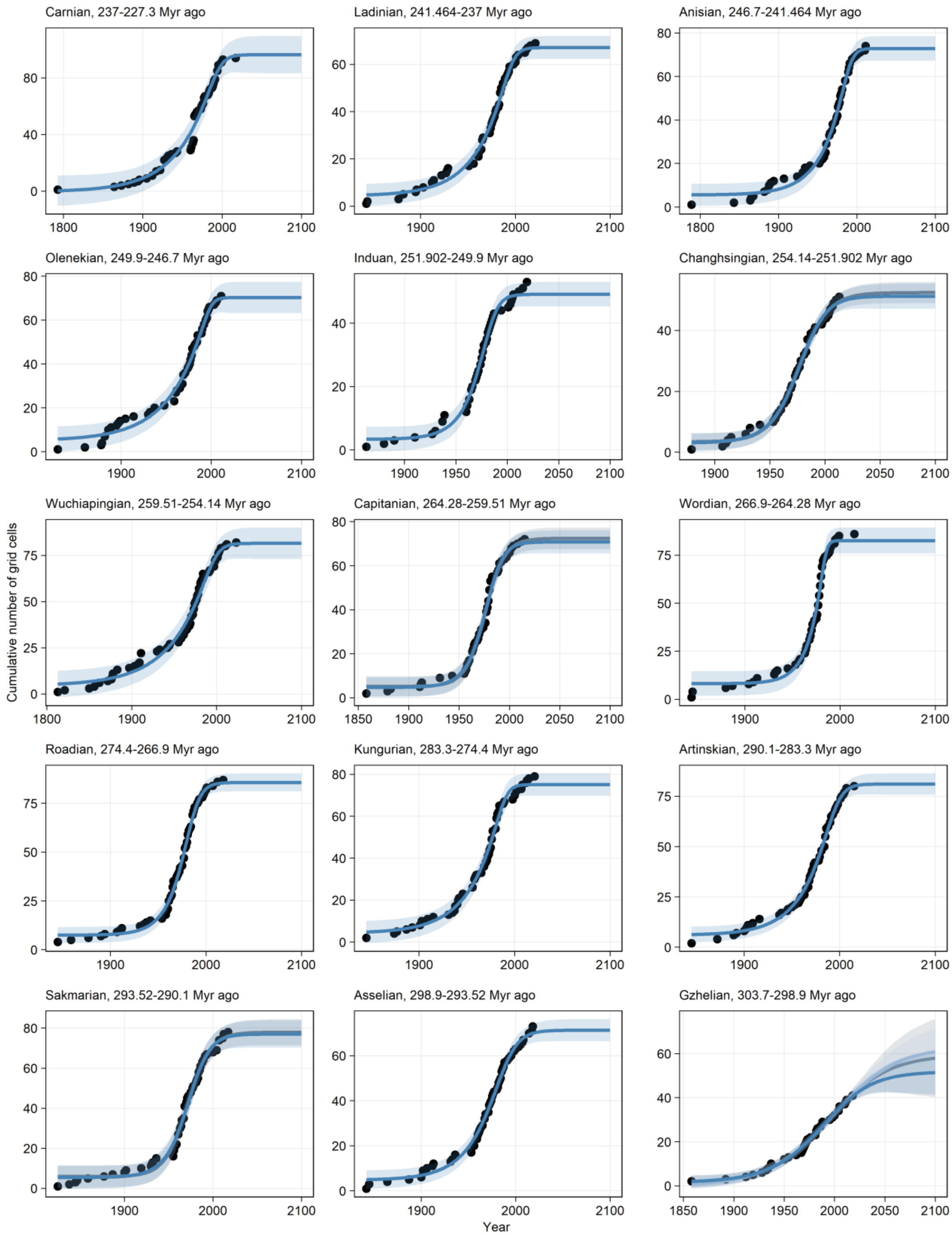

Fig. S17. Continued.

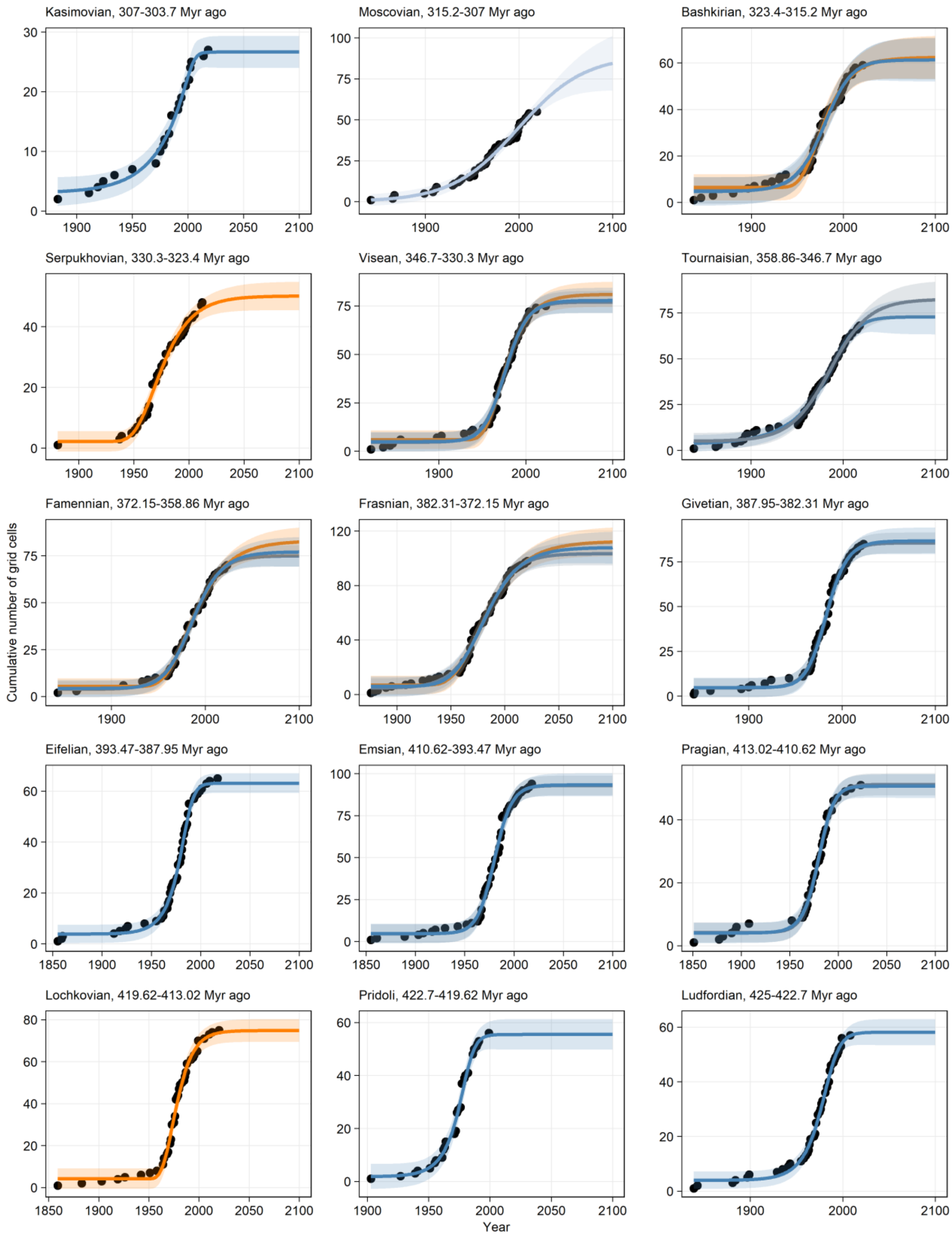

Fig. S17. Continued.

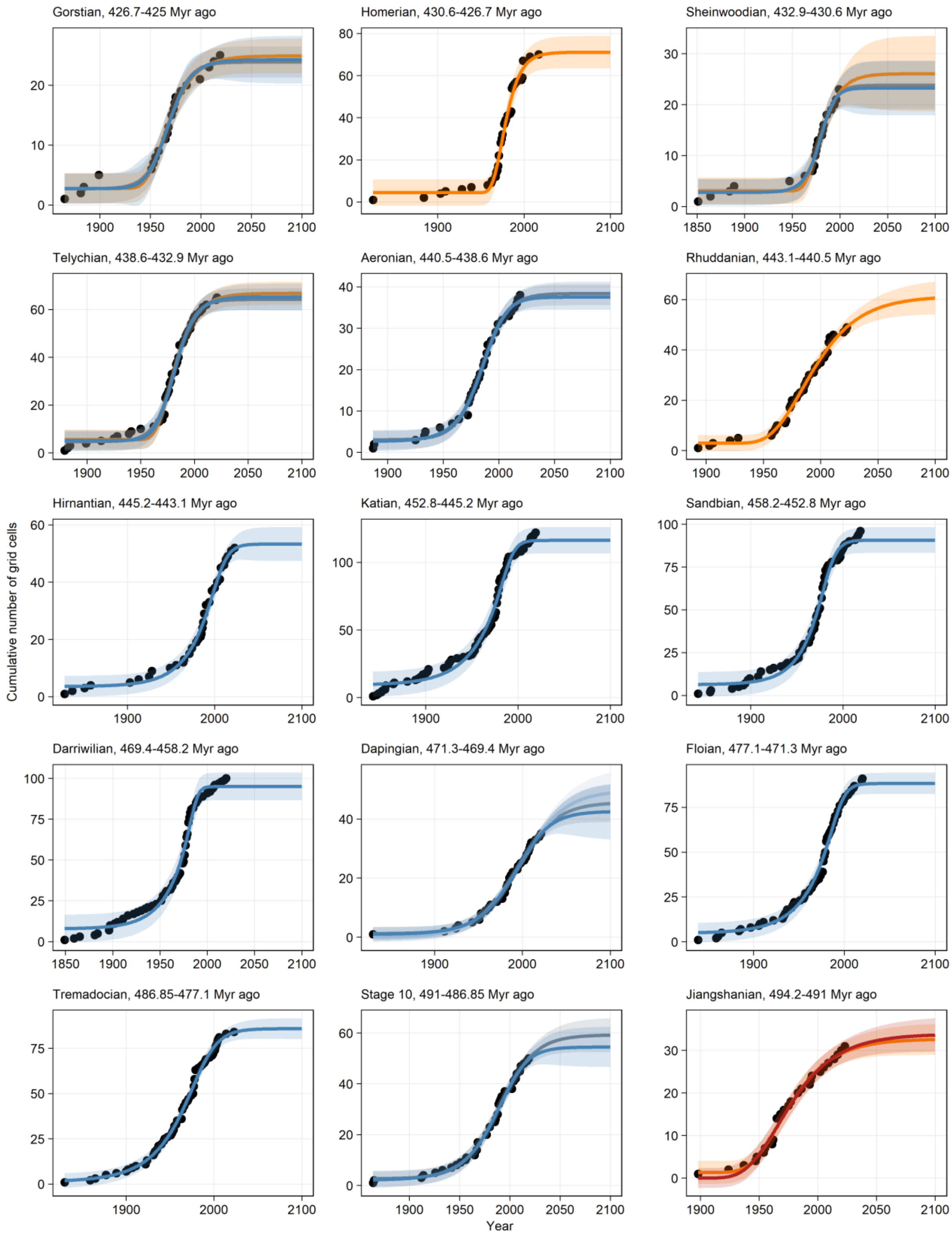

Fig. S17. Continued.

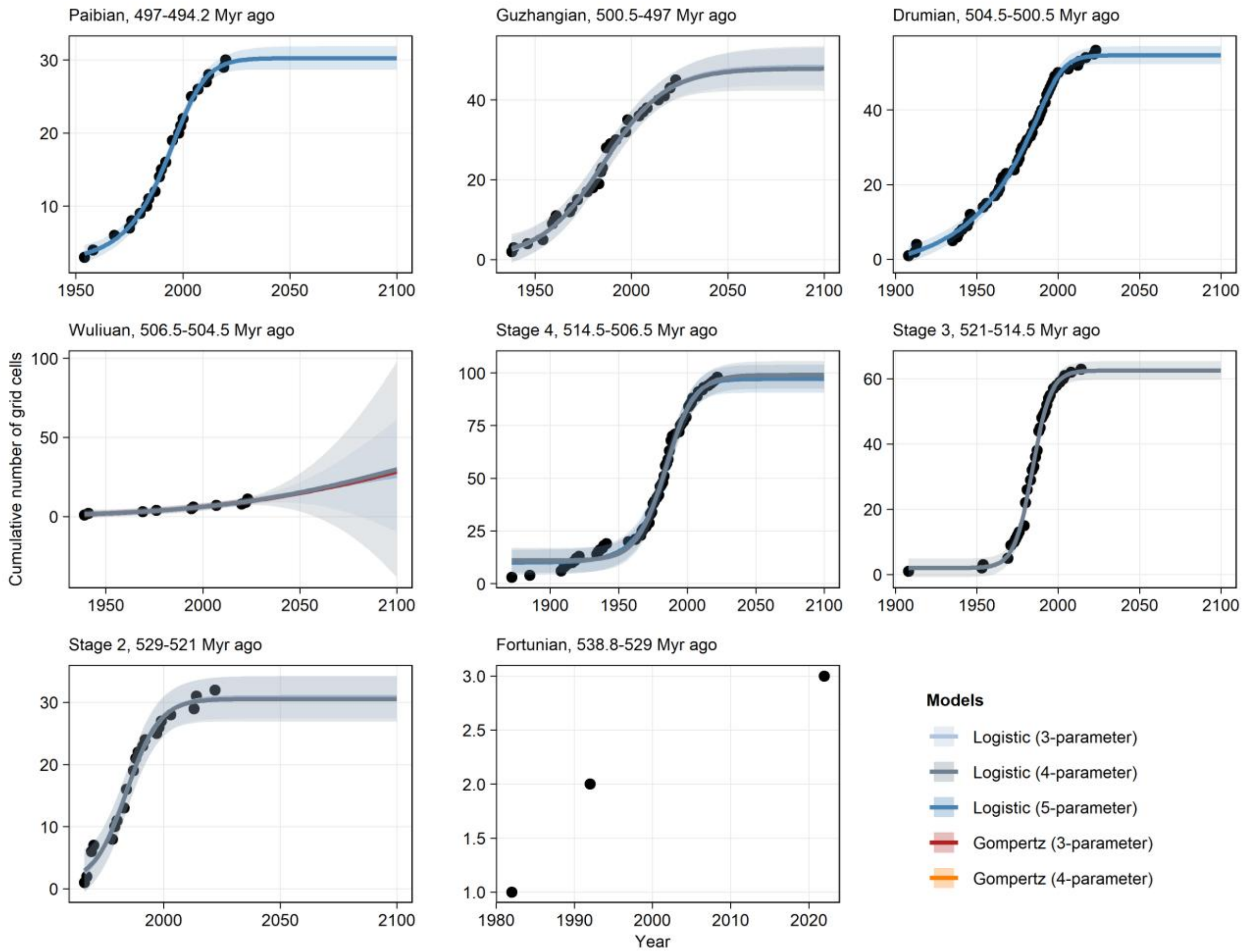

Fig. S17. Continued.

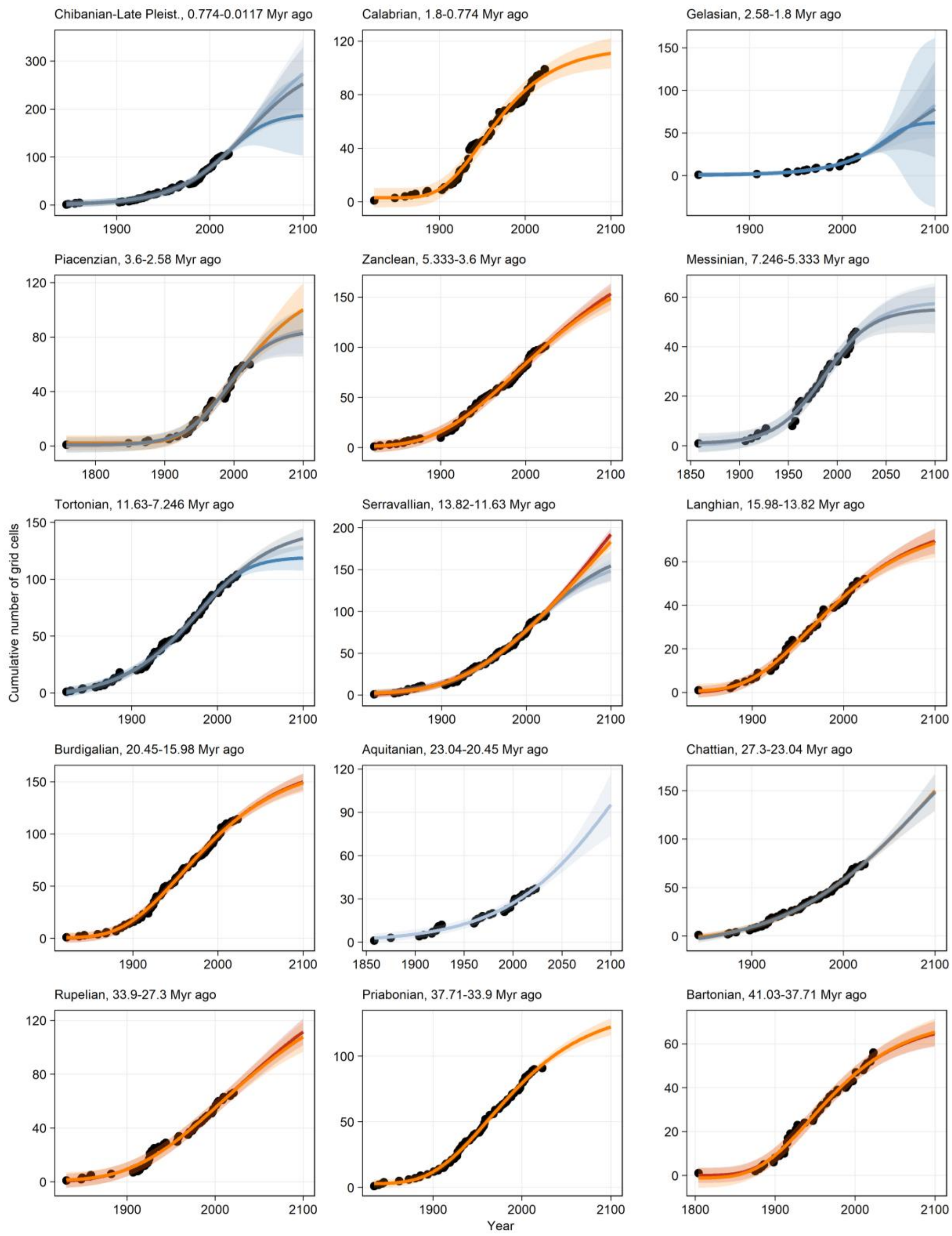

Fig. S18.

Growth models predicting future collection effort across grid cells until the year 2100 for each stage for the 750 km flat-topped grid (all marine data, not shallow-water only). Note that for some stages, more than one model qualified as the best (i.e., within ΔAIC = 2). Underlying data is summarized in table S6.

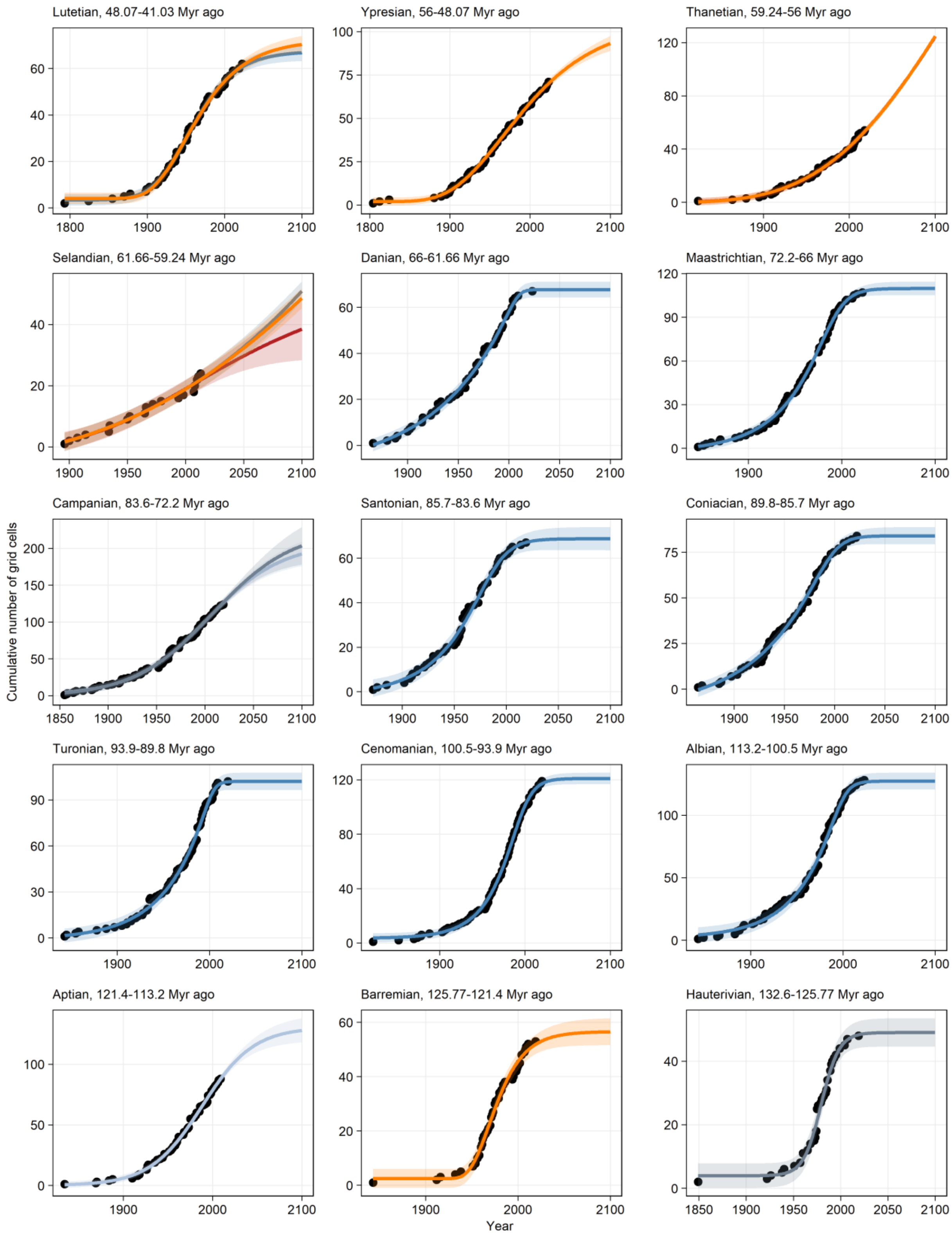

Fig. S18. Continued.

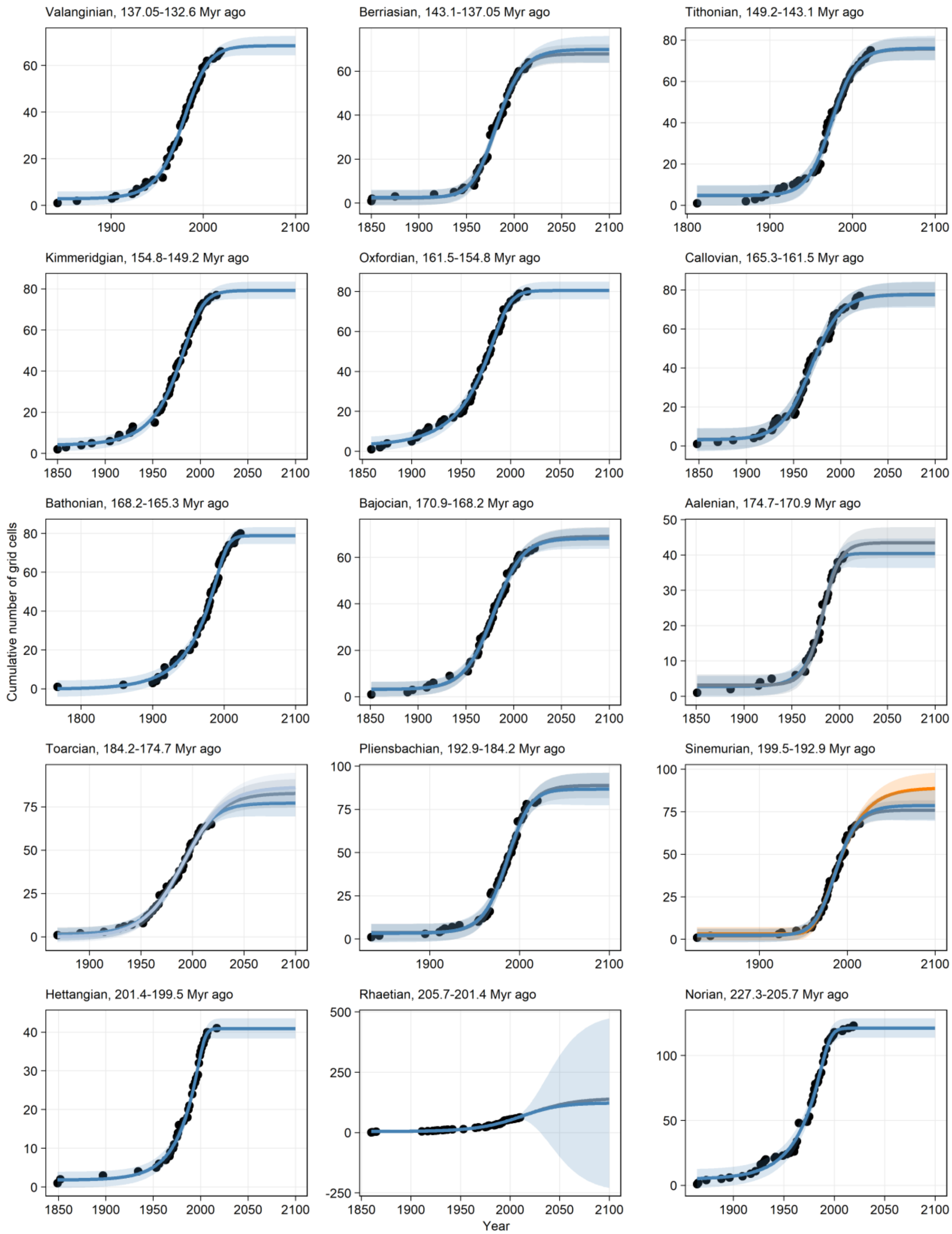

Fig. S18. Continued.

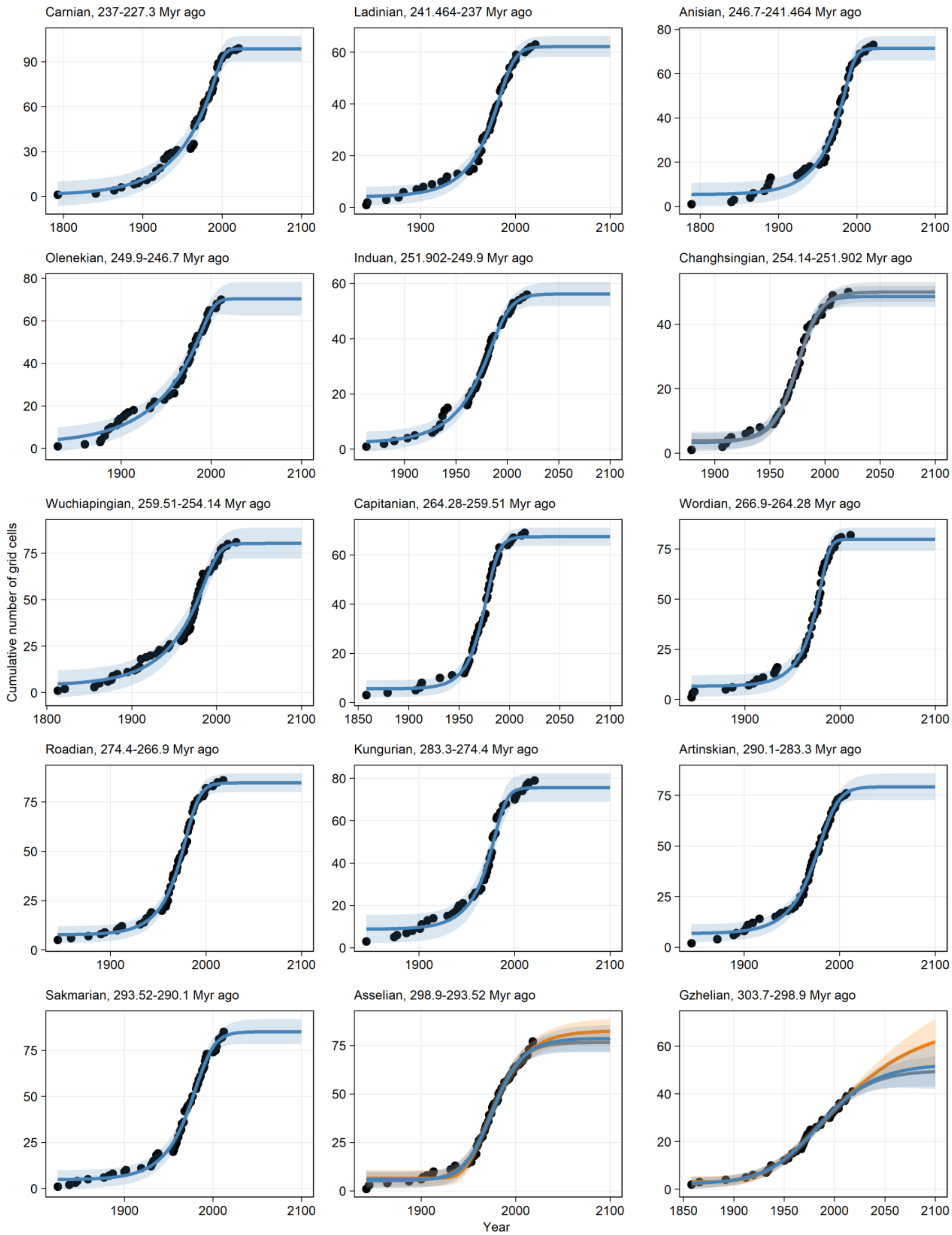

Fig. S18. Continued.

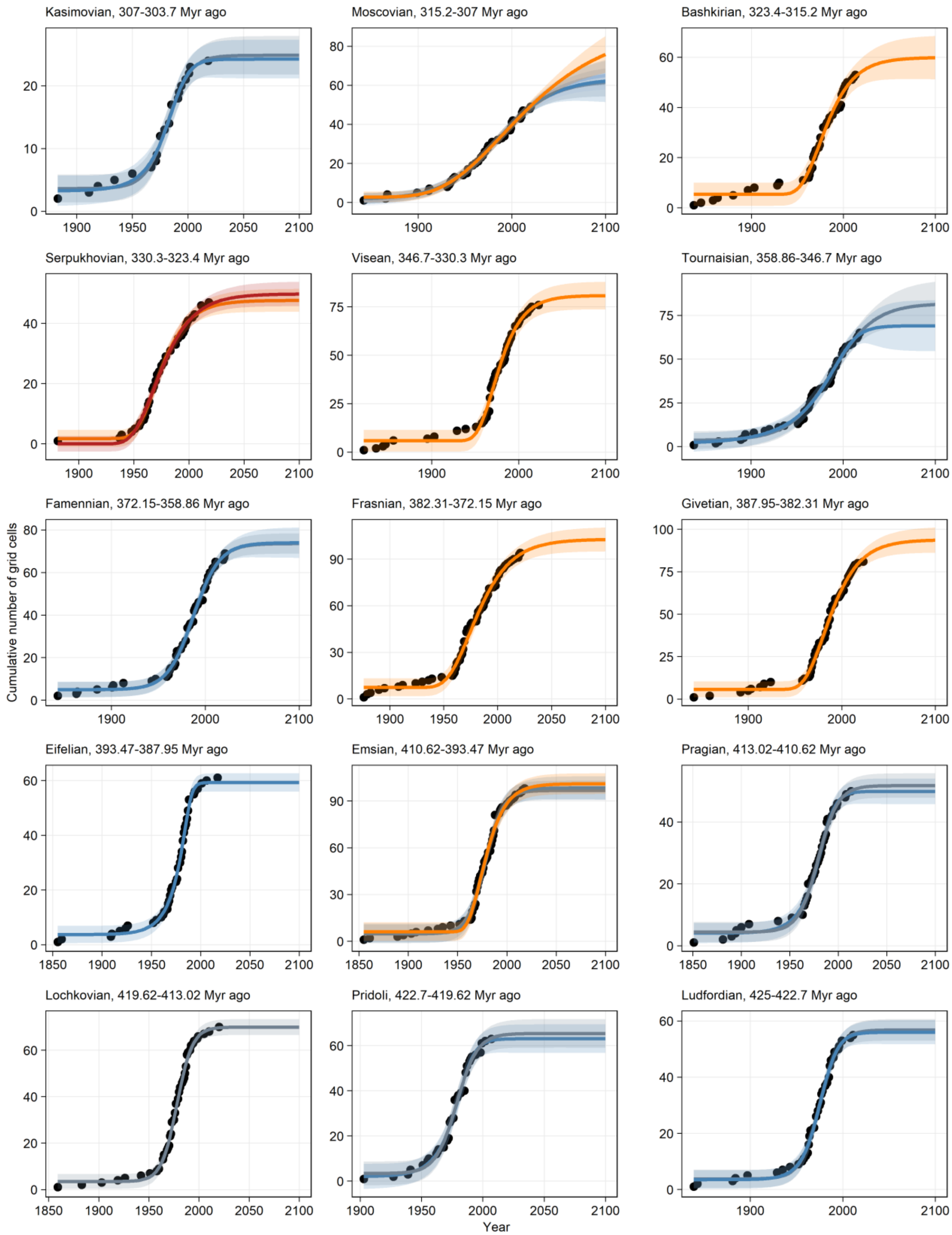

Fig. S18. Continued.

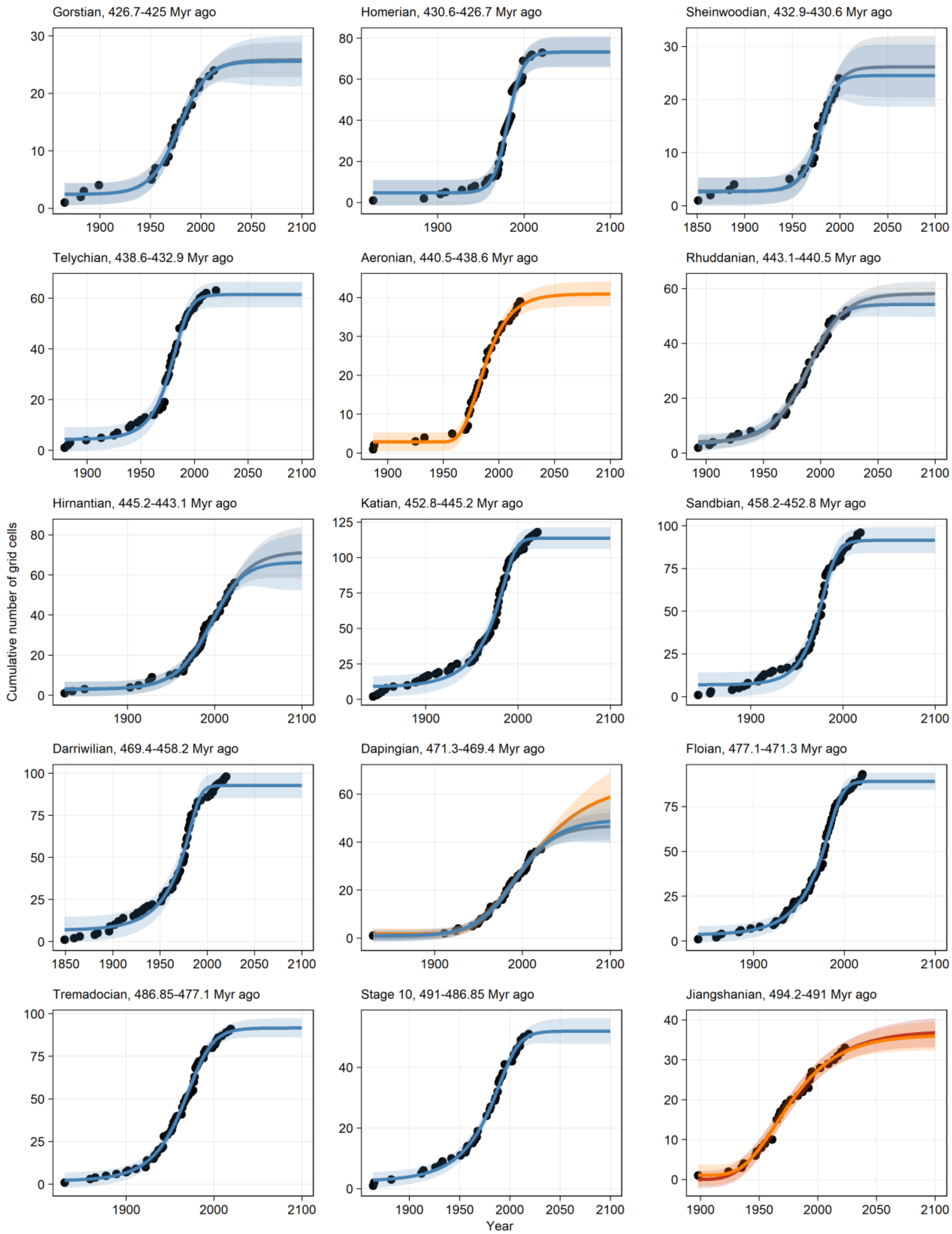

Fig. S18. Continued.

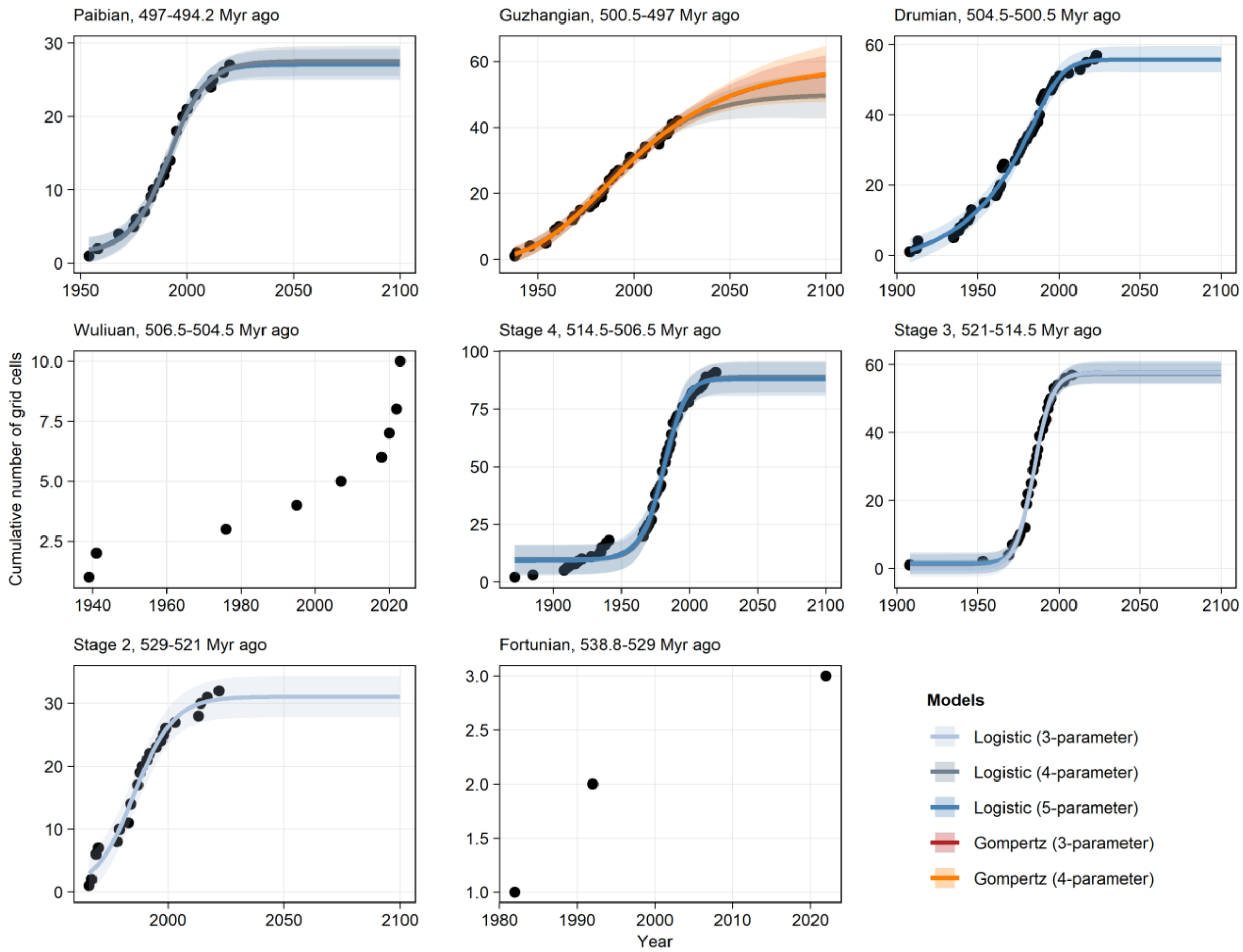

Fig. S18. Continued.

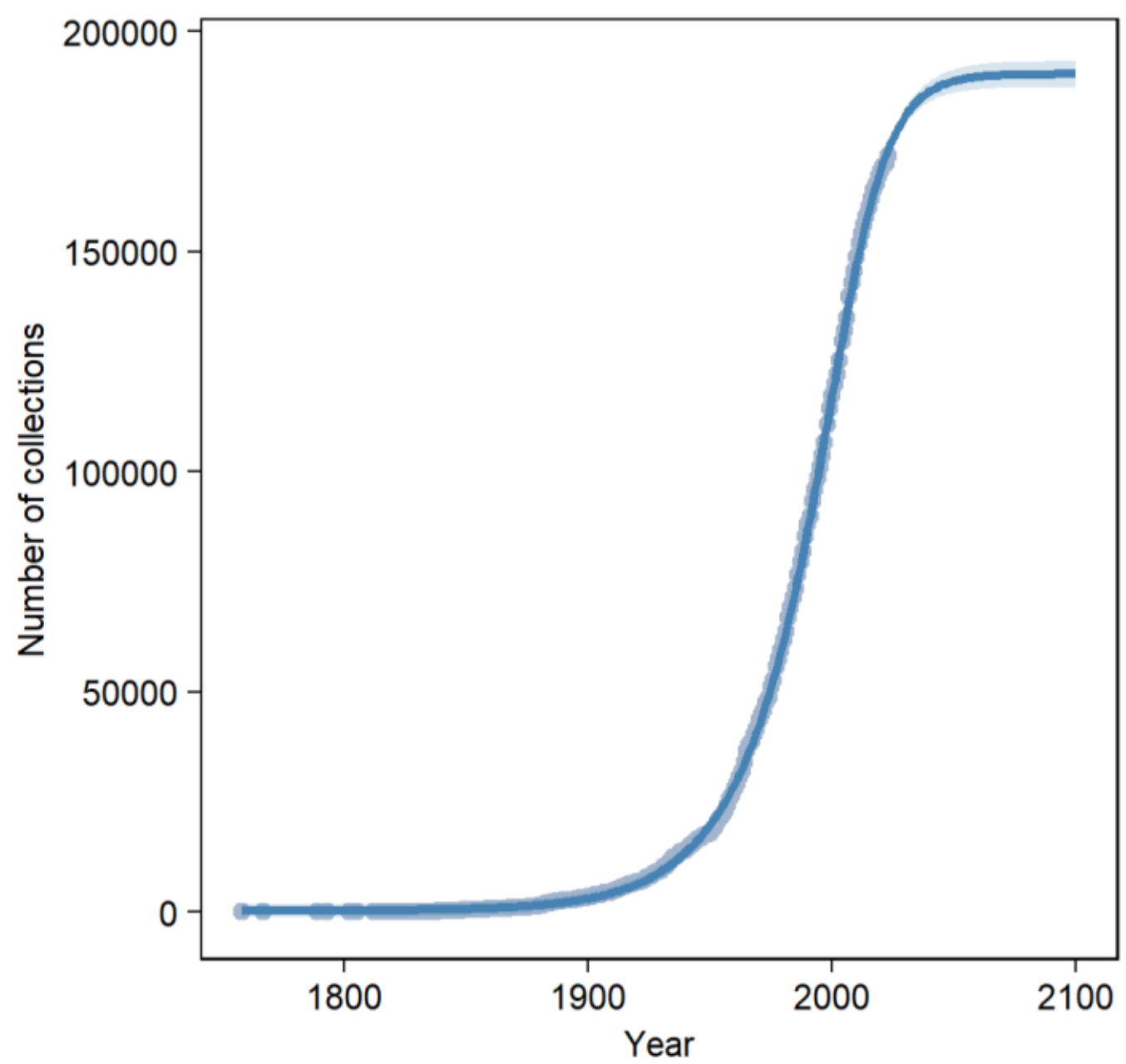

Fig. S19.

Total historical collection effort across all marine assemblages, irrespective of grid or stage. If trends persist, saturation is expected by the mid-21st century. The best model with lowest AIC is the 5-parameter logistic model (AIC = 3435.2, compared to AIC = 3709.2 for the second-best option, the 4-parameter logistic model).

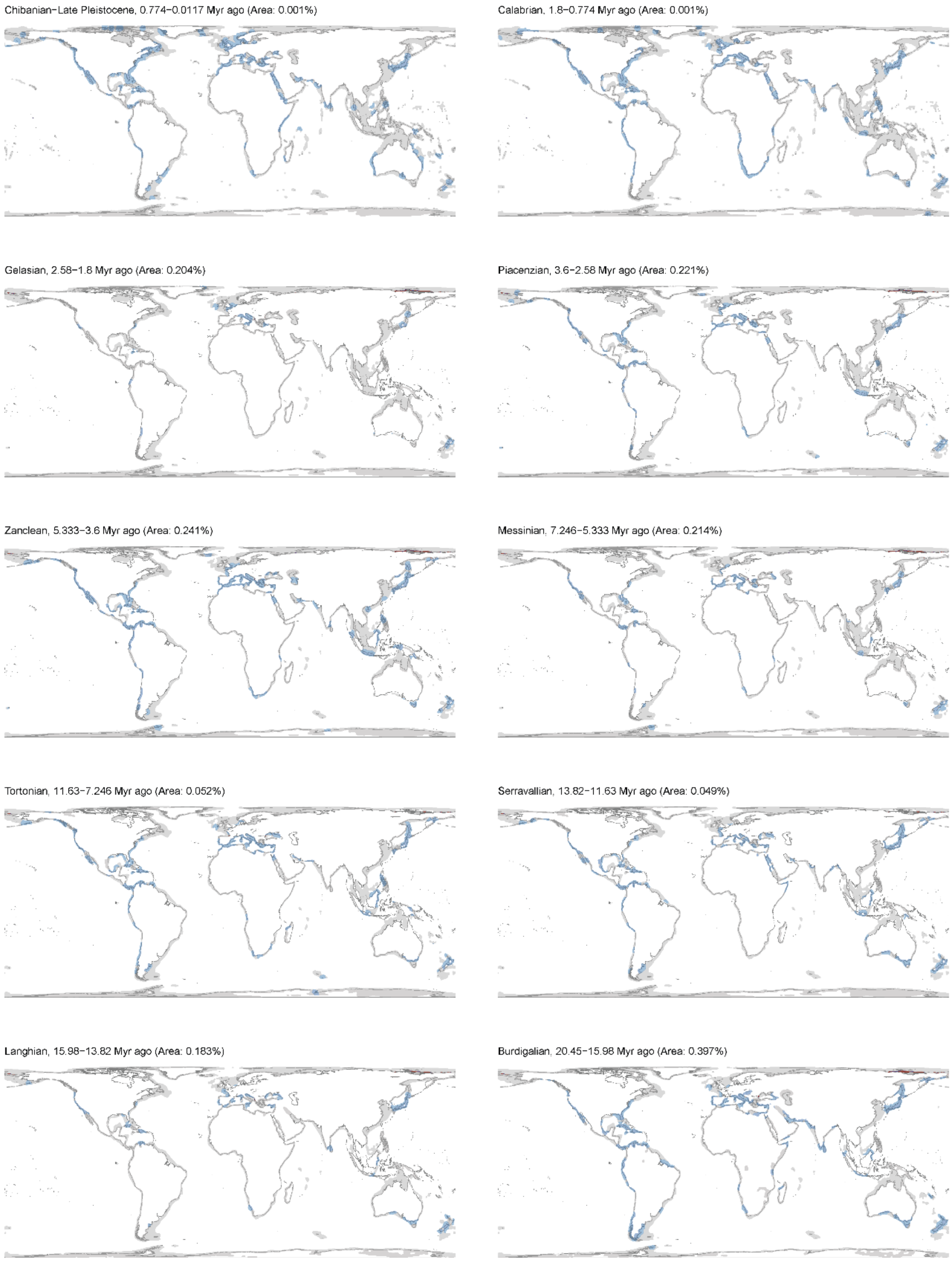

Fig. S20.

Reconstructed extents of past shallow-water areas and corresponding unexplored deposits for each stage. Data on deposits were retrieved from Macrostrat Database, applying the strict filter (see Methods). Relevant areas are summarized in tables S7–8.

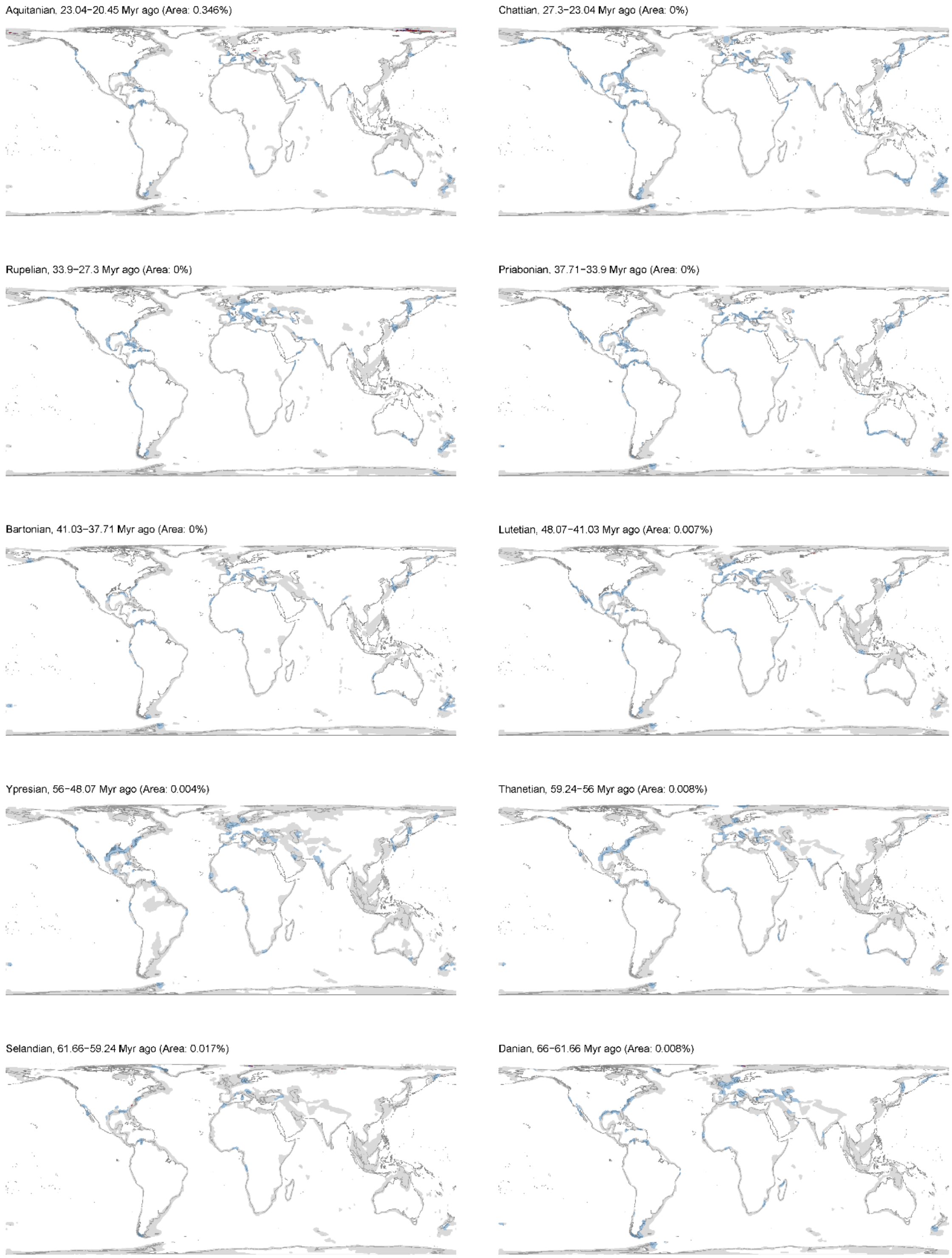

Fig. S20. Continued.

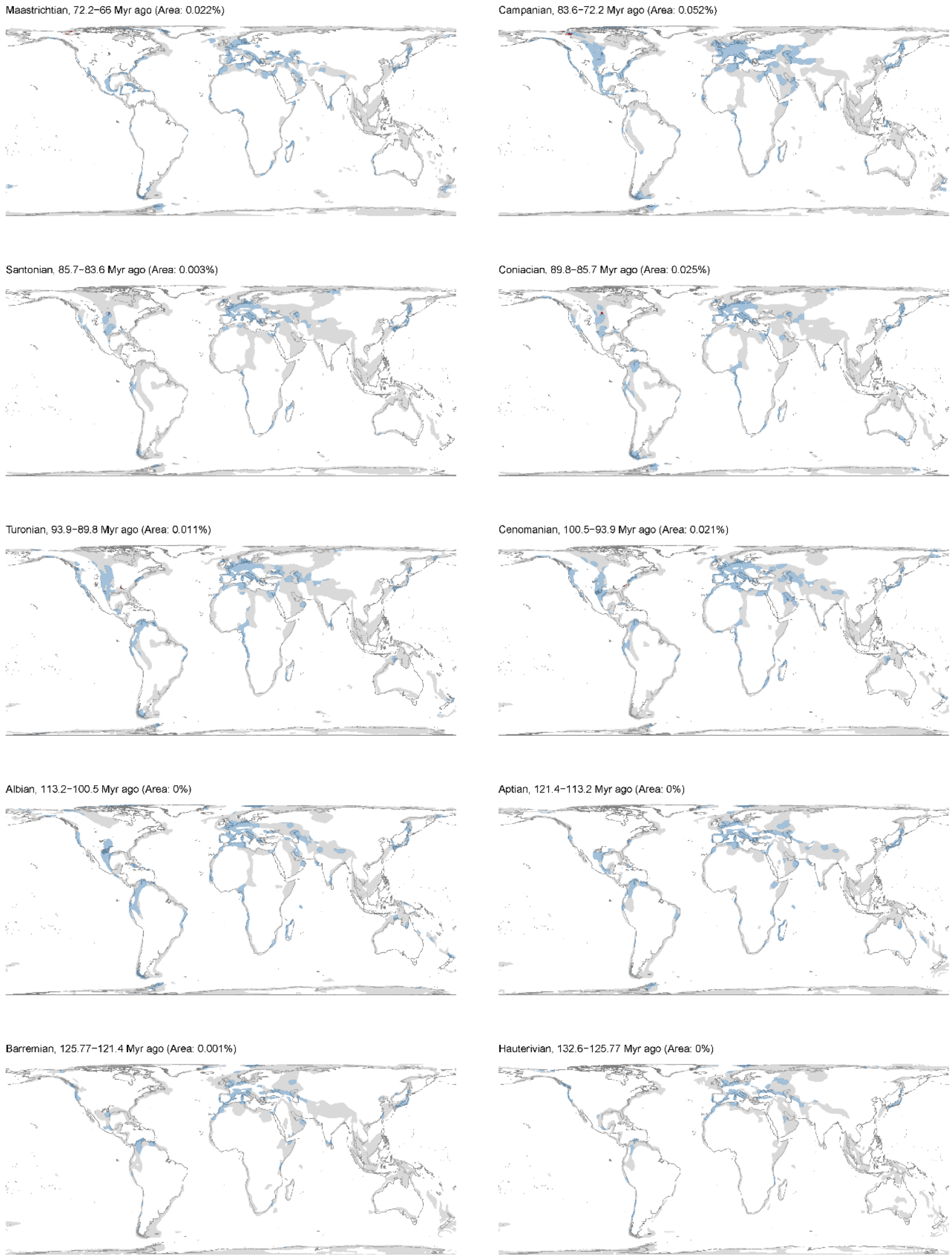

Fig. S20. Continued.

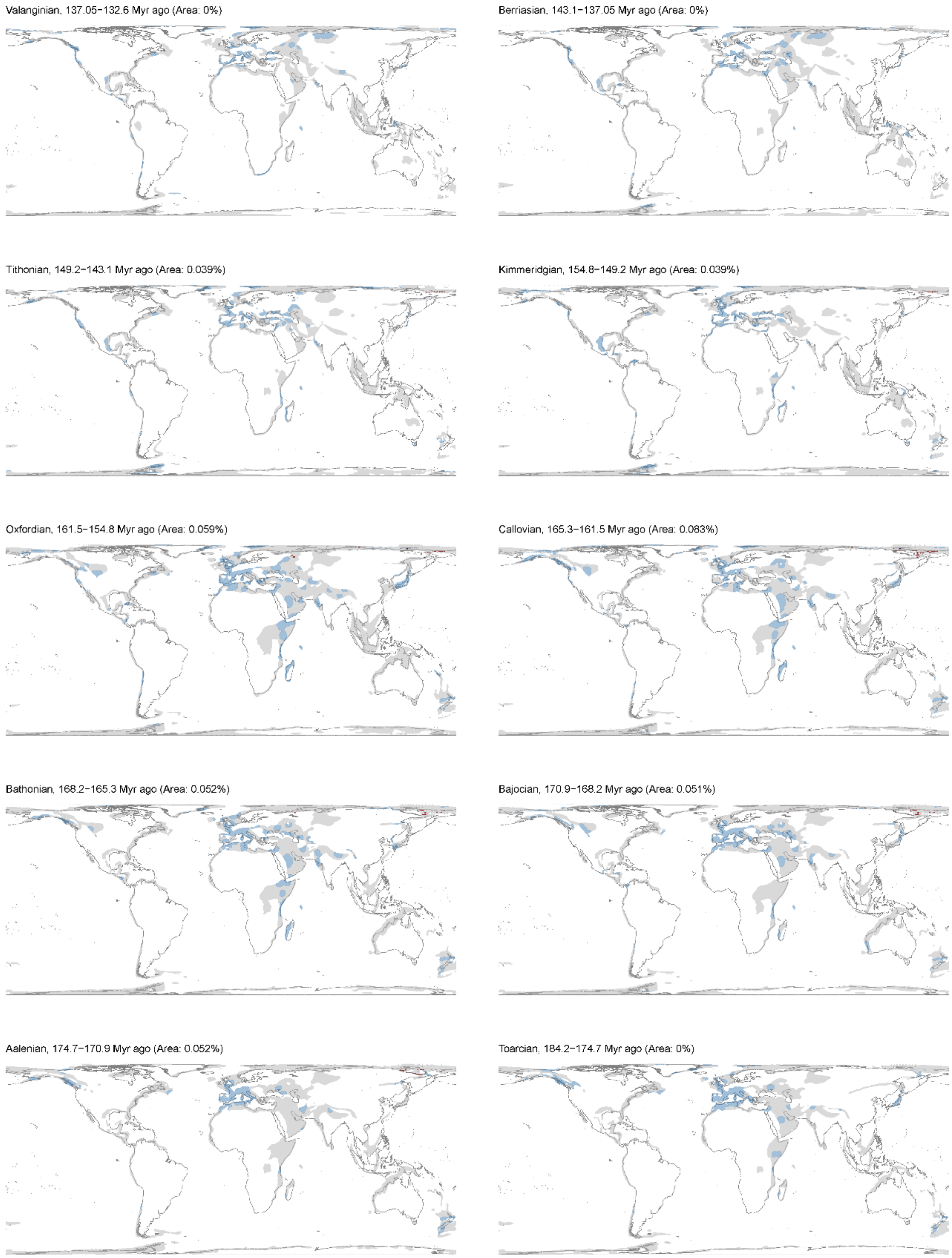

Fig. S20. Continued.

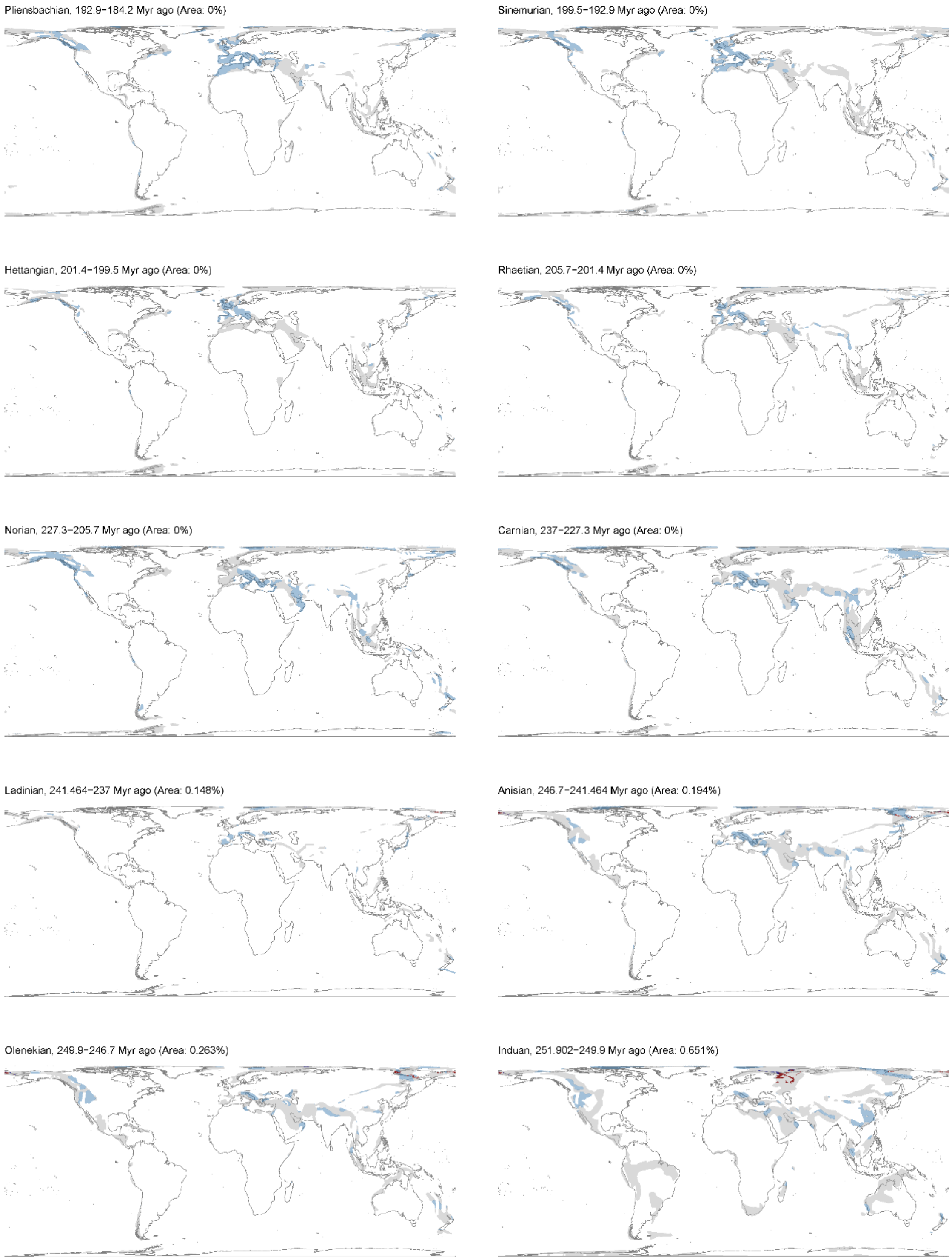

Fig. S20. Continued.

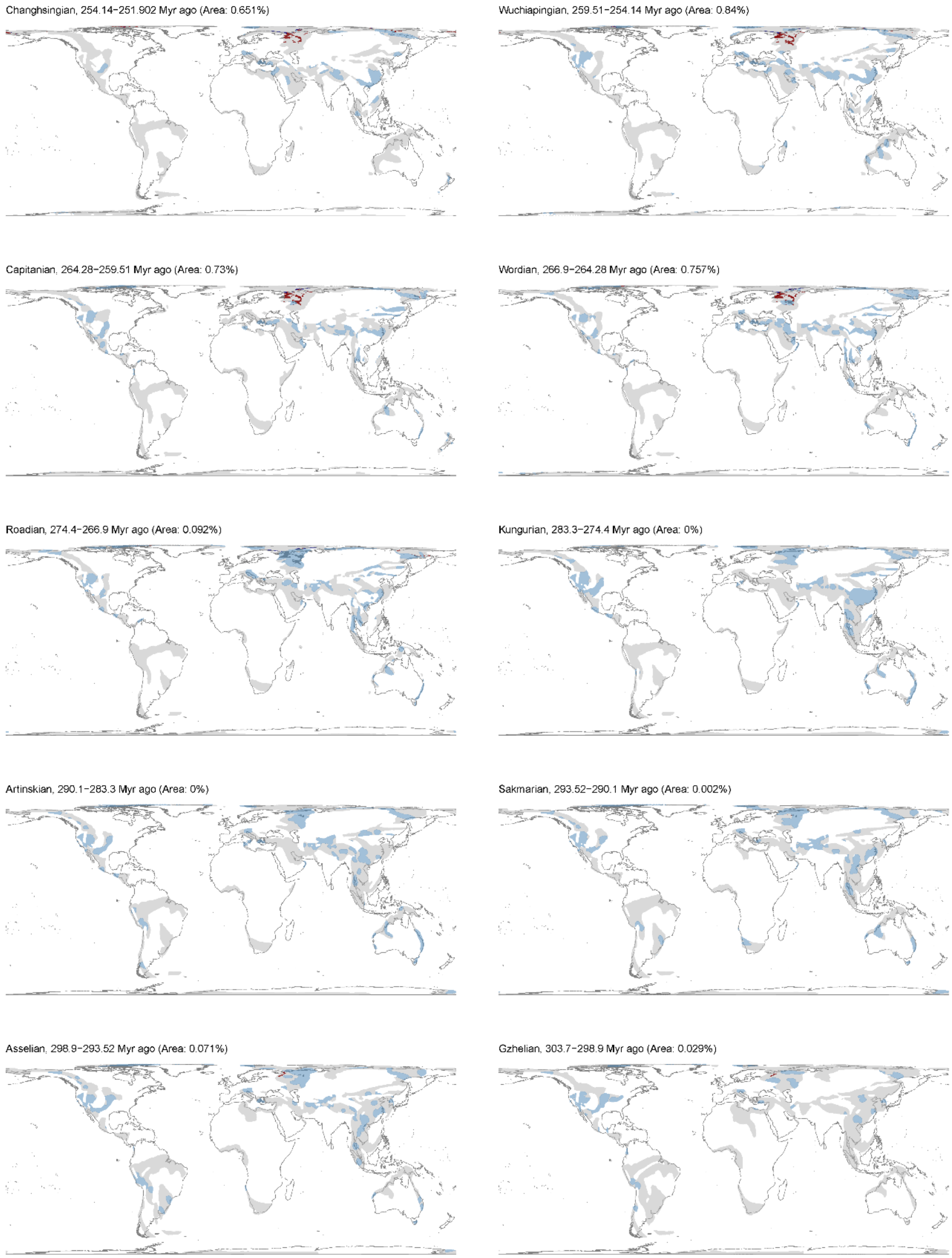

Fig. S20. Continued.

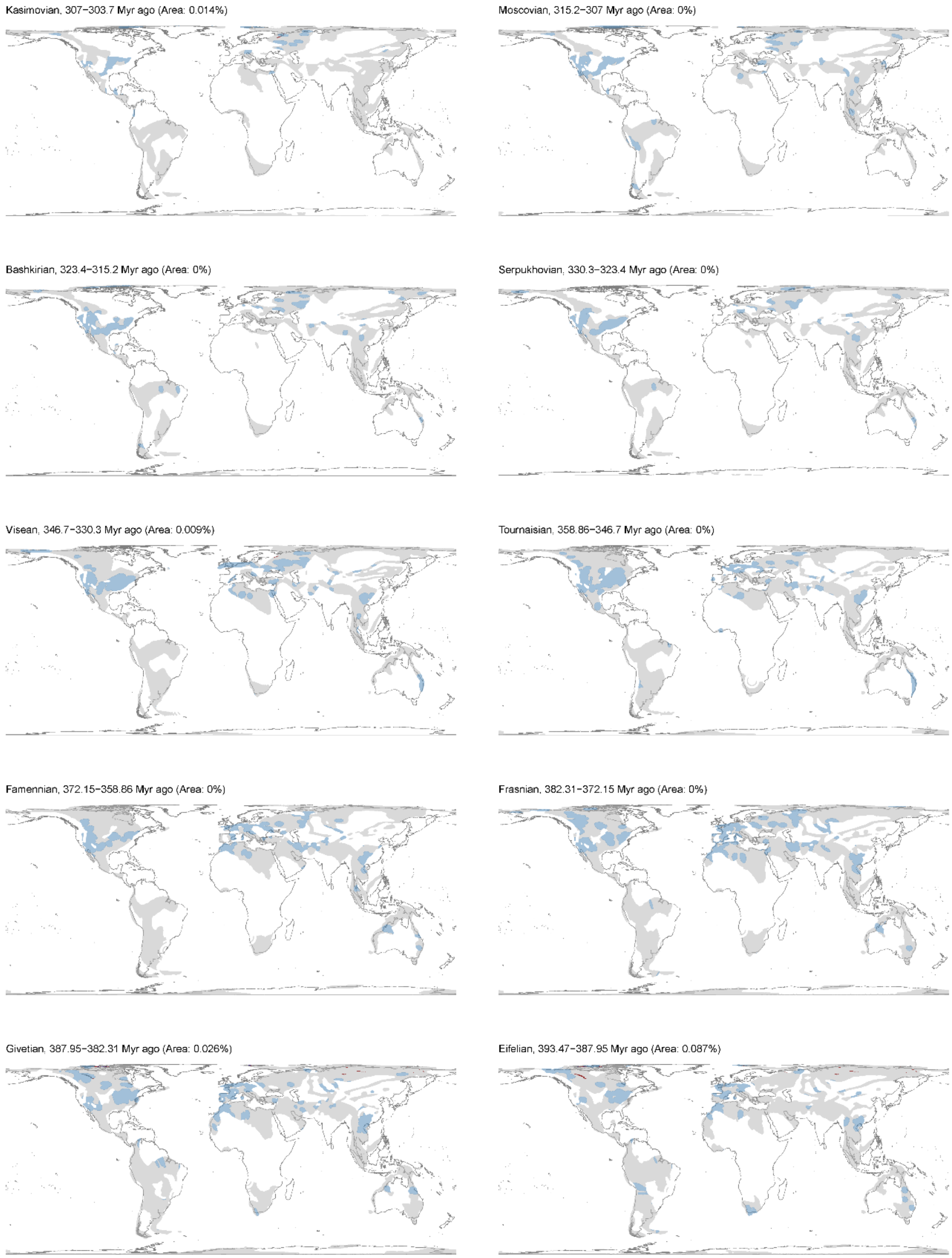

Fig. S20. Continued.

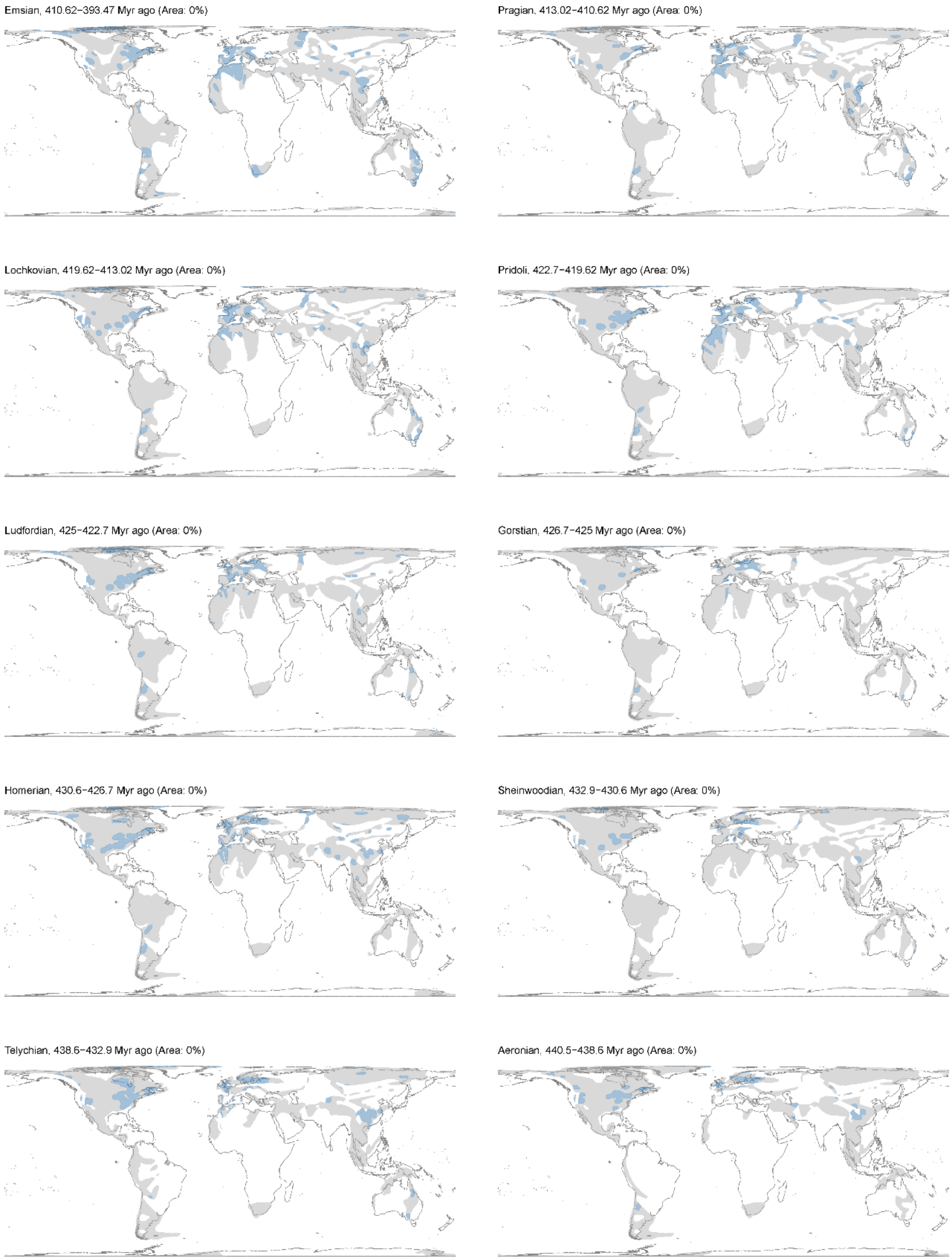

Fig. S20. Continued.

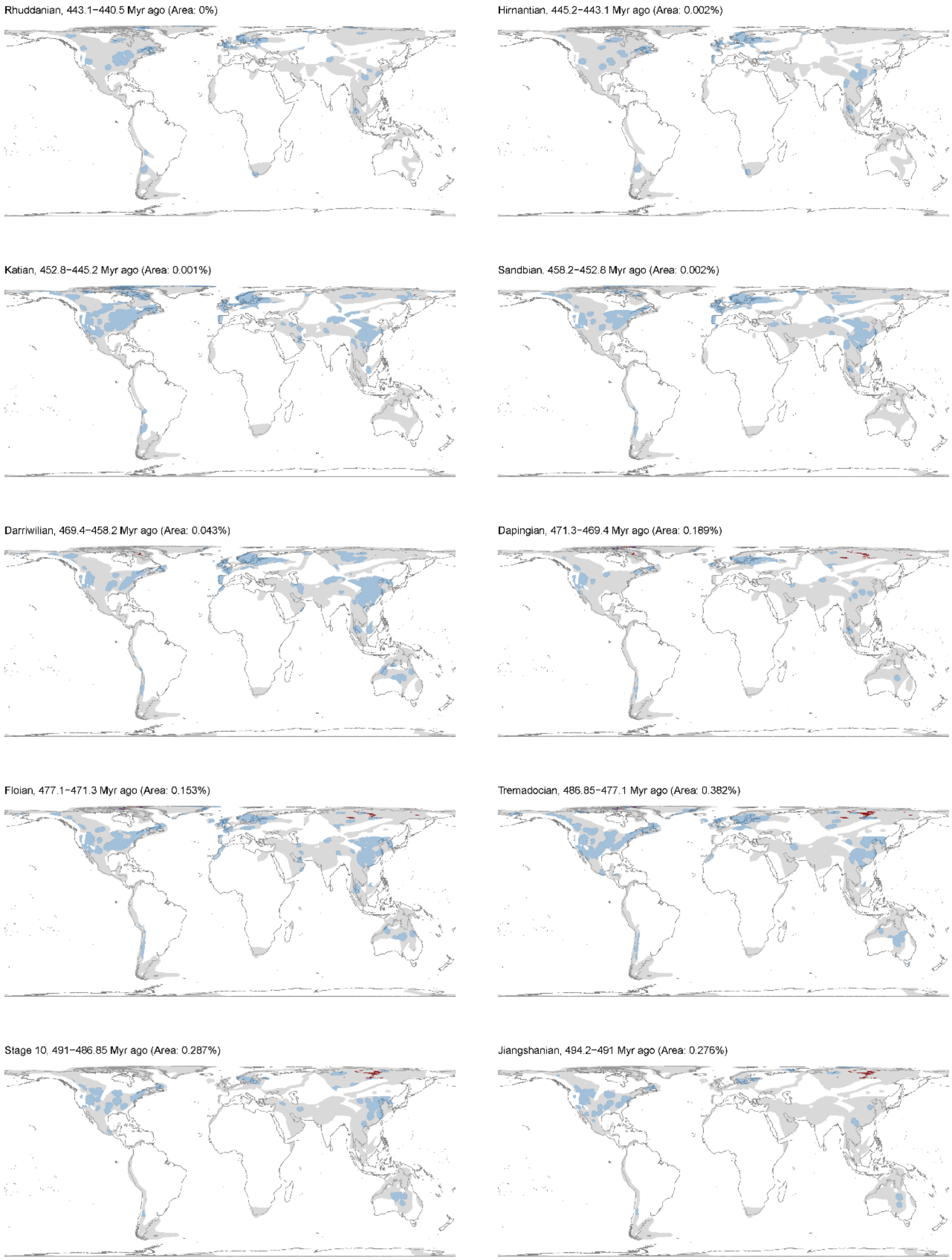

Fig. S20. Continued.

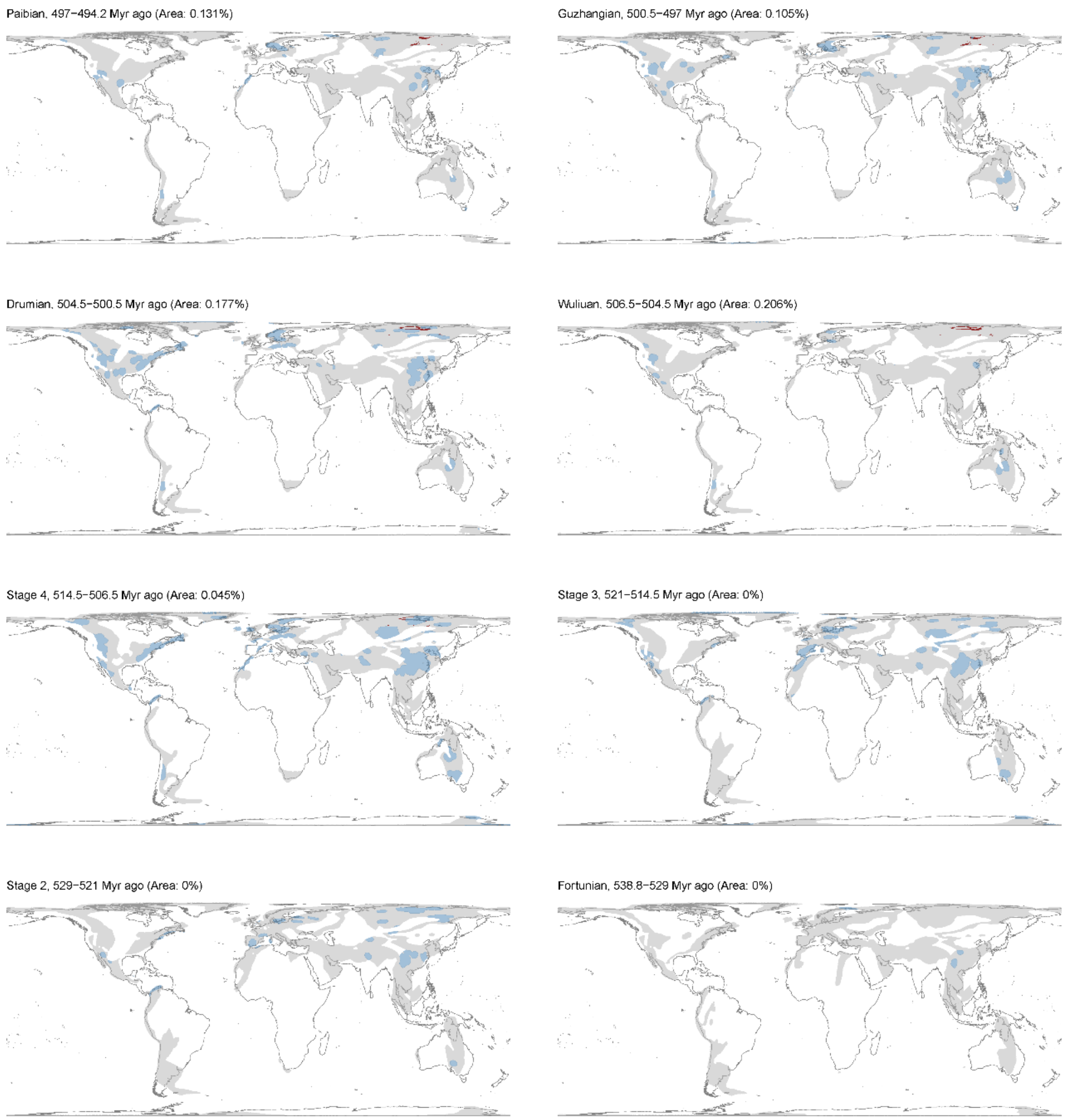

Fig. S20. Continued.

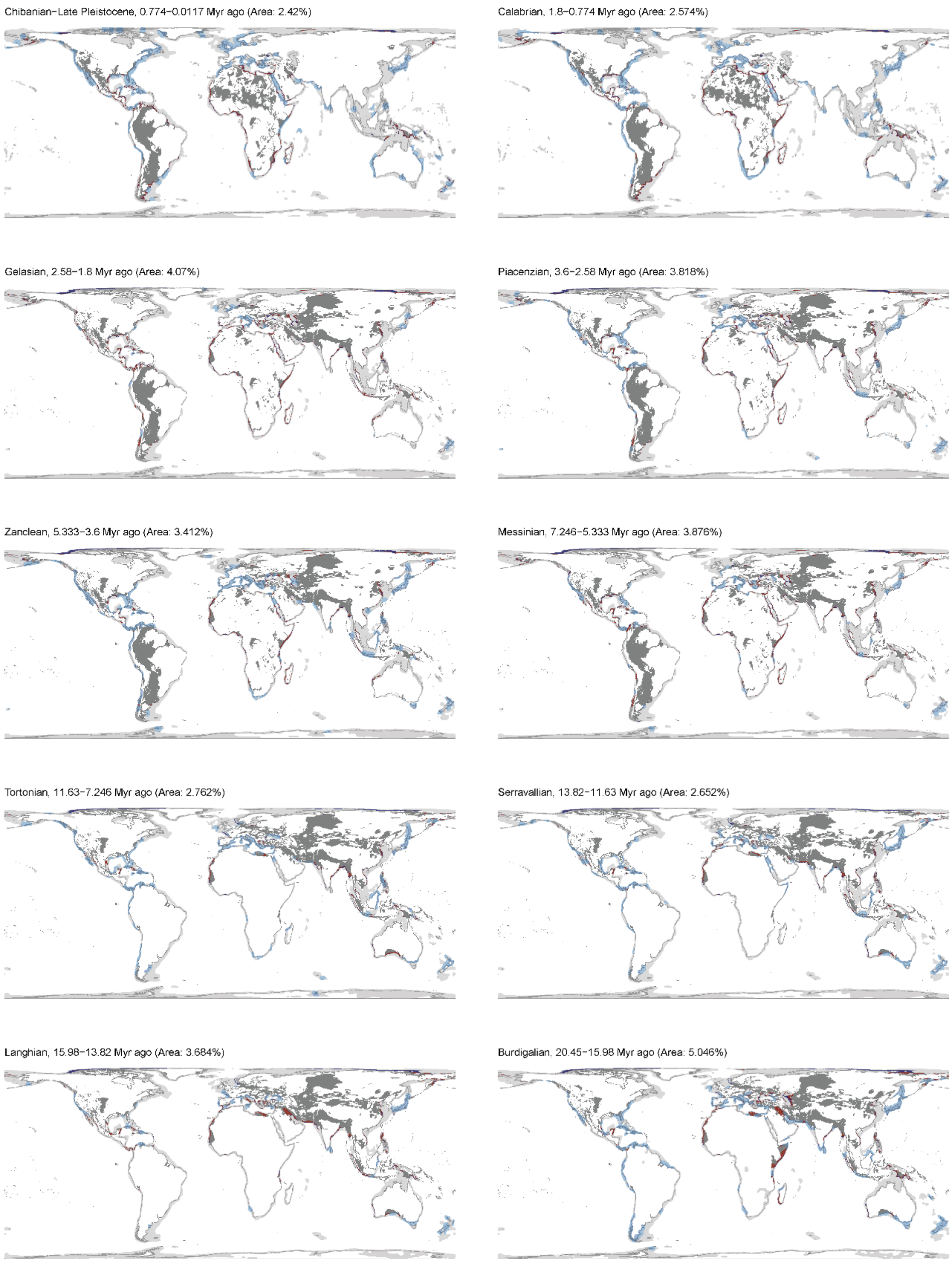

Fig. S21.

Reconstructed extents of past shallow-water areas and corresponding unexplored deposits for each stage. Data on deposits were retrieved from Macrostrat Database, applying the moderate filter (see Methods). Relevant areas are summarized in tables S7 and S9.

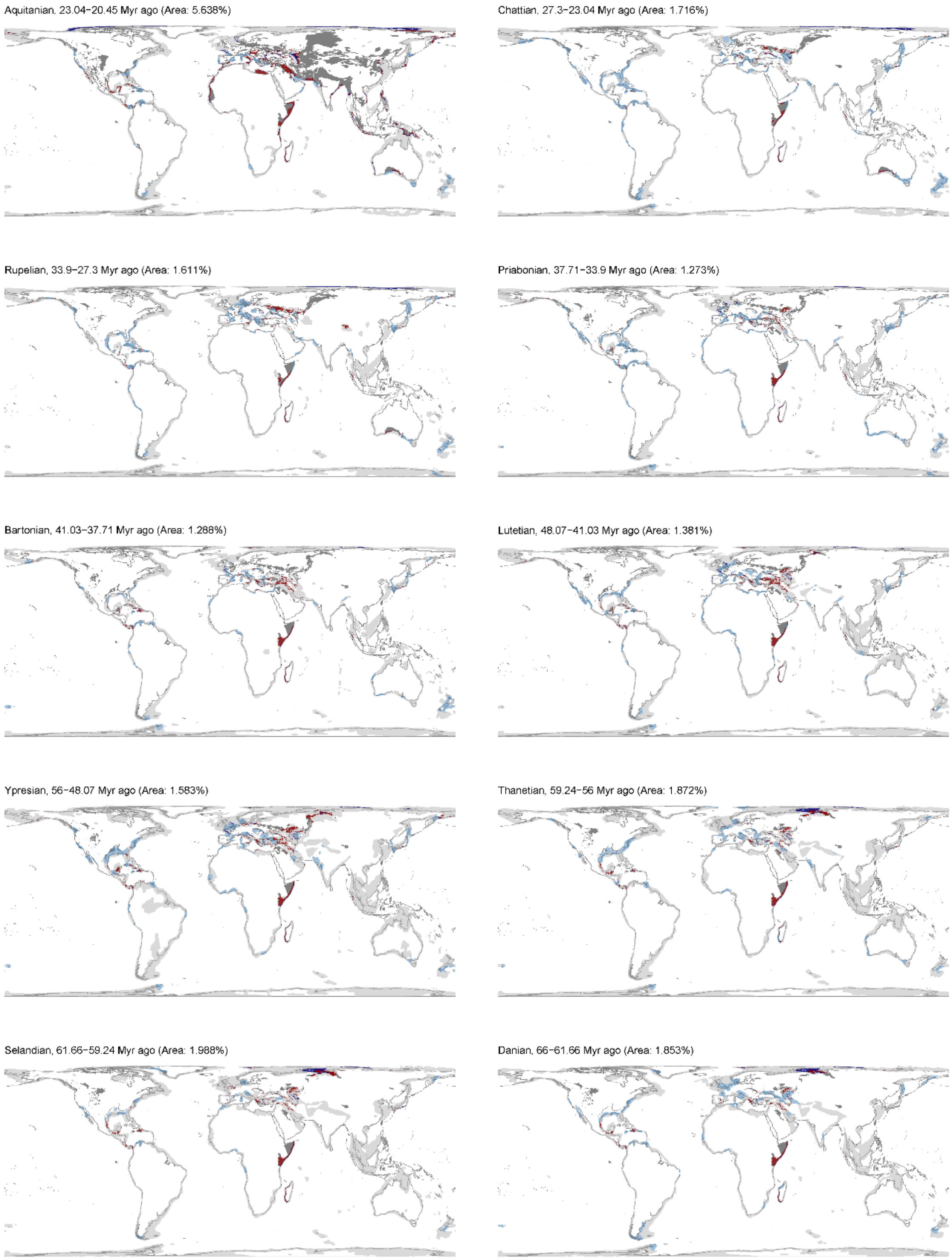

Fig. S21. Continued.

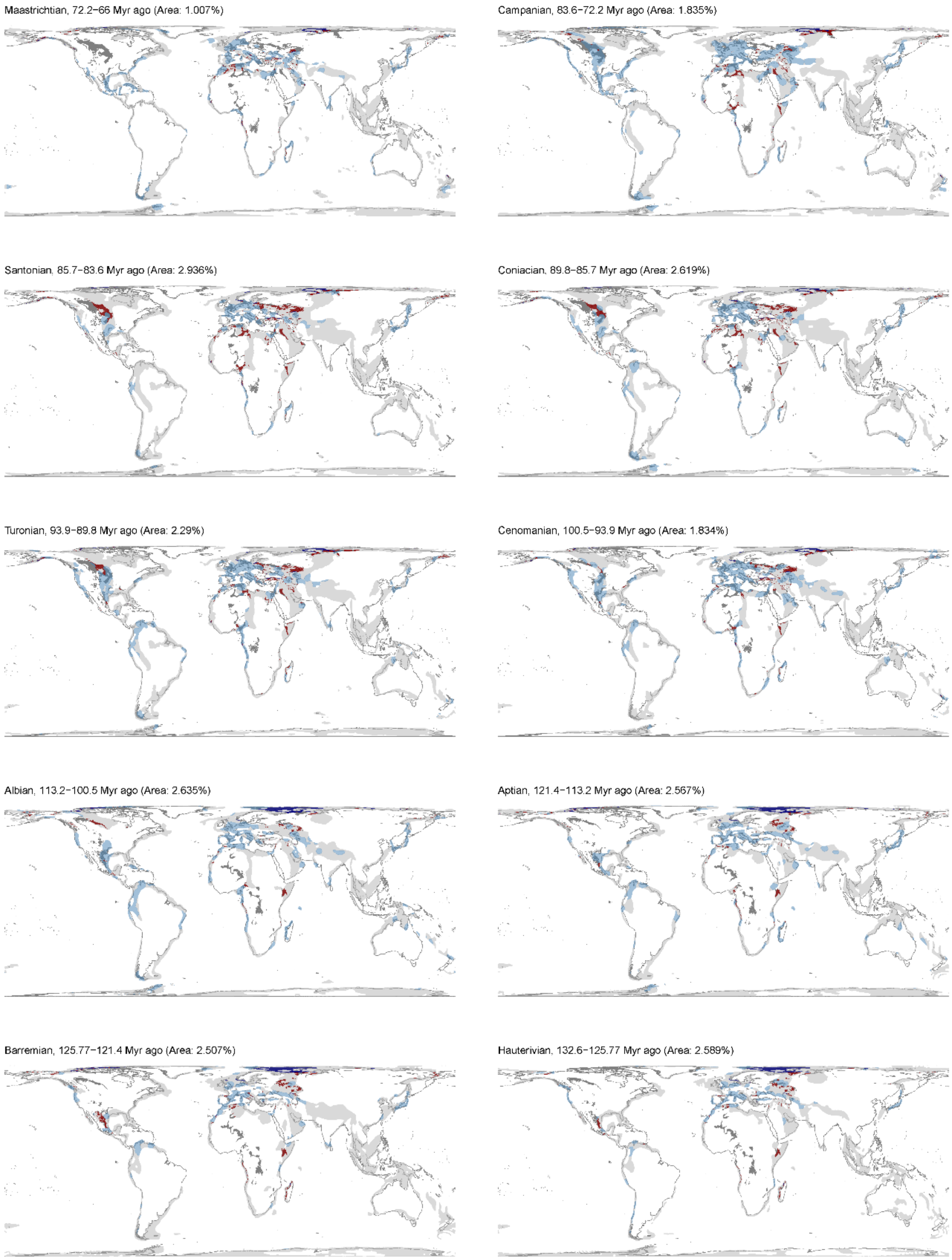

Fig. S21. Continued.

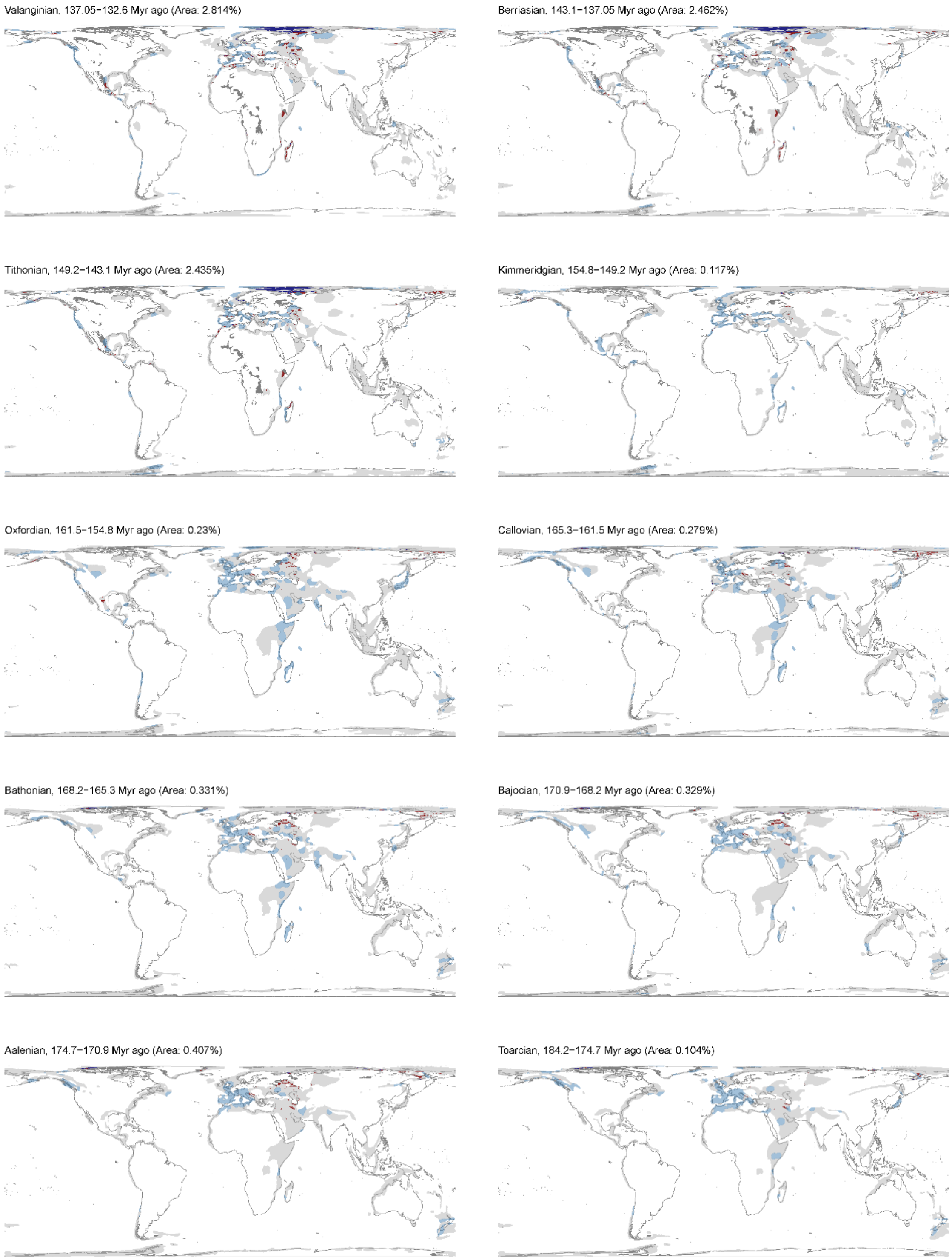

Fig. S21. Continued.

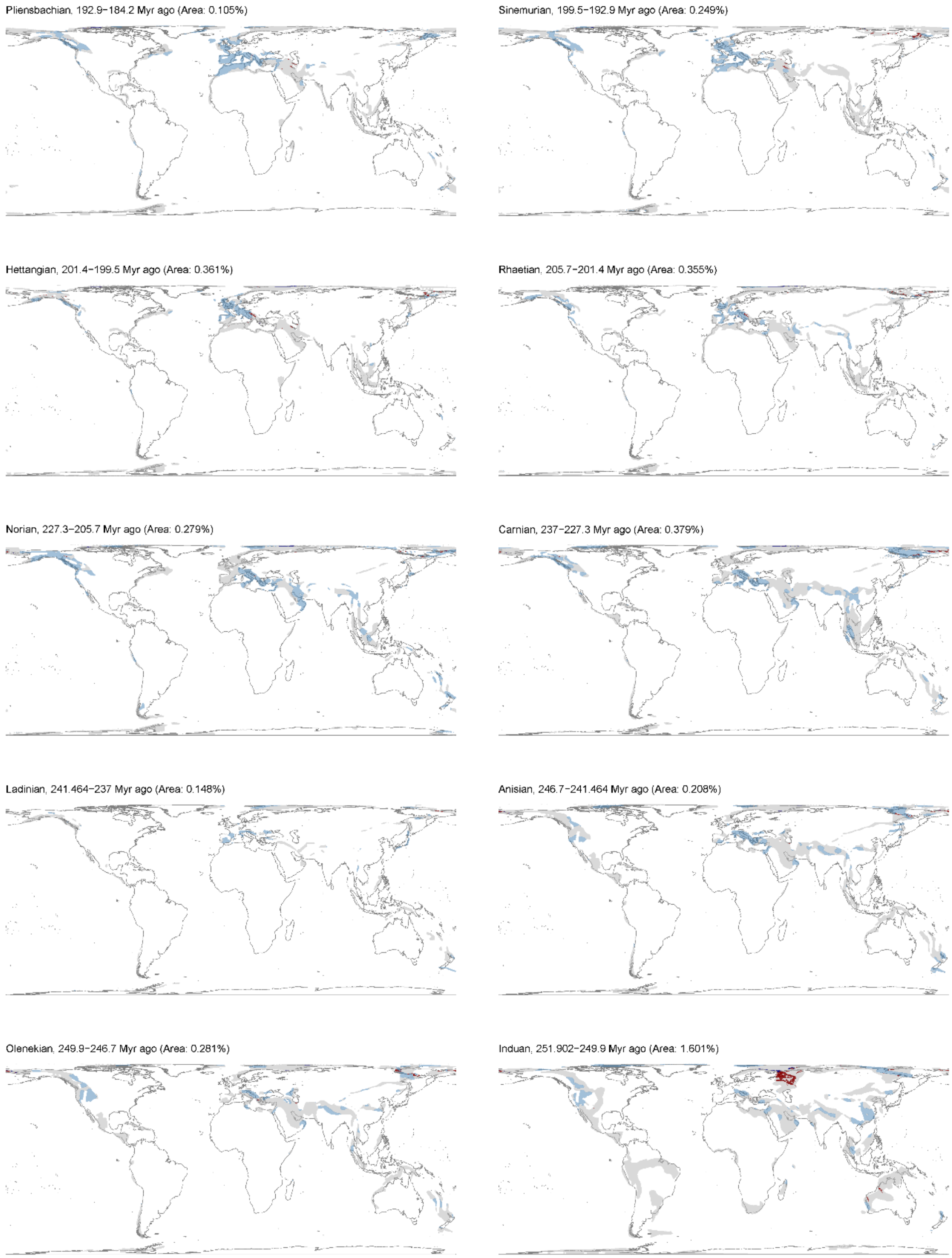

Fig. S21. Continued.

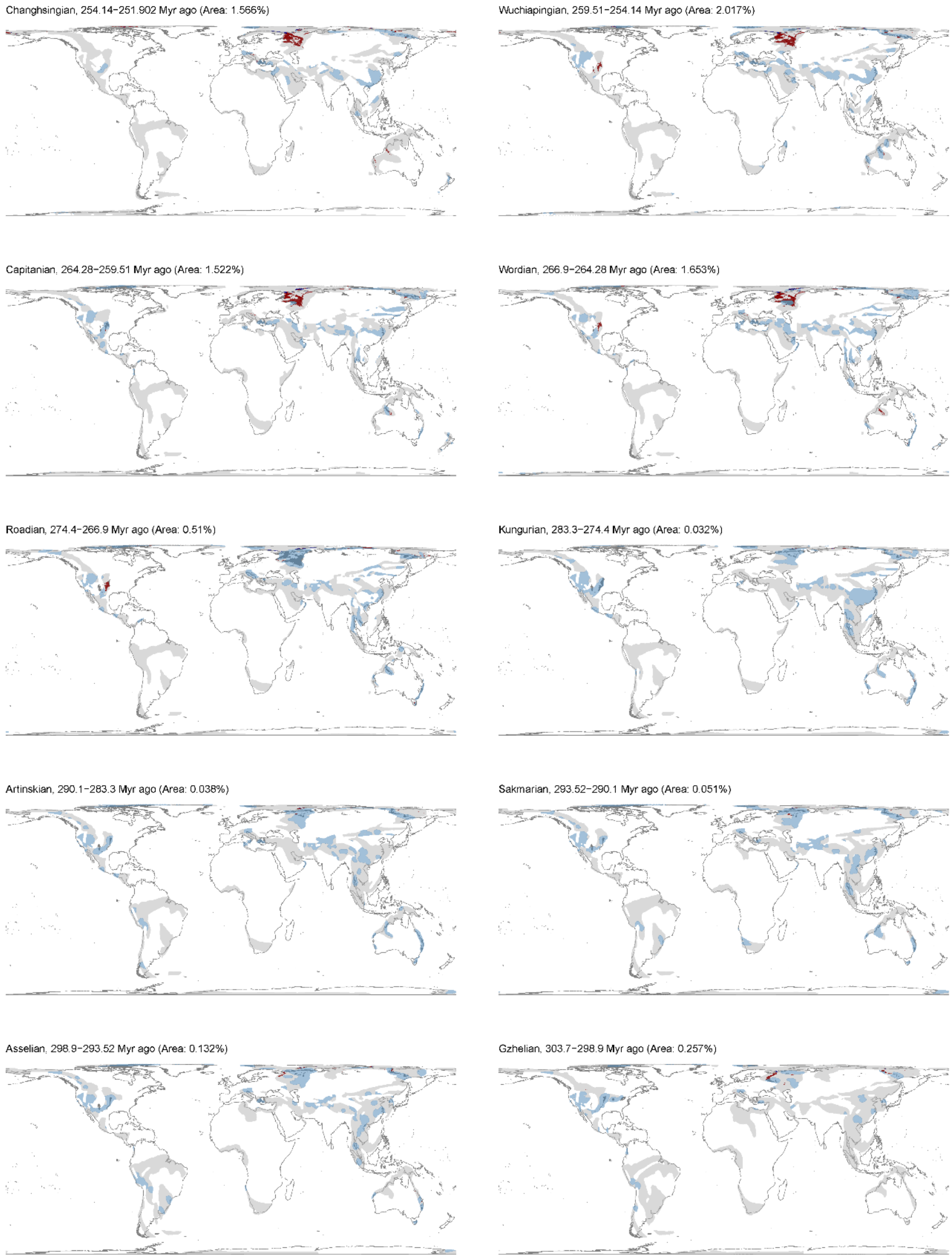

Fig. S21. Continued.

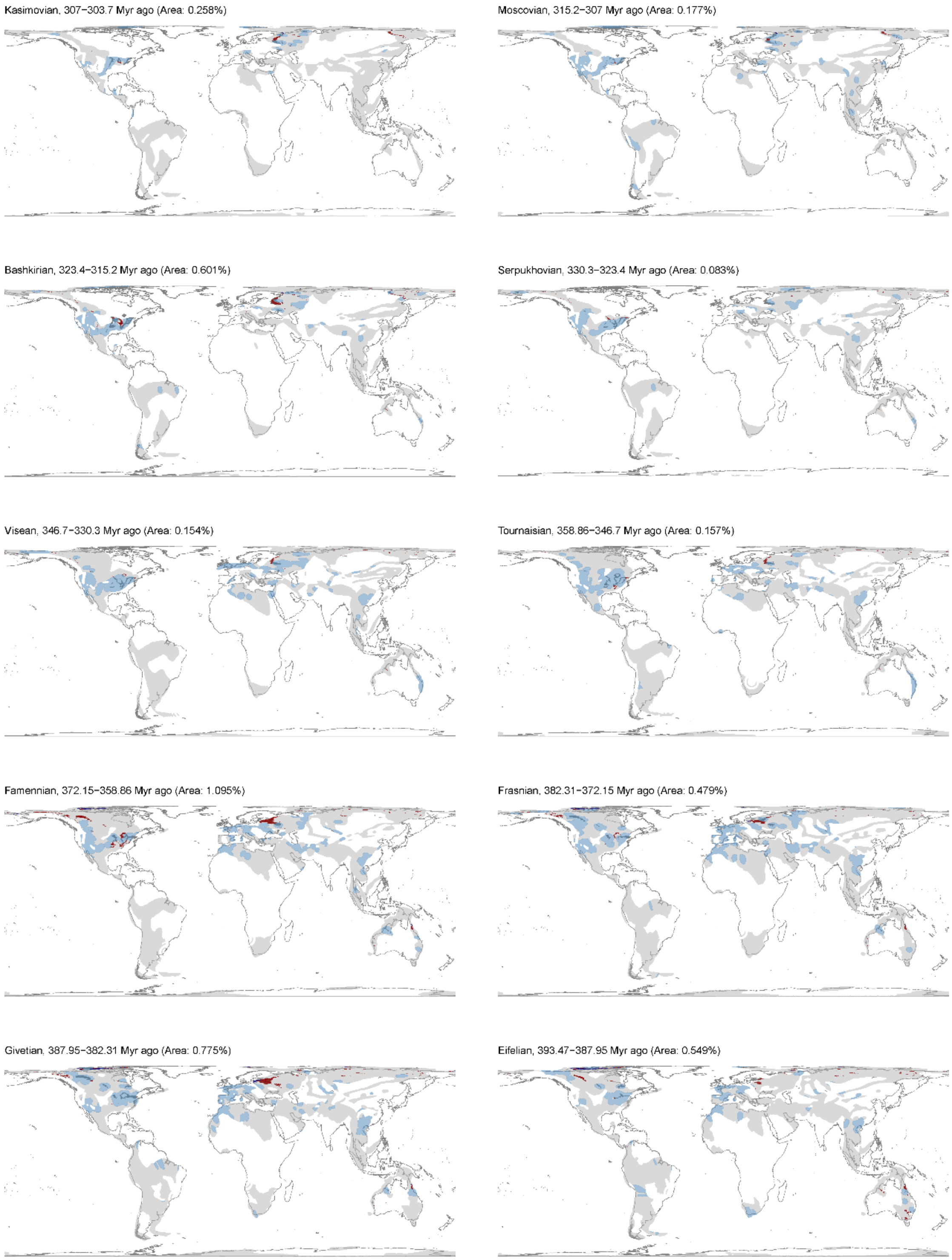

Fig. S21. Continued.

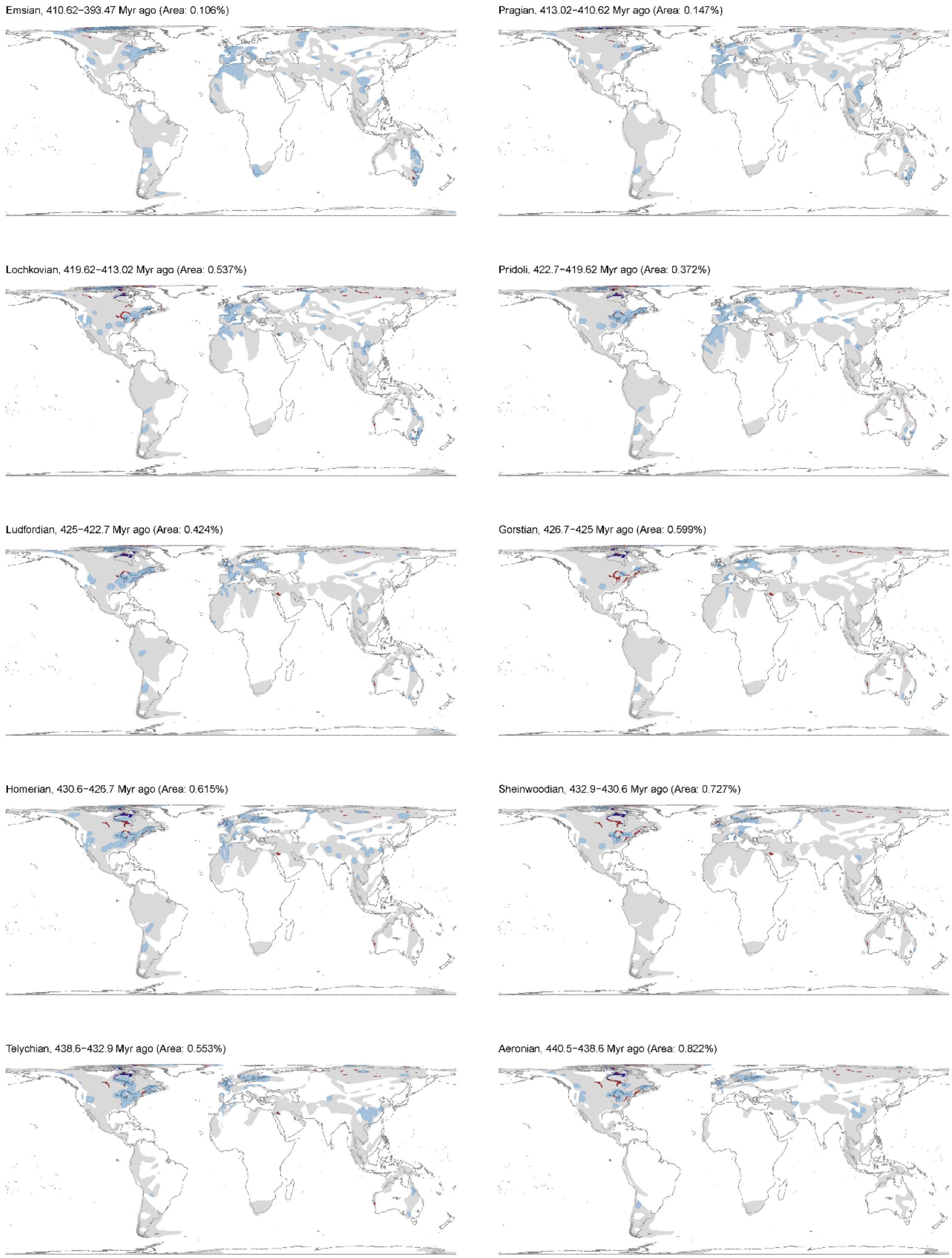

Fig. S21. Continued.

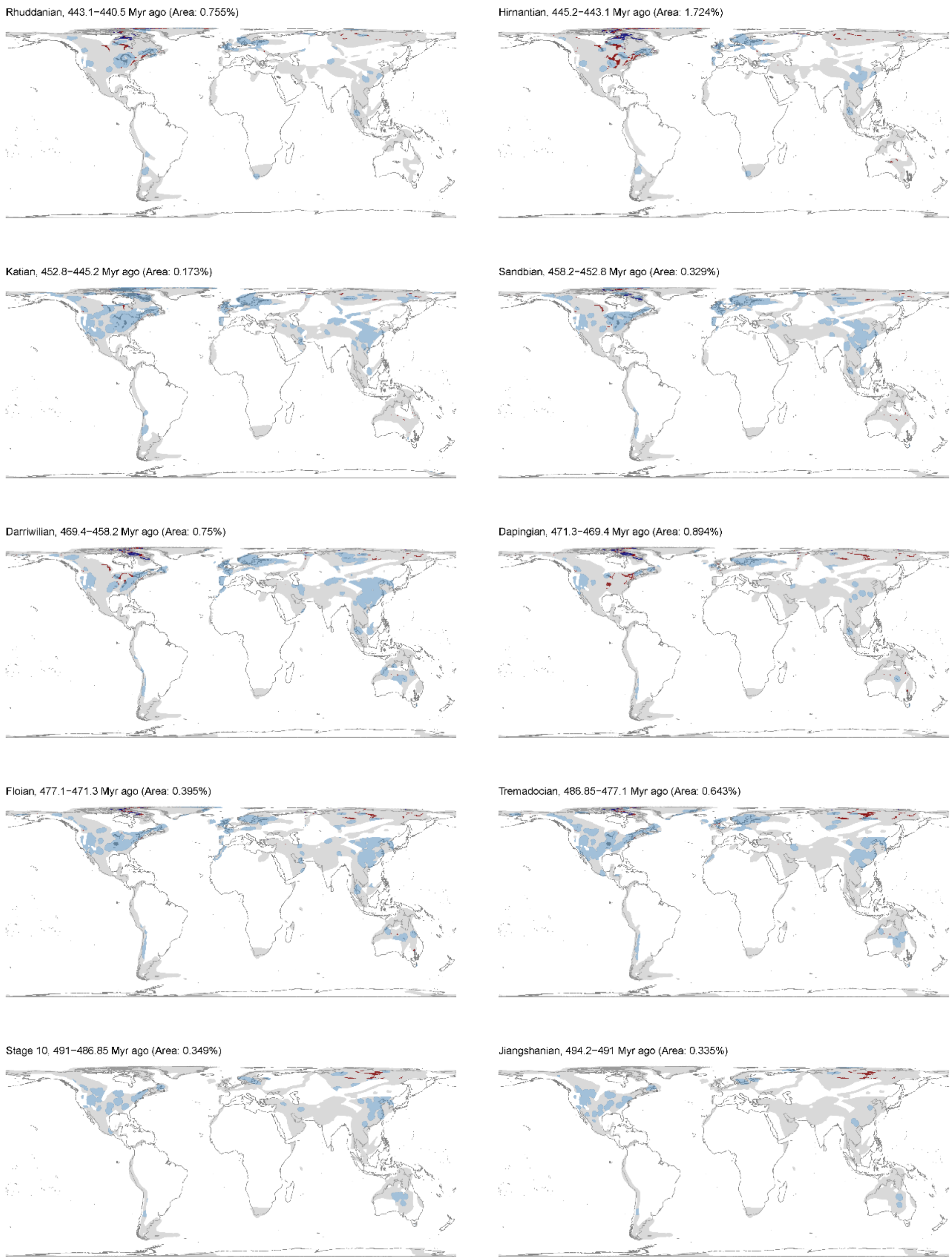

Fig. S21. Continued.

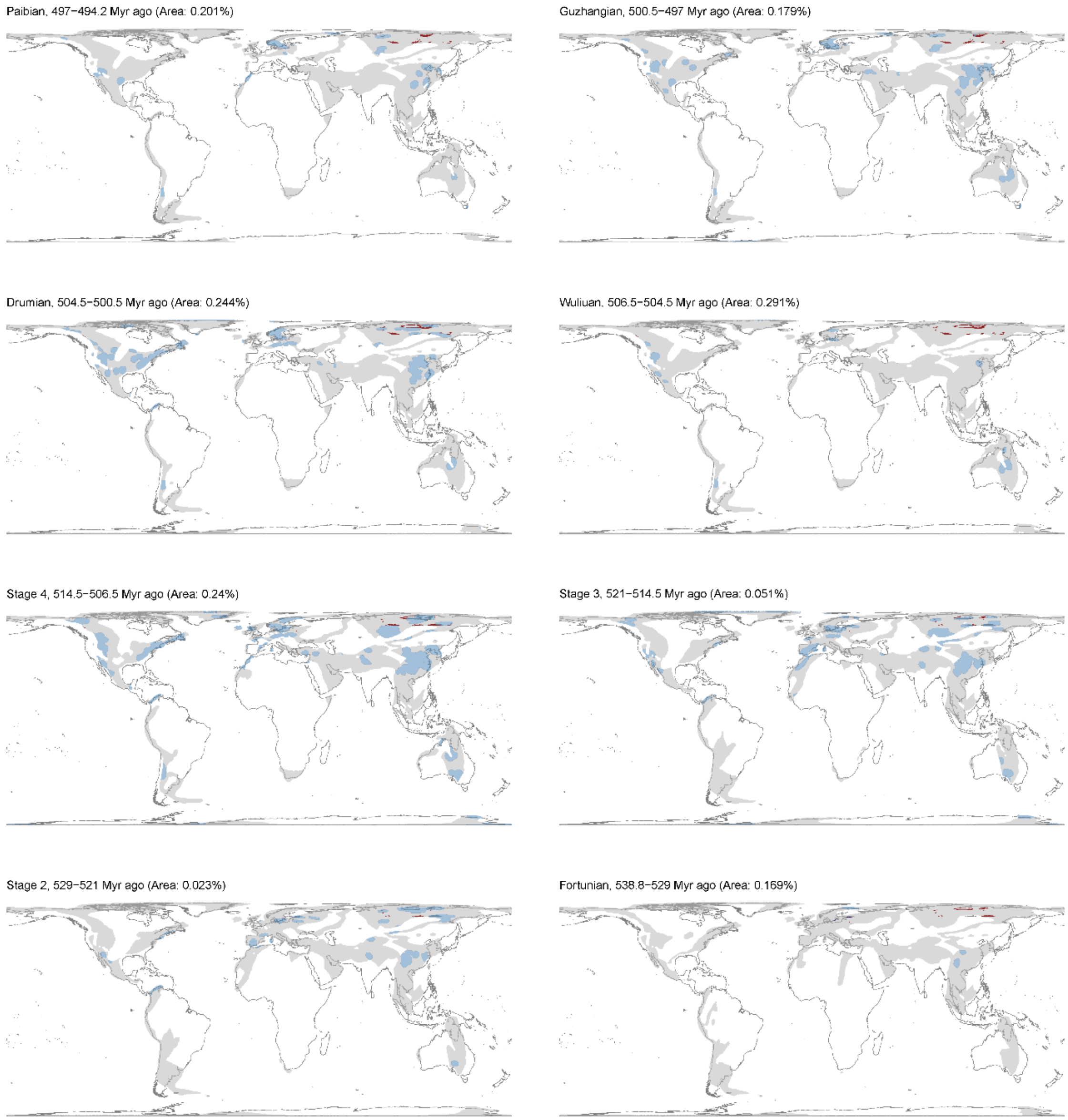

Fig. S21. Continued.

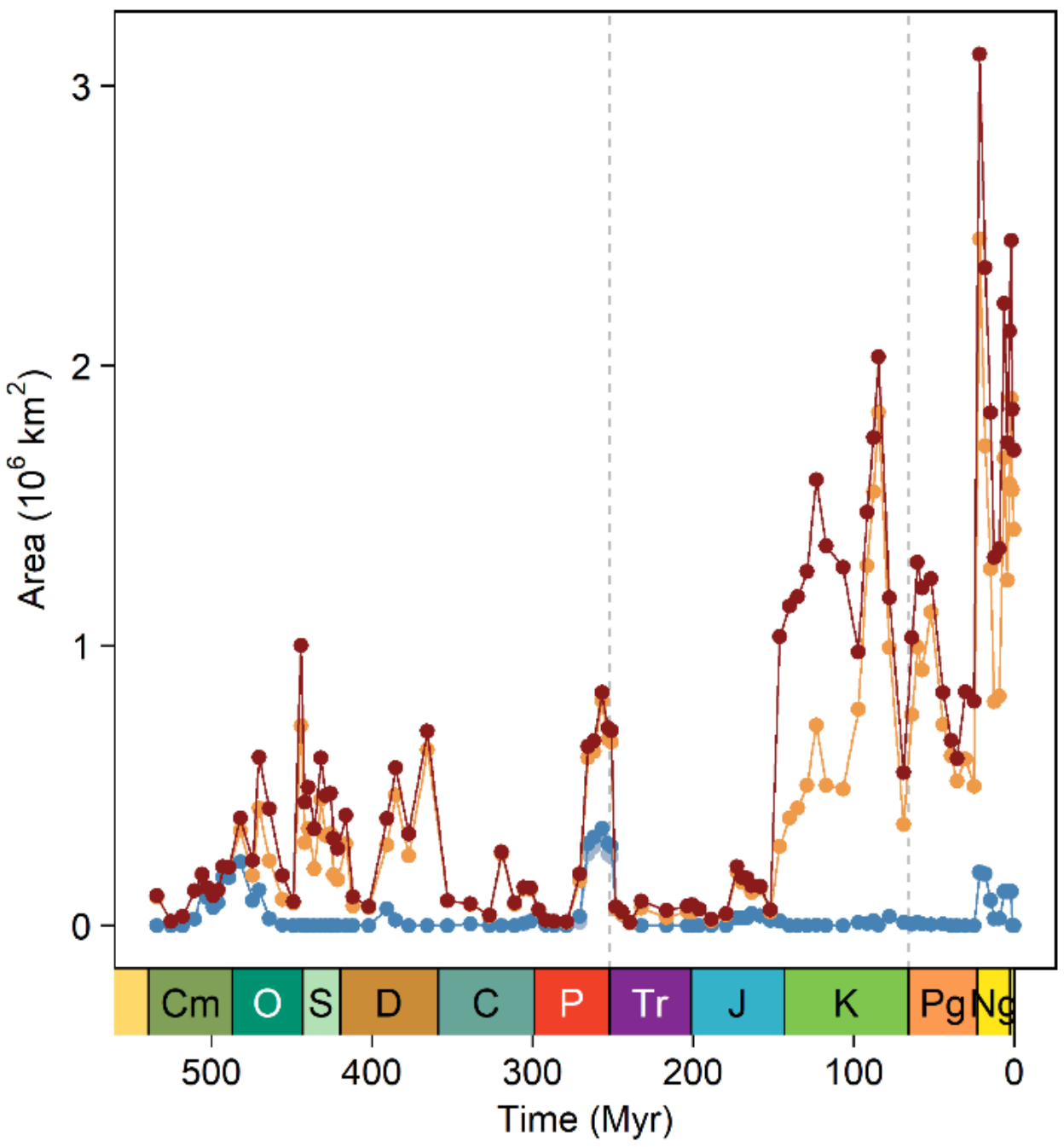

Fig. S22.

Areas of unexplored shallow-water deposits through time, for two data filters and distinguishing total area and deposits on land. The corresponding figure for percentages is in the main text (Figure 4); see tables S8–9 for the underlying data.

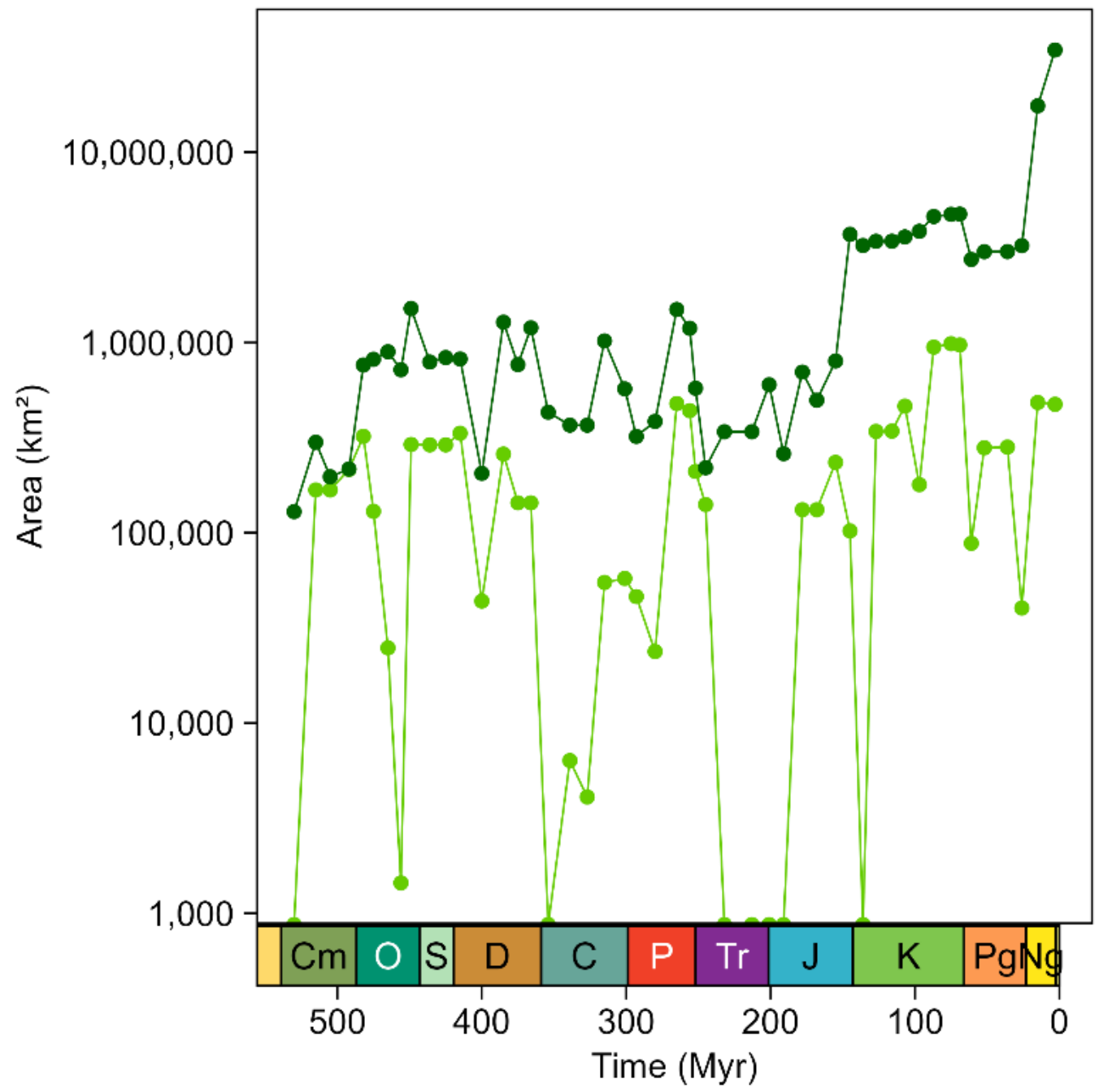

Fig. S23.
Areas of shallow-water deposits through time calculated from Macrostrat data (*31*), using the strict (chartreuse) and moderate (dark green) filters discussed in the Methods section. See tables S8–9 for data.

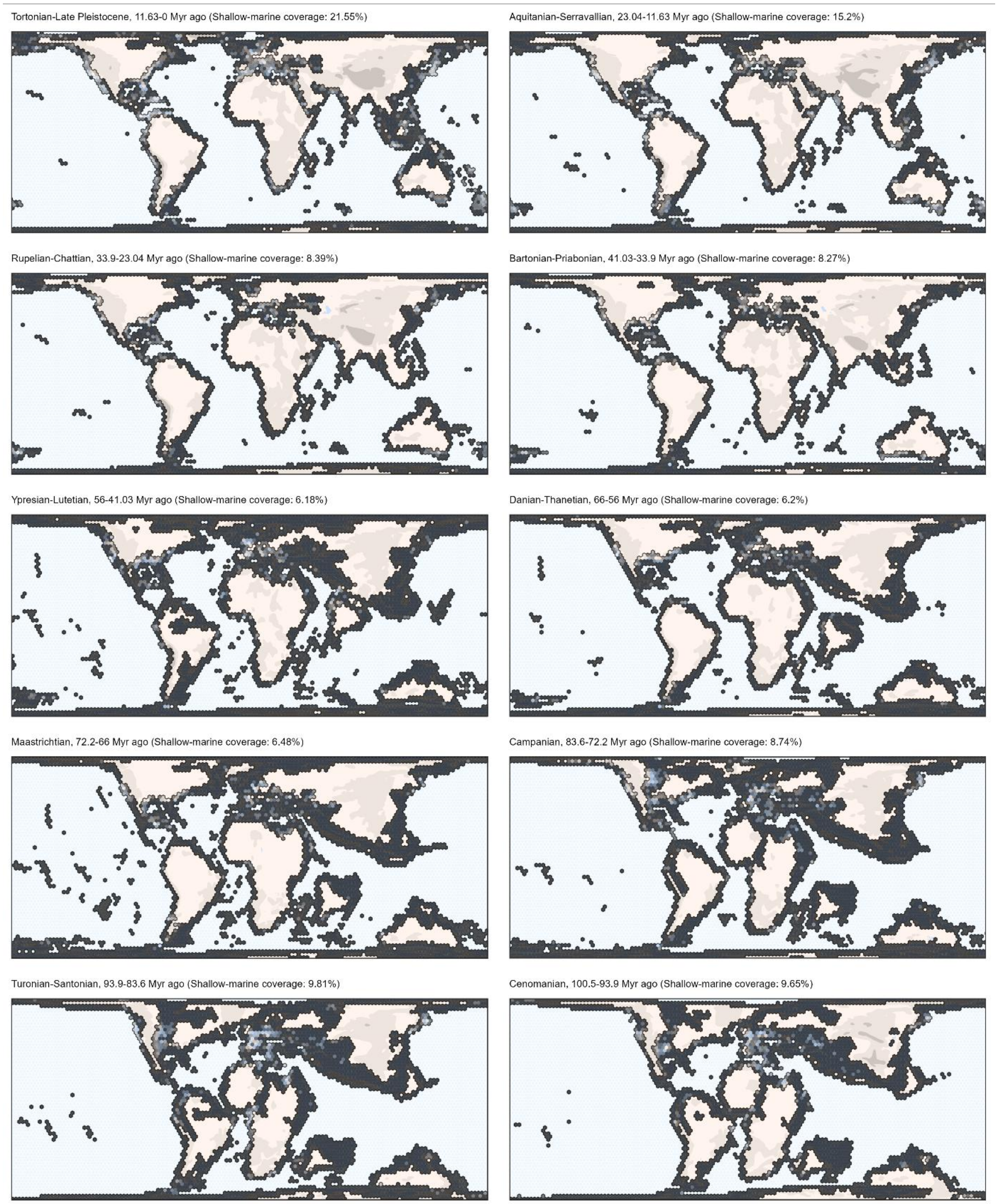

Fig. S24.

Paleogeographic maps for the 49-bin approach showing information density on shallow-marine metazoans as light hexagons for the 250 km pointy-topped grid. The number of fossil collections per grid cell is displayed as relative transparency (see Figure 1 in main text for legend). Corresponding data on coverage is summarized in table S10.

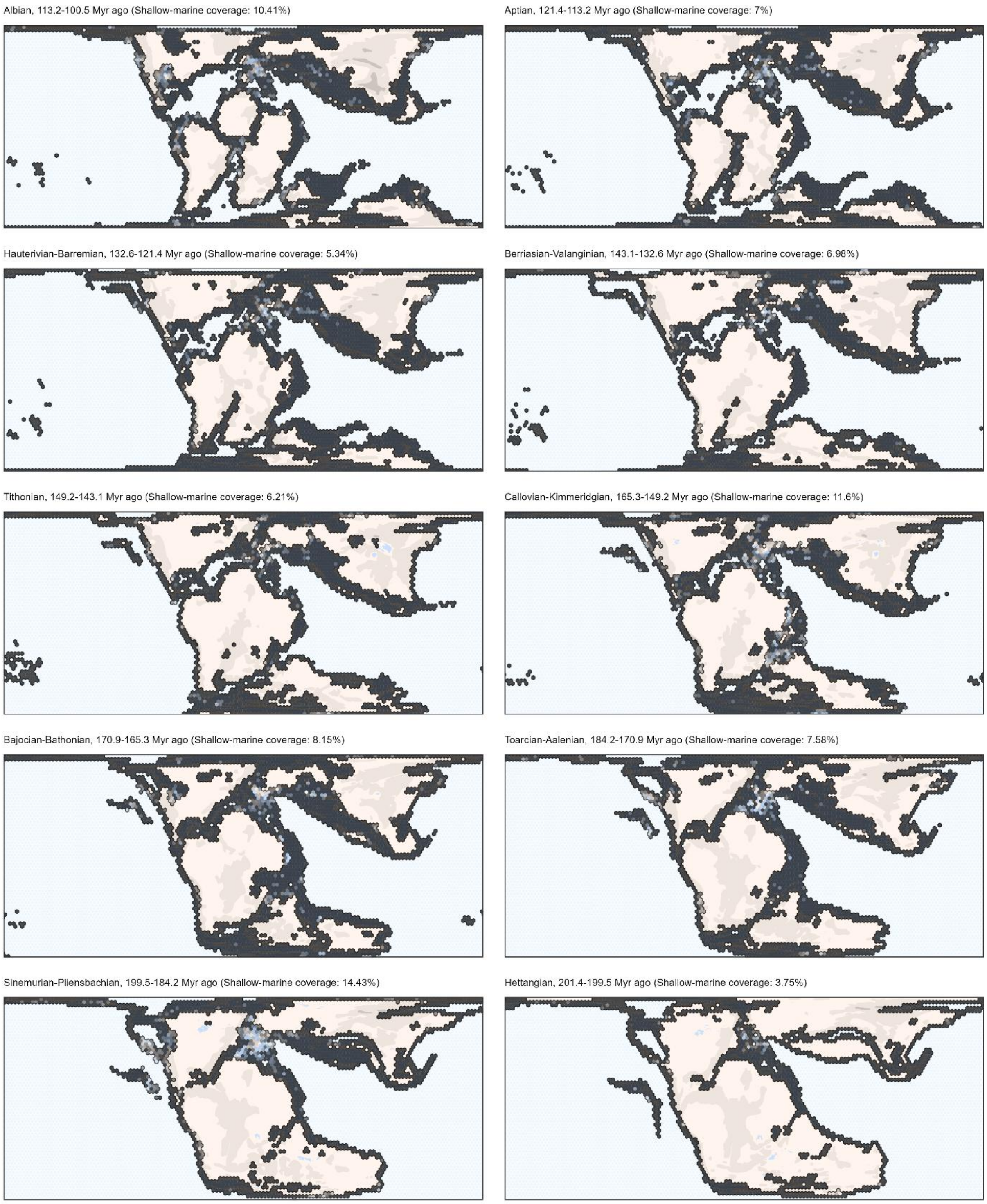

Fig. S24. Continued.

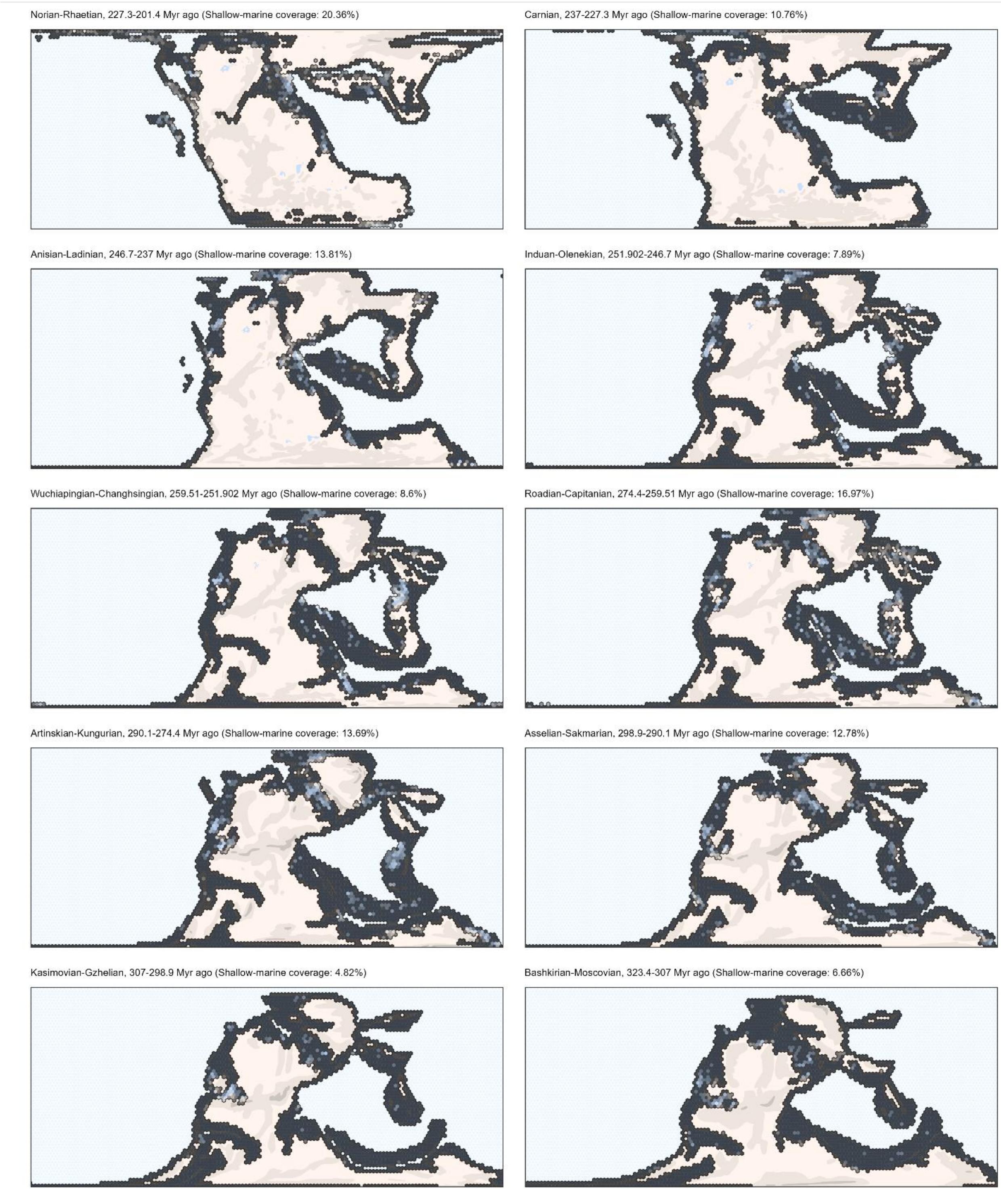

Fig. S24. Continued.

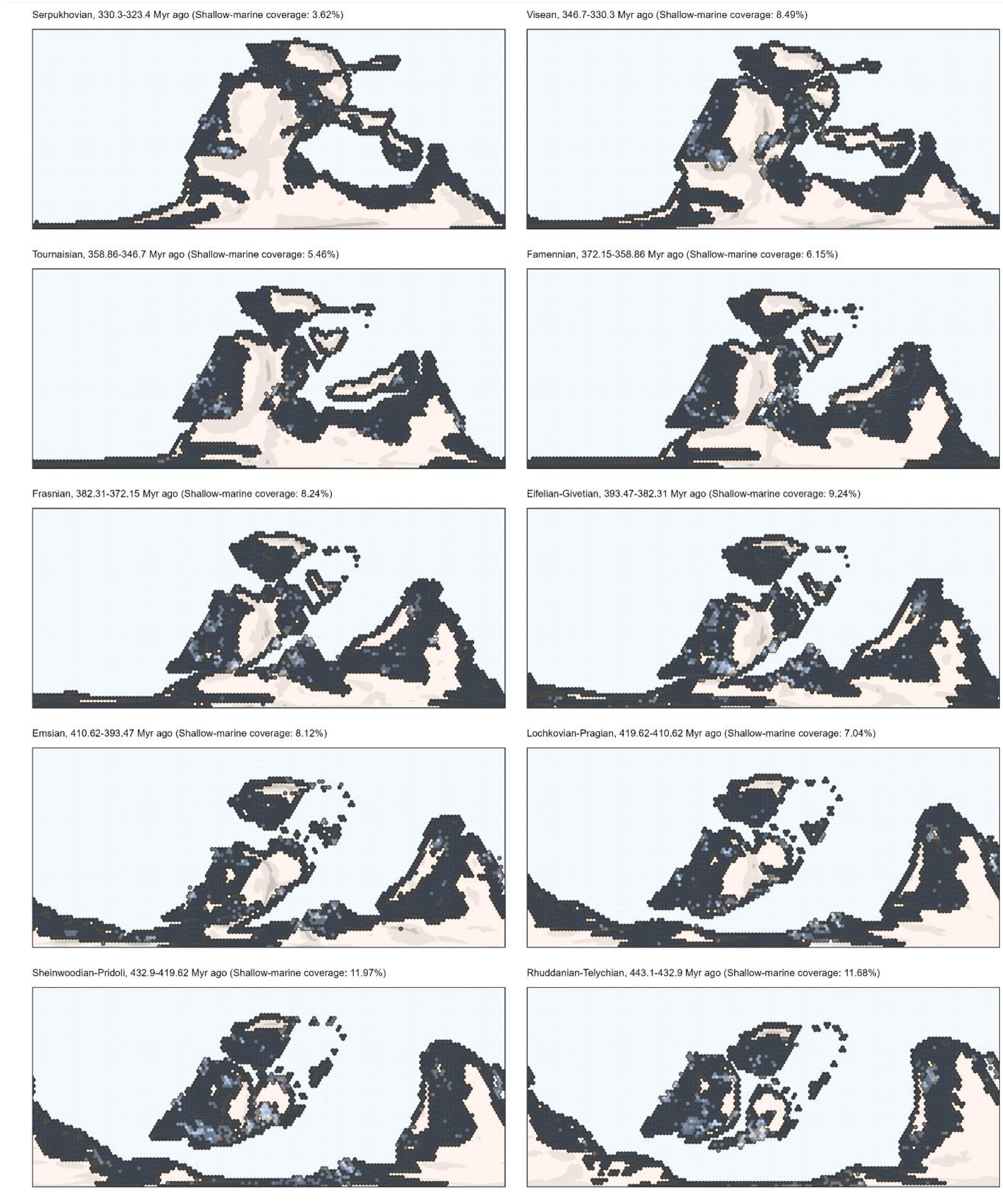

Fig. S24. Continued.

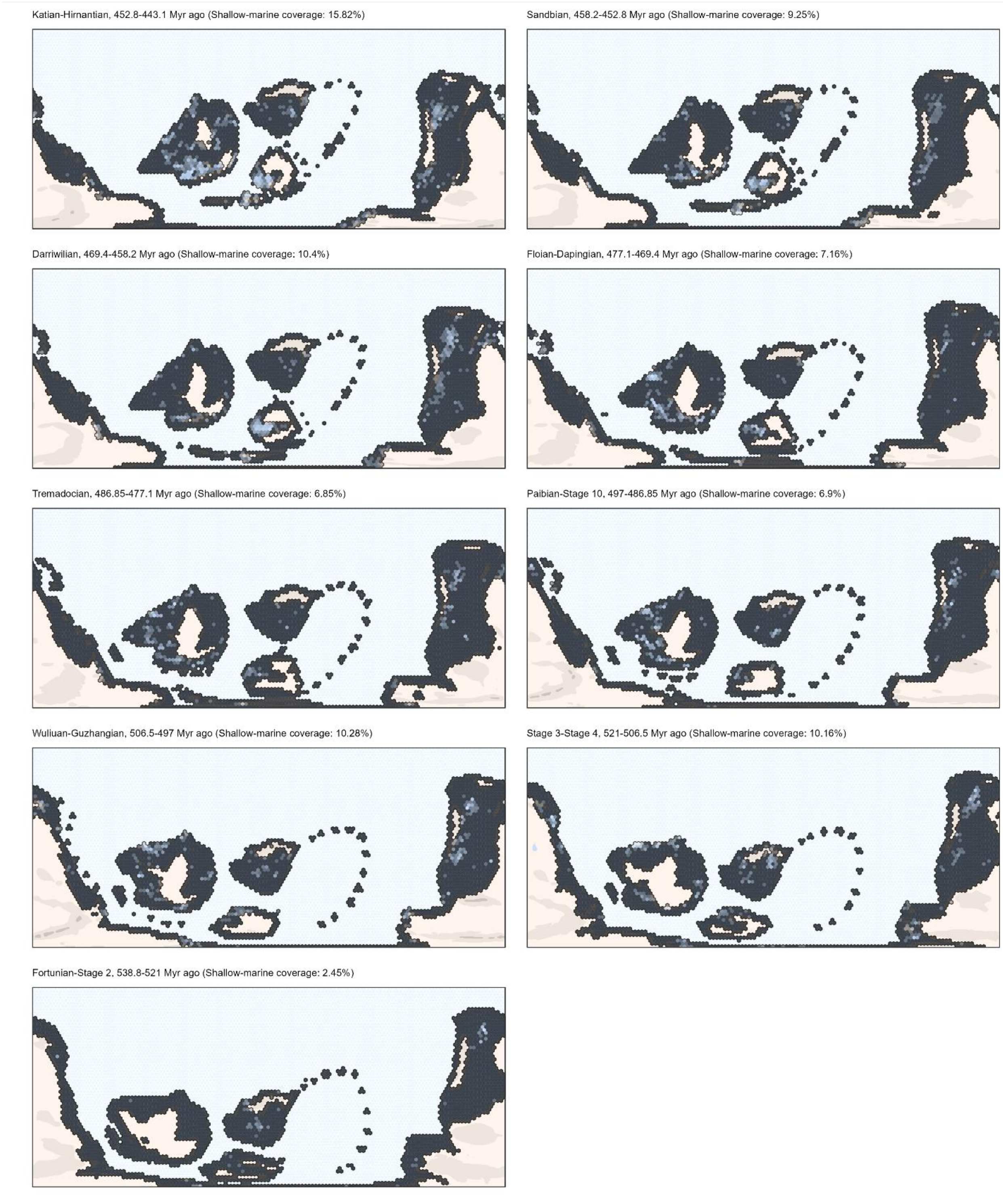

Fig. S24. Continued.

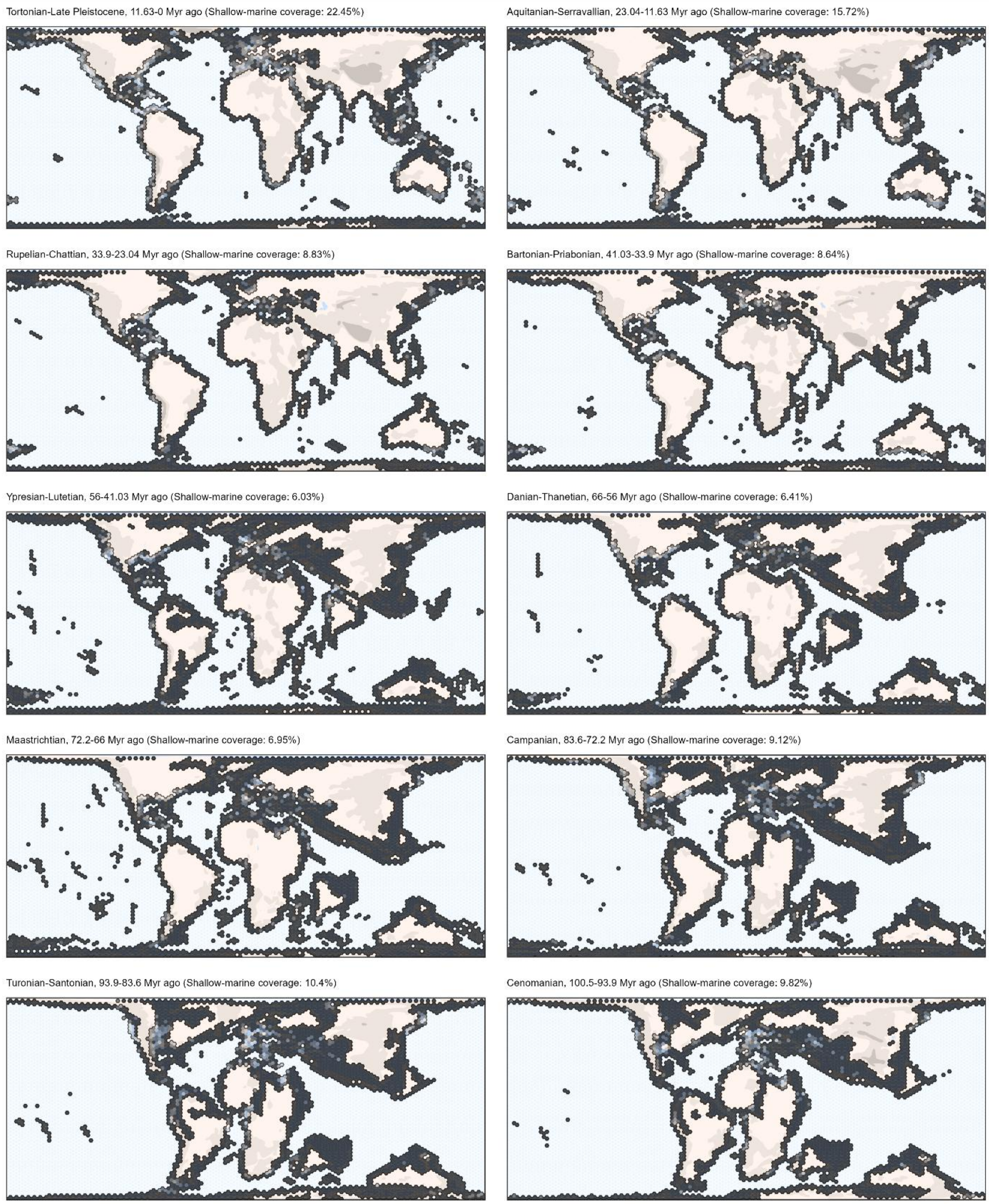

Fig. S25.

Paleogeographic maps for the 49-bin approach showing information density on shallow-marine metazoans as light hexagons for the 250 km flat-topped grid. The number of fossil collections per grid cell is displayed as relative transparency (see Figure 1 in main text for legend). Corresponding data on coverage is summarized in table S10.

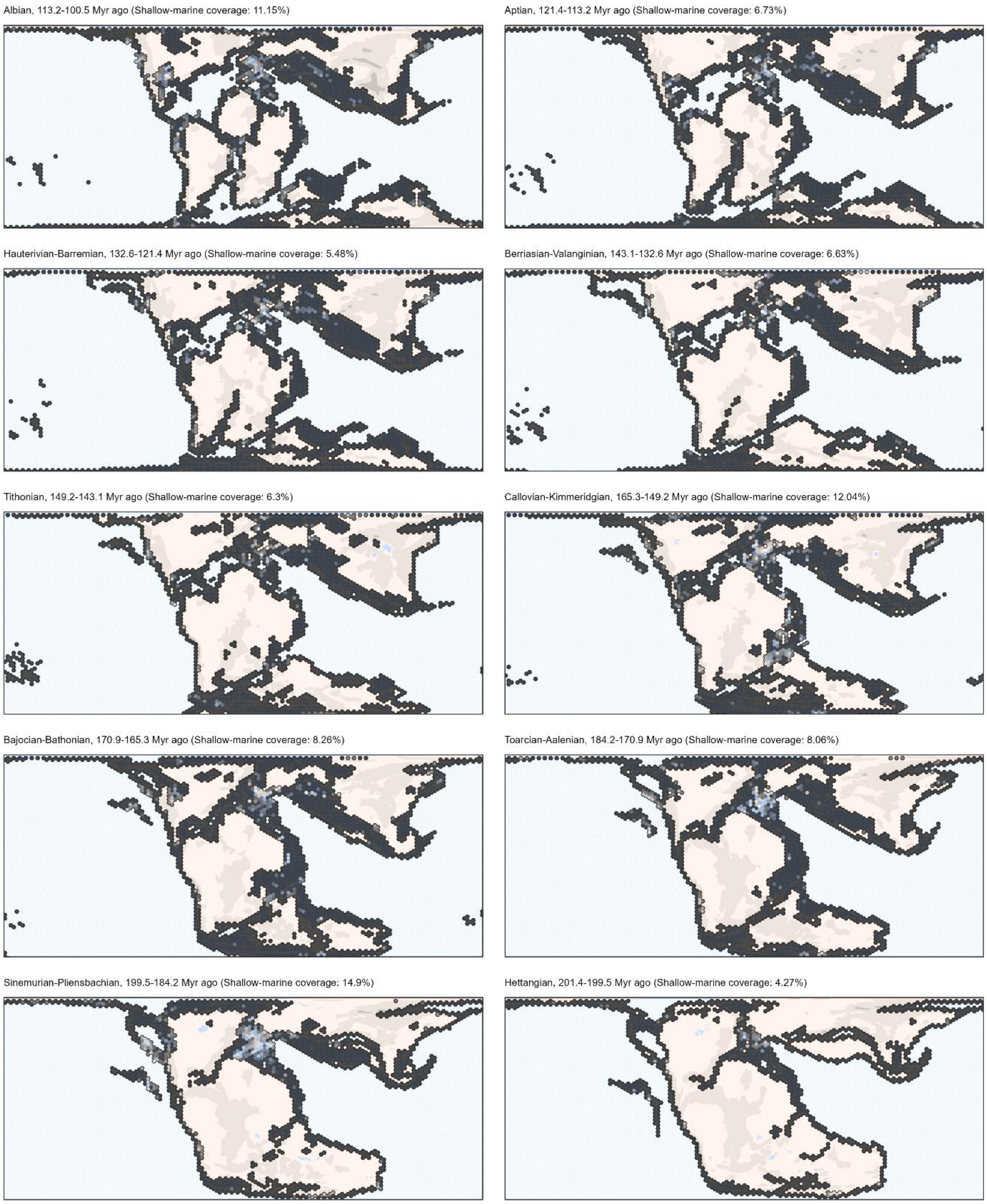

Fig. S25. Continued.

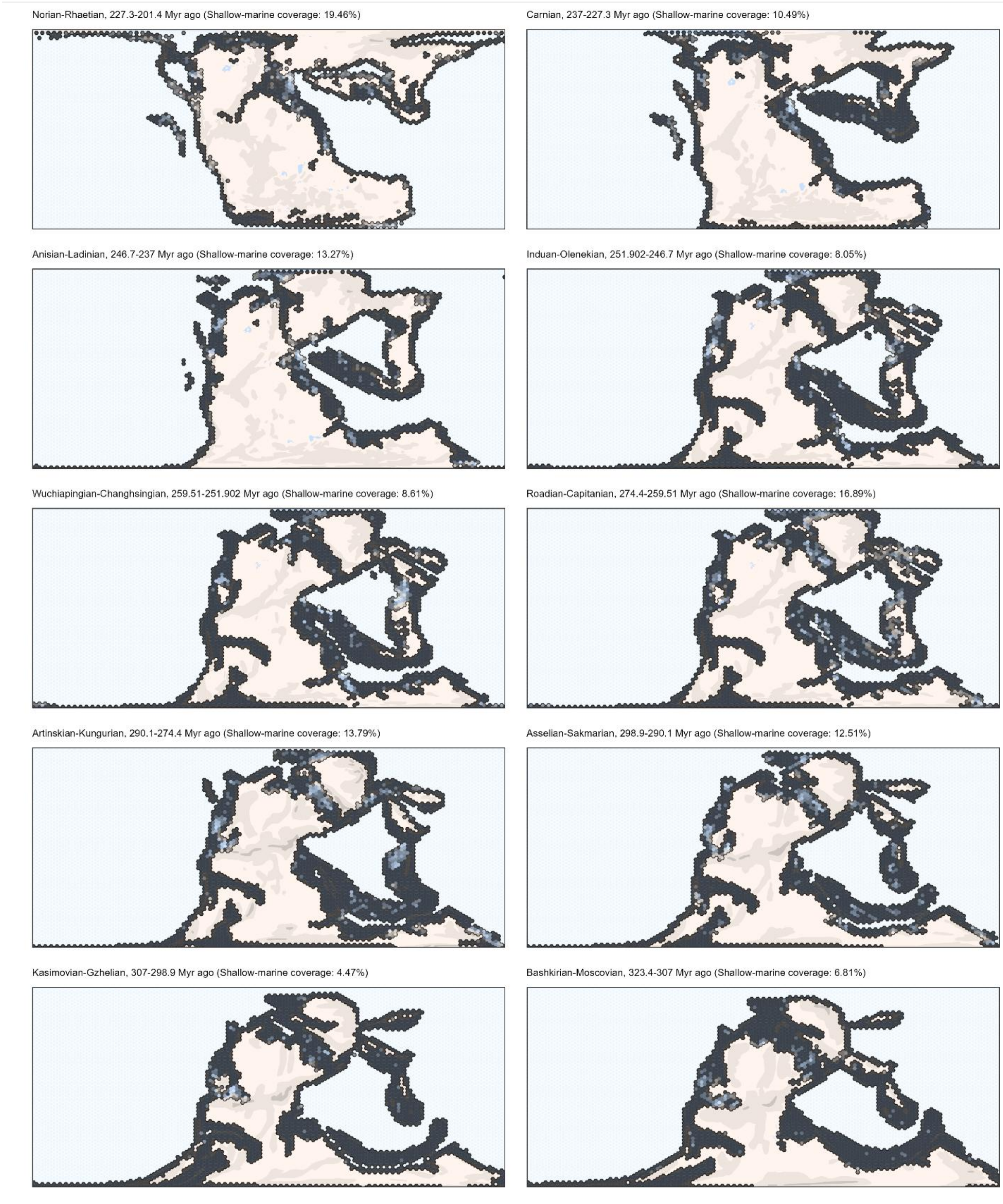

Fig. S25. Continued.

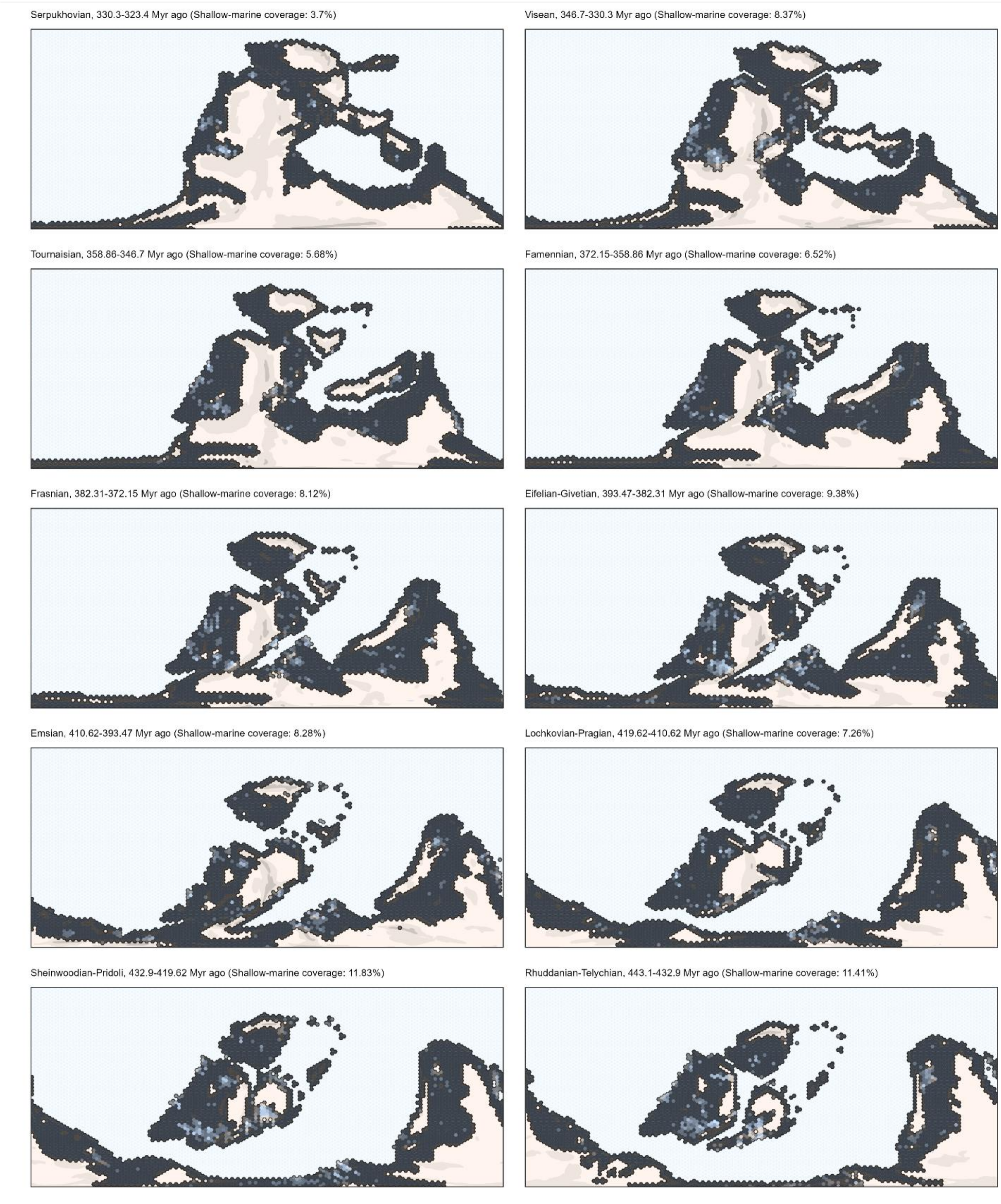

Fig. S25. Continued.

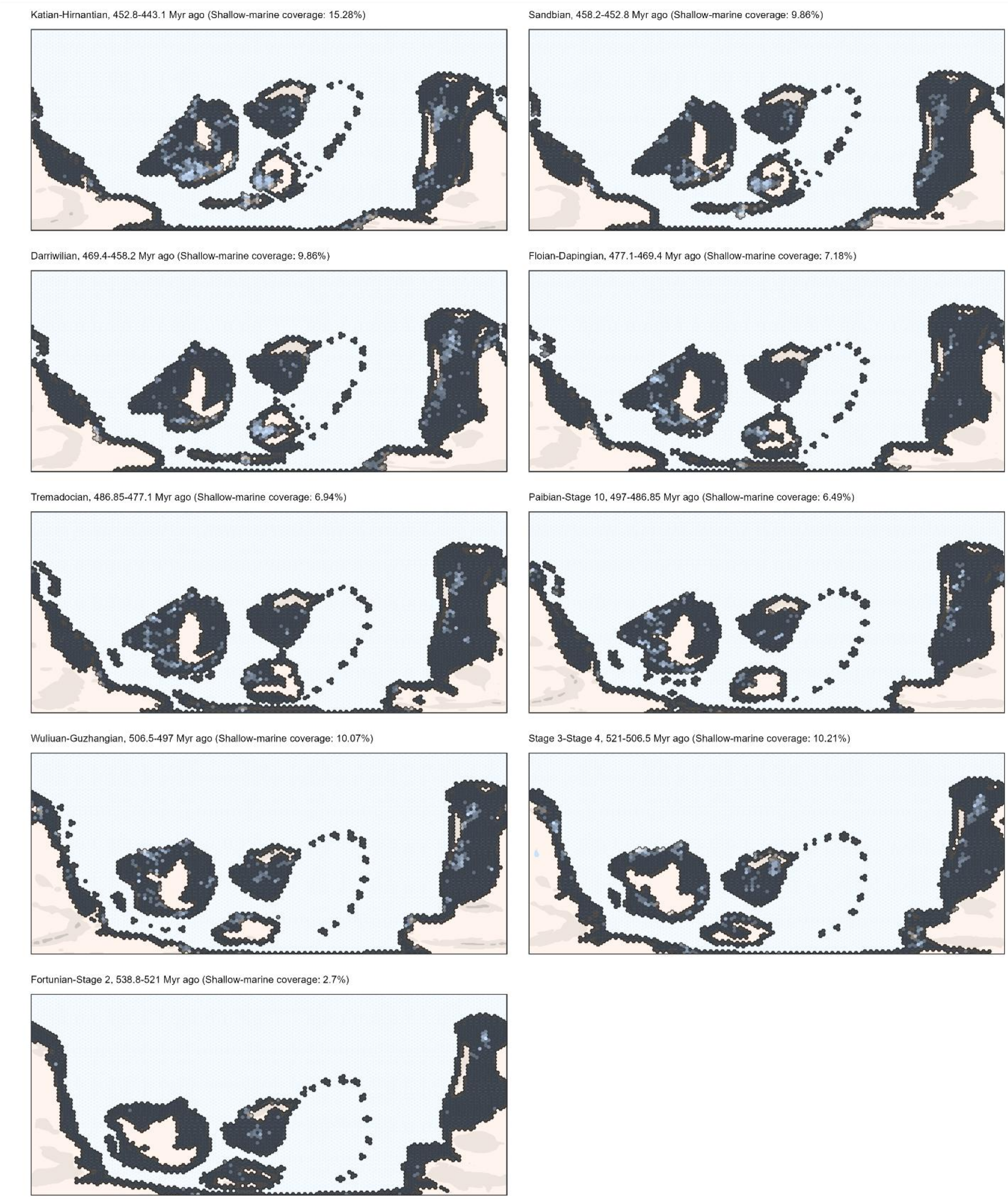

Fig. S25. Continued.

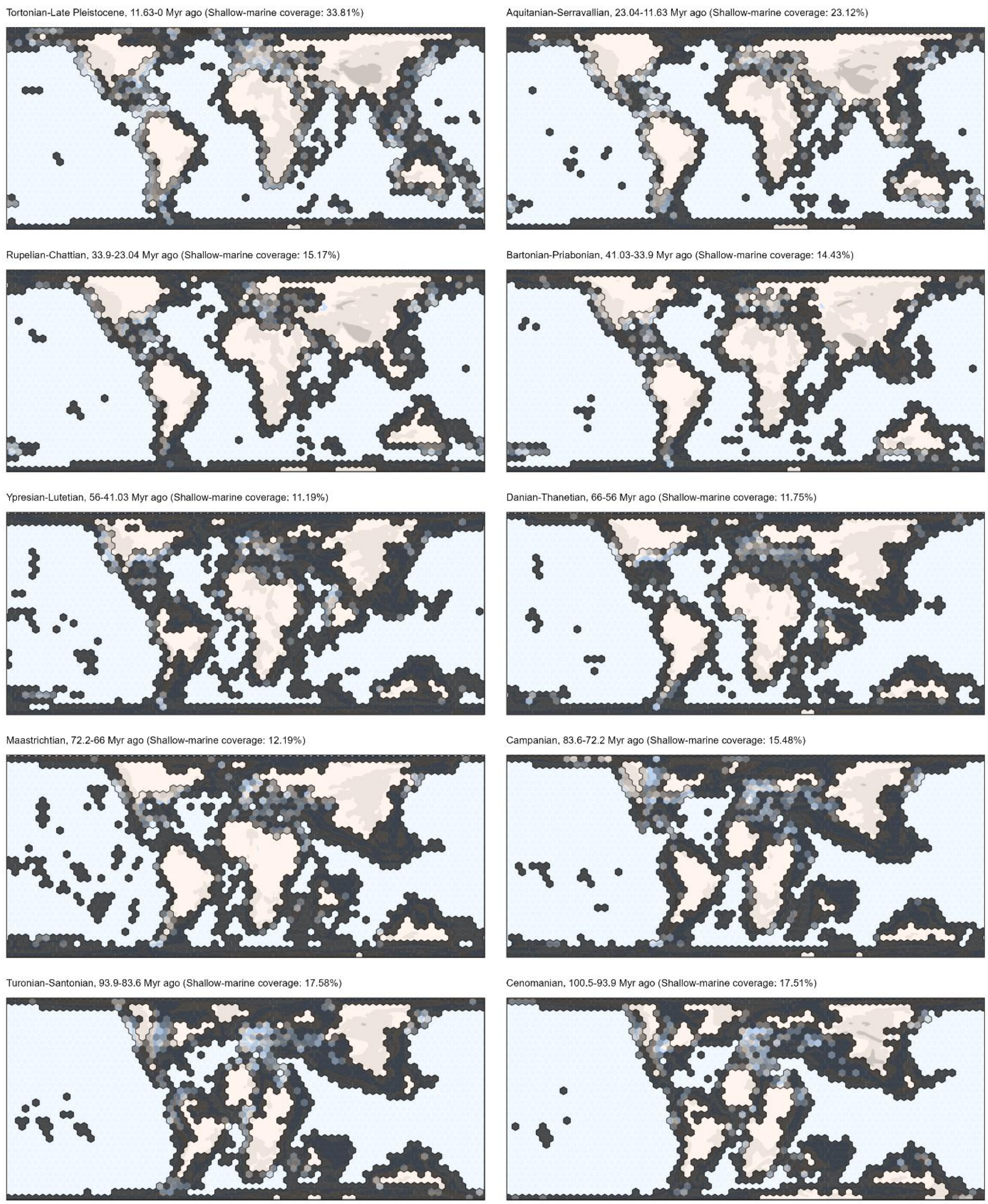

Fig. S26.

Paleogeographic maps for the 49-bin approach showing information density on shallow-marine metazoans as light hexagons for the 500 km pointy-topped grid. The number of fossil collections per grid cell is displayed as relative transparency (see Figure 1 in main text for legend). Corresponding data on coverage is summarized in table S10.

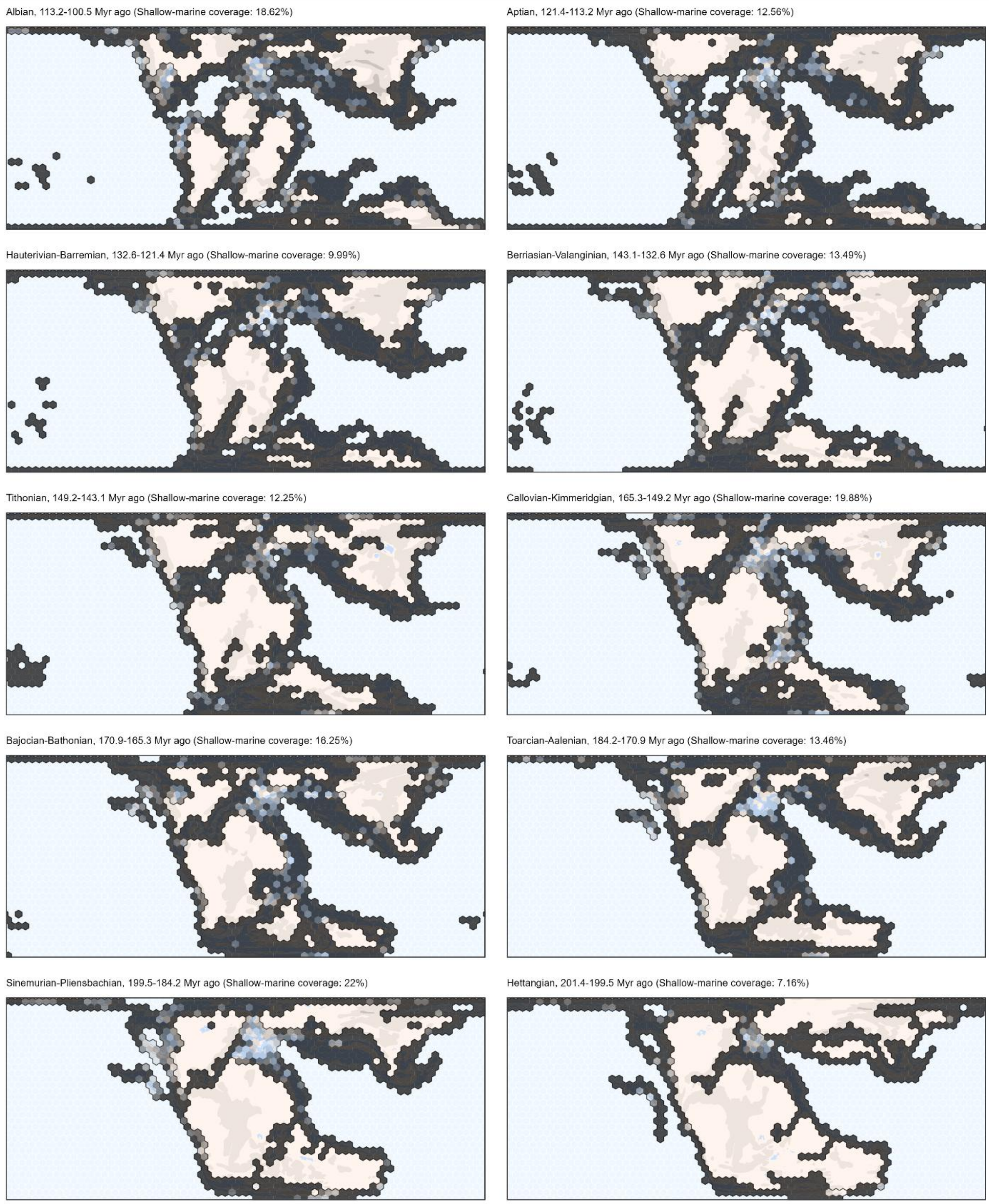

Fig. S26. Continued.

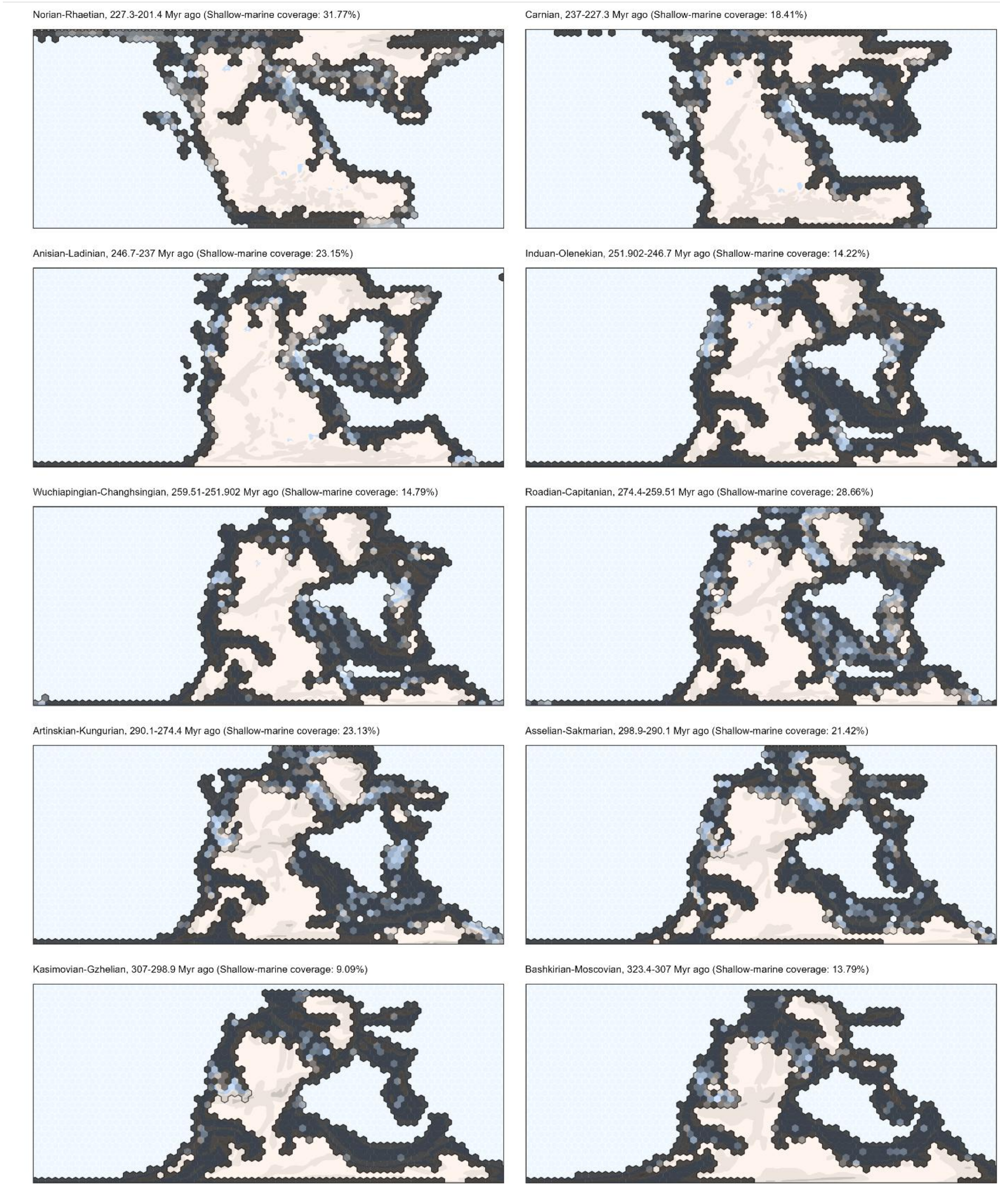

Fig. S26. Continued.

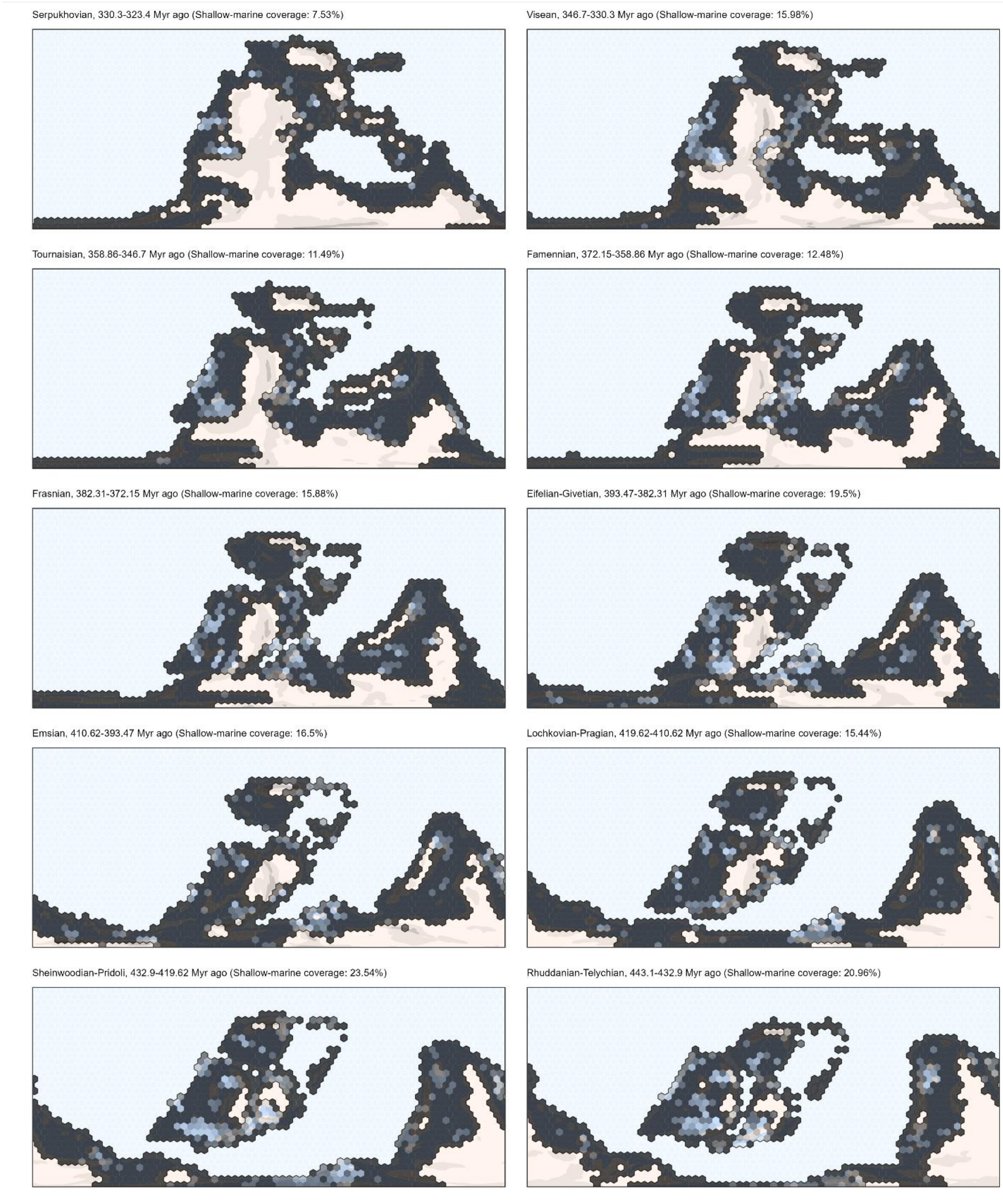

Fig. S26. Continued.

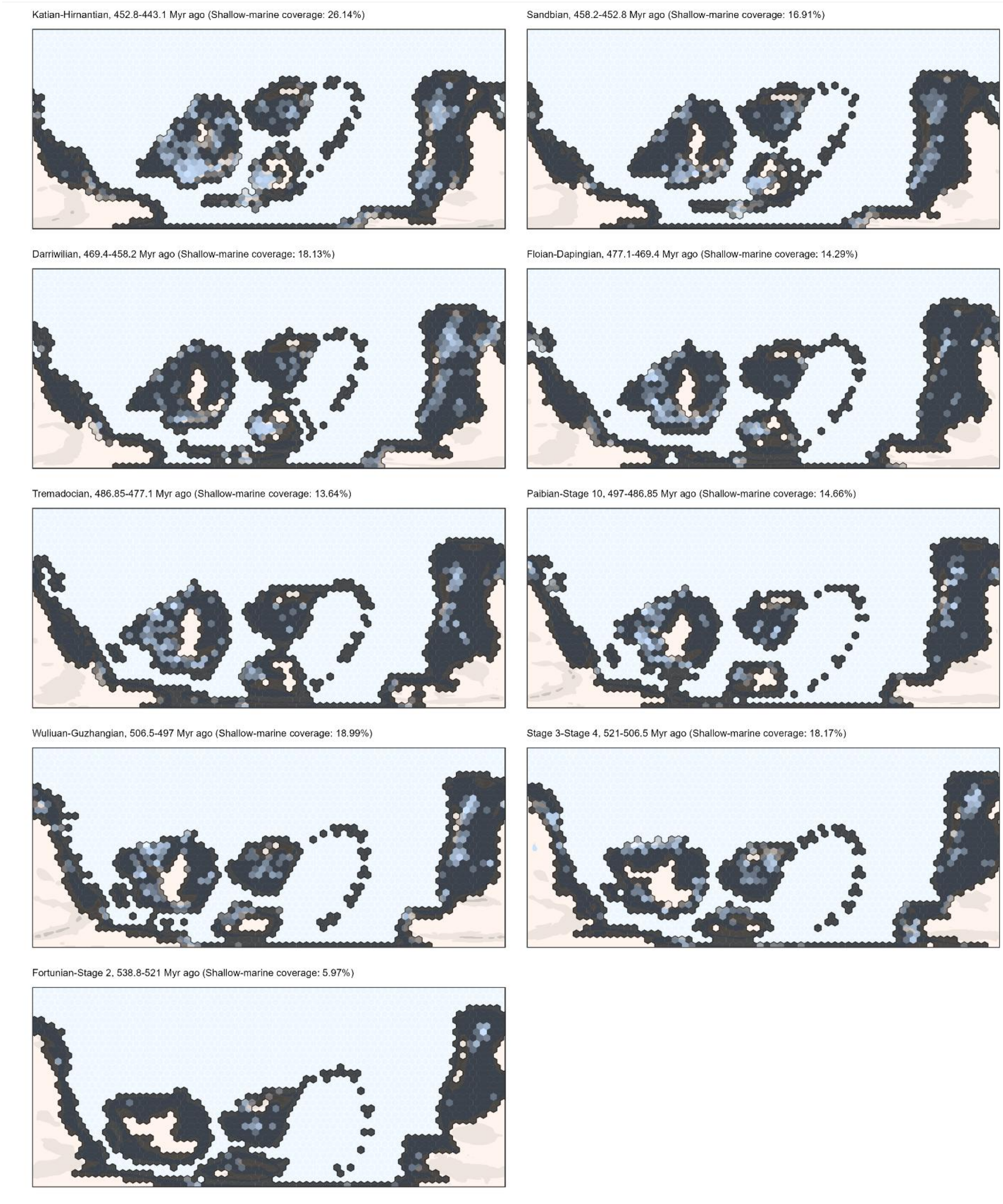

Fig. S26. Continued.

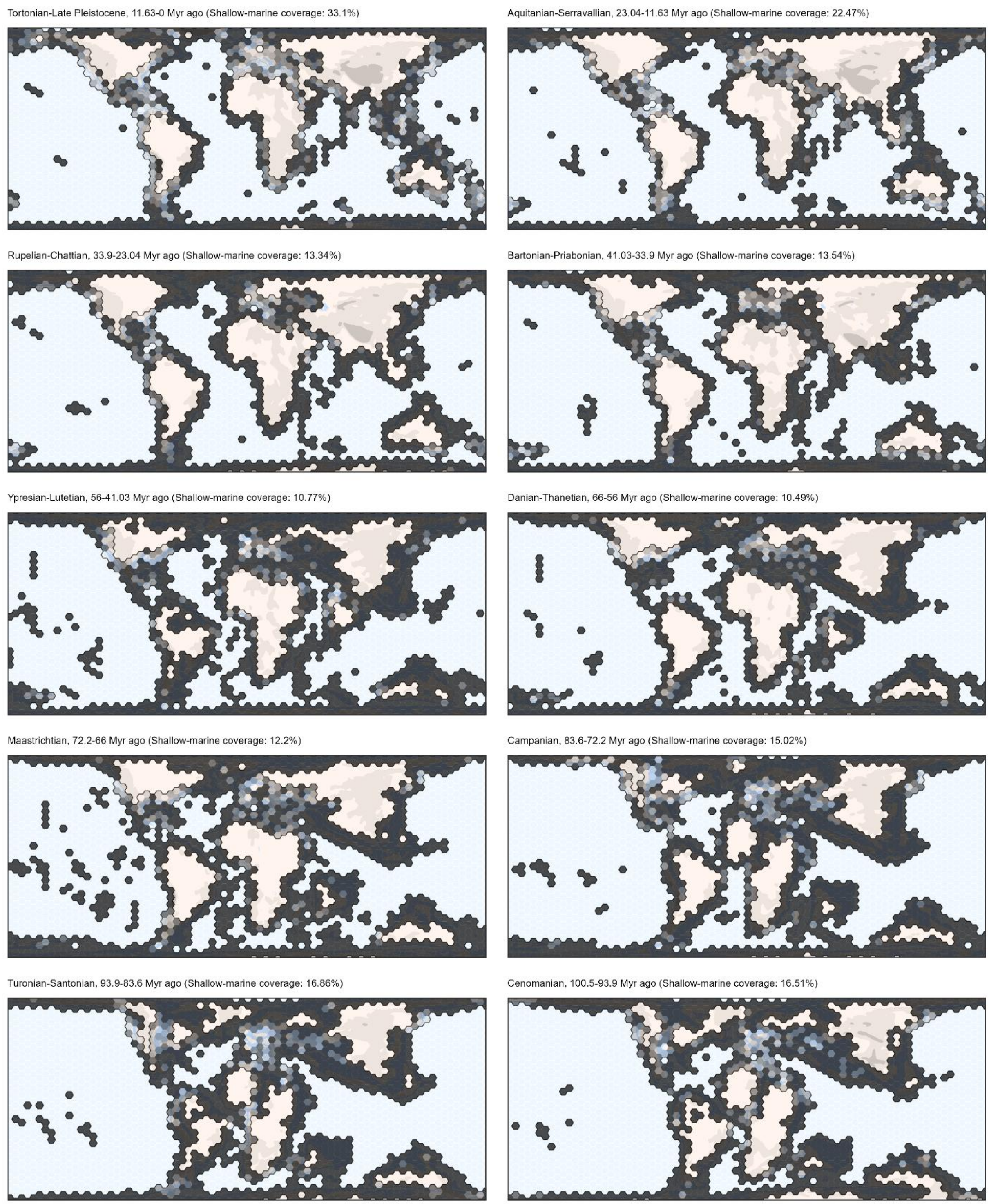

Fig. S27.

Paleogeographic maps for the 49-bin approach showing information density on shallow-marine metazoans as light hexagons for the 500 km flat-topped grid. The number of fossil collections per grid cell is displayed as relative transparency (see Figure 1 in main text for legend). Corresponding data on coverage is summarized in table S10.

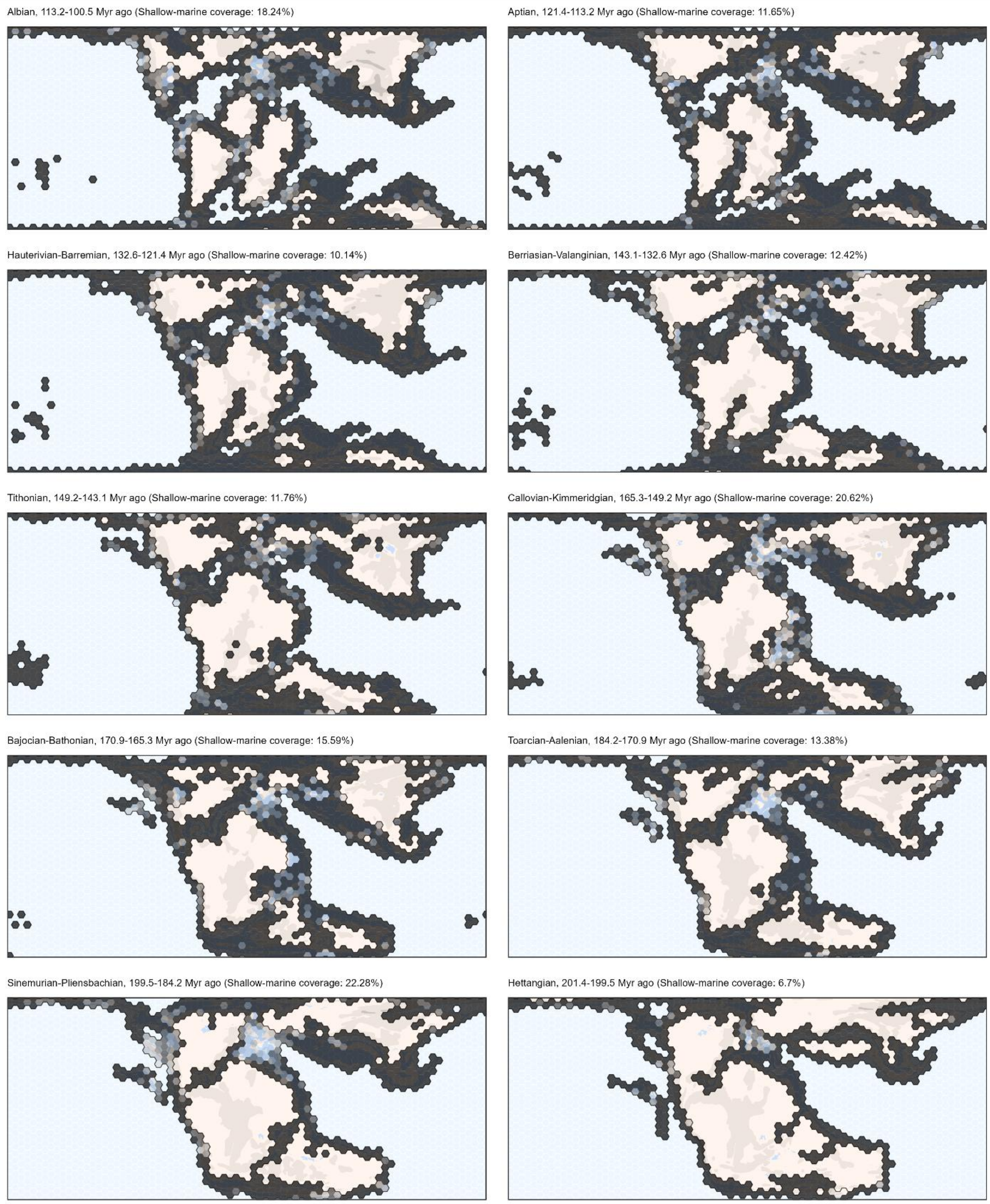

Fig. S27. Continued.

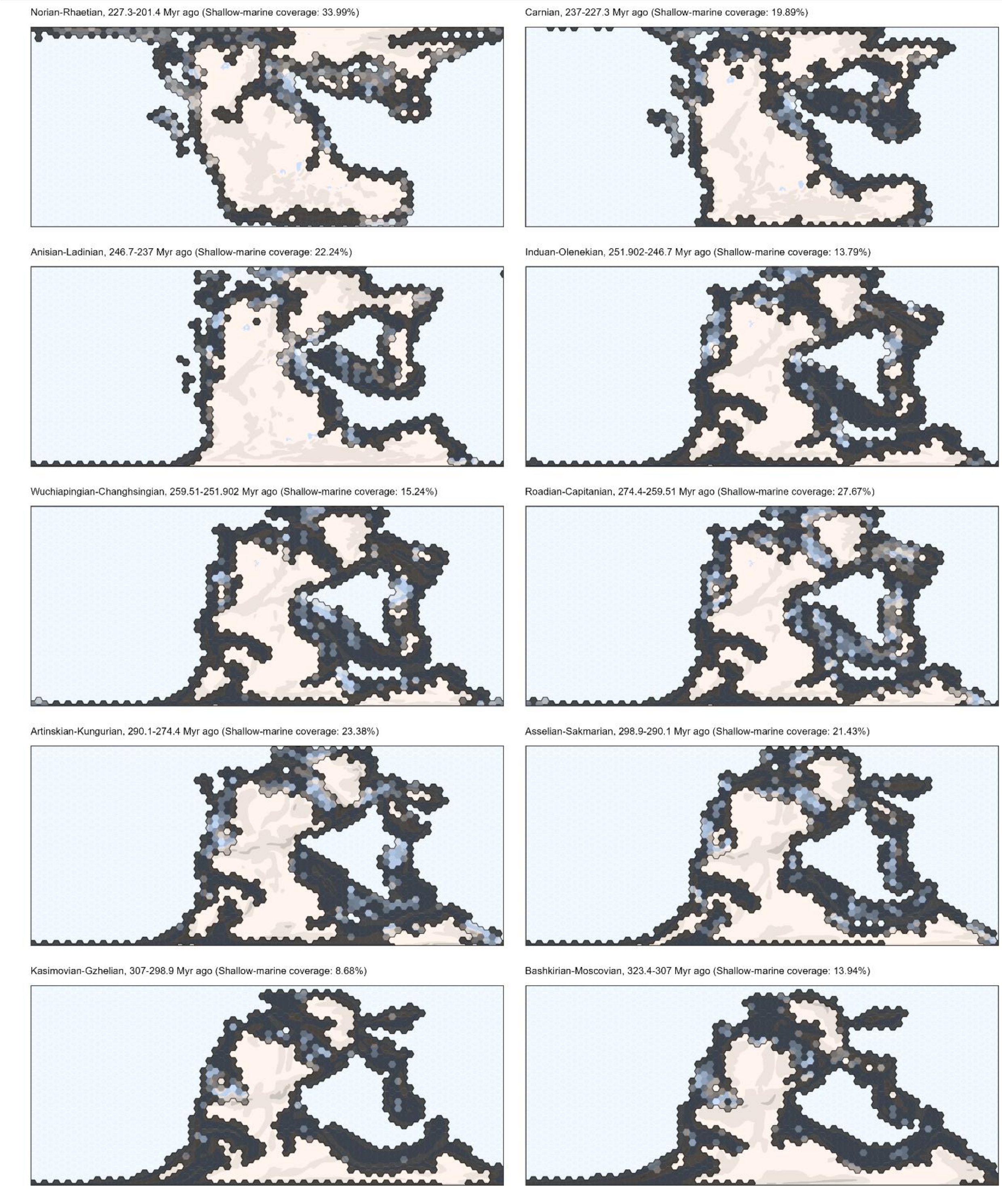

Fig. S27. Continued.

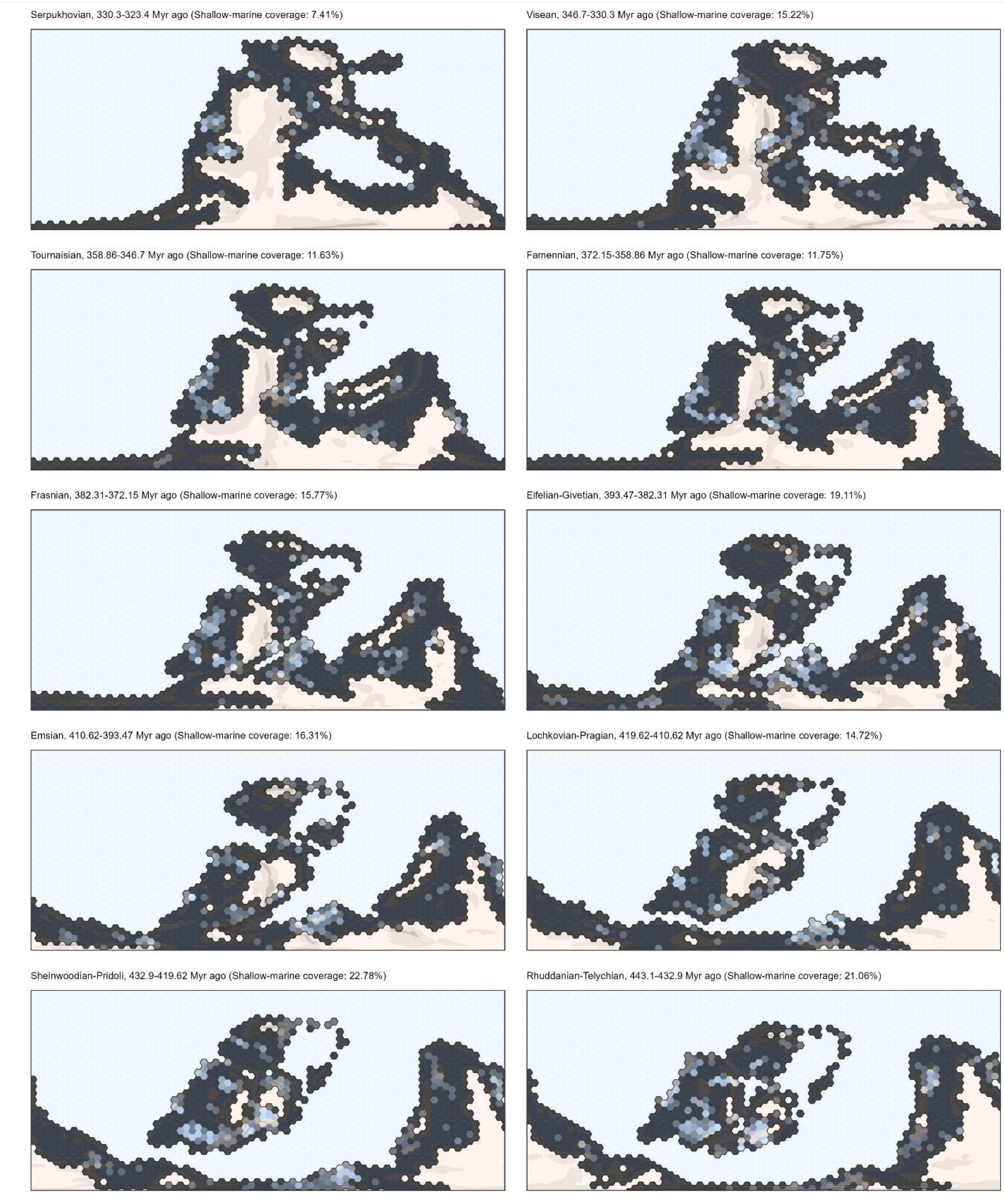

Fig. S27. Continued.

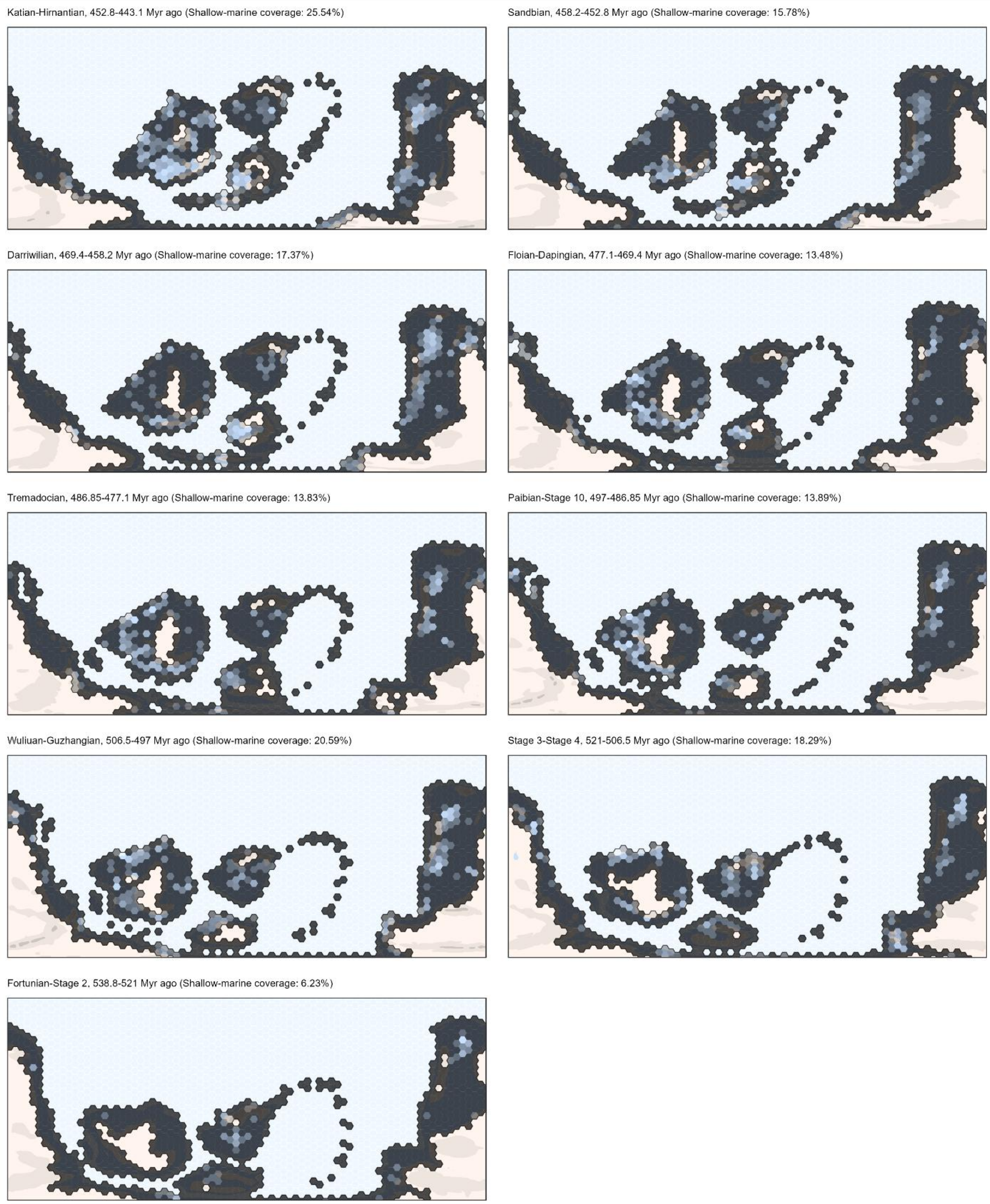

Fig. S27. Continued.

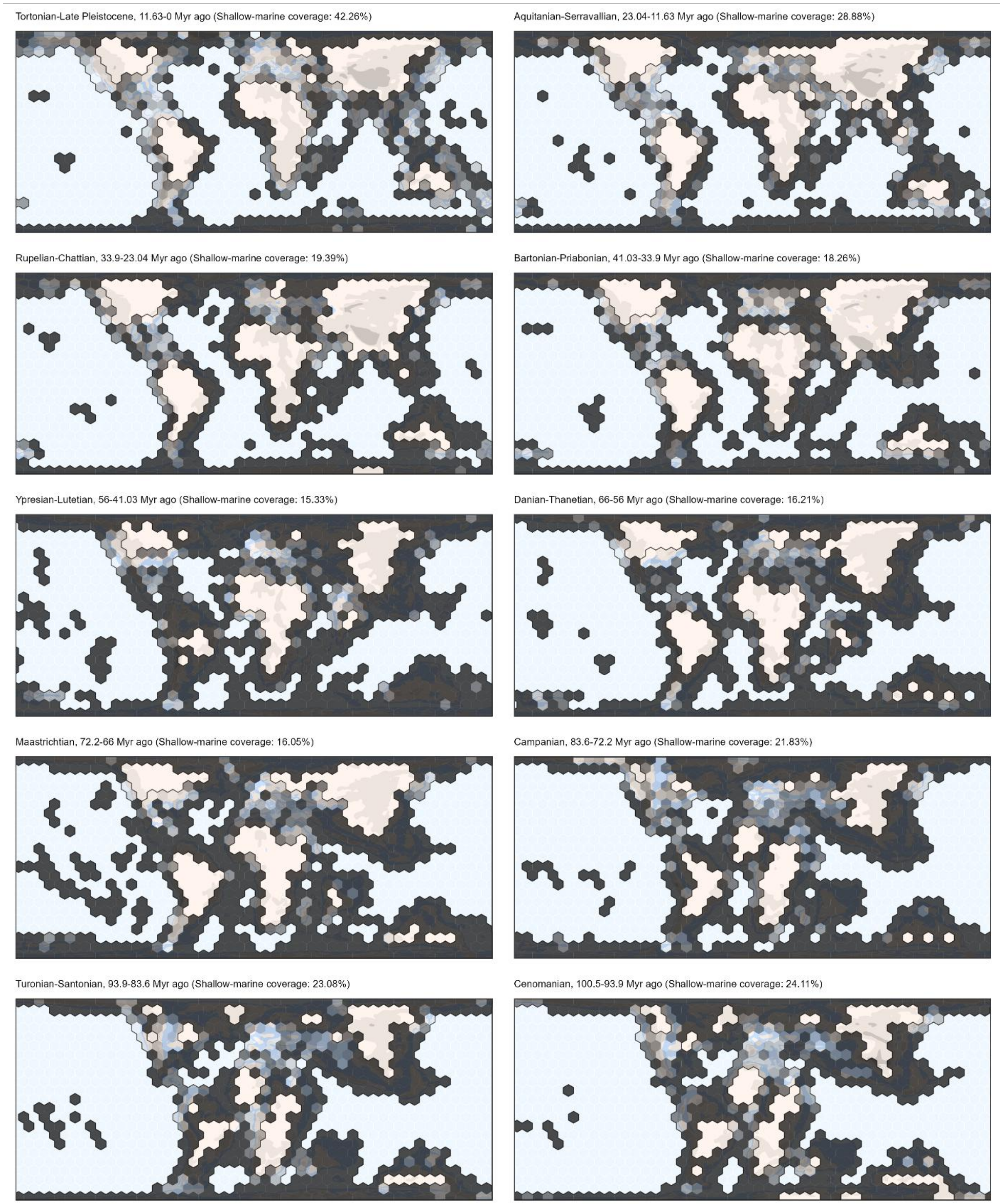

Fig. S28.

Paleogeographic maps for the 49-bin approach showing information density on shallow-marine metazoans as light hexagons for the 750 km pointy-topped grid. The number of fossil collections per grid cell is displayed as relative transparency (see Figure 1 in main text for legend). Corresponding data on coverage is summarized in table S10.

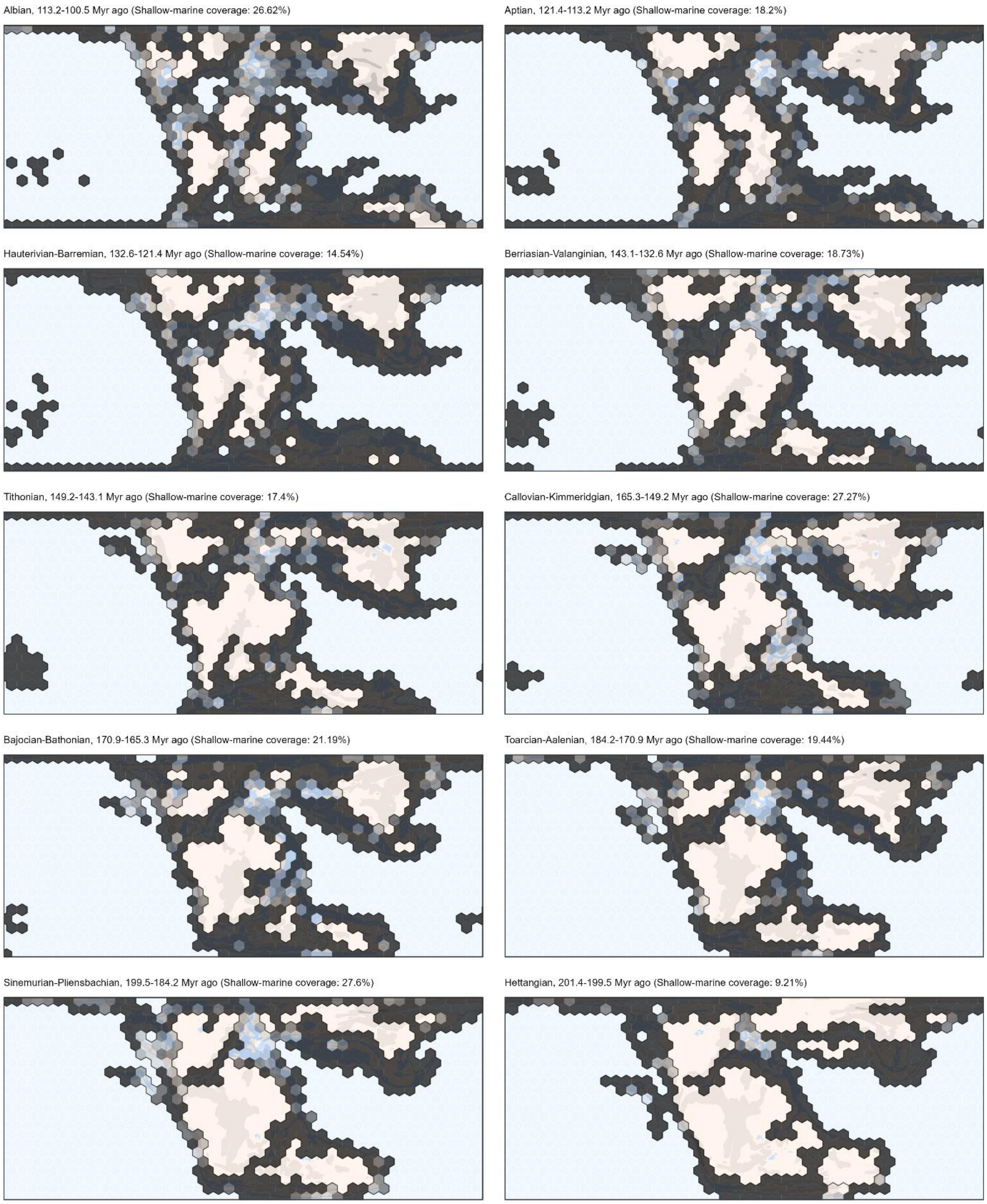

Fig. S28. Continued.

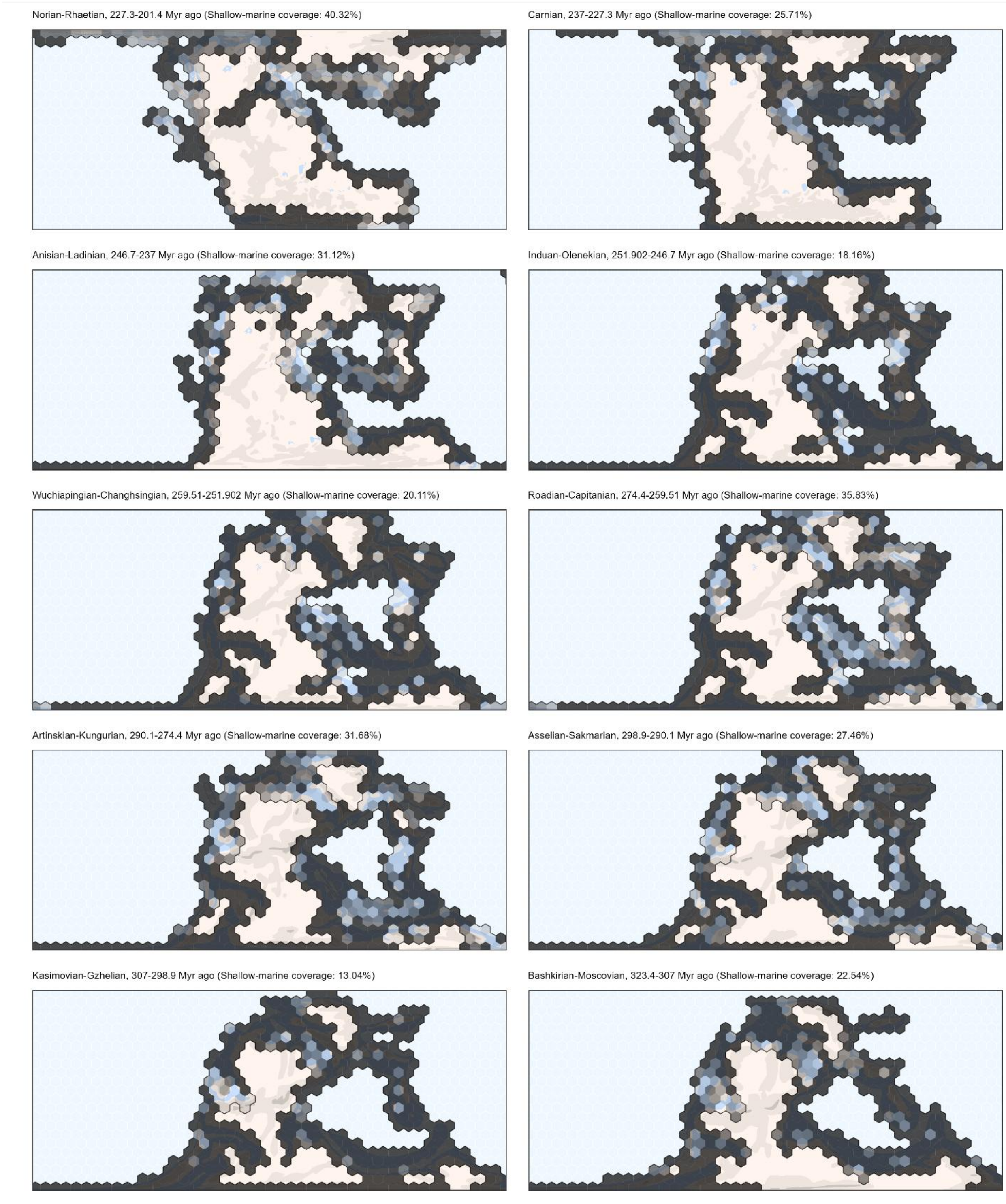

Fig. S28. Continued.

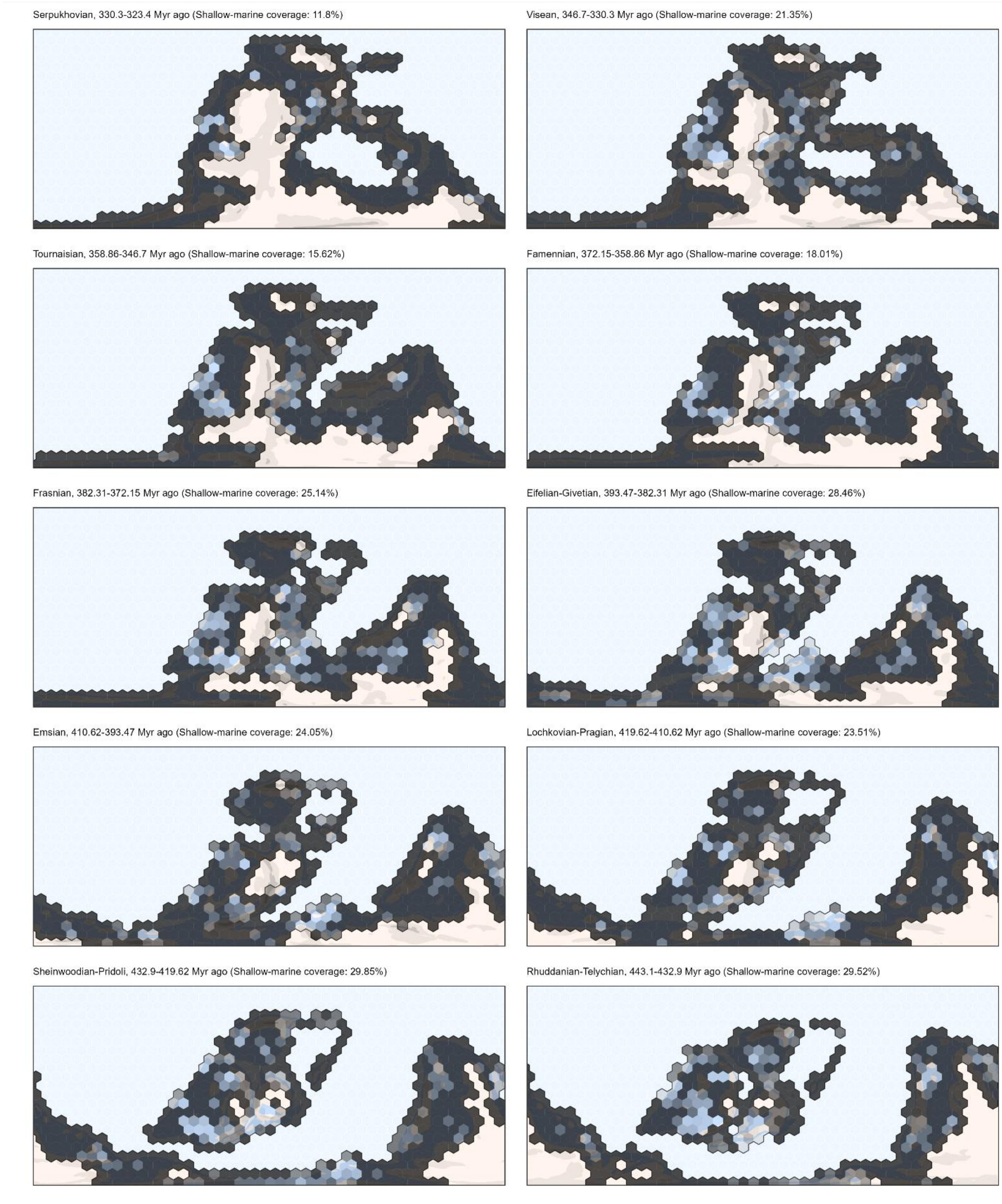

Fig. S28. Continued.

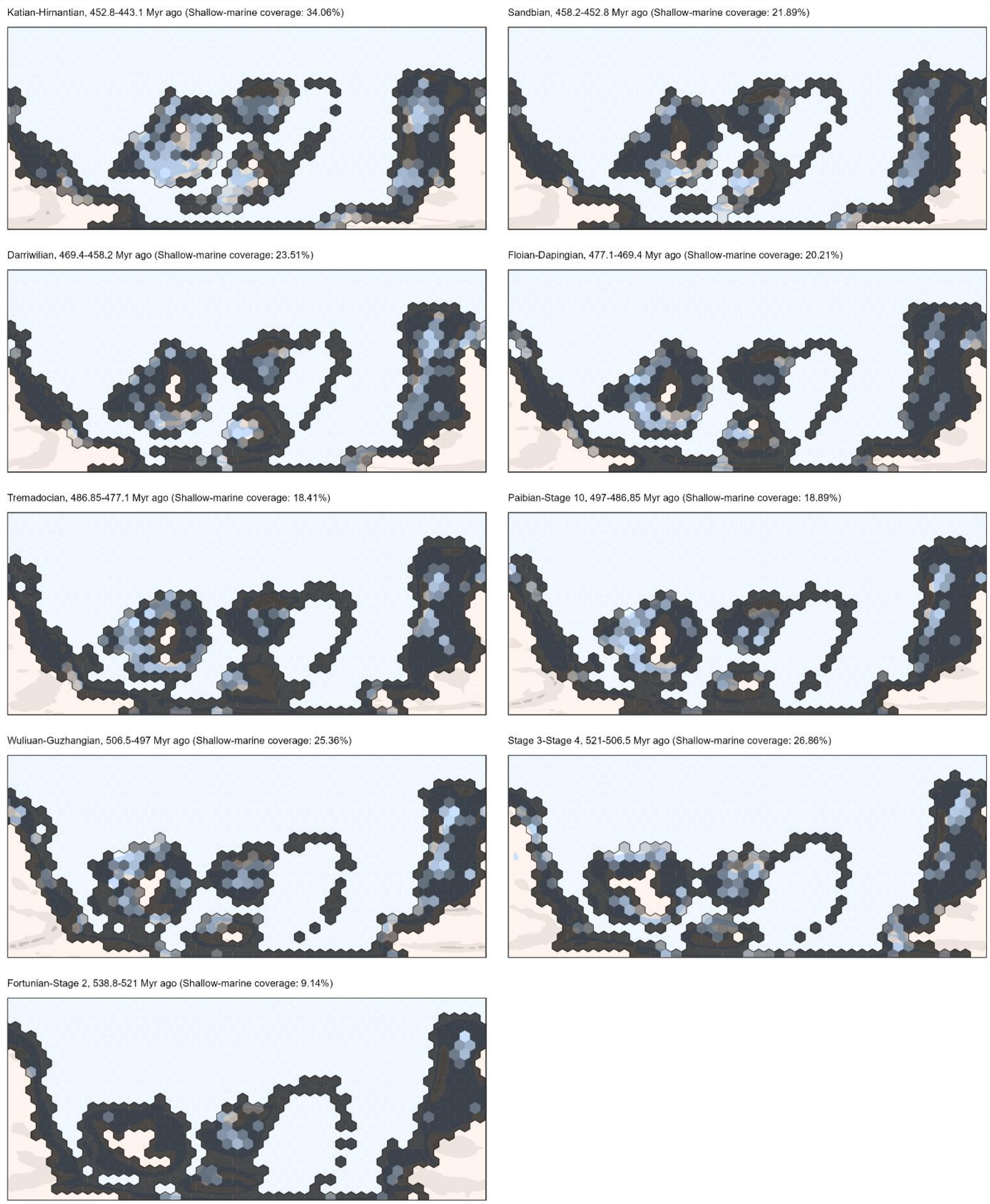

Fig. S28. Continued.

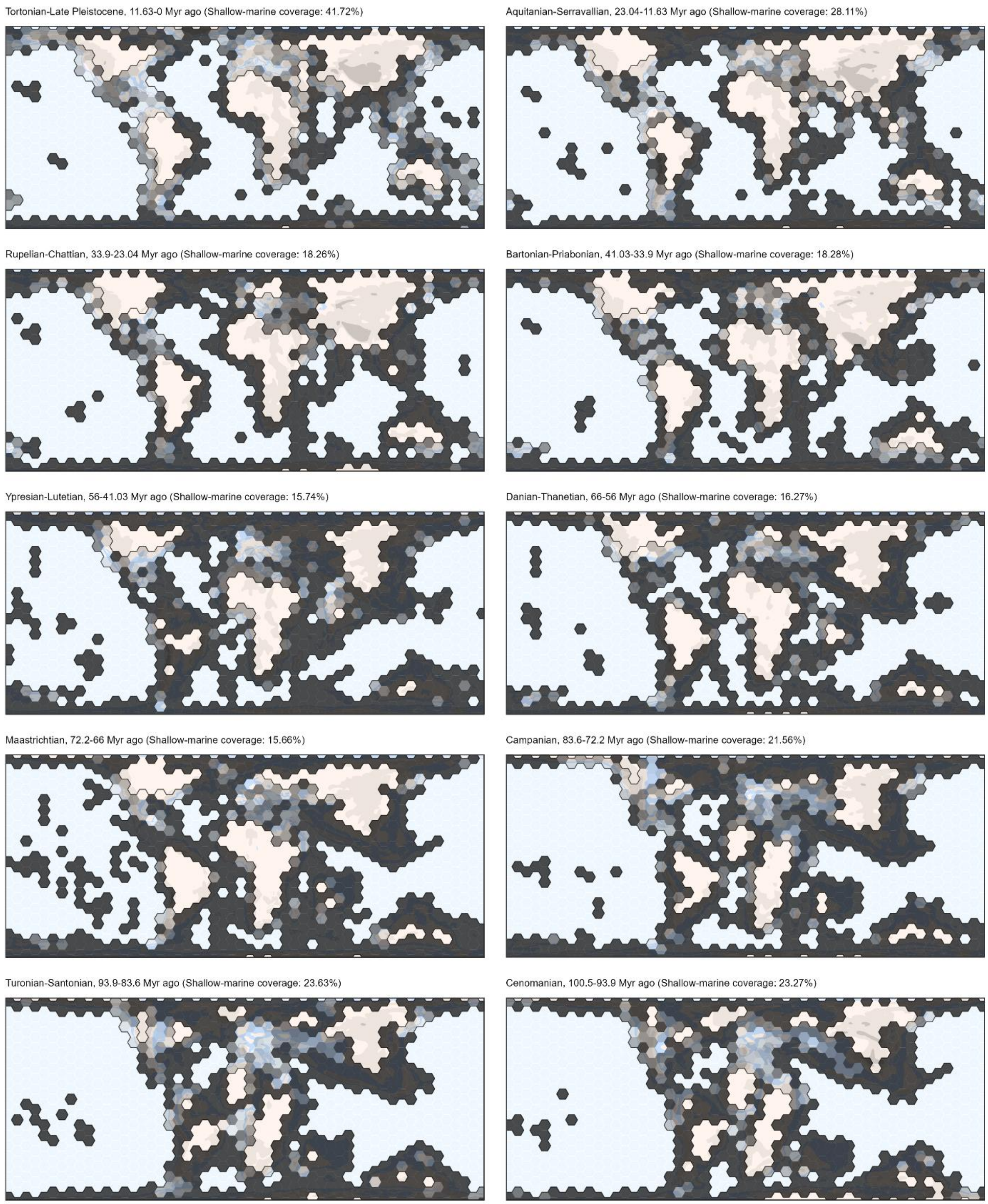

Fig. S29.

Paleogeographic maps for the 49-bin approach showing information density on shallow-marine metazoans as light hexagons for the 750 km flat-topped grid. The number of fossil collections per grid cell is displayed as relative transparency (see Figure 1 in main text for legend). Corresponding data on coverage is summarized in table S10.

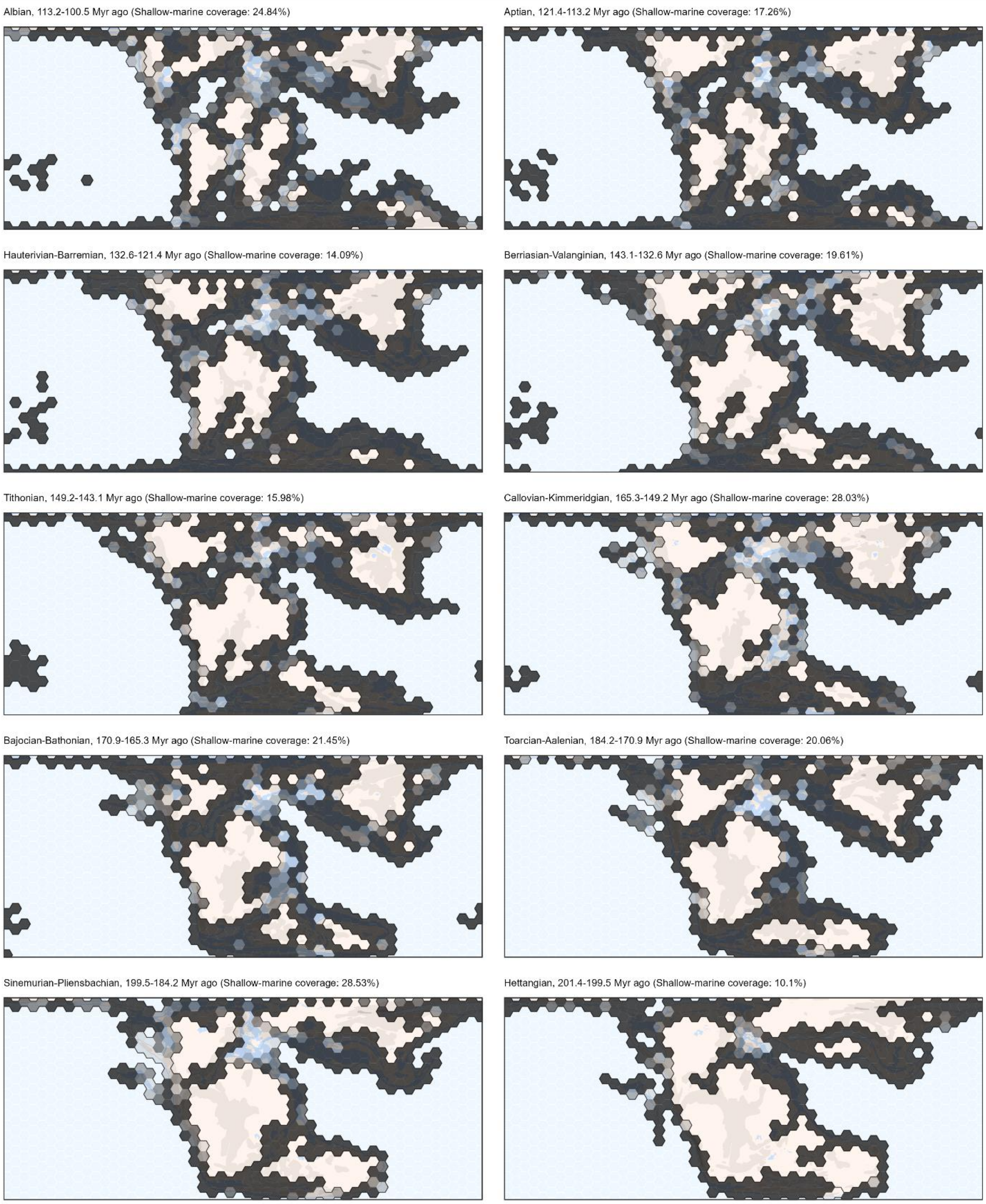

Fig. S29. Continued.

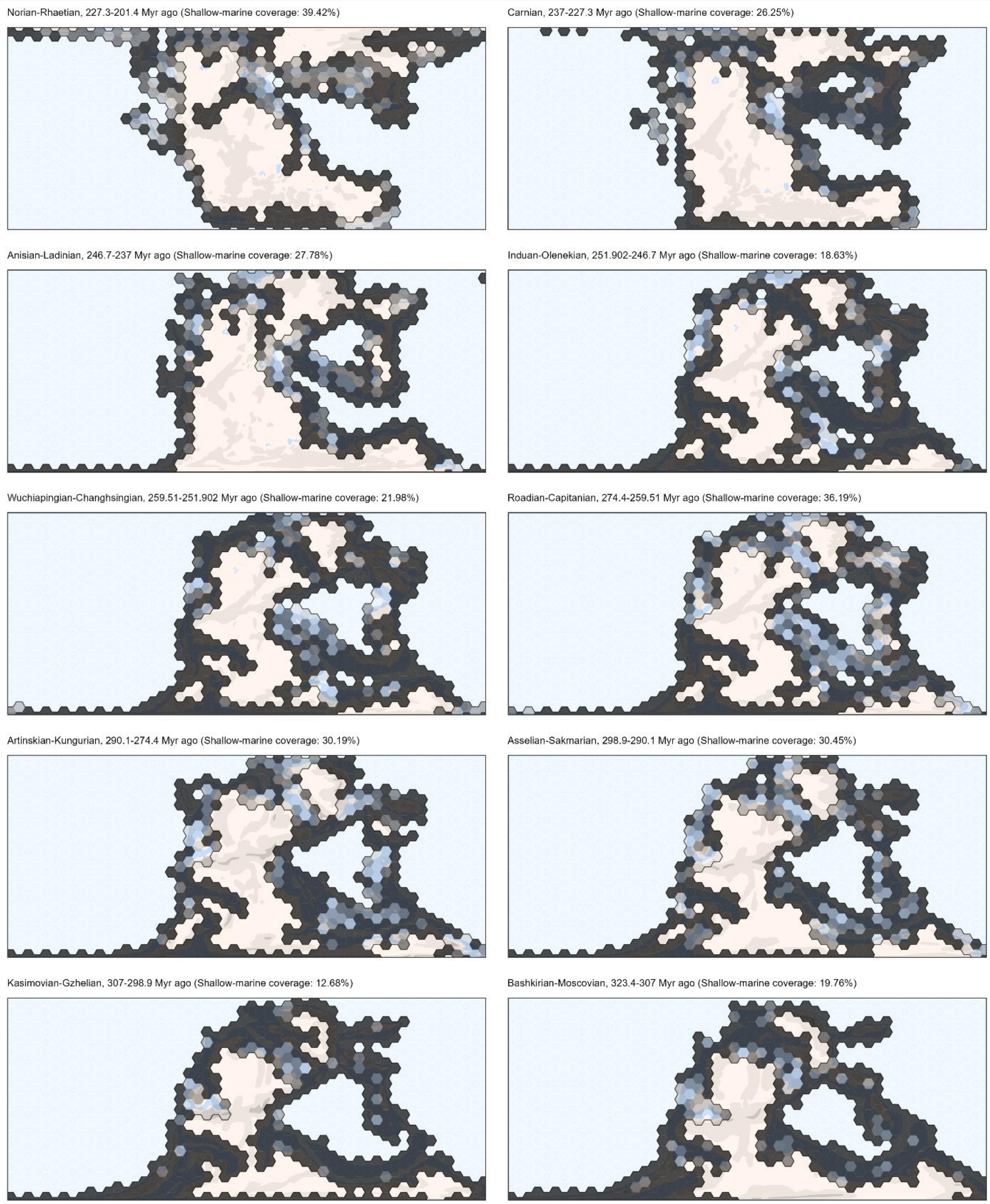

Fig. S29. Continued.

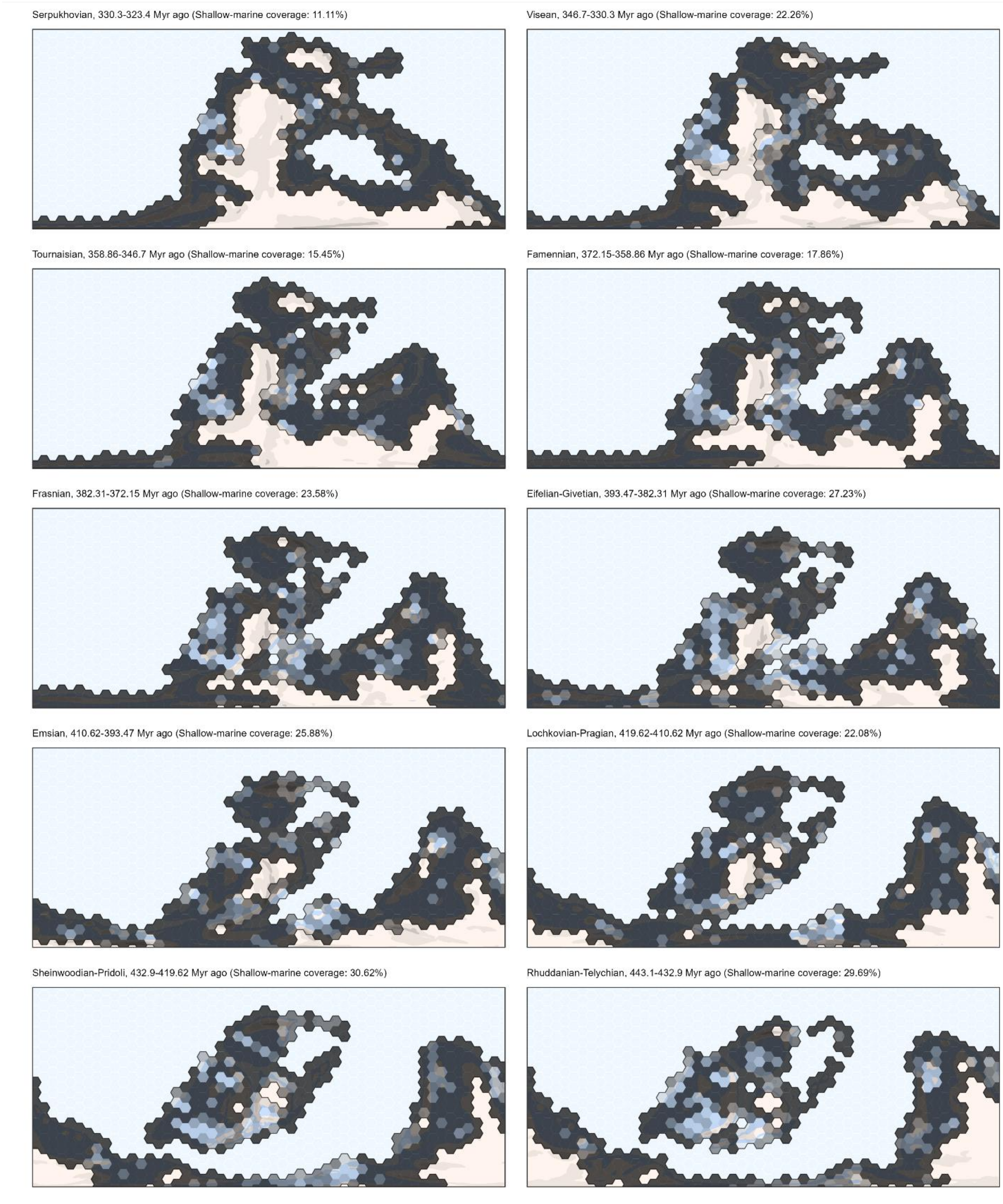

Fig. S29. Continued.

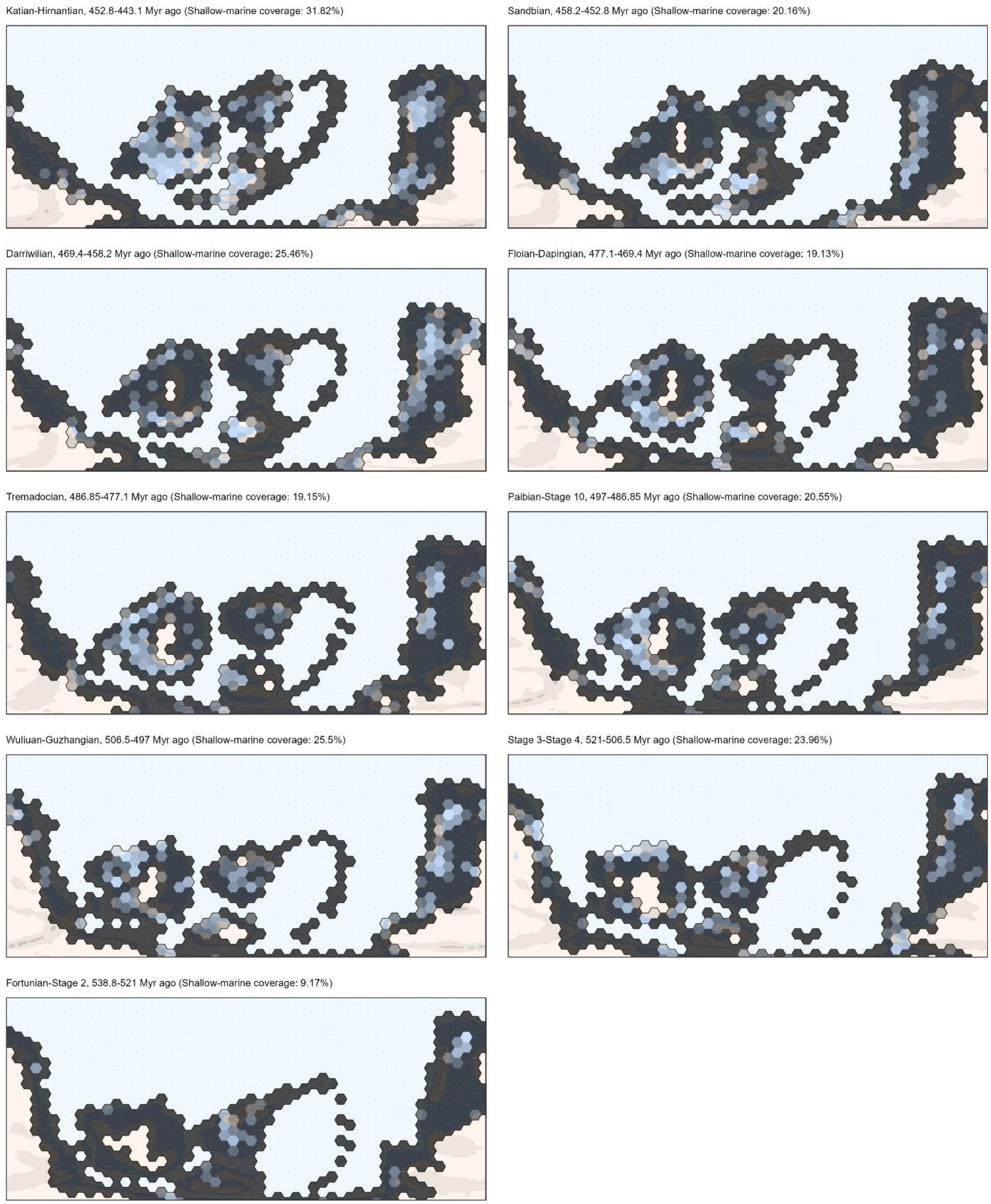

Fig. S29. Continued.

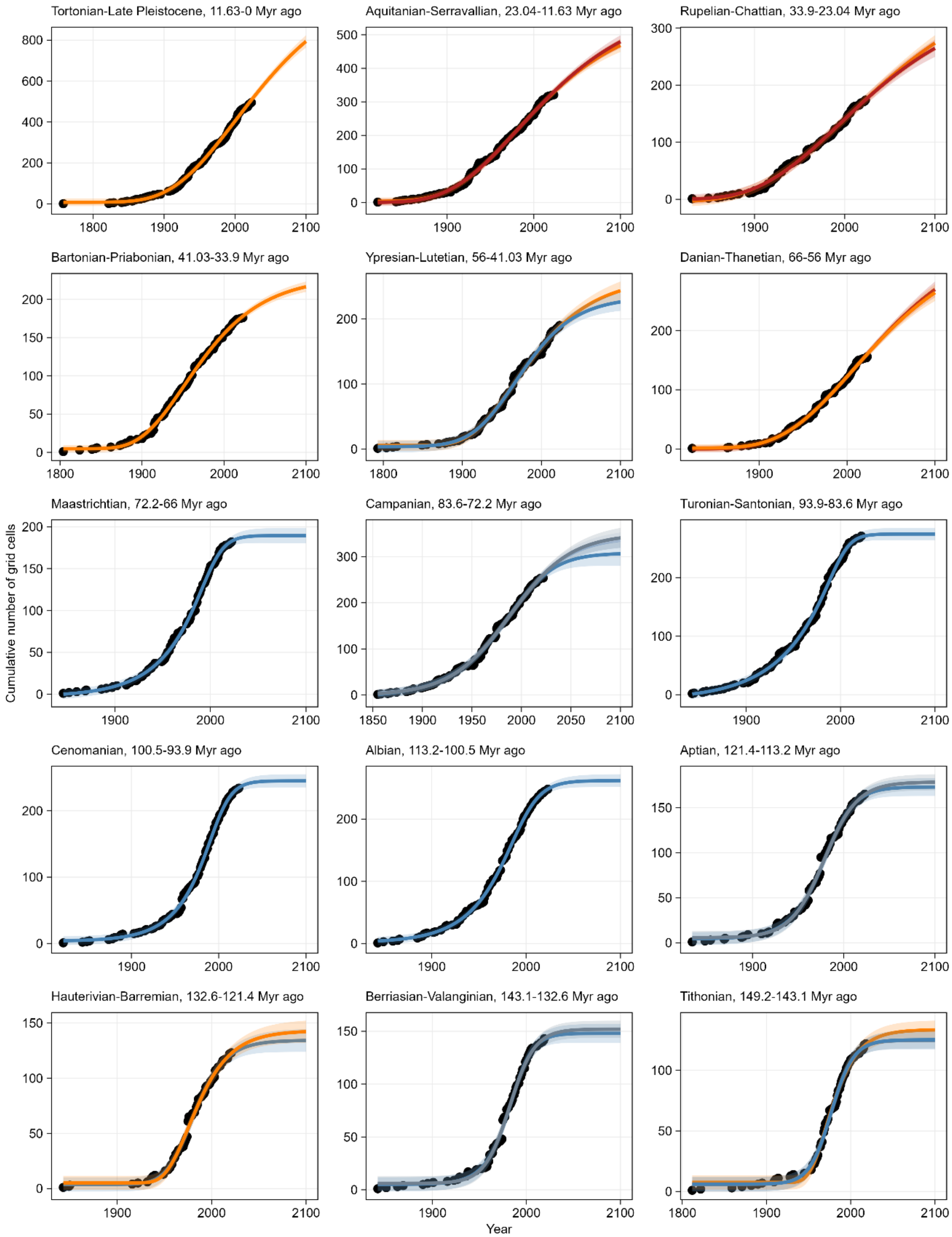

Fig. S30.

Growth models for the 49-bin approach predicting future collection effort across grid cells until the year 2100 for each time bin for the 250 km pointy-topped grid. Note that for some bins, more than one model qualified as the best (i.e., within ΔAIC = 2). Underlying data is summarized in table S11.

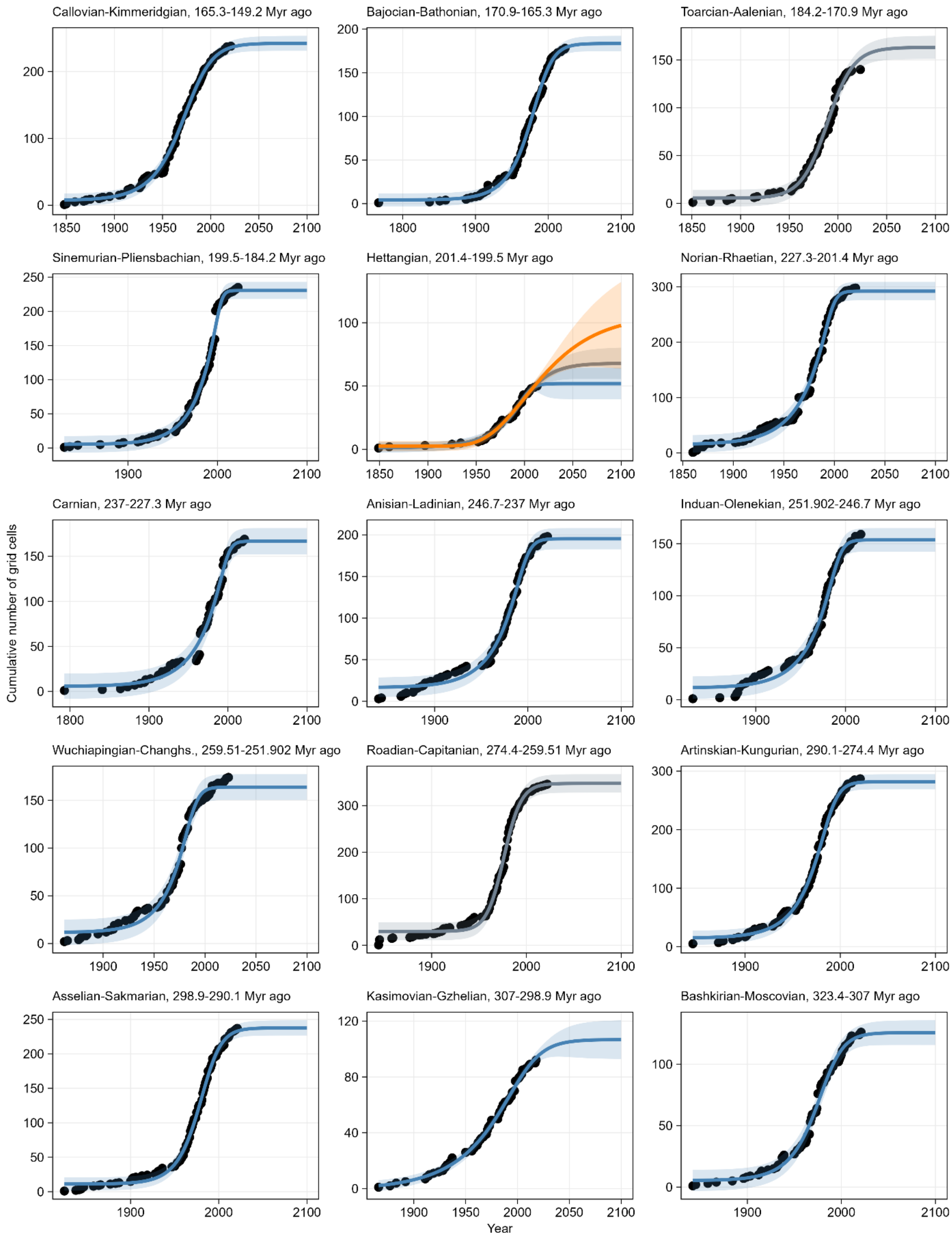

Fig. S30. Continued.

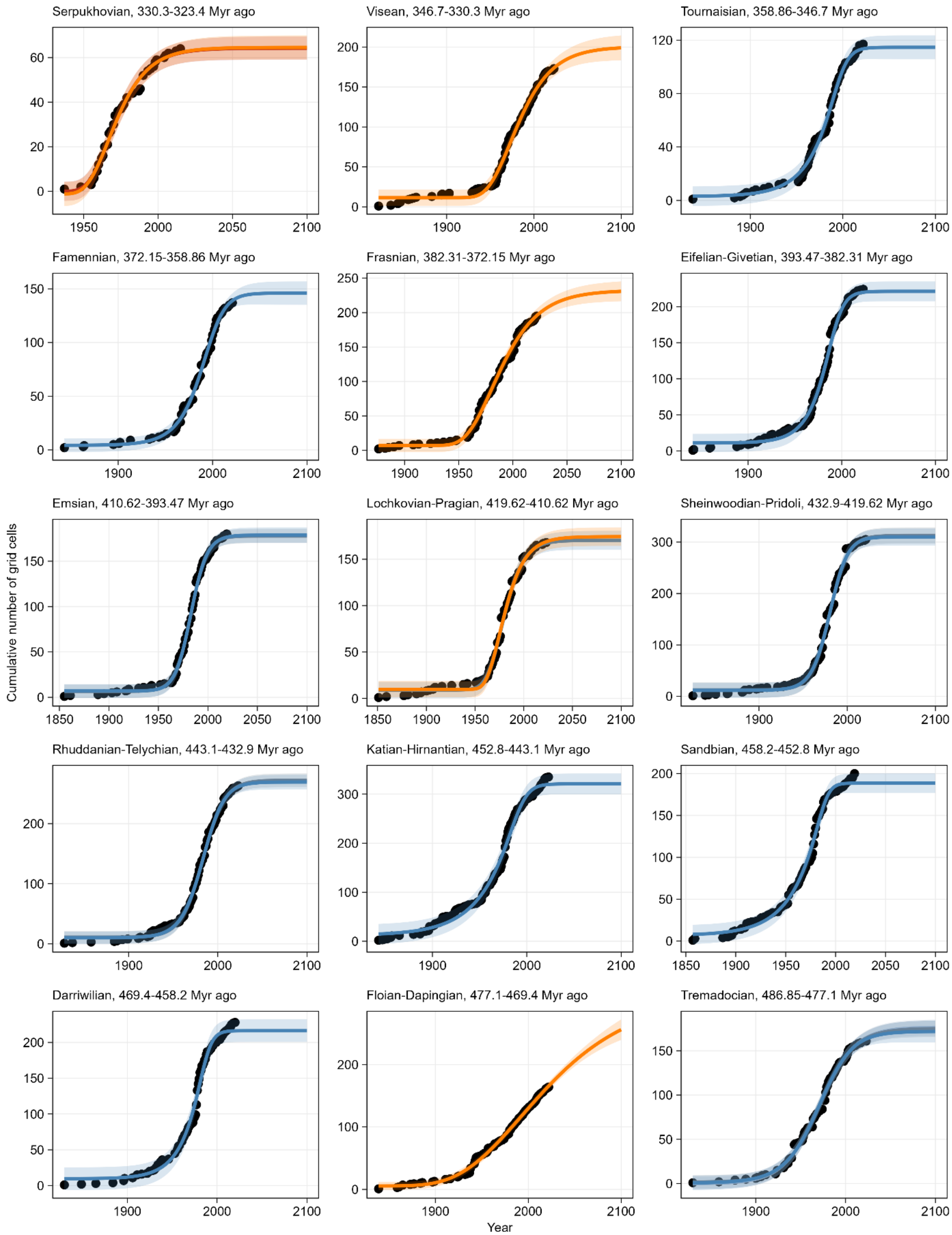

Fig. S30. Continued.

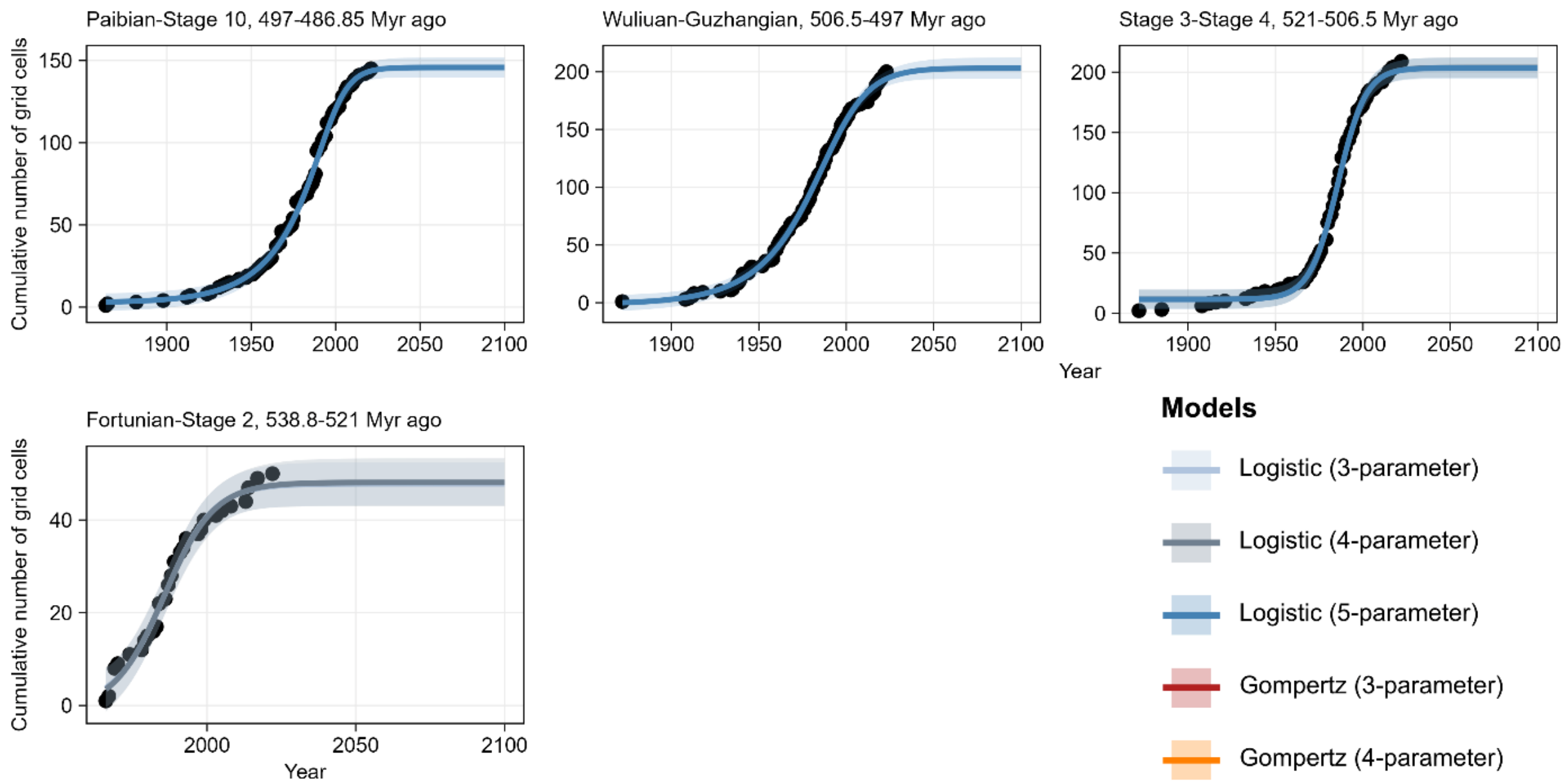

Fig. S30. Continued.

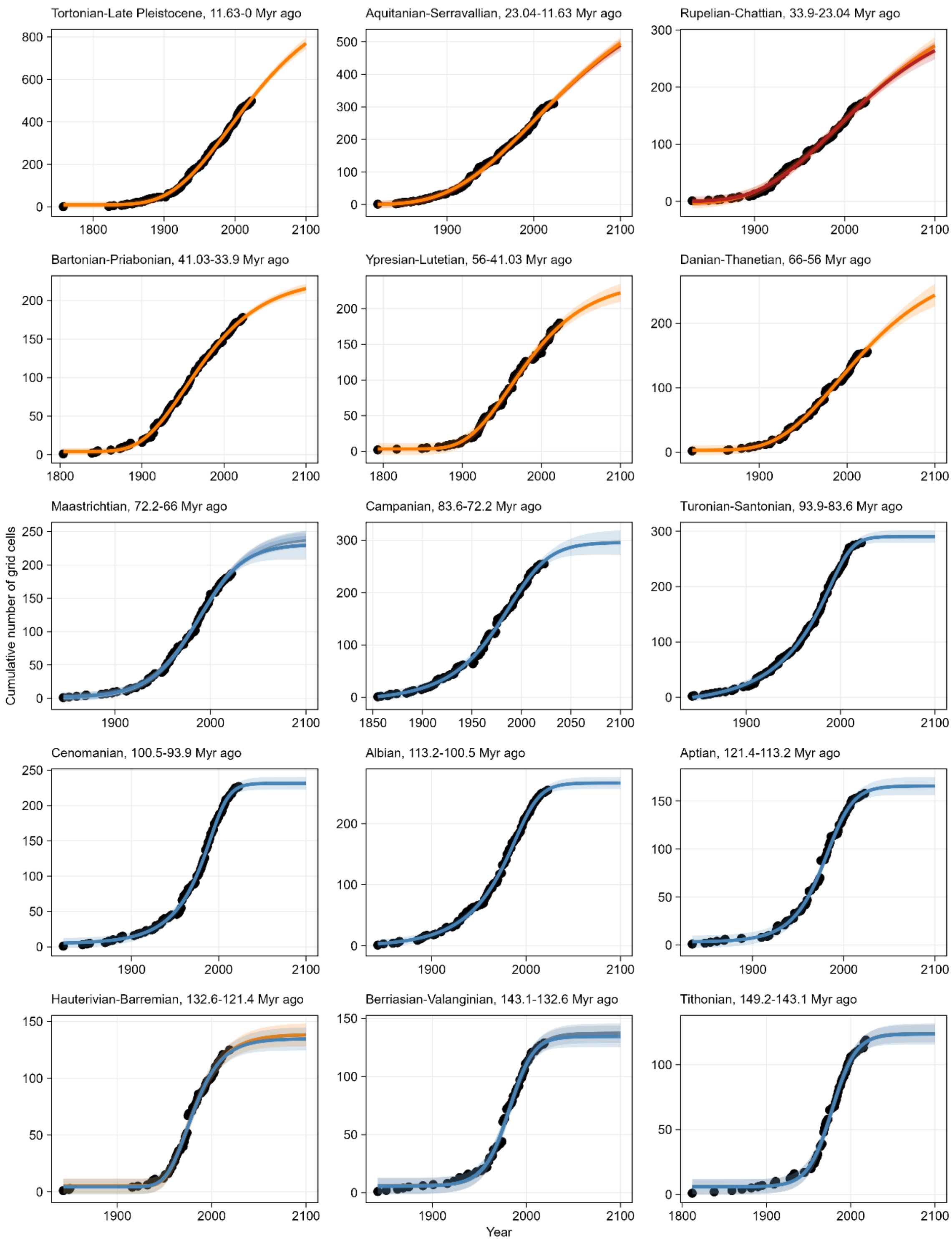

Fig. S31.

Growth models for the 49-bin approach predicting future collection effort across grid cells until the year 2100 for each time bin for the 250 km flat-topped grid. Note that for some bins, more than one model qualified as the best (i.e., within ΔAIC = 2). Underlying data is summarized in table S11.

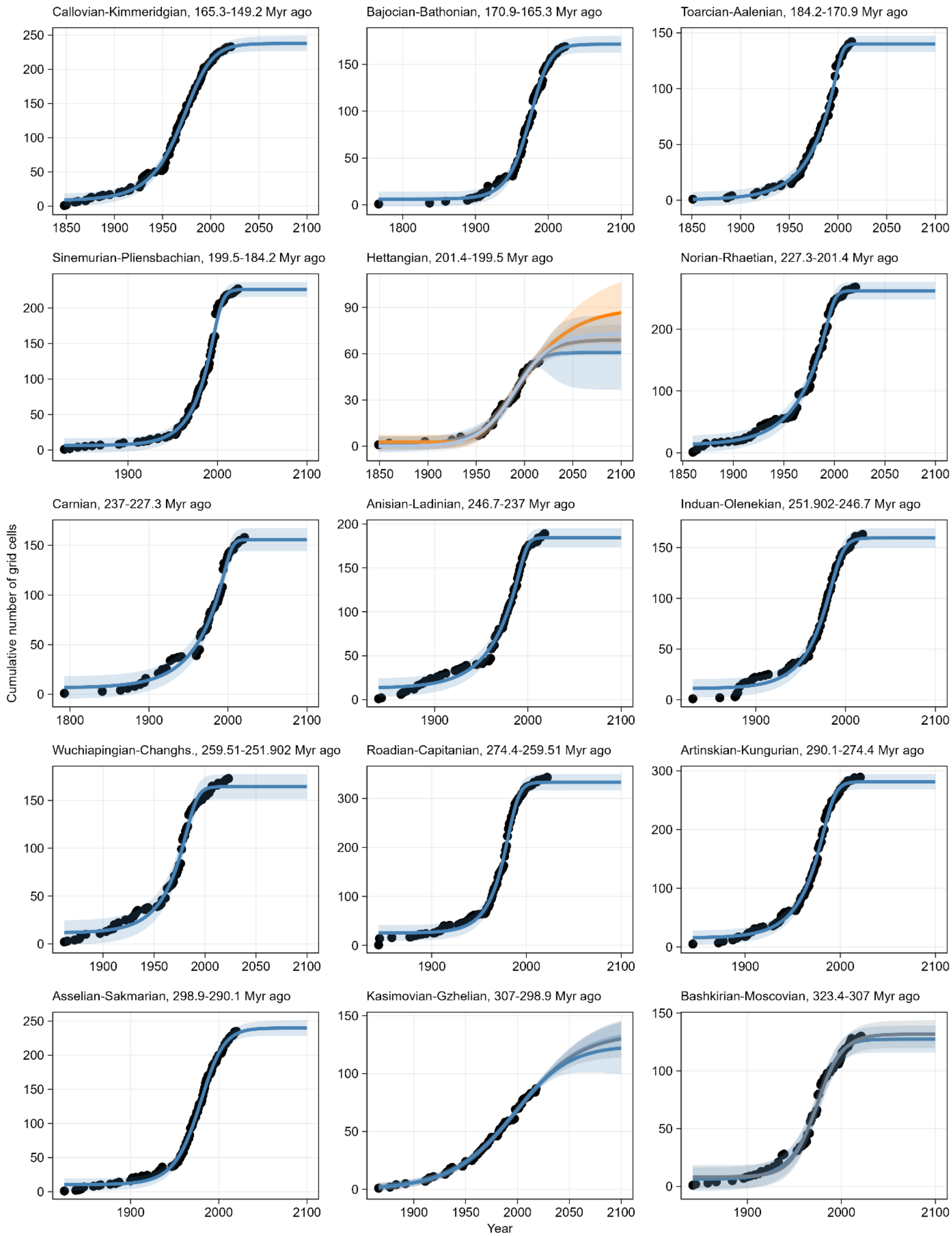

Fig. S31. Continued.

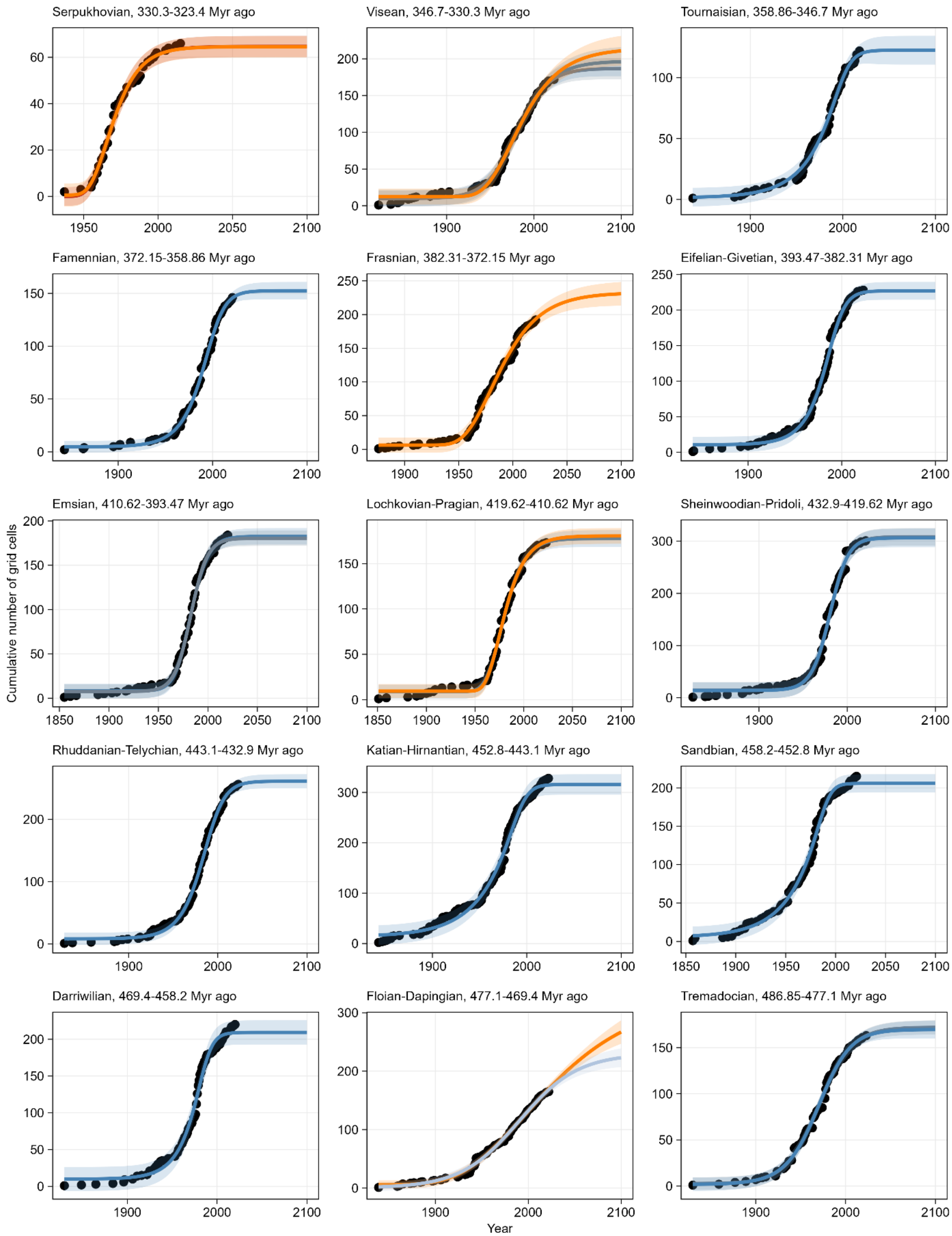

Fig. S31. Continued.

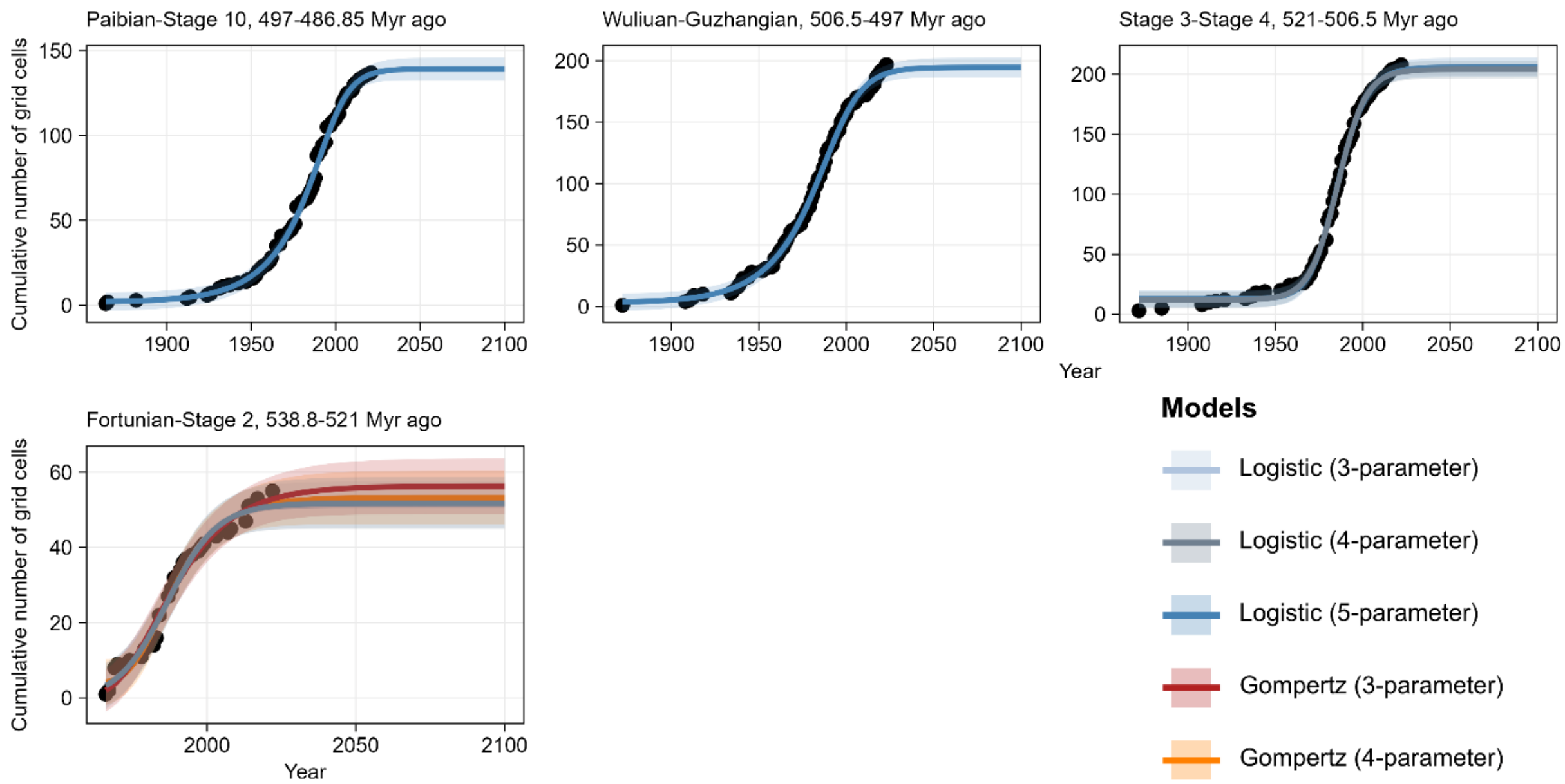

Fig. S31. Continued.

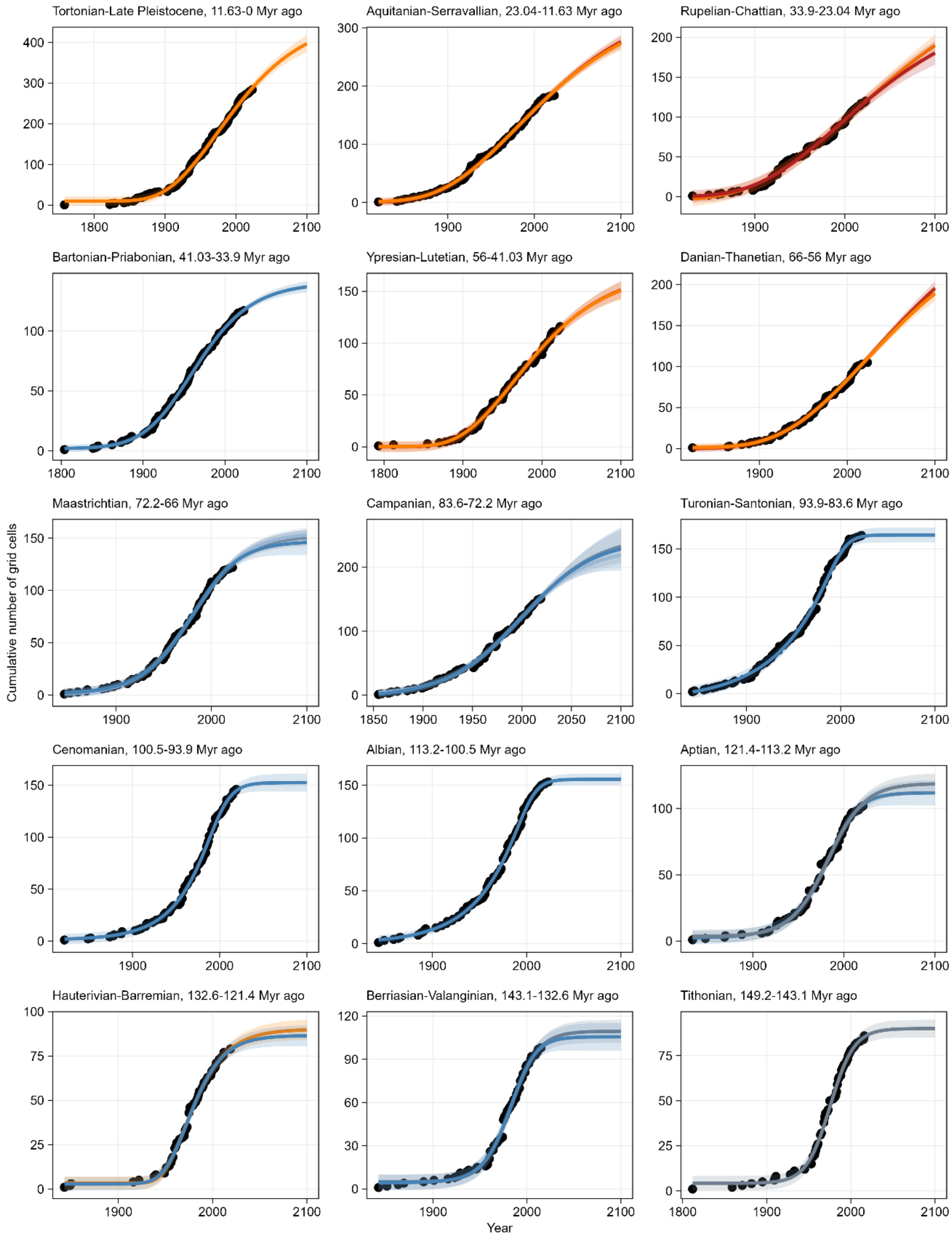

Fig. S32.

Growth models for the 49-bin approach predicting future collection effort across grid cells until the year 2100 for each time bin for the 500 km pointy-topped grid. Note that for some bins, more than one model qualified as the best (i.e., within ΔAIC = 2). Underlying data is summarized in table S11.

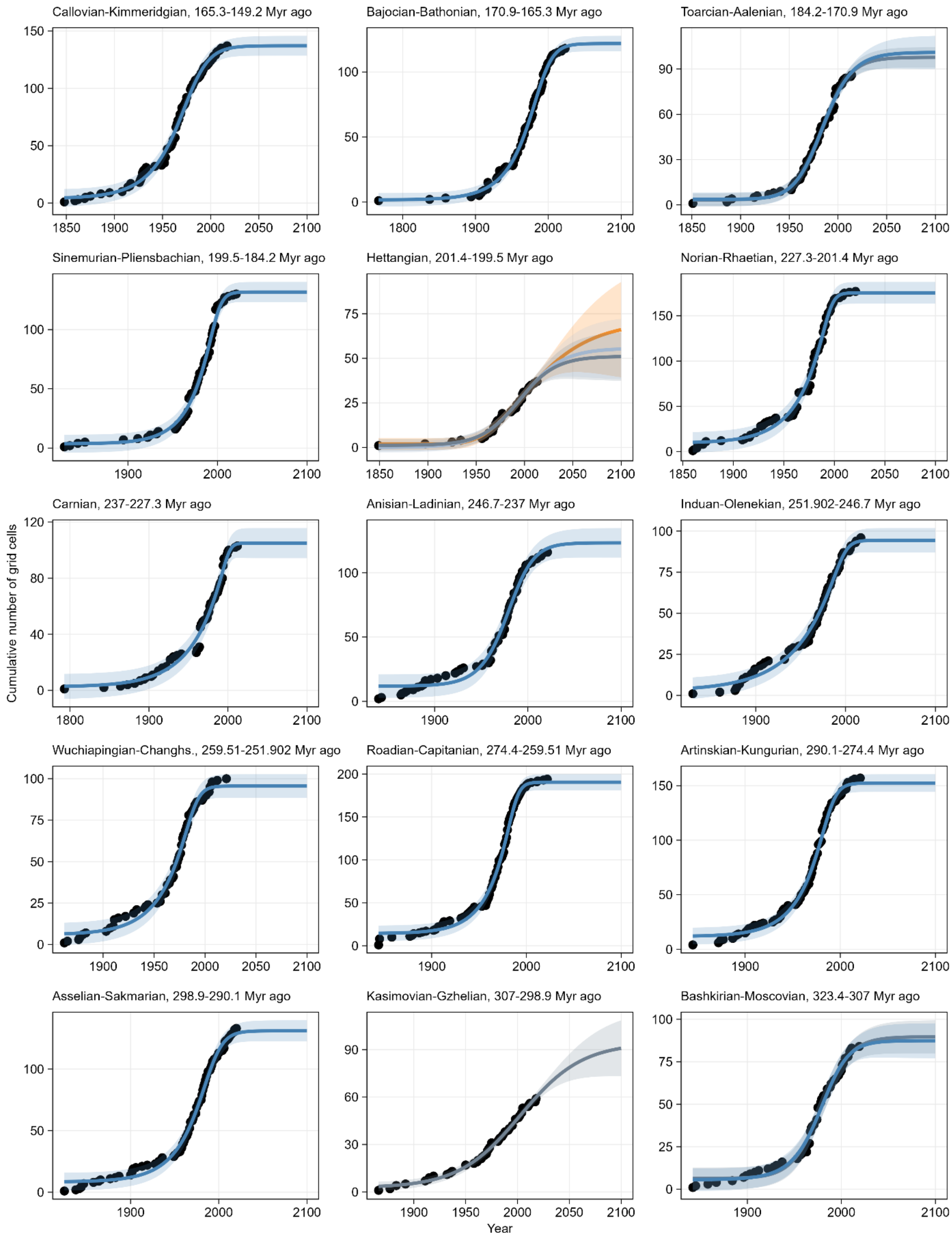

Fig. S32. Continued.

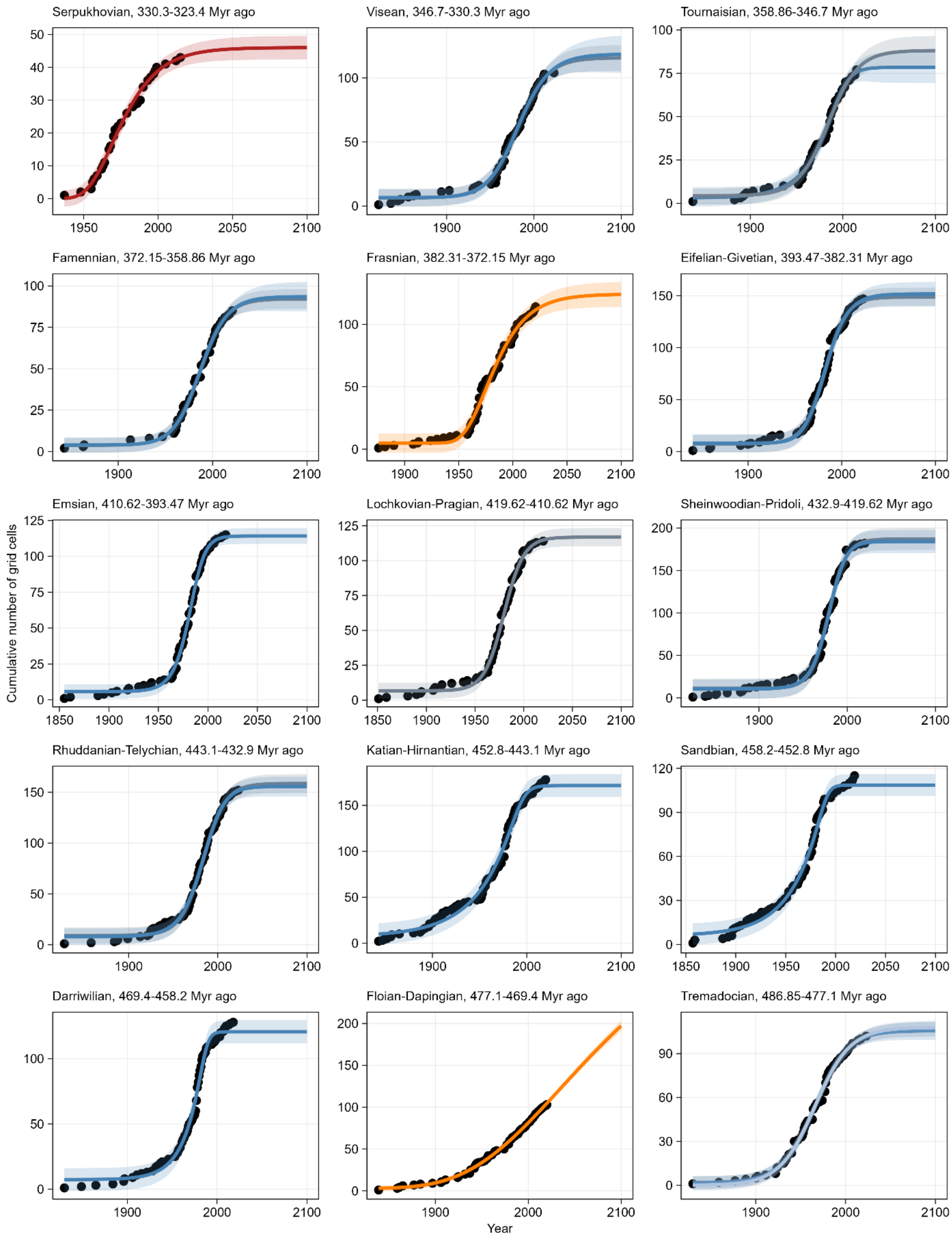

Fig. S32. Continued.

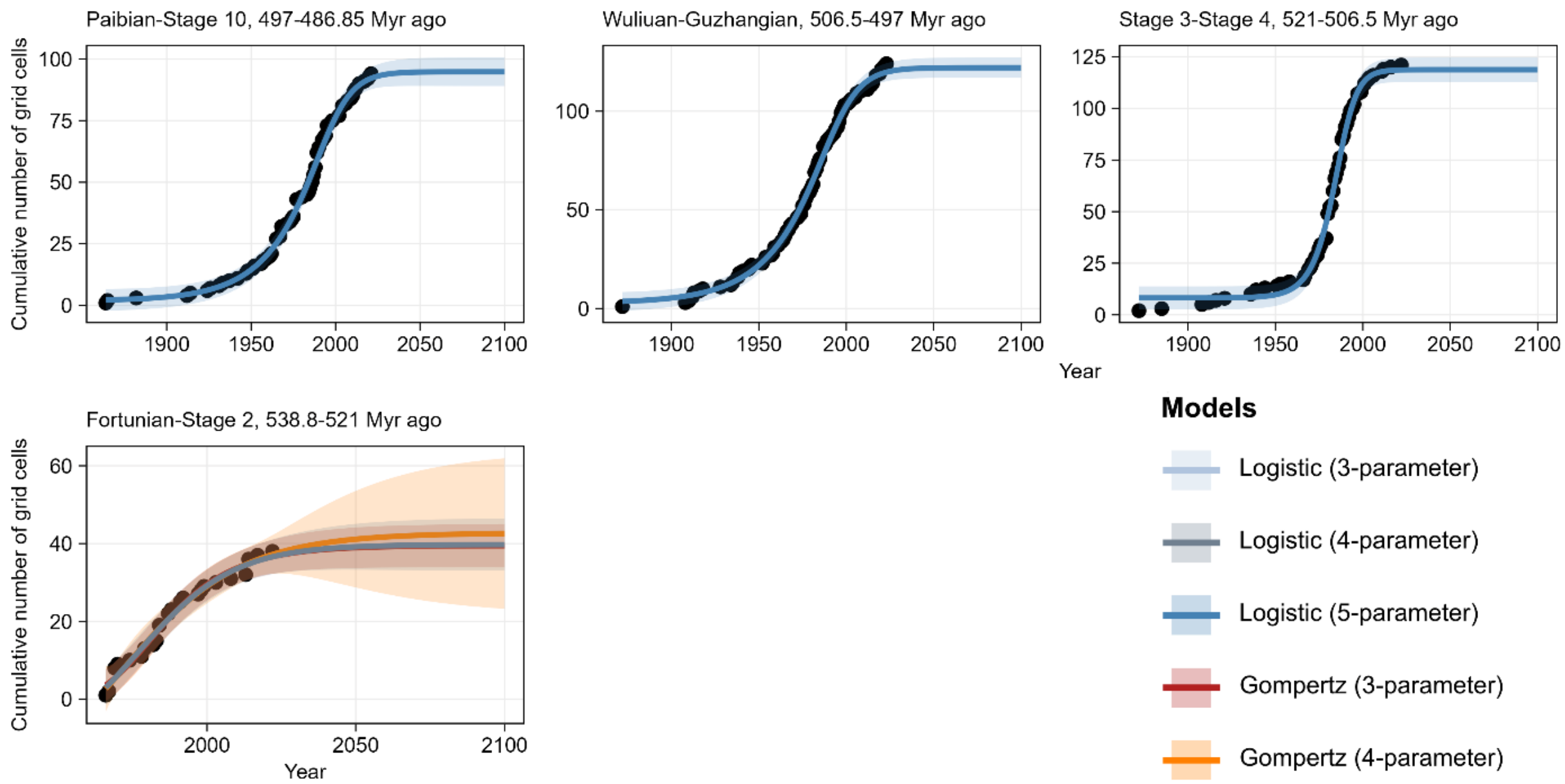

Fig. S32. Continued.

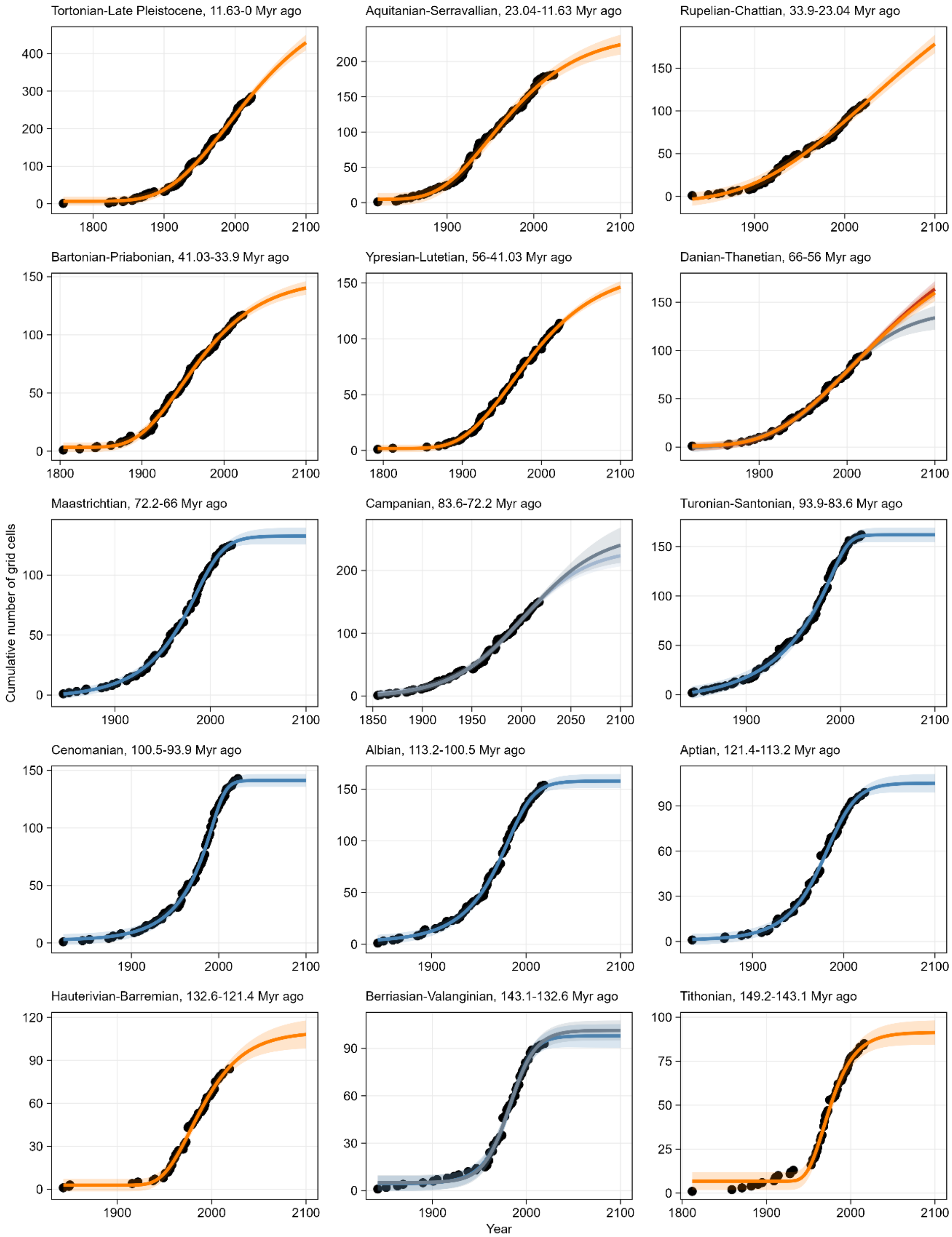

Fig. S33.

Growth models for the 49-bin approach predicting future collection effort across grid cells until the year 2100 for each time bin for the 500 km flat-topped grid. Note that for some bins, more than one model qualified as the best (i.e., within ΔAIC = 2). Underlying data is summarized in table S11.

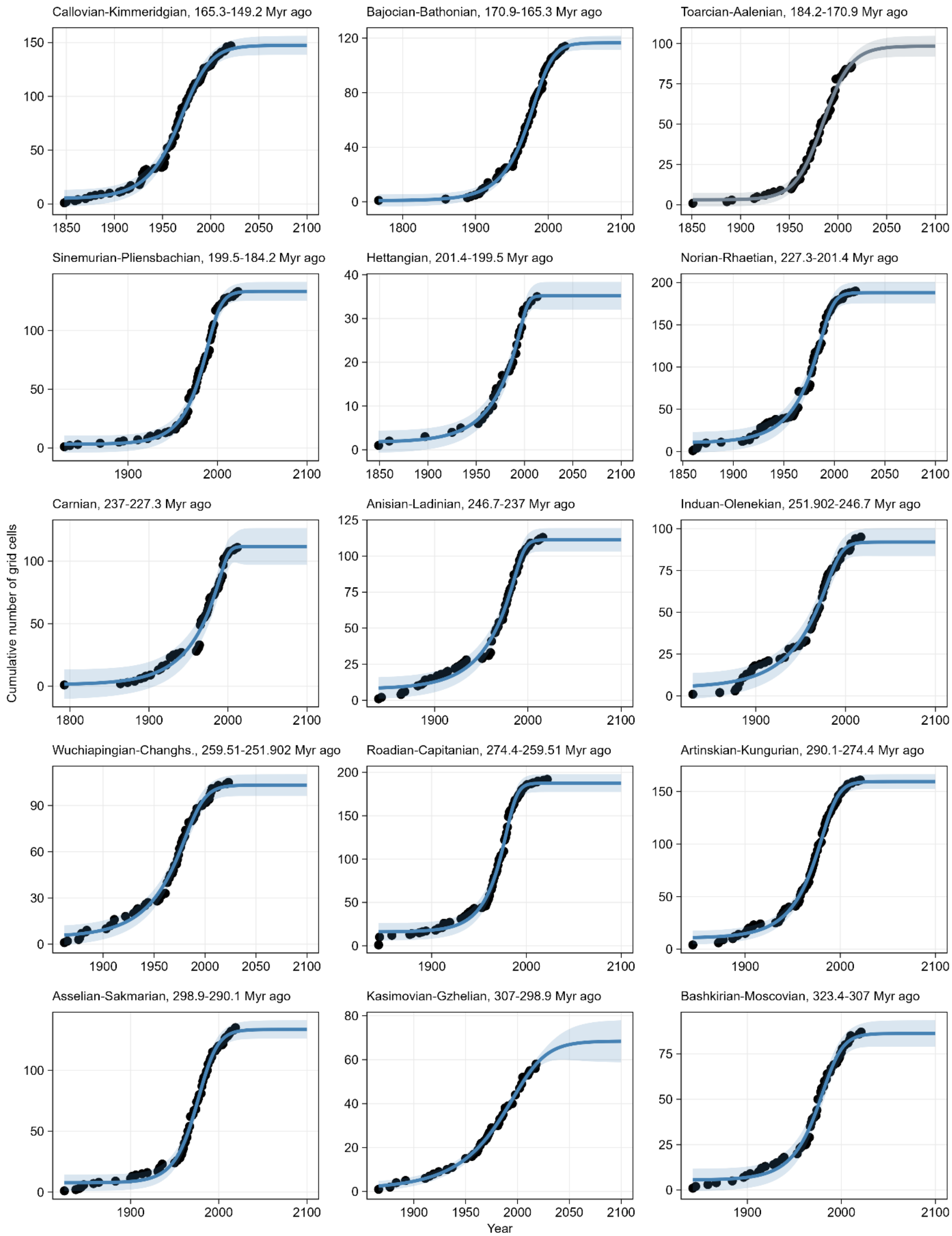

Fig. S33. Continued.

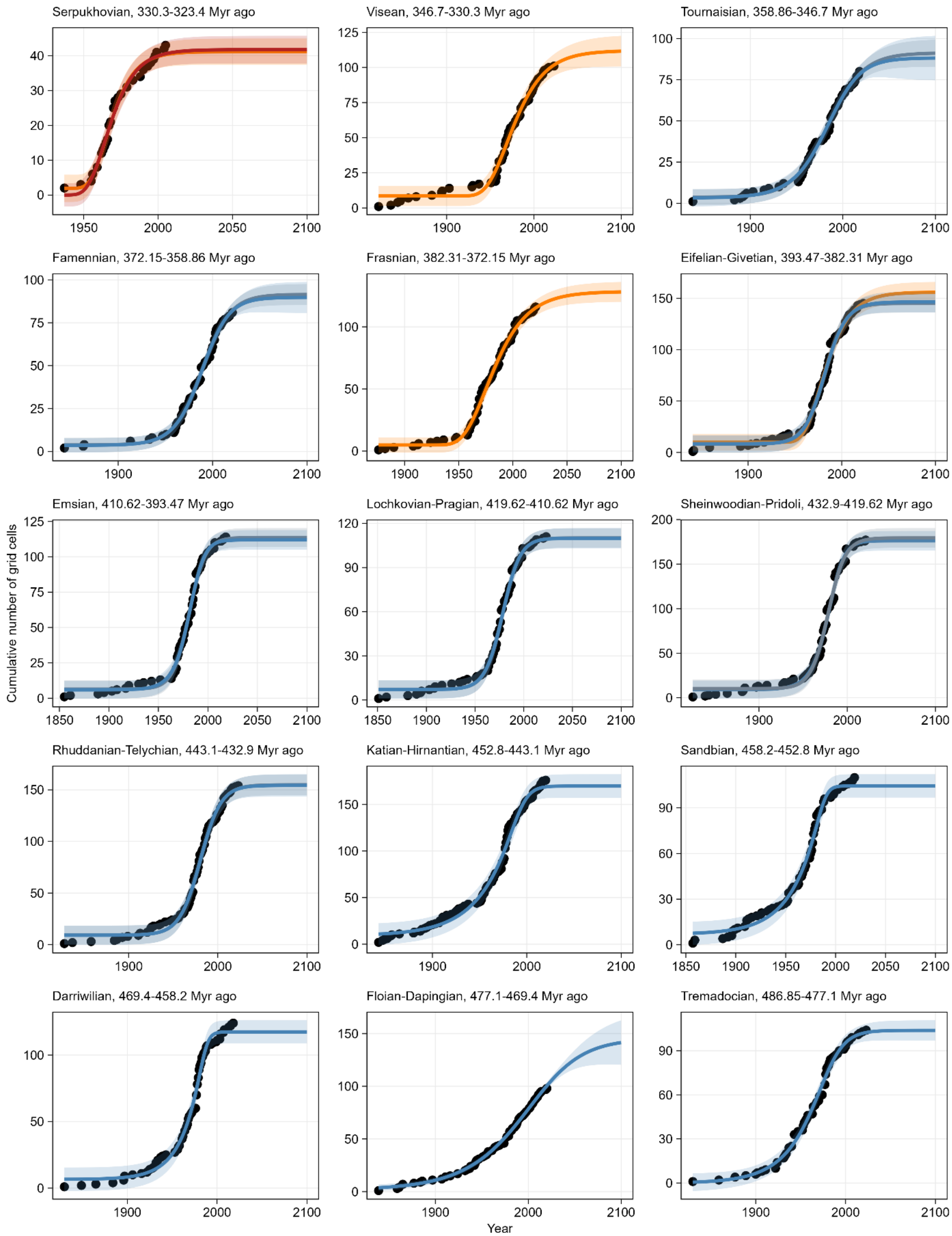

Fig. S33. Continued.

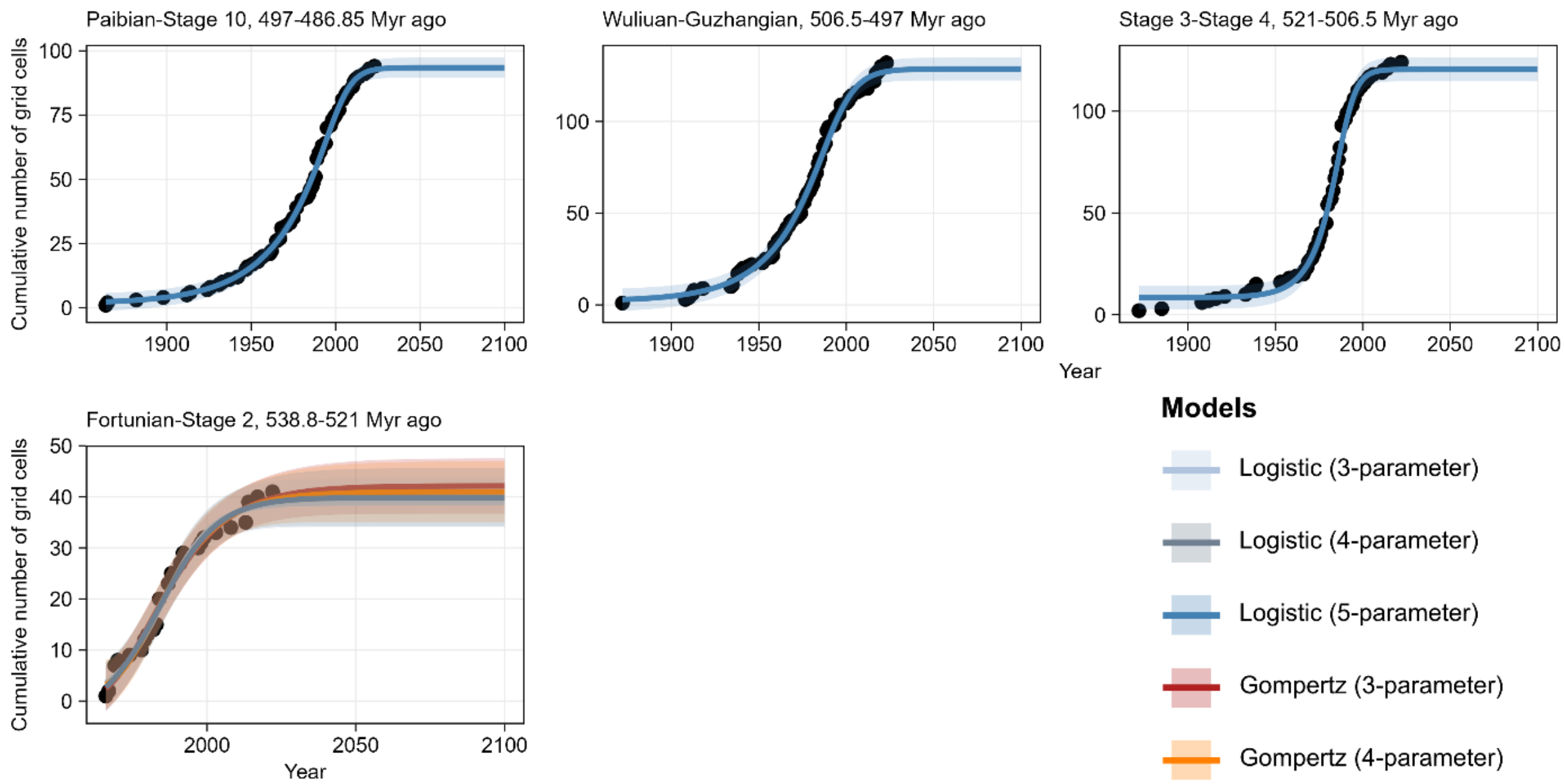

Fig. S33. Continued.

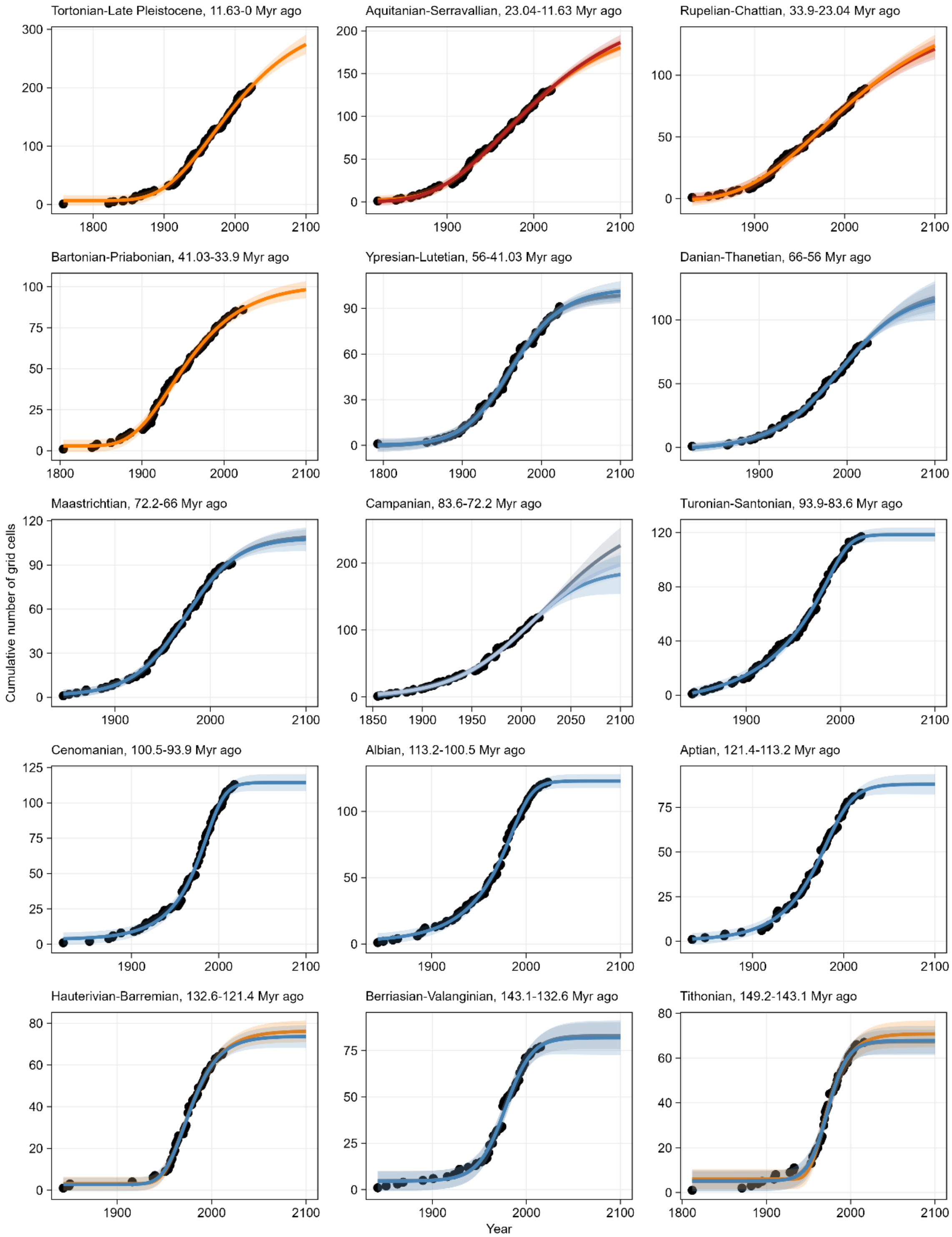

Fig. S34.

Growth models for the 49-bin approach predicting future collection effort across grid cells until the year 2100 for each time bin for the 750 km pointy-topped grid. Note that for some bins, more than one model qualified as the best (i.e., within ΔAIC = 2). Underlying data is summarized in table S11.

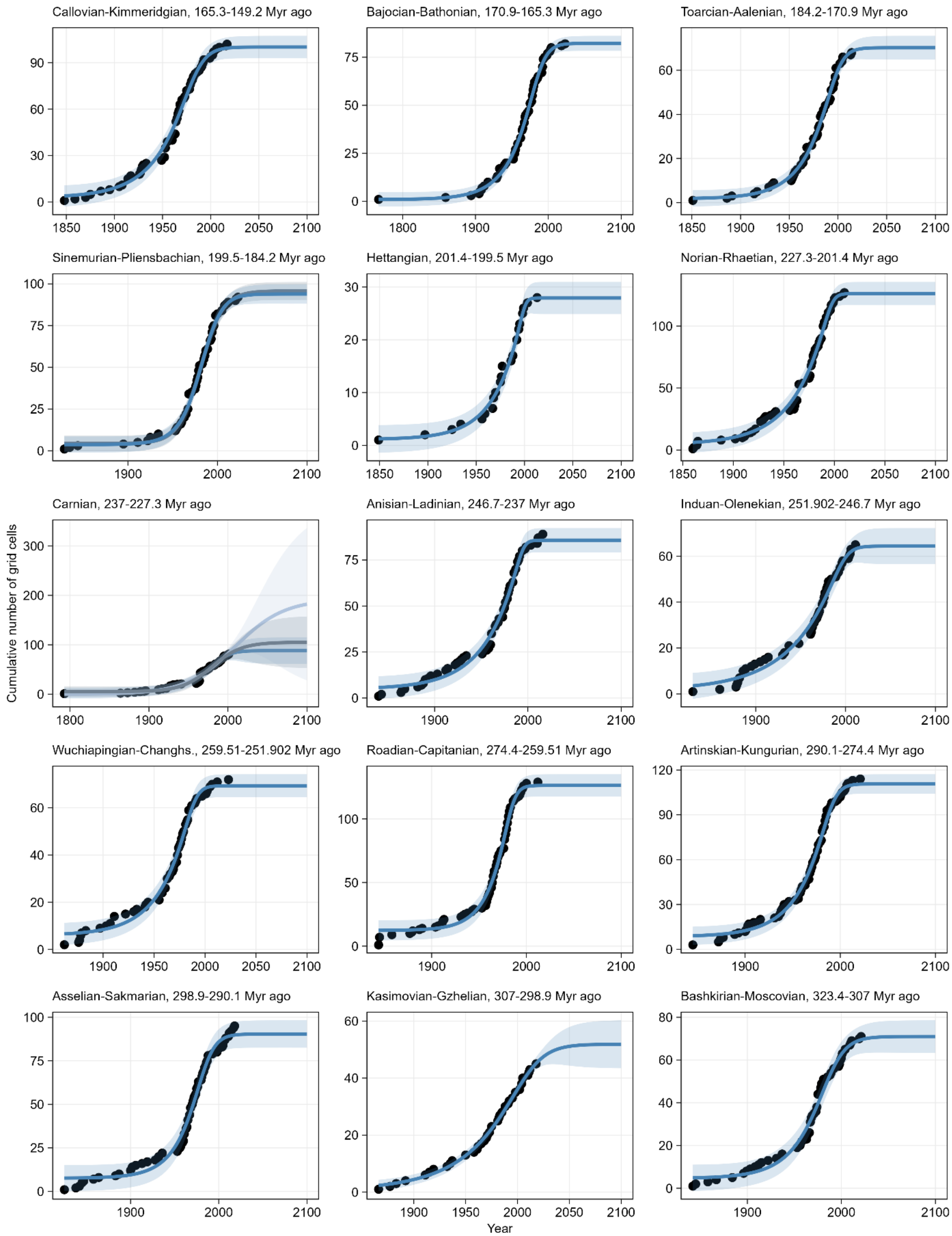

Fig. S34. Continued.

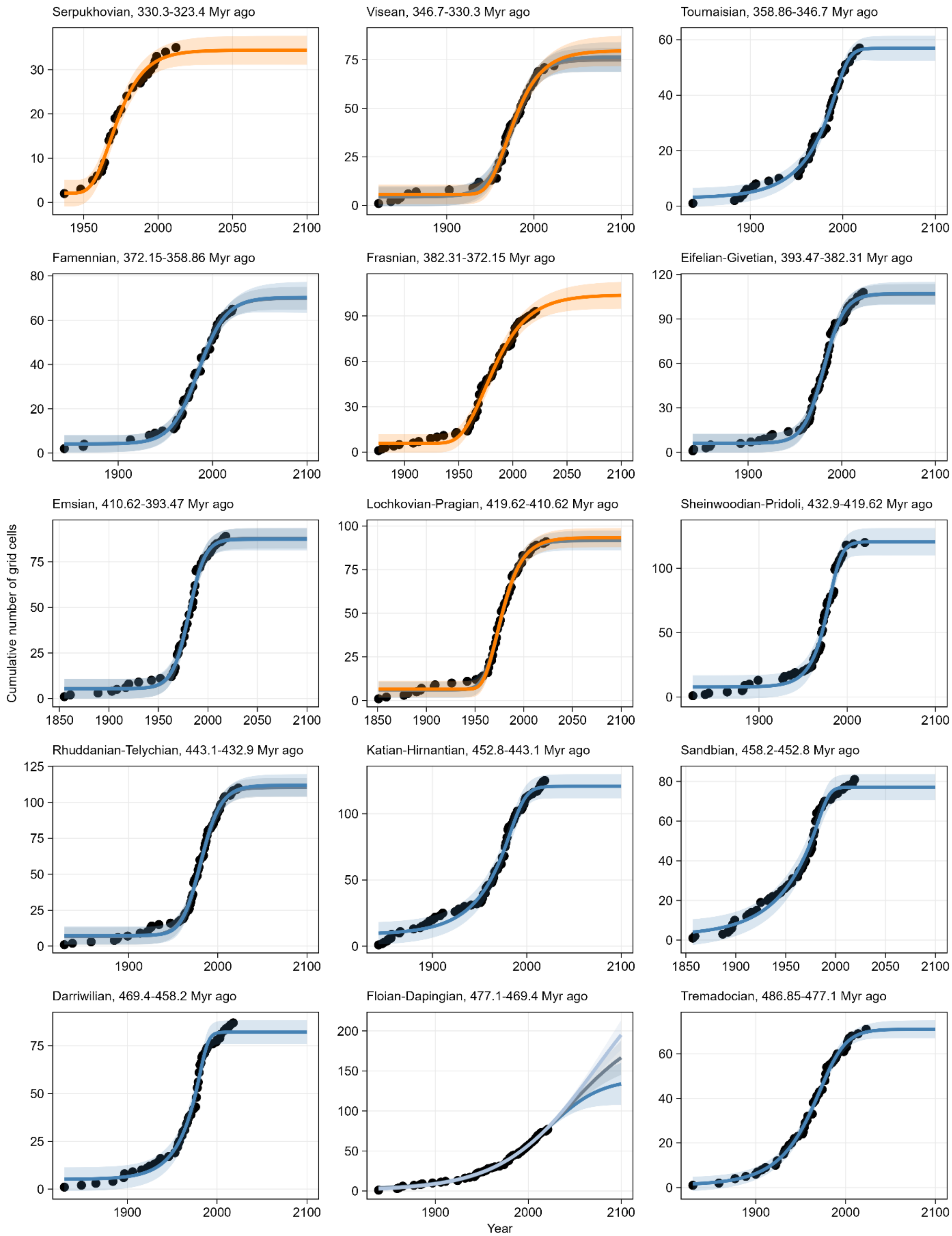

Fig. S34. Continued.

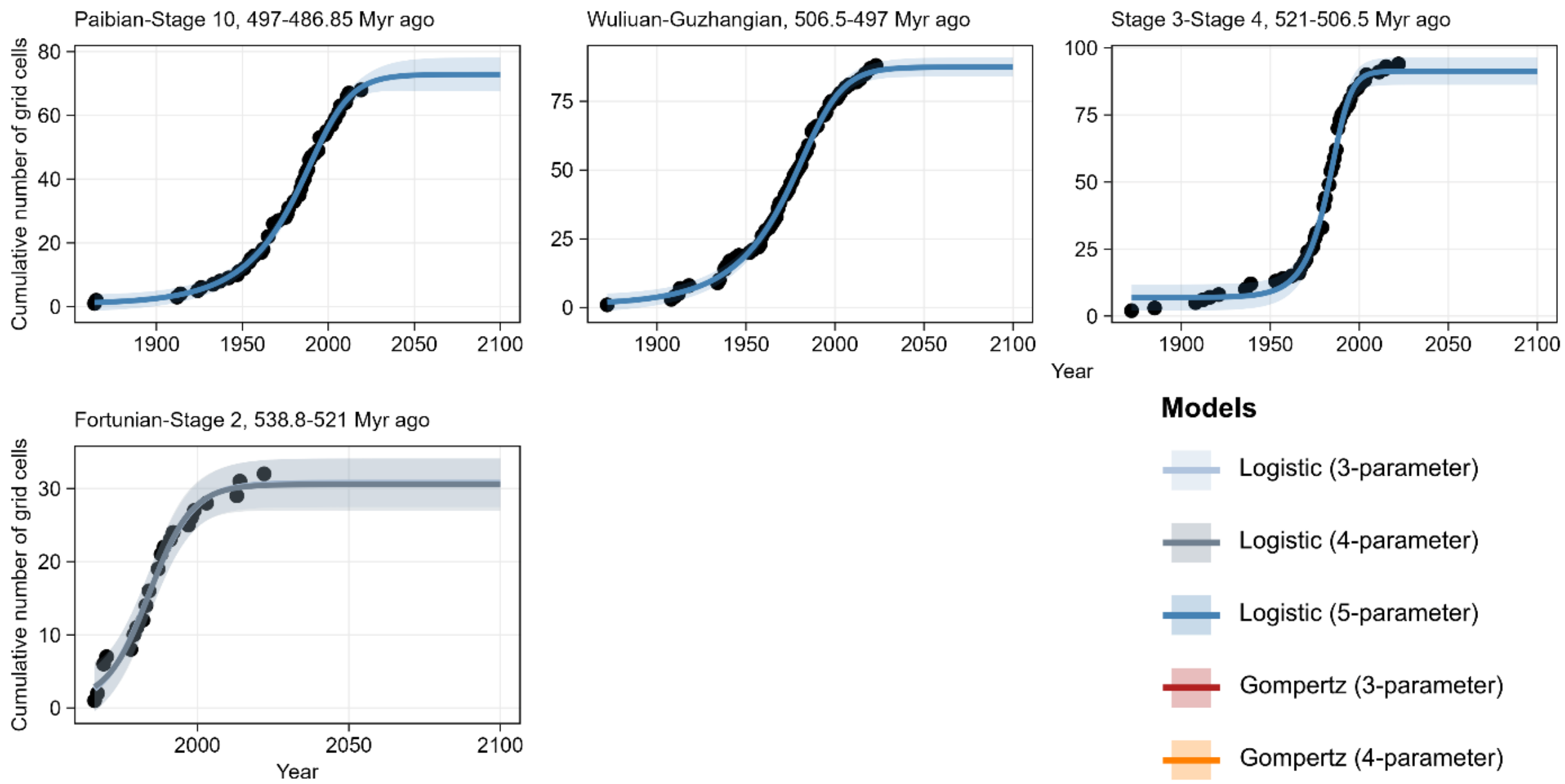

Fig. S34. Continued.

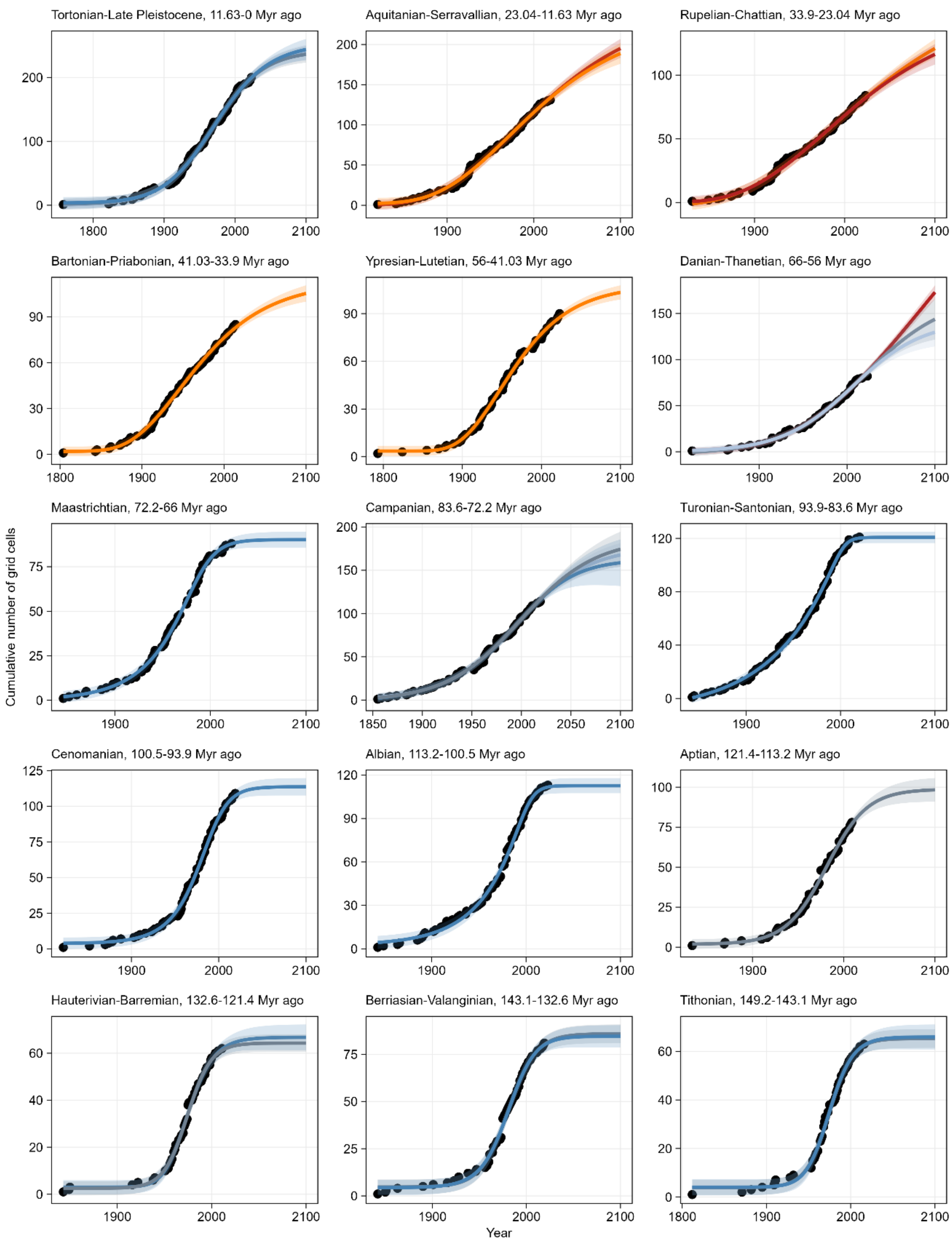

Fig. S35.

Growth models for the 49-bin approach predicting future collection effort across grid cells until the year 2100 for each time bin for the 750 km flat-topped grid. Note that for some bins, more than one model qualified as the best (i.e., within ΔAIC = 2). Underlying data is summarized in table S11.

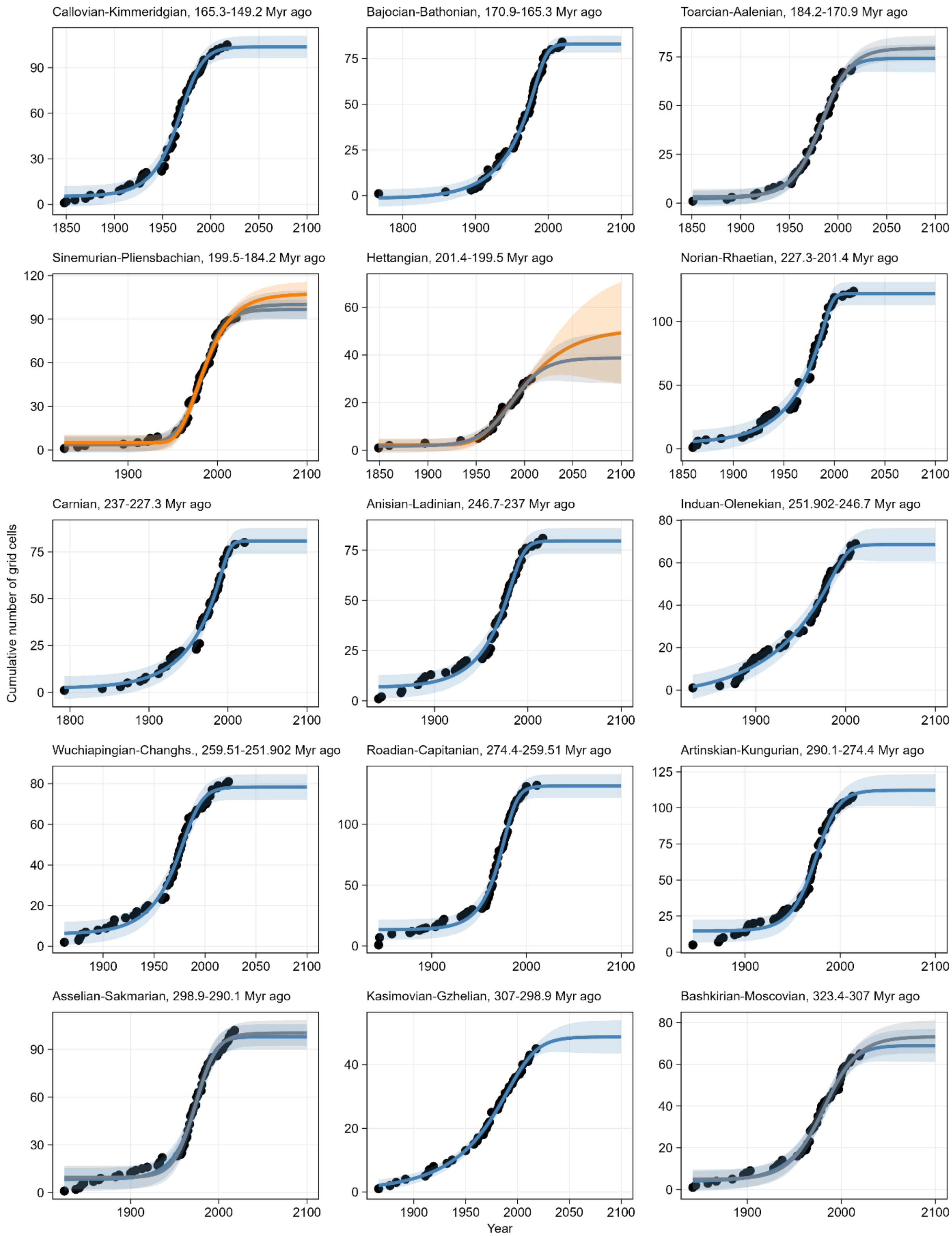

Fig. S35. Continued.

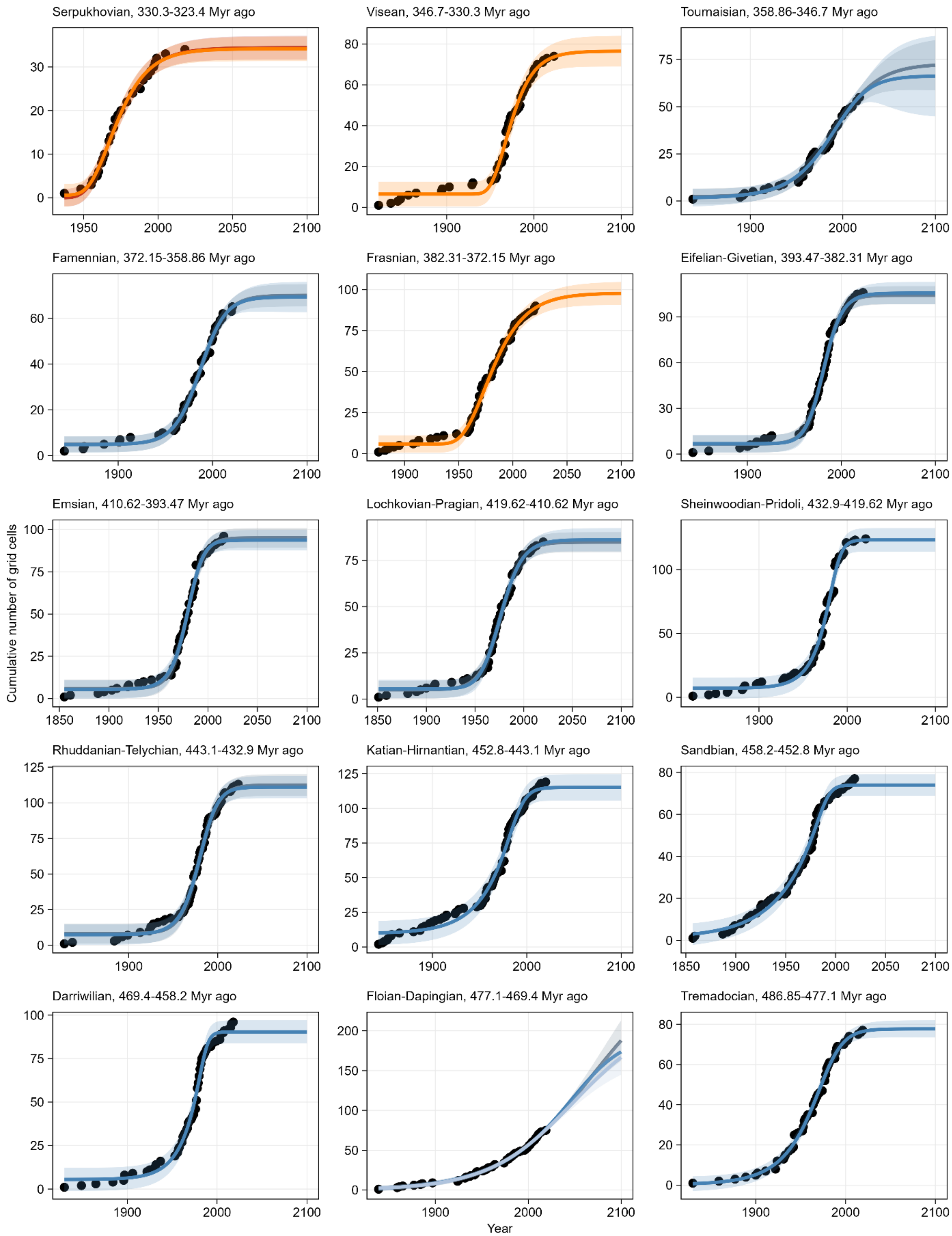

Fig. S35. Continued.

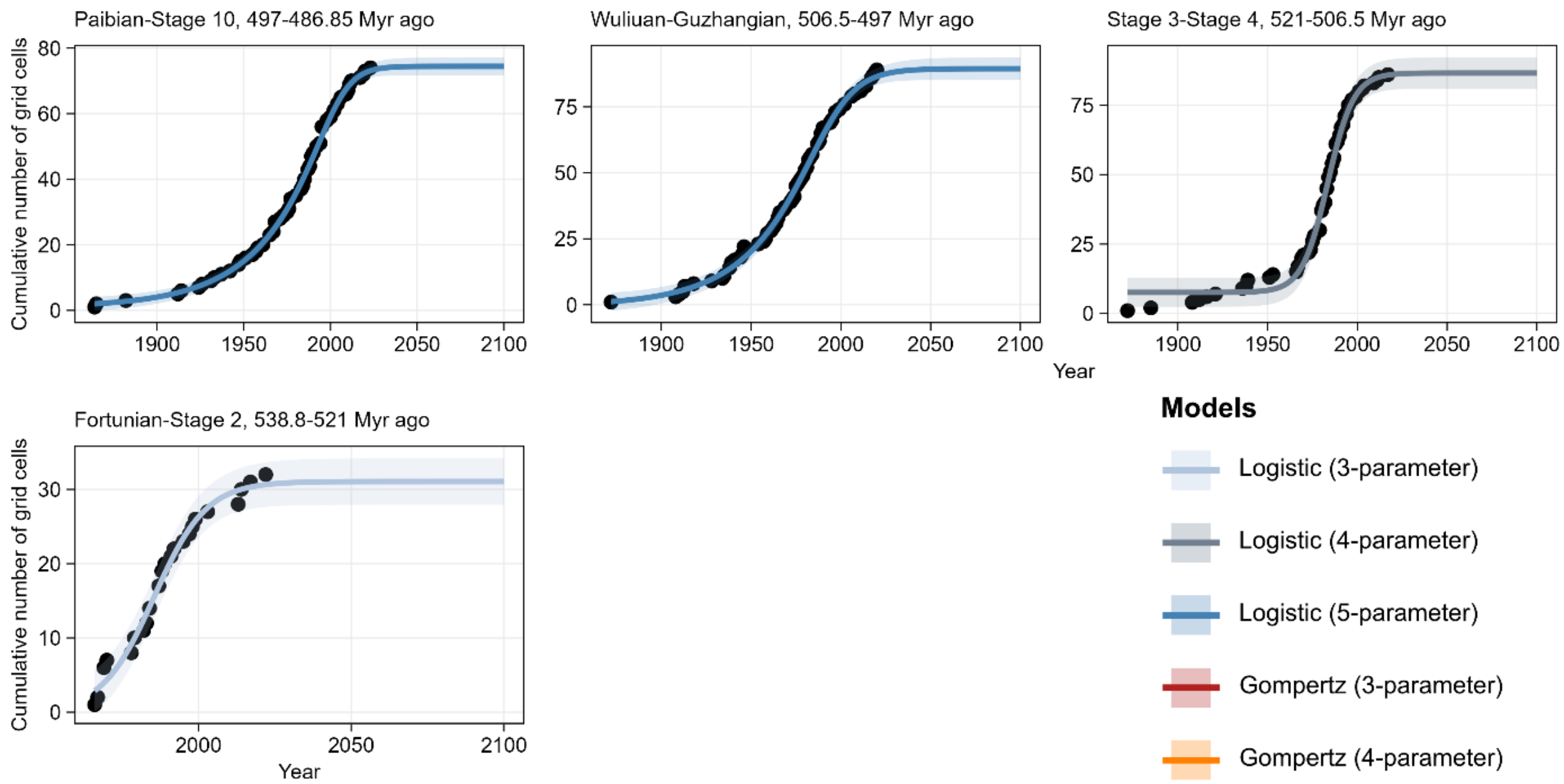

Fig. S35. Continued.

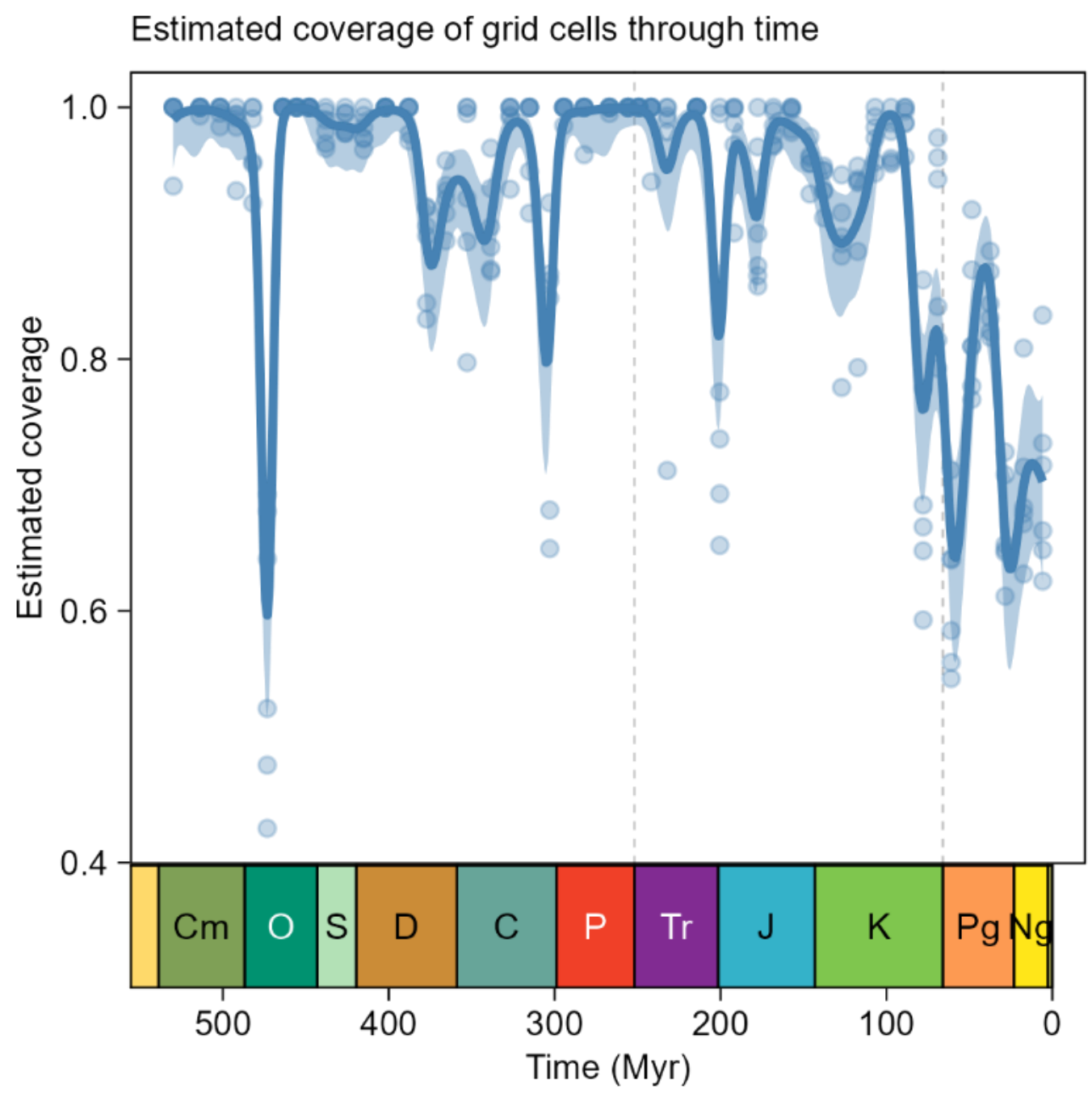

Fig. S36.

Summary graph based on predictions across all the 49 bins and six grid types. Completeness is quantified as the difference between the observed maximum number of grid cells and the predicted number based on logistic or logistic-like models. The temporal trend was visualized using a quasi-binomial generalized additive model. The graph is based on the 500 km, pointy-topped grid; see fig. S30–35 for all models across grid types; compare Fig. 2D in the main paper for results on stage-level.

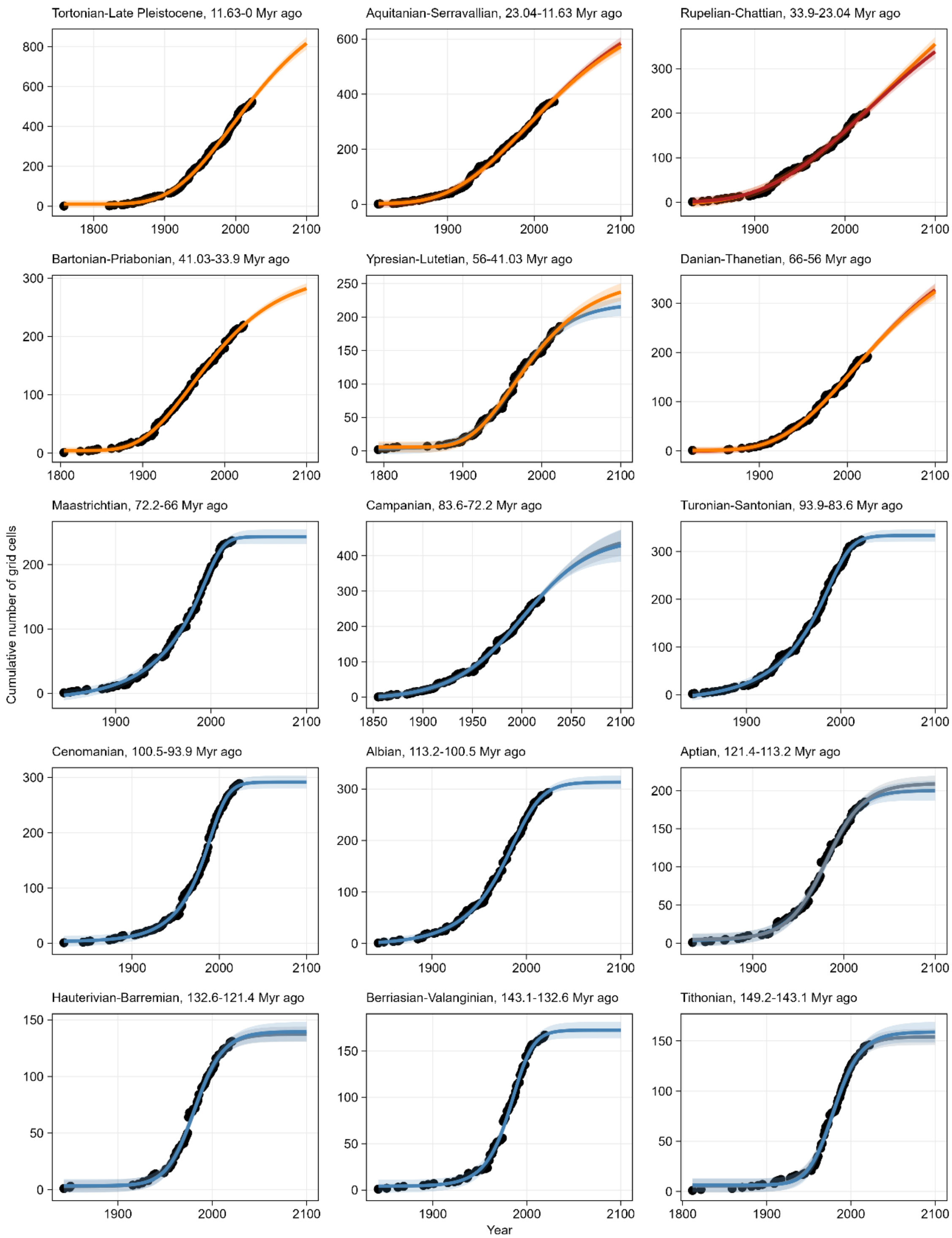

Fig. S37.

Growth models for the 49-bin approach predicting future collection effort across grid cells until the year 2100 for each time bin for the 250 km pointy-topped grid (all marine data, not shallow-water only). Note that for some bins, more than one model qualified as the best (i.e., within ΔAIC = 2). Underlying data is summarized in table S12.

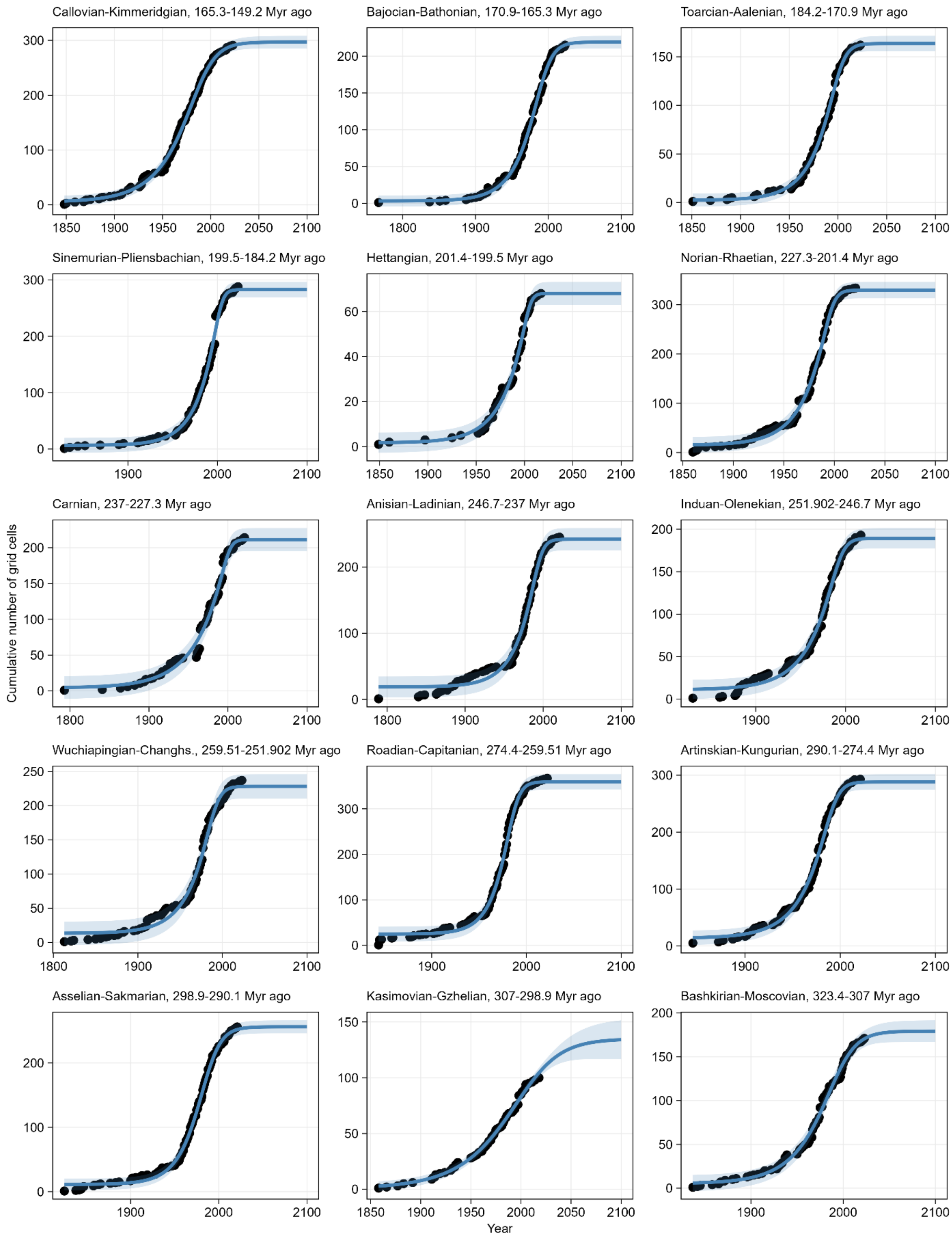

Fig. S37. Continued.

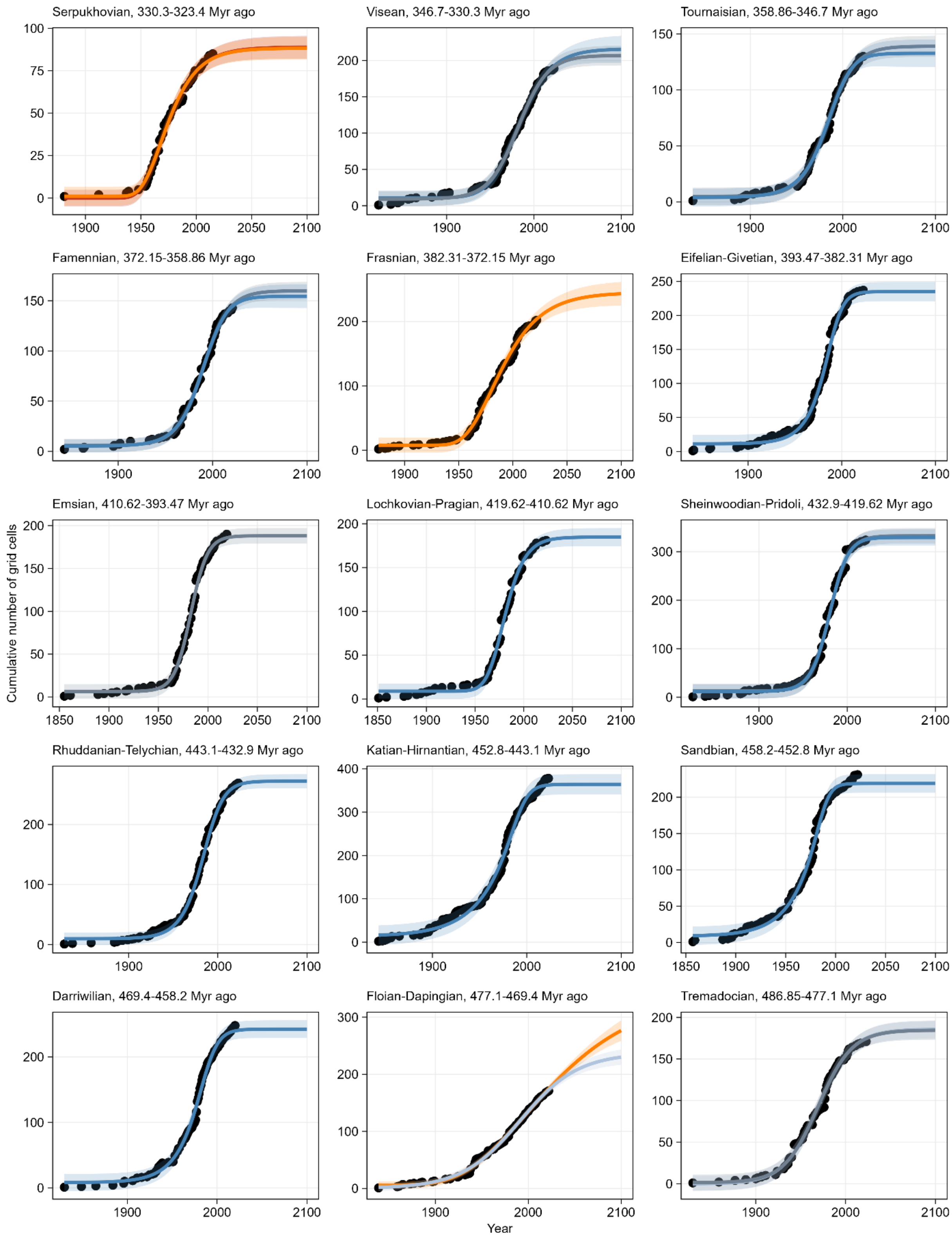

Fig. S37. Continued.

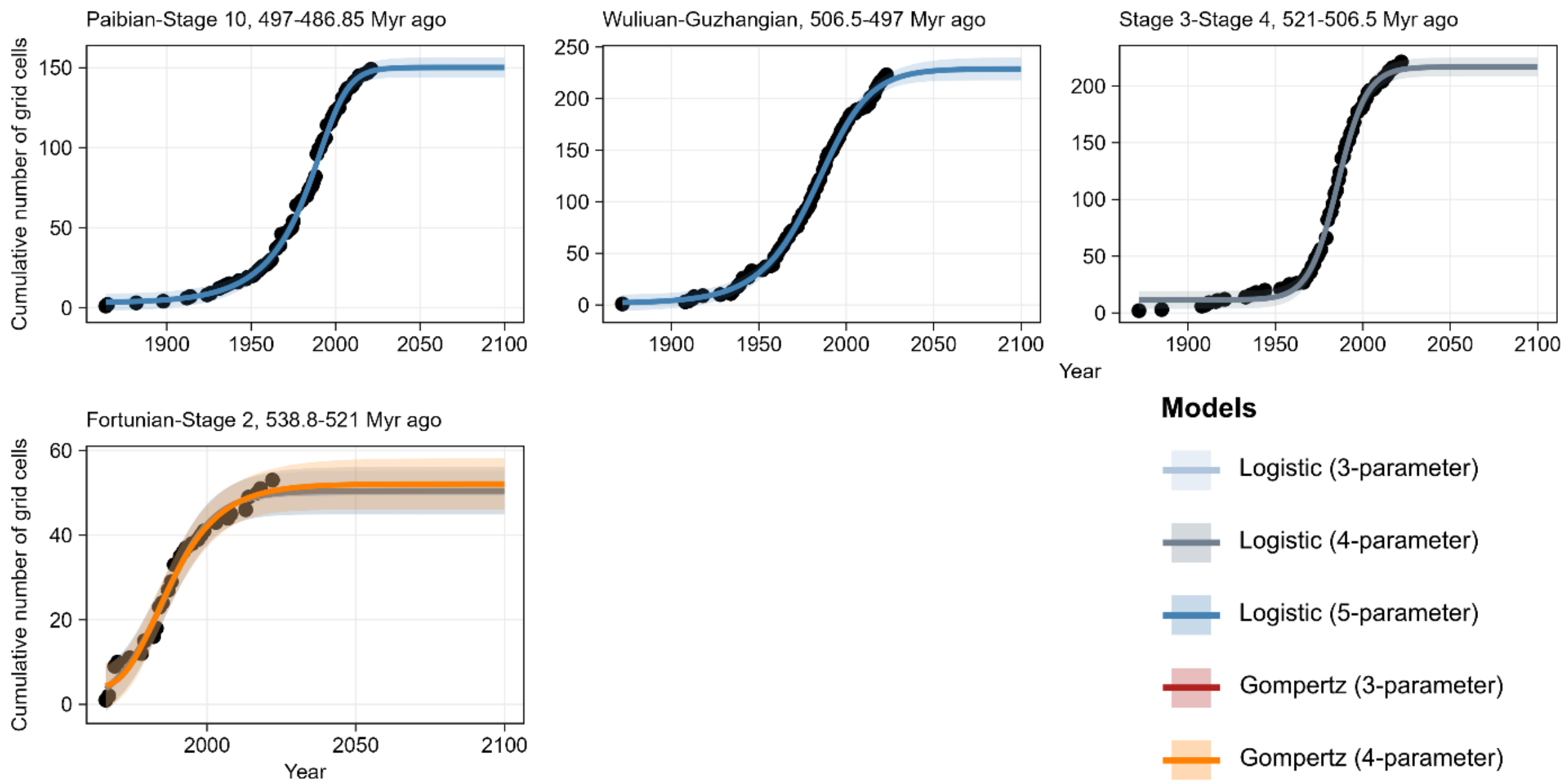

Fig. S37. Continued.

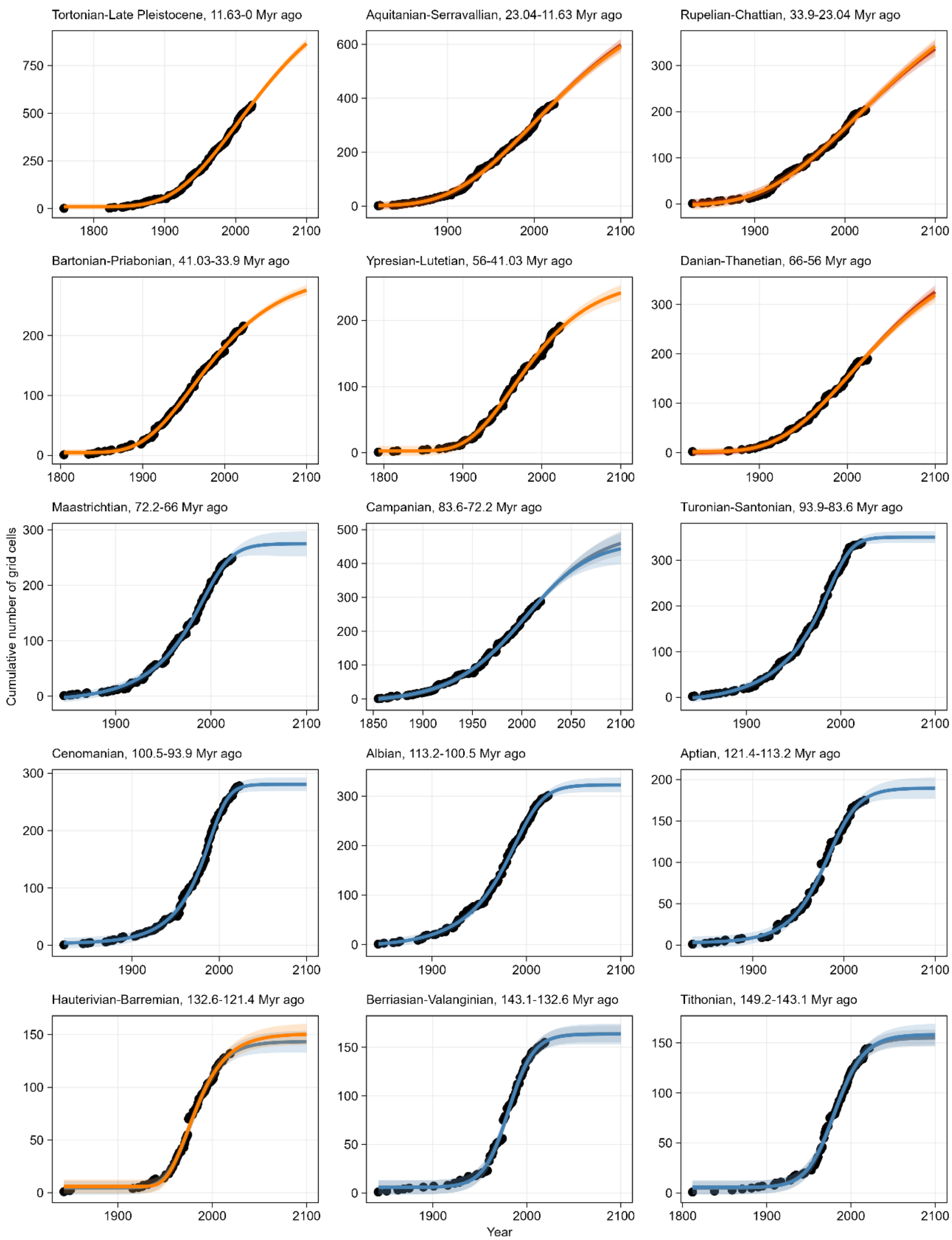

Fig. S38.

Growth models for the 49-bin approach predicting future collection effort across grid cells until the year 2100 for each time bin for the 250 km flat-topped grid (all marine data, not shallow-water only). Note that for some bins, more than one model qualified as the best (i.e., within ΔAIC = 2). Underlying data is summarized in table S12.

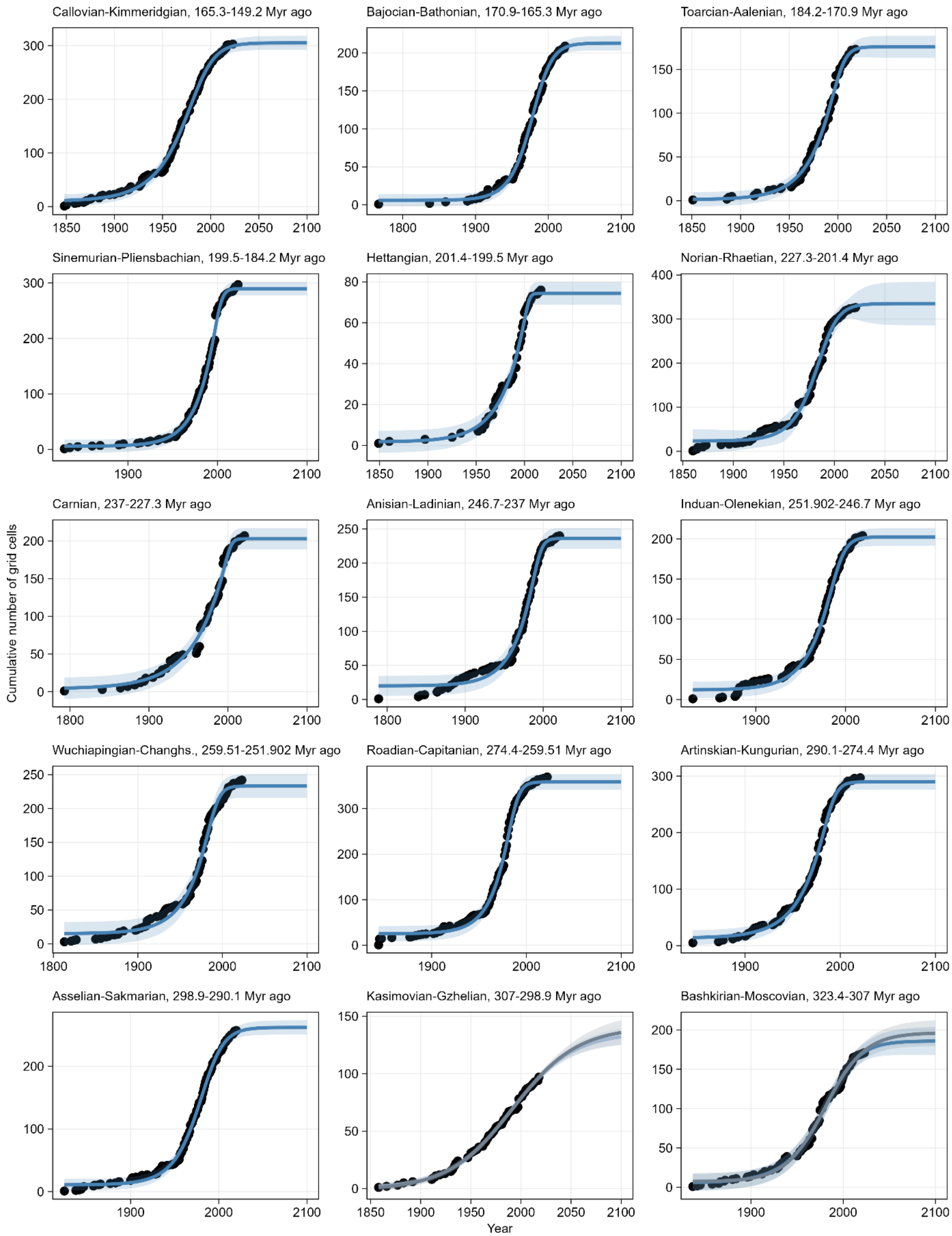

Fig. S38. Continued.

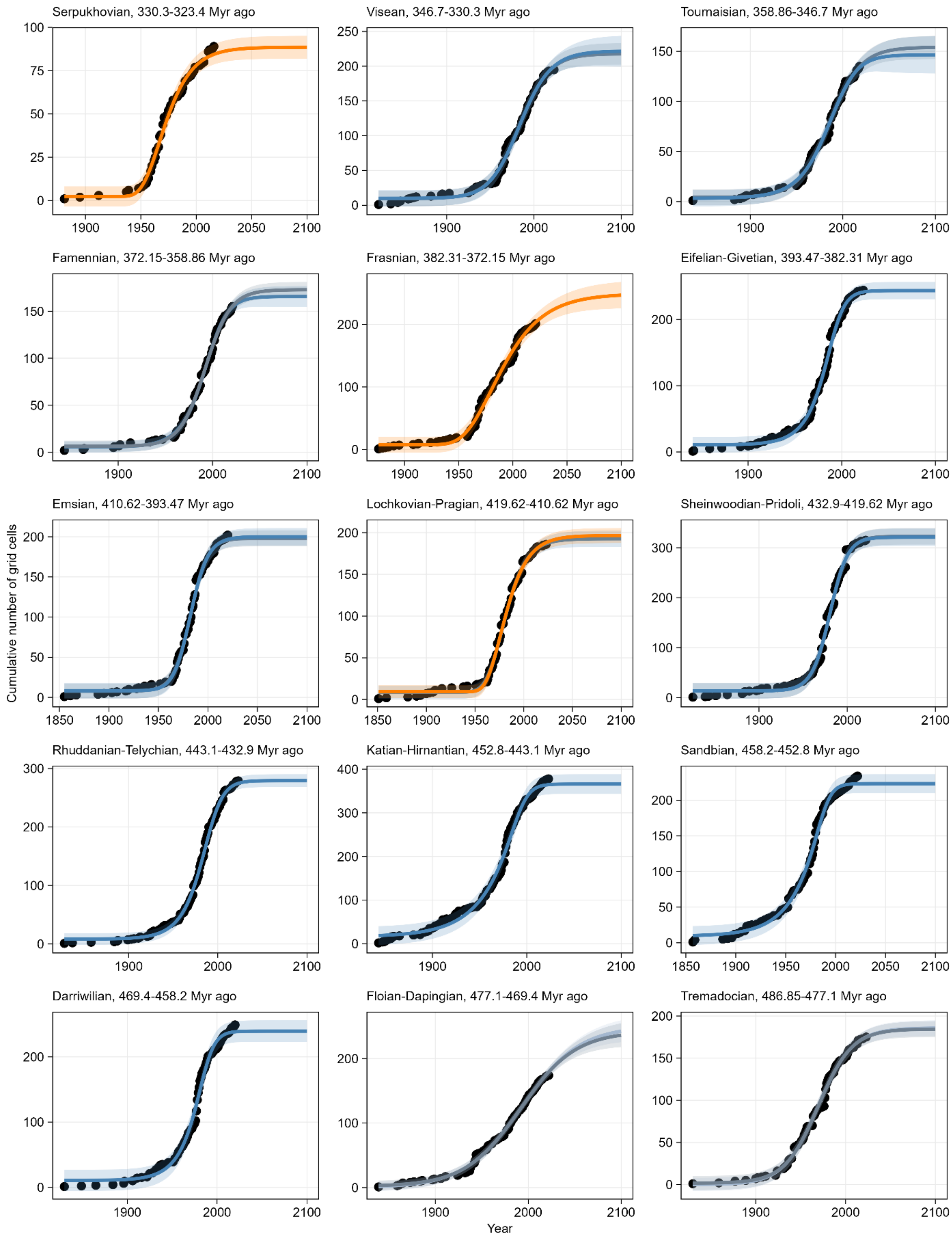

Fig. S38. Continued.

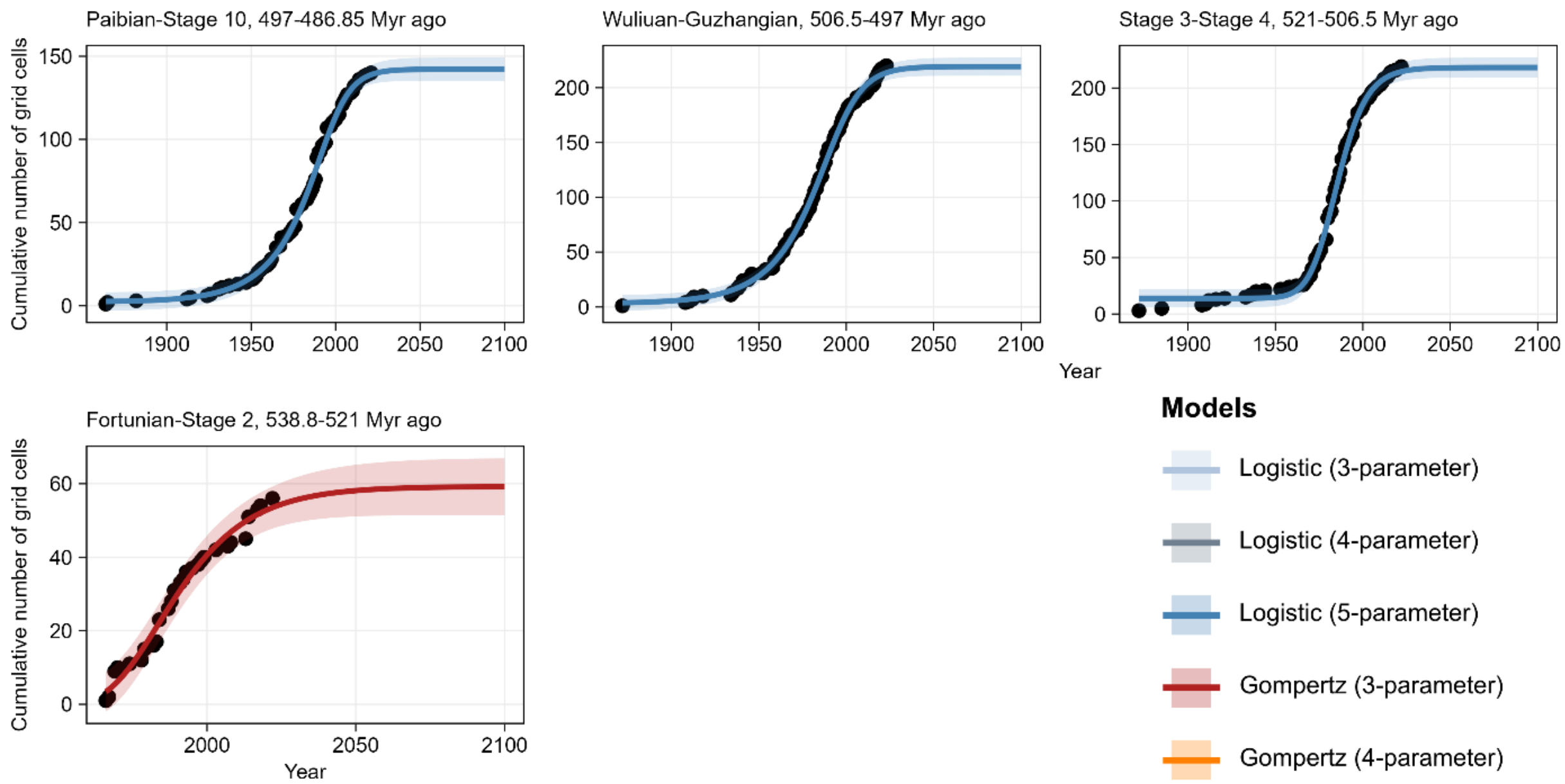

Fig. S38. Continued.

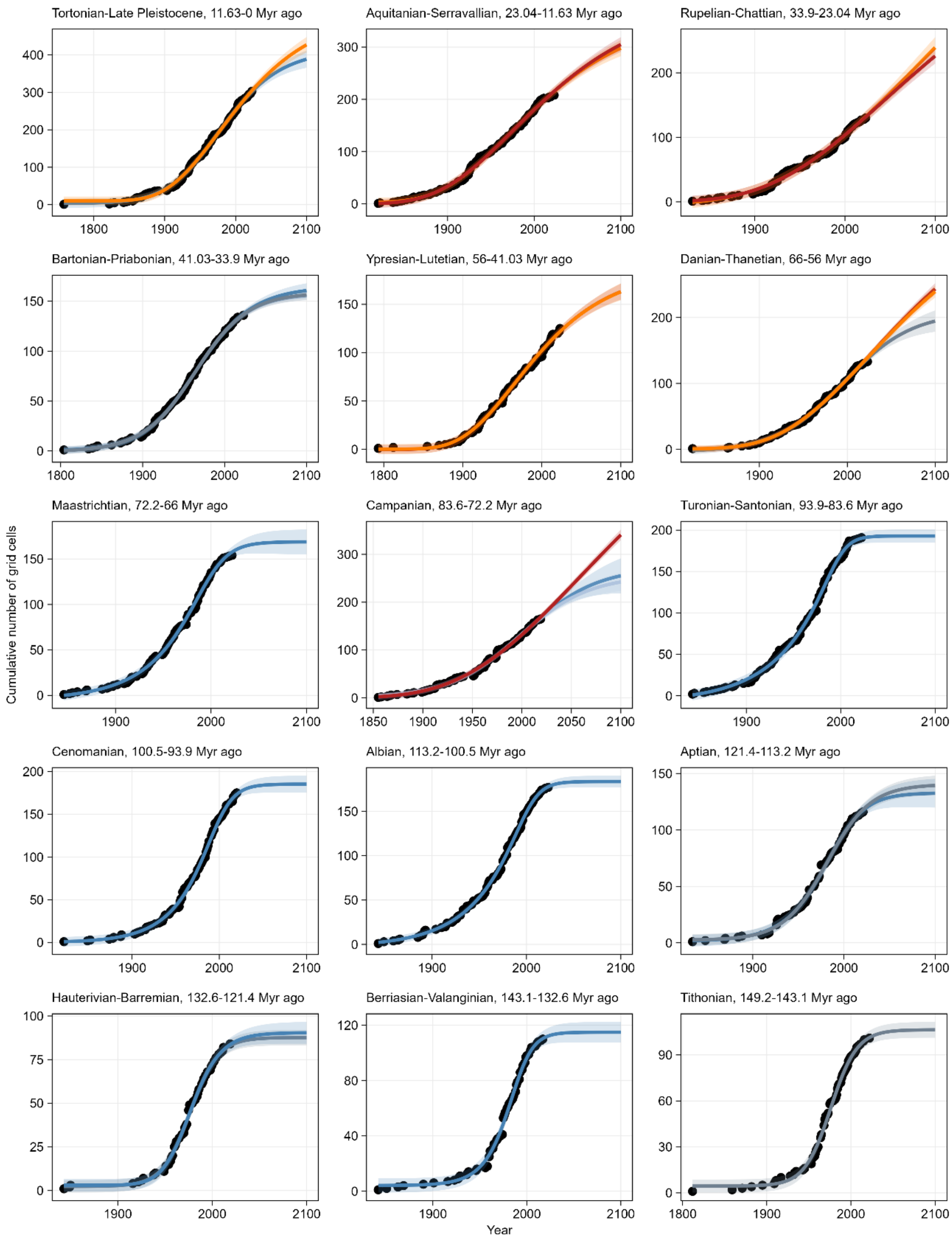

Fig. S39.

Growth models for the 49-bin approach predicting future collection effort across grid cells until the year 2100 for each time bin for the 500 km pointy-topped grid (all marine data, not shallow-water only). Note that for some bins, more than one model qualified as the best (i.e., within ΔAIC = 2). Underlying data is summarized in table S12.

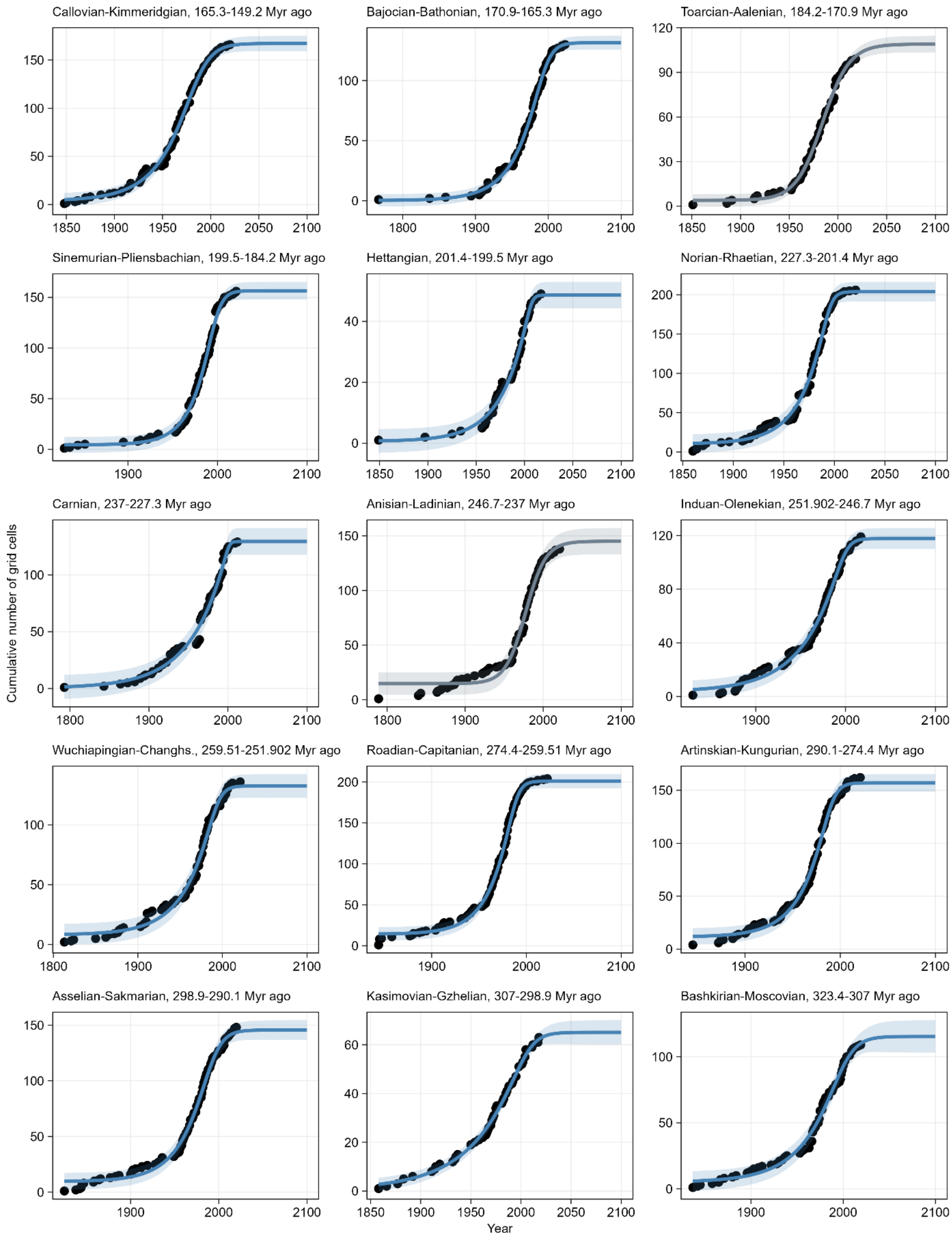

Fig. S39. Continued.

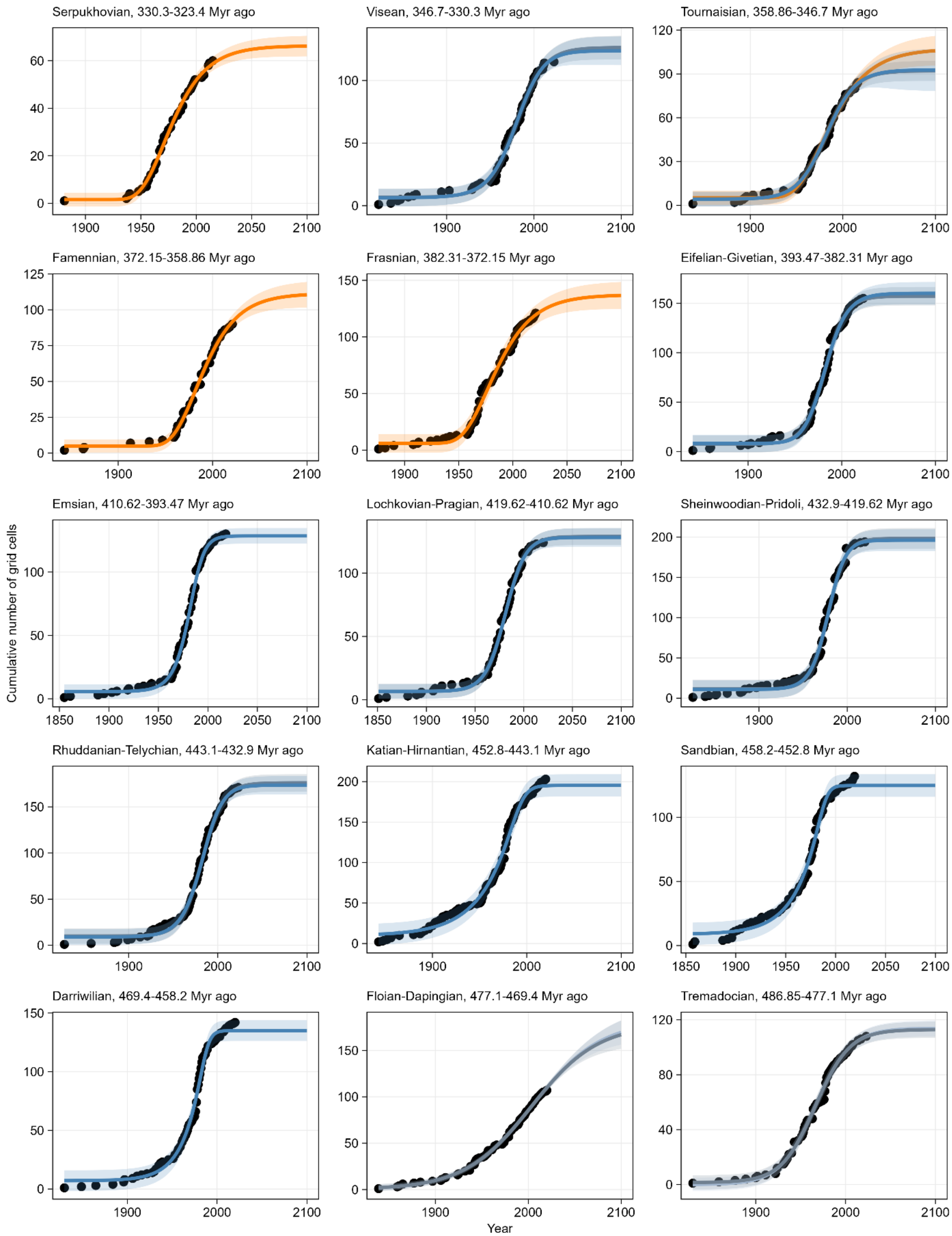

Fig. S39. Continued.

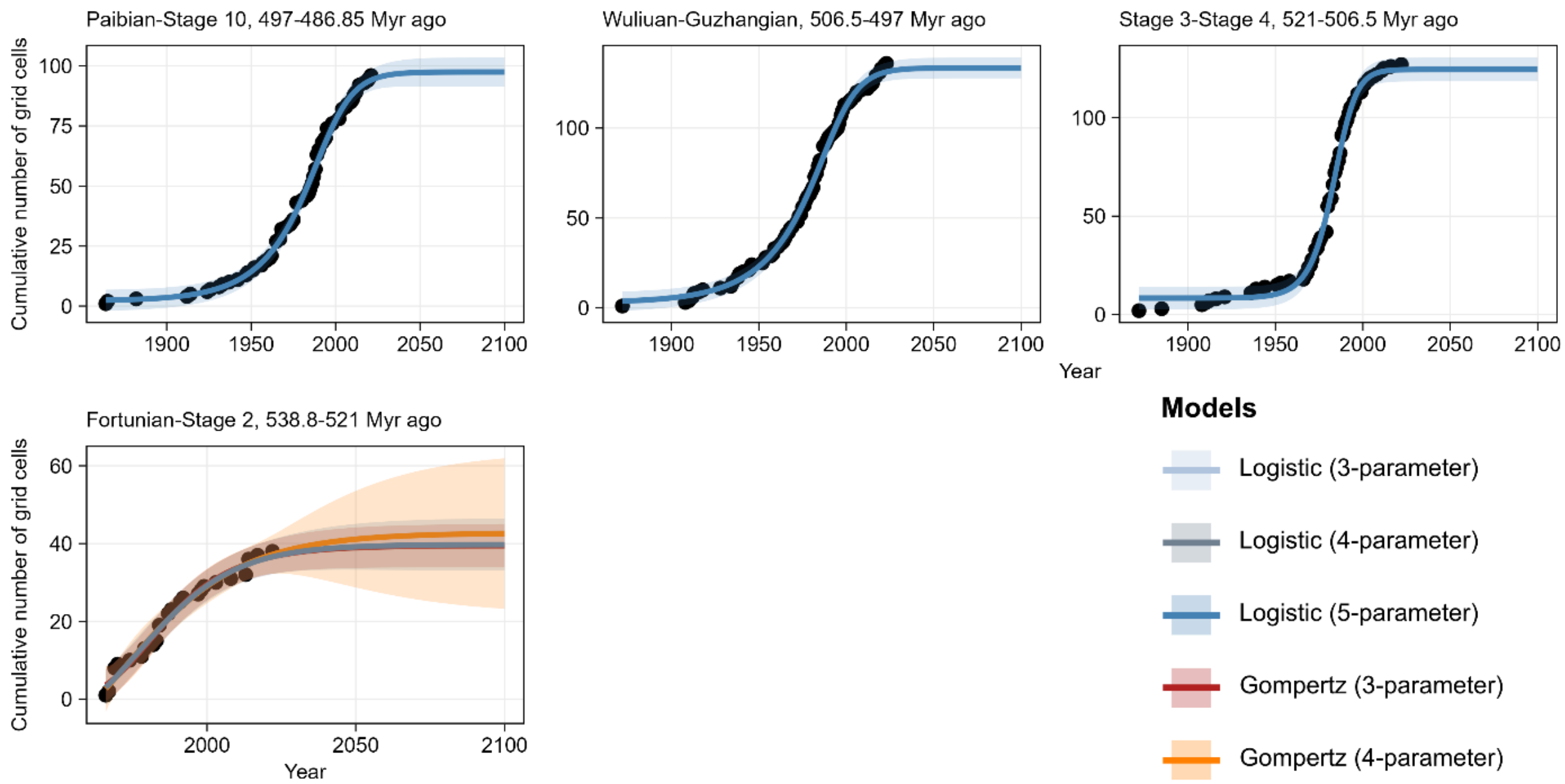

Fig. S39. Continued.

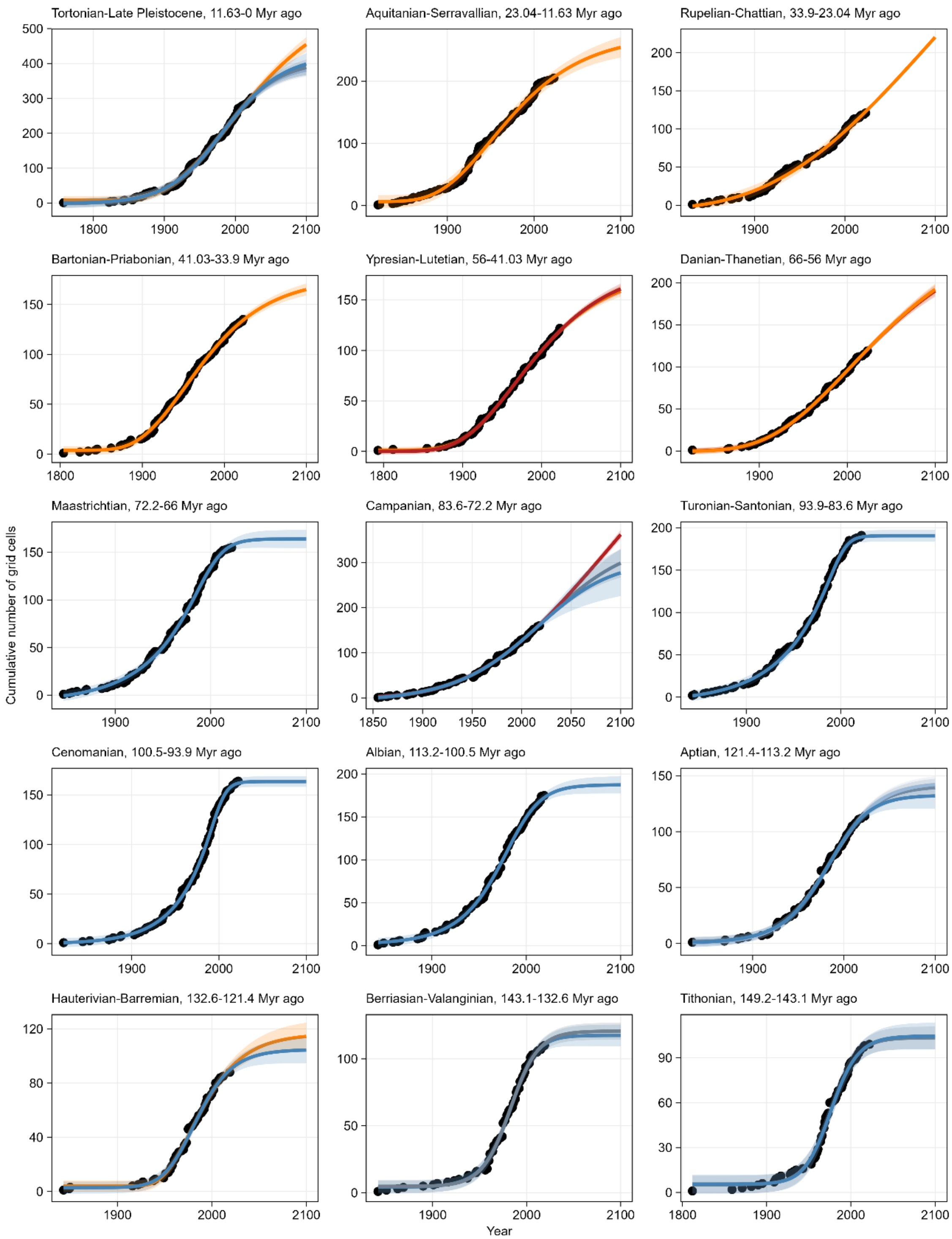

Fig. S40.

Growth models for the 49-bin approach predicting future collection effort across grid cells until the year 2100 for each time bin for the 500 km flat-topped grid (all marine data, not shallow-water only). Note that for some bins, more than one model qualified as the best (i.e., within ΔAIC = 2). Underlying data is summarized in table S12.

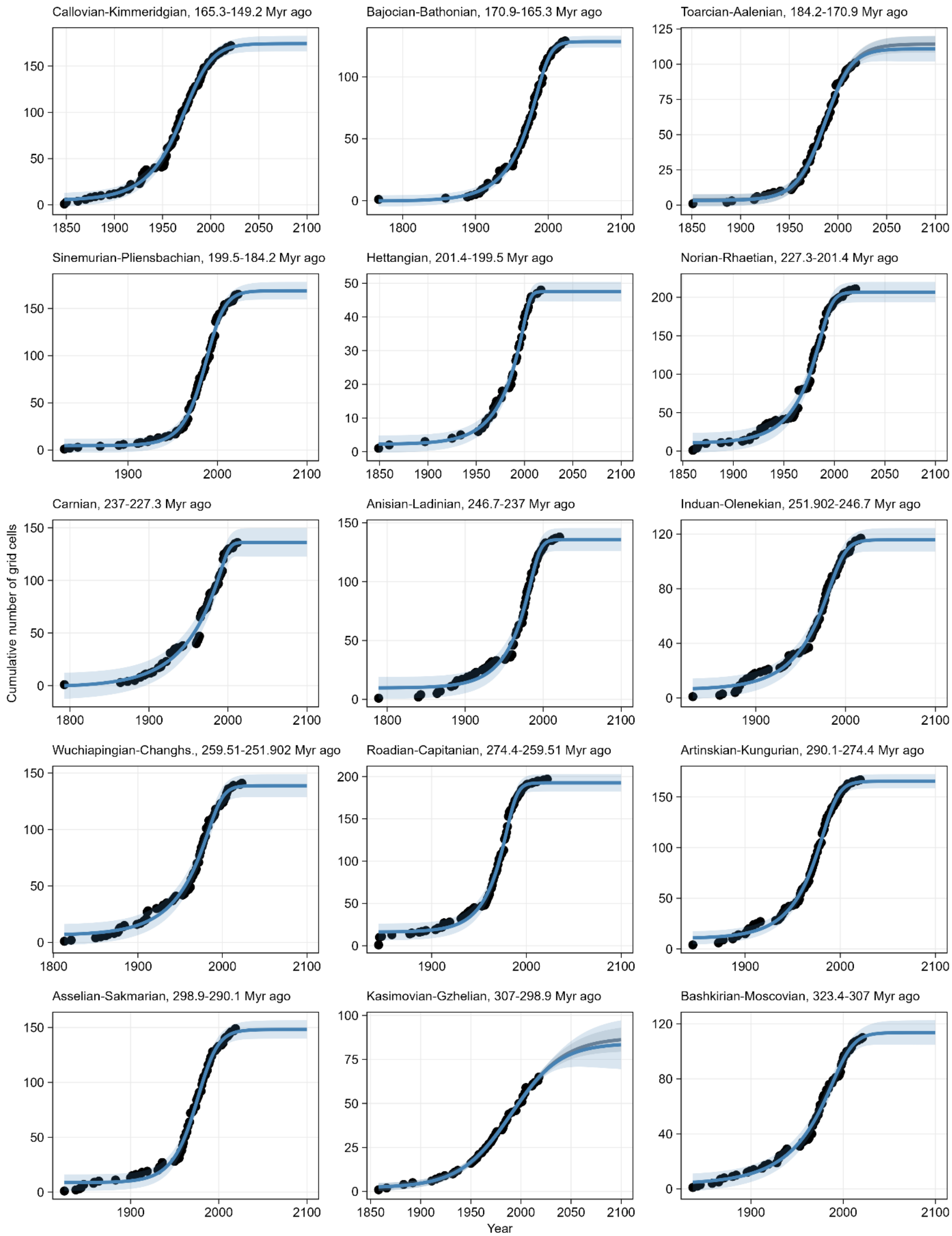

Fig. S40. Continued.

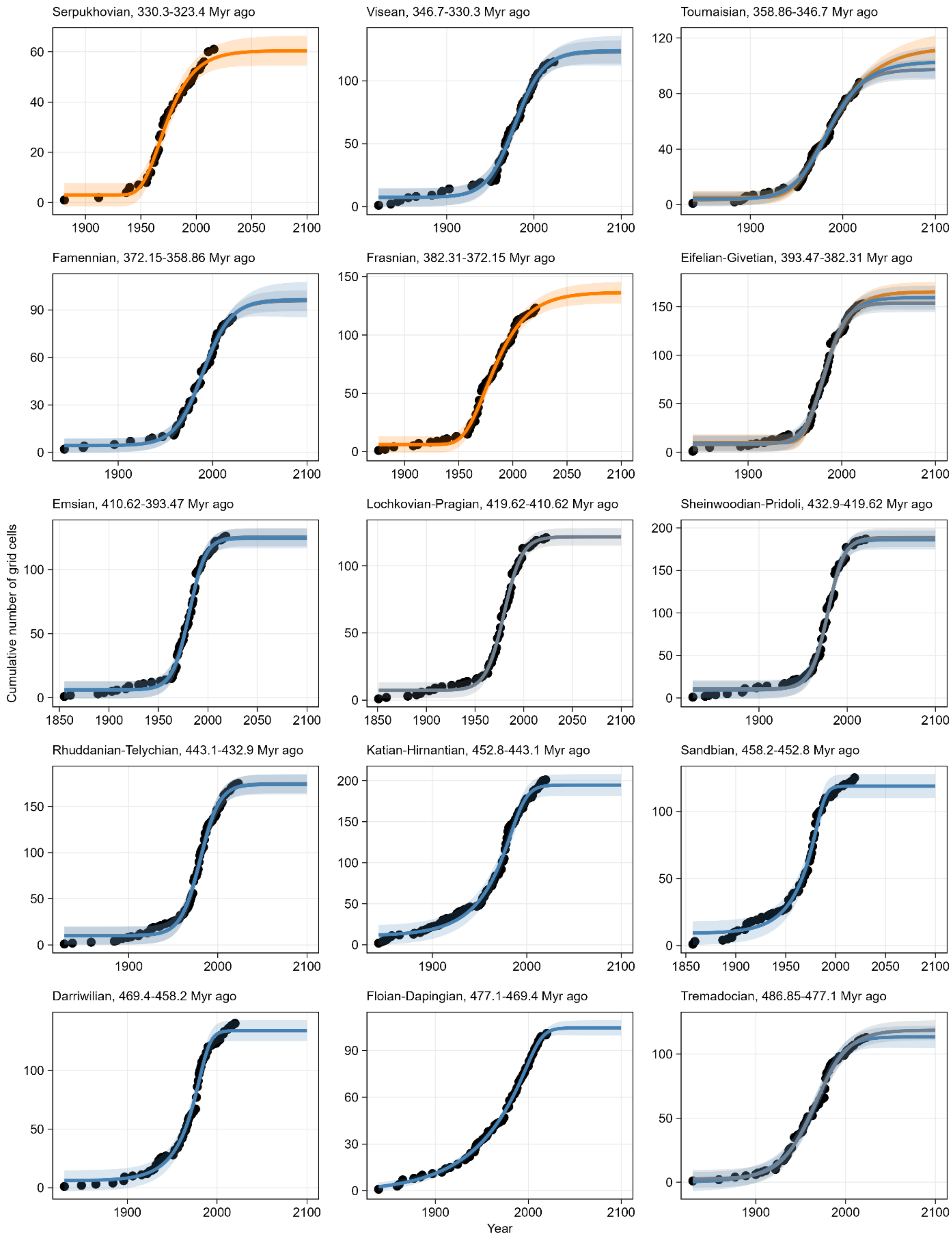

Fig. S40. Continued.

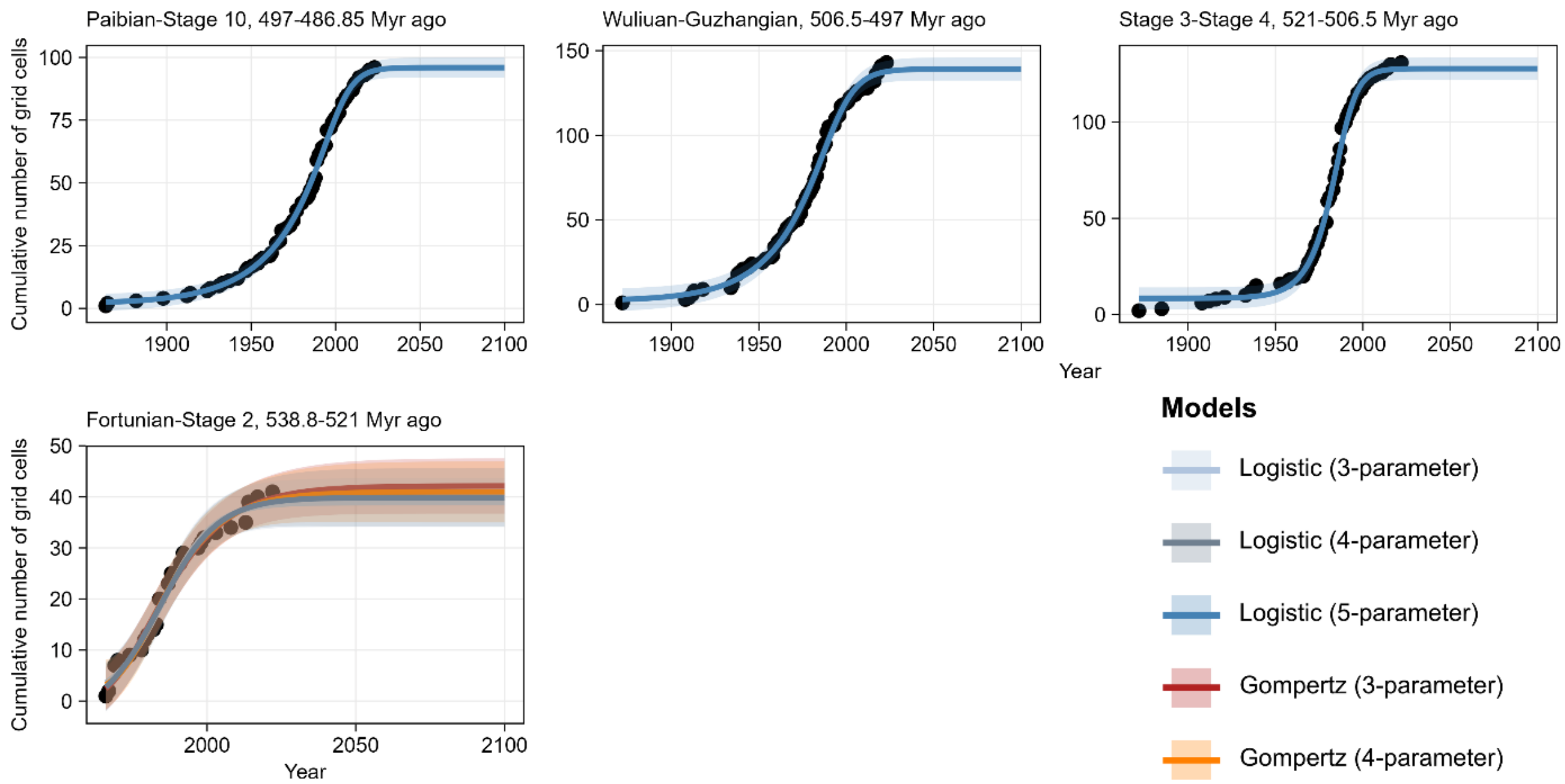

Fig. S40. Continued.

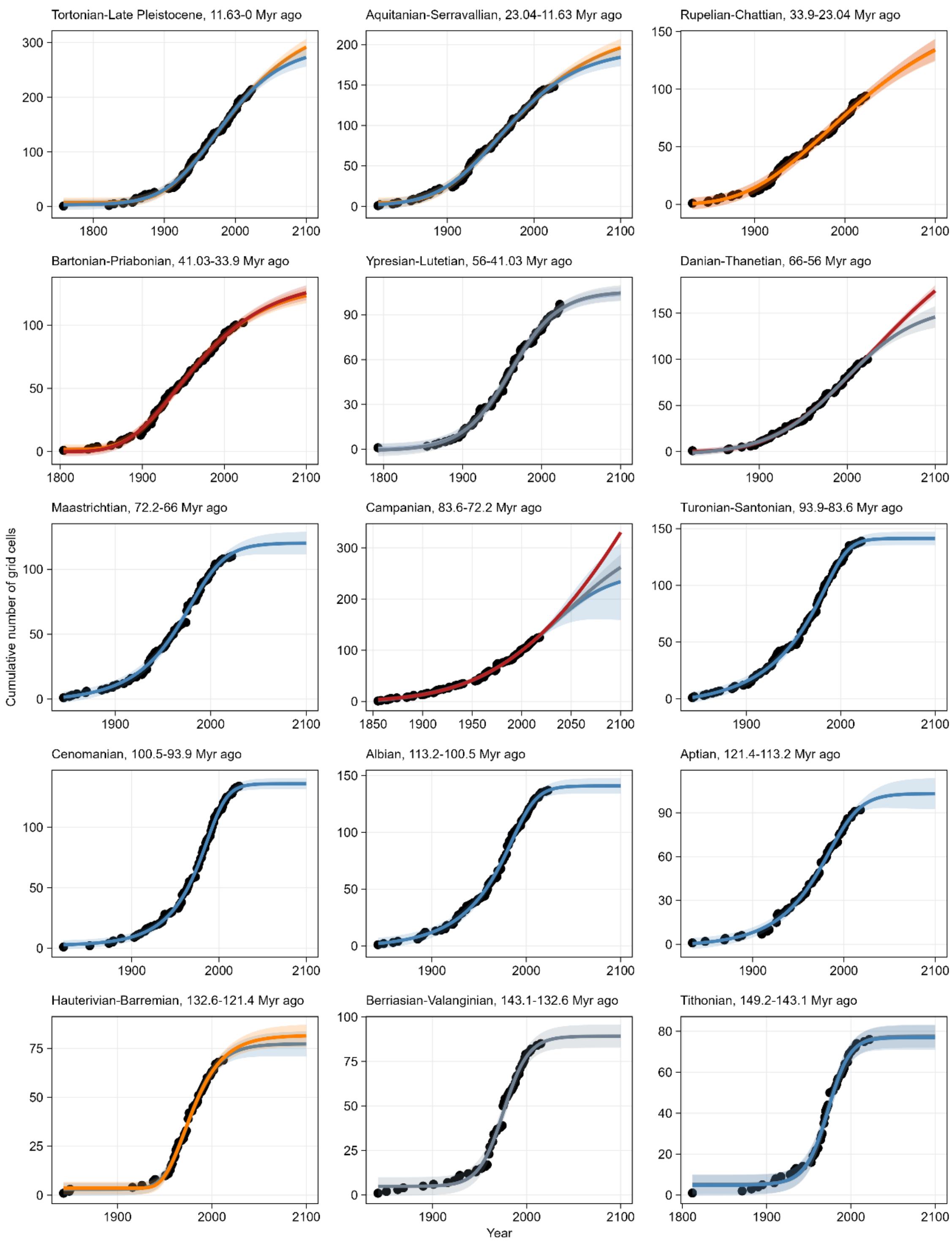

Fig. S41.

Growth models for the 49-bin approach predicting future collection effort across grid cells until the year 2100 for each time bin for the 750 km pointy-topped grid (all marine data, not shallow-water only). Note that for some bins, more than one model qualified as the best (i.e., within ΔAIC = 2). Underlying data is summarized in table S12.

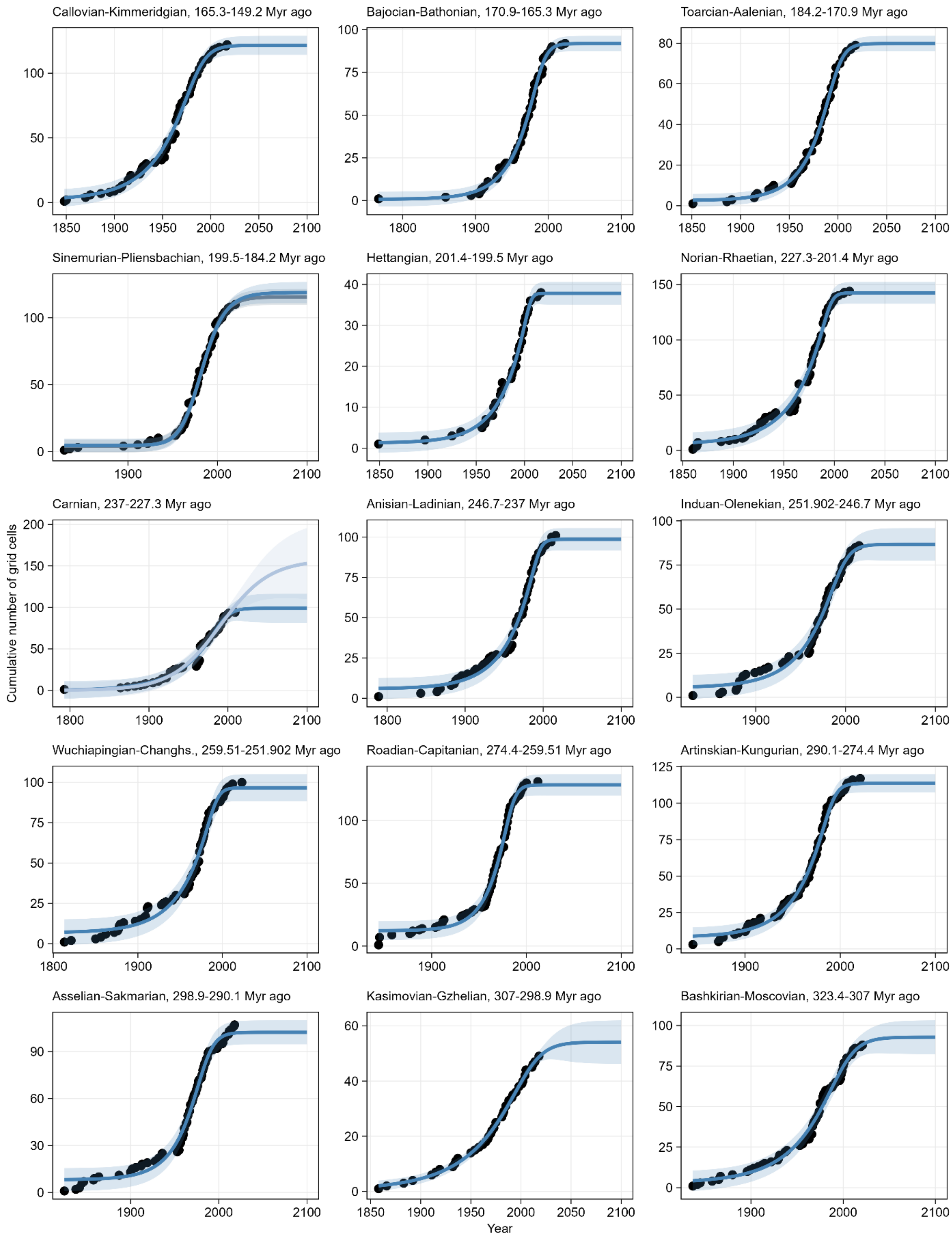

Fig. S41. Continued.

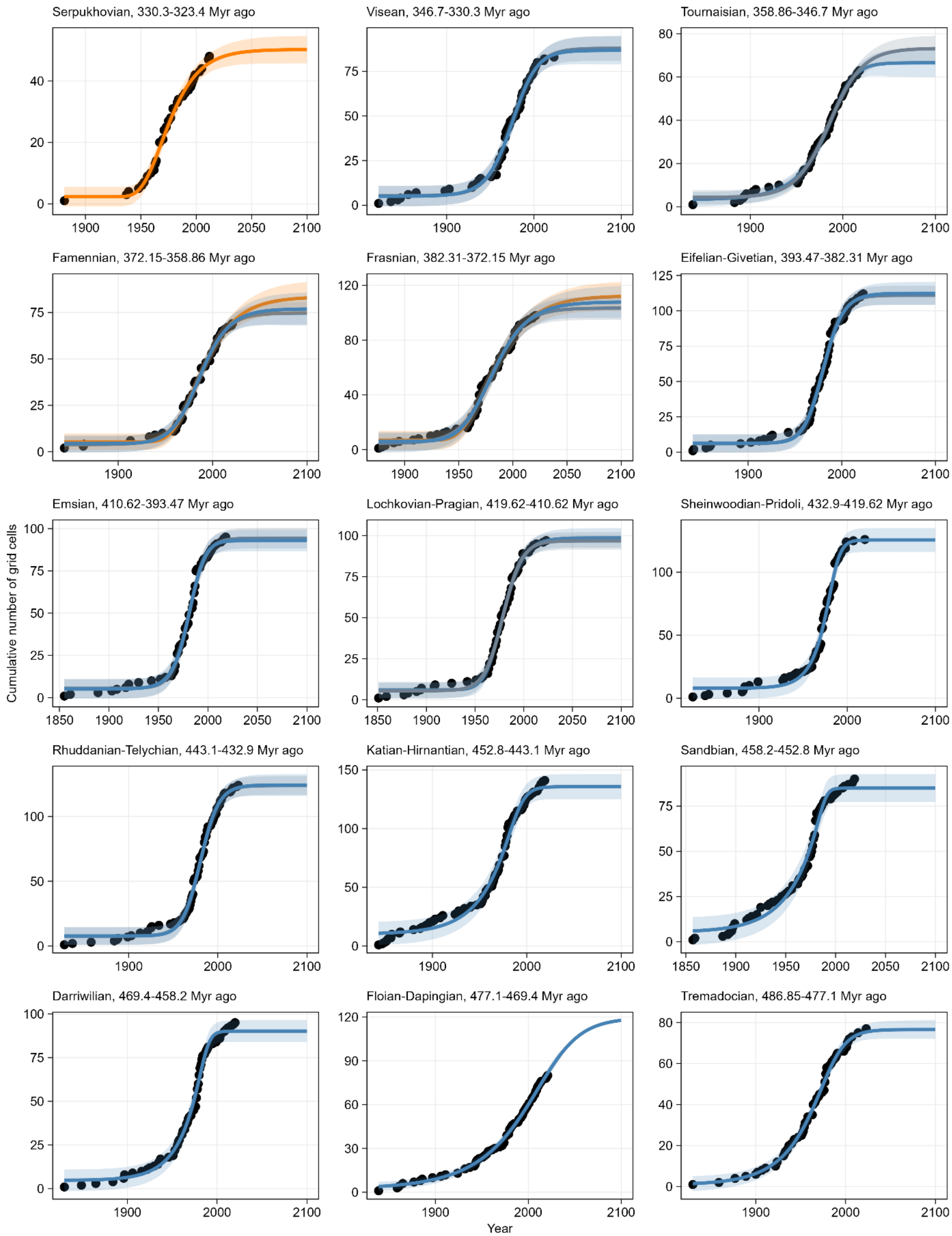

Fig. S41. Continued.

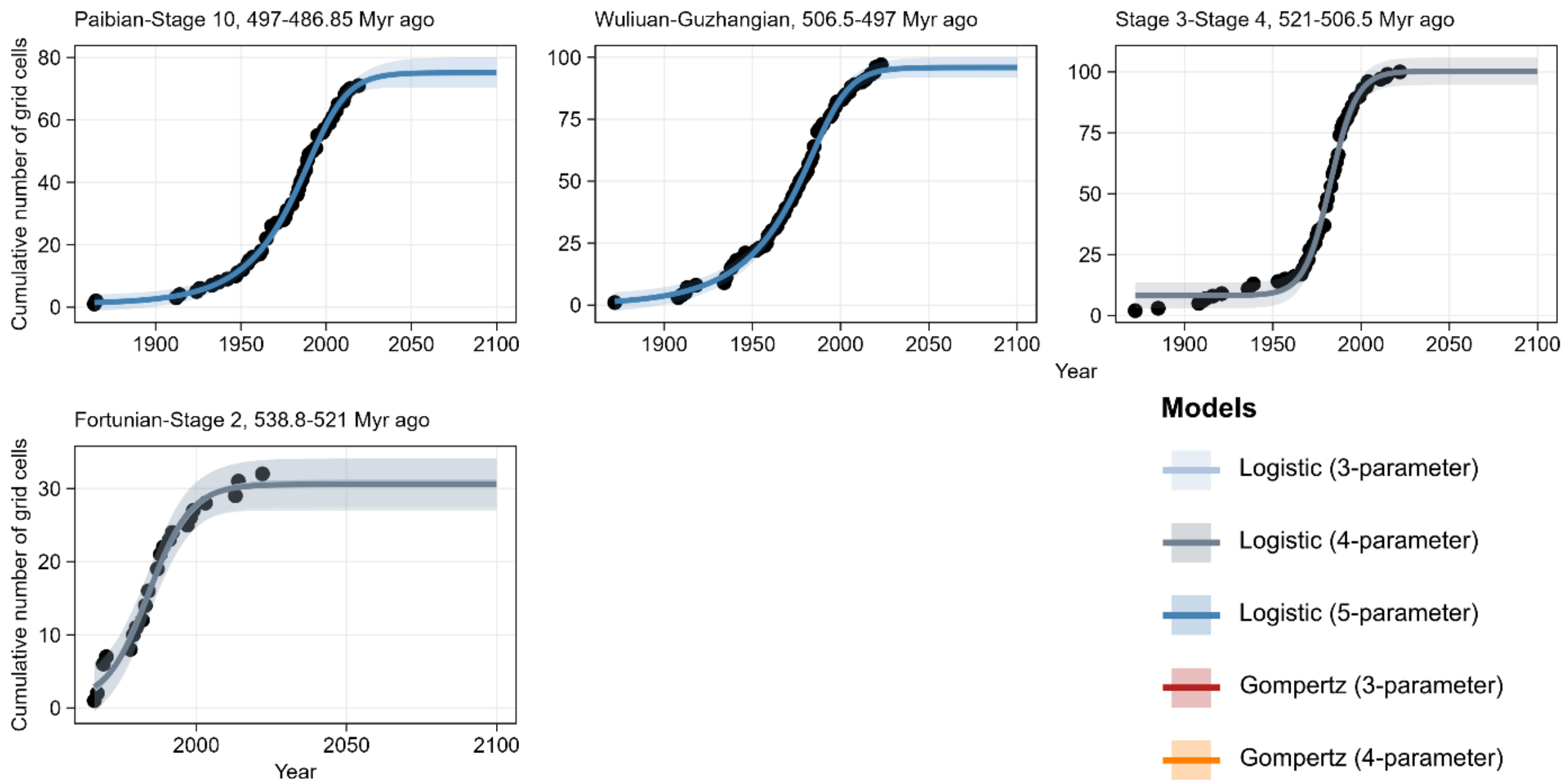

Fig. S41. Continued.

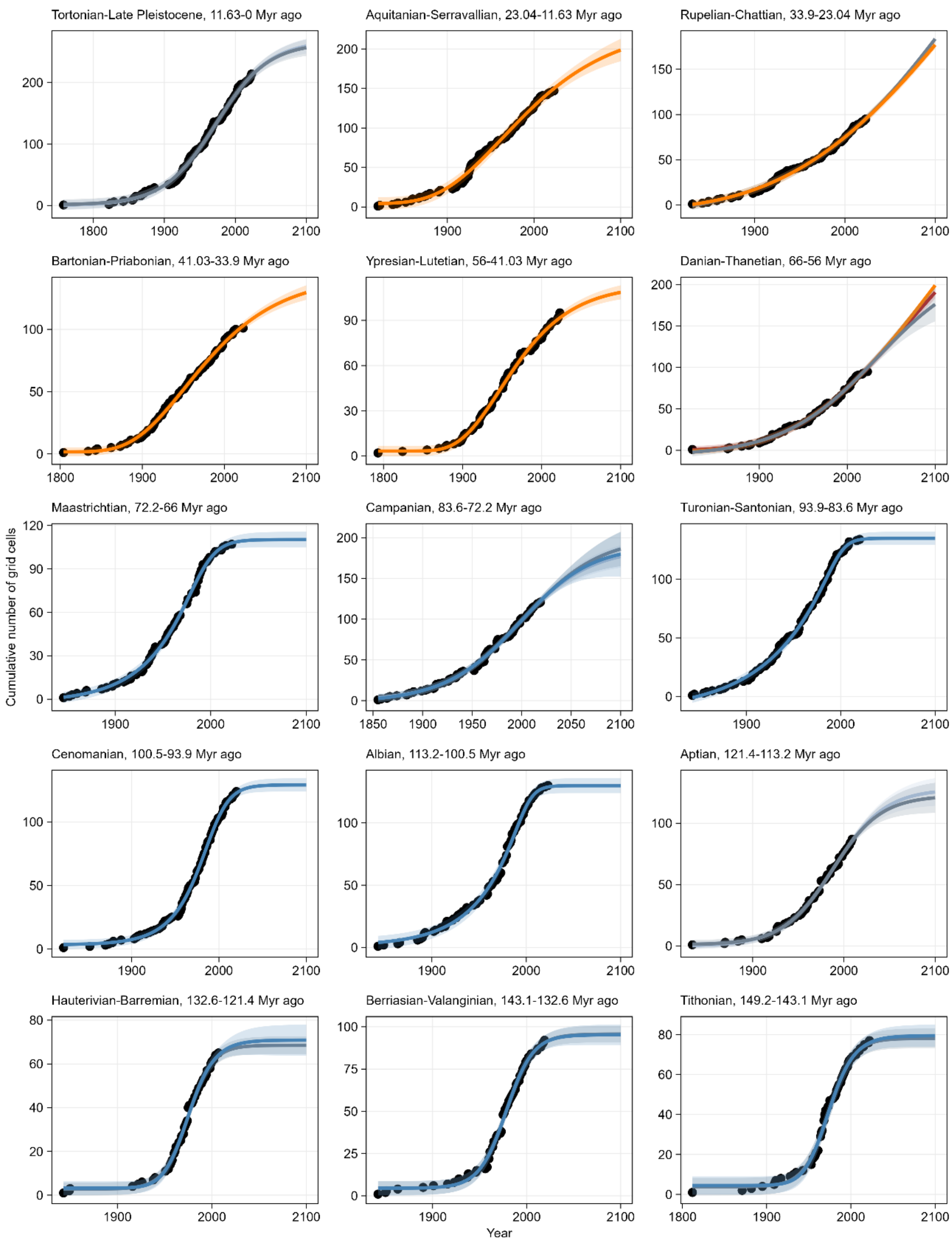

Fig. S42.

Growth models for the 49-bin approach predicting future collection effort across grid cells until the year 2100 for each time bin for the 750 km flat-topped grid (all marine data, not shallow-water only). Note that for some bins, more than one model qualified as the best (i.e., within ΔAIC = 2). Underlying data is summarized in table S12.

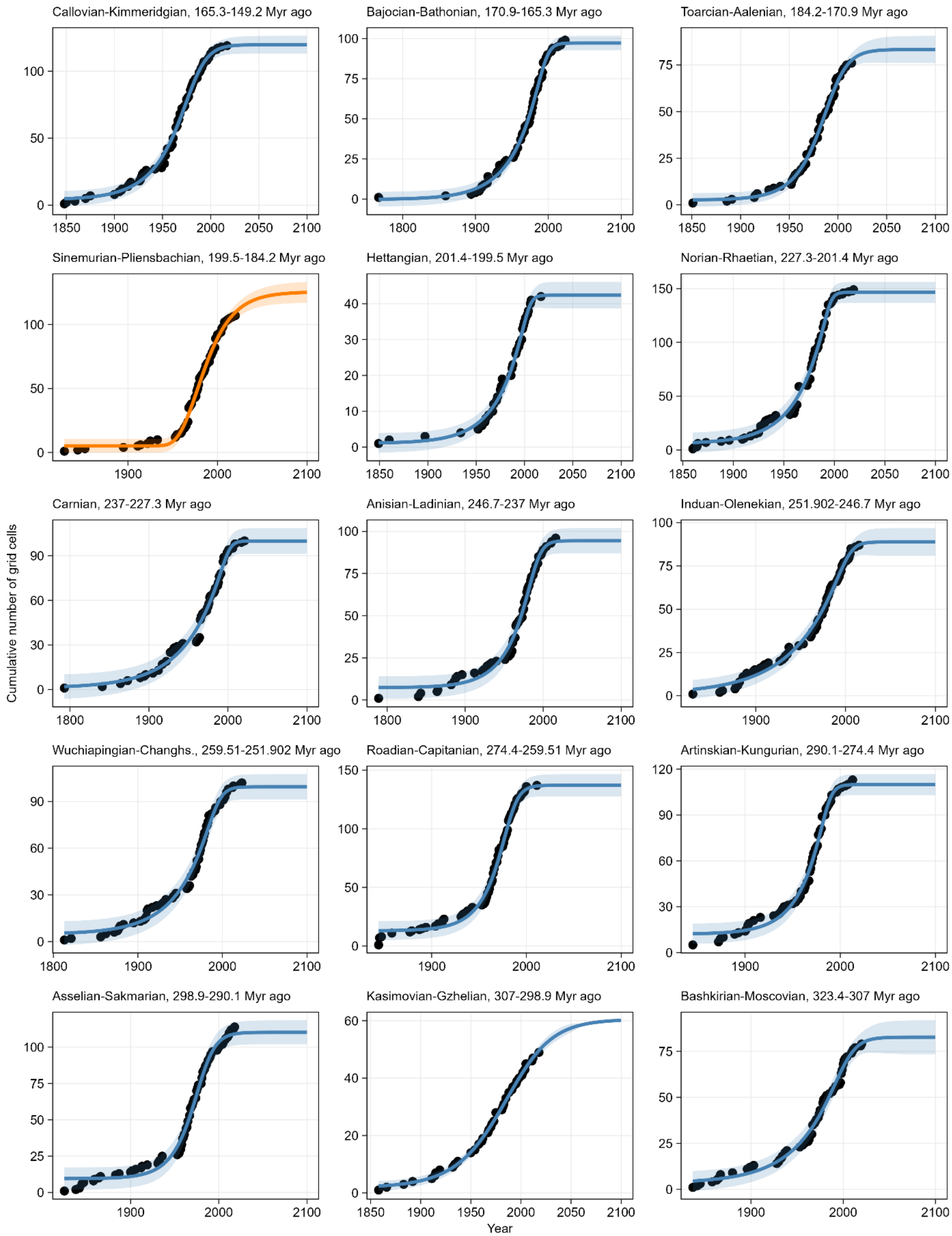

Fig. S42. Continued.

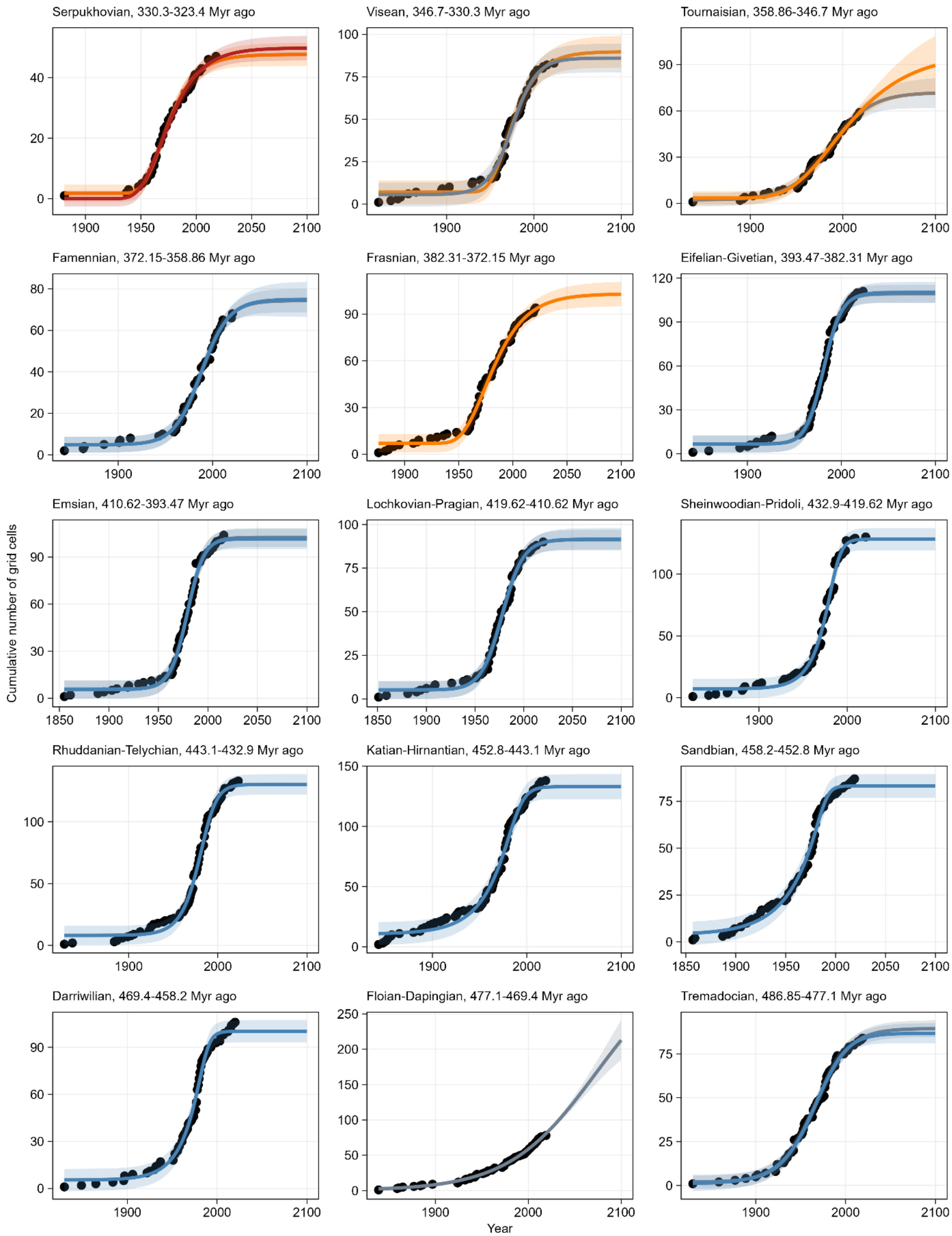

Fig. S42. Continued.

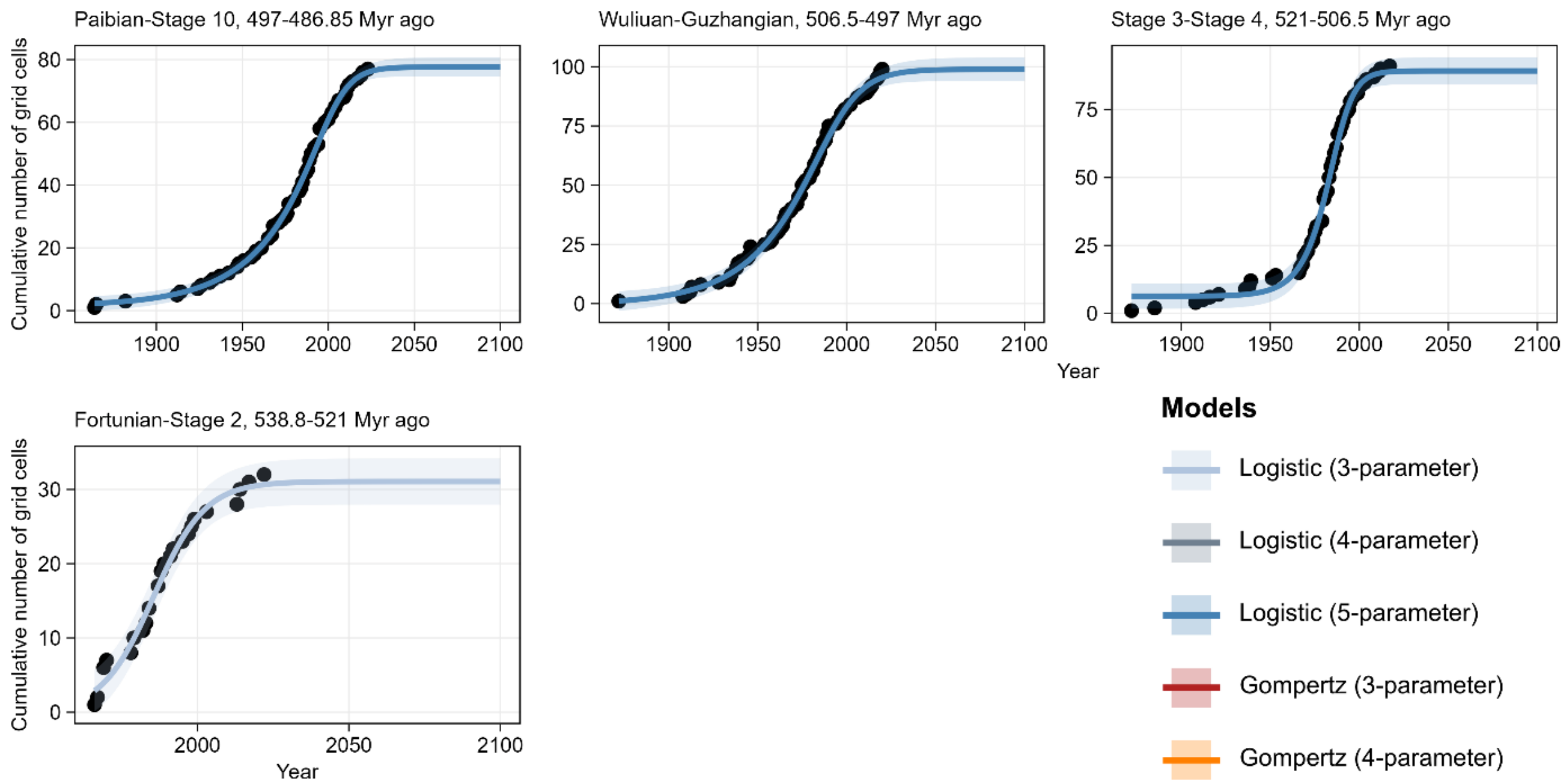

Fig. S42. Continued.

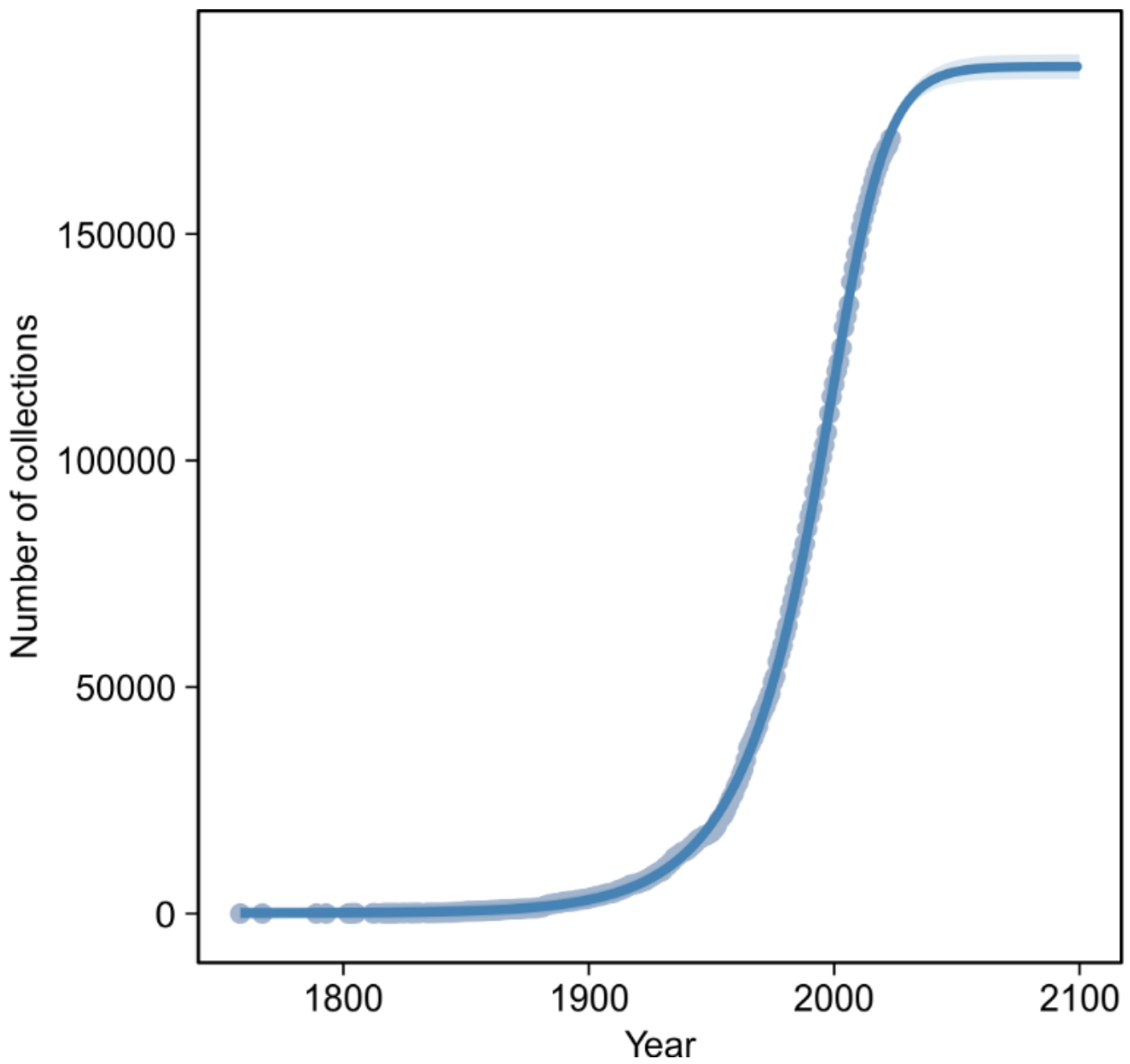

Fig. S43.

Total historical collection effort for the 49-bin approach across all marine assemblages, irrespective of grid or time bin. If trends persist, saturation is expected by the mid-21st century. The best model with lowest AIC is the 5-parameter logistic model (AIC = 3491.5, compared to AIC = 3828.1 for the second-best option, the 4-parameter logistic model).

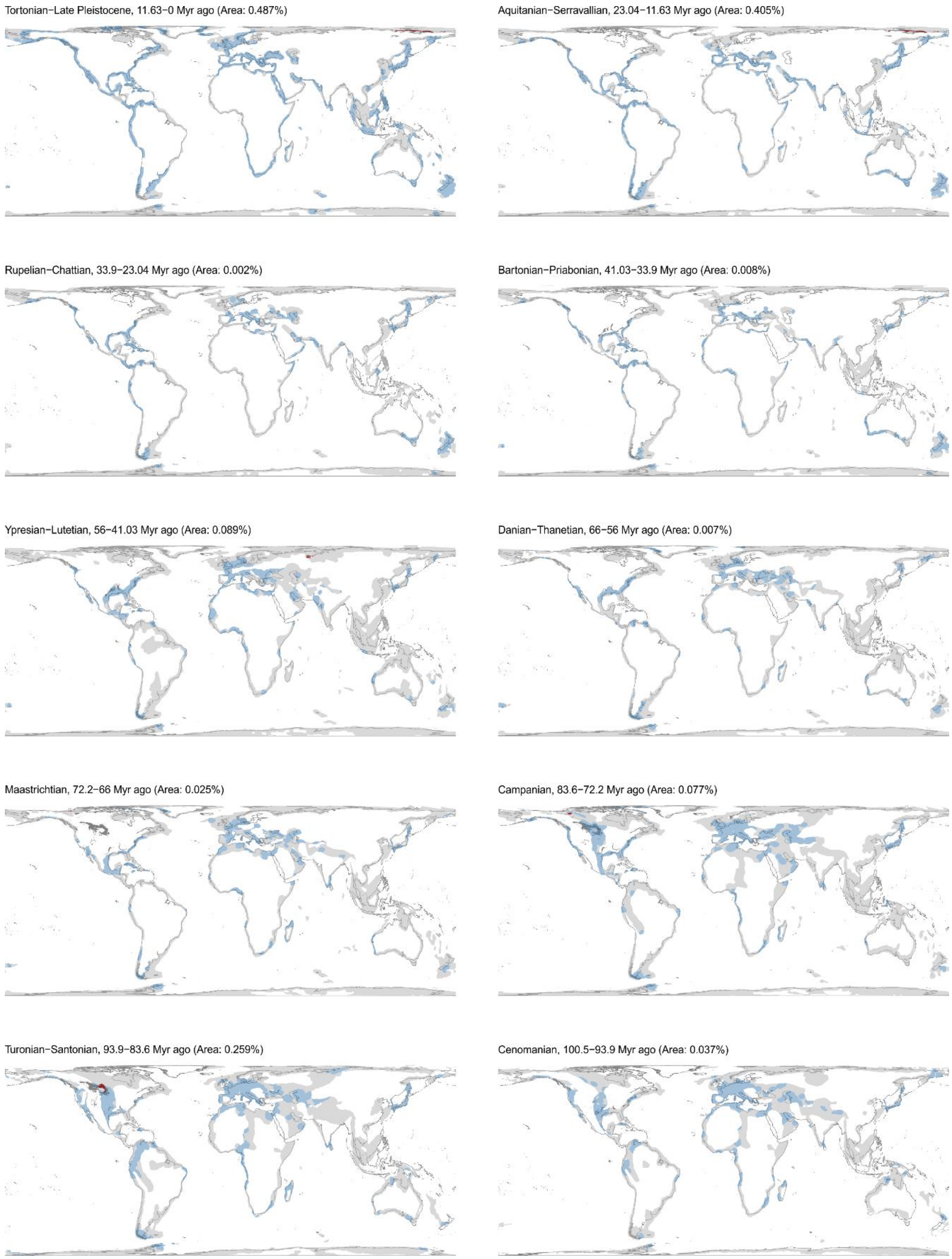

Fig. S44.

Reconstructed extents of past shallow-water areas and corresponding unexplored deposits for each time bin for the 49-bin approach. Data on deposits were retrieved from Macrostrat Database, applying the strict filter (see Methods). Data are summarized in tables S13–14.

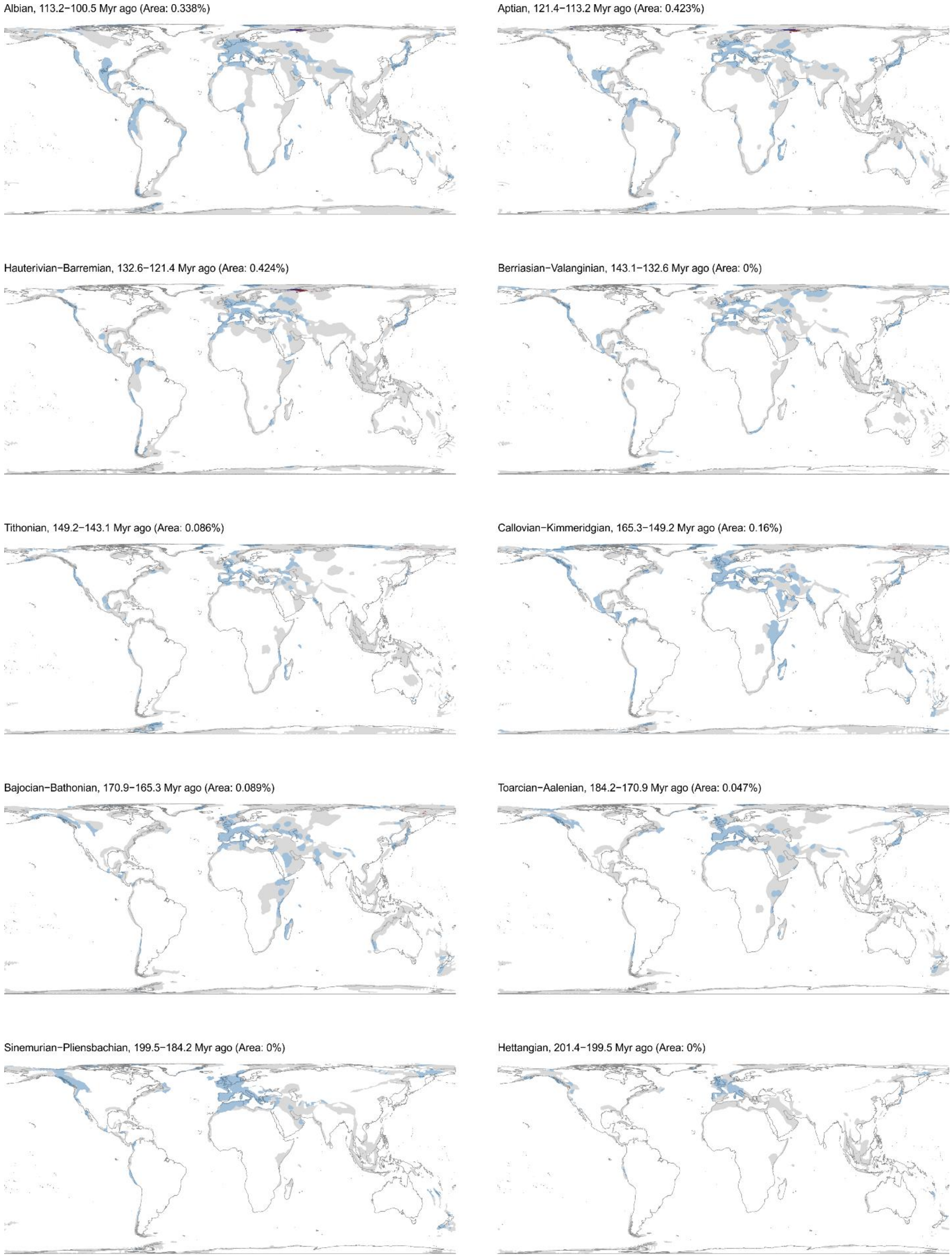

Fig. S44. Continued.

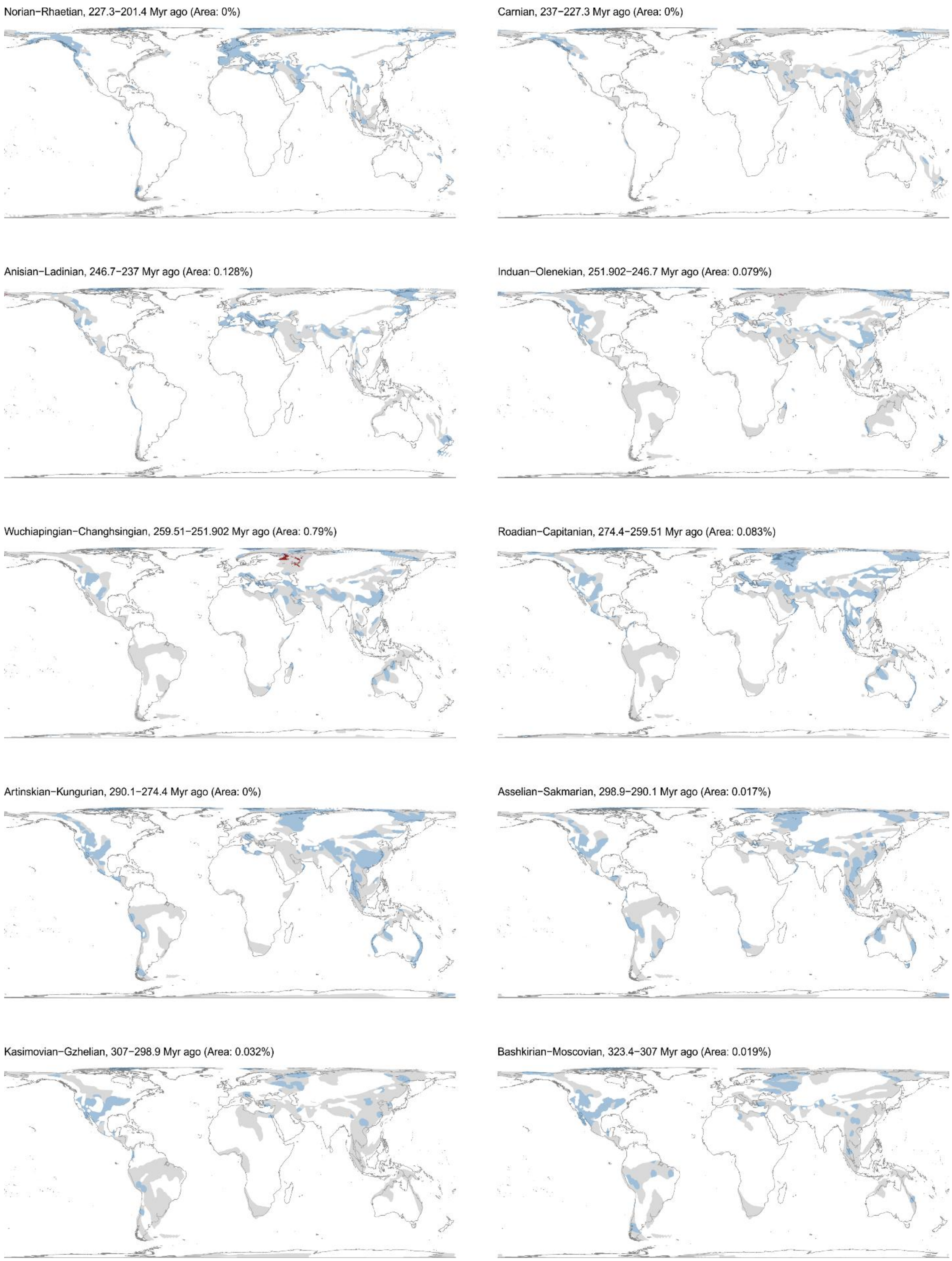

Fig. S44. Continued.

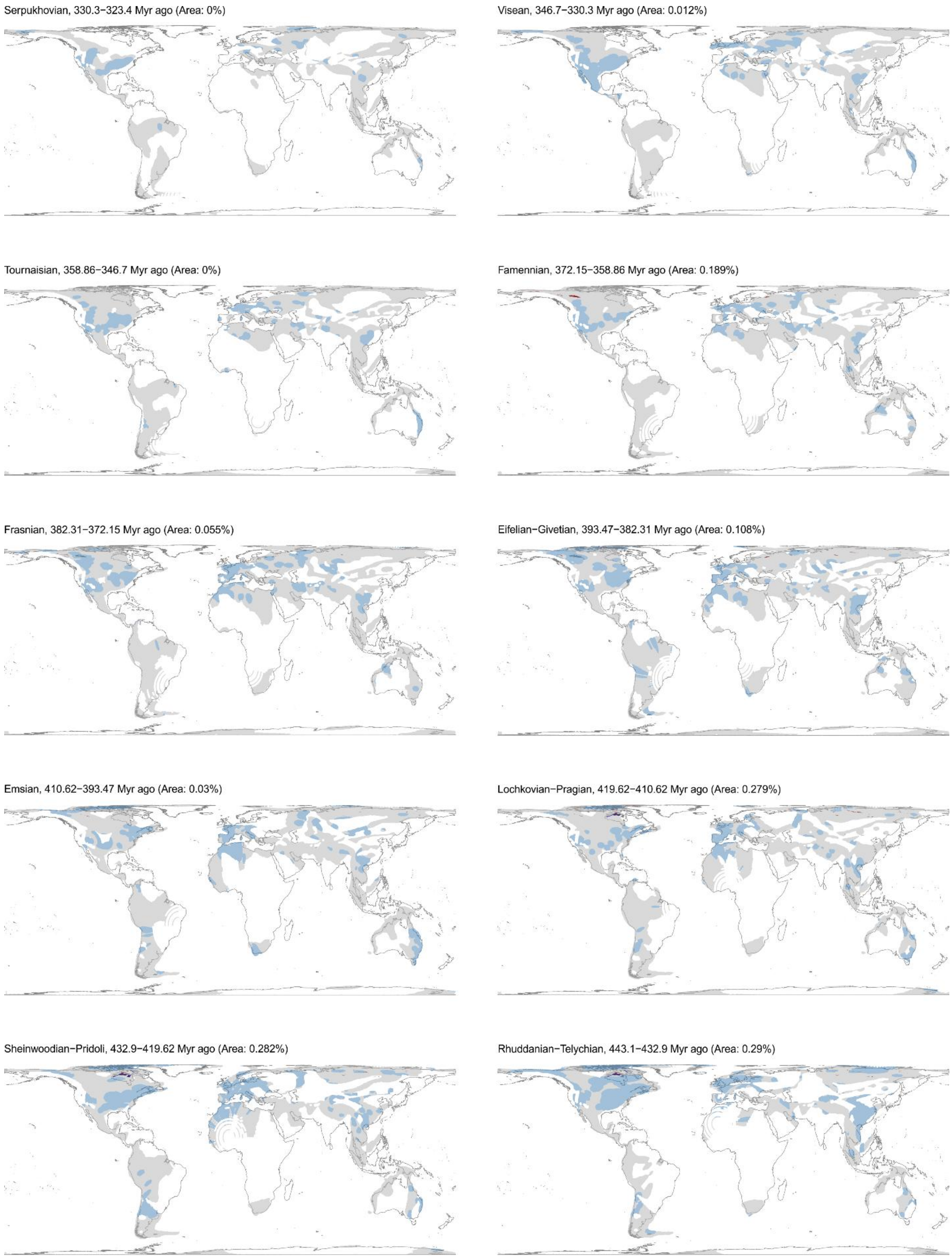

Fig. S44. Continued.

Katian−Hirnantian, 452.8−443.1 Myr ago (Area: 0.057%)

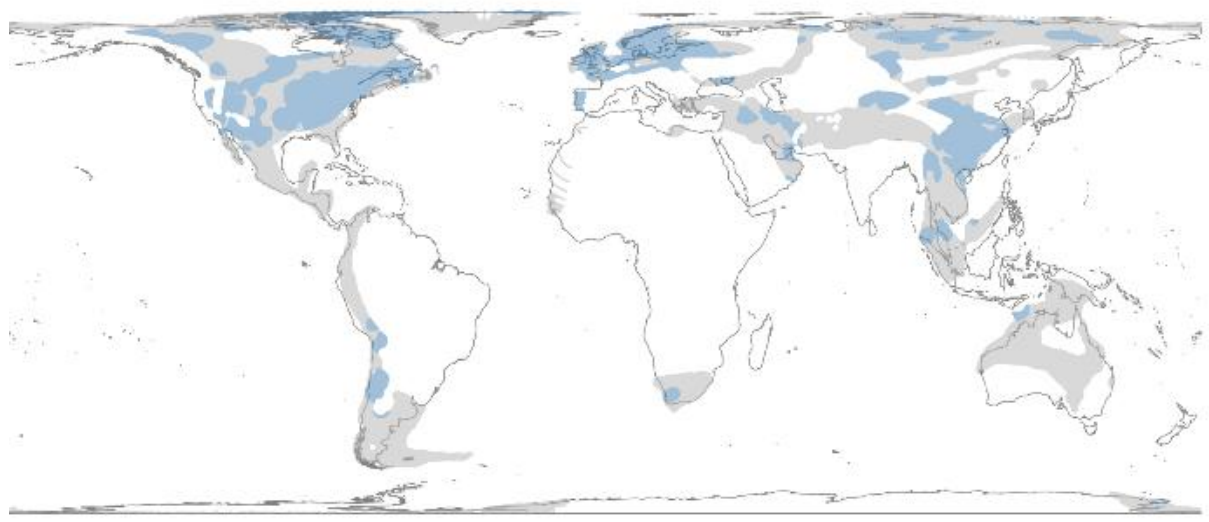

Sandbian, 458.2−452.8 Myr ago (Area: 0.003%)

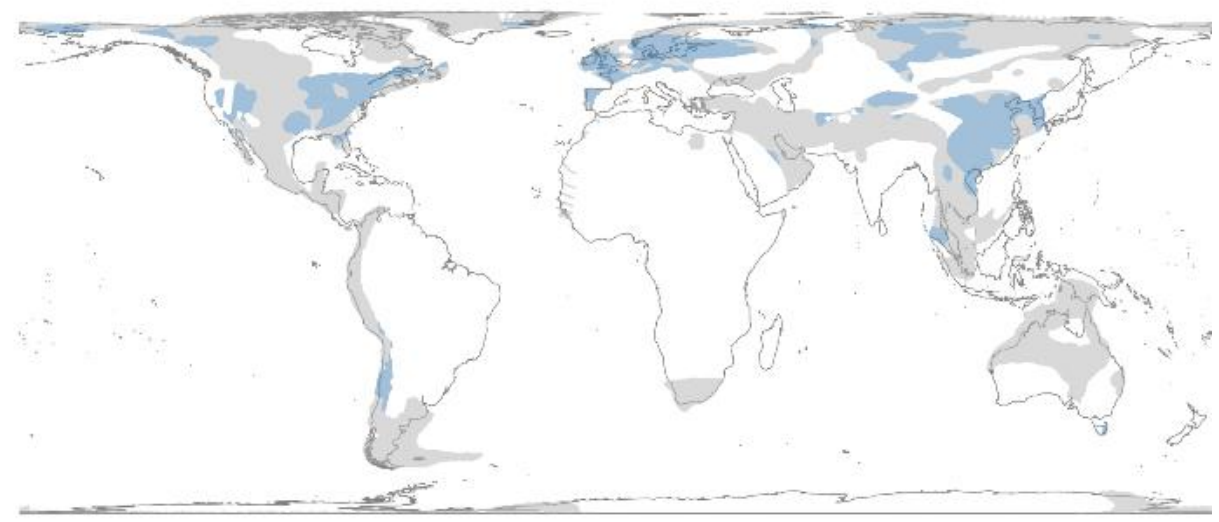

Darriwilian, 469.4−458.2 Myr ago (Area: 0.043%)

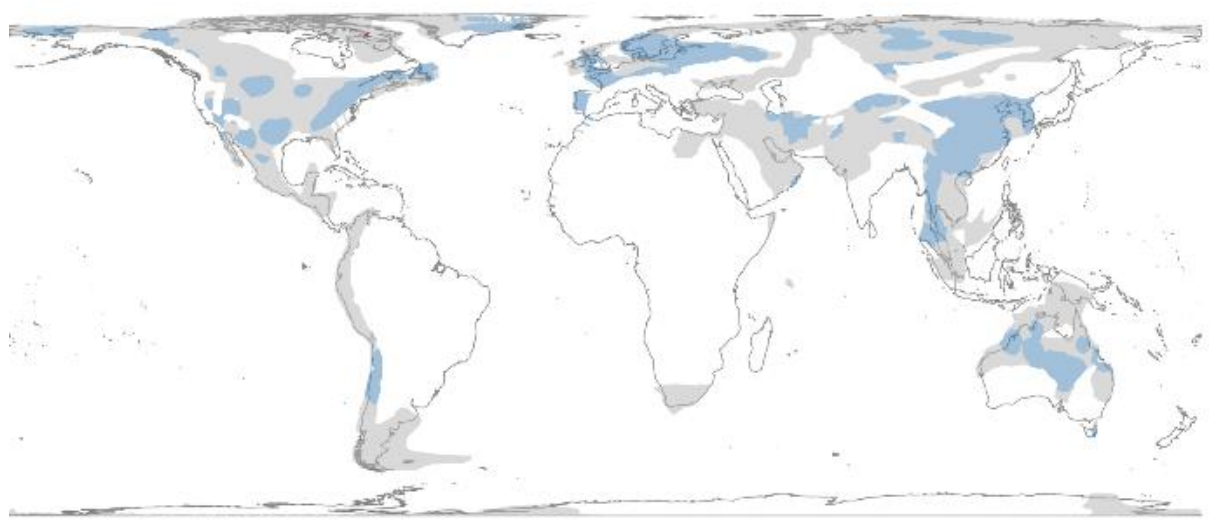

Floian−Dapingian, 477.1−469.4 Myr ago (Area: 0.183%)

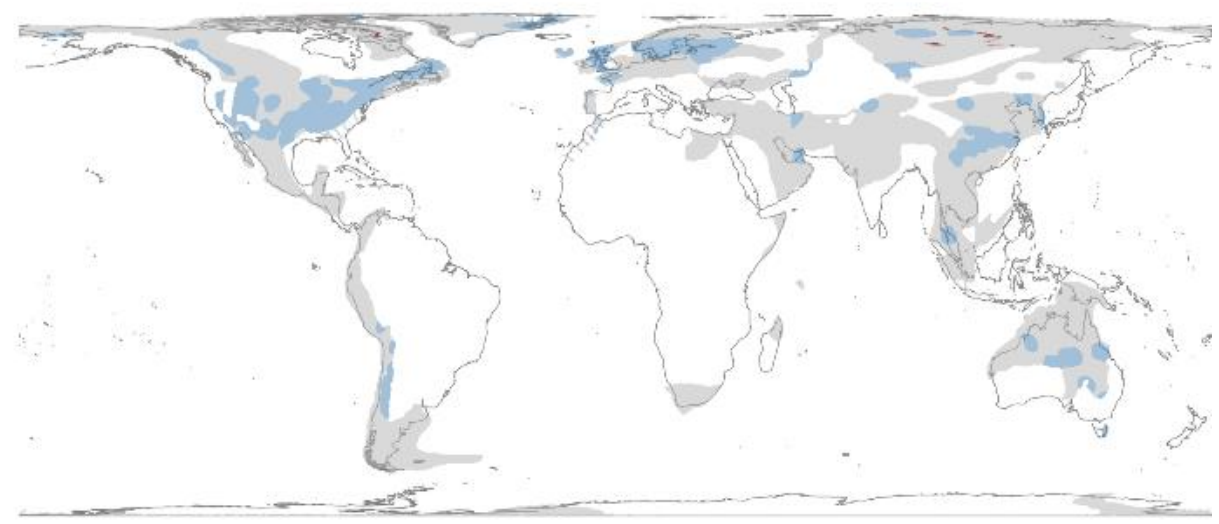

Tremadocian, 486.85−477.1 Myr ago (Area: 0.395%)

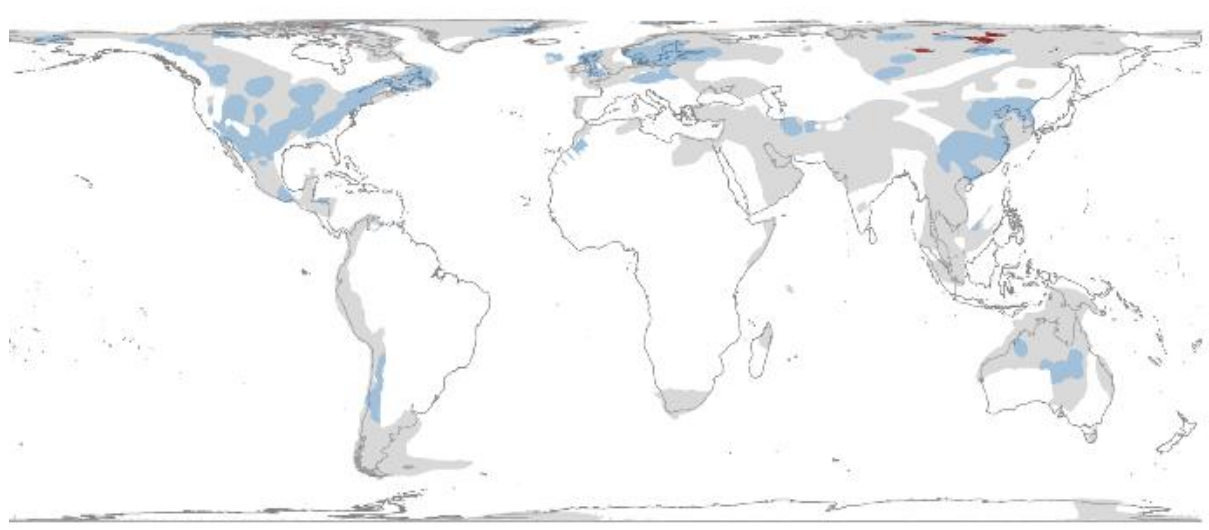

Paibian−Stage 10, 497−486.85 Myr ago (Area: 0.346%)

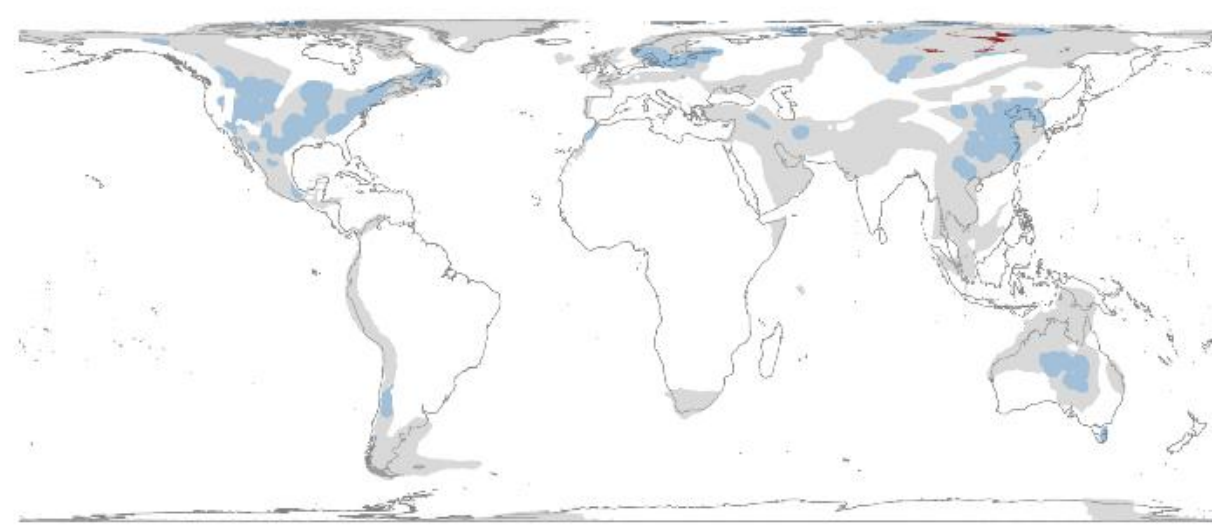

Wuliuan−Guzhangian, 506.5−497 Myr ago (Area: 0.104%)

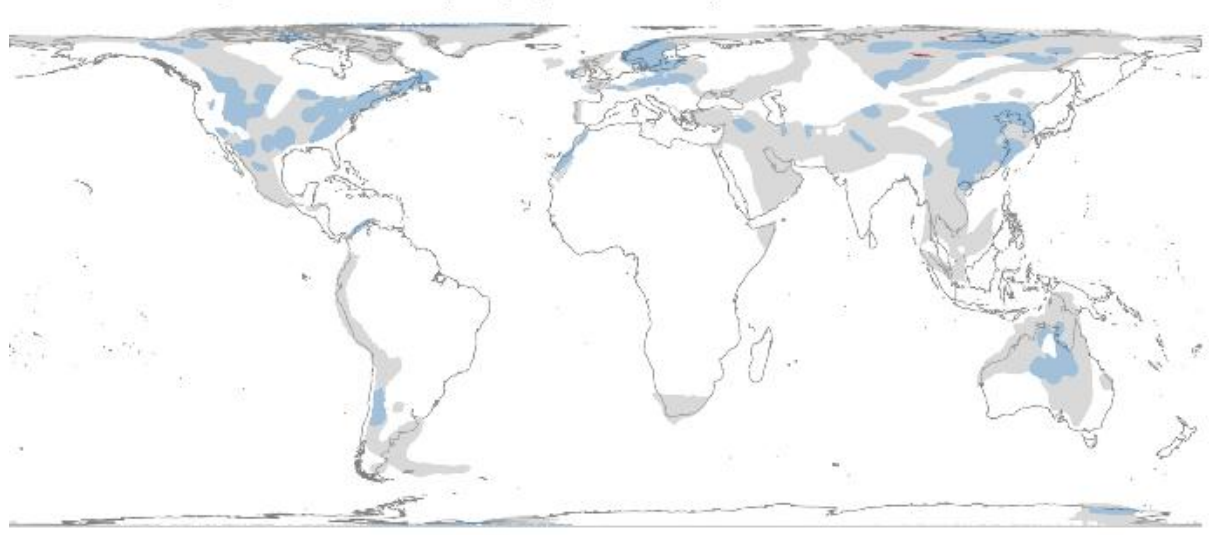

Stage 3−Stage 4, 521−506.5 Myr ago (Area: 0.084%)

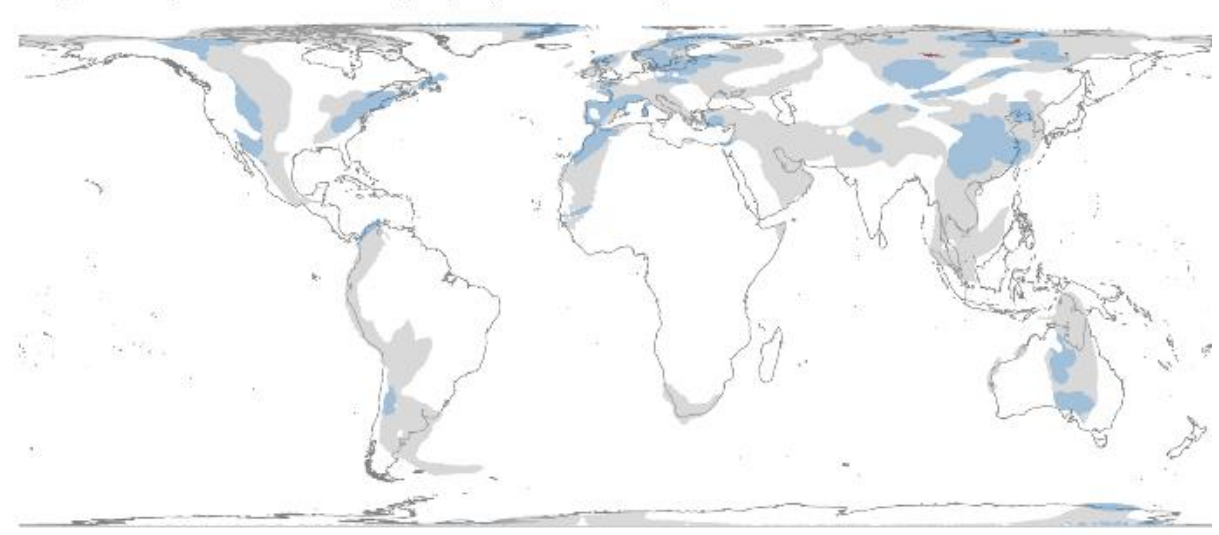

Fortunian−Stage 2, 538.8−521 Myr ago (Area: 0%)

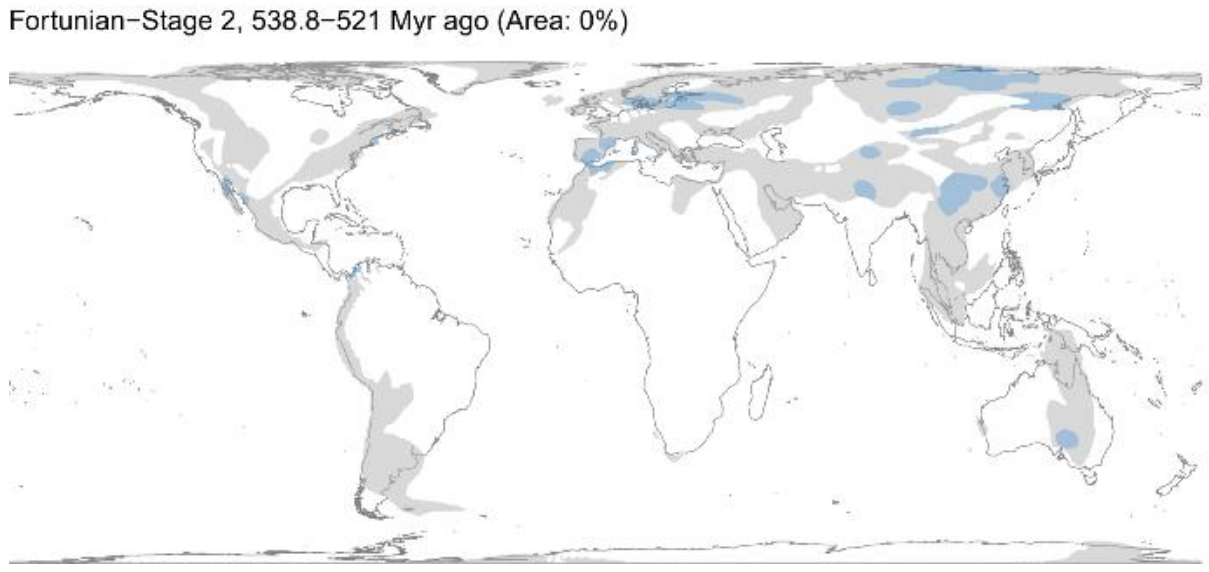

Fig. S44. Continued.

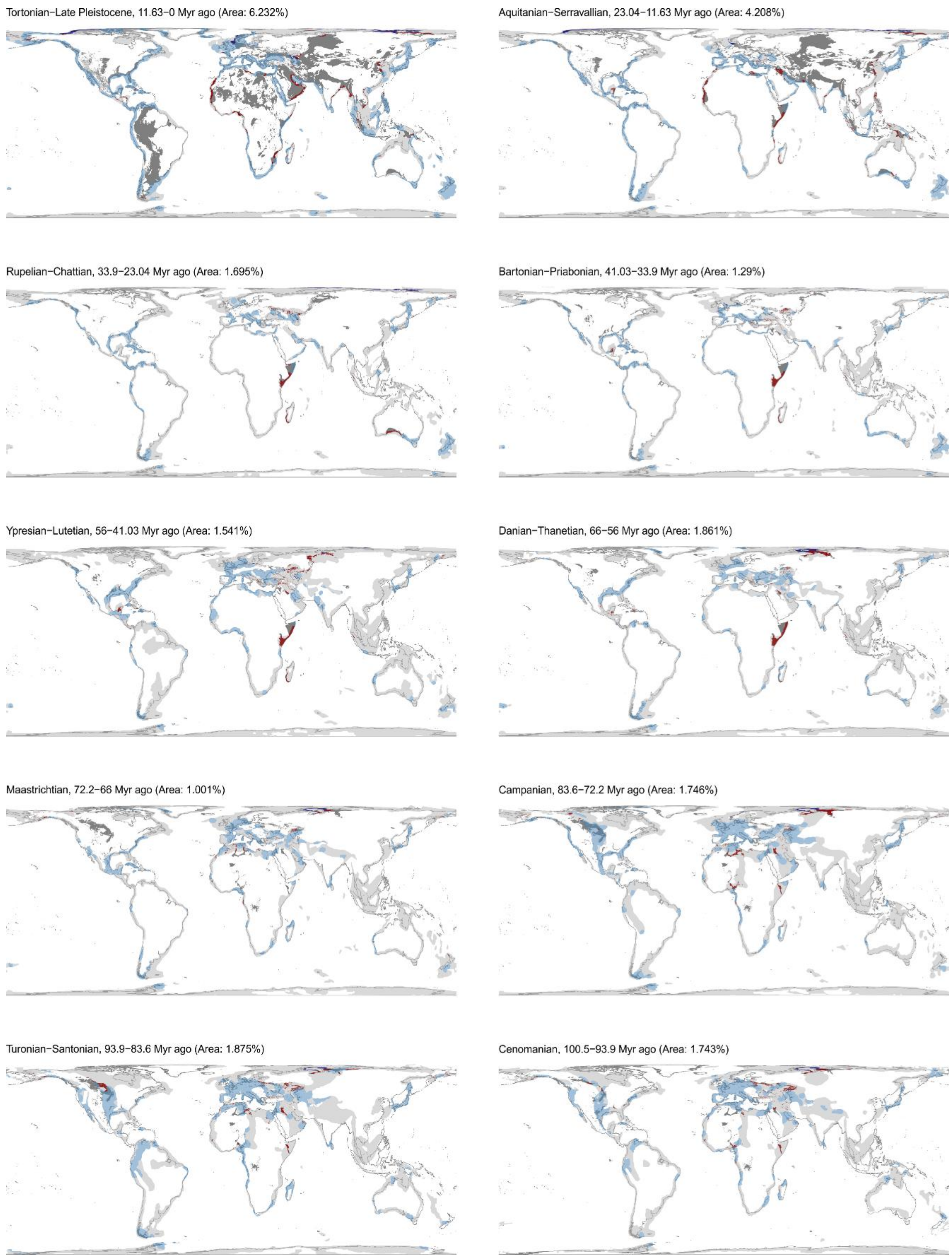

Fig. S45.

Reconstructed extents of past shallow-water areas and corresponding unexplored deposits for each time bin for the 49-bin approach. Data on deposits were retrieved from Macrostrat Database, applying the moderate filter (see Methods). Data are summarized in tables S13 and S15.

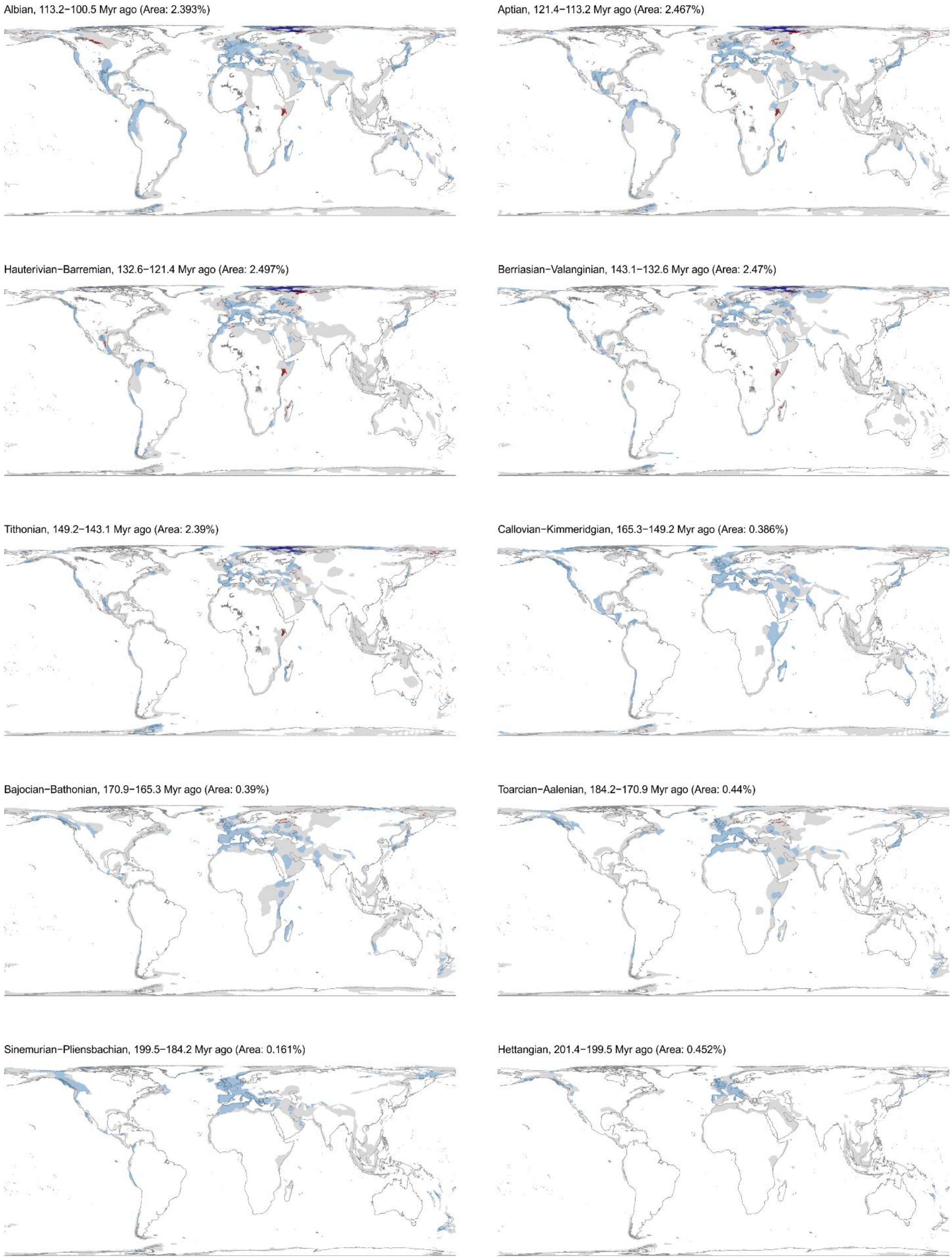

Fig. S45. Continued.

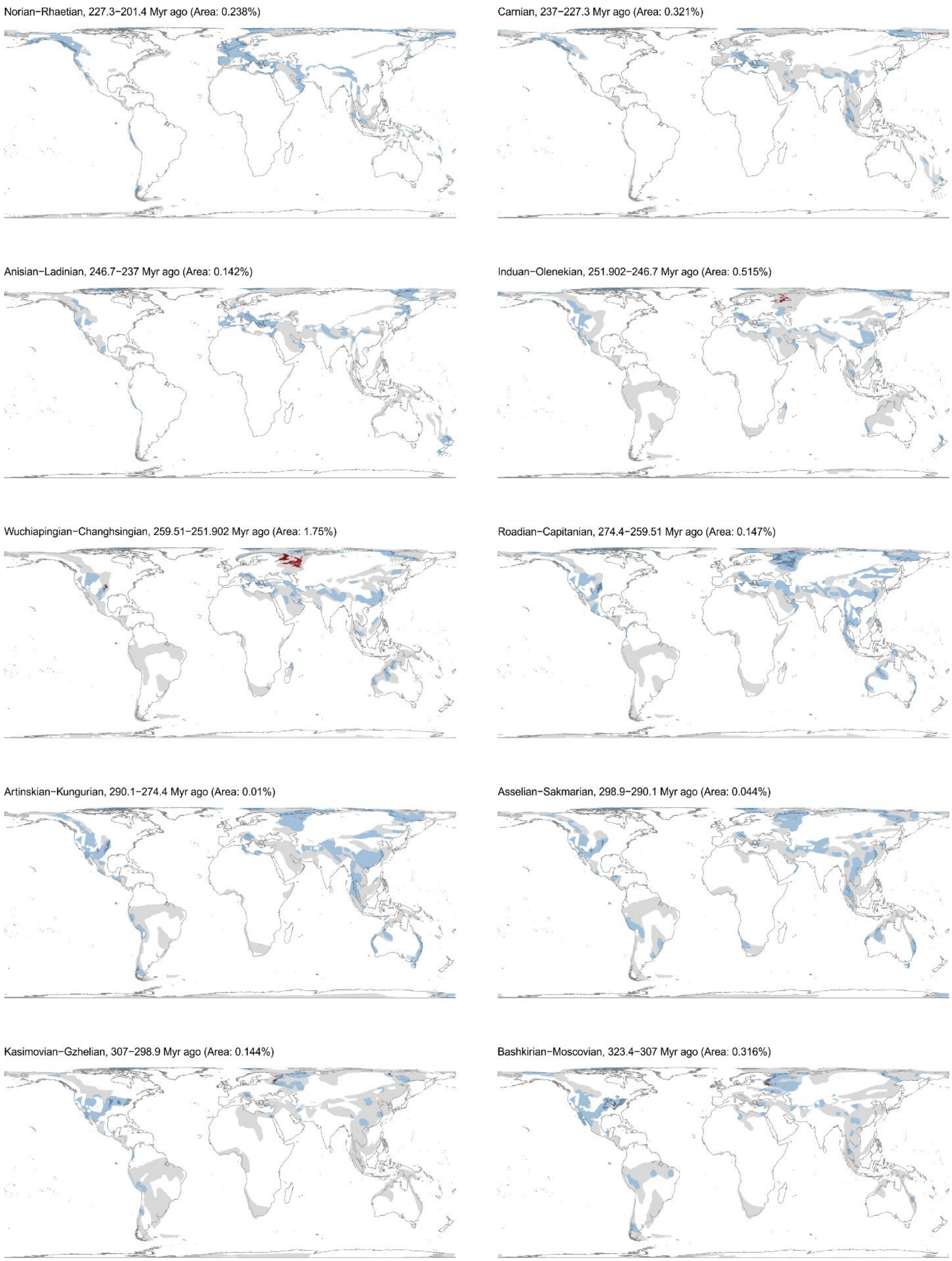

Fig. S45. Continued.

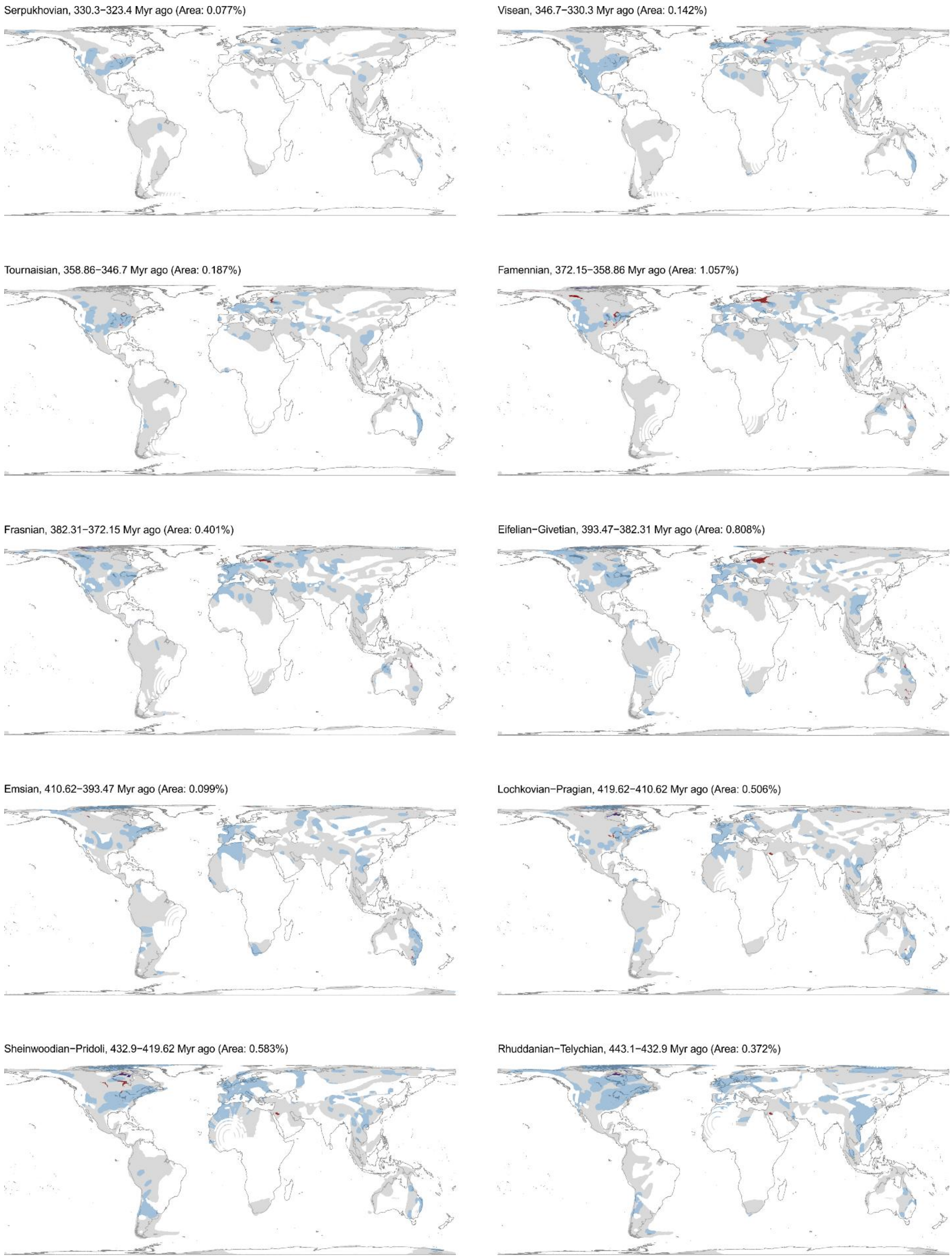

Fig. S45. Continued.

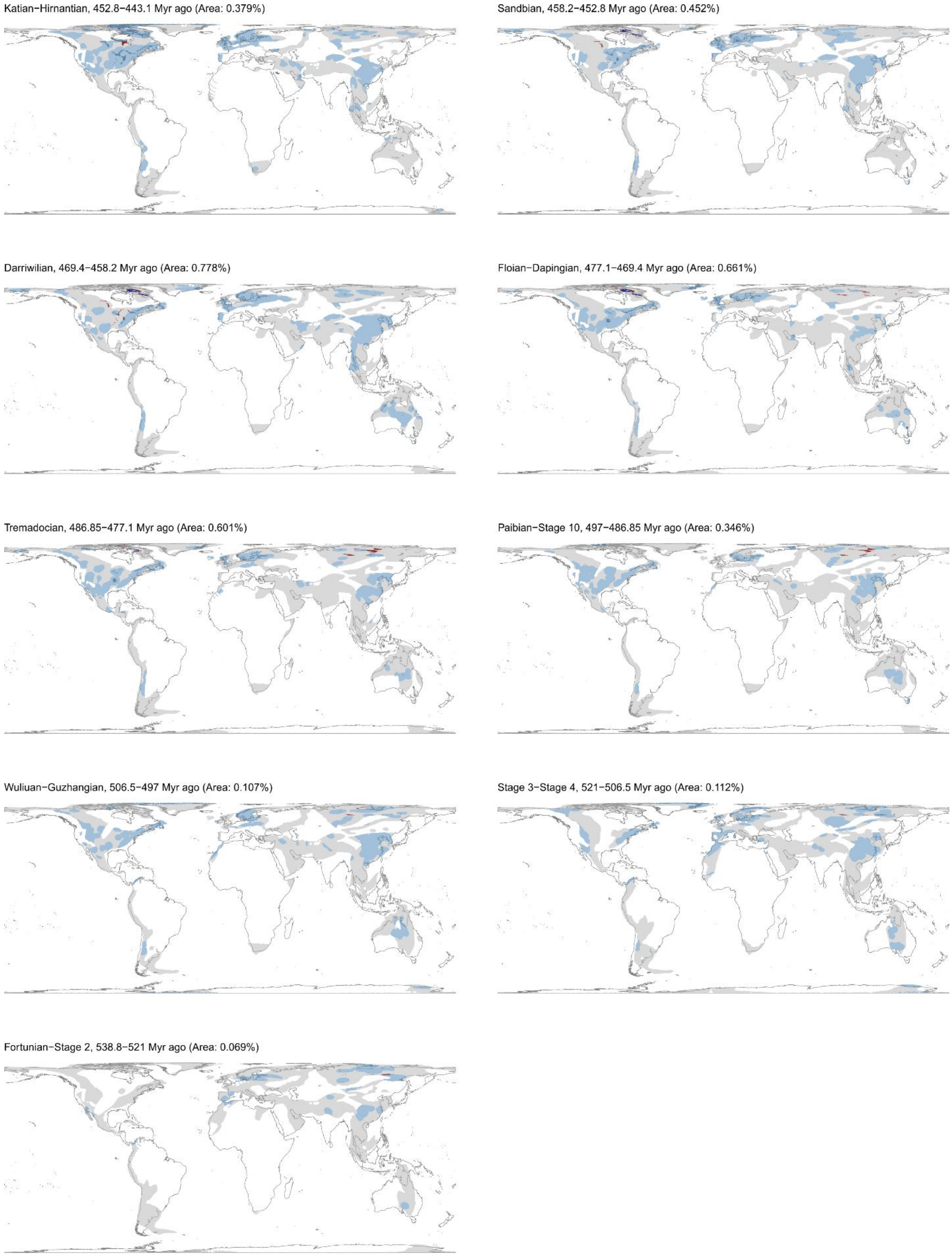

Fig. S45. Continued.

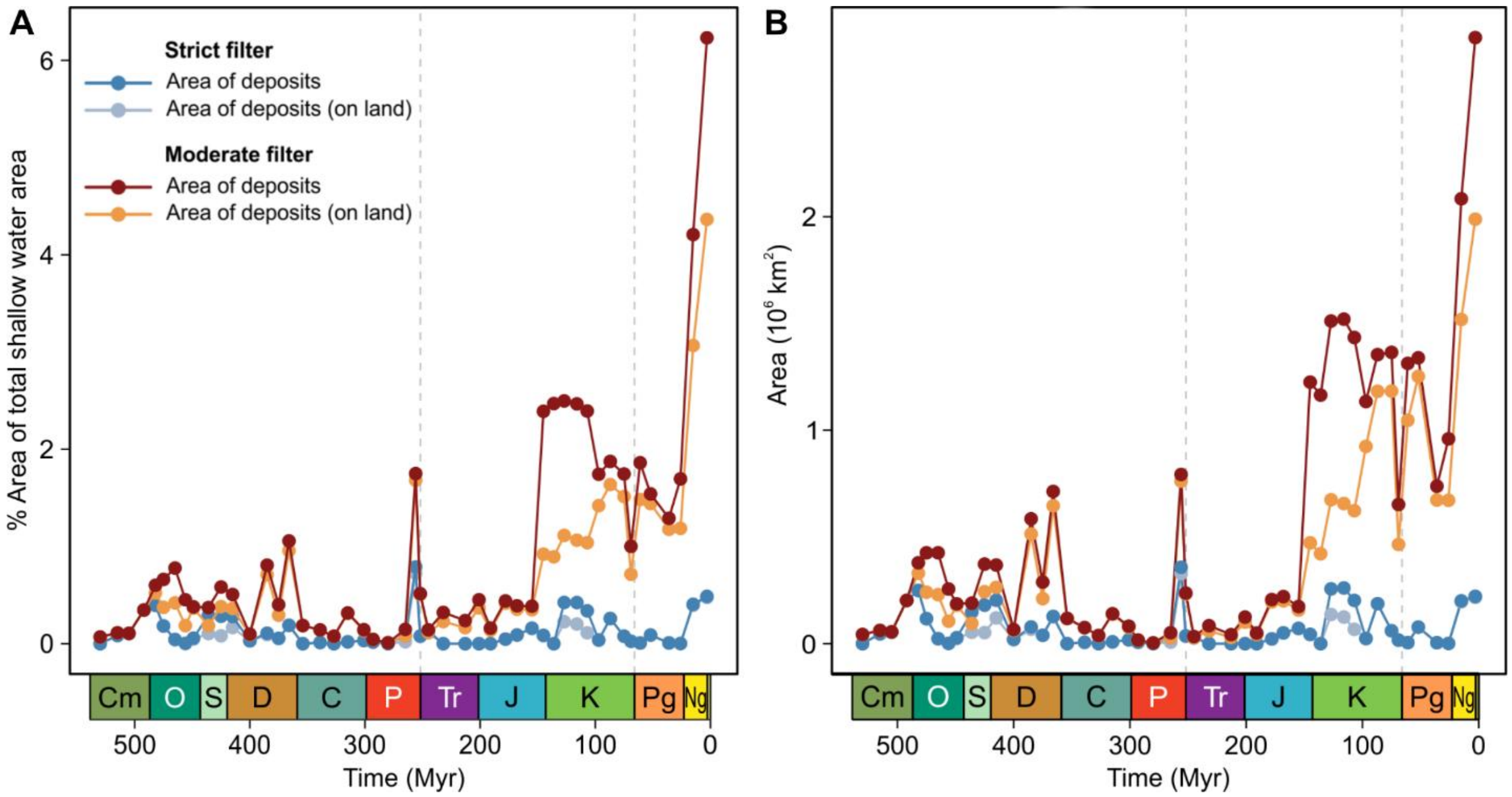

Fig. S46.

Areas of unexplored shallow-water deposits through time, for two data filters and distinguishing total area and deposits on land, for the 49-bin approach. (**A**) Areas in percent relative to total shallow water area; (**B**) total unexplored areas. See tables S14–15 for the underlying data.

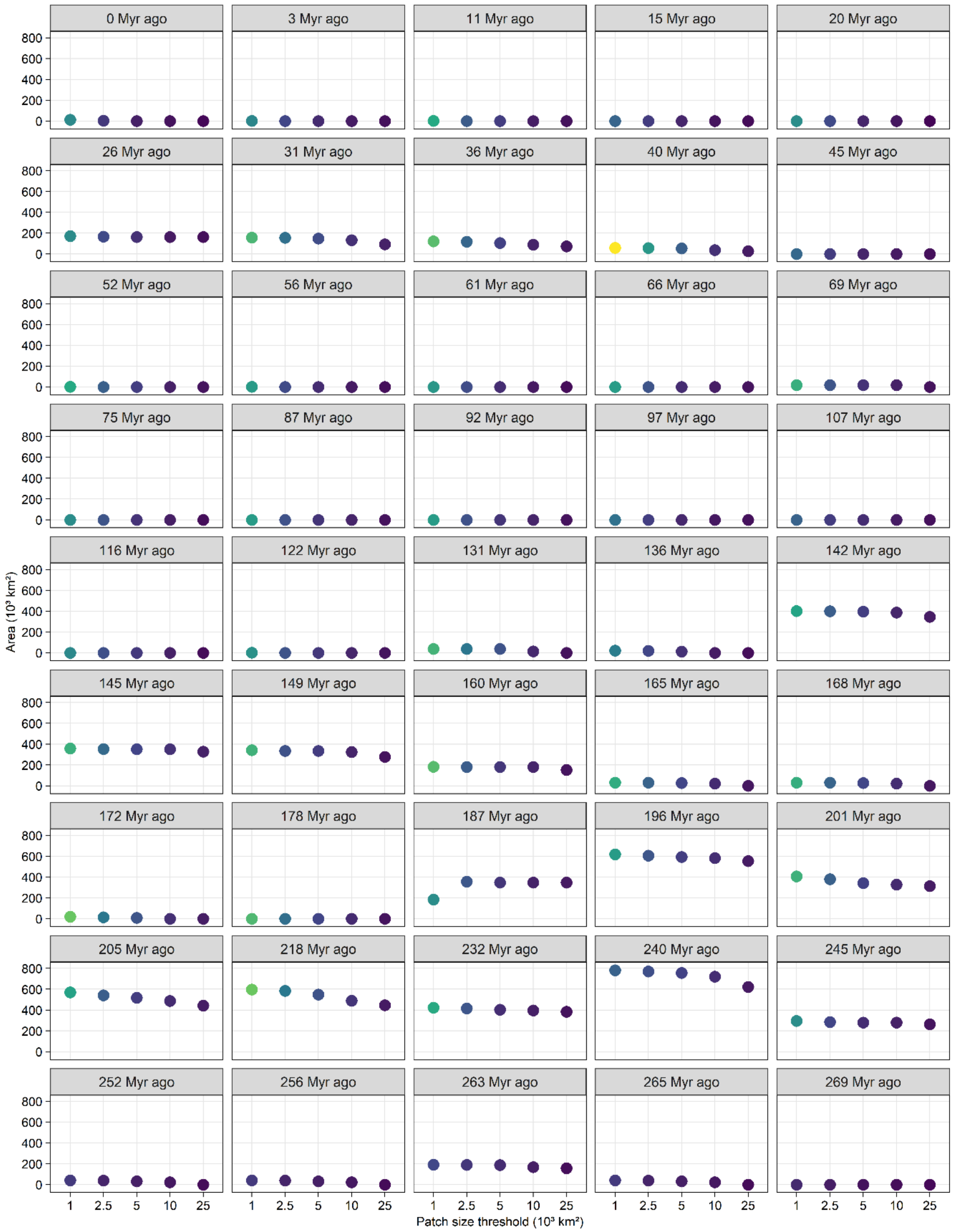

Fig. S47.

Results of the test for the impact of the chosen patch size threshold to determine inland depressions. Shown are the summed areas across isolated patches after discarding patches below 1000, 2500, 5000, 10,000, and 25,000 km², respectively; color corresponds to the number of patches. See table S16 for the underlying data.

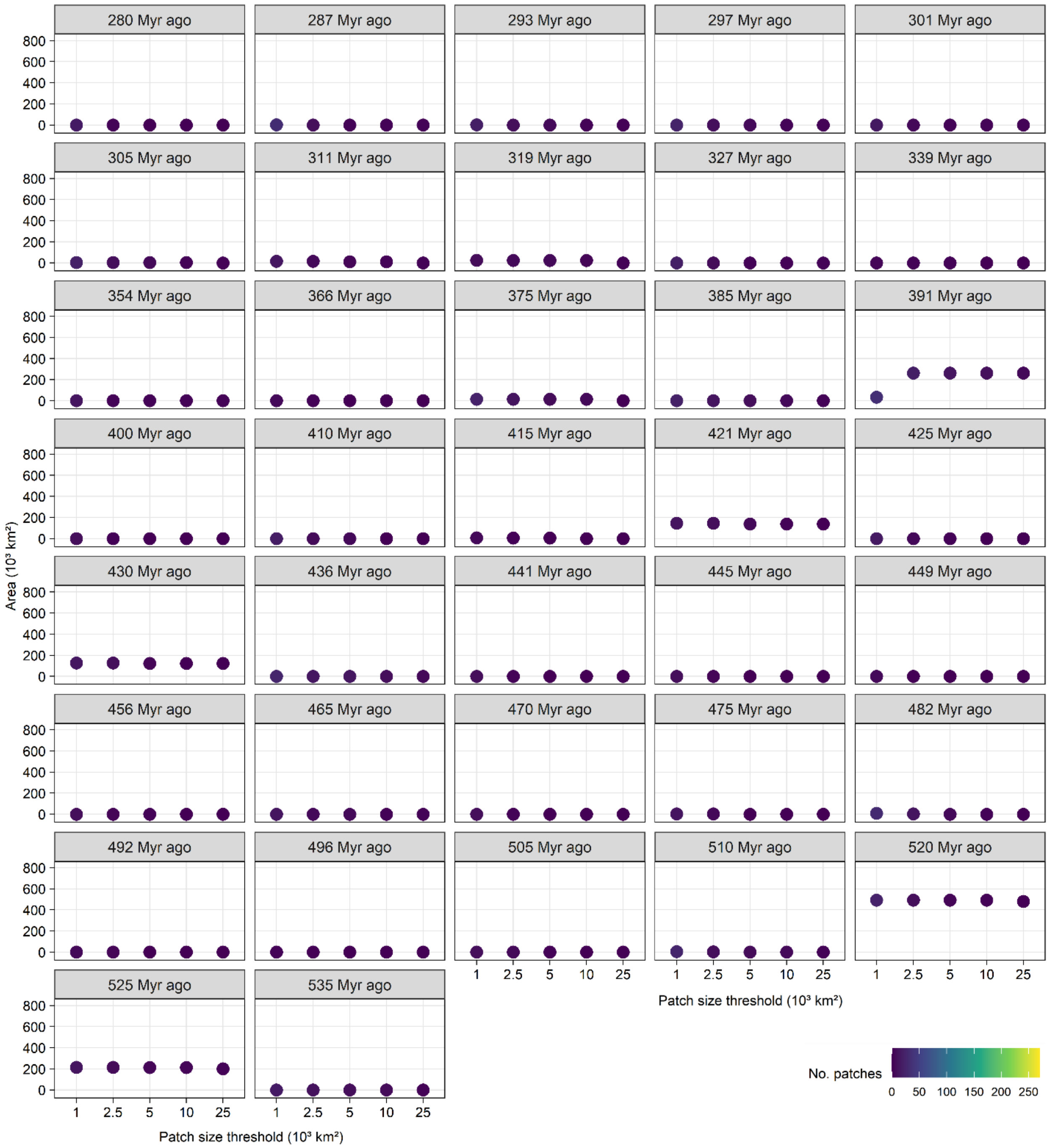

Fig. S47. Continued.

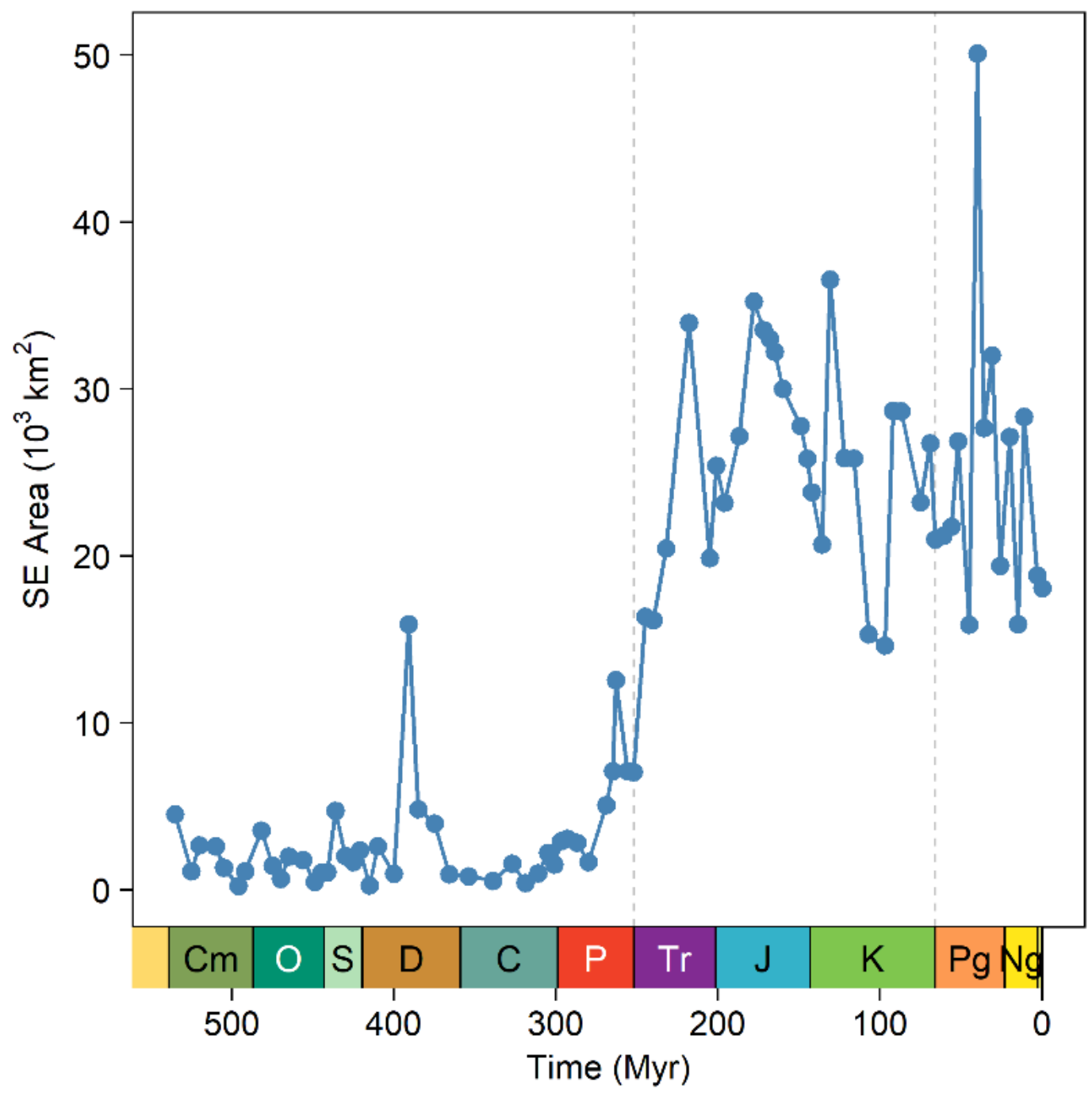

Fig. S48.
Results of the test for the impact of the chosen patch size threshold to determine inland depressions. Shown is the standard error of the summed areas of isolated patches across the five thresholds (1000, 2500, 5000, 10,000, and 25,000 km²) per map. See table S16 for the underlying data.

Table S1.

List of used R packages with version number and source.

Package name	Package version	Source
**CoordinateCleaner**	3.0.1	(*57*)
**deeptime**	2.3.1	(*58*)
**dplyr**	1.1.4	(*59*)
**drc**	3.0-1	(*60*)
**fossilbrush**	1.0.6	(*47*)
**furrr**	0.3.1	(*61*)
**future**	1.69.0	(*62*)
**ggplot2**	4.0.1	(*63*)
**gridExtra**	2.3	(*64*)
**httr2**	1.2.2	(*65*)
**MASS**	7.3-61	(*66*)
**mgcv**	1.9-4	(*53*)
**nlme**	3.1-166	(*67*, *68*)
**patchwork**	1.3.2	(*65*)
**palaeoverse**	1.4.0	(*49*)
**paleobioDB**	1.0.1	(*70*)
**permute**	0.9-8	(*71*)
**purrr**	1.2.0	(*72*)
**Rcpp**	1.1.1	(*73*)
**rgplates**	0.6.1	(*74*, *75*)
**rmacrostrat**	1.0.0	(*55*)
**rnaturalearth**	1.1.0	(*76*)
**sf**	1.0-19	(*77*, *78*)
**sfheaders**	0.4.5	(*79*)
**smoothr**	1.2.1	(*54*)
**statforbiology**	1.0.2	(*80*)
**stringr**	1.6.0	(*81*)
**terra**	1.7-83	(*82*)
**tidyr**	1.3.2	(*83*)
**vegan**	2.7-2	(*84*)

Table S2.
List of time bins used in the analyses and maps.

Bin identifier	Minimum age (Myr ago)	Maximum age (Myr ago)	Age range (Myr)	Nearest paleo-map (Salles et al. 2023)	Stratigraphic stages
**1**	0.774	0	0.774	0	Chibanian-Late Pleistocene
**2**	1.8	0.774	1.026	0	Calabrian
**3**	2.58	1.8	0.78	3	Gelasian
**4**	3.6	2.58	1.02	3	Piacenzian
**5**	5.333	3.6	1.733	3	Zanclean
**6**	7.246	5.333	1.913	3	Messinian
**7**	11.63	7.246	4.384	11	Tortonian
**8**	13.82	11.63	2.19	11	Serravallian
**9**	15.98	13.82	2.16	15	Langhian
**10**	20.45	15.98	4.47	20	Burdigalian
**11**	23.04	20.45	2.59	20	Aquitanian
**12**	27.3	23.04	4.26	26	Chattian
**13**	33.9	27.3	6.6	31	Rupelian
**14**	37.71	33.9	3.81	36	Priabonian
**15**	41.03	37.71	3.32	40	Bartonian
**16**	48.07	41.03	7.04	45	Lutetian
**17**	56	48.07	7.93	52	Ypresian
**18**	59.24	56	3.24	56	Thanetian
**19**	61.66	59.24	2.42	61	Selandian
**20**	66	61.66	4.34	66	Danian
**21**	72.2	66	6.2	69	Maastrichtian
**22**	83.6	72.2	11.4	75	Campanian
**23**	85.7	83.6	2.1	87	Santonian
**24**	89.8	85.7	4.1	87	Coniacian
**25**	93.9	89.8	4.1	92	Turonian
**26**	100.5	93.9	6.6	97	Cenomanian
**27**	113.2	100.5	12.7	107	Albian
**28**	121.4	113.2	8.2	116	Aptian
**29**	125.77	121.4	4.37	122	Barremian
**30**	132.6	125.77	6.83	131	Hauterivian
**31**	137.05	132.6	4.45	136	Valanginian
**32**	143.1	137.05	6.05	142	Berriasian
**33**	149.2	143.1	6.1	145	Tithonian
**34**	154.8	149.2	5.6	149	Kimmeridgian
**35**	161.5	154.8	6.7	160	Oxfordian
**36**	165.3	161.5	3.8	165	Callovian
**37**	168.2	165.3	2.9	168	Bathonian
**38**	170.9	168.2	2.7	168	Bajocian
**39**	174.7	170.9	3.8	172	Aalenian

**40**	184.2	174.7	9.5	178	Toarcian
**41**	192.9	184.2	8.7	187	Pliensbachian
**42**	199.5	192.9	6.6	196	Sinemurian
**43**	201.4	199.5	1.9	201	Hettangian
**44**	205.7	201.4	4.3	205	Rhaetian
**45**	227.3	205.7	21.6	218	Norian
**46**	237	227.3	9.7	232	Carnian
**47**	241.464	237	4.464	240	Ladinian
**48**	246.7	241.464	5.236	245	Anisian
**49**	249.9	246.7	3.2	245	Olenekian
**50**	251.902	249.9	2.002	252	Induan
**51**	254.14	251.902	2.238	252	Changhsingian
**52**	259.51	254.14	5.37	256	Wuchiapingian
**53**	264.28	259.51	4.77	263	Capitanian
**54**	266.9	264.28	2.62	265	Wordian
**55**	274.4	266.9	7.5	269	Roadian
**56**	283.3	274.4	8.9	280	Kungurian
**57**	290.1	283.3	6.8	287	Artinskian
**58**	293.52	290.1	3.42	293	Sakmarian
**59**	298.9	293.52	5.38	297	Asselian
**60**	303.7	298.9	4.8	301	Gzhelian
**61**	307	303.7	3.3	305	Kasimovian
**62**	315.2	307	8.2	311	Moscovian
**63**	323.4	315.2	8.2	319	Bashkirian
**64**	330.3	323.4	6.9	327	Serpukhovian
**65**	346.7	330.3	16.4	339	Visean
**66**	358.86	346.7	12.16	354	Tournaisian
**67**	372.15	358.86	13.29	366	Famennian
**68**	382.31	372.15	10.16	375	Frasnian
**69**	387.95	382.31	5.64	385	Givetian
**70**	393.47	387.95	5.52	391	Eifelian
**71**	410.62	393.47	17.15	400	Emsian
**72**	413.02	410.62	2.4	410	Pragian
**73**	419.62	413.02	6.6	415	Lochkovian
**74**	422.7	419.62	3.08	421	Pridoli
**75**	425	422.7	2.3	425	Ludfordian
**76**	426.7	425	1.7	425	Gorstian
**77**	430.6	426.7	3.9	430	Homerian
**78**	432.9	430.6	2.3	430	Sheinwoodian
**79**	438.6	432.9	5.7	436	Telychian
**80**	440.5	438.6	1.9	441	Aeronian
**81**	443.1	440.5	2.6	441	Rhuddanian
**82**	445.2	443.1	2.1	445	Hirnantian
**83**	452.8	445.2	7.6	449	Katian

**84**	458.2	452.8	5.4	456	Sandbian
**85**	469.4	458.2	11.2	465	Darriwilian
**86**	471.3	469.4	1.9	470	Dapingian
**87**	477.1	471.3	5.8	475	Floian
**88**	486.85	477.1	9.75	482	Tremadocian
**89**	491	486.85	4.15	492	Stage 10
**90**	494.2	491	3.2	492	Jiangshanian
**91**	497	494.2	2.8	496	Paibian
**92**	500.5	497	3.5	496	Guzhangian
**93**	504.5	500.5	4	505	Drumian
**94**	506.5	504.5	2	505	Wuliuan
**95**	514.5	506.5	8	510	Stage 4
**96**	521	514.5	6.5	520	Stage 3
**97**	529	521	8	525	Stage 2
**98**	538.8	529	9.8	535	Fortunian

Table S3.

List of time bins used in the analyses and maps for the 49-bin approach.

Bin identifier	Minimum age (Myr ago)	Maximum age (Myr ago)	Age range (Myr)	Nearest paleo-map (Salles et al. 2023)	Stratigraphic stages
**Cen6**	0	11.63	11.6183	3	Tortonian-Late Pleistocene
**Cen5**	11.63	23.04	11.41	15	Aquitanian-Serravallian
**Cen4**	23.04	33.9	10.86	26	Rupelian-Chattian
**Cen3**	33.9	41.03	7.13	36	Bartonian-Priabonian
**Cen2**	41.03	56	14.97	52	Ypresian-Lutetian
**Cen1**	56	66	10	61	Danian-Thanetian
**K8**	66	72.2	6.2	69	Maastrichtian
**K7**	72.2	83.6	11.4	75	Campanian
**K6**	83.6	93.9	10.3	87	Turonian-Santonian
**K5**	93.9	100.5	6.6	97	Cenomanian
**K4**	100.5	113.2	12.7	107	Albian
**K3**	113.2	121.4	8.2	116	Aptian
**K2**	121.4	132.6	11.2	127	Hauterivian-Barremian
**K1**	132.6	143.1	10.5	136	Berriasian-Valanginian
**J6**	143.1	149.2	6.1	145	Tithonian
**J5**	149.2	165.3	16.1	155	Callovian-Kimmeridgian
**J4**	165.3	170.9	5.6	168	Bajocian-Bathonian
**J3**	170.9	184.2	13.3	178	Toarcian-Aalenian
**J2**	184.2	199.5	15.3	191	Sinemurian-Pliensbachian
**J1**	199.5	201.4	1.9	201	Hettangian
**Tr4**	201.4	227.3	25.9	213	Norian-Rhaetian
**Tr3**	227.3	237	9.7	232	Carnian
**Tr2**	237	246.7	9.7	245	Anisian-Ladinian
**Tr1**	246.7	251.902	5.202	252	Induan-Olenekian
**P4**	251.902	259.51	7.608	256	Wuchiapingian-Changhsingian
**P3**	259.51	274.4	14.89	265	Roadian-Capitanian
**P2**	274.4	290.1	15.7	280	Artinskian-Kungurian
**P1**	290.1	298.9	8.8	293	Asselian-Sakmarian
**C5**	298.9	307	8.1	301	Kasimovian-Gzhelian
**C4**	307	323.4	16.4	315	Bashkirian-Moscovian
**C3**	323.4	330.3	6.9	327	Serpukhovian
**C2**	330.3	346.7	16.4	339	Visean
**C1**	346.7	358.86	12.16	354	Tournaisian
**D5**	358.86	372.15	13.29	366	Famennian
**D4**	372.15	382.31	10.16	375	Frasnian

**D3**	382.31	393.47	11.16	385	Eifelian-Givetian
**D2**	393.47	410.62	17.15	400	Emsian
**D1**	410.62	419.62	9	415	Lochkovian-Pragian
**S2**	419.62	432.9	13.28	425	Sheinwoodian-Pridoli
**S1**	432.9	443.1	10.2	436	Rhuddanian-Telychian
**Or5**	443.1	452.8	9.7	449	Katian-Hirnantian
**Or4**	452.8	458.2	5.4	456	Sandbian
**Or3**	458.2	469.4	11.2	465	Darriwilian
**Or2**	469.4	477.1	7.7	475	Floian-Dapingian
**Or1**	477.1	486.85	9.75	482	Tremadocian
**Cm4**	486.85	497	10.15	492	Paibian-Stage 10
**Cm3**	497	506.5	9.5	505	Wuliuan-Guzhangian
**Cm2**	506.5	521	14.5	515	Stage 3-Stage 4
**Cm1**	521	538.8	17.8	530	Fortunian-Stage 2

Table S4.
Summary statistics for the fossil collections across stratigraphic stages for the six grid types.

Stage	Map age (Myr)	No. cells with fossil data	No. cells overlapping shallow-water regions	% cells with data in shallow-water	Grid type
**Chibanian-Late Pleist.**	0	190	2700	7.04	250 km, pointy-topped
**Chibanian-Late Pleist.**	0	133	991	13.42	500 km, pointy-topped
**Chibanian-Late Pleist.**	0	112	575	19.48	750 km, pointy-topped
**Chibanian-Late Pleist.**	0	187	2610	7.16	250 km, flat-topped
**Chibanian-Late Pleist.**	0	130	1016	12.8	500 km, flat-topped
**Chibanian-Late Pleist.**	0	104	578	17.99	750 km, flat-topped
**Calabrian**	0	170	2700	6.3	250 km, pointy-topped
**Calabrian**	0	120	991	12.11	500 km, pointy-topped
**Calabrian**	0	100	575	17.39	750 km, pointy-topped
**Calabrian**	0	170	2605	6.53	250 km, flat-topped
**Calabrian**	0	118	1018	11.59	500 km, flat-topped
**Calabrian**	0	95	577	16.46	750 km, flat-topped
**Gelasian**	3	31	2229	1.39	250 km, pointy-topped
**Gelasian**	3	25	820	3.05	500 km, pointy-topped
**Gelasian**	3	21	468	4.49	750 km, pointy-topped
**Gelasian**	3	29	2139	1.36	250 km, flat-topped
**Gelasian**	3	22	848	2.59	500 km, flat-topped
**Gelasian**	3	22	468	4.7	750 km, flat-topped
**Piacenzian**	3	99	2232	4.44	250 km, pointy-topped
**Piacenzian**	3	78	823	9.48	500 km, pointy-topped
**Piacenzian**	3	61	469	13.01	750 km, pointy-topped
**Piacenzian**	3	99	2142	4.62	250 km, flat-topped
**Piacenzian**	3	68	850	8	500 km, flat-topped
**Piacenzian**	3	60	468	12.82	750 km, flat-topped
**Zanclean**	3	200	2245	8.91	250 km, pointy-topped
**Zanclean**	3	133	824	16.14	500 km, pointy-topped
**Zanclean**	3	97	470	20.64	750 km, pointy-topped
**Zanclean**	3	195	2156	9.04	250 km, flat-topped
**Zanclean**	3	132	850	15.53	500 km, flat-topped
**Zanclean**	3	97	468	20.73	750 km, flat-topped
**Messinian**	3	70	2227	3.14	250 km, pointy-topped
**Messinian**	3	47	820	5.73	500 km, pointy-topped
**Messinian**	3	44	470	9.36	750 km, pointy-topped
**Messinian**	3	67	2142	3.13	250 km, flat-topped
**Messinian**	3	52	846	6.15	500 km, flat-topped
**Messinian**	3	44	467	9.42	750 km, flat-topped
**Tortonian**	11	183	2373	7.71	250 km, pointy-topped
**Tortonian**	11	118	890	13.26	500 km, pointy-topped

**Tortonian**	11	99	520	19.04	750 km, pointy-topped
**Tortonian**	11	182	2273	8.01	250 km, flat-topped
**Tortonian**	11	122	949	12.86	500 km, flat-topped
**Tortonian**	11	97	509	19.06	750 km, flat-topped
**Serravallian**	11	151	2377	6.35	250 km, pointy-topped
**Serravallian**	11	111	889	12.49	500 km, pointy-topped
**Serravallian**	11	81	520	15.58	750 km, pointy-topped
**Serravallian**	11	157	2266	6.93	250 km, flat-topped
**Serravallian**	11	107	947	11.3	500 km, flat-topped
**Serravallian**	11	87	508	17.13	750 km, flat-topped
**Langhian**	15	73	2067	3.53	250 km, pointy-topped
**Langhian**	15	57	786	7.25	500 km, pointy-topped
**Langhian**	15	43	451	9.53	750 km, pointy-topped
**Langhian**	15	71	1938	3.66	250 km, flat-topped
**Langhian**	15	52	795	6.54	500 km, flat-topped
**Langhian**	15	47	458	10.26	750 km, flat-topped
**Burdigalian**	20	231	2414	9.57	250 km, pointy-topped
**Burdigalian**	20	146	914	15.97	500 km, pointy-topped
**Burdigalian**	20	105	544	19.3	750 km, pointy-topped
**Burdigalian**	20	227	2310	9.83	250 km, flat-topped
**Burdigalian**	20	150	961	15.61	500 km, flat-topped
**Burdigalian**	20	105	544	19.3	750 km, flat-topped
**Aquitanian**	20	50	2384	2.1	250 km, pointy-topped
**Aquitanian**	20	42	910	4.62	500 km, pointy-topped
**Aquitanian**	20	33	540	6.11	750 km, pointy-topped
**Aquitanian**	20	47	2285	2.06	250 km, flat-topped
**Aquitanian**	20	36	950	3.79	500 km, flat-topped
**Aquitanian**	20	34	541	6.28	750 km, flat-topped
**Chattian**	26	125	2062	6.06	250 km, pointy-topped
**Chattian**	26	91	785	11.59	500 km, pointy-topped
**Chattian**	26	74	459	16.12	750 km, pointy-topped
**Chattian**	26	130	1968	6.61	250 km, flat-topped
**Chattian**	26	92	814	11.3	500 km, flat-topped
**Chattian**	26	67	458	14.63	750 km, flat-topped
**Rupelian**	31	102	2383	4.28	250 km, pointy-topped
**Rupelian**	31	79	913	8.65	500 km, pointy-topped
**Rupelian**	31	59	535	11.03	750 km, pointy-topped
**Rupelian**	31	104	2319	4.48	250 km, flat-topped
**Rupelian**	31	73	950	7.68	500 km, flat-topped
**Rupelian**	31	60	531	11.3	750 km, flat-topped
**Priabonian**	36	135	2114	6.39	250 km, pointy-topped
**Priabonian**	36	95	806	11.79	500 km, pointy-topped
**Priabonian**	36	76	470	16.17	750 km, pointy-topped
**Priabonian**	36	140	2046	6.84	250 km, flat-topped

**Priabonian**	36	95	860	11.05	500 km, flat-topped
**Priabonian**	36	76	464	16.38	750 km, flat-topped
**Bartonian**	40	80	2384	3.36	250 km, pointy-topped
**Bartonian**	40	62	906	6.84	500 km, pointy-topped
**Bartonian**	40	50	520	9.62	750 km, pointy-topped
**Bartonian**	40	83	2315	3.59	250 km, flat-topped
**Bartonian**	40	59	933	6.32	500 km, flat-topped
**Bartonian**	40	46	512	8.98	750 km, flat-topped
**Lutetian**	45	97	2440	3.98	250 km, pointy-topped
**Lutetian**	45	65	873	7.45	500 km, pointy-topped
**Lutetian**	45	55	499	11.02	750 km, pointy-topped
**Lutetian**	45	89	2341	3.8	250 km, flat-topped
**Lutetian**	45	65	898	7.24	500 km, flat-topped
**Lutetian**	45	54	488	11.07	750 km, flat-topped
**Ypresian**	52	104	3073	3.38	250 km, pointy-topped
**Ypresian**	52	76	1043	7.29	500 km, pointy-topped
**Ypresian**	52	64	597	10.72	750 km, pointy-topped
**Ypresian**	52	102	2985	3.42	250 km, flat-topped
**Ypresian**	52	74	1064	6.95	500 km, flat-topped
**Ypresian**	52	65	576	11.28	750 km, flat-topped
**Thanetian**	56	67	2495	2.69	250 km, pointy-topped
**Thanetian**	56	50	878	5.69	500 km, pointy-topped
**Thanetian**	56	43	489	8.79	750 km, pointy-topped
**Thanetian**	56	67	2373	2.82	250 km, flat-topped
**Thanetian**	56	48	904	5.31	500 km, flat-topped
**Thanetian**	56	44	491	8.96	750 km, flat-topped
**Selandian**	61	31	2511	1.23	250 km, pointy-topped
**Selandian**	61	23	896	2.57	500 km, pointy-topped
**Selandian**	61	25	506	4.94	750 km, pointy-topped
**Selandian**	61	32	2410	1.33	250 km, flat-topped
**Selandian**	61	25	927	2.7	500 km, flat-topped
**Selandian**	61	22	493	4.46	750 km, flat-topped
**Danian**	66	107	2372	4.51	250 km, pointy-topped
**Danian**	66	83	852	9.74	500 km, pointy-topped
**Danian**	66	61	484	12.6	750 km, pointy-topped
**Danian**	66	102	2292	4.45	250 km, flat-topped
**Danian**	66	75	879	8.53	500 km, flat-topped
**Danian**	66	60	477	12.58	750 km, flat-topped
**Maastrichtian**	69	184	2822	6.52	250 km, pointy-topped
**Maastrichtian**	69	125	1009	12.39	500 km, pointy-topped
**Maastrichtian**	69	92	567	16.23	750 km, pointy-topped
**Maastrichtian**	69	189	2704	6.99	250 km, flat-topped
**Maastrichtian**	69	127	1033	12.29	500 km, flat-topped
**Maastrichtian**	69	88	562	15.66	750 km, flat-topped

**Campanian**	75	266	2919	9.11	250 km, pointy-topped
**Campanian**	75	160	983	16.28	500 km, pointy-topped
**Campanian**	75	121	545	22.2	750 km, pointy-topped
**Campanian**	75	266	2797	9.51	250 km, flat-topped
**Campanian**	75	155	1005	15.42	500 km, flat-topped
**Campanian**	75	115	523	21.99	750 km, flat-topped
**Santonian**	87	92	2745	3.35	250 km, pointy-topped
**Santonian**	87	69	926	7.45	500 km, pointy-topped
**Santonian**	87	52	506	10.28	750 km, pointy-topped
**Santonian**	87	97	2652	3.66	250 km, flat-topped
**Santonian**	87	66	955	6.91	500 km, flat-topped
**Santonian**	87	55	510	10.78	750 km, flat-topped
**Coniacian**	87	143	2748	5.2	250 km, pointy-topped
**Coniacian**	87	93	925	10.05	500 km, pointy-topped
**Coniacian**	87	68	507	13.41	750 km, pointy-topped
**Coniacian**	87	132	2651	4.98	250 km, flat-topped
**Coniacian**	87	82	954	8.6	500 km, flat-topped
**Coniacian**	87	76	511	14.87	750 km, flat-topped
**Turonian**	92	179	2760	6.49	250 km, pointy-topped
**Turonian**	92	122	927	13.16	500 km, pointy-topped
**Turonian**	92	91	508	17.91	750 km, pointy-topped
**Turonian**	92	179	2669	6.71	250 km, flat-topped
**Turonian**	92	118	958	12.32	500 km, flat-topped
**Turonian**	92	94	511	18.4	750 km, flat-topped
**Cenomanian**	97	229	2454	9.33	250 km, pointy-topped
**Cenomanian**	97	145	851	17.04	500 km, pointy-topped
**Cenomanian**	97	111	477	23.27	750 km, pointy-topped
**Cenomanian**	97	220	2341	9.4	250 km, flat-topped
**Cenomanian**	97	138	875	15.77	500 km, flat-topped
**Cenomanian**	97	106	477	22.22	750 km, flat-topped
**Albian**	107	251	2391	10.5	250 km, pointy-topped
**Albian**	107	157	827	18.98	500 km, pointy-topped
**Albian**	107	126	461	27.33	750 km, pointy-topped
**Albian**	107	253	2294	11.03	250 km, flat-topped
**Albian**	107	154	850	18.12	500 km, flat-topped
**Albian**	107	114	459	24.84	750 km, flat-topped
**Aptian**	116	165	2356	7	250 km, pointy-topped
**Aptian**	116	101	813	12.42	500 km, pointy-topped
**Aptian**	116	83	456	18.2	750 km, pointy-topped
**Aptian**	116	160	2346	6.82	250 km, flat-topped
**Aptian**	116	99	850	11.65	500 km, flat-topped
**Aptian**	116	78	452	17.26	750 km, flat-topped
**Barremian**	122	84	2678	3.14	250 km, pointy-topped
**Barremian**	122	59	885	6.67	500 km, pointy-topped

**Barremian**	122	56	485	11.55	750 km, pointy-topped
**Barremian**	122	86	2599	3.31	250 km, flat-topped
**Barremian**	122	66	931	7.09	500 km, flat-topped
**Barremian**	122	54	480	11.25	750 km, flat-topped
**Hauterivian**	131	81	2238	3.62	250 km, pointy-topped
**Hauterivian**	131	59	791	7.46	500 km, pointy-topped
**Hauterivian**	131	51	447	11.41	750 km, pointy-topped
**Hauterivian**	131	76	2167	3.51	250 km, flat-topped
**Hauterivian**	131	61	813	7.5	500 km, flat-topped
**Hauterivian**	131	47	427	11.01	750 km, flat-topped
**Valanginian**	136	102	2044	4.99	250 km, pointy-topped
**Valanginian**	136	72	716	10.06	500 km, pointy-topped
**Valanginian**	136	59	410	14.39	750 km, pointy-topped
**Valanginian**	136	92	1926	4.78	250 km, flat-topped
**Valanginian**	136	68	747	9.1	500 km, flat-topped
**Valanginian**	136	58	407	14.25	750 km, flat-topped
**Berriasian**	142	79	2245	3.52	250 km, pointy-topped
**Berriasian**	142	65	788	8.25	500 km, pointy-topped
**Berriasian**	142	55	432	12.73	750 km, pointy-topped
**Berriasian**	142	76	2139	3.55	250 km, flat-topped
**Berriasian**	142	62	812	7.64	500 km, flat-topped
**Berriasian**	142	54	435	12.41	750 km, flat-topped
**Tithonian**	145	117	1964	5.96	250 km, pointy-topped
**Tithonian**	145	81	702	11.54	500 km, pointy-topped
**Tithonian**	145	63	385	16.36	750 km, pointy-topped
**Tithonian**	145	117	1888	6.2	250 km, flat-topped
**Tithonian**	145	80	723	11.07	500 km, flat-topped
**Tithonian**	145	61	388	15.72	750 km, flat-topped
**Kimmeridgian**	149	99	2092	4.73	250 km, pointy-topped
**Kimmeridgian**	149	72	726	9.92	500 km, pointy-topped
**Kimmeridgian**	149	58	392	14.8	750 km, pointy-topped
**Kimmeridgian**	149	99	2028	4.88	250 km, flat-topped
**Kimmeridgian**	149	77	740	10.41	500 km, flat-topped
**Kimmeridgian**	149	60	400	15	750 km, flat-topped
**Oxfordian**	160	144	2451	5.88	250 km, pointy-topped
**Oxfordian**	160	102	791	12.9	500 km, pointy-topped
**Oxfordian**	160	79	424	18.63	750 km, pointy-topped
**Oxfordian**	160	134	2364	5.67	250 km, flat-topped
**Oxfordian**	160	101	797	12.67	500 km, flat-topped
**Oxfordian**	160	75	418	17.94	750 km, flat-topped
**Callovian**	165	136	2159	6.3	250 km, pointy-topped
**Callovian**	165	92	712	12.92	500 km, pointy-topped
**Callovian**	165	72	386	18.65	750 km, pointy-topped
**Callovian**	165	137	2040	6.72	250 km, flat-topped

**Callovian**	165	91	723	12.59	500 km, flat-topped
**Callovian**	165	73	387	18.86	750 km, flat-topped
**Bathonian**	168	117	2157	5.42	250 km, pointy-topped
**Bathonian**	168	83	715	11.61	500 km, pointy-topped
**Bathonian**	168	63	387	16.28	750 km, pointy-topped
**Bathonian**	168	108	2034	5.31	250 km, flat-topped
**Bathonian**	168	82	724	11.33	500 km, flat-topped
**Bathonian**	168	64	385	16.62	750 km, flat-topped
**Bajocian**	168	118	2160	5.46	250 km, pointy-topped
**Bajocian**	168	77	714	10.78	500 km, pointy-topped
**Bajocian**	168	62	387	16.02	750 km, pointy-topped
**Bajocian**	168	116	2039	5.69	250 km, flat-topped
**Bajocian**	168	76	720	10.56	500 km, flat-topped
**Bajocian**	168	56	386	14.51	750 km, flat-topped
**Aalenian**	172	72	2072	3.47	250 km, pointy-topped
**Aalenian**	172	46	681	6.75	500 km, pointy-topped
**Aalenian**	172	38	375	10.13	750 km, pointy-topped
**Aalenian**	172	68	1954	3.48	250 km, flat-topped
**Aalenian**	172	44	695	6.33	500 km, flat-topped
**Aalenian**	172	35	370	9.46	750 km, flat-topped
**Toarcian**	178	116	1868	6.21	250 km, pointy-topped
**Toarcian**	178	72	639	11.27	500 km, pointy-topped
**Toarcian**	178	58	354	16.38	750 km, pointy-topped
**Toarcian**	178	117	1772	6.6	250 km, flat-topped
**Toarcian**	178	75	649	11.56	500 km, flat-topped
**Toarcian**	178	57	344	16.57	750 km, flat-topped
**Pliensbachian**	187	152	1486	10.23	250 km, pointy-topped
**Pliensbachian**	187	86	539	15.96	500 km, pointy-topped
**Pliensbachian**	187	68	312	21.79	750 km, pointy-topped
**Pliensbachian**	187	146	1369	10.66	250 km, flat-topped
**Pliensbachian**	187	92	553	16.64	500 km, flat-topped
**Pliensbachian**	187	63	301	20.93	750 km, flat-topped
**Sinemurian**	196	115	1466	7.84	250 km, pointy-topped
**Sinemurian**	196	79	531	14.88	500 km, pointy-topped
**Sinemurian**	196	59	308	19.16	750 km, pointy-topped
**Sinemurian**	196	113	1377	8.21	250 km, flat-topped
**Sinemurian**	196	76	543	14	500 km, flat-topped
**Sinemurian**	196	56	298	18.79	750 km, flat-topped
**Hettangian**	201	50	1331	3.76	250 km, pointy-topped
**Hettangian**	201	36	517	6.96	500 km, pointy-topped
**Hettangian**	201	28	304	9.21	750 km, pointy-topped
**Hettangian**	201	56	1266	4.42	250 km, flat-topped
**Hettangian**	201	37	522	7.09	500 km, flat-topped
**Hettangian**	201	30	287	10.45	750 km, flat-topped

**Rhaetian**	205	66	1310	5.04	250 km, pointy-topped
**Rhaetian**	205	49	524	9.35	500 km, pointy-topped
**Rhaetian**	205	48	304	15.79	750 km, pointy-topped
**Rhaetian**	205	67	1226	5.46	250 km, flat-topped
**Rhaetian**	205	57	521	10.94	500 km, flat-topped
**Rhaetian**	205	47	292	16.1	750 km, flat-topped
**Norian**	218	231	1476	15.65	250 km, pointy-topped
**Norian**	218	144	568	25.35	500 km, pointy-topped
**Norian**	218	104	330	31.52	750 km, pointy-topped
**Norian**	218	206	1434	14.37	250 km, flat-topped
**Norian**	218	142	565	25.13	500 km, flat-topped
**Norian**	218	96	308	31.17	750 km, flat-topped
**Carnian**	232	168	1561	10.76	250 km, pointy-topped
**Carnian**	232	102	554	18.41	500 km, pointy-topped
**Carnian**	232	81	315	25.71	750 km, pointy-topped
**Carnian**	232	157	1497	10.49	250 km, flat-topped
**Carnian**	232	110	553	19.89	500 km, flat-topped
**Carnian**	232	79	301	26.25	750 km, flat-topped
**Ladinian**	240	60	893	6.72	250 km, pointy-topped
**Ladinian**	240	46	382	12.04	500 km, pointy-topped
**Ladinian**	240	37	232	15.95	750 km, pointy-topped
**Ladinian**	240	60	871	6.89	250 km, flat-topped
**Ladinian**	240	45	404	11.14	500 km, flat-topped
**Ladinian**	240	34	231	14.72	750 km, flat-topped
**Anisian**	245	119	1410	8.44	250 km, pointy-topped
**Anisian**	245	79	494	15.99	500 km, pointy-topped
**Anisian**	245	65	284	22.89	750 km, pointy-topped
**Anisian**	245	115	1398	8.23	250 km, flat-topped
**Anisian**	245	80	503	15.9	500 km, flat-topped
**Anisian**	245	61	287	21.25	750 km, flat-topped
**Olenekian**	245	107	1415	7.56	250 km, pointy-topped
**Olenekian**	245	75	496	15.12	500 km, pointy-topped
**Olenekian**	245	59	285	20.7	750 km, pointy-topped
**Olenekian**	245	104	1399	7.43	250 km, flat-topped
**Olenekian**	245	76	511	14.87	500 km, flat-topped
**Olenekian**	245	57	290	19.66	750 km, flat-topped
**Induan**	252	91	2003	4.54	250 km, pointy-topped
**Induan**	252	62	671	9.24	500 km, pointy-topped
**Induan**	252	45	357	12.61	750 km, pointy-topped
**Induan**	252	92	1997	4.61	250 km, flat-topped
**Induan**	252	61	686	8.89	500 km, flat-topped
**Induan**	252	46	363	12.67	750 km, flat-topped
**Changhsingian**	252	78	2004	3.89	250 km, pointy-topped
**Changhsingian**	252	49	671	7.3	500 km, pointy-topped

**Changhsingian**	252	36	357	10.08	750 km, pointy-topped
**Changhsingian**	252	78	1995	3.91	250 km, flat-topped
**Changhsingian**	252	53	685	7.74	500 km, flat-topped
**Changhsingian**	252	40	362	11.05	750 km, flat-topped
**Wuchiapingian**	256	127	2015	6.3	250 km, pointy-topped
**Wuchiapingian**	256	80	675	11.85	500 km, pointy-topped
**Wuchiapingian**	256	62	358	17.32	750 km, pointy-topped
**Wuchiapingian**	256	128	1998	6.41	250 km, flat-topped
**Wuchiapingian**	256	81	687	11.79	500 km, flat-topped
**Wuchiapingian**	256	65	363	17.91	750 km, flat-topped
**Capitanian**	263	130	2201	5.91	250 km, pointy-topped
**Capitanian**	263	87	719	12.1	500 km, pointy-topped
**Capitanian**	263	68	386	17.62	750 km, pointy-topped
**Capitanian**	263	130	2191	5.93	250 km, flat-topped
**Capitanian**	263	83	737	11.26	500 km, flat-topped
**Capitanian**	263	65	398	16.33	750 km, flat-topped
**Wordian**	265	149	2012	7.41	250 km, pointy-topped
**Wordian**	265	102	673	15.16	500 km, pointy-topped
**Wordian**	265	83	358	23.18	750 km, pointy-topped
**Wordian**	265	140	1998	7.01	250 km, flat-topped
**Wordian**	265	100	687	14.56	500 km, flat-topped
**Wordian**	265	76	362	20.99	750 km, flat-topped
**Roadian**	269	177	1968	8.99	250 km, pointy-topped
**Roadian**	269	108	645	16.74	500 km, pointy-topped
**Roadian**	269	86	353	24.36	750 km, pointy-topped
**Roadian**	269	163	1973	8.26	250 km, flat-topped
**Roadian**	269	110	668	16.47	500 km, flat-topped
**Roadian**	269	83	355	23.38	750 km, flat-topped
**Kungurian**	280	177	2078	8.52	250 km, pointy-topped
**Kungurian**	280	104	678	15.34	500 km, pointy-topped
**Kungurian**	280	78	358	21.79	750 km, pointy-topped
**Kungurian**	280	178	2091	8.51	250 km, flat-topped
**Kungurian**	280	107	686	15.6	500 km, flat-topped
**Kungurian**	280	76	360	21.11	750 km, flat-topped
**Artinskian**	287	152	1966	7.73	250 km, pointy-topped
**Artinskian**	287	97	648	14.97	500 km, pointy-topped
**Artinskian**	287	78	349	22.35	750 km, pointy-topped
**Artinskian**	287	147	1964	7.48	250 km, flat-topped
**Artinskian**	287	94	662	14.2	500 km, flat-topped
**Artinskian**	287	74	353	20.96	750 km, flat-topped
**Sakmarian**	293	155	1839	8.43	250 km, pointy-topped
**Sakmarian**	293	97	619	15.67	500 km, pointy-topped
**Sakmarian**	293	73	343	21.28	750 km, pointy-topped
**Sakmarian**	293	152	1870	8.13	250 km, flat-topped

**Sakmarian**	293	98	625	15.68	500 km, flat-topped
**Sakmarian**	293	78	334	23.35	750 km, flat-topped
**Asselian**	297	129	1829	7.05	250 km, pointy-topped
**Asselian**	297	85	624	13.62	500 km, pointy-topped
**Asselian**	297	68	335	20.3	750 km, pointy-topped
**Asselian**	297	129	1872	6.89	250 km, flat-topped
**Asselian**	297	86	633	13.59	500 km, flat-topped
**Asselian**	297	72	342	21.05	750 km, flat-topped
**Gzhelian**	301	58	1920	3.02	250 km, pointy-topped
**Gzhelian**	301	45	644	6.99	500 km, pointy-topped
**Gzhelian**	301	38	344	11.05	750 km, pointy-topped
**Gzhelian**	301	57	1962	2.91	250 km, flat-topped
**Gzhelian**	301	44	666	6.61	500 km, flat-topped
**Gzhelian**	301	38	355	10.7	750 km, flat-topped
**Kasimovian**	305	42	1878	2.24	250 km, pointy-topped
**Kasimovian**	305	26	621	4.19	500 km, pointy-topped
**Kasimovian**	305	22	328	6.71	750 km, pointy-topped
**Kasimovian**	305	37	1921	1.93	250 km, flat-topped
**Kasimovian**	305	26	630	4.13	500 km, flat-topped
**Kasimovian**	305	21	339	6.19	750 km, flat-topped
**Moscovian**	311	79	1858	4.25	250 km, pointy-topped
**Moscovian**	311	54	608	8.88	500 km, pointy-topped
**Moscovian**	311	45	310	14.52	750 km, pointy-topped
**Moscovian**	311	81	1866	4.34	250 km, flat-topped
**Moscovian**	311	56	616	9.09	500 km, flat-topped
**Moscovian**	311	41	330	12.42	750 km, flat-topped
**Bashkirian**	319	75	1851	4.05	250 km, pointy-topped
**Bashkirian**	319	51	590	8.64	500 km, pointy-topped
**Bashkirian**	319	43	314	13.69	750 km, pointy-topped
**Bashkirian**	319	75	1875	4	250 km, flat-topped
**Bashkirian**	319	48	604	7.95	500 km, flat-topped
**Bashkirian**	319	40	328	12.2	750 km, flat-topped
**Serpukhovian**	327	66	1798	3.67	250 km, pointy-topped
**Serpukhovian**	327	45	584	7.71	500 km, pointy-topped
**Serpukhovian**	327	36	305	11.8	750 km, pointy-topped
**Serpukhovian**	327	68	1812	3.75	250 km, flat-topped
**Serpukhovian**	327	45	594	7.58	500 km, flat-topped
**Serpukhovian**	327	35	315	11.11	750 km, flat-topped
**Visean**	339	146	2054	7.11	250 km, pointy-topped
**Visean**	339	93	655	14.2	500 km, pointy-topped
**Visean**	339	67	341	19.65	750 km, pointy-topped
**Visean**	339	147	2063	7.13	250 km, flat-topped
**Visean**	339	92	668	13.77	500 km, flat-topped
**Visean**	339	69	337	20.47	750 km, flat-topped

**Tournaisian**	354	136	2143	6.35	250 km, pointy-topped
**Tournaisian**	354	87	671	12.97	500 km, pointy-topped
**Tournaisian**	354	62	365	16.99	750 km, pointy-topped
**Tournaisian**	354	142	2169	6.55	250 km, flat-topped
**Tournaisian**	354	86	688	12.5	500 km, flat-topped
**Tournaisian**	354	61	357	17.09	750 km, flat-topped
**Famennian**	366	142	2245	6.33	250 km, pointy-topped
**Famennian**	366	87	689	12.63	500 km, pointy-topped
**Famennian**	366	66	361	18.28	750 km, pointy-topped
**Famennian**	366	150	2256	6.65	250 km, flat-topped
**Famennian**	366	84	698	12.03	500 km, flat-topped
**Famennian**	366	66	364	18.13	750 km, flat-topped
**Frasnian**	375	198	2366	8.37	250 km, pointy-topped
**Frasnian**	375	116	724	16.02	500 km, pointy-topped
**Frasnian**	375	93	370	25.14	750 km, pointy-topped
**Frasnian**	375	196	2402	8.16	250 km, flat-topped
**Frasnian**	375	119	742	16.04	500 km, flat-topped
**Frasnian**	375	91	386	23.58	750 km, flat-topped
**Givetian**	385	153	2421	6.32	250 km, pointy-topped
**Givetian**	385	114	756	15.08	500 km, pointy-topped
**Givetian**	385	81	382	21.2	750 km, pointy-topped
**Givetian**	385	155	2426	6.39	250 km, flat-topped
**Givetian**	385	105	760	13.82	500 km, flat-topped
**Givetian**	385	79	390	20.26	750 km, flat-topped
**Eifelian**	391	107	2261	4.73	250 km, pointy-topped
**Eifelian**	391	73	708	10.31	500 km, pointy-topped
**Eifelian**	391	62	366	16.94	750 km, pointy-topped
**Eifelian**	391	113	2266	4.99	250 km, flat-topped
**Eifelian**	391	77	724	10.64	500 km, flat-topped
**Eifelian**	391	60	375	16	750 km, flat-topped
**Emsian**	400	176	2210	7.96	250 km, pointy-topped
**Emsian**	400	113	694	16.28	500 km, pointy-topped
**Emsian**	400	86	368	23.37	750 km, pointy-topped
**Emsian**	400	183	2215	8.26	250 km, flat-topped
**Emsian**	400	110	693	15.87	500 km, flat-topped
**Emsian**	400	90	366	24.59	750 km, flat-topped
**Pragian**	410	70	2234	3.13	250 km, pointy-topped
**Pragian**	410	52	699	7.44	500 km, pointy-topped
**Pragian**	410	47	364	12.91	750 km, pointy-topped
**Pragian**	410	76	2222	3.42	250 km, flat-topped
**Pragian**	410	51	707	7.21	500 km, flat-topped
**Pragian**	410	47	371	12.67	750 km, flat-topped
**Lochkovian**	415	119	2395	4.97	250 km, pointy-topped
**Lochkovian**	415	89	745	11.95	500 km, pointy-topped

**Lochkovian**	415	71	385	18.44	750 km, pointy-topped
**Lochkovian**	415	119	2389	4.98	250 km, flat-topped
**Lochkovian**	415	85	752	11.3	500 km, flat-topped
**Lochkovian**	415	66	385	17.14	750 km, flat-topped
**Pridoli**	421	106	2541	4.17	250 km, pointy-topped
**Pridoli**	421	75	783	9.58	500 km, pointy-topped
**Pridoli**	421	53	403	13.15	750 km, pointy-topped
**Pridoli**	421	102	2512	4.06	250 km, flat-topped
**Pridoli**	421	72	796	9.05	500 km, flat-topped
**Pridoli**	421	58	410	14.15	750 km, flat-topped
**Ludfordian**	425	99	2522	3.93	250 km, pointy-topped
**Ludfordian**	425	72	768	9.38	500 km, pointy-topped
**Ludfordian**	425	54	400	13.5	750 km, pointy-topped
**Ludfordian**	425	104	2517	4.13	250 km, flat-topped
**Ludfordian**	425	68	772	8.81	500 km, flat-topped
**Ludfordian**	425	52	403	12.9	750 km, flat-topped
**Gorstian**	425	35	2509	1.39	250 km, pointy-topped
**Gorstian**	425	29	764	3.8	500 km, pointy-topped
**Gorstian**	425	25	399	6.27	750 km, pointy-topped
**Gorstian**	425	35	2504	1.4	250 km, flat-topped
**Gorstian**	425	31	771	4.02	500 km, flat-topped
**Gorstian**	425	24	402	5.97	750 km, flat-topped
**Homerian**	430	119	2524	4.71	250 km, pointy-topped
**Homerian**	430	92	787	11.69	500 km, pointy-topped
**Homerian**	430	68	397	17.13	750 km, pointy-topped
**Homerian**	430	112	2538	4.41	250 km, flat-topped
**Homerian**	430	92	796	11.56	500 km, flat-topped
**Homerian**	430	70	407	17.2	750 km, flat-topped
**Sheinwoodian**	430	36	2520	1.43	250 km, pointy-topped
**Sheinwoodian**	430	25	783	3.19	500 km, pointy-topped
**Sheinwoodian**	430	22	397	5.54	750 km, pointy-topped
**Sheinwoodian**	430	36	2532	1.42	250 km, flat-topped
**Sheinwoodian**	430	25	794	3.15	500 km, flat-topped
**Sheinwoodian**	430	23	407	5.65	750 km, flat-topped
**Telychian**	436	113	2224	5.08	250 km, pointy-topped
**Telychian**	436	72	723	9.96	500 km, pointy-topped
**Telychian**	436	60	371	16.17	750 km, pointy-topped
**Telychian**	436	109	2214	4.92	250 km, flat-topped
**Telychian**	436	72	722	9.97	500 km, flat-topped
**Telychian**	436	58	380	15.26	750 km, flat-topped
**Aeronian**	441	56	2043	2.74	250 km, pointy-topped
**Aeronian**	441	45	649	6.93	500 km, pointy-topped
**Aeronian**	441	36	361	9.97	750 km, pointy-topped
**Aeronian**	441	55	2033	2.71	250 km, flat-topped

**Aeronian**	441	44	670	6.57	500 km, flat-topped
**Aeronian**	441	37	350	10.57	750 km, flat-topped
**Rhuddanian**	441	74	2049	3.61	250 km, pointy-topped
**Rhuddanian**	441	54	649	8.32	500 km, pointy-topped
**Rhuddanian**	441	46	361	12.74	750 km, pointy-topped
**Rhuddanian**	441	75	2036	3.68	250 km, flat-topped
**Rhuddanian**	441	54	670	8.06	500 km, flat-topped
**Rhuddanian**	441	50	352	14.2	750 km, flat-topped
**Hirnantian**	445	78	2043	3.82	250 km, pointy-topped
**Hirnantian**	445	60	653	9.19	500 km, pointy-topped
**Hirnantian**	445	46	358	12.85	750 km, pointy-topped
**Hirnantian**	445	79	2061	3.83	250 km, flat-topped
**Hirnantian**	445	58	660	8.79	500 km, flat-topped
**Hirnantian**	445	50	362	13.81	750 km, flat-topped
**Katian**	449	273	2114	12.91	250 km, pointy-topped
**Katian**	449	159	677	23.49	500 km, pointy-topped
**Katian**	449	110	368	29.89	750 km, pointy-topped
**Katian**	449	270	2149	12.56	250 km, flat-topped
**Katian**	449	152	687	22.13	500 km, flat-topped
**Katian**	449	104	372	27.96	750 km, flat-topped
**Sandbian**	456	193	2157	8.95	250 km, pointy-topped
**Sandbian**	456	117	680	17.21	500 km, pointy-topped
**Sandbian**	456	88	371	23.72	750 km, pointy-topped
**Sandbian**	456	194	2173	8.93	250 km, flat-topped
**Sandbian**	456	120	697	17.22	500 km, flat-topped
**Sandbian**	456	87	381	22.83	750 km, flat-topped
**Darriwilian**	465	226	2207	10.24	250 km, pointy-topped
**Darriwilian**	465	128	712	17.98	500 km, pointy-topped
**Darriwilian**	465	86	370	23.24	750 km, pointy-topped
**Darriwilian**	465	223	2241	9.95	250 km, flat-topped
**Darriwilian**	465	124	716	17.32	500 km, flat-topped
**Darriwilian**	465	86	377	22.81	750 km, flat-topped
**Dapingian**	470	60	2236	2.68	250 km, pointy-topped
**Dapingian**	470	47	713	6.59	500 km, pointy-topped
**Dapingian**	470	34	374	9.09	750 km, pointy-topped
**Dapingian**	470	58	2255	2.57	250 km, flat-topped
**Dapingian**	470	43	716	6.01	500 km, flat-topped
**Dapingian**	470	34	377	9.02	750 km, flat-topped
**Floian**	475	190	2290	8.3	250 km, pointy-topped
**Floian**	475	117	719	16.27	500 km, pointy-topped
**Floian**	475	89	382	23.3	750 km, pointy-topped
**Floian**	475	193	2301	8.39	250 km, flat-topped
**Floian**	475	112	726	15.43	500 km, flat-topped
**Floian**	475	92	393	23.41	750 km, flat-topped

**Tremadocian**	482	181	2366	7.65	250 km, pointy-topped
**Tremadocian**	482	111	750	14.8	500 km, pointy-topped
**Tremadocian**	482	79	391	20.2	750 km, pointy-topped
**Tremadocian**	482	179	2366	7.57	250 km, flat-topped
**Tremadocian**	482	115	754	15.25	500 km, flat-topped
**Tremadocian**	482	84	402	20.9	750 km, flat-topped
**Stage 10**	492	89	2116	4.21	250 km, pointy-topped
**Stage 10**	492	63	647	9.74	500 km, pointy-topped
**Stage 10**	492	49	358	13.69	750 km, pointy-topped
**Stage 10**	492	87	2123	4.1	250 km, flat-topped
**Stage 10**	492	61	684	8.92	500 km, flat-topped
**Stage 10**	492	50	365	13.7	750 km, flat-topped
**Jiangshanian**	492	51	2114	2.41	250 km, pointy-topped
**Jiangshanian**	492	40	645	6.2	500 km, pointy-topped
**Jiangshanian**	492	30	358	8.38	750 km, pointy-topped
**Jiangshanian**	492	50	2120	2.36	250 km, flat-topped
**Jiangshanian**	492	41	684	5.99	500 km, flat-topped
**Jiangshanian**	492	32	364	8.79	750 km, flat-topped
**Paibian**	496	34	2048	1.66	250 km, pointy-topped
**Paibian**	496	26	649	4.01	500 km, pointy-topped
**Paibian**	496	30	353	8.5	750 km, pointy-topped
**Paibian**	496	28	2085	1.34	250 km, flat-topped
**Paibian**	496	31	665	4.66	500 km, flat-topped
**Paibian**	496	26	357	7.28	750 km, flat-topped
**Guzhangian**	496	62	2048	3.03	250 km, pointy-topped
**Guzhangian**	496	49	650	7.54	500 km, pointy-topped
**Guzhangian**	496	44	352	12.5	750 km, pointy-topped
**Guzhangian**	496	66	2084	3.17	250 km, flat-topped
**Guzhangian**	496	54	664	8.13	500 km, flat-topped
**Guzhangian**	496	39	357	10.92	750 km, flat-topped
**Drumian**	505	90	1960	4.59	250 km, pointy-topped
**Drumian**	505	64	651	9.83	500 km, pointy-topped
**Drumian**	505	53	344	15.41	750 km, pointy-topped
**Drumian**	505	86	1965	4.38	250 km, flat-topped
**Drumian**	505	63	638	9.87	500 km, flat-topped
**Drumian**	505	55	349	15.76	750 km, flat-topped
**Wuliuan**	505	17	1959	0.87	250 km, pointy-topped
**Wuliuan**	505	14	650	2.15	500 km, pointy-topped
**Wuliuan**	505	12	344	3.49	750 km, pointy-topped
**Wuliuan**	505	17	1964	0.87	250 km, flat-topped
**Wuliuan**	505	13	638	2.04	500 km, flat-topped
**Wuliuan**	505	11	348	3.16	750 km, flat-topped
**Stage 4**	510	208	1995	10.43	250 km, pointy-topped
**Stage 4**	510	125	653	19.14	500 km, pointy-topped

**Stage 4**	510	92	345	26.67	750 km, pointy-topped
**Stage 4**	510	215	2016	10.66	250 km, flat-topped
**Stage 4**	510	129	652	19.79	500 km, flat-topped
**Stage 4**	510	88	353	24.93	750 km, flat-topped
**Stage 3**	520	101	2132	4.74	250 km, pointy-topped
**Stage 3**	520	68	670	10.15	500 km, pointy-topped
**Stage 3**	520	59	366	16.12	750 km, pointy-topped
**Stage 3**	520	100	2147	4.66	250 km, flat-topped
**Stage 3**	520	72	689	10.45	500 km, flat-topped
**Stage 3**	520	54	370	14.59	750 km, flat-topped
**Stage 2**	525	49	2085	2.35	250 km, pointy-topped
**Stage 2**	525	37	663	5.58	500 km, pointy-topped
**Stage 2**	525	32	362	8.84	750 km, pointy-topped
**Stage 2**	525	52	2099	2.48	250 km, flat-topped
**Stage 2**	525	40	670	5.97	500 km, flat-topped
**Stage 2**	525	32	367	8.72	750 km, flat-topped
**Fortunian**	535	4	2015	0.2	250 km, pointy-topped
**Fortunian**	535	4	634	0.63	500 km, pointy-topped
**Fortunian**	535	3	346	0.87	750 km, pointy-topped
**Fortunian**	535	4	2014	0.2	250 km, flat-topped
**Fortunian**	535	4	658	0.61	500 km, flat-topped
**Fortunian**	535	3	349	0.86	750 km, flat-topped

Table S5.

Results for the logistic and logistic-like (Gompertz) models estimating fossil data coverage of shallow-marine regions based on collection history. Predicted completeness refers to the percentage of the observed number of grid cells containing fossil data relative to the predicted value for 2100 (see Methods for details).

Bin ID	Model	AIC	Predicted no. cells	Upper CI	Lower CI	Obser-ved no. cells with fossils	Predicted complete-ness	Grid type
**1**	Gompertz Type-3	462.33	568.46	NA	NA	188	33.07	250 km, flat-topped
**1**	Logistic Type-3	460.70	389.11	472.03	306.19	188	48.31	250 km, flat-topped
**1**	Logistic Type-3	465.39	346.58	394.12	299.05	190	54.82	250 km, pointy-topped
**1**	Logistic Type-3	403.88	283.54	366.73	200.36	130	45.85	500 km, flat-topped
**1**	Logistic Type-4	405.61	262.62	356.93	173.65	130	49.50	500 km, flat-topped
**1**	Logistic Type-5	405.83	218.57	439.18	129.47	130	59.48	500 km, flat-topped
**1**	Logistic Type-3	396.46	396.15	484.80	307.50	133	33.57	500 km, pointy-topped
**1**	Logistic Type-5	394.48	210.99	442.51	127.72	133	63.04	500 km, pointy-topped
**1**	Logistic Type-5	303.61	103.98	110.61	97.37	105	100.98	750 km, flat-topped
**1**	Logistic Type-5	316.11	109.89	117.34	102.45	112	101.92	750 km, pointy-topped
**2**	Gompertz Type-4	513.54	230.99	257.92	204.06	171	74.03	250 km, flat-topped
**2**	Gompertz Type-3	511.55	231.76	257.26	206.25	171	73.78	250 km, flat-topped
**2**	Gompertz Type-3	501.11	225.03	249.44	200.63	170	75.54	250 km, pointy-topped
**2**	Gompertz Type-4	502.37	220.71	245.94	195.48	170	77.02	250 km, pointy-topped
**2**	Gompertz Type-4	373.25	149.26	166.82	131.71	118	79.05	500 km, flat-topped
**2**	Gompertz Type-3	372.45	152.40	169.89	134.92	118	77.43	500 km, flat-topped
**2**	Gompertz Type-4	373.83	147.96	164.24	131.68	120	81.10	500 km, pointy-topped
**2**	Gompertz Type-3	373.44	151.50	167.94	135.07	120	79.21	500 km, pointy-topped
**2**	Gompertz Type-4	296.86	108.50	120.08	96.93	96	88.48	750 km, flat-topped
**2**	Gompertz Type-3	298.74	112.69	125.12	100.27	96	85.19	750 km, flat-topped
**2**	Gompertz Type-3	314.39	122.49	133.97	111.00	100	81.64	750 km, pointy-topped
**2**	Gompertz Type-4	313.62	119.21	130.34	108.07	100	83.89	750 km, pointy-topped
**3**	Logistic Type-3	80.14	69.47	113.57	34.92	30	43.19	250 km, flat-topped
**3**	Logistic Type-4	82.08	66.63	110.20	33.90	30	45.03	250 km, flat-topped
**3**	Gompertz Type-3	80.14	97.07	NA	NA	30	30.91	250 km, flat-topped
**3**	Gompertz Type-4	81.19	80.55	106.30	54.79	30	37.25	250 km, flat-topped
**3**	Gompertz Type-3	83.18	65.72	74.14	57.30	31	47.17	250 km, pointy-topped
**3**	Gompertz Type-4	84.48	60.39	71.79	48.99	31	51.33	250 km, pointy-topped
**3**	Logistic Type-3	58.01	77.76	118.56	38.50	22	28.29	500 km, flat-topped
**3**	Gompertz Type-3	59.09	74.32	NA	NA	22	29.60	500 km, flat-topped
**3**	Logistic Type-4	74.58	80.71	160.65	28.74	25	30.97	500 km, pointy-topped
**3**	Gompertz Type-4	74.70	80.46	NA	NA	25	31.07	500 km, pointy-topped
**3**	Gompertz Type-3	72.85	94.11	NA	NA	25	26.57	500 km, pointy-topped
**3**	Logistic Type-3	72.79	68.55	119.48	30.45	25	36.47	500 km, pointy-topped
**3**	Logistic Type-5	57.83	61.93	161.27	26.89	22	35.52	750 km, flat-topped

**3**	Logistic Type-3	56.28	82.78	122.01	43.69	22	26.58	750 km, flat-topped
**3**	Logistic Type-4	57.01	78.04	134.81	30.63	22	28.19	750 km, flat-topped
**3**	Logistic Type-3	61.89	62.88	110.63	26.27	21	33.40	750 km, pointy-topped
**3**	Gompertz Type-3	61.91	66.93	NA	NA	21	31.37	750 km, pointy-topped
**3**	Gompertz Type-4	63.59	64.69	NA	NA	21	32.46	750 km, pointy-topped
**4**	Logistic Type-5	272.75	99.88	107.00	92.77	99	99.12	250 km, flat-topped
**4**	Logistic Type-3	281.59	149.84	172.45	127.22	99	66.07	250 km, pointy-topped
**4**	Logistic Type-4	283.47	138.88	157.17	120.59	99	71.28	250 km, pointy-topped
**4**	Logistic Type-3	199.86	91.46	104.49	78.43	68	74.35	500 km, flat-topped
**4**	Logistic Type-4	201.52	89.75	103.90	75.60	68	75.77	500 km, flat-topped
**4**	Logistic Type-3	213.43	108.76	122.93	94.59	78	71.72	500 km, pointy-topped
**4**	Logistic Type-4	215.11	106.80	122.41	91.18	78	73.03	500 km, pointy-topped
**4**	Logistic Type-3	174.88	84.71	101.36	68.07	60	70.83	750 km, flat-topped
**4**	Logistic Type-4	176.54	82.58	99.57	65.64	60	72.66	750 km, flat-topped
**4**	Gompertz Type-4	175.92	100.04	119.52	80.57	60	59.97	750 km, flat-topped
**4**	Gompertz Type-3	186.28	108.69	125.03	92.35	61	56.12	750 km, pointy-topped
**4**	Logistic Type-4	186.76	83.19	98.70	67.67	61	73.33	750 km, pointy-topped
**4**	Logistic Type-3	185.07	84.78	99.41	70.15	61	71.95	750 km, pointy-topped
**4**	Gompertz Type-4	184.82	100.92	118.48	83.37	61	60.44	750 km, pointy-topped
**5**	Gompertz Type-4	516.04	326.94	339.46	314.43	194	59.34	250 km, flat-topped
**5**	Gompertz Type-3	514.32	330.22	342.49	317.95	194	58.75	250 km, flat-topped
**5**	Gompertz Type-4	546.84	317.82	332.42	303.23	200	62.93	250 km, pointy-topped
**5**	Gompertz Type-3	545.05	318.99	333.38	304.60	200	62.70	250 km, pointy-topped
**5**	Gompertz Type-3	409.44	198.39	211.38	185.41	132	66.53	500 km, flat-topped
**5**	Gompertz Type-4	410.14	192.60	207.21	177.99	132	68.54	500 km, flat-topped
**5**	Gompertz Type-4	384.92	187.03	200.87	173.19	133	71.11	500 km, pointy-topped
**5**	Gompertz Type-4	328.39	150.06	160.76	139.36	97	64.64	750 km, flat-topped
**5**	Gompertz Type-3	326.92	154.36	164.09	144.64	97	62.84	750 km, flat-topped
**5**	Gompertz Type-4	294.85	138.19	148.41	127.97	97	70.19	750 km, pointy-topped
**6**	Logistic Type-5	186.65	73.41	82.24	65.33	67	91.26	250 km, flat-topped
**6**	Logistic Type-4	186.89	80.27	87.72	72.81	67	83.47	250 km, flat-topped
**6**	Logistic Type-4	212.62	89.44	100.40	78.48	70	78.27	250 km, pointy-topped
**6**	Logistic Type-5	214.59	86.82	102.47	72.73	70	80.62	250 km, pointy-topped
**6**	Logistic Type-5	145.11	60.69	70.88	51.87	52	85.68	500 km, flat-topped
**6**	Logistic Type-4	143.33	62.92	69.67	56.18	52	82.64	500 km, flat-topped
**6**	Logistic Type-5	136.08	48.12	54.21	42.87	47	97.68	500 km, pointy-topped
**6**	Logistic Type-4	137.48	54.71	61.05	48.38	47	85.90	500 km, pointy-topped
**6**	Logistic Type-4	130.79	56.96	69.55	44.96	44	77.24	750 km, flat-topped
**6**	Logistic Type-3	129.05	58.95	68.87	49.04	44	74.63	750 km, flat-topped
**6**	Logistic Type-4	128.93	60.24	73.09	47.73	44	73.04	750 km, pointy-topped
**6**	Logistic Type-3	127.43	63.90	75.05	52.76	44	68.85	750 km, pointy-topped
**6**	Gompertz Type-4	129.20	79.07	90.52	67.61	44	55.65	750 km, pointy-topped
**7**	Gompertz Type-3	521.49	306.39	318.42	294.36	181	59.08	250 km, flat-topped
**7**	Logistic Type-5	521.25	251.66	285.32	218.15	181	71.92	250 km, flat-topped

**7**	Logistic Type-4	519.81	257.47	275.49	239.45	181	70.30	250 km, flat-topped
**7**	Gompertz Type-3	510.41	296.72	308.94	284.50	181	61.00	250 km, pointy-topped
**7**	Gompertz Type-4	512.37	295.57	308.06	283.09	181	61.24	250 km, pointy-topped
**7**	Logistic Type-5	356.05	165.28	183.04	147.52	122	73.81	500 km, flat-topped
**7**	Gompertz Type-3	356.05	196.98	204.70	189.27	122	61.93	500 km, flat-topped
**7**	Logistic Type-4	354.15	168.10	179.37	156.84	122	72.57	500 km, flat-topped
**7**	Gompertz Type-4	340.75	164.69	173.33	156.05	118	71.65	500 km, pointy-topped
**7**	Gompertz Type-3	342.32	169.49	177.60	161.38	118	69.62	500 km, pointy-topped
**7**	Logistic Type-4	342.69	145.57	153.97	137.16	118	81.06	500 km, pointy-topped
**7**	Logistic Type-4	299.95	122.74	132.24	113.24	97	79.03	750 km, flat-topped
**7**	Gompertz Type-3	300.75	141.27	149.94	132.59	97	68.66	750 km, flat-topped
**7**	Gompertz Type-4	301.71	138.63	148.26	128.99	97	69.97	750 km, flat-topped
**7**	Logistic Type-5	270.31	102.10	109.82	94.94	98	95.98	750 km, pointy-topped
**8**	Gompertz Type-3	466.42	372.18	NA	NA	156	41.92	250 km, flat-topped
**8**	Gompertz Type-3	442.73	287.68	298.83	276.52	150	52.14	250 km, pointy-topped
**8**	Gompertz Type-4	444.46	283.42	296.02	270.83	150	52.92	250 km, pointy-topped
**8**	Gompertz Type-3	351.04	182.03	193.74	170.32	106	58.23	500 km, flat-topped
**8**	Gompertz Type-4	352.66	176.12	189.43	162.82	106	60.19	500 km, flat-topped
**8**	Gompertz Type-3	360.76	224.50	234.83	214.16	110	49.00	500 km, pointy-topped
**8**	Gompertz Type-4	362.62	219.42	232.06	206.79	110	50.13	500 km, pointy-topped
**8**	Gompertz Type-3	271.17	190.45	NA	NA	86	45.16	750 km, flat-topped
**8**	Logistic Type-3	247.96	107.74	117.51	97.97	80	74.26	750 km, pointy-topped
**8**	Gompertz Type-4	248.41	126.72	136.65	116.78	80	63.13	750 km, pointy-topped
**8**	Logistic Type-4	249.82	108.82	120.62	97.03	80	73.51	750 km, pointy-topped
**9**	Gompertz Type-3	205.67	117.89	127.13	108.65	71	60.23	250 km, flat-topped
**9**	Gompertz Type-4	207.66	119.31	128.38	110.25	71	59.51	250 km, flat-topped
**9**	Gompertz Type-3	234.00	120.44	131.28	109.60	73	60.61	250 km, pointy-topped
**9**	Gompertz Type-4	235.98	121.86	132.45	111.27	73	59.90	250 km, pointy-topped
**9**	Gompertz Type-3	159.43	72.71	84.69	60.74	52	71.51	500 km, flat-topped
**9**	Gompertz Type-4	161.17	70.14	83.39	56.89	52	74.14	500 km, flat-topped
**9**	Gompertz Type-3	179.24	92.99	101.90	84.09	57	61.29	500 km, pointy-topped
**9**	Gompertz Type-4	181.07	96.13	104.69	87.57	57	59.29	500 km, pointy-topped
**9**	Gompertz Type-4	134.88	63.63	71.72	55.54	47	73.87	750 km, flat-topped
**9**	Gompertz Type-3	133.66	65.76	73.35	58.17	47	71.47	750 km, flat-topped
**9**	Gompertz Type-3	144.44	53.53	63.78	43.28	43	80.33	750 km, pointy-topped
**9**	Gompertz Type-4	145.75	51.25	61.85	40.66	43	83.91	750 km, pointy-topped
**10**	Gompertz Type-4	612.03	383.99	398.29	369.69	227	59.12	250 km, flat-topped
**10**	Gompertz Type-4	587.11	442.77	459.40	426.13	231	52.17	250 km, pointy-topped
**10**	Gompertz Type-3	433.85	214.72	226.76	202.67	150	69.86	500 km, flat-topped
**10**	Gompertz Type-4	434.82	219.07	231.78	206.36	150	68.47	500 km, flat-topped
**10**	Gompertz Type-4	423.99	207.17	218.33	196.02	146	70.47	500 km, pointy-topped
**10**	Gompertz Type-3	302.15	138.95	146.57	131.34	105	75.56	750 km, flat-topped
**10**	Gompertz Type-4	302.95	141.81	150.54	133.07	105	74.04	750 km, flat-topped
**10**	Gompertz Type-4	318.08	145.20	154.49	135.90	105	72.32	750 km, pointy-topped

**10**	Gompertz Type-3	319.66	139.40	147.82	130.98	105	75.32	750 km, pointy-topped
**11**	Logistic Type-5	149.70	124.12	250.09	64.18	46	37.06	250 km, flat-topped
**11**	Logistic Type-4	149.97	137.26	191.00	83.53	46	33.51	250 km, flat-topped
**11**	Logistic Type-3	150.41	144.30	188.32	100.28	46	31.88	250 km, flat-topped
**11**	Logistic Type-4	154.40	143.15	195.77	90.54	49	34.23	250 km, pointy-topped
**11**	Logistic Type-5	152.59	105.87	192.92	58.79	49	46.28	250 km, pointy-topped
**11**	Logistic Type-3	111.72	106.01	213.94	31.84	35	33.02	500 km, flat-topped
**11**	Logistic Type-3	132.34	129.95	NA	NA	41	31.55	500 km, pointy-topped
**11**	Logistic Type-3	115.64	79.41	99.40	59.43	34	42.81	750 km, flat-topped
**11**	Gompertz Type-3	117.35	79.28	82.25	76.32	34	42.88	750 km, flat-topped
**11**	Logistic Type-4	117.17	82.87	NA	NA	34	41.03	750 km, flat-topped
**11**	Logistic Type-4	108.05	83.57	NA	NA	33	39.49	750 km, pointy-topped
**11**	Logistic Type-3	106.67	76.94	97.50	56.38	33	42.89	750 km, pointy-topped
**11**	Gompertz Type-3	107.77	80.16	82.79	77.53	33	41.17	750 km, pointy-topped
**12**	Gompertz Type-4	360.62	211.89	222.60	201.19	130	61.35	250 km, flat-topped
**12**	Gompertz Type-4	391.41	229.36	240.50	218.23	125	54.50	250 km, pointy-topped
**12**	Logistic Type-4	284.33	205.55	NA	NA	92	44.76	500 km, flat-topped
**12**	Gompertz Type-4	286.01	201.95	205.70	198.21	92	45.56	500 km, flat-topped
**12**	Gompertz Type-4	330.03	190.02	196.77	183.27	91	47.89	500 km, pointy-topped
**12**	Logistic Type-4	328.11	192.80	196.37	189.23	91	47.20	500 km, pointy-topped
**12**	Logistic Type-4	200.84	121.48	131.64	111.32	67	55.15	750 km, flat-topped
**12**	Gompertz Type-4	201.62	120.56	128.95	112.18	67	55.57	750 km, flat-topped
**12**	Logistic Type-4	235.31	164.57	NA	NA	74	44.97	750 km, pointy-topped
**12**	Logistic Type-5	235.53	179.66	192.50	166.82	74	41.19	750 km, pointy-topped
**13**	Gompertz Type-3	322.07	153.60	168.88	138.33	104	67.71	250 km, flat-topped
**13**	Gompertz Type-4	324.01	153.66	169.54	137.78	104	67.68	250 km, flat-topped
**13**	Gompertz Type-4	328.46	175.38	187.30	163.46	102	58.16	250 km, pointy-topped
**13**	Gompertz Type-3	326.88	173.61	185.78	161.43	102	58.75	250 km, pointy-topped
**13**	Gompertz Type-4	263.11	102.76	115.28	90.24	73	71.04	500 km, flat-topped
**13**	Gompertz Type-3	261.20	103.16	114.76	91.56	73	70.76	500 km, flat-topped
**13**	Gompertz Type-4	240.54	118.27	129.33	107.22	79	66.79	500 km, pointy-topped
**13**	Gompertz Type-3	239.40	120.80	131.35	110.25	79	65.40	500 km, pointy-topped
**13**	Gompertz Type-4	232.55	81.56	92.87	70.25	60	73.56	750 km, flat-topped
**13**	Gompertz Type-3	231.41	83.71	94.89	72.53	60	71.68	750 km, flat-topped
**13**	Gompertz Type-4	197.85	74.26	83.98	64.53	59	79.45	750 km, pointy-topped
**13**	Gompertz Type-3	197.49	76.76	86.62	66.91	59	76.86	750 km, pointy-topped
**14**	Gompertz Type-4	344.69	182.52	191.33	173.70	140	76.71	250 km, flat-topped
**14**	Gompertz Type-4	349.57	169.12	176.47	161.77	135	79.83	250 km, pointy-topped
**14**	Gompertz Type-4	283.88	120.07	127.56	112.57	95	79.12	500 km, flat-topped
**14**	Gompertz Type-4	244.72	126.96	133.14	120.77	95	74.83	500 km, pointy-topped
**14**	Gompertz Type-4	208.43	99.86	105.96	93.76	76	76.11	750 km, flat-topped
**14**	Gompertz Type-4	212.40	92.62	98.39	86.84	76	82.06	750 km, pointy-topped
**15**	Gompertz Type-3	237.77	94.98	100.14	89.82	83	87.39	250 km, flat-topped
**15**	Gompertz Type-4	239.77	94.90	100.43	89.38	83	87.46	250 km, flat-topped

**15**	Gompertz Type-3	213.02	92.95	97.53	88.36	80	86.07	250 km, pointy-topped
**15**	Gompertz Type-4	215.00	92.83	97.76	87.90	80	86.18	250 km, pointy-topped
**15**	Gompertz Type-4	204.80	66.72	72.94	60.50	59	88.43	500 km, flat-topped
**15**	Gompertz Type-3	202.95	67.08	73.00	61.17	59	87.95	500 km, flat-topped
**15**	Gompertz Type-4	184.18	72.46	77.62	67.29	62	85.57	500 km, pointy-topped
**15**	Gompertz Type-3	182.18	72.50	77.28	67.71	62	85.52	500 km, pointy-topped
**15**	Gompertz Type-4	135.15	54.73	59.97	49.49	46	84.05	750 km, flat-topped
**15**	Gompertz Type-3	133.49	54.21	58.96	49.47	46	84.85	750 km, flat-topped
**15**	Gompertz Type-3	159.31	54.60	60.18	49.02	50	91.57	750 km, pointy-topped
**15**	Gompertz Type-4	161.17	54.90	60.86	48.95	50	91.07	750 km, pointy-topped
**16**	Gompertz Type-4	264.54	111.72	121.14	102.30	88	78.77	250 km, flat-topped
**16**	Logistic Type-5	264.74	99.96	109.15	90.77	88	88.04	250 km, flat-topped
**16**	Logistic Type-4	264.87	94.96	101.70	88.23	88	92.67	250 km, flat-topped
**16**	Logistic Type-3	266.17	97.51	104.20	90.82	88	90.25	250 km, flat-topped
**16**	Gompertz Type-4	268.94	129.06	139.44	118.67	96	74.39	250 km, pointy-topped
**16**	Logistic Type-4	269.39	109.39	117.56	101.21	96	87.76	250 km, pointy-topped
**16**	Logistic Type-5	269.41	115.49	126.61	104.37	96	83.12	250 km, pointy-topped
**16**	Logistic Type-3	267.72	110.43	117.83	103.02	96	86.93	250 km, pointy-topped
**16**	Logistic Type-4	167.89	74.67	79.88	69.46	64	85.71	500 km, flat-topped
**16**	Logistic Type-3	166.34	75.55	80.30	70.79	64	84.72	500 km, flat-topped
**16**	Gompertz Type-4	179.16	86.82	93.82	79.82	64	73.72	500 km, pointy-topped
**16**	Gompertz Type-3	180.77	90.25	96.98	83.52	64	70.91	500 km, pointy-topped
**16**	Logistic Type-5	133.17	54.62	59.08	50.29	53	97.03	750 km, flat-topped
**16**	Logistic Type-4	133.03	57.12	60.58	53.65	53	92.79	750 km, flat-topped
**16**	Logistic Type-4	141.74	59.21	63.12	55.29	54	91.21	750 km, pointy-topped
**16**	Logistic Type-3	141.41	60.21	63.95	56.47	54	89.68	750 km, pointy-topped
**17**	Gompertz Type-4	302.48	136.36	146.78	125.93	102	74.80	250 km, flat-topped
**17**	Gompertz Type-3	300.52	135.59	145.15	126.04	102	75.22	250 km, flat-topped
**17**	Gompertz Type-4	316.61	141.12	151.93	130.31	104	73.70	250 km, pointy-topped
**17**	Gompertz Type-4	161.86	96.68	100.72	92.65	74	76.54	500 km, flat-topped
**17**	Gompertz Type-3	162.77	97.92	101.83	94.01	74	75.57	500 km, flat-topped
**17**	Gompertz Type-4	194.90	97.10	102.58	91.63	76	78.27	500 km, pointy-topped
**17**	Gompertz Type-3	195.34	98.74	103.99	93.49	76	76.97	500 km, pointy-topped
**17**	Logistic Type-5	126.13	79.88	83.79	75.97	65	81.37	750 km, flat-topped
**17**	Gompertz Type-4	124.44	88.01	91.53	84.48	65	73.86	750 km, flat-topped
**17**	Logistic Type-3	159.33	70.25	73.86	66.64	64	91.10	750 km, pointy-topped
**17**	Logistic Type-4	161.32	70.31	74.43	66.20	64	91.02	750 km, pointy-topped
**18**	Gompertz Type-3	199.54	156.97	175.44	138.50	66	42.05	250 km, flat-topped
**18**	Gompertz Type-4	201.46	142.04	154.87	129.21	66	46.47	250 km, flat-topped
**18**	Gompertz Type-4	203.66	160.33	NA	NA	66	41.16	250 km, pointy-topped
**18**	Gompertz Type-3	201.80	156.99	169.30	144.67	66	42.04	250 km, pointy-topped
**18**	Gompertz Type-3	142.97	122.82	124.70	120.94	47	38.27	500 km, flat-topped
**18**	Gompertz Type-4	155.38	105.63	NA	NA	49	46.39	500 km, pointy-topped
**18**	Gompertz Type-3	153.41	103.66	110.70	96.62	49	47.27	500 km, pointy-topped

**18**	Gompertz Type-4	136.09	105.46	108.24	102.67	43	40.78	750 km, flat-topped
**18**	Gompertz Type-3	134.17	104.81	NA	NA	43	41.03	750 km, flat-topped
**18**	Gompertz Type-3	120.49	108.29	109.90	106.68	42	38.78	750 km, pointy-topped
**18**	Logistic Type-4	122.40	104.00	130.32	77.68	42	40.39	750 km, pointy-topped
**19**	Gompertz Type-4	69.30	59.76	66.22	53.30	32	53.55	250 km, flat-topped
**19**	Gompertz Type-3	68.54	55.44	62.14	48.74	32	57.72	250 km, flat-topped
**19**	Logistic Type-4	69.19	58.57	71.69	45.44	32	54.64	250 km, flat-topped
**19**	Gompertz Type-3	63.56	73.42	82.29	64.55	31	42.22	250 km, pointy-topped
**19**	Logistic Type-4	64.09	71.24	87.96	54.51	31	43.52	250 km, pointy-topped
**19**	Gompertz Type-4	64.10	79.26	81.03	77.48	31	39.11	250 km, pointy-topped
**19**	Logistic Type-5	67.95	60.73	73.31	48.15	25	41.17	500 km, flat-topped
**19**	Gompertz Type-4	67.41	53.95	56.50	51.39	25	46.34	500 km, flat-topped
**19**	Gompertz Type-3	66.75	54.36	NA	NA	25	45.99	500 km, flat-topped
**19**	Logistic Type-4	66.55	56.24	NA	NA	25	44.45	500 km, flat-topped
**19**	Gompertz Type-3	55.91	33.47	42.73	24.22	23	68.71	500 km, pointy-topped
**19**	Gompertz Type-4	56.82	39.74	50.73	28.76	23	57.87	500 km, pointy-topped
**19**	Linear	57.37	35.44	38.73	32.14	23	64.90	500 km, pointy-topped
**19**	Logistic Type-4	56.77	39.89	53.03	26.74	23	57.66	500 km, pointy-topped
**19**	Gompertz Type-3	58.02	35.73	44.91	26.54	22	61.58	750 km, flat-topped
**19**	Logistic Type-4	58.45	41.11	NA	NA	22	53.51	750 km, flat-topped
**19**	Gompertz Type-4	58.64	43.12	45.00	41.25	22	51.02	750 km, flat-topped
**19**	Linear	59.67	33.52	37.06	29.98	22	65.64	750 km, flat-topped
**19**	Gompertz Type-3	66.19	44.45	52.63	36.27	25	56.24	750 km, pointy-topped
**19**	Logistic Type-4	66.05	51.91	NA	NA	25	48.16	750 km, pointy-topped
**19**	Logistic Type-5	68.04	56.05	72.38	39.72	25	44.60	750 km, pointy-topped
**19**	Gompertz Type-4	66.43	51.12	54.32	47.91	25	48.91	750 km, pointy-topped
**20**	Logistic Type-5	253.16	109.15	118.55	100.15	102	93.45	250 km, flat-topped
**20**	Logistic Type-5	255.87	113.36	122.22	104.76	106	93.51	250 km, pointy-topped
**20**	Logistic Type-3	192.88	90.17	97.37	82.96	74	82.07	500 km, flat-topped
**20**	Logistic Type-5	193.81	81.13	90.62	72.32	74	91.21	500 km, flat-topped
**20**	Logistic Type-4	194.79	89.46	98.14	80.78	74	82.72	500 km, flat-topped
**20**	Logistic Type-5	208.58	89.11	98.27	80.66	82	92.03	500 km, pointy-topped
**20**	Logistic Type-3	210.13	102.56	110.01	95.12	82	79.95	500 km, pointy-topped
**20**	Logistic Type-5	143.05	61.78	65.41	58.15	61	98.74	750 km, flat-topped
**20**	Logistic Type-5	149.75	64.33	70.51	59.02	61	94.82	750 km, pointy-topped
**21**	Logistic Type-5	399.17	218.42	236.22	200.86	188	86.07	250 km, flat-topped
**21**	Logistic Type-3	400.66	241.22	251.47	230.97	188	77.94	250 km, flat-topped
**21**	Logistic Type-4	399.88	236.95	247.84	226.06	188	79.34	250 km, flat-topped
**21**	Logistic Type-5	381.34	193.77	203.27	184.35	183	94.44	250 km, pointy-topped
**21**	Logistic Type-5	279.56	133.69	139.83	127.57	126	94.25	500 km, flat-topped
**21**	Logistic Type-5	310.78	139.40	150.14	128.90	124	88.95	500 km, pointy-topped
**21**	Logistic Type-5	203.83	89.60	93.47	85.74	88	98.21	750 km, flat-topped
**21**	Logistic Type-4	216.40	110.09	115.00	105.18	92	83.57	750 km, pointy-topped
**21**	Logistic Type-5	217.24	106.25	113.66	98.85	92	86.59	750 km, pointy-topped

**22**	Logistic Type-5	570.25	304.24	327.67	281.03	266	87.43	250 km, flat-topped
**22**	Logistic Type-5	584.10	298.57	322.88	275.06	266	89.09	250 km, pointy-topped
**22**	Logistic Type-5	402.50	231.88	269.83	194.10	154	66.42	500 km, flat-topped
**22**	Logistic Type-3	401.09	239.17	258.19	220.16	154	64.39	500 km, flat-topped
**22**	Logistic Type-4	402.59	243.81	271.84	215.79	154	63.16	500 km, flat-topped
**22**	Logistic Type-5	399.91	236.18	268.48	203.88	160	67.74	500 km, pointy-topped
**22**	Logistic Type-3	399.20	240.35	258.04	222.65	160	66.57	500 km, pointy-topped
**22**	Logistic Type-4	400.25	255.68	285.14	226.21	160	62.58	500 km, pointy-topped
**22**	Logistic Type-4	329.55	191.07	217.27	164.88	116	60.71	750 km, flat-topped
**22**	Logistic Type-3	328.28	182.76	200.24	165.28	116	63.47	750 km, flat-topped
**22**	Logistic Type-5	329.19	170.78	198.30	143.40	116	67.92	750 km, flat-topped
**22**	Logistic Type-3	318.65	206.74	229.21	184.28	121	58.53	750 km, pointy-topped
**22**	Logistic Type-4	319.95	220.66	253.27	188.04	121	54.84	750 km, pointy-topped
**22**	Logistic Type-5	318.97	189.35	229.53	150.56	121	63.90	750 km, pointy-topped
**23**	Logistic Type-3	221.46	119.61	126.88	112.33	97	81.10	250 km, flat-topped
**23**	Logistic Type-4	223.10	121.11	130.38	111.84	97	80.09	250 km, flat-topped
**23**	Logistic Type-5	210.74	93.09	99.06	87.30	92	98.83	250 km, pointy-topped
**23**	Logistic Type-3	153.04	88.59	95.93	81.24	66	74.50	500 km, flat-topped
**23**	Logistic Type-5	151.37	72.83	83.12	64.86	66	90.63	500 km, flat-topped
**23**	Logistic Type-5	148.77	69.31	72.94	65.73	69	99.56	500 km, pointy-topped
**23**	Logistic Type-5	122.13	55.80	58.94	52.75	55	98.56	750 km, flat-topped
**23**	Logistic Type-3	125.67	78.29	87.51	69.06	52	66.42	750 km, pointy-topped
**23**	Logistic Type-5	124.46	77.58	100.11	59.13	52	67.03	750 km, pointy-topped
**23**	Logistic Type-4	125.34	86.99	102.83	71.16	52	59.77	750 km, pointy-topped
**24**	Logistic Type-5	309.38	134.56	142.53	126.73	130	96.61	250 km, flat-topped
**24**	Logistic Type-5	316.65	147.31	155.24	139.40	141	95.72	250 km, pointy-topped
**24**	Logistic Type-5	219.80	81.69	86.99	76.48	81	99.15	500 km, flat-topped
**24**	Logistic Type-5	226.91	96.98	104.34	89.86	91	93.83	500 km, pointy-topped
**24**	Logistic Type-5	178.85	74.72	78.80	70.73	74	99.04	750 km, flat-topped
**24**	Logistic Type-5	175.90	66.92	72.08	61.95	67	100.12	750 km, pointy-topped
**25**	Logistic Type-5	412.07	184.49	192.50	176.51	180	97.57	250 km, flat-topped
**25**	Logistic Type-5	427.22	185.14	193.82	176.47	179	96.68	250 km, pointy-topped
**25**	Logistic Type-5	274.64	118.47	122.93	114.01	118	99.61	500 km, flat-topped
**25**	Logistic Type-5	288.48	122.93	127.77	118.09	122	99.24	500 km, pointy-topped
**25**	Logistic Type-5	239.37	94.65	99.31	90.00	94	99.31	750 km, flat-topped
**25**	Logistic Type-5	225.28	91.52	95.07	87.97	91	99.43	750 km, pointy-topped
**26**	Logistic Type-5	482.30	219.42	228.87	209.97	217	98.90	250 km, flat-topped
**26**	Logistic Type-5	476.32	232.04	241.19	222.88	226	97.40	250 km, pointy-topped
**26**	Logistic Type-5	341.60	133.95	139.43	128.48	136	101.53	500 km, flat-topped
**26**	Logistic Type-5	342.74	143.29	150.97	135.66	142	99.10	500 km, pointy-topped
**26**	Logistic Type-5	266.48	104.97	110.02	99.92	104	99.07	750 km, flat-topped
**26**	Logistic Type-5	279.44	108.93	113.85	104.01	109	100.06	750 km, pointy-topped
**27**	Logistic Type-5	486.18	268.98	280.81	257.15	252	93.69	250 km, flat-topped
**27**	Logistic Type-5	480.48	262.93	273.60	252.26	249	94.70	250 km, pointy-topped

**27**	Logistic Type-5	373.63	154.33	160.80	147.87	153	99.14	500 km, flat-topped
**27**	Logistic Type-5	351.52	160.49	166.16	154.81	156	97.20	500 km, pointy-topped
**27**	Logistic Type-5	317.25	113.27	118.89	107.64	113	99.77	750 km, flat-topped
**27**	Logistic Type-5	341.42	126.33	132.29	120.38	125	98.94	750 km, pointy-topped
**28**	Logistic Type-5	320.75	168.04	176.54	159.54	160	95.22	250 km, flat-topped
**28**	Logistic Type-5	376.37	170.95	179.37	162.53	165	96.52	250 km, pointy-topped
**28**	Logistic Type-5	219.02	102.38	107.55	97.21	99	96.70	500 km, flat-topped
**28**	Logistic Type-5	238.99	106.70	114.12	99.33	101	94.66	500 km, pointy-topped
**28**	Logistic Type-3	176.41	110.06	120.14	99.98	78	70.87	750 km, flat-topped
**28**	Gompertz Type-4	176.52	146.34	160.03	132.66	78	53.30	750 km, flat-topped
**28**	Logistic Type-4	177.47	107.20	118.28	96.13	78	72.76	750 km, flat-topped
**28**	Logistic Type-5	197.86	87.83	93.80	81.96	83	94.50	750 km, pointy-topped
**29**	Gompertz Type-4	236.82	90.36	99.24	81.48	84	92.96	250 km, flat-topped
**29**	Gompertz Type-3	237.26	92.16	101.30	83.03	84	91.14	250 km, flat-topped
**29**	Gompertz Type-3	223.76	92.56	102.55	82.57	82	88.59	250 km, pointy-topped
**29**	Gompertz Type-4	224.38	91.11	100.95	81.27	82	90.00	250 km, pointy-topped
**29**	Gompertz Type-4	193.33	74.11	83.75	64.47	65	87.71	500 km, flat-topped
**29**	Gompertz Type-3	192.19	75.06	84.79	65.33	65	86.60	500 km, flat-topped
**29**	Gompertz Type-4	176.23	64.03	71.02	57.03	58	90.59	500 km, pointy-topped
**29**	Gompertz Type-4	150.06	56.50	61.45	51.54	53	93.81	750 km, flat-topped
**29**	Gompertz Type-4	158.25	62.41	67.97	56.84	56	89.73	750 km, pointy-topped
**30**	Gompertz Type-4	186.55	90.53	98.13	82.92	76	83.95	250 km, flat-topped
**30**	Logistic Type-5	188.50	86.85	94.17	79.54	76	87.50	250 km, flat-topped
**30**	Logistic Type-5	193.37	84.57	92.29	76.98	80	94.59	250 km, pointy-topped
**30**	Logistic Type-4	192.10	82.57	88.04	77.09	80	96.89	250 km, pointy-topped
**30**	Logistic Type-4	144.59	60.94	65.71	56.17	60	98.46	500 km, flat-topped
**30**	Logistic Type-5	145.57	63.24	69.84	56.79	60	94.88	500 km, flat-topped
**30**	Gompertz Type-4	146.46	67.74	74.47	61.02	60	88.57	500 km, flat-topped
**30**	Logistic Type-5	133.22	59.53	65.73	53.67	58	97.43	500 km, pointy-topped
**30**	Logistic Type-4	131.28	59.71	64.22	55.21	58	97.13	500 km, pointy-topped
**30**	Logistic Type-5	120.47	45.98	50.76	41.25	46	100.05	750 km, flat-topped
**30**	Logistic Type-4	119.48	46.76	51.26	42.27	46	98.37	750 km, flat-topped
**30**	Logistic Type-4	105.84	51.09	54.24	47.94	50	97.87	750 km, pointy-topped
**30**	Logistic Type-5	107.47	50.68	54.36	47.02	50	98.66	750 km, pointy-topped
**31**	Logistic Type-5	216.42	97.20	104.81	89.71	93	95.68	250 km, flat-topped
**31**	Logistic Type-5	236.90	104.36	110.61	98.12	102	97.74	250 km, pointy-topped
**31**	Logistic Type-5	153.07	72.16	78.21	66.28	68	94.24	500 km, flat-topped
**31**	Logistic Type-4	153.28	74.10	78.96	69.25	68	91.76	500 km, flat-topped
**31**	Logistic Type-5	180.08	76.07	81.42	70.75	73	95.97	500 km, pointy-topped
**31**	Logistic Type-5	136.38	60.41	64.17	56.66	59	97.67	750 km, flat-topped
**31**	Logistic Type-5	150.91	63.49	70.32	57.06	59	92.93	750 km, pointy-topped
**31**	Logistic Type-4	152.20	67.98	74.44	61.53	59	86.78	750 km, pointy-topped
**32**	Logistic Type-4	176.56	80.21	85.51	74.90	76	94.76	250 km, flat-topped
**32**	Logistic Type-5	178.33	81.13	88.41	73.98	76	93.67	250 km, flat-topped

**32**	Logistic Type-4	180.24	83.98	89.38	78.59	79	94.07	250 km, pointy-topped
**32**	Gompertz Type-4	137.82	69.58	75.33	63.82	61	87.67	500 km, flat-topped
**32**	Gompertz Type-4	147.96	72.84	79.00	66.68	65	89.24	500 km, pointy-topped
**32**	Logistic Type-5	148.59	69.96	76.49	63.50	65	92.91	500 km, pointy-topped
**32**	Logistic Type-4	148.70	67.23	72.15	62.31	65	96.68	500 km, pointy-topped
**32**	Gompertz Type-4	132.40	63.46	69.64	57.28	55	86.67	750 km, flat-topped
**32**	Logistic Type-4	130.37	56.10	61.38	50.82	55	98.04	750 km, pointy-topped
**32**	Gompertz Type-4	129.92	61.09	67.98	54.21	55	90.03	750 km, pointy-topped
**32**	Logistic Type-5	130.70	58.34	65.15	51.67	55	94.27	750 km, pointy-topped
**32**	Logistic Type-3	131.30	57.10	62.59	51.62	55	96.32	750 km, pointy-topped
**33**	Logistic Type-4	321.10	117.76	124.81	110.71	117	99.36	250 km, flat-topped
**33**	Logistic Type-4	328.91	117.05	123.99	110.10	117	99.96	250 km, pointy-topped
**33**	Gompertz Type-4	327.43	124.08	131.72	116.44	117	94.29	250 km, pointy-topped
**33**	Gompertz Type-4	253.84	82.79	89.67	75.91	80	96.63	500 km, flat-topped
**33**	Logistic Type-4	234.82	82.90	88.09	77.71	81	97.71	500 km, pointy-topped
**33**	Gompertz Type-4	185.80	65.69	70.61	60.77	62	94.38	750 km, flat-topped
**33**	Logistic Type-4	186.23	62.56	66.98	58.15	62	99.10	750 km, flat-topped
**33**	Gompertz Type-4	193.92	64.65	70.25	59.04	63	97.45	750 km, pointy-topped
**34**	Logistic Type-5	243.57	100.94	106.59	95.30	99	98.07	250 km, flat-topped
**34**	Logistic Type-5	226.06	101.96	107.35	96.57	99	97.10	250 km, pointy-topped
**34**	Logistic Type-5	178.90	80.22	85.62	74.88	76	94.74	500 km, flat-topped
**34**	Logistic Type-4	180.90	82.96	87.58	78.34	76	91.61	500 km, flat-topped
**34**	Logistic Type-4	178.09	76.66	81.64	71.68	72	93.92	500 km, pointy-topped
**34**	Logistic Type-4	137.72	64.24	67.78	60.71	61	94.95	750 km, flat-topped
**34**	Logistic Type-5	139.71	64.59	69.18	60.04	61	94.44	750 km, flat-topped
**34**	Logistic Type-4	148.70	57.99	62.06	53.92	58	100.01	750 km, pointy-topped
**34**	Gompertz Type-4	149.51	60.65	65.14	56.16	58	95.64	750 km, pointy-topped
**34**	Logistic Type-5	150.66	58.19	63.19	53.29	58	99.67	750 km, pointy-topped
**35**	Logistic Type-5	318.33	138.70	146.78	130.63	135	97.33	250 km, flat-topped
**35**	Logistic Type-5	324.37	148.45	158.37	138.56	142	95.65	250 km, pointy-topped
**35**	Logistic Type-5	242.67	99.28	104.05	94.51	100	100.73	500 km, flat-topped
**35**	Logistic Type-5	263.88	100.45	105.98	94.91	102	101.55	500 km, pointy-topped
**35**	Logistic Type-5	198.89	77.31	82.46	72.18	76	98.31	750 km, flat-topped
**35**	Logistic Type-5	226.82	78.17	83.10	73.24	79	101.06	750 km, pointy-topped
**36**	Logistic Type-4	319.11	139.98	149.20	130.77	137	97.87	250 km, flat-topped
**36**	Logistic Type-3	318.54	141.37	150.42	132.33	137	96.91	250 km, flat-topped
**36**	Logistic Type-5	319.10	143.08	153.80	132.37	137	95.75	250 km, flat-topped
**36**	Logistic Type-3	319.58	136.46	145.44	127.47	134	98.20	250 km, pointy-topped
**36**	Gompertz Type-4	318.16	148.71	160.44	136.99	134	90.11	250 km, pointy-topped
**36**	Logistic Type-5	317.72	142.94	154.23	131.66	134	93.74	250 km, pointy-topped
**36**	Logistic Type-4	248.59	91.00	97.61	84.39	90	98.90	500 km, flat-topped
**36**	Logistic Type-5	250.59	91.01	98.55	83.48	90	98.89	500 km, flat-topped
**36**	Logistic Type-4	236.37	95.93	102.32	89.55	92	95.90	500 km, pointy-topped
**36**	Logistic Type-5	238.36	96.19	103.97	88.42	92	95.65	500 km, pointy-topped

**36**	Logistic Type-4	230.98	75.44	81.60	69.27	74	98.10	750 km, flat-topped
**36**	Logistic Type-5	232.97	75.52	82.52	68.55	74	97.98	750 km, flat-topped
**36**	Logistic Type-4	204.52	70.67	76.23	65.11	71	100.46	750 km, pointy-topped
**36**	Logistic Type-5	205.06	69.53	75.24	63.83	71	102.11	750 km, pointy-topped
**37**	Logistic Type-5	240.94	111.65	117.93	105.48	108	96.73	250 km, flat-topped
**37**	Logistic Type-5	273.89	123.90	131.10	116.74	118	95.24	250 km, pointy-topped
**37**	Logistic Type-5	172.49	84.90	89.04	80.81	83	97.76	500 km, flat-topped
**37**	Logistic Type-5	208.34	84.84	89.81	79.88	84	99.01	500 km, pointy-topped
**37**	Logistic Type-5	156.54	64.92	69.39	60.58	65	100.12	750 km, flat-topped
**37**	Logistic Type-5	141.74	64.04	67.59	60.50	63	98.38	750 km, pointy-topped
**38**	Gompertz Type-4	258.93	132.41	139.88	124.93	116	87.61	250 km, flat-topped
**38**	Gompertz Type-4	292.03	141.84	150.78	132.90	118	83.19	250 km, pointy-topped
**38**	Logistic Type-5	293.65	136.23	145.29	127.16	118	86.62	250 km, pointy-topped
**38**	Gompertz Type-4	187.31	82.64	87.94	77.35	76	91.96	500 km, flat-topped
**38**	Logistic Type-5	188.66	80.10	86.18	74.04	76	94.88	500 km, flat-topped
**38**	Logistic Type-4	191.77	84.13	89.16	79.10	77	91.53	500 km, pointy-topped
**38**	Logistic Type-5	193.48	85.64	93.78	77.77	77	89.92	500 km, pointy-topped
**38**	Logistic Type-4	133.70	60.36	64.47	56.26	57	94.43	750 km, flat-topped
**38**	Logistic Type-5	133.47	58.54	63.14	54.02	57	97.36	750 km, flat-topped
**38**	Gompertz Type-4	161.19	67.63	72.95	62.31	62	91.68	750 km, pointy-topped
**38**	Logistic Type-4	162.12	63.25	67.78	58.73	62	98.02	750 km, pointy-topped
**38**	Logistic Type-5	162.38	65.41	71.61	59.35	62	94.78	750 km, pointy-topped
**39**	Logistic Type-5	138.72	80.23	89.29	71.55	68	84.76	250 km, flat-topped
**39**	Logistic Type-4	138.08	76.39	81.20	71.58	68	89.02	250 km, flat-topped
**39**	Logistic Type-4	144.56	77.06	81.06	73.07	72	93.43	250 km, pointy-topped
**39**	Gompertz Type-4	95.04	50.55	54.98	46.12	44	87.04	500 km, flat-topped
**39**	Logistic Type-4	95.80	45.65	48.90	42.40	44	96.40	500 km, flat-topped
**39**	Logistic Type-5	95.41	47.89	53.07	42.95	44	91.88	500 km, flat-topped
**39**	Logistic Type-5	104.00	48.76	54.23	43.63	46	94.33	500 km, pointy-topped
**39**	Logistic Type-4	103.29	51.93	57.02	46.85	46	88.58	500 km, pointy-topped
**39**	Logistic Type-5	82.34	35.46	39.46	31.59	35	98.71	750 km, flat-topped
**39**	Logistic Type-5	84.73	39.31	44.11	35.07	38	96.67	750 km, pointy-topped
**40**	Logistic Type-5	279.78	114.65	122.76	106.57	116	101.18	250 km, flat-topped
**40**	Logistic Type-5	288.21	112.39	120.49	104.29	114	101.43	250 km, pointy-topped
**40**	Logistic Type-5	202.71	75.56	83.93	68.01	74	97.93	500 km, flat-topped
**40**	Logistic Type-3	215.84	102.07	122.05	82.17	72	70.54	500 km, pointy-topped
**40**	Logistic Type-4	216.83	98.29	117.32	79.53	72	73.25	500 km, pointy-topped
**40**	Gompertz Type-4	217.14	130.96	167.80	94.43	72	54.98	500 km, pointy-topped
**40**	Logistic Type-4	143.02	71.42	80.07	62.78	57	79.81	750 km, flat-topped
**40**	Logistic Type-3	143.44	74.67	83.93	65.40	57	76.34	750 km, flat-topped
**40**	Logistic Type-5	128.05	56.63	60.03	53.27	57	100.64	750 km, pointy-topped
**41**	Logistic Type-5	318.21	145.86	154.96	136.77	147	100.78	250 km, flat-topped
**41**	Logistic Type-5	333.19	146.91	157.12	136.70	151	102.79	250 km, pointy-topped
**41**	Logistic Type-5	234.63	92.00	98.88	85.17	93	101.08	500 km, flat-topped

**41**	Logistic Type-5	210.10	85.70	91.65	79.78	87	101.51	500 km, pointy-topped
**41**	Logistic Type-5	170.70	66.70	74.13	59.83	64	95.96	750 km, flat-topped
**41**	Logistic Type-5	179.31	67.35	72.57	62.34	68	100.96	750 km, pointy-topped
**42**	Logistic Type-5	271.24	108.96	116.91	101.04	111	101.87	250 km, flat-topped
**42**	Logistic Type-5	298.01	108.59	116.41	100.77	113	104.06	250 km, pointy-topped
**42**	Logistic Type-5	207.46	72.63	77.12	68.14	75	103.26	500 km, flat-topped
**42**	Logistic Type-5	196.26	77.27	82.30	72.24	78	100.95	500 km, pointy-topped
**42**	Logistic Type-5	136.22	57.26	63.62	51.76	55	96.05	750 km, flat-topped
**42**	Logistic Type-4	137.29	60.98	65.89	56.07	55	90.20	750 km, flat-topped
**42**	Logistic Type-5	137.42	57.76	61.32	54.20	58	100.42	750 km, pointy-topped
**43**	Logistic Type-5	129.04	57.82	61.26	54.40	57	98.58	250 km, flat-topped
**43**	Logistic Type-4	110.90	70.10	82.64	57.71	50	71.33	250 km, pointy-topped
**43**	Logistic Type-4	96.99	57.64	73.56	42.81	37	64.19	500 km, flat-topped
**43**	Gompertz Type-4	101.15	91.34	118.04	64.64	36	39.41	500 km, pointy-topped
**43**	Logistic Type-4	80.13	45.40	60.04	32.56	31	68.28	750 km, flat-topped
**43**	Gompertz Type-4	80.96	71.00	131.15	33.07	31	43.66	750 km, flat-topped
**43**	Logistic Type-4	62.13	39.99	54.63	28.25	28	70.01	750 km, pointy-topped
**44**	Logistic Type-5	168.41	149.92	NA	NA	68	45.36	250 km, flat-topped
**44**	Logistic Type-4	166.74	148.20	238.71	80.93	68	45.88	250 km, flat-topped
**44**	Logistic Type-4	188.85	172.35	256.38	97.54	67	38.87	250 km, pointy-topped
**44**	Logistic Type-5	190.83	127.32	169.03	87.97	67	52.63	250 km, pointy-topped
**44**	Logistic Type-4	153.21	118.85	223.51	59.60	58	48.80	500 km, flat-topped
**44**	Logistic Type-5	154.75	104.18	177.52	59.63	58	55.68	500 km, flat-topped
**44**	Logistic Type-4	135.18	111.76	181.04	60.29	50	44.74	500 km, pointy-topped
**44**	Logistic Type-3	136.63	179.18	265.86	95.85	50	27.91	500 km, pointy-topped
**44**	Logistic Type-5	136.37	88.58	NA	NA	50	56.44	500 km, pointy-topped
**44**	Logistic Type-5	140.62	76.34	NA	NA	48	62.87	750 km, flat-topped
**44**	Logistic Type-4	140.95	121.45	229.95	55.77	48	39.52	750 km, flat-topped
**44**	Logistic Type-4	135.02	107.02	NA	NA	48	44.85	750 km, pointy-topped
**44**	Logistic Type-5	136.56	97.25	237.42	45.76	48	49.36	750 km, pointy-topped
**44**	Logistic Type-3	136.37	145.17	234.79	70.17	48	33.07	750 km, pointy-topped
**45**	Logistic Type-5	392.89	203.10	214.37	191.83	207	101.92	250 km, flat-topped
**45**	Logistic Type-5	426.12	231.87	245.34	218.41	232	100.05	250 km, pointy-topped
**45**	Logistic Type-5	314.08	140.70	150.48	130.92	143	101.63	500 km, flat-topped
**45**	Logistic Type-5	314.19	142.50	151.31	133.69	145	101.75	500 km, pointy-topped
**45**	Logistic Type-5	214.98	96.46	103.32	89.61	97	100.56	750 km, flat-topped
**45**	Logistic Type-5	225.82	102.56	109.33	95.78	104	101.41	750 km, pointy-topped
**46**	Logistic Type-5	364.15	155.80	167.44	144.17	158	101.41	250 km, flat-topped
**46**	Logistic Type-5	388.24	166.76	181.35	152.25	169	101.34	250 km, pointy-topped
**46**	Logistic Type-5	317.17	111.76	126.47	99.05	111	99.32	500 km, flat-topped
**46**	Logistic Type-5	271.21	105.05	115.77	94.54	103	98.05	500 km, pointy-topped
**46**	Logistic Type-5	198.05	80.77	87.67	73.92	80	99.04	750 km, flat-topped
**46**	Logistic Type-5	253.47	88.15	114.82	68.89	81	91.89	750 km, pointy-topped
**46**	Logistic Type-4	254.30	105.07	157.21	71.27	81	77.09	750 km, pointy-topped

**46**	Logistic Type-3	253.56	182.14	336.15	85.45	81	44.47	750 km, pointy-topped
**47**	Logistic Type-5	170.19	59.90	64.05	55.75	61	101.84	250 km, flat-topped
**47**	Logistic Type-5	177.22	59.39	64.52	54.28	60	101.03	250 km, pointy-topped
**47**	Logistic Type-4	148.46	45.73	51.39	40.07	45	98.41	500 km, flat-topped
**47**	Logistic Type-5	148.73	44.32	50.01	38.75	45	101.53	500 km, flat-topped
**47**	Logistic Type-5	126.43	44.96	48.73	41.20	46	102.32	500 km, pointy-topped
**47**	Logistic Type-5	106.48	34.34	37.98	30.72	35	101.93	750 km, flat-topped
**47**	Logistic Type-5	116.45	36.36	40.40	32.36	37	101.76	750 km, pointy-topped
**48**	Logistic Type-5	309.45	112.89	121.80	104.08	116	102.76	250 km, flat-topped
**48**	Logistic Type-5	317.94	116.07	126.45	105.71	119	102.52	250 km, pointy-topped
**48**	Logistic Type-5	225.83	77.83	84.56	71.12	80	102.79	500 km, flat-topped
**48**	Logistic Type-5	217.67	77.77	84.06	71.48	79	101.59	500 km, pointy-topped
**48**	Logistic Type-5	172.17	60.75	65.76	55.73	62	102.07	750 km, flat-topped
**48**	Logistic Type-5	174.19	64.01	69.07	58.96	65	101.54	750 km, pointy-topped
**49**	Logistic Type-5	295.35	105.25	115.27	95.71	105	99.76	250 km, flat-topped
**49**	Logistic Type-5	308.37	106.86	115.97	97.97	107	100.13	250 km, pointy-topped
**49**	Logistic Type-5	220.35	76.16	83.91	68.78	76	99.79	500 km, flat-topped
**49**	Logistic Type-5	228.50	75.65	82.96	68.71	75	99.15	500 km, pointy-topped
**49**	Logistic Type-5	188.44	57.95	64.79	51.84	58	100.09	750 km, flat-topped
**49**	Logistic Type-5	185.81	58.38	64.20	52.82	59	101.05	750 km, pointy-topped
**50**	Logistic Type-4	214.95	92.87	98.07	87.68	93	100.14	250 km, flat-topped
**50**	Logistic Type-5	214.33	91.14	96.51	85.77	93	102.04	250 km, flat-topped
**50**	Logistic Type-5	223.23	87.83	93.41	82.26	91	103.61	250 km, pointy-topped
**50**	Logistic Type-5	176.25	58.44	62.95	53.93	61	104.39	500 km, flat-topped
**50**	Logistic Type-5	182.71	59.66	64.34	54.99	62	103.92	500 km, pointy-topped
**50**	Logistic Type-5	140.52	45.91	49.54	42.30	47	102.36	750 km, flat-topped
**50**	Logistic Type-5	125.83	41.75	45.05	38.46	45	107.78	750 km, pointy-topped
**51**	Gompertz Type-4	238.91	81.66	89.56	73.76	79	96.74	250 km, flat-topped
**51**	Logistic Type-5	238.53	77.99	86.48	69.69	79	101.29	250 km, flat-topped
**51**	Logistic Type-4	237.68	75.95	82.69	69.21	79	104.02	250 km, flat-topped
**51**	Logistic Type-4	244.07	72.97	80.13	65.81	78	106.89	250 km, pointy-topped
**51**	Logistic Type-5	246.03	73.28	81.33	65.30	78	106.44	250 km, pointy-topped
**51**	Logistic Type-4	136.37	54.79	58.62	50.97	53	96.73	500 km, flat-topped
**51**	Logistic Type-5	138.34	54.97	59.99	50.04	53	96.42	500 km, flat-topped
**51**	Logistic Type-5	140.57	47.63	51.88	43.45	49	102.87	500 km, pointy-topped
**51**	Logistic Type-4	141.72	50.05	54.38	45.72	49	97.90	500 km, pointy-topped
**51**	Gompertz Type-4	114.25	43.39	47.62	39.17	41	94.48	750 km, flat-topped
**51**	Logistic Type-5	114.93	41.29	45.81	36.92	41	99.30	750 km, flat-topped
**51**	Logistic Type-4	113.83	40.44	43.92	36.97	41	101.37	750 km, flat-topped
**51**	Logistic Type-5	85.94	34.67	37.15	32.21	36	103.83	750 km, pointy-topped
**52**	Logistic Type-5	355.04	122.90	133.38	112.43	128	104.15	250 km, flat-topped
**52**	Logistic Type-5	362.35	118.67	129.26	108.08	125	105.34	250 km, pointy-topped
**52**	Logistic Type-5	267.01	78.17	85.57	70.82	80	102.34	500 km, flat-topped
**52**	Logistic Type-5	242.75	75.58	82.43	68.73	79	104.53	500 km, pointy-topped

**52**	Logistic Type-5	217.00	63.66	70.31	57.07	66	103.67	750 km, flat-topped
**52**	Logistic Type-5	209.94	59.88	65.97	53.88	62	103.54	750 km, pointy-topped
**53**	Logistic Type-5	299.06	125.70	134.18	117.22	131	104.22	250 km, flat-topped
**53**	Logistic Type-5	299.96	126.58	134.44	118.71	130	102.71	250 km, pointy-topped
**53**	Logistic Type-4	301.31	128.88	136.66	121.10	130	100.87	250 km, pointy-topped
**53**	Logistic Type-5	215.39	78.55	84.52	72.58	83	105.66	500 km, flat-topped
**53**	Logistic Type-5	242.93	83.14	89.86	76.43	87	104.64	500 km, pointy-topped
**53**	Logistic Type-5	153.11	63.55	67.26	59.85	65	102.28	750 km, flat-topped
**53**	Logistic Type-4	172.48	68.06	72.62	63.49	68	99.91	750 km, pointy-topped
**53**	Logistic Type-5	172.96	66.54	71.54	61.57	68	102.20	750 km, pointy-topped
**54**	Logistic Type-5	318.67	132.65	141.87	123.42	139	104.79	250 km, flat-topped
**54**	Logistic Type-5	343.42	142.23	152.63	131.83	149	104.76	250 km, pointy-topped
**54**	Logistic Type-4	272.06	99.27	107.41	91.13	100	100.73	500 km, flat-topped
**54**	Logistic Type-5	252.28	98.57	105.65	91.49	102	103.48	500 km, pointy-topped
**54**	Logistic Type-5	193.86	75.01	80.40	69.65	77	102.65	750 km, flat-topped
**54**	Logistic Type-5	214.76	79.84	86.38	73.32	83	103.95	750 km, pointy-topped
**55**	Logistic Type-5	311.27	160.51	166.70	154.32	162	100.93	250 km, flat-topped
**55**	Logistic Type-5	327.88	174.46	181.07	167.85	176	100.88	250 km, pointy-topped
**55**	Logistic Type-5	226.52	110.41	115.09	105.73	110	99.63	500 km, flat-topped
**55**	Logistic Type-5	215.34	106.35	110.31	102.40	108	101.55	500 km, pointy-topped
**55**	Logistic Type-5	194.83	83.37	88.26	78.47	84	100.76	750 km, flat-topped
**55**	Logistic Type-5	208.97	85.53	90.16	80.90	86	100.56	750 km, pointy-topped
**56**	Logistic Type-5	432.25	169.04	180.98	157.09	177	104.71	250 km, flat-topped
**56**	Logistic Type-5	413.32	167.29	178.69	155.89	176	105.21	250 km, pointy-topped
**56**	Logistic Type-5	296.53	102.59	109.80	95.39	106	103.32	500 km, flat-topped
**56**	Logistic Type-5	291.03	98.73	105.06	92.40	103	104.33	500 km, pointy-topped
**56**	Logistic Type-5	220.59	71.03	77.07	64.99	75	105.59	750 km, flat-topped
**56**	Logistic Type-5	217.75	73.85	79.40	68.31	77	104.27	750 km, pointy-topped
**57**	Logistic Type-5	361.62	150.48	159.44	141.55	146	97.02	250 km, flat-topped
**57**	Logistic Type-5	358.97	156.63	165.07	148.18	152	97.05	250 km, pointy-topped
**57**	Logistic Type-5	239.47	97.55	104.32	91.19	94	96.36	500 km, flat-topped
**57**	Logistic Type-5	247.99	100.32	106.95	93.79	97	96.69	500 km, pointy-topped
**57**	Logistic Type-5	213.97	77.35	83.89	70.96	74	95.67	750 km, flat-topped
**57**	Logistic Type-5	197.92	78.89	83.85	74.00	78	98.87	750 km, pointy-topped
**58**	Logistic Type-5	342.57	154.58	162.30	146.86	151	97.68	250 km, flat-topped
**58**	Logistic Type-4	362.52	166.59	176.08	157.09	155	93.04	250 km, pointy-topped
**58**	Logistic Type-5	363.24	162.57	174.24	150.98	155	95.34	250 km, pointy-topped
**58**	Logistic Type-5	262.76	102.03	110.25	93.86	98	96.05	500 km, flat-topped
**58**	Logistic Type-4	260.77	102.20	109.03	95.37	98	95.89	500 km, flat-topped
**58**	Logistic Type-5	276.27	96.75	103.17	90.35	97	100.26	500 km, pointy-topped
**58**	Logistic Type-5	219.47	78.10	85.17	71.16	78	99.87	750 km, flat-topped
**58**	Logistic Type-5	210.98	71.28	78.30	64.28	73	102.42	750 km, pointy-topped
**58**	Logistic Type-4	210.51	72.66	79.72	65.60	73	100.47	750 km, pointy-topped
**59**	Logistic Type-4	282.56	134.98	140.43	129.53	129	95.57	250 km, flat-topped

**59**	Logistic Type-5	281.28	131.69	137.75	125.62	129	97.96	250 km, flat-topped
**59**	Logistic Type-5	273.89	128.27	133.34	123.20	129	100.57	250 km, pointy-topped
**59**	Logistic Type-4	220.79	86.38	91.00	81.76	86	99.56	500 km, flat-topped
**59**	Logistic Type-5	220.94	84.81	89.82	79.80	86	101.41	500 km, flat-topped
**59**	Logistic Type-5	204.24	85.20	90.08	80.32	85	99.77	500 km, pointy-topped
**59**	Logistic Type-4	213.30	70.69	75.61	65.76	72	101.86	750 km, flat-topped
**59**	Gompertz Type-4	214.15	76.10	82.09	70.11	72	94.61	750 km, flat-topped
**59**	Logistic Type-5	214.39	72.41	79.21	65.77	72	99.43	750 km, flat-topped
**59**	Logistic Type-5	207.19	65.77	70.76	60.80	68	103.39	750 km, pointy-topped
**60**	Logistic Type-3	115.87	80.79	88.26	73.33	56	69.31	250 km, flat-topped
**60**	Gompertz Type-4	114.93	103.49	110.02	96.96	56	54.11	250 km, flat-topped
**60**	Logistic Type-3	129.02	98.18	112.47	83.89	57	58.05	250 km, pointy-topped
**60**	Logistic Type-4	128.00	88.84	102.20	75.49	57	64.16	250 km, pointy-topped
**60**	Logistic Type-4	85.71	75.33	91.80	58.86	43	57.08	500 km, flat-topped
**60**	Logistic Type-3	84.23	78.66	92.17	65.16	43	54.66	500 km, flat-topped
**60**	Logistic Type-5	82.86	54.40	64.46	45.85	44	80.88	500 km, pointy-topped
**60**	Logistic Type-4	84.36	66.84	76.76	56.92	44	65.83	500 km, pointy-topped
**60**	Logistic Type-5	78.66	56.90	NA	NA	37	65.03	750 km, flat-topped
**60**	Logistic Type-4	76.74	59.23	73.79	44.86	37	62.47	750 km, flat-topped
**60**	Logistic Type-3	76.78	67.21	80.74	53.67	37	55.05	750 km, flat-topped
**60**	Logistic Type-3	93.27	85.04	111.86	58.22	38	44.69	750 km, pointy-topped
**60**	Logistic Type-4	94.30	73.77	104.93	46.46	38	51.51	750 km, pointy-topped
**61**	Logistic Type-5	101.56	37.15	42.14	32.33	37	99.60	250 km, flat-topped
**61**	Logistic Type-5	109.66	41.41	45.50	37.42	41	99.00	250 km, pointy-topped
**61**	Logistic Type-5	55.90	25.92	28.59	23.35	26	100.31	500 km, flat-topped
**61**	Logistic Type-5	57.58	26.40	29.97	23.16	26	98.49	500 km, pointy-topped
**61**	Logistic Type-4	58.81	28.04	31.99	24.13	26	92.73	500 km, pointy-topped
**61**	Logistic Type-5	48.78	21.27	24.42	18.32	21	98.74	750 km, flat-topped
**61**	Logistic Type-4	47.42	21.63	24.51	18.76	21	97.07	750 km, flat-topped
**61**	Logistic Type-5	49.21	22.19	24.95	19.55	22	99.12	750 km, pointy-topped
**62**	Logistic Type-5	228.06	91.38	102.54	80.53	81	88.64	250 km, flat-topped
**62**	Logistic Type-4	226.10	90.37	98.25	82.50	81	89.63	250 km, flat-topped
**62**	Logistic Type-4	199.78	85.56	91.19	79.94	79	92.33	250 km, pointy-topped
**62**	Logistic Type-4	151.34	60.81	66.36	55.26	56	92.09	500 km, flat-topped
**62**	Logistic Type-5	150.01	57.36	62.87	52.00	56	97.62	500 km, flat-topped
**62**	Logistic Type-5	166.26	55.32	61.82	49.14	54	97.61	500 km, pointy-topped
**62**	Logistic Type-4	166.99	59.00	65.77	52.24	54	91.52	500 km, pointy-topped
**62**	Logistic Type-4	93.13	49.86	55.00	44.72	41	82.23	750 km, flat-topped
**62**	Logistic Type-4	125.45	58.08	67.35	48.82	45	77.48	750 km, pointy-topped
**62**	Logistic Type-5	126.79	53.71	64.99	44.39	45	83.79	750 km, pointy-topped
**63**	Gompertz Type-4	208.64	74.71	81.50	67.92	74	99.05	250 km, flat-topped
**63**	Gompertz Type-4	193.77	74.41	79.96	68.87	74	99.44	250 km, pointy-topped
**63**	Logistic Type-5	150.73	48.24	54.33	42.34	47	97.44	500 km, flat-topped
**63**	Logistic Type-4	149.44	49.17	54.97	43.39	47	95.58	500 km, flat-topped

**63**	Gompertz Type-4	145.42	54.00	61.24	46.79	50	92.59	500 km, pointy-topped
**63**	Gompertz Type-4	114.74	43.29	49.32	37.31	39	90.09	750 km, flat-topped
**63**	Gompertz Type-4	128.21	42.91	47.96	37.86	42	97.88	750 km, pointy-topped
**64**	Gompertz Type-3	151.62	65.95	70.88	61.01	67	101.60	250 km, flat-topped
**64**	Gompertz Type-4	153.56	65.86	70.92	60.80	67	101.73	250 km, flat-topped
**64**	Gompertz Type-4	156.92	66.23	72.03	60.42	65	98.15	250 km, pointy-topped
**64**	Gompertz Type-3	155.71	65.80	71.39	60.20	65	98.79	250 km, pointy-topped
**64**	Gompertz Type-4	111.69	42.33	46.70	37.96	44	103.95	500 km, flat-topped
**64**	Gompertz Type-3	111.63	42.93	47.39	38.47	44	102.50	500 km, flat-topped
**64**	Gompertz Type-3	99.51	47.88	52.00	43.75	44	91.90	500 km, pointy-topped
**64**	Gompertz Type-4	101.51	47.85	52.23	43.46	44	91.96	500 km, pointy-topped
**64**	Gompertz Type-3	77.74	34.40	37.13	31.66	34	98.85	750 km, flat-topped
**64**	Gompertz Type-4	79.13	34.15	36.96	31.34	34	99.56	750 km, flat-topped
**64**	Gompertz Type-4	85.81	34.26	37.79	30.72	35	102.17	750 km, pointy-topped
**65**	Gompertz Type-4	391.25	173.43	187.97	158.89	146	84.19	250 km, flat-topped
**65**	Gompertz Type-4	378.43	165.32	177.91	152.72	144	87.11	250 km, pointy-topped
**65**	Gompertz Type-4	255.29	99.76	107.52	92.00	91	91.22	500 km, flat-topped
**65**	Gompertz Type-4	243.25	107.02	115.18	98.87	92	85.96	500 km, pointy-topped
**65**	Gompertz Type-4	200.49	70.13	76.08	64.18	68	96.96	750 km, flat-topped
**65**	Gompertz Type-4	177.18	72.66	78.43	66.88	66	90.84	750 km, pointy-topped
**66**	Logistic Type-4	370.98	168.50	184.88	152.13	141	83.68	250 km, flat-topped
**66**	Logistic Type-5	370.44	147.49	169.60	130.39	141	95.60	250 km, flat-topped
**66**	Logistic Type-5	374.60	139.62	153.55	126.59	136	97.41	250 km, pointy-topped
**66**	Logistic Type-4	375.52	149.97	161.49	138.45	136	90.68	250 km, pointy-topped
**66**	Logistic Type-4	254.59	101.37	113.07	89.66	86	84.84	500 km, flat-topped
**66**	Logistic Type-5	253.91	90.32	103.48	79.35	86	95.21	500 km, flat-topped
**66**	Logistic Type-5	244.47	89.56	102.18	79.53	87	97.14	500 km, pointy-topped
**66**	Logistic Type-5	193.19	60.39	67.12	54.29	61	101.01	750 km, flat-topped
**66**	Logistic Type-5	179.88	62.35	68.07	57.03	62	99.44	750 km, pointy-topped
**67**	Logistic Type-5	282.73	154.37	161.83	146.91	149	96.52	250 km, flat-topped
**67**	Logistic Type-5	291.02	145.63	153.44	137.83	141	96.82	250 km, pointy-topped
**67**	Logistic Type-4	199.74	90.08	95.00	85.16	83	92.14	500 km, flat-topped
**67**	Logistic Type-5	201.50	88.98	96.18	81.95	83	93.28	500 km, flat-topped
**67**	Logistic Type-4	177.17	92.98	98.66	87.30	86	92.49	500 km, pointy-topped
**67**	Logistic Type-5	178.94	94.37	103.29	85.72	86	91.13	500 km, pointy-topped
**67**	Logistic Type-5	156.63	68.34	73.75	63.02	66	96.58	750 km, flat-topped
**67**	Logistic Type-4	155.13	69.56	73.92	65.19	66	94.89	750 km, flat-topped
**67**	Logistic Type-4	153.03	70.15	74.93	65.38	66	94.08	750 km, pointy-topped
**67**	Logistic Type-5	154.91	70.66	77.07	64.37	66	93.41	750 km, pointy-topped
**68**	Gompertz Type-4	466.67	229.57	246.11	213.03	193	84.07	250 km, flat-topped
**68**	Gompertz Type-4	418.42	233.05	247.89	218.21	197	84.53	250 km, pointy-topped
**68**	Gompertz Type-4	301.48	130.44	138.58	122.30	118	90.46	500 km, flat-topped
**68**	Gompertz Type-4	314.01	124.12	133.76	114.48	114	91.85	500 km, pointy-topped
**68**	Gompertz Type-4	252.47	97.54	104.36	90.72	90	92.27	750 km, flat-topped

**68**	Gompertz Type-4	255.30	103.57	112.40	94.74	93	89.79	750 km, pointy-topped
**69**	Logistic Type-5	395.03	155.70	166.10	145.32	153	98.26	250 km, flat-topped
**69**	Logistic Type-5	371.37	154.59	165.24	143.94	152	98.33	250 km, pointy-topped
**69**	Logistic Type-4	280.83	110.67	118.90	102.44	104	93.97	500 km, flat-topped
**69**	Logistic Type-4	292.56	121.36	130.57	112.15	113	93.11	500 km, pointy-topped
**69**	Logistic Type-5	294.56	121.38	133.95	109.17	113	93.10	500 km, pointy-topped
**69**	Gompertz Type-4	216.83	92.74	100.34	85.14	78	84.11	750 km, flat-topped
**69**	Logistic Type-5	216.83	81.26	87.89	74.70	80	98.45	750 km, pointy-topped
**69**	Logistic Type-4	214.86	81.60	86.96	76.24	80	98.04	750 km, pointy-topped
**70**	Logistic Type-5	243.04	110.79	116.81	104.77	113	102.00	250 km, flat-topped
**70**	Logistic Type-5	237.78	104.26	110.46	98.07	107	102.62	250 km, pointy-topped
**70**	Logistic Type-5	169.12	74.60	78.87	70.34	77	103.22	500 km, flat-topped
**70**	Logistic Type-5	170.77	72.72	77.31	68.14	73	100.38	500 km, pointy-topped
**70**	Logistic Type-5	141.65	59.00	62.72	55.28	60	101.70	750 km, flat-topped
**70**	Logistic Type-5	148.33	59.84	63.98	55.73	62	103.60	750 km, pointy-topped
**71**	Logistic Type-4	379.48	178.95	187.43	170.47	183	102.26	250 km, flat-topped
**71**	Logistic Type-5	378.02	181.02	190.02	172.01	183	101.09	250 km, flat-topped
**71**	Logistic Type-5	337.10	175.31	184.05	166.58	176	100.39	250 km, pointy-topped
**71**	Logistic Type-4	335.73	174.38	182.25	166.50	176	100.93	250 km, pointy-topped
**71**	Logistic Type-5	256.99	108.28	115.40	101.16	110	101.59	500 km, flat-topped
**71**	Logistic Type-4	255.23	108.85	115.33	102.36	110	101.06	500 km, flat-topped
**71**	Logistic Type-4	258.03	113.36	118.68	108.03	113	99.69	500 km, pointy-topped
**71**	Logistic Type-5	258.52	112.04	117.67	106.41	113	100.86	500 km, pointy-topped
**71**	Logistic Type-5	225.68	89.44	95.49	83.39	90	100.63	750 km, flat-topped
**71**	Logistic Type-4	223.76	89.16	94.60	83.73	90	100.94	750 km, flat-topped
**71**	Logistic Type-4	204.32	84.57	89.72	79.41	86	101.69	750 km, pointy-topped
**72**	Logistic Type-4	195.41	75.72	80.15	71.29	76	100.37	250 km, flat-topped
**72**	Logistic Type-5	196.56	76.59	82.13	71.09	76	99.23	250 km, flat-topped
**72**	Logistic Type-4	195.29	69.14	73.74	64.55	70	101.24	250 km, pointy-topped
**72**	Logistic Type-5	129.25	49.51	52.96	46.05	51	103.02	500 km, flat-topped
**72**	Logistic Type-4	129.25	50.09	53.52	46.66	51	101.82	500 km, flat-topped
**72**	Logistic Type-5	147.83	50.49	54.11	46.88	52	102.99	500 km, pointy-topped
**72**	Logistic Type-4	147.86	51.43	54.92	47.94	52	101.11	500 km, pointy-topped
**72**	Logistic Type-5	130.45	46.29	49.95	42.65	47	101.53	750 km, flat-topped
**72**	Logistic Type-5	119.64	45.96	49.45	42.47	47	102.27	750 km, pointy-topped
**73**	Gompertz Type-4	253.24	124.10	132.06	116.14	118	95.08	250 km, flat-topped
**73**	Gompertz Type-4	254.27	120.75	130.23	111.28	118	97.72	250 km, pointy-topped
**73**	Gompertz Type-4	183.81	88.40	94.61	82.19	85	96.15	500 km, flat-topped
**73**	Logistic Type-5	184.95	86.62	93.46	79.84	85	98.12	500 km, flat-topped
**73**	Gompertz Type-4	199.56	92.84	99.67	86.01	88	94.79	500 km, pointy-topped
**73**	Logistic Type-4	138.62	64.84	67.91	61.78	66	101.78	750 km, flat-topped
**73**	Logistic Type-5	140.15	65.32	68.67	61.97	66	101.04	750 km, flat-topped
**73**	Gompertz Type-4	163.77	68.69	74.27	63.12	70	101.90	750 km, pointy-topped
**74**	Logistic Type-3	198.13	104.20	111.44	96.96	102	97.89	250 km, flat-topped

**74**	Logistic Type-4	199.34	103.69	111.02	96.36	102	98.37	250 km, flat-topped
**74**	Gompertz Type-4	205.63	113.56	123.61	103.51	106	93.34	250 km, pointy-topped
**74**	Logistic Type-3	206.08	106.91	115.70	98.13	106	99.15	250 km, pointy-topped
**74**	Logistic Type-5	206.34	110.09	120.27	99.93	106	96.29	250 km, pointy-topped
**74**	Logistic Type-4	158.80	72.19	78.53	65.85	72	99.74	500 km, flat-topped
**74**	Logistic Type-5	160.05	71.44	77.92	64.96	72	100.78	500 km, flat-topped
**74**	Logistic Type-3	150.03	76.19	82.53	69.85	75	98.44	500 km, pointy-topped
**74**	Logistic Type-4	150.46	75.39	81.75	69.04	75	99.48	500 km, pointy-topped
**74**	Logistic Type-4	126.20	60.99	66.55	55.43	58	95.10	750 km, flat-topped
**74**	Logistic Type-5	125.65	58.82	64.63	53.16	58	98.60	750 km, flat-topped
**74**	Logistic Type-4	105.37	54.80	59.79	49.81	53	96.71	750 km, pointy-topped
**74**	Logistic Type-5	104.91	52.79	57.99	47.71	53	100.40	750 km, pointy-topped
**75**	Logistic Type-5	233.87	108.33	114.57	102.09	104	96.01	250 km, flat-topped
**75**	Logistic Type-4	235.12	112.20	117.97	106.44	104	92.69	250 km, flat-topped
**75**	Logistic Type-4	223.33	107.80	114.65	100.95	99	91.84	250 km, pointy-topped
**75**	Logistic Type-5	225.04	110.38	NA	NA	99	89.69	250 km, pointy-topped
**75**	Logistic Type-4	150.14	69.81	73.46	66.15	68	97.41	500 km, flat-topped
**75**	Logistic Type-5	150.71	68.51	72.46	64.56	68	99.26	500 km, flat-topped
**75**	Logistic Type-5	188.83	73.96	79.30	68.64	72	97.35	500 km, pointy-topped
**75**	Logistic Type-4	189.55	76.49	81.59	71.38	72	94.14	500 km, pointy-topped
**75**	Logistic Type-5	130.87	52.68	56.98	48.42	52	98.72	750 km, flat-topped
**75**	Logistic Type-4	131.24	54.56	58.67	50.46	52	95.30	750 km, flat-topped
**75**	Logistic Type-5	140.24	57.40	62.86	52.04	54	94.07	750 km, pointy-topped
**75**	Logistic Type-4	141.07	60.62	66.15	55.08	54	89.08	750 km, pointy-topped
**76**	Logistic Type-4	78.16	38.81	42.23	35.40	35	90.18	250 km, flat-topped
**76**	Gompertz Type-4	77.63	45.91	51.49	40.32	35	76.24	250 km, flat-topped
**76**	Logistic Type-4	78.82	38.08	40.81	35.36	35	91.90	250 km, pointy-topped
**76**	Logistic Type-5	79.48	36.35	39.75	33.15	35	96.29	250 km, pointy-topped
**76**	Logistic Type-4	71.67	33.96	37.98	29.96	31	91.28	500 km, flat-topped
**76**	Gompertz Type-4	72.20	38.09	43.83	32.35	31	81.39	500 km, flat-topped
**76**	Gompertz Type-4	73.59	33.66	38.28	29.06	29	86.14	500 km, pointy-topped
**76**	Logistic Type-5	60.16	25.57	29.96	21.94	24	93.86	750 km, flat-topped
**76**	Logistic Type-4	58.19	25.83	28.84	22.83	24	92.92	750 km, flat-topped
**76**	Logistic Type-4	67.04	23.87	26.47	21.26	25	104.76	750 km, pointy-topped
**76**	Gompertz Type-4	67.17	24.89	27.76	22.01	25	100.45	750 km, pointy-topped
**76**	Logistic Type-5	68.84	24.24	28.20	21.03	25	103.15	750 km, pointy-topped
**77**	Gompertz Type-4	266.88	120.32	131.35	109.28	112	93.09	250 km, flat-topped
**77**	Logistic Type-4	264.61	124.89	135.25	114.53	119	95.28	250 km, pointy-topped
**77**	Logistic Type-5	266.59	125.45	137.71	113.30	119	94.86	250 km, pointy-topped
**77**	Gompertz Type-4	221.19	96.04	106.50	85.58	92	95.79	500 km, flat-topped
**77**	Gompertz Type-4	225.88	103.29	116.31	90.29	92	89.07	500 km, pointy-topped
**77**	Logistic Type-5	227.66	97.36	109.52	85.38	92	94.49	500 km, pointy-topped
**77**	Logistic Type-4	227.44	94.42	104.48	84.36	92	97.44	500 km, pointy-topped
**77**	Logistic Type-4	182.13	70.50	77.53	63.47	70	99.29	750 km, flat-topped

**77**	Gompertz Type-4	165.56	69.74	77.16	62.32	68	97.50	750 km, pointy-topped
**78**	Logistic Type-5	99.84	35.39	39.54	31.28	36	101.73	250 km, flat-topped
**78**	Logistic Type-4	101.40	36.67	41.25	32.10	36	98.16	250 km, flat-topped
**78**	Gompertz Type-4	101.61	39.50	45.94	33.22	36	91.14	250 km, pointy-topped
**78**	Logistic Type-4	103.15	36.60	41.71	31.53	36	98.36	250 km, pointy-topped
**78**	Logistic Type-4	69.95	25.30	28.73	21.91	25	98.81	500 km, flat-topped
**78**	Gompertz Type-4	70.91	26.78	31.10	22.58	25	93.36	500 km, flat-topped
**78**	Logistic Type-4	66.97	41.76	73.99	23.70	25	59.86	500 km, pointy-topped
**78**	Logistic Type-5	68.86	44.19	139.37	20.73	25	56.57	500 km, pointy-topped
**78**	Gompertz Type-4	68.42	100.74	134.59	66.89	25	24.82	500 km, pointy-topped
**78**	Logistic Type-5	65.58	24.02	31.21	19.26	23	95.74	750 km, flat-topped
**78**	Logistic Type-4	64.35	27.18	35.82	20.37	23	84.62	750 km, flat-topped
**78**	Logistic Type-5	62.90	23.10	29.97	18.30	22	95.25	750 km, pointy-topped
**78**	Logistic Type-4	61.45	24.17	31.26	18.68	22	91.02	750 km, pointy-topped
**79**	Logistic Type-5	262.38	110.90	117.49	104.33	109	98.28	250 km, flat-topped
**79**	Logistic Type-5	251.28	116.69	123.99	109.39	113	96.84	250 km, pointy-topped
**79**	Logistic Type-4	187.06	76.51	82.32	70.70	72	94.10	500 km, flat-topped
**79**	Logistic Type-5	188.29	75.31	82.04	68.66	72	95.61	500 km, flat-topped
**79**	Logistic Type-5	179.21	73.79	79.24	68.35	72	97.58	500 km, pointy-topped
**79**	Logistic Type-4	179.76	76.07	81.30	70.85	72	94.64	500 km, pointy-topped
**79**	Logistic Type-4	176.52	58.24	63.32	53.16	58	99.58	750 km, flat-topped
**79**	Logistic Type-5	174.96	56.80	61.70	51.90	58	102.12	750 km, flat-topped
**79**	Gompertz Type-4	161.56	62.40	66.92	57.89	60	96.15	750 km, pointy-topped
**80**	Logistic Type-5	138.10	55.32	59.37	51.31	55	99.42	250 km, flat-topped
**80**	Logistic Type-4	136.87	56.25	59.84	52.66	55	97.78	250 km, flat-topped
**80**	Logistic Type-4	134.86	59.03	62.57	55.50	56	94.86	250 km, pointy-topped
**80**	Logistic Type-5	133.75	56.67	60.44	52.92	56	98.83	250 km, pointy-topped
**80**	Logistic Type-4	108.21	44.98	48.24	41.72	44	97.82	500 km, flat-topped
**80**	Logistic Type-5	110.06	44.48	48.65	40.49	44	98.91	500 km, flat-topped
**80**	Gompertz Type-4	123.01	50.27	55.83	44.70	45	89.52	500 km, pointy-topped
**80**	Gompertz Type-4	97.62	38.53	41.95	35.12	37	96.02	750 km, flat-topped
**80**	Logistic Type-5	97.18	35.40	38.59	32.29	36	101.69	750 km, pointy-topped
**80**	Logistic Type-4	96.34	36.40	39.36	33.45	36	98.89	750 km, pointy-topped
**81**	Logistic Type-4	161.44	82.37	85.95	78.78	75	91.06	250 km, flat-topped
**81**	Logistic Type-5	161.56	80.08	84.54	75.63	75	93.66	250 km, flat-topped
**81**	Logistic Type-4	152.14	79.25	82.54	75.97	74	93.37	250 km, pointy-topped
**81**	Logistic Type-5	153.45	78.26	82.54	73.99	74	94.55	250 km, pointy-topped
**81**	Gompertz Type-4	131.32	63.71	68.02	59.39	54	84.76	500 km, flat-topped
**81**	Logistic Type-5	139.49	59.04	64.20	53.98	54	91.46	500 km, pointy-topped
**81**	Logistic Type-4	137.89	57.79	61.15	54.43	54	93.44	500 km, pointy-topped
**81**	Logistic Type-4	127.13	56.89	61.94	51.84	50	87.89	750 km, flat-topped
**81**	Logistic Type-5	127.16	53.73	59.39	48.40	50	93.07	750 km, flat-topped
**81**	Gompertz Type-4	128.26	55.49	61.60	49.38	46	82.90	750 km, pointy-topped
**82**	Logistic Type-5	193.24	82.85	91.41	74.80	77	92.94	250 km, flat-topped

**82**	Logistic Type-5	202.12	104.66	143.51	77.92	77	73.57	250 km, pointy-topped
**82**	Logistic Type-4	201.96	118.06	147.65	89.33	77	65.22	250 km, pointy-topped
**82**	Logistic Type-5	167.03	58.65	66.02	51.84	58	98.89	500 km, flat-topped
**82**	Logistic Type-5	165.17	61.23	69.27	53.79	60	97.99	500 km, pointy-topped
**82**	Logistic Type-4	164.38	64.59	72.27	56.95	60	92.89	500 km, pointy-topped
**82**	Logistic Type-4	135.14	59.79	70.04	49.80	50	83.62	750 km, flat-topped
**82**	Logistic Type-5	136.04	54.87	65.52	46.03	50	91.12	750 km, flat-topped
**82**	Logistic Type-5	121.05	46.60	51.56	41.92	46	98.71	750 km, pointy-topped
**83**	Logistic Type-5	658.85	261.05	276.98	245.11	270	103.43	250 km, flat-topped
**83**	Logistic Type-5	705.83	261.56	279.53	243.58	273	104.37	250 km, pointy-topped
**83**	Logistic Type-5	492.67	147.42	158.35	136.50	152	103.10	500 km, flat-topped
**83**	Logistic Type-5	504.96	151.24	162.40	140.08	159	105.13	500 km, pointy-topped
**83**	Logistic Type-5	334.36	100.45	108.03	92.87	104	103.54	750 km, flat-topped
**83**	Logistic Type-5	394.46	105.14	114.31	95.98	110	104.62	750 km, pointy-topped
**84**	Logistic Type-5	512.31	185.21	198.58	171.85	194	104.75	250 km, flat-topped
**84**	Logistic Type-5	527.39	180.50	193.21	167.80	193	106.92	250 km, pointy-topped
**84**	Logistic Type-5	350.80	113.84	123.65	104.03	120	105.41	500 km, flat-topped
**84**	Logistic Type-5	342.40	108.79	118.67	98.92	117	107.54	500 km, pointy-topped
**84**	Logistic Type-5	274.78	82.16	89.59	74.72	87	105.90	750 km, flat-topped
**84**	Logistic Type-5	280.78	82.57	89.55	75.59	88	106.57	750 km, pointy-topped
**85**	Logistic Type-5	520.81	212.32	228.64	196.01	222	104.56	250 km, flat-topped
**85**	Logistic Type-5	510.10	215.05	230.13	199.97	225	104.63	250 km, pointy-topped
**85**	Logistic Type-5	380.38	118.46	129.36	107.56	124	104.68	500 km, flat-topped
**85**	Logistic Type-5	401.56	121.07	131.21	110.92	128	105.73	500 km, pointy-topped
**85**	Logistic Type-5	292.83	80.39	87.76	73.02	85	105.74	750 km, flat-topped
**85**	Logistic Type-5	293.04	81.91	89.52	74.30	86	104.99	750 km, pointy-topped
**86**	Logistic Type-4	118.26	76.31	82.03	70.59	58	76.01	250 km, flat-topped
**86**	Gompertz Type-4	118.37	96.33	104.37	88.30	58	60.21	250 km, flat-topped
**86**	Logistic Type-3	116.50	77.17	82.17	72.16	58	75.16	250 km, flat-topped
**86**	Gompertz Type-4	128.95	97.08	107.45	86.71	59	60.77	250 km, pointy-topped
**86**	Logistic Type-3	129.35	75.62	81.19	70.05	59	78.02	250 km, pointy-topped
**86**	Gompertz Type-3	130.56	102.79	112.94	92.65	59	57.40	250 km, pointy-topped
**86**	Gompertz Type-3	105.63	86.15	94.64	77.67	43	49.91	500 km, flat-topped
**86**	Gompertz Type-4	106.85	81.34	90.89	71.79	43	52.87	500 km, flat-topped
**86**	Logistic Type-4	107.87	63.71	71.24	56.19	47	73.77	500 km, pointy-topped
**86**	Logistic Type-3	108.76	67.17	74.72	59.62	47	69.98	500 km, pointy-topped
**86**	Logistic Type-4	73.66	44.07	49.31	38.84	34	77.15	750 km, flat-topped
**86**	Logistic Type-5	74.75	46.37	54.69	38.35	34	73.33	750 km, flat-topped
**86**	Gompertz Type-4	72.86	55.81	63.96	47.67	34	60.92	750 km, flat-topped
**86**	Logistic Type-3	72.98	45.54	50.56	40.51	34	74.67	750 km, flat-topped
**86**	Logistic Type-3	73.68	59.07	71.81	46.33	34	57.56	750 km, pointy-topped
**86**	Logistic Type-4	74.14	54.42	67.12	41.84	34	62.47	750 km, pointy-topped
**86**	Gompertz Type-4	75.51	77.93	83.98	71.89	34	43.63	750 km, pointy-topped
**87**	Logistic Type-5	438.53	187.81	197.76	177.86	193	102.76	250 km, flat-topped

**87**	Logistic Type-5	455.29	182.64	193.40	171.89	190	104.03	250 km, pointy-topped
**87**	Logistic Type-5	295.77	108.47	114.29	102.65	112	103.25	500 km, flat-topped
**87**	Logistic Type-5	318.37	113.72	119.80	107.65	117	102.88	500 km, pointy-topped
**87**	Logistic Type-5	265.37	87.92	92.66	83.19	92	104.64	750 km, flat-topped
**87**	Logistic Type-5	274.00	86.35	91.91	80.80	89	103.06	750 km, pointy-topped
**88**	Logistic Type-5	371.30	188.61	198.54	178.67	178	94.38	250 km, flat-topped
**88**	Logistic Type-4	370.07	191.39	199.42	183.35	178	93.00	250 km, flat-topped
**88**	Logistic Type-4	394.94	198.73	209.08	188.38	180	90.57	250 km, pointy-topped
**88**	Logistic Type-3	395.02	200.87	211.06	190.68	180	89.61	250 km, pointy-topped
**88**	Logistic Type-5	287.32	119.07	127.13	111.10	115	96.58	500 km, flat-topped
**88**	Logistic Type-4	270.77	122.44	127.95	116.93	111	90.65	500 km, pointy-topped
**88**	Logistic Type-5	269.78	119.01	125.96	112.07	111	93.27	500 km, pointy-topped
**88**	Logistic Type-5	204.21	83.52	87.56	79.48	84	100.58	750 km, flat-topped
**88**	Logistic Type-5	201.73	80.35	85.47	75.25	78	97.08	750 km, pointy-topped
**89**	Logistic Type-5	219.51	85.84	93.55	78.17	86	100.18	250 km, flat-topped
**89**	Logistic Type-5	219.75	87.92	95.32	80.58	88	100.09	250 km, pointy-topped
**89**	Logistic Type-5	140.98	61.66	67.17	56.45	60	97.31	500 km, flat-topped
**89**	Logistic Type-5	153.87	65.63	73.70	58.19	63	95.99	500 km, pointy-topped
**89**	Logistic Type-5	110.23	51.52	55.90	47.36	50	97.04	750 km, flat-topped
**89**	Logistic Type-5	111.73	56.54	66.84	48.04	49	86.66	750 km, pointy-topped
**89**	Logistic Type-4	110.74	59.16	65.85	52.47	49	82.83	750 km, pointy-topped
**90**	Gompertz Type-4	155.76	52.92	59.20	46.65	49	92.58	250 km, flat-topped
**90**	Gompertz Type-3	154.50	53.70	59.87	47.53	49	91.25	250 km, flat-topped
**90**	Gompertz Type-4	144.11	50.43	54.51	46.35	50	99.15	250 km, pointy-topped
**90**	Gompertz Type-3	115.26	42.87	46.96	38.78	40	93.31	500 km, flat-topped
**90**	Gompertz Type-4	113.46	41.61	45.49	37.73	40	96.13	500 km, flat-topped
**90**	Gompertz Type-3	124.16	41.60	46.25	36.94	39	93.76	500 km, pointy-topped
**90**	Gompertz Type-4	122.93	40.31	44.75	35.87	39	96.75	500 km, pointy-topped
**90**	Gompertz Type-4	81.72	32.71	36.37	29.06	31	94.76	750 km, flat-topped
**90**	Gompertz Type-3	81.81	33.54	37.25	29.84	31	92.42	750 km, flat-topped
**90**	Gompertz Type-4	82.20	29.82	33.37	26.26	29	97.25	750 km, pointy-topped
**90**	Gompertz Type-3	83.64	30.75	34.62	26.89	29	94.30	750 km, pointy-topped
**91**	Logistic Type-5	46.90	28.07	30.51	25.80	28	99.74	250 km, flat-topped
**91**	Logistic Type-4	45.81	28.82	30.74	26.91	28	97.14	250 km, flat-topped
**91**	Logistic Type-5	53.27	33.94	36.25	31.70	34	100.17	250 km, pointy-topped
**91**	Logistic Type-4	54.79	35.38	37.60	33.16	34	96.10	250 km, pointy-topped
**91**	Logistic Type-5	56.87	30.08	32.19	27.98	31	103.06	500 km, flat-topped
**91**	Logistic Type-5	47.11	25.76	28.07	23.58	26	100.95	500 km, pointy-topped
**91**	Logistic Type-3	45.26	27.65	29.89	25.40	26	94.05	750 km, flat-topped
**91**	Logistic Type-5	43.67	25.72	27.79	23.69	26	101.08	750 km, flat-topped
**91**	Logistic Type-4	44.03	26.83	28.96	24.70	26	96.90	750 km, flat-topped
**91**	Logistic Type-5	35.09	30.25	31.85	28.66	30	99.19	750 km, pointy-topped
**92**	Logistic Type-3	147.78	70.20	75.68	64.71	65	92.60	250 km, flat-topped
**92**	Logistic Type-5	147.52	65.63	71.68	59.73	65	99.04	250 km, flat-topped

**92**	Logistic Type-4	146.72	67.78	73.26	62.30	65	95.90	250 km, flat-topped
**92**	Logistic Type-3	144.49	67.30	73.35	61.25	61	90.63	250 km, pointy-topped
**92**	Logistic Type-5	145.94	61.82	69.50	54.97	61	98.67	250 km, pointy-topped
**92**	Logistic Type-4	146.45	66.81	74.56	59.11	61	91.31	250 km, pointy-topped
**92**	Logistic Type-5	150.33	51.76	58.70	45.28	53	102.40	500 km, flat-topped
**92**	Logistic Type-3	148.81	56.55	62.97	50.12	53	93.73	500 km, flat-topped
**92**	Logistic Type-4	149.39	54.13	60.91	47.40	53	97.92	500 km, flat-topped
**92**	Logistic Type-4	115.13	50.02	54.91	45.14	48	95.96	500 km, pointy-topped
**92**	Logistic Type-5	114.01	47.85	53.16	42.94	48	100.31	500 km, pointy-topped
**92**	Logistic Type-4	68.86	44.98	50.37	39.62	38	84.48	750 km, flat-topped
**92**	Gompertz Type-4	69.43	49.46	55.46	43.46	38	76.83	750 km, flat-topped
**92**	Gompertz Type-3	67.95	48.14	52.03	44.24	38	78.94	750 km, flat-topped
**92**	Logistic Type-4	90.49	47.93	53.30	42.58	43	89.72	750 km, pointy-topped
**92**	Logistic Type-3	88.83	47.05	50.78	43.32	43	91.40	750 km, pointy-topped
**93**	Logistic Type-5	219.13	81.83	86.18	77.48	84	102.65	250 km, flat-topped
**93**	Logistic Type-5	226.70	85.81	91.51	80.23	88	102.56	250 km, pointy-topped
**93**	Logistic Type-5	166.63	59.71	63.11	56.30	62	103.84	500 km, flat-topped
**93**	Logistic Type-5	154.46	60.80	63.61	58.00	62	101.97	500 km, pointy-topped
**93**	Logistic Type-5	143.87	51.25	54.45	48.06	53	103.41	750 km, flat-topped
**93**	Logistic Type-5	128.36	49.44	51.19	48.45	51	103.15	750 km, pointy-topped
**94**	Expontential	36.11	88.31	4086033.77	-61675.78	14	15.85	250 km, flat-topped
**94**	Expontential	42.04	94.44	4369737.82	-61510.31	15	15.88	250 km, pointy-topped
**94**	Expontential	28.40	65.02	NA	NA	12	18.46	500 km, flat-topped
**94**	Expontential	30.98	81.09	3856455.28	-59444.95	13	16.03	500 km, pointy-topped
**94**	insufficient data	NA	NA	NA	NA	NA	NA	750 km, flat-topped
**94**	Gompertz Type-3	27.96	28.38	NA	NA	11	38.76	750 km, pointy-topped
**94**	Logistic Type-3	27.92	25.79	61.24	8.80	11	42.65	750 km, pointy-topped
**94**	Logistic Type-4	29.81	29.86	98.14	7.11	11	36.84	750 km, pointy-topped
**95**	Logistic Type-5	428.84	218.22	230.21	206.23	210	96.23	250 km, flat-topped
**95**	Logistic Type-4	429.13	213.06	222.76	203.35	210	98.57	250 km, flat-topped
**95**	Logistic Type-4	420.04	212.56	221.27	203.85	208	97.85	250 km, pointy-topped
**95**	Logistic Type-5	421.15	210.41	220.26	200.55	208	98.86	250 km, pointy-topped
**95**	Logistic Type-5	338.97	128.05	135.63	120.46	129	100.75	500 km, flat-topped
**95**	Logistic Type-4	341.04	122.72	130.13	115.32	125	101.85	500 km, pointy-topped
**95**	Logistic Type-5	342.64	123.91	132.28	115.55	125	100.88	500 km, pointy-topped
**95**	Logistic Type-5	255.57	85.28	92.11	78.46	88	103.19	750 km, flat-topped
**95**	Logistic Type-4	255.48	86.90	93.61	80.19	88	101.27	750 km, flat-topped
**95**	Logistic Type-5	275.28	90.67	96.41	84.93	92	101.46	750 km, pointy-topped
**96**	Gompertz Type-4	150.30	101.43	105.83	97.03	98	96.62	250 km, flat-topped
**96**	Logistic Type-5	150.99	100.31	104.93	95.69	98	97.70	250 km, flat-topped
**96**	Gompertz Type-4	151.57	100.17	104.97	95.37	99	98.83	250 km, pointy-topped
**96**	Logistic Type-5	114.72	69.91	73.35	66.46	71	101.56	500 km, flat-topped
**96**	Logistic Type-4	113.27	69.76	73.08	66.45	71	101.77	500 km, flat-topped
**96**	Logistic Type-3	114.54	70.08	73.50	66.66	71	101.32	500 km, flat-topped

**96**	Logistic Type-5	110.46	68.69	72.37	65.02	68	98.99	500 km, pointy-topped
**96**	Gompertz Type-4	109.17	69.65	73.13	66.18	68	97.62	500 km, pointy-topped
**96**	Gompertz Type-4	91.39	57.80	61.65	53.96	54	93.42	750 km, flat-topped
**96**	Logistic Type-5	91.59	56.17	59.91	52.44	54	96.13	750 km, flat-topped
**96**	Logistic Type-4	93.12	54.65	58.04	51.25	54	98.82	750 km, flat-topped
**96**	Logistic Type-3	93.26	55.03	58.46	51.60	54	98.13	750 km, flat-topped
**96**	Logistic Type-4	100.11	58.63	61.39	55.88	59	100.63	750 km, pointy-topped
**97**	Gompertz Type-4	121.31	51.61	57.98	45.26	52	100.76	250 km, flat-topped
**97**	Logistic Type-3	120.09	49.53	54.86	44.20	52	104.99	250 km, flat-topped
**97**	Gompertz Type-3	121.72	53.68	60.04	47.31	52	96.88	250 km, flat-topped
**97**	Logistic Type-4	121.87	50.00	56.02	44.00	52	104.00	250 km, flat-topped
**97**	Logistic Type-4	118.87	47.71	53.09	42.35	49	102.71	250 km, pointy-topped
**97**	Logistic Type-3	117.37	47.03	51.70	42.36	49	104.18	250 km, pointy-topped
**97**	Gompertz Type-3	119.16	51.33	56.98	45.69	49	95.45	250 km, pointy-topped
**97**	Gompertz Type-3	97.73	41.32	46.61	36.03	40	96.80	500 km, flat-topped
**97**	Logistic Type-3	99.71	38.08	42.80	33.37	40	105.04	500 km, flat-topped
**97**	Gompertz Type-4	99.66	40.88	47.34	34.64	40	97.84	500 km, flat-topped
**97**	Gompertz Type-4	95.39	41.42	NA	NA	37	89.33	500 km, pointy-topped
**97**	Gompertz Type-3	93.86	38.38	43.26	33.50	37	96.40	500 km, pointy-topped
**97**	Logistic Type-4	95.76	37.50	42.88	32.22	37	98.66	500 km, pointy-topped
**97**	Logistic Type-3	79.86	31.07	34.36	27.78	32	102.99	750 km, flat-topped
**97**	Logistic Type-4	80.02	30.55	34.20	26.90	32	104.75	750 km, pointy-topped
**97**	Logistic Type-3	78.18	30.83	34.24	27.41	32	103.81	750 km, pointy-topped
**98**	insufficient data	NA	NA	NA	NA	NA	NA	250 km, flat-topped
**98**	insufficient data	NA	NA	NA	NA	NA	NA	250 km, pointy-topped
**98**	insufficient data	NA	NA	NA	NA	NA	NA	500 km, flat-topped
**98**	insufficient data	NA	NA	NA	NA	NA	NA	500 km, pointy-topped
**98**	insufficient data	NA	NA	NA	NA	NA	NA	750 km, flat-topped
**98**	insufficient data	NA	NA	NA	NA	NA	NA	750 km, pointy-topped

Table S6.

Results for the logistic and logistic-like (Gompertz) models estimating fossil data coverage of marine regions (not shallow-water only) based on collection history for the 49-bin approach. Predicted completeness refers to the percentage of the observed number of grid cells containing fossil data relative to the predicted value for 2100 (see Methods for details).

Bin ID	Model	AIC	Predicted no. cells	Upper CI	Lower CI	Obser-ved no. cells with fossils	Predicted complete-ness	Grid type
**1**	Logistic Type-3	486.44	363.14	417.47	308.81	195	53.70	250 km, pointy-topped
**1**	Logistic Type-4	488.39	362.09	426.48	297.70	195	53.85	250 km, pointy-topped
**1**	Logistic Type-3	423.16	425.99	515.79	336.19	137	32.16	500 km, pointy-topped
**1**	Logistic Type-4	424.54	386.34	478.81	293.87	137	35.46	500 km, pointy-topped
**1**	Logistic Type-3	358.62	238.50	308.45	168.59	114	47.80	750 km, pointy-topped
**1**	Logistic Type-3	341.24	273.55	347.52	199.59	107	39.11	750 km, flat-topped
**1**	Logistic Type-5	342.02	186.40	270.01	124.25	107	57.40	750 km, flat-topped
**1**	Logistic Type-5	418.30	208.56	NA	NA	133	63.77	500 km, flat-topped
**1**	Logistic Type-3	482.06	425.91	515.06	336.76	194	45.55	250 km, flat-topped
**1**	Logistic Type-4	420.15	280.67	387.32	180.69	133	47.39	500 km, flat-topped
**1**	Logistic Type-5	423.09	226.78	293.04	168.63	137	60.41	500 km, pointy-topped
**1**	Logistic Type-5	358.02	193.20	485.66	107.48	114	59.01	750 km, pointy-topped
**1**	Logistic Type-3	418.34	297.87	385.67	210.08	133	44.65	500 km, flat-topped
**1**	Logistic Type-4	342.92	252.38	328.47	176.29	107	42.40	750 km, flat-topped
**1**	Logistic Type-4	358.05	207.84	275.47	144.11	114	54.85	750 km, pointy-topped
**2**	Gompertz Type-3	312.52	125.66	137.24	114.08	103	81.97	750 km, pointy-topped
**2**	Gompertz Type-4	505.89	240.22	265.27	215.17	179	74.52	250 km, flat-topped
**2**	Gompertz Type-4	367.81	155.29	171.26	139.33	126	81.14	500 km, pointy-topped
**2**	Gompertz Type-4	311.76	122.35	133.65	111.04	103	84.19	750 km, pointy-topped
**2**	Gompertz Type-4	367.81	156.71	173.98	139.44	123	78.49	500 km, flat-topped
**2**	Gompertz Type-3	492.44	236.15	259.62	212.68	178	75.38	250 km, pointy-topped
**2**	Gompertz Type-3	367.68	159.32	175.51	143.14	126	79.08	500 km, pointy-topped
**2**	Gompertz Type-3	503.96	241.80	266.17	217.43	179	74.03	250 km, flat-topped
**2**	Gompertz Type-4	493.29	230.74	254.54	206.94	178	77.14	250 km, pointy-topped
**2**	Gompertz Type-4	293.63	110.85	122.16	99.55	99	89.31	750 km, flat-topped
**2**	Gompertz Type-3	367.26	160.75	178.24	143.26	123	76.52	500 km, flat-topped
**3**	Gompertz Type-3	72.85	94.11	NA	NA	25	26.57	500 km, pointy-topped
**3**	Logistic Type-3	61.89	62.88	110.63	26.27	21	33.40	750 km, pointy-topped
**3**	Logistic Type-3	72.79	68.55	119.48	30.45	25	36.47	500 km, pointy-topped
**3**	Logistic Type-3	80.14	69.47	113.57	34.92	30	43.19	250 km, flat-topped
**3**	Gompertz Type-3	61.91	66.93	NA	NA	21	31.37	750 km, pointy-topped
**3**	Logistic Type-4	82.08	66.63	110.20	33.90	30	45.03	250 km, flat-topped
**3**	Logistic Type-3	58.01	77.76	118.56	38.50	22	28.29	500 km, flat-topped
**3**	Gompertz Type-3	59.09	74.32	NA	NA	22	29.60	500 km, flat-topped
**3**	Gompertz Type-3	80.14	97.07	NA	NA	30	30.91	250 km, flat-topped

**3**	Logistic Type-4	74.58	80.71	160.65	28.74	25	30.97	500 km, pointy-topped
**3**	Gompertz Type-4	74.70	80.46	NA	NA	25	31.07	500 km, pointy-topped
**3**	Logistic Type-5	57.83	61.93	161.27	26.89	22	35.52	750 km, flat-topped
**3**	Gompertz Type-4	81.19	80.55	106.30	54.79	30	37.25	250 km, flat-topped
**3**	Logistic Type-3	56.28	82.78	122.01	43.69	22	26.58	750 km, flat-topped
**3**	Gompertz Type-4	63.59	64.69	NA	NA	21	32.46	750 km, pointy-topped
**3**	Gompertz Type-3	83.18	65.72	74.14	57.30	31	47.17	250 km, pointy-topped
**3**	Gompertz Type-4	84.48	60.39	71.79	48.99	31	51.33	250 km, pointy-topped
**3**	Logistic Type-4	57.01	78.04	134.81	30.63	22	28.19	750 km, flat-topped
**4**	Logistic Type-3	199.86	91.46	104.49	78.43	68	74.35	500 km, flat-topped
**4**	Logistic Type-4	289.47	151.48	178.58	124.39	100	66.01	250 km, pointy-topped
**4**	Gompertz Type-4	184.82	100.92	118.48	83.37	61	60.44	750 km, pointy-topped
**4**	Gompertz Type-4	175.92	100.04	119.52	80.57	60	59.97	750 km, flat-topped
**4**	Logistic Type-4	201.52	89.75	103.90	75.60	68	75.77	500 km, flat-topped
**4**	Logistic Type-3	213.43	108.76	122.93	94.59	78	71.72	500 km, pointy-topped
**4**	Logistic Type-3	287.95	156.02	180.93	131.11	100	64.09	250 km, pointy-topped
**4**	Logistic Type-4	215.11	106.80	122.41	91.18	78	73.03	500 km, pointy-topped
**4**	Logistic Type-4	186.76	83.19	98.70	67.67	61	73.33	750 km, pointy-topped
**4**	Logistic Type-5	272.75	99.88	107.00	92.77	99	99.12	250 km, flat-topped
**4**	Logistic Type-3	174.88	84.71	101.36	68.07	60	70.83	750 km, flat-topped
**4**	Logistic Type-3	185.07	84.78	99.41	70.15	61	71.95	750 km, pointy-topped
**4**	Logistic Type-4	176.54	82.58	99.57	65.64	60	72.66	750 km, flat-topped
**4**	Gompertz Type-3	186.28	108.69	125.03	92.35	61	56.12	750 km, pointy-topped
**5**	Gompertz Type-3	531.71	328.08	340.32	315.85	199	60.66	250 km, flat-topped
**5**	Gompertz Type-3	345.83	153.12	163.74	142.50	101	65.96	750 km, flat-topped
**5**	Gompertz Type-3	426.65	197.29	211.21	183.37	136	68.94	500 km, flat-topped
**5**	Gompertz Type-4	533.64	326.84	339.42	314.26	199	60.89	250 km, flat-topped
**5**	Gompertz Type-4	406.11	188.96	203.20	174.73	138	73.03	500 km, pointy-topped
**5**	Gompertz Type-3	560.18	318.69	333.30	304.08	205	64.33	250 km, pointy-topped
**5**	Gompertz Type-4	310.14	137.82	148.19	127.46	101	73.28	750 km, pointy-topped
**5**	Gompertz Type-4	562.09	317.32	332.10	302.53	205	64.60	250 km, pointy-topped
**5**	Gompertz Type-4	427.17	191.54	206.70	176.37	136	71.00	500 km, flat-topped
**5**	Gompertz Type-4	347.24	148.67	160.81	136.54	101	67.93	750 km, flat-topped
**6**	Logistic Type-3	140.08	61.51	69.65	53.37	47	76.41	750 km, pointy-topped
**6**	Logistic Type-5	148.11	50.20	55.33	45.44	50	99.61	500 km, pointy-topped
**6**	Logistic Type-5	227.08	86.92	101.97	73.63	73	83.99	250 km, pointy-topped
**6**	Logistic Type-4	198.96	81.11	88.03	74.19	70	86.30	250 km, flat-topped
**6**	Logistic Type-4	139.39	54.81	64.18	45.63	46	83.93	750 km, flat-topped
**6**	Gompertz Type-4	140.94	68.29	82.35	54.23	47	68.83	750 km, pointy-topped
**6**	Logistic Type-4	140.05	57.18	65.33	49.05	47	82.20	750 km, pointy-topped
**6**	Logistic Type-4	225.19	88.95	98.12	79.77	73	82.07	250 km, pointy-topped
**6**	Logistic Type-5	198.59	75.04	83.15	67.44	70	93.29	250 km, flat-topped
**6**	Logistic Type-5	151.29	61.80	70.91	53.63	54	87.38	500 km, flat-topped
**6**	Logistic Type-4	149.41	63.16	69.16	57.17	54	85.49	500 km, flat-topped

**6**	Logistic Type-5	141.47	59.79	71.07	49.14	47	78.61	750 km, pointy-topped
**6**	Logistic Type-3	138.04	57.36	65.57	49.14	46	80.20	750 km, flat-topped
**7**	Logistic Type-4	360.67	167.50	175.82	159.19	131	78.21	500 km, pointy-topped
**7**	Logistic Type-5	538.39	283.78	310.04	257.52	202	71.18	250 km, flat-topped
**7**	Logistic Type-4	536.76	292.98	310.74	275.22	202	68.95	250 km, flat-topped
**7**	Logistic Type-5	363.18	181.46	195.07	167.86	134	73.84	500 km, flat-topped
**7**	Logistic Type-4	361.60	187.49	197.85	177.13	134	71.47	500 km, flat-topped
**7**	Logistic Type-5	296.31	118.62	129.72	107.78	104	87.67	750 km, flat-topped
**7**	Logistic Type-4	296.77	135.63	144.70	126.56	104	76.68	750 km, flat-topped
**7**	Logistic Type-5	280.00	115.21	123.81	106.77	105	91.14	750 km, pointy-topped
**7**	Gompertz Type-3	535.86	342.24	352.50	331.98	200	58.44	250 km, pointy-topped
**7**	Gompertz Type-4	537.22	336.39	347.21	325.57	200	59.45	250 km, pointy-topped
**8**	Logistic Type-3	267.62	132.51	142.57	122.46	94	70.94	750 km, pointy-topped
**8**	Logistic Type-3	385.05	193.09	210.71	175.46	127	65.77	500 km, pointy-topped
**8**	Logistic Type-4	385.80	200.43	223.39	177.46	127	63.36	500 km, pointy-topped
**8**	Logistic Type-4	269.59	133.44	146.40	120.47	94	70.45	750 km, pointy-topped
**8**	Gompertz Type-4	519.42	388.28	403.66	372.90	190	48.93	250 km, flat-topped
**8**	Gompertz Type-3	519.93	404.81	418.55	391.06	190	46.94	250 km, flat-topped
**8**	Gompertz Type-4	499.85	319.72	333.81	305.64	184	57.55	250 km, pointy-topped
**8**	Gompertz Type-3	386.26	256.24	265.59	246.89	127	49.56	500 km, pointy-topped
**8**	Gompertz Type-4	382.99	209.27	221.33	197.21	126	60.21	500 km, flat-topped
**8**	Gompertz Type-4	385.58	242.49	252.90	232.09	127	52.37	500 km, pointy-topped
**8**	Gompertz Type-3	383.42	223.06	233.31	212.80	126	56.49	500 km, flat-topped
**8**	Logistic Type-4	384.30	181.73	203.34	160.12	126	69.33	500 km, flat-topped
**8**	Logistic Type-3	384.54	171.47	186.85	156.09	126	73.48	500 km, flat-topped
**8**	Logistic Type-3	292.71	148.35	161.92	134.79	97	65.38	750 km, flat-topped
**8**	Logistic Type-4	293.65	154.44	172.37	136.50	97	62.81	750 km, flat-topped
**8**	Gompertz Type-3	294.23	191.90	199.20	184.60	97	50.55	750 km, flat-topped
**8**	Gompertz Type-4	294.32	182.97	191.04	174.91	97	53.01	750 km, flat-topped
**9**	Gompertz Type-3	257.49	165.36	172.71	158.00	89	53.82	250 km, flat-topped
**9**	Gompertz Type-4	175.81	65.58	75.78	55.39	50	76.24	750 km, pointy-topped
**9**	Gompertz Type-3	273.03	173.09	181.44	164.74	92	53.15	250 km, pointy-topped
**9**	Gompertz Type-4	192.25	111.16	117.56	104.75	65	58.48	500 km, pointy-topped
**9**	Gompertz Type-3	173.91	64.38	73.39	55.38	50	77.66	750 km, pointy-topped
**9**	Gompertz Type-3	192.41	104.00	111.19	96.81	65	62.50	500 km, pointy-topped
**9**	Gompertz Type-4	191.16	82.48	93.59	71.36	60	72.75	500 km, flat-topped
**9**	Gompertz Type-4	274.82	172.49	181.77	163.20	92	53.34	250 km, pointy-topped
**9**	Gompertz Type-3	189.27	83.31	92.96	73.66	60	72.02	500 km, flat-topped
**9**	Gompertz Type-3	143.93	69.52	75.30	63.74	52	74.80	750 km, flat-topped
**9**	Gompertz Type-4	145.59	68.53	75.08	61.97	52	75.88	750 km, flat-topped
**9**	Gompertz Type-4	258.99	168.90	177.32	160.47	89	52.69	250 km, flat-topped
**10**	Gompertz Type-3	336.38	147.24	154.59	139.89	114	77.43	750 km, pointy-topped
**10**	Gompertz Type-4	710.38	399.38	418.37	380.39	250	62.60	250 km, flat-topped
**10**	Gompertz Type-3	482.85	216.76	229.09	204.43	160	73.81	500 km, pointy-topped

**10**	Gompertz Type-4	484.79	218.36	231.82	204.90	160	73.27	500 km, pointy-topped
**10**	Gompertz Type-4	482.53	209.59	221.92	197.26	160	76.34	500 km, flat-topped
**10**	Gompertz Type-4	661.50	443.84	461.20	426.48	253	57.00	250 km, pointy-topped
**10**	Gompertz Type-3	710.83	385.46	406.46	364.45	250	64.86	250 km, flat-topped
**10**	Gompertz Type-3	482.29	212.95	224.72	201.18	160	75.13	500 km, flat-topped
**10**	Gompertz Type-3	349.38	149.84	157.90	141.78	114	76.08	750 km, flat-topped
**10**	Gompertz Type-4	338.20	146.45	154.41	138.50	114	77.84	750 km, pointy-topped
**10**	Gompertz Type-3	662.10	424.41	442.74	406.08	253	59.61	250 km, pointy-topped
**10**	Gompertz Type-4	351.12	148.82	157.50	140.14	114	76.60	750 km, flat-topped
**11**	Logistic Type-5	148.23	147.13	NA	NA	45	30.59	500 km, pointy-topped
**11**	Logistic Type-4	165.39	136.85	189.33	84.89	53	38.73	250 km, flat-topped
**11**	Logistic Type-3	146.36	136.89	172.90	100.88	45	32.87	500 km, pointy-topped
**11**	Logistic Type-5	164.14	130.84	235.36	70.71	53	40.51	250 km, flat-topped
**11**	Logistic Type-5	166.17	118.84	261.47	61.41	55	46.28	250 km, pointy-topped
**11**	Logistic Type-4	166.27	187.97	265.70	110.31	55	29.26	250 km, pointy-topped
**11**	Logistic Type-3	120.62	100.40	131.15	69.65	36	35.86	750 km, pointy-topped
**11**	Logistic Type-3	123.57	95.32	116.91	73.73	37	38.82	750 km, flat-topped
**11**	Logistic Type-4	147.68	133.50	187.82	79.18	45	33.71	500 km, pointy-topped
**11**	Logistic Type-3	127.72	118.79	151.43	86.16	39	32.83	500 km, flat-topped
**12**	Gompertz Type-4	393.82	246.70	258.14	235.26	142	57.56	250 km, flat-topped
**12**	Logistic Type-5	359.06	229.03	238.88	219.18	97	42.35	500 km, pointy-topped
**12**	Gompertz Type-4	428.29	268.27	282.13	254.41	138	51.44	250 km, pointy-topped
**12**	Logistic Type-4	358.85	200.65	239.02	162.28	97	48.34	500 km, pointy-topped
**12**	Gompertz Type-4	254.81	146.01	155.96	136.06	77	52.74	750 km, pointy-topped
**12**	Logistic Type-4	232.50	148.46	167.75	129.18	74	49.84	750 km, flat-topped
**12**	Gompertz Type-4	358.06	214.26	221.50	207.02	97	45.27	500 km, pointy-topped
**12**	Logistic Type-4	311.52	207.55	228.14	186.96	100	48.18	500 km, flat-topped
**12**	Gompertz Type-4	310.89	219.57	NA	NA	100	45.54	500 km, flat-topped
**12**	Gompertz Type-4	232.12	150.00	152.35	147.66	74	49.33	750 km, flat-topped
**13**	Gompertz Type-3	226.93	96.83	109.11	84.56	66	68.16	750 km, pointy-topped
**13**	Gompertz Type-3	277.39	150.16	160.59	139.74	85	56.60	500 km, pointy-topped
**13**	Gompertz Type-4	363.05	217.32	233.80	200.84	114	52.46	250 km, pointy-topped
**13**	Gompertz Type-4	306.41	127.13	140.79	113.47	82	64.50	500 km, flat-topped
**13**	Gompertz Type-3	371.65	182.54	200.42	164.67	118	64.64	250 km, flat-topped
**13**	Gompertz Type-4	373.58	181.92	200.11	163.73	118	64.86	250 km, flat-topped
**13**	Gompertz Type-3	304.44	127.99	141.06	114.93	82	64.07	500 km, flat-topped
**13**	Gompertz Type-3	260.29	111.45	121.35	101.55	66	59.22	750 km, flat-topped
**13**	Gompertz Type-4	227.05	91.26	104.02	78.50	66	72.32	750 km, pointy-topped
**13**	Gompertz Type-3	361.78	206.53	218.89	194.18	114	55.20	250 km, pointy-topped
**13**	Gompertz Type-4	261.88	107.72	118.88	96.55	66	61.27	750 km, flat-topped
**13**	Gompertz Type-4	279.19	149.27	160.50	138.03	85	56.94	500 km, pointy-topped
**14**	Gompertz Type-4	429.45	230.69	240.66	220.71	167	72.39	250 km, flat-topped
**14**	Gompertz Type-4	227.32	130.87	137.24	124.49	92	70.30	750 km, pointy-topped
**14**	Gompertz Type-4	283.25	163.15	169.83	156.48	113	69.26	500 km, pointy-topped

**14**	Gompertz Type-4	450.12	219.11	227.65	210.57	169	77.13	250 km, pointy-topped
**14**	Gompertz Type-4	304.27	148.80	156.17	141.43	112	75.27	500 km, flat-topped
**14**	Gompertz Type-3	226.81	133.17	139.09	127.25	92	69.09	750 km, pointy-topped
**14**	Gompertz Type-4	245.07	122.18	128.58	115.79	91	74.48	750 km, flat-topped
**15**	Gompertz Type-4	312.02	118.46	127.38	109.54	107	90.32	250 km, flat-topped
**15**	Gompertz Type-3	310.02	118.40	126.84	109.95	107	90.37	250 km, flat-topped
**15**	Gompertz Type-4	222.17	84.87	90.80	78.93	74	87.20	500 km, pointy-topped
**15**	Gompertz Type-4	194.51	69.35	75.63	63.07	62	89.40	750 km, pointy-topped
**15**	Gompertz Type-3	220.80	85.58	91.29	79.87	74	86.47	500 km, pointy-topped
**15**	Gompertz Type-3	193.39	68.61	74.57	62.64	62	90.37	750 km, pointy-topped
**15**	Gompertz Type-4	313.19	119.24	127.42	111.07	107	89.73	250 km, pointy-topped
**15**	Gompertz Type-3	242.31	78.88	85.43	72.33	72	91.28	500 km, flat-topped
**15**	Gompertz Type-3	312.45	120.52	128.55	112.50	107	88.78	250 km, pointy-topped
**15**	Gompertz Type-4	170.87	65.34	71.51	59.16	56	85.71	750 km, flat-topped
**15**	Gompertz Type-4	243.91	78.35	85.09	71.62	72	91.89	500 km, flat-topped
**15**	Gompertz Type-3	169.70	64.57	70.37	58.76	56	86.73	750 km, flat-topped
**16**	Logistic Type-3	162.27	66.35	69.85	62.86	63	94.95	750 km, pointy-topped
**16**	Logistic Type-5	160.33	68.42	72.85	63.99	63	92.08	750 km, pointy-topped
**16**	Gompertz Type-4	299.99	116.09	124.39	107.80	99	85.28	250 km, flat-topped
**16**	Gompertz Type-4	219.95	91.49	99.48	83.50	72	78.70	500 km, pointy-topped
**16**	Logistic Type-5	185.76	87.44	93.86	81.03	73	83.48	500 km, flat-topped
**16**	Gompertz Type-4	161.10	72.72	77.02	68.41	63	86.64	750 km, pointy-topped
**16**	Gompertz Type-4	184.21	95.07	101.26	88.88	73	76.79	500 km, flat-topped
**16**	Gompertz Type-4	298.59	136.31	145.45	127.18	108	79.23	250 km, pointy-topped
**16**	Gompertz Type-4	143.40	70.31	73.94	66.68	62	88.18	750 km, flat-topped
**16**	Logistic Type-5	142.24	66.79	70.43	63.16	62	92.82	750 km, flat-topped
**17**	Gompertz Type-3	219.19	111.74	117.03	106.45	85	76.07	500 km, pointy-topped
**17**	Logistic Type-4	184.85	79.05	83.53	74.57	71	89.81	750 km, pointy-topped
**17**	Logistic Type-5	184.89	82.85	88.80	76.90	71	85.70	750 km, pointy-topped
**17**	Gompertz Type-4	178.81	111.93	116.29	107.58	85	75.94	500 km, flat-topped
**17**	Gompertz Type-4	219.40	110.26	115.87	104.66	85	77.09	500 km, pointy-topped
**17**	Logistic Type-3	183.83	78.06	81.89	74.22	71	90.96	750 km, pointy-topped
**17**	Gompertz Type-3	177.90	112.73	116.83	108.62	85	75.40	500 km, flat-topped
**17**	Gompertz Type-4	332.27	165.31	176.87	153.75	116	70.17	250 km, flat-topped
**17**	Gompertz Type-3	330.70	163.37	174.48	152.25	116	71.01	250 km, flat-topped
**17**	Gompertz Type-4	157.84	93.16	97.45	88.87	71	76.22	750 km, flat-topped
**17**	Gompertz Type-4	320.26	162.16	172.84	151.48	115	70.92	250 km, pointy-topped
**18**	Gompertz Type-3	169.45	152.52	NA	NA	62	40.65	500 km, flat-topped
**18**	Gompertz Type-4	139.91	125.15	NA	NA	54	43.15	750 km, pointy-topped
**18**	Gompertz Type-3	138.16	124.87	NA	NA	54	43.24	750 km, pointy-topped
**18**	Gompertz Type-4	153.16	124.88	NA	NA	54	43.24	750 km, flat-topped
**18**	Gompertz Type-4	229.99	171.78	181.22	162.33	83	48.32	250 km, flat-topped
**18**	Gompertz Type-4	250.89	176.70	188.21	165.19	84	47.54	250 km, pointy-topped
**18**	Gompertz Type-3	185.52	134.09	140.29	127.90	64	47.73	500 km, pointy-topped

**18**	Gompertz Type-4	187.52	133.96	143.18	124.75	64	47.77	500 km, pointy-topped
**18**	Gompertz Type-3	228.17	186.26	194.92	177.61	83	44.56	250 km, flat-topped
**18**	Gompertz Type-3	249.17	172.31	180.08	164.55	84	48.75	250 km, pointy-topped
**18**	Gompertz Type-3	151.28	124.50	NA	NA	54	43.37	750 km, flat-topped
**19**	Logistic Type-4	78.37	61.90	NA	NA	27	43.62	500 km, flat-topped
**19**	Gompertz Type-3	78.69	60.00	NA	NA	27	45.00	500 km, flat-topped
**19**	Logistic Type-4	76.74	58.50	61.32	55.68	27	46.15	750 km, pointy-topped
**19**	Gompertz Type-3	77.00	47.47	56.35	38.60	27	56.87	750 km, pointy-topped
**19**	Gompertz Type-3	76.55	75.09	81.72	68.46	33	43.94	250 km, pointy-topped
**19**	Gompertz Type-3	68.60	38.44	48.57	28.30	24	62.44	750 km, flat-topped
**19**	Logistic Type-4	68.81	50.88	54.00	47.77	24	47.17	750 km, flat-topped
**19**	Gompertz Type-4	69.16	48.54	51.74	45.33	24	49.45	750 km, flat-topped
**19**	Gompertz Type-4	67.89	43.54	53.31	33.77	25	57.42	500 km, pointy-topped
**19**	Gompertz Type-4	79.29	59.87	62.69	57.05	27	45.10	500 km, flat-topped
**19**	Logistic Type-5	79.86	66.52	83.51	49.53	27	40.59	500 km, flat-topped
**19**	Gompertz Type-3	66.84	36.96	47.23	26.68	25	67.64	500 km, pointy-topped
**19**	Gompertz Type-4	82.05	65.22	73.00	57.45	34	52.13	250 km, flat-topped
**19**	Gompertz Type-3	81.27	58.74	66.00	51.48	34	57.88	250 km, flat-topped
**19**	Gompertz Type-4	77.27	56.35	59.83	52.87	27	47.91	750 km, pointy-topped
**19**	Logistic Type-4	82.04	66.25	79.63	52.87	34	51.32	250 km, flat-topped
**19**	Gompertz Type-4	76.95	84.77	86.50	83.04	33	38.93	250 km, pointy-topped
**19**	Logistic Type-4	76.59	79.96	109.87	50.05	33	41.27	250 km, pointy-topped
**19**	Logistic Type-4	67.90	44.20	58.79	29.61	25	56.56	500 km, pointy-topped
**20**	Logistic Type-5	296.13	129.09	137.51	121.00	126	97.61	250 km, flat-topped
**20**	Logistic Type-4	214.19	109.80	119.86	99.74	88	80.15	500 km, flat-topped
**20**	Logistic Type-5	299.53	138.27	147.58	129.16	130	94.02	250 km, pointy-topped
**20**	Logistic Type-5	233.94	100.95	107.57	94.54	98	97.07	500 km, pointy-topped
**20**	Logistic Type-5	214.45	100.61	113.26	88.92	88	87.47	500 km, flat-topped
**20**	Logistic Type-5	146.07	67.74	71.18	64.30	67	98.91	750 km, flat-topped
**20**	Logistic Type-3	213.05	107.26	114.81	99.71	88	82.04	500 km, flat-topped
**20**	Logistic Type-5	160.16	72.21	76.51	68.11	71	98.33	750 km, pointy-topped
**21**	Logistic Type-5	509.95	275.29	296.20	255.35	250	90.81	250 km, flat-topped
**21**	Logistic Type-5	243.74	109.73	114.42	105.05	107	97.51	750 km, flat-topped
**21**	Logistic Type-5	386.71	164.02	172.80	155.37	156	95.11	500 km, flat-topped
**21**	Logistic Type-5	506.56	243.87	254.14	233.64	238	97.59	250 km, pointy-topped
**21**	Logistic Type-5	379.47	164.75	173.67	155.98	156	94.69	500 km, pointy-topped
**21**	Logistic Type-5	284.31	117.63	124.58	110.79	111	94.37	750 km, pointy-topped
**22**	Logistic Type-3	339.72	192.33	208.84	175.82	124	64.47	750 km, flat-topped
**22**	Logistic Type-4	431.84	276.49	307.33	245.64	175	63.29	500 km, pointy-topped
**22**	Logistic Type-5	333.78	192.26	212.67	171.86	128	66.58	750 km, pointy-topped
**22**	Logistic Type-4	332.83	263.45	298.66	228.24	128	48.59	750 km, pointy-topped
**22**	Logistic Type-3	431.06	264.40	283.40	245.40	175	66.19	500 km, pointy-topped
**22**	Logistic Type-5	431.69	259.80	297.97	221.65	175	67.36	500 km, pointy-topped
**22**	Logistic Type-4	599.09	461.65	497.68	425.61	297	64.34	250 km, flat-topped

**22**	Logistic Type-5	605.20	439.79	522.62	361.68	289	65.71	250 km, pointy-topped
**22**	Logistic Type-3	332.07	246.39	276.36	216.42	128	51.95	750 km, pointy-topped
**22**	Logistic Type-4	401.12	299.43	329.49	269.37	166	55.44	500 km, flat-topped
**22**	Logistic Type-4	340.77	203.33	228.72	177.93	124	60.99	750 km, flat-topped
**22**	Logistic Type-5	597.90	432.91	477.63	388.19	297	68.61	250 km, flat-topped
**22**	Logistic Type-5	401.65	270.08	302.18	237.98	166	61.46	500 km, flat-topped
**23**	Logistic Type-5	197.39	68.67	73.86	63.56	67	97.56	750 km, flat-topped
**23**	Logistic Type-3	290.83	156.73	166.89	146.58	122	77.84	250 km, pointy-topped
**23**	Logistic Type-4	292.81	156.35	168.58	144.12	122	78.03	250 km, pointy-topped
**23**	Logistic Type-3	201.49	101.64	110.24	93.05	74	72.80	750 km, pointy-topped
**23**	Logistic Type-5	203.37	90.42	104.38	77.73	74	81.84	750 km, pointy-topped
**23**	Logistic Type-4	203.49	101.81	114.21	89.41	74	72.68	750 km, pointy-topped
**23**	Logistic Type-3	239.33	118.86	127.85	109.87	91	76.56	500 km, flat-topped
**23**	Logistic Type-4	241.20	120.30	132.76	107.85	91	75.64	500 km, flat-topped
**23**	Logistic Type-3	235.17	115.37	123.61	107.13	90	78.01	500 km, pointy-topped
**23**	Logistic Type-3	312.29	166.56	177.48	155.64	129	77.45	250 km, flat-topped
**23**	Logistic Type-4	311.26	173.80	189.09	158.51	129	74.22	250 km, flat-topped
**23**	Logistic Type-4	235.74	111.57	120.99	102.14	90	80.67	500 km, pointy-topped
**23**	Logistic Type-5	234.11	99.52	109.88	89.71	90	90.44	500 km, pointy-topped
**24**	Logistic Type-5	224.11	84.01	88.71	79.36	84	99.99	750 km, flat-topped
**24**	Logistic Type-5	390.70	179.46	192.30	166.80	165	91.94	250 km, pointy-topped
**24**	Logistic Type-5	388.71	164.76	176.51	153.34	155	94.08	250 km, flat-topped
**24**	Logistic Type-5	228.33	78.23	84.55	72.12	78	99.70	750 km, pointy-topped
**24**	Logistic Type-5	292.18	117.75	128.05	107.95	109	92.57	500 km, pointy-topped
**24**	Logistic Type-5	279.49	101.33	108.77	94.09	98	96.71	500 km, flat-topped
**25**	Logistic Type-5	282.55	102.05	107.66	96.47	102	99.95	750 km, flat-topped
**25**	Logistic Type-5	455.39	213.47	222.51	204.44	209	97.91	250 km, flat-topped
**25**	Logistic Type-5	261.42	102.44	106.93	97.96	101	98.59	750 km, pointy-topped
**25**	Logistic Type-5	307.65	137.12	142.16	132.08	136	99.19	500 km, pointy-topped
**25**	Logistic Type-5	296.62	132.98	137.80	128.16	132	99.26	500 km, flat-topped
**25**	Logistic Type-5	452.92	208.22	217.09	199.35	203	97.49	250 km, pointy-topped
**26**	Logistic Type-5	396.86	176.22	185.47	167.02	171	97.04	500 km, pointy-topped
**26**	Logistic Type-5	557.57	266.51	278.52	254.49	269	100.94	250 km, flat-topped
**26**	Logistic Type-5	362.93	156.79	162.14	151.44	158	100.77	500 km, flat-topped
**26**	Logistic Type-5	572.47	280.09	291.53	268.65	282	100.68	250 km, pointy-topped
**26**	Logistic Type-5	280.62	120.93	125.15	116.71	119	98.40	750 km, flat-topped
**26**	Logistic Type-5	281.05	132.72	136.88	128.56	131	98.70	750 km, pointy-topped
**27**	Logistic Type-5	543.74	319.77	335.15	304.40	296	92.57	250 km, flat-topped
**27**	Logistic Type-5	382.20	182.62	189.18	176.06	178	97.47	500 km, pointy-topped
**27**	Logistic Type-5	348.83	127.18	133.87	120.49	128	100.65	750 km, flat-topped
**27**	Logistic Type-5	409.59	178.68	187.27	170.09	173	96.82	500 km, flat-topped
**27**	Logistic Type-5	522.04	311.46	325.12	297.81	291	93.43	250 km, pointy-topped
**27**	Logistic Type-5	364.19	141.97	149.10	134.85	139	97.91	750 km, pointy-topped
**28**	Logistic Type-5	354.55	192.29	203.80	180.80	177	92.05	250 km, flat-topped

**28**	Logistic Type-5	266.51	125.84	136.23	115.74	115	91.38	500 km, pointy-topped
**28**	Logistic Type-3	228.26	124.87	133.71	116.02	92	73.68	750 km, pointy-topped
**28**	Logistic Type-5	253.87	125.30	134.05	116.61	114	90.98	500 km, flat-topped
**28**	Logistic Type-5	409.95	197.78	208.64	186.92	186	94.04	250 km, pointy-topped
**28**	Logistic Type-3	179.81	127.91	137.89	117.93	88	68.80	750 km, flat-topped
**28**	Logistic Type-5	227.74	104.58	116.47	93.87	92	87.97	750 km, pointy-topped
**29**	Gompertz Type-4	158.25	62.41	67.97	56.84	56	89.73	750 km, pointy-topped
**29**	Gompertz Type-3	227.19	95.01	105.99	84.04	83	87.36	250 km, pointy-topped
**29**	Gompertz Type-4	190.27	69.23	78.43	60.04	60	86.66	500 km, pointy-topped
**29**	Gompertz Type-4	228.13	93.59	104.49	82.70	83	88.68	250 km, pointy-topped
**29**	Gompertz Type-4	150.06	56.50	61.45	51.54	53	93.81	750 km, flat-topped
**29**	Gompertz Type-3	195.18	77.05	87.83	66.27	66	85.66	500 km, flat-topped
**29**	Gompertz Type-3	190.72	71.63	81.51	61.75	60	83.76	500 km, pointy-topped
**29**	Gompertz Type-4	239.04	92.40	103.22	81.57	85	92.00	250 km, flat-topped
**29**	Gompertz Type-4	196.48	75.96	86.62	65.30	66	86.89	500 km, flat-topped
**29**	Gompertz Type-3	238.88	94.62	105.81	83.43	85	89.83	250 km, flat-topped
**30**	Logistic Type-5	108.59	52.66	56.23	49.11	52	98.75	750 km, pointy-topped
**30**	Logistic Type-5	204.08	93.95	102.62	85.32	82	87.28	250 km, flat-topped
**30**	Logistic Type-4	106.97	53.12	56.14	50.09	52	97.90	750 km, pointy-topped
**30**	Logistic Type-4	139.71	63.70	68.23	59.16	61	95.77	500 km, pointy-topped
**30**	Logistic Type-4	122.70	49.02	53.49	44.55	48	97.92	750 km, flat-topped
**30**	Logistic Type-4	205.40	90.78	96.83	84.74	86	94.73	250 km, pointy-topped
**30**	Logistic Type-4	154.66	64.80	69.35	60.24	63	97.23	500 km, flat-topped
**30**	Gompertz Type-4	203.37	100.03	108.51	91.56	82	81.97	250 km, flat-topped
**31**	Logistic Type-5	243.04	118.36	123.97	112.75	116	98.01	250 km, pointy-topped
**31**	Logistic Type-5	228.07	110.20	117.50	102.94	105	95.28	250 km, flat-topped
**31**	Logistic Type-4	157.95	71.37	76.26	66.47	65	91.08	750 km, pointy-topped
**31**	Logistic Type-5	157.59	68.79	74.26	63.40	65	94.49	750 km, pointy-topped
**31**	Logistic Type-4	229.37	114.31	120.74	107.89	105	91.85	250 km, flat-topped
**31**	Logistic Type-5	145.55	68.42	72.56	64.31	66	96.46	750 km, flat-topped
**31**	Logistic Type-4	190.29	89.21	93.65	84.76	83	93.04	500 km, pointy-topped
**31**	Logistic Type-5	189.47	86.75	92.22	81.32	83	95.67	500 km, pointy-topped
**31**	Logistic Type-4	165.53	89.03	93.69	84.38	81	90.98	500 km, flat-topped
**31**	Logistic Type-5	166.51	87.77	94.88	80.90	81	92.28	500 km, flat-topped
**32**	Logistic Type-4	218.61	102.11	108.28	95.93	94	92.06	250 km, pointy-topped
**32**	Logistic Type-5	151.11	69.85	75.92	63.87	64	91.62	750 km, flat-topped
**32**	Logistic Type-4	150.09	67.88	72.10	63.66	64	94.29	750 km, flat-topped
**32**	Logistic Type-4	232.02	105.29	112.03	98.55	98	93.08	250 km, flat-topped
**32**	Logistic Type-4	148.60	66.81	71.86	61.76	64	95.79	750 km, pointy-topped
**32**	Logistic Type-4	175.66	81.74	87.29	76.18	76	92.98	500 km, pointy-topped
**32**	Logistic Type-5	150.48	67.58	73.96	61.28	64	94.70	750 km, pointy-topped
**32**	Logistic Type-4	154.57	75.87	80.35	71.39	71	93.58	500 km, flat-topped
**33**	Logistic Type-5	235.04	72.34	78.82	65.88	72	99.53	750 km, pointy-topped
**33**	Logistic Type-4	380.72	147.16	155.54	138.78	141	95.81	250 km, pointy-topped

**33**	Logistic Type-5	381.53	150.62	160.81	140.43	141	93.61	250 km, pointy-topped
**33**	Logistic Type-5	243.16	76.03	81.94	70.13	75	98.64	750 km, flat-topped
**33**	Logistic Type-4	233.08	72.43	78.39	66.47	72	99.41	750 km, pointy-topped
**33**	Logistic Type-4	304.56	96.83	104.78	88.87	94	97.08	500 km, flat-topped
**33**	Logistic Type-4	241.18	75.67	81.00	70.34	75	99.12	750 km, flat-topped
**33**	Logistic Type-5	387.56	151.19	162.90	139.48	141	93.26	250 km, flat-topped
**33**	Logistic Type-4	385.99	148.43	157.77	139.09	141	95.00	250 km, flat-topped
**33**	Logistic Type-4	279.72	100.25	106.12	94.39	96	95.76	500 km, pointy-topped
**33**	Logistic Type-5	306.51	97.82	106.91	88.75	94	96.10	500 km, flat-topped
**34**	Logistic Type-5	178.96	72.88	77.63	68.15	72	98.79	750 km, pointy-topped
**34**	Logistic Type-5	165.54	79.23	83.50	74.98	77	97.18	750 km, flat-topped
**34**	Logistic Type-5	211.18	94.75	99.76	89.74	91	96.04	500 km, pointy-topped
**34**	Logistic Type-5	282.46	134.26	139.69	128.84	134	99.81	250 km, pointy-topped
**34**	Logistic Type-5	293.96	136.81	143.03	130.60	136	99.40	250 km, flat-topped
**34**	Logistic Type-5	197.13	100.66	105.14	96.19	97	96.36	500 km, flat-topped
**35**	Logistic Type-5	238.76	85.73	90.53	80.93	86	100.32	750 km, pointy-topped
**35**	Logistic Type-5	332.59	162.11	172.46	151.83	154	95.00	250 km, pointy-topped
**35**	Logistic Type-5	203.63	80.51	84.95	76.09	80	99.36	750 km, flat-topped
**35**	Logistic Type-5	254.34	107.05	111.65	102.45	107	99.95	500 km, flat-topped
**35**	Logistic Type-5	330.23	151.71	160.54	142.90	146	96.24	250 km, flat-topped
**35**	Logistic Type-5	266.24	109.63	115.25	104.01	110	100.34	500 km, pointy-topped
**36**	Logistic Type-4	372.14	159.77	170.48	149.07	154	96.39	250 km, flat-topped
**36**	Logistic Type-4	212.06	74.63	80.32	68.94	75	100.49	750 km, pointy-topped
**36**	Logistic Type-5	360.34	161.37	172.91	149.83	148	91.72	250 km, pointy-topped
**36**	Gompertz Type-4	371.49	171.71	184.43	158.99	154	89.69	250 km, flat-topped
**36**	Logistic Type-4	235.02	77.77	83.94	71.60	77	99.01	750 km, flat-topped
**36**	Logistic Type-5	237.00	77.54	84.22	70.87	77	99.30	750 km, flat-topped
**36**	Logistic Type-5	212.78	73.61	79.45	67.77	75	101.89	750 km, pointy-topped
**36**	Logistic Type-5	370.29	165.69	178.35	153.03	154	92.94	250 km, flat-topped
**36**	Logistic Type-3	370.58	160.62	171.03	150.22	154	95.88	250 km, flat-topped
**36**	Gompertz Type-4	359.96	166.45	178.58	154.32	148	88.92	250 km, pointy-topped
**36**	Logistic Type-4	261.14	102.13	108.12	96.14	99	96.93	500 km, pointy-topped
**36**	Logistic Type-5	263.06	102.69	109.78	95.60	99	96.41	500 km, pointy-topped
**36**	Logistic Type-4	272.43	103.03	110.05	96.02	101	98.03	500 km, flat-topped
**36**	Logistic Type-5	274.34	103.48	111.34	95.61	101	97.61	500 km, flat-topped
**37**	Logistic Type-5	287.75	142.42	149.20	135.67	142	99.71	250 km, flat-topped
**37**	Logistic Type-5	184.58	78.81	83.16	74.47	80	101.51	750 km, flat-topped
**37**	Logistic Type-5	173.18	73.04	77.38	68.71	72	98.58	750 km, pointy-topped
**37**	Logistic Type-5	230.55	96.59	102.70	90.53	94	97.32	500 km, pointy-topped
**37**	Logistic Type-5	301.87	153.83	161.31	146.36	150	97.51	250 km, pointy-topped
**37**	Logistic Type-5	201.64	95.21	99.52	90.91	95	99.78	500 km, flat-topped
**38**	Logistic Type-4	209.26	93.62	98.60	88.63	86	91.86	500 km, pointy-topped
**38**	Logistic Type-5	166.42	69.82	76.04	63.72	66	94.54	750 km, pointy-topped
**38**	Logistic Type-5	302.01	155.52	163.52	147.51	135	86.81	250 km, flat-topped

**38**	Gompertz Type-4	300.05	160.85	168.82	152.89	135	83.93	250 km, flat-topped
**38**	Logistic Type-4	200.46	87.87	92.51	83.22	86	97.88	500 km, flat-topped
**38**	Logistic Type-4	150.28	69.01	73.00	65.02	64	92.74	750 km, flat-topped
**38**	Logistic Type-5	151.09	68.16	72.72	63.61	64	93.90	750 km, flat-topped
**38**	Logistic Type-4	316.23	151.32	159.61	143.04	138	91.20	250 km, pointy-topped
**38**	Logistic Type-4	164.53	69.19	73.80	64.58	66	95.39	750 km, pointy-topped
**38**	Logistic Type-5	318.15	152.28	165.42	139.47	138	90.62	250 km, pointy-topped
**38**	Logistic Type-5	201.51	89.42	95.39	83.46	86	96.18	500 km, flat-topped
**38**	Logistic Type-5	211.06	92.55	99.34	85.85	86	92.92	500 km, pointy-topped
**39**	Logistic Type-4	162.87	90.59	95.06	86.13	83	91.62	250 km, pointy-topped
**39**	Logistic Type-4	153.34	82.00	85.96	78.04	75	91.46	250 km, flat-topped
**39**	Logistic Type-5	102.10	43.02	46.14	39.90	43	99.96	750 km, pointy-topped
**39**	Logistic Type-5	95.48	40.46	44.59	36.52	40	98.87	750 km, flat-topped
**39**	Logistic Type-4	114.26	58.42	62.98	53.86	52	89.01	500 km, pointy-topped
**39**	Logistic Type-5	114.91	55.40	61.21	50.02	52	93.86	500 km, pointy-topped
**39**	Logistic Type-4	97.11	43.49	47.87	39.11	40	91.98	750 km, flat-topped
**39**	Logistic Type-4	104.14	49.72	52.53	46.90	49	98.56	500 km, flat-topped
**40**	Logistic Type-5	299.99	131.42	140.12	122.74	133	101.20	250 km, flat-topped
**40**	Logistic Type-5	309.07	128.09	136.27	119.91	130	101.49	250 km, pointy-topped
**40**	Logistic Type-5	240.35	90.01	108.64	77.01	81	89.99	500 km, pointy-topped
**40**	Logistic Type-4	161.32	82.72	90.94	74.50	65	78.58	750 km, flat-topped
**40**	Logistic Type-5	161.34	77.15	84.89	69.48	65	84.25	750 km, flat-topped
**40**	Logistic Type-5	131.29	66.83	70.46	63.28	66	98.76	750 km, pointy-topped
**40**	Logistic Type-4	239.98	100.48	112.74	88.22	81	80.61	500 km, pointy-topped
**40**	Logistic Type-3	162.89	86.09	94.64	77.53	65	75.51	750 km, flat-topped
**40**	Logistic Type-3	239.90	104.13	116.90	91.36	81	77.79	500 km, pointy-topped
**40**	Logistic Type-5	229.38	86.37	94.14	78.94	86	99.57	500 km, flat-topped
**41**	Logistic Type-5	189.73	88.44	96.16	81.02	83	93.85	750 km, pointy-topped
**41**	Logistic Type-5	234.51	108.34	114.01	102.68	109	100.61	500 km, pointy-topped
**41**	Logistic Type-5	337.15	189.45	197.15	181.76	195	102.93	250 km, flat-topped
**41**	Logistic Type-5	349.61	183.93	193.22	174.63	189	102.76	250 km, pointy-topped
**41**	Logistic Type-4	201.53	88.85	96.17	81.52	80	90.04	750 km, flat-topped
**41**	Logistic Type-4	189.03	92.00	97.94	86.05	83	90.22	750 km, pointy-topped
**41**	Logistic Type-5	203.28	86.72	96.11	77.78	80	92.25	750 km, flat-topped
**41**	Logistic Type-5	247.66	120.57	128.98	112.24	117	97.04	500 km, flat-topped
**41**	Logistic Type-4	247.72	126.22	133.56	118.87	117	92.70	500 km, flat-topped
**42**	Logistic Type-5	288.21	133.75	140.91	126.59	136	101.68	250 km, flat-topped
**42**	Logistic Type-4	149.35	72.85	76.66	69.05	70	96.08	750 km, pointy-topped
**42**	Logistic Type-5	297.18	138.93	146.17	131.69	143	102.93	250 km, pointy-topped
**42**	Logistic Type-5	224.56	98.78	105.17	92.44	97	98.20	500 km, pointy-topped
**42**	Gompertz Type-4	155.85	88.63	97.89	79.38	68	76.72	750 km, flat-topped
**42**	Logistic Type-5	213.71	89.51	94.23	84.79	90	100.55	500 km, flat-topped
**42**	Logistic Type-5	150.58	71.90	76.17	67.64	70	97.35	750 km, pointy-topped
**42**	Logistic Type-4	155.17	75.97	81.63	70.30	68	89.51	750 km, flat-topped

**42**	Logistic Type-5	156.12	78.64	87.63	70.03	68	86.47	750 km, flat-topped
**43**	Logistic Type-5	143.04	65.22	69.07	61.39	65	99.66	250 km, pointy-topped
**43**	Logistic Type-5	108.52	49.55	52.57	46.55	50	100.91	500 km, flat-topped
**43**	Logistic Type-5	83.75	38.09	40.77	35.46	38	99.76	750 km, pointy-topped
**43**	Logistic Type-5	163.21	73.05	76.75	69.35	74	101.30	250 km, flat-topped
**43**	Logistic Type-5	90.38	40.92	43.54	38.37	41	100.19	750 km, flat-topped
**43**	Logistic Type-4	156.25	62.03	73.12	51.08	47	75.77	500 km, pointy-topped
**44**	Logistic Type-4	181.05	103.47	160.80	65.57	64	61.85	750 km, pointy-topped
**44**	Logistic Type-4	204.94	172.08	NA	NA	75	43.58	500 km, flat-topped
**44**	Logistic Type-5	206.68	158.91	NA	NA	75	47.20	500 km, flat-topped
**44**	Logistic Type-5	199.32	106.80	NA	NA	71	66.48	500 km, pointy-topped
**44**	Logistic Type-4	200.91	145.40	263.84	76.71	71	48.83	500 km, pointy-topped
**44**	Logistic Type-4	257.26	195.04	NA	NA	92	47.17	250 km, pointy-topped
**44**	Logistic Type-5	258.10	95.21	103.62	87.01	95	99.77	250 km, flat-topped
**44**	Logistic Type-4	188.16	138.11	NA	NA	63	45.62	750 km, flat-topped
**44**	Logistic Type-5	188.87	121.76	473.34	55.57	63	51.74	750 km, flat-topped
**44**	Logistic Type-5	256.23	156.72	NA	NA	92	58.70	250 km, pointy-topped
**45**	Logistic Type-5	458.44	263.52	277.02	250.03	269	102.08	250 km, flat-topped
**45**	Logistic Type-5	333.02	167.21	177.61	156.82	170	101.67	500 km, flat-topped
**45**	Logistic Type-4	368.11	183.91	197.66	170.16	172	93.52	500 km, pointy-topped
**45**	Logistic Type-5	248.14	121.12	128.59	113.64	123	101.56	750 km, flat-topped
**45**	Logistic Type-5	458.48	273.40	287.17	259.63	279	102.05	250 km, pointy-topped
**45**	Logistic Type-5	264.50	120.41	127.58	113.24	122	101.32	750 km, pointy-topped
**46**	Logistic Type-5	456.52	209.88	225.73	194.02	213	101.49	250 km, pointy-topped
**46**	Logistic Type-5	354.21	135.02	147.36	122.76	136	100.72	500 km, flat-topped
**46**	Logistic Type-4	323.79	231.69	326.23	150.83	128	55.25	500 km, pointy-topped
**46**	Logistic Type-5	324.36	222.68	NA	NA	128	57.48	500 km, pointy-topped
**46**	Logistic Type-5	268.46	96.41	109.52	83.88	94	97.50	750 km, pointy-topped
**46**	Logistic Type-3	322.97	271.26	364.77	179.51	128	47.19	500 km, pointy-topped
**46**	Logistic Type-5	441.86	201.94	215.91	187.96	206	102.01	250 km, flat-topped
**46**	Logistic Type-5	260.15	98.69	107.20	90.21	99	100.32	750 km, flat-topped
**47**	Logistic Type-5	227.76	84.77	90.72	78.81	85	100.28	500 km, flat-topped
**47**	Logistic Type-5	209.87	67.10	71.96	62.25	69	102.83	750 km, pointy-topped
**47**	Logistic Type-5	306.96	125.66	131.59	119.73	128	101.86	250 km, pointy-topped
**47**	Logistic Type-5	304.63	127.90	134.08	121.71	130	101.64	250 km, flat-topped
**47**	Logistic Type-5	175.99	62.18	66.22	58.14	63	101.32	750 km, flat-topped
**47**	Logistic Type-5	214.58	86.81	91.35	82.27	88	101.37	500 km, pointy-topped
**48**	Logistic Type-5	428.48	152.15	164.73	139.58	156	102.53	250 km, flat-topped
**48**	Logistic Type-5	205.31	72.86	78.52	67.21	74	101.57	750 km, pointy-topped
**48**	Logistic Type-5	431.65	151.16	164.76	137.56	154	101.88	250 km, pointy-topped
**48**	Logistic Type-5	279.65	93.60	101.71	85.52	97	103.63	500 km, flat-topped
**48**	Logistic Type-5	207.95	71.39	76.94	65.85	73	102.26	750 km, flat-topped
**48**	Logistic Type-5	271.31	93.29	100.66	85.92	95	101.84	500 km, pointy-topped
**49**	Logistic Type-5	224.46	70.27	77.40	63.52	71	101.04	750 km, pointy-topped

**49**	Logistic Type-4	372.86	156.75	169.98	143.52	142	90.59	250 km, flat-topped
**49**	Logistic Type-5	373.77	139.10	149.75	128.46	140	100.65	250 km, pointy-topped
**49**	Logistic Type-5	222.46	70.38	78.31	63.06	70	99.45	750 km, flat-topped
**49**	Logistic Type-5	269.88	90.77	101.84	80.93	89	98.05	500 km, pointy-topped
**49**	Logistic Type-5	373.41	156.71	173.52	140.35	142	90.61	250 km, flat-topped
**49**	Logistic Type-5	267.96	91.59	100.58	82.85	91	99.35	500 km, flat-topped
**50**	Logistic Type-5	158.50	49.10	52.95	45.26	53	107.94	750 km, pointy-topped
**50**	Logistic Type-5	207.11	68.05	72.86	63.24	70	102.87	500 km, flat-topped
**50**	Logistic Type-5	170.77	56.22	60.59	51.87	56	99.61	750 km, flat-topped
**50**	Logistic Type-5	266.72	102.45	108.74	96.15	106	103.47	250 km, pointy-topped
**50**	Logistic Type-5	274.00	112.26	119.13	105.40	114	101.55	250 km, flat-topped
**50**	Logistic Type-5	216.88	70.50	75.73	65.26	73	103.55	500 km, pointy-topped
**51**	Logistic Type-5	310.89	100.97	110.20	91.78	103	102.01	250 km, flat-topped
**51**	Logistic Type-5	138.80	51.21	55.36	47.13	51	99.60	750 km, pointy-topped
**51**	Logistic Type-4	184.58	71.57	76.46	66.68	70	97.81	500 km, flat-topped
**51**	Logistic Type-5	292.92	100.50	109.54	91.48	102	101.49	250 km, pointy-topped
**51**	Logistic Type-4	137.65	52.40	55.90	48.90	51	97.33	750 km, pointy-topped
**51**	Logistic Type-4	127.60	50.03	53.14	46.91	50	99.95	750 km, flat-topped
**51**	Logistic Type-4	177.55	69.16	74.36	63.95	65	93.99	500 km, pointy-topped
**51**	Logistic Type-5	176.63	65.72	71.35	60.20	65	98.91	500 km, pointy-topped
**51**	Logistic Type-4	291.57	98.76	106.58	90.94	102	103.28	250 km, pointy-topped
**51**	Logistic Type-5	126.43	48.60	51.85	45.36	50	102.88	750 km, flat-topped
**51**	Logistic Type-4	308.89	101.06	109.20	92.91	103	101.92	250 km, flat-topped
**52**	Logistic Type-5	511.65	171.13	185.52	156.74	177	103.43	250 km, flat-topped
**52**	Logistic Type-5	277.77	80.36	88.81	72.01	81	100.80	750 km, flat-topped
**52**	Logistic Type-5	353.44	104.30	112.97	95.67	105	100.67	500 km, flat-topped
**52**	Logistic Type-5	515.31	165.92	180.16	151.68	171	103.06	250 km, pointy-topped
**52**	Logistic Type-5	283.32	81.53	90.01	73.15	82	100.57	750 km, pointy-topped
**52**	Logistic Type-5	325.00	102.05	111.50	92.63	104	101.91	500 km, pointy-topped
**53**	Logistic Type-5	325.86	135.36	144.58	126.14	142	104.91	250 km, flat-topped
**53**	Logistic Type-5	237.81	83.94	90.54	77.34	89	106.03	500 km, flat-topped
**53**	Logistic Type-5	190.93	70.77	75.97	65.59	72	101.74	750 km, pointy-topped
**53**	Logistic Type-4	328.82	139.69	149.10	130.27	142	101.66	250 km, pointy-topped
**53**	Logistic Type-5	328.08	137.33	146.91	127.75	142	103.40	250 km, pointy-topped
**53**	Logistic Type-5	164.78	67.41	71.10	63.73	69	102.36	750 km, flat-topped
**53**	Logistic Type-4	190.64	72.43	77.27	67.58	72	99.41	750 km, pointy-topped
**53**	Logistic Type-5	268.48	91.62	98.76	84.49	95	103.68	500 km, pointy-topped
**54**	Logistic Type-4	370.82	154.36	165.64	143.09	157	101.71	250 km, flat-topped
**54**	Logistic Type-5	384.93	158.47	169.29	147.65	165	104.12	250 km, pointy-topped
**54**	Logistic Type-5	211.79	79.85	85.67	74.04	82	102.69	750 km, flat-topped
**54**	Logistic Type-5	216.82	82.56	89.29	75.86	86	104.16	750 km, pointy-topped
**54**	Logistic Type-5	258.73	103.99	111.14	96.84	107	102.90	500 km, pointy-topped
**54**	Logistic Type-4	295.93	106.85	115.64	98.05	107	100.14	500 km, flat-topped
**55**	Logistic Type-5	309.04	167.44	173.53	161.36	169	100.93	250 km, flat-topped

**55**	Logistic Type-5	209.04	85.65	90.30	81.01	87	101.57	750 km, pointy-topped
**55**	Logistic Type-5	333.33	179.32	185.92	172.72	181	100.94	250 km, pointy-topped
**55**	Logistic Type-5	227.25	116.04	120.91	111.17	116	99.96	500 km, flat-topped
**55**	Logistic Type-5	249.19	115.34	119.70	110.97	113	97.97	500 km, pointy-topped
**55**	Logistic Type-5	195.03	84.68	89.47	79.90	86	101.55	750 km, flat-topped
**56**	Logistic Type-5	450.94	178.35	191.35	165.35	187	104.85	250 km, flat-topped
**56**	Logistic Type-5	234.23	75.54	82.28	68.80	79	104.59	750 km, flat-topped
**56**	Logistic Type-5	313.28	107.64	115.31	99.96	111	103.13	500 km, flat-topped
**56**	Logistic Type-5	226.39	75.11	80.41	69.80	79	105.19	750 km, pointy-topped
**56**	Logistic Type-5	446.63	175.62	188.21	163.03	185	105.34	250 km, pointy-topped
**56**	Logistic Type-5	294.61	102.81	109.33	96.29	107	104.08	500 km, pointy-topped
**57**	Logistic Type-5	371.75	158.98	167.69	150.27	155	97.50	250 km, pointy-topped
**57**	Logistic Type-5	217.83	79.19	85.79	72.73	76	95.97	750 km, flat-topped
**57**	Logistic Type-5	247.99	99.47	106.49	92.82	96	96.51	500 km, flat-topped
**57**	Logistic Type-5	254.87	103.22	110.16	96.35	99	95.91	500 km, pointy-topped
**57**	Logistic Type-5	372.25	152.18	161.67	142.72	148	97.26	250 km, flat-topped
**57**	Logistic Type-5	206.24	81.08	86.36	75.85	80	98.67	750 km, pointy-topped
**58**	Logistic Type-5	368.17	162.26	171.03	153.51	159	97.99	250 km, flat-topped
**58**	Logistic Type-4	367.35	172.65	181.81	163.49	162	93.83	250 km, pointy-topped
**58**	Logistic Type-5	226.03	76.99	83.79	70.21	78	101.31	750 km, pointy-topped
**58**	Logistic Type-5	367.83	168.60	179.62	157.61	162	96.09	250 km, pointy-topped
**58**	Logistic Type-4	224.97	77.93	84.42	71.44	78	100.09	750 km, pointy-topped
**58**	Logistic Type-5	242.98	85.07	91.87	78.34	85	99.92	750 km, flat-topped
**58**	Logistic Type-4	287.83	108.99	116.62	101.37	104	95.42	500 km, flat-topped
**58**	Logistic Type-5	288.61	102.56	108.94	96.19	103	100.43	500 km, pointy-topped
**59**	Logistic Type-5	209.57	71.32	76.23	66.42	73	102.36	750 km, pointy-topped
**59**	Logistic Type-5	270.32	136.17	141.04	131.30	136	99.87	250 km, pointy-topped
**59**	Logistic Type-4	277.64	143.62	148.94	138.31	136	94.69	250 km, flat-topped
**59**	Logistic Type-4	225.93	92.42	97.08	87.76	92	99.55	500 km, flat-topped
**59**	Logistic Type-5	205.84	90.66	96.06	85.28	89	98.17	500 km, pointy-topped
**59**	Logistic Type-5	276.84	140.50	146.66	134.35	136	96.79	250 km, flat-topped
**59**	Logistic Type-5	218.85	78.57	85.41	71.81	77	98.00	750 km, flat-topped
**59**	Gompertz Type-4	218.58	82.32	88.54	76.09	77	93.54	750 km, flat-topped
**59**	Logistic Type-5	227.43	91.69	97.03	86.35	92	100.34	500 km, flat-topped
**59**	Logistic Type-4	218.07	76.54	81.65	71.43	77	100.60	750 km, flat-topped
**60**	Logistic Type-3	104.74	62.93	69.43	56.42	47	74.69	500 km, flat-topped
**60**	Logistic Type-5	103.30	51.38	61.33	42.32	41	79.79	750 km, pointy-topped
**60**	Logistic Type-4	103.40	59.10	65.52	52.68	47	79.53	500 km, flat-topped
**60**	Logistic Type-4	102.47	57.94	75.65	42.58	41	70.76	750 km, pointy-topped
**60**	Logistic Type-3	103.26	60.98	71.16	50.79	41	67.24	750 km, pointy-topped
**60**	Gompertz Type-4	137.57	100.61	109.80	91.42	63	62.62	250 km, flat-topped
**60**	Logistic Type-5	105.11	60.28	70.34	50.72	47	77.97	500 km, flat-topped
**60**	Logistic Type-4	128.20	84.19	91.30	77.09	64	76.02	250 km, pointy-topped
**60**	Gompertz Type-4	103.62	75.28	84.22	66.35	47	62.43	500 km, flat-topped

**60**	Logistic Type-4	101.21	49.35	55.64	43.05	41	83.08	750 km, flat-topped
**60**	Logistic Type-5	107.53	59.06	67.86	51.06	50	84.66	500 km, pointy-topped
**60**	Logistic Type-4	106.28	62.77	69.28	56.26	50	79.66	500 km, pointy-topped
**60**	Gompertz Type-4	101.57	61.80	71.30	52.29	41	66.35	750 km, flat-topped
**60**	Logistic Type-5	102.53	51.53	61.12	42.54	41	79.57	750 km, flat-topped
**61**	Logistic Type-5	64.65	26.65	29.32	24.05	27	101.30	750 km, pointy-topped
**61**	Logistic Type-5	114.95	41.48	46.03	36.97	42	101.24	250 km, flat-topped
**61**	Logistic Type-5	128.13	46.28	49.96	42.60	47	101.56	250 km, pointy-topped
**61**	Logistic Type-5	57.57	24.25	27.32	21.31	24	98.97	750 km, flat-topped
**61**	Logistic Type-5	71.07	31.67	35.48	28.20	31	97.87	500 km, pointy-topped
**61**	Logistic Type-4	56.99	24.86	27.92	21.82	24	96.52	750 km, flat-topped
**61**	Logistic Type-5	68.87	30.52	32.97	28.08	31	101.58	500 km, flat-topped
**62**	Logistic Type-5	242.62	111.10	123.71	98.73	95	85.50	250 km, pointy-topped
**62**	Logistic Type-5	267.53	118.70	136.07	101.92	96	80.87	250 km, flat-topped
**62**	Logistic Type-5	190.06	71.28	82.82	60.91	63	88.38	500 km, pointy-topped
**62**	Logistic Type-3	158.83	84.50	101.10	67.89	55	65.09	750 km, pointy-topped
**62**	Logistic Type-5	124.38	62.15	72.88	51.98	49	78.85	750 km, flat-topped
**62**	Logistic Type-4	265.96	115.05	125.53	104.57	96	83.44	250 km, flat-topped
**62**	Logistic Type-4	188.41	73.88	82.75	65.01	63	85.27	500 km, pointy-topped
**62**	Gompertz Type-4	124.43	75.77	85.19	66.34	49	64.67	750 km, flat-topped
**62**	Logistic Type-3	124.23	65.01	72.22	57.80	49	75.37	750 km, flat-topped
**62**	Logistic Type-4	122.60	61.42	68.59	54.25	49	79.78	750 km, flat-topped
**62**	Logistic Type-4	240.82	109.02	116.79	101.26	95	87.14	250 km, pointy-topped
**62**	Logistic Type-4	185.96	78.66	86.13	71.19	67	85.17	500 km, flat-topped
**62**	Logistic Type-5	186.06	73.68	82.14	65.63	67	90.93	500 km, flat-topped
**63**	Logistic Type-5	320.16	109.94	120.41	99.53	106	96.42	250 km, pointy-topped
**63**	Gompertz Type-4	202.78	62.33	71.53	53.22	59	94.66	750 km, pointy-topped
**63**	Gompertz Type-4	319.70	114.38	124.40	104.37	106	92.67	250 km, pointy-topped
**63**	Logistic Type-4	202.09	61.84	70.83	52.98	59	95.40	750 km, pointy-topped
**63**	Logistic Type-5	222.76	80.86	96.37	67.21	70	86.57	500 km, pointy-topped
**63**	Logistic Type-4	220.98	78.85	90.17	67.61	70	88.78	500 km, pointy-topped
**63**	Logistic Type-5	325.21	110.12	121.97	98.37	106	96.26	250 km, flat-topped
**63**	Logistic Type-5	203.97	61.30	70.59	52.41	59	96.24	750 km, pointy-topped
**63**	Gompertz Type-4	220.88	84.71	99.15	70.45	70	82.64	500 km, pointy-topped
**63**	Logistic Type-5	228.20	70.26	78.19	62.55	68	96.79	500 km, flat-topped
**63**	Logistic Type-4	324.15	112.67	124.00	101.34	106	94.08	250 km, flat-topped
**63**	Gompertz Type-4	175.88	59.80	68.41	51.24	53	88.63	750 km, flat-topped
**63**	Logistic Type-4	318.66	108.32	117.12	99.52	106	97.86	250 km, pointy-topped
**64**	Gompertz Type-4	118.05	50.10	54.77	45.42	48	95.81	750 km, pointy-topped
**64**	Gompertz Type-4	203.35	90.35	97.55	83.15	86	95.18	250 km, pointy-topped
**64**	Gompertz Type-4	130.70	68.64	73.44	63.84	61	88.87	500 km, pointy-topped
**64**	Gompertz Type-3	132.09	70.23	75.12	65.34	61	86.86	500 km, pointy-topped
**64**	Gompertz Type-3	202.07	90.91	98.05	83.77	86	94.60	250 km, pointy-topped
**64**	Gompertz Type-3	225.49	91.42	98.60	84.23	90	98.45	250 km, flat-topped

**64**	Gompertz Type-4	170.80	62.65	69.17	56.14	62	98.96	500 km, flat-topped
**64**	Gompertz Type-4	112.54	47.65	51.45	43.84	47	98.64	750 km, flat-topped
**64**	Gompertz Type-4	223.59	90.29	97.19	83.39	90	99.68	250 km, flat-topped
**64**	Gompertz Type-3	114.23	49.76	53.81	45.71	47	94.46	750 km, flat-topped
**65**	Gompertz Type-4	220.79	80.89	87.38	74.41	75	92.71	750 km, pointy-topped
**65**	Gompertz Type-4	407.58	205.52	221.49	189.54	169	82.23	250 km, flat-topped
**65**	Logistic Type-5	272.88	111.16	120.96	101.49	103	92.66	500 km, flat-topped
**65**	Logistic Type-4	220.93	76.96	82.67	71.25	75	97.46	750 km, pointy-topped
**65**	Logistic Type-5	222.03	77.78	84.30	71.28	75	96.42	750 km, pointy-topped
**65**	Logistic Type-4	407.39	183.52	195.38	171.66	169	92.09	250 km, flat-topped
**65**	Logistic Type-5	261.59	108.82	117.45	100.26	102	93.73	500 km, pointy-topped
**65**	Logistic Type-5	406.57	195.82	210.62	181.02	169	86.30	250 km, pointy-topped
**65**	Logistic Type-4	272.06	108.28	115.36	101.19	103	95.13	500 km, flat-topped
**65**	Gompertz Type-4	404.63	201.14	216.04	186.24	169	84.02	250 km, pointy-topped
**65**	Logistic Type-5	407.36	192.60	210.30	174.99	169	87.75	250 km, flat-topped
**65**	Logistic Type-4	259.77	107.13	113.69	100.58	102	95.21	500 km, pointy-topped
**65**	Gompertz Type-4	235.51	80.78	87.89	73.66	76	94.09	750 km, flat-topped
**65**	Gompertz Type-4	273.64	116.87	125.76	107.99	103	88.13	500 km, flat-topped
**66**	Logistic Type-5	204.55	72.78	82.33	64.53	68	93.43	750 km, pointy-topped
**66**	Logistic Type-5	274.64	103.37	116.51	91.12	94	90.93	500 km, pointy-topped
**66**	Logistic Type-5	410.57	156.38	170.51	142.50	149	95.28	250 km, pointy-topped
**66**	Logistic Type-4	272.91	106.28	116.19	96.37	94	88.45	500 km, pointy-topped
**66**	Logistic Type-4	410.03	160.78	172.24	149.32	149	92.68	250 km, pointy-topped
**66**	Logistic Type-4	204.60	81.19	94.12	68.30	65	80.05	750 km, flat-topped
**66**	Logistic Type-5	204.71	69.03	83.55	59.11	65	94.17	750 km, flat-topped
**66**	Logistic Type-4	206.16	82.08	91.89	72.28	68	82.84	750 km, pointy-topped
**66**	Logistic Type-4	280.25	106.23	115.88	96.58	94	88.49	500 km, flat-topped
**66**	Logistic Type-5	282.17	104.57	118.92	91.43	94	89.89	500 km, flat-topped
**66**	Logistic Type-4	389.28	178.29	192.54	164.04	154	86.37	250 km, flat-topped
**66**	Logistic Type-5	390.18	167.12	188.21	148.38	154	92.15	250 km, flat-topped
**67**	Gompertz Type-4	164.45	82.35	89.98	74.72	70	85.00	750 km, pointy-topped
**67**	Logistic Type-5	165.10	77.00	84.87	69.27	70	90.91	750 km, pointy-topped
**67**	Logistic Type-4	163.00	73.59	78.36	68.82	69	93.76	750 km, flat-topped
**67**	Logistic Type-4	164.17	74.90	80.59	69.22	70	93.45	750 km, pointy-topped
**67**	Logistic Type-4	307.85	159.47	166.84	152.11	147	92.18	250 km, pointy-topped
**67**	Logistic Type-5	291.23	167.54	177.21	157.91	158	94.31	250 km, flat-topped
**67**	Logistic Type-4	205.90	94.37	99.62	89.11	87	92.19	500 km, flat-topped
**67**	Gompertz Type-4	181.56	111.37	119.88	102.86	91	81.71	500 km, pointy-topped
**67**	Logistic Type-5	183.03	102.53	111.68	93.45	91	88.76	500 km, pointy-topped
**67**	Logistic Type-4	291.11	172.54	179.94	165.14	158	91.57	250 km, flat-topped
**67**	Logistic Type-5	207.83	95.35	103.61	87.27	87	91.24	500 km, flat-topped
**67**	Logistic Type-5	164.99	73.96	81.08	67.11	69	93.29	750 km, flat-topped
**67**	Logistic Type-5	306.78	154.24	163.05	145.44	147	95.31	250 km, pointy-topped
**68**	Gompertz Type-4	501.46	242.73	261.48	223.98	202	83.22	250 km, flat-topped

**68**	Gompertz Type-4	457.09	245.59	263.24	227.94	205	83.47	250 km, pointy-topped
**68**	Gompertz Type-4	280.33	111.92	122.47	101.37	98	87.56	750 km, pointy-topped
**68**	Gompertz Type-4	349.50	136.37	147.54	125.20	121	88.73	500 km, pointy-topped
**68**	Logistic Type-4	281.38	103.34	111.86	94.82	98	94.83	750 km, pointy-topped
**68**	Logistic Type-5	281.46	107.76	119.41	96.28	98	90.95	750 km, pointy-topped
**68**	Gompertz Type-4	339.21	138.41	147.71	129.11	125	90.31	500 km, flat-topped
**68**	Gompertz Type-4	272.13	102.56	110.24	94.89	94	91.65	750 km, flat-topped
**69**	Logistic Type-5	410.88	168.81	180.41	157.23	164	97.15	250 km, flat-topped
**69**	Logistic Type-5	377.92	162.42	173.90	150.96	159	97.89	250 km, pointy-topped
**69**	Logistic Type-5	298.52	125.98	139.25	113.13	117	92.87	500 km, pointy-topped
**69**	Logistic Type-5	226.85	86.65	94.10	79.32	85	98.09	750 km, pointy-topped
**69**	Logistic Type-4	296.58	124.80	134.17	115.43	117	93.75	500 km, pointy-topped
**69**	Logistic Type-4	225.18	85.56	91.36	79.77	85	99.34	750 km, pointy-topped
**69**	Gompertz Type-4	222.88	93.52	100.93	86.11	81	86.61	750 km, flat-topped
**69**	Logistic Type-4	286.11	116.28	124.71	107.86	110	94.60	500 km, flat-topped
**70**	Logistic Type-5	254.47	115.06	121.13	108.99	117	101.68	250 km, flat-topped
**70**	Logistic Type-5	177.77	75.53	79.87	71.18	77	101.95	500 km, pointy-topped
**70**	Logistic Type-5	248.35	108.73	114.83	102.63	111	102.09	250 km, pointy-topped
**70**	Logistic Type-5	138.28	59.26	62.60	55.92	61	102.93	750 km, flat-topped
**70**	Logistic Type-5	160.08	63.11	66.94	59.28	65	103.00	750 km, pointy-topped
**70**	Logistic Type-5	186.23	78.52	82.66	74.38	81	103.16	500 km, flat-topped
**71**	Logistic Type-5	373.02	187.95	198.50	177.40	189	100.56	250 km, pointy-topped
**71**	Logistic Type-4	371.12	187.39	196.94	177.84	189	100.86	250 km, pointy-topped
**71**	Logistic Type-4	286.18	126.46	132.70	120.23	126	99.63	500 km, pointy-topped
**71**	Logistic Type-5	261.56	98.30	105.69	90.96	98	99.70	750 km, flat-topped
**71**	Logistic Type-5	413.05	201.35	212.53	190.18	202	100.32	250 km, flat-topped
**71**	Logistic Type-5	286.85	125.09	131.70	118.48	126	100.73	500 km, pointy-topped
**71**	Logistic Type-4	261.09	96.71	102.86	90.55	98	101.34	750 km, flat-topped
**71**	Logistic Type-4	230.30	92.96	98.86	87.06	94	101.12	750 km, pointy-topped
**71**	Gompertz Type-4	262.22	100.98	107.62	94.35	98	97.05	750 km, flat-topped
**71**	Logistic Type-4	414.19	197.91	207.95	187.86	202	102.07	250 km, flat-topped
**71**	Logistic Type-4	295.45	120.64	128.02	113.25	122	101.13	500 km, flat-topped
**71**	Logistic Type-5	232.28	93.32	100.16	86.50	94	100.73	750 km, pointy-topped
**71**	Logistic Type-5	297.17	121.56	130.15	112.99	122	100.37	500 km, flat-topped
**72**	Logistic Type-5	140.28	49.93	54.06	45.87	50	100.14	750 km, flat-topped
**72**	Logistic Type-4	150.41	55.68	58.97	52.38	56	100.58	500 km, pointy-topped
**72**	Logistic Type-5	151.89	55.19	58.84	51.54	56	101.48	500 km, pointy-topped
**72**	Logistic Type-4	194.75	73.26	77.37	69.15	74	101.01	250 km, pointy-topped
**72**	Logistic Type-4	131.93	51.23	54.74	47.72	51	99.55	750 km, pointy-topped
**72**	Logistic Type-5	133.15	50.68	54.42	46.95	51	100.62	750 km, pointy-topped
**72**	Logistic Type-4	140.98	51.78	55.73	47.84	50	96.56	750 km, flat-topped
**72**	Logistic Type-5	196.58	73.70	78.50	68.90	74	100.41	250 km, pointy-topped
**72**	Gompertz Type-4	202.16	85.78	90.62	80.94	81	94.43	250 km, flat-topped
**72**	Logistic Type-5	203.38	83.93	90.46	77.55	81	96.51	250 km, flat-topped

**72**	Logistic Type-5	128.93	53.21	56.43	49.99	54	101.49	500 km, flat-topped
**72**	Logistic Type-4	129.35	53.86	57.03	50.69	54	100.26	500 km, flat-topped
**73**	Gompertz Type-4	200.29	102.91	110.05	95.76	95	92.32	500 km, pointy-topped
**73**	Logistic Type-4	146.12	69.93	73.34	66.52	70	100.10	750 km, flat-topped
**73**	Gompertz Type-4	256.33	132.19	141.30	123.08	127	96.08	250 km, pointy-topped
**73**	Gompertz Type-4	256.23	133.78	141.53	126.03	126	94.19	250 km, flat-topped
**73**	Gompertz Type-4	165.69	74.83	80.24	69.41	75	100.23	750 km, pointy-topped
**73**	Logistic Type-4	181.56	93.43	99.09	87.77	92	98.47	500 km, flat-topped
**73**	Logistic Type-5	183.14	94.18	100.97	87.43	92	97.69	500 km, flat-topped
**73**	Logistic Type-5	201.44	98.86	105.81	91.91	95	96.10	500 km, pointy-topped
**74**	Logistic Type-5	228.76	120.49	132.98	108.17	116	96.27	250 km, pointy-topped
**74**	Logistic Type-5	174.47	77.47	84.32	70.61	78	100.69	500 km, flat-topped
**74**	Logistic Type-4	216.87	110.18	118.67	101.70	109	98.93	250 km, flat-topped
**74**	Logistic Type-5	144.84	63.04	69.34	56.82	63	99.93	750 km, flat-topped
**74**	Logistic Type-3	228.45	116.81	126.82	106.80	116	99.31	250 km, pointy-topped
**74**	Logistic Type-3	215.93	110.84	119.27	102.41	109	98.34	250 km, flat-topped
**74**	Logistic Type-4	167.53	82.56	89.63	75.49	82	99.32	500 km, pointy-topped
**74**	Logistic Type-5	169.45	82.85	90.70	75.02	82	98.98	500 km, pointy-topped
**74**	Gompertz Type-4	227.90	123.53	134.93	112.12	116	93.91	250 km, pointy-topped
**74**	Logistic Type-3	167.51	83.55	90.66	76.43	82	98.15	500 km, pointy-topped
**74**	Logistic Type-5	114.49	55.51	61.27	49.88	56	100.88	750 km, pointy-topped
**74**	Logistic Type-4	172.91	77.99	84.66	71.31	78	100.02	500 km, flat-topped
**74**	Logistic Type-4	145.23	65.37	71.68	59.05	63	96.38	750 km, flat-topped
**75**	Logistic Type-5	239.31	115.21	121.55	108.87	111	96.35	250 km, flat-topped
**75**	Logistic Type-5	204.86	80.22	85.60	74.83	79	98.48	500 km, pointy-topped
**75**	Logistic Type-5	145.33	58.13	62.90	53.45	57	98.05	750 km, pointy-topped
**75**	Logistic Type-5	133.41	55.98	60.23	51.75	55	98.25	750 km, flat-topped
**75**	Logistic Type-4	160.78	72.63	76.40	68.85	72	99.14	500 km, flat-topped
**75**	Logistic Type-5	160.38	71.42	75.31	67.54	72	100.81	500 km, flat-topped
**75**	Logistic Type-4	132.21	56.80	60.63	52.98	55	96.83	750 km, flat-topped
**75**	Logistic Type-4	239.41	117.89	123.65	112.12	111	94.16	250 km, flat-topped
**75**	Logistic Type-4	229.99	114.08	120.62	107.54	107	93.79	250 km, pointy-topped
**75**	Logistic Type-5	231.68	115.04	123.67	106.46	107	93.01	250 km, pointy-topped
**75**	Logistic Type-4	206.04	82.75	88.03	77.47	79	95.47	500 km, pointy-topped
**76**	Logistic Type-4	58.19	25.83	28.84	22.83	24	92.92	750 km, flat-topped
**76**	Logistic Type-4	67.04	23.87	26.47	21.26	25	104.76	750 km, pointy-topped
**76**	Logistic Type-5	78.98	37.73	42.21	33.71	35	92.77	250 km, pointy-topped
**76**	Gompertz Type-4	67.17	24.89	27.76	22.01	25	100.45	750 km, pointy-topped
**76**	Logistic Type-4	76.98	37.76	40.37	35.15	35	92.69	250 km, pointy-topped
**76**	Gompertz Type-4	76.95	36.48	41.95	31.03	31	84.98	500 km, flat-topped
**76**	Logistic Type-4	77.43	32.93	37.03	28.86	31	94.14	500 km, flat-topped
**76**	Logistic Type-5	68.84	24.24	28.20	21.03	25	103.15	750 km, pointy-topped
**76**	Gompertz Type-4	73.59	33.66	38.28	29.06	29	86.14	500 km, pointy-topped
**76**	Gompertz Type-4	78.69	44.56	49.96	39.15	35	78.55	250 km, flat-topped

**76**	Logistic Type-5	60.16	25.57	29.96	21.94	24	93.86	750 km, flat-topped
**77**	Gompertz Type-4	222.43	98.30	108.79	87.81	95	96.64	500 km, flat-topped
**77**	Gompertz Type-4	162.90	71.08	78.74	63.41	70	98.49	750 km, pointy-topped
**77**	Gompertz Type-4	229.04	106.11	119.43	92.81	95	89.53	500 km, pointy-topped
**77**	Logistic Type-4	275.80	131.10	142.09	120.11	125	95.35	250 km, pointy-topped
**77**	Logistic Type-5	193.85	73.32	80.99	65.69	73	99.56	750 km, flat-topped
**77**	Logistic Type-4	191.90	73.18	80.15	66.20	73	99.76	750 km, flat-topped
**77**	Logistic Type-5	277.51	132.36	145.34	119.48	125	94.44	250 km, pointy-topped
**77**	Logistic Type-5	231.00	100.72	113.57	88.11	95	94.32	500 km, pointy-topped
**77**	Gompertz Type-4	280.28	128.16	139.41	116.90	119	92.86	250 km, flat-topped
**78**	Logistic Type-5	70.17	24.51	30.38	20.07	24	97.90	750 km, flat-topped
**78**	Logistic Type-4	102.42	38.43	42.62	34.23	38	98.89	250 km, flat-topped
**78**	Logistic Type-5	102.08	37.48	41.56	33.45	38	101.38	250 km, flat-topped
**78**	Logistic Type-4	64.76	23.81	28.51	19.53	23	96.60	750 km, pointy-topped
**78**	Logistic Type-4	72.24	34.55	62.36	22.02	26	75.24	500 km, pointy-topped
**78**	Gompertz Type-4	73.74	27.15	30.99	23.36	26	95.77	500 km, flat-topped
**78**	Logistic Type-4	68.69	26.15	31.95	20.98	24	91.77	750 km, flat-topped
**78**	Gompertz Type-4	66.49	26.08	33.47	20.01	23	88.20	750 km, pointy-topped
**78**	Logistic Type-5	66.54	23.27	28.62	19.09	23	98.85	750 km, pointy-topped
**78**	Logistic Type-4	74.20	25.84	29.10	22.59	26	100.62	500 km, flat-topped
**78**	Logistic Type-5	75.60	26.56	31.92	22.54	26	97.90	500 km, flat-topped
**78**	Gompertz Type-4	105.91	39.92	45.40	34.49	38	95.20	250 km, pointy-topped
**79**	Logistic Type-4	199.40	82.80	88.30	77.31	79	95.41	500 km, pointy-topped
**79**	Gompertz Type-4	179.31	66.77	71.66	61.88	65	97.35	750 km, pointy-topped
**79**	Logistic Type-4	179.50	64.24	68.79	59.69	65	101.18	750 km, pointy-topped
**79**	Logistic Type-5	279.28	118.95	125.95	111.96	116	97.52	250 km, flat-topped
**79**	Logistic Type-5	180.79	65.26	70.85	59.78	65	99.60	750 km, pointy-topped
**79**	Logistic Type-5	271.94	124.59	132.42	116.77	120	96.32	250 km, pointy-topped
**79**	Logistic Type-4	273.90	129.58	136.89	122.28	120	92.60	250 km, pointy-topped
**79**	Logistic Type-5	199.99	81.08	86.93	75.25	79	97.43	500 km, pointy-topped
**79**	Logistic Type-4	207.32	82.95	88.96	76.95	79	95.23	500 km, flat-topped
**79**	Logistic Type-5	188.73	61.41	66.47	56.34	63	102.60	750 km, flat-topped
**79**	Logistic Type-5	209.17	82.55	89.64	75.51	79	95.70	500 km, flat-topped
**80**	Logistic Type-5	139.32	57.23	61.24	53.24	57	99.60	250 km, flat-topped
**80**	Gompertz Type-4	121.26	52.28	57.45	47.12	47	89.89	500 km, pointy-topped
**80**	Logistic Type-4	137.86	61.02	64.62	57.41	58	95.06	250 km, pointy-topped
**80**	Logistic Type-4	138.26	58.30	61.91	54.69	57	97.77	250 km, flat-topped
**80**	Logistic Type-5	96.46	37.52	40.60	34.48	38	101.28	750 km, pointy-topped
**80**	Logistic Type-5	135.89	58.31	62.11	54.54	58	99.47	250 km, pointy-topped
**80**	Gompertz Type-4	94.03	40.89	44.06	37.71	39	95.39	750 km, flat-topped
**80**	Logistic Type-4	95.91	38.40	41.25	35.54	38	98.97	750 km, pointy-topped
**80**	Logistic Type-4	108.52	46.90	50.07	43.73	46	98.09	500 km, flat-topped
**80**	Logistic Type-5	110.26	46.25	50.06	42.52	46	99.46	500 km, flat-topped
**81**	Logistic Type-5	158.03	81.39	85.68	77.10	77	94.61	250 km, pointy-topped

**81**	Logistic Type-5	141.42	62.24	66.98	57.53	57	91.58	500 km, pointy-topped
**81**	Logistic Type-4	139.81	61.47	64.82	58.13	57	92.72	500 km, pointy-topped
**81**	Gompertz Type-4	133.66	60.60	67.12	54.07	49	80.86	750 km, pointy-topped
**81**	Logistic Type-4	156.80	82.57	85.88	79.25	77	93.26	250 km, pointy-topped
**81**	Logistic Type-4	128.34	58.10	62.52	53.69	52	89.49	750 km, flat-topped
**81**	Logistic Type-5	126.75	54.24	58.79	49.85	52	95.88	750 km, flat-topped
**81**	Logistic Type-4	164.97	85.50	89.03	81.96	78	91.23	250 km, flat-topped
**81**	Gompertz Type-4	137.32	68.98	73.70	64.25	57	82.64	500 km, flat-topped
**81**	Logistic Type-5	164.37	82.63	86.95	78.32	78	94.40	250 km, flat-topped
**82**	Logistic Type-5	177.67	71.47	82.32	61.65	66	92.34	500 km, pointy-topped
**82**	Logistic Type-5	132.28	53.31	59.19	47.79	52	97.54	750 km, pointy-topped
**82**	Logistic Type-4	213.94	146.16	176.66	115.65	87	59.52	250 km, pointy-topped
**82**	Logistic Type-4	145.94	71.01	83.78	58.49	56	78.86	750 km, flat-topped
**82**	Logistic Type-4	175.98	73.73	82.57	64.92	66	89.51	500 km, pointy-topped
**82**	Logistic Type-5	147.33	66.18	80.30	54.33	56	84.62	750 km, flat-topped
**82**	Logistic Type-5	175.73	69.54	78.96	60.97	65	93.47	500 km, flat-topped
**82**	Logistic Type-5	199.64	93.28	102.58	84.72	86	92.20	250 km, flat-topped
**82**	Logistic Type-4	177.19	76.82	86.98	66.68	65	84.62	500 km, flat-topped
**83**	Logistic Type-5	769.42	298.08	318.28	277.89	308	103.33	250 km, pointy-topped
**83**	Logistic Type-5	370.76	113.67	121.35	105.99	118	103.81	750 km, flat-topped
**83**	Logistic Type-5	539.48	166.93	178.52	155.34	172	103.04	500 km, flat-topped
**83**	Logistic Type-5	539.29	167.95	179.36	156.54	176	104.79	500 km, pointy-topped
**83**	Logistic Type-5	732.52	297.07	314.36	279.77	304	102.33	250 km, flat-topped
**83**	Logistic Type-5	421.37	116.26	125.96	106.55	122	104.94	750 km, pointy-topped
**84**	Logistic Type-5	383.63	123.50	133.51	113.49	131	106.07	500 km, pointy-topped
**84**	Logistic Type-5	313.15	90.68	98.23	83.13	96	105.87	750 km, pointy-topped
**84**	Logistic Type-5	306.54	91.49	99.13	83.85	96	104.93	750 km, flat-topped
**84**	Logistic Type-5	572.77	203.97	217.26	190.67	216	105.90	250 km, pointy-topped
**84**	Logistic Type-5	566.18	209.84	223.47	196.21	217	103.41	250 km, flat-topped
**84**	Logistic Type-5	390.41	124.87	135.14	114.59	131	104.91	500 km, flat-topped
**85**	Logistic Type-5	456.55	138.17	149.17	127.17	146	105.67	500 km, pointy-topped
**85**	Logistic Type-5	326.01	95.02	103.44	86.60	100	105.24	750 km, pointy-topped
**85**	Logistic Type-5	404.81	133.90	145.26	122.54	140	104.55	500 km, flat-topped
**85**	Logistic Type-5	544.73	244.13	259.29	228.98	256	104.86	250 km, pointy-topped
**85**	Logistic Type-5	317.87	92.78	100.30	85.26	98	105.63	750 km, flat-topped
**85**	Logistic Type-5	558.15	237.42	253.98	220.87	248	104.45	250 km, flat-topped
**86**	Gompertz Type-3	139.79	110.90	122.87	98.92	63	56.81	250 km, pointy-topped
**86**	Gompertz Type-4	137.96	103.76	115.70	91.83	63	60.72	250 km, pointy-topped
**86**	Logistic Type-3	138.65	80.08	86.35	73.81	63	78.67	250 km, pointy-topped
**86**	Logistic Type-3	124.17	68.38	77.48	59.27	49	71.66	500 km, pointy-topped
**86**	Gompertz Type-4	115.43	103.54	112.33	94.75	62	59.88	250 km, flat-topped
**86**	Logistic Type-4	115.66	80.48	86.00	74.96	62	77.04	250 km, flat-topped
**86**	Logistic Type-5	88.28	48.64	57.65	40.18	37	76.07	750 km, flat-topped
**86**	Logistic Type-4	123.28	64.24	73.00	55.48	49	76.28	500 km, pointy-topped

**86**	Logistic Type-5	86.28	42.43	51.79	34.98	35	82.49	750 km, pointy-topped
**86**	Gompertz Type-4	114.46	78.78	90.85	66.72	45	57.12	500 km, flat-topped
**86**	Logistic Type-4	84.61	45.21	51.53	38.89	35	77.41	750 km, pointy-topped
**86**	Logistic Type-3	86.18	48.76	55.64	41.88	35	71.78	750 km, pointy-topped
**86**	Logistic Type-4	86.81	46.49	52.23	40.76	37	79.58	750 km, flat-topped
**86**	Logistic Type-3	114.17	81.66	86.51	76.81	62	75.93	250 km, flat-topped
**86**	Logistic Type-3	114.72	60.92	68.90	52.95	45	73.86	500 km, flat-topped
**86**	Gompertz Type-4	87.46	58.78	68.87	48.69	37	62.94	750 km, flat-topped
**86**	Gompertz Type-3	114.04	82.74	94.57	70.91	45	54.39	500 km, flat-topped
**86**	Logistic Type-3	86.57	48.41	54.10	42.72	37	76.44	750 km, flat-topped
**87**	Logistic Type-5	474.10	190.35	201.55	179.14	198	104.02	250 km, pointy-topped
**87**	Logistic Type-5	288.09	88.32	94.29	82.35	91	103.04	750 km, pointy-topped
**87**	Logistic Type-5	268.90	89.09	93.82	84.36	93	104.39	750 km, flat-topped
**87**	Logistic Type-5	450.52	196.29	206.58	186.00	202	102.91	250 km, flat-topped
**87**	Logistic Type-5	306.92	111.35	117.25	105.45	115	103.28	500 km, flat-topped
**87**	Logistic Type-5	333.74	116.94	123.07	110.82	120	102.61	500 km, pointy-topped
**88**	Logistic Type-4	436.03	211.32	223.60	199.04	193	91.33	250 km, pointy-topped
**88**	Logistic Type-3	435.30	213.08	225.13	201.04	193	90.57	250 km, pointy-topped
**88**	Logistic Type-5	226.38	85.82	91.52	80.16	84	97.88	750 km, pointy-topped
**88**	Logistic Type-5	303.49	126.03	135.56	116.62	117	92.84	500 km, pointy-topped
**88**	Logistic Type-4	302.70	128.44	134.98	121.89	117	91.09	500 km, pointy-topped
**88**	Logistic Type-3	419.66	206.80	216.73	196.87	191	92.36	250 km, flat-topped
**88**	Logistic Type-5	246.36	91.57	97.21	85.96	91	99.38	750 km, flat-topped
**88**	Logistic Type-4	329.52	133.56	141.58	125.53	124	92.84	500 km, flat-topped
**88**	Logistic Type-4	418.58	204.84	214.79	194.90	191	93.24	250 km, flat-topped
**89**	Logistic Type-5	161.98	65.81	73.60	58.53	64	97.25	500 km, pointy-topped
**89**	Logistic Type-5	231.57	91.41	99.17	83.71	91	99.56	250 km, pointy-topped
**89**	Logistic Type-5	229.71	87.79	96.04	79.59	88	100.24	250 km, flat-topped
**89**	Logistic Type-4	120.17	59.13	65.75	52.51	50	84.56	750 km, pointy-topped
**89**	Logistic Type-5	117.46	51.89	56.11	47.84	51	98.29	750 km, flat-topped
**89**	Logistic Type-5	120.23	54.47	62.34	47.63	50	91.80	750 km, pointy-topped
**89**	Logistic Type-5	152.45	64.19	70.54	58.22	62	96.58	500 km, flat-topped
**90**	Gompertz Type-4	83.20	32.55	36.17	28.92	31	95.24	750 km, pointy-topped
**90**	Gompertz Type-4	125.75	43.17	47.47	38.86	41	94.98	500 km, pointy-topped
**90**	Gompertz Type-3	127.03	44.47	48.96	39.99	41	92.20	500 km, pointy-topped
**90**	Gompertz Type-4	83.10	35.95	39.54	32.35	33	91.80	750 km, flat-topped
**90**	Gompertz Type-3	84.57	33.58	37.52	29.65	31	92.31	750 km, pointy-topped
**90**	Gompertz Type-4	146.64	52.67	56.72	48.62	52	98.73	250 km, pointy-topped
**90**	Gompertz Type-3	83.06	36.79	40.39	33.18	33	89.71	750 km, flat-topped
**90**	Gompertz Type-3	116.95	45.77	49.94	41.60	42	91.76	500 km, flat-topped
**90**	Gompertz Type-4	155.44	56.55	62.80	50.29	51	90.19	250 km, flat-topped
**90**	Gompertz Type-4	115.24	44.37	48.34	40.40	42	94.65	500 km, flat-topped
**90**	Gompertz Type-3	154.10	57.32	63.43	51.20	51	88.98	250 km, flat-topped
**91**	Logistic Type-5	35.09	30.25	31.85	28.66	30	99.19	750 km, pointy-topped

**91**	Logistic Type-4	46.79	27.53	29.54	25.51	27	98.09	750 km, flat-topped
**91**	Logistic Type-4	59.56	35.89	38.19	33.59	35	97.53	250 km, pointy-topped
**91**	Logistic Type-5	46.69	27.05	29.14	24.98	27	99.81	750 km, flat-topped
**91**	Logistic Type-5	50.55	26.18	28.19	24.18	27	103.14	500 km, pointy-topped
**91**	Logistic Type-5	53.42	28.80	31.25	26.45	29	100.69	250 km, flat-topped
**91**	Logistic Type-5	60.34	31.09	33.38	28.81	32	102.94	500 km, flat-topped
**91**	Logistic Type-5	59.20	34.82	37.28	32.41	35	100.53	250 km, pointy-topped
**91**	Logistic Type-4	51.97	29.33	31.36	27.31	29	98.86	250 km, flat-topped
**92**	Logistic Type-3	161.56	74.02	81.24	66.80	67	90.52	250 km, pointy-topped
**92**	Logistic Type-4	162.55	71.54	79.61	63.51	67	93.65	250 km, pointy-topped
**92**	Logistic Type-4	160.33	72.97	79.13	66.80	71	97.31	250 km, flat-topped
**92**	Logistic Type-5	162.75	67.64	75.63	60.12	67	99.05	250 km, pointy-topped
**92**	Logistic Type-4	126.90	53.69	58.86	48.51	53	98.72	500 km, pointy-topped
**92**	Logistic Type-4	103.81	47.81	53.40	42.25	45	94.12	750 km, pointy-topped
**92**	Logistic Type-4	156.13	57.30	64.20	50.43	57	99.47	500 km, flat-topped
**92**	Logistic Type-5	161.87	71.69	78.67	64.85	71	99.04	250 km, flat-topped
**92**	Logistic Type-3	101.88	48.30	52.98	43.62	45	93.17	750 km, pointy-topped
**92**	Gompertz Type-4	86.89	56.14	64.57	47.72	42	74.81	750 km, flat-topped
**92**	Logistic Type-4	86.30	49.60	56.53	42.79	42	84.68	750 km, flat-topped
**92**	Logistic Type-5	156.96	55.22	62.02	48.63	57	103.21	500 km, flat-topped
**92**	Logistic Type-5	125.54	51.24	56.15	46.42	53	103.43	500 km, pointy-topped
**92**	Logistic Type-3	157.17	60.79	67.95	53.63	57	93.76	500 km, flat-topped
**92**	Gompertz Type-3	84.89	56.00	61.86	50.14	42	75.00	750 km, flat-topped
**93**	Logistic Type-5	158.87	55.82	59.53	52.13	57	102.12	750 km, flat-topped
**93**	Logistic Type-5	237.30	88.09	92.52	83.66	91	103.31	250 km, flat-topped
**93**	Logistic Type-5	170.29	65.53	68.52	62.55	67	102.24	500 km, pointy-topped
**93**	Logistic Type-5	179.63	65.47	69.01	61.93	68	103.86	500 km, flat-topped
**93**	Logistic Type-5	246.38	92.21	97.86	86.63	95	103.02	250 km, pointy-topped
**93**	Logistic Type-5	135.98	54.65	57.02	52.28	56	102.47	750 km, pointy-topped
**94**	Exponential	30.98	81.09	3856455.28	-59444.95	13	16.03	500 km, pointy-topped
**94**	Logistic Type-4	29.81	29.86	98.14	7.11	11	36.84	750 km, pointy-topped
**94**	Exponential	42.04	94.44	4369737.82	-61510.31	15	15.88	250 km, pointy-topped
**94**	insufficient data	NA	NA	NA	NA	NA	NA	750 km, flat-topped
**94**	Exponential	36.11	88.31	4086033.77	-61675.78	14	15.85	250 km, flat-topped
**94**	Logistic Type-3	27.92	25.79	61.24	8.80	11	42.65	750 km, pointy-topped
**94**	Gompertz Type-3	27.96	28.38	NA	NA	11	38.76	750 km, pointy-topped
**94**	Exponential	28.40	65.02	NA	NA	12	18.46	500 km, flat-topped
**95**	Logistic Type-5	338.68	128.73	137.17	120.29	132	102.54	500 km, pointy-topped
**95**	Logistic Type-4	253.84	88.97	95.72	82.23	91	102.28	750 km, flat-topped
**95**	Logistic Type-4	436.96	223.61	232.85	214.37	223	99.73	250 km, pointy-topped
**95**	Logistic Type-4	336.81	128.02	135.59	120.46	132	103.11	500 km, pointy-topped
**95**	Logistic Type-5	255.30	87.92	95.18	80.71	91	103.50	750 km, flat-topped
**95**	Logistic Type-4	342.08	136.84	144.29	129.38	136	99.39	500 km, flat-topped
**95**	Logistic Type-5	436.71	221.16	231.17	211.15	223	100.83	250 km, pointy-topped

**95**	Logistic Type-4	442.15	223.67	233.91	213.42	224	100.15	250 km, flat-topped
**95**	Logistic Type-5	441.81	227.52	239.16	215.87	224	98.45	250 km, flat-topped
**95**	Logistic Type-5	341.14	134.67	142.27	127.08	136	100.99	500 km, flat-topped
**95**	Logistic Type-4	285.93	99.00	105.54	92.47	98	98.99	750 km, pointy-topped
**95**	Logistic Type-5	285.52	97.12	103.75	90.49	98	100.91	750 km, pointy-topped
**96**	Logistic Type-5	94.25	57.75	61.02	54.47	57	98.71	750 km, flat-topped
**96**	Logistic Type-5	119.43	72.40	76.33	68.46	72	99.45	500 km, pointy-topped
**96**	Gompertz Type-4	158.63	103.96	109.02	98.90	102	98.11	250 km, pointy-topped
**96**	Logistic Type-5	157.81	104.50	109.19	99.81	102	97.61	250 km, flat-topped
**96**	Logistic Type-5	120.29	72.71	76.24	69.19	74	101.77	500 km, flat-topped
**96**	Logistic Type-4	93.34	57.32	60.38	54.27	57	99.44	750 km, flat-topped
**96**	Logistic Type-4	118.31	72.58	75.94	69.22	74	101.96	500 km, flat-topped
**96**	Logistic Type-3	94.36	57.67	60.81	54.54	57	98.83	750 km, flat-topped
**96**	Logistic Type-4	109.67	62.55	65.40	59.70	63	100.72	750 km, pointy-topped
**97**	Logistic Type-3	120.09	49.53	54.86	44.20	52	104.99	250 km, flat-topped
**97**	Logistic Type-3	117.37	47.03	51.70	42.36	49	104.18	250 km, pointy-topped
**97**	Logistic Type-4	118.87	47.71	53.09	42.35	49	102.71	250 km, pointy-topped
**97**	Gompertz Type-3	119.16	51.33	56.98	45.69	49	95.45	250 km, pointy-topped
**97**	Logistic Type-4	95.76	37.50	42.88	32.22	37	98.66	500 km, pointy-topped
**97**	Gompertz Type-3	93.86	38.38	43.26	33.50	37	96.40	500 km, pointy-topped
**97**	Logistic Type-4	121.87	50.00	56.02	44.00	52	104.00	250 km, flat-topped
**97**	Gompertz Type-3	97.73	41.32	46.61	36.03	40	96.80	500 km, flat-topped
**97**	Gompertz Type-4	121.31	51.61	57.98	45.26	52	100.76	250 km, flat-topped
**97**	Gompertz Type-3	121.72	53.68	60.04	47.31	52	96.88	250 km, flat-topped
**97**	Logistic Type-3	78.18	30.83	34.24	27.41	32	103.81	750 km, pointy-topped
**97**	Logistic Type-3	79.86	31.07	34.36	27.78	32	102.99	750 km, flat-topped
**97**	Gompertz Type-4	95.39	41.42	NA	NA	37	89.33	500 km, pointy-topped
**97**	Gompertz Type-4	99.66	40.88	47.34	34.64	40	97.84	500 km, flat-topped
**97**	Logistic Type-3	99.71	38.08	42.80	33.37	40	105.04	500 km, flat-topped
**97**	Logistic Type-4	80.02	30.55	34.20	26.90	32	104.75	750 km, pointy-topped
**98**	insufficient data	NA	NA	NA	NA	NA	NA	750 km, pointy-topped
**98**	insufficient data	NA	NA	NA	NA	NA	NA	250 km, pointy-topped
**98**	insufficient data	NA	NA	NA	NA	NA	NA	500 km, pointy-topped
**98**	insufficient data	NA	NA	NA	NA	NA	NA	500 km, flat-topped
**98**	insufficient data	NA	NA	NA	NA	NA	NA	250 km, flat-topped
**98**	insufficient data	NA	NA	NA	NA	NA	NA	750 km, flat-topped

Table S7.
Shallow-water areas through the Phanerozoic. All values in km².

Bin ID	Stage	Map age (Myr)	Shallow-water area (on paleo-map)	Shallow-water area (re-projected)	Shallow-water area with fossil data (re-projected)
**1**	Chibanian-Late Pleistocene	0	43246117	70159941	14834687
**2**	Calabrian	0	43246117	71674125	13337612
**3**	Gelasian	3	37381009	60152844	2003659
**4**	Piacenzian	3	37381009	55619181	6439704
**5**	Zanclean	3	37381009	50581994	11436111
**6**	Messinian	3	37381009	57361797	4715517
**7**	Tortonian	11	33073271	48723317	9350457
**8**	Serravallian	11	33073271	49570189	8495028
**9**	Langhian	15	29791849	49763642	4247615
**10**	Burdigalian	20	34971182	46564798	11642192
**11**	Aquitanian	20	34971182	55239112	3094686
**12**	Chattian	26	30231773	46748745	7697567
**13**	Rupelian	31	33181700	51851947	5810494
**14**	Priabonian	36	30371347	46906727	5981389
**15**	Bartonian	40	32999610	51361815	3656750
**16**	Lutetian	45	41731810	60295781	4635281
**17**	Ypresian	52	62861103	78336256	6388358
**18**	Thanetian	56	49534951	64393275	4440915
**19**	Selandian	61	49574328	65258415	2095244
**20**	Danian	66	45601796	55521977	5358138
**21**	Maastrichtian	69	51015193	54214404	10143160
**22**	Campanian	75	67074021	63786994	16860484
**23**	Santonian	87	66165265	69186456	7144084
**24**	Coniacian	87	66165265	66595425	9822656
**25**	Turonian	92	66159185	64498493	12428669
**26**	Cenomanian	97	58236532	53296865	13231749
**27**	Albian	107	53746727	48618066	12857804
**28**	Aptian	116	56535746	52842430	10116884
**29**	Barremian	122	67405385	63502836	6241701
**30**	Hauterivian	131	46567553	48816220	5200094
**31**	Valanginian	136	40714078	41791098	5216419
**32**	Berriasian	142	42675157	46401667	5126153
**33**	Tithonian	145	36296354	42360732	5897907
**34**	Kimmeridgian	149	41119059	47379197	5747814
**35**	Oxfordian	160	64980435	60630751	11057038
**36**	Callovian	165	54134106	50892163	8964784
**37**	Bathonian	168	54124580	51118327	8315554
**38**	Bajocian	168	54124580	52192191	7142041

**39**	Aalenian	172	50999193	51788451	4801981
**40**	Toarcian	178	43840101	41067111	6743320
**41**	Pliensbachian	187	25074959	22794039	7022618
**42**	Sinemurian	196	25046448	23480538	5528595
**43**	Hettangian	201	15959354	20683410	2576954
**44**	Rhaetian	205	17665233	19636230	3187438
**45**	Norian	218	24417915	19780591	8387126
**46**	Carnian	232	40069786	23315824	7605359
**47**	Ladinian	240	7845876	7081121	1525684
**48**	Anisian	245	37043499	22976729	5538643
**49**	Olenekian	245	37043499	24115038	4290862
**50**	Induan	252	62981208	43494285	6239007
**51**	Changhsingian	252	62981208	45039645	4832953
**52**	Wuchiapingian	256	62976599	41330924	8166609
**53**	Capitanian	263	70775438	43379343	7476726
**54**	Wordian	265	62980243	38697510	8723492
**55**	Roadian	269	60834265	36135984	10299589
**56**	Kungurian	280	68589864	39439805	12259739
**57**	Artinskian	287	64568254	39907484	11212408
**58**	Sakmarian	293	58645837	37980694	11671864
**59**	Asselian	297	58234710	42637143	11047616
**60**	Gzhelian	301	62350481	52153555	6087222
**61**	Kasimovian	305	61601998	52372727	3401766
**62**	Moscovian	311	62618918	46419089	6897425
**63**	Bashkirian	319	67143819	43640867	6329114
**64**	Serpukhovian	327	62094073	44168740	5633621
**65**	Visean	339	72358314	50628436	10948640
**66**	Tournaisian	354	77702243	57503360	10419644
**67**	Famennian	366	82721260	63403201	10847668
**68**	Frasnian	375	89351292	68322036	14938693
**69**	Givetian	385	90092774	72685557	12402231
**70**	Eifelian	391	82672336	69579386	8918890
**71**	Emsian	400	81808027	63735236	13228450
**72**	Pragian	410	83964146	70077566	6957808
**73**	Lochkovian	415	90832434	73223391	8897295
**74**	Pridoli	421	97138849	73979314	8855489
**75**	Ludfordian	425	95156457	73843036	7629779
**76**	Gorstian	425	95156457	78773813	2807131
**77**	Homerian	430	96523706	75445422	9992518
**78**	Sheinwoodian	430	96523706	82302238	3331586
**79**	Telychian	436	78658276	62579819	7989565
**80**	Aeronian	441	72931666	60008705	4859233
**81**	Rhuddanian	441	72931666	58445603	6485733
**82**	Hirnantian	445	73711274	58083772	6818935

**83**	Katian	449	76587123	49200735	18527111
**84**	Sandbian	456	79013434	54589739	14606981
**85**	Darriwilian	465	81165030	55714769	15621858
**86**	Dapingian	470	83506706	67308667	5324755
**87**	Floian	475	85715370	58667626	15107077
**88**	Tremadocian	482	88424911	59653136	15245024
**89**	Stage 10	492	77585145	59621543	8514331
**90**	Jiangshanian	492	77585145	62682525	5449405
**91**	Paibian	496	76185609	62981368	3162853
**92**	Guzhangian	496	76185609	59939153	6148974
**93**	Drumian	505	73273303	56102420	8604025
**94**	Wuliuan	505	73273303	63001136	1768260
**95**	Stage 4	510	73547688	51801846	13449361
**96**	Stage 3	520	81633423	64045149	9035930
**97**	Stage 2	525	79333911	65513708	4817252
**98**	Fortunian	535	73413096	63058612	485841

Table S8.

Areas of deposits (in km²) retrieved from Macrostrat Database through the Phanerozoic, applying the strict filter (see Methods).

Bin ID	Stage	Area shallow-water deposits	% Area of deposits of reprojected area	Area of deposits (today in the sea)	Area of deposits (today on land)	% Area of deposits on land	Area of deposits total
**1**	Chibanian-Late Pleistocene	504	0.001	123	380	0.001	818
**2**	Calabrian	577	0.001	123	454	0.001	818
**3**	Gelasian	122438	0.204	3294	119144	0.198	359527
**4**	Piacenzian	122686	0.221	3271	119415	0.215	359527
**5**	Zanclean	122143	0.241	3271	118872	0.235	359527
**6**	Messinian	122687	0.214	3271	119416	0.208	359527
**7**	Tortonian	25265	0.052	747	24518	0.050	429427
**8**	Serravallian	24305	0.049	646	23659	0.048	429427
**9**	Langhian	91155	0.183	3158	87997	0.177	429427
**10**	Burdigalian	184919	0.397	4309	180610	0.388	460862
**11**	Aquitanian	191031	0.346	4411	186620	0.338	460862
**12**	Chattian	125	0.000	0	125	0.000	40192
**13**	Rupelian	0	0.000	0	0	0.000	40192
**14**	Priabonian	0	0.000	0	0	0.000	131713
**15**	Bartonian	0	0.000	0	0	0.000	264586
**16**	Lutetian	4495	0.007	0	4495	0.007	220580
**17**	Ypresian	3282	0.004	0	3282	0.004	192748
**18**	Thanetian	5020	0.008	0	5020	0.008	78931
**19**	Selandian	11135	0.017	781	10354	0.016	16256
**20**	Danian	4490	0.008	781	3709	0.007	24793
**21**	Maastrichtian	11936	0.022	5	11932	0.022	199748
**22**	Campanian	33455	0.052	36	33420	0.052	214310
**23**	Santonian	2091	0.003	0	2091	0.003	158638
**24**	Coniacian	16419	0.025	0	16419	0.025	158638
**25**	Turonian	6994	0.011	0	6994	0.011	93553
**26**	Cenomanian	11418	0.021	0	11418	0.021	156670
**27**	Albian	0	0.000	0	0	0.000	174226
**28**	Aptian	0	0.000	0	0	0.000	53706
**29**	Barremian	610	0.001	0	610	0.001	52488
**30**	Hauterivian	0	0.000	0	0	0.000	0
**31**	Valanginian	0	0.000	0	0	0.000	0
**32**	Berriasian	0	0.000	0	0	0.000	0
**33**	Tithonian	16504	0.039	1622	14883	0.035	102315
**34**	Kimmeridgian	18529	0.039	1622	16907	0.036	102315

**35**	Oxfordian	35548	0.059	1622	33926	0.056	102315
**36**	Callovian	42383	0.083	1955	40428	0.079	234434
**37**	Bathonian	26551	0.052	359	26192	0.051	132119
**38**	Bajocian	26806	0.051	359	26447	0.051	132119
**39**	Aalenian	26776	0.052	6	26770	0.052	132119
**40**	Toarcian	0	0.000	0	0	0.000	0
**41**	Pliensbachian	0	0.000	0	0	0.000	0
**42**	Sinemurian	0	0.000	0	0	0.000	0
**43**	Hettangian	0	0.000	0	0	0.000	0
**44**	Rhaetian	0	0.000	0	0	0.000	0
**45**	Norian	0	0.000	0	0	0.000	0
**46**	Carnian	0	0.000	0	0	0.000	0
**47**	Ladinian	10460	0.148	579	9881	0.140	140643
**48**	Anisian	44538	0.194	5600	38938	0.169	167431
**49**	Olenekian	63504	0.263	5525	57978	0.240	167431
**50**	Induan	283307	0.651	38187	245120	0.564	347560
**51**	Changhsingian	293201	0.651	37681	255520	0.567	347560
**52**	Wuchiapingian	347076	0.840	31187	315889	0.764	438078
**53**	Capitanian	316591	0.730	35108	281483	0.649	438078
**54**	Wordian	293053	0.757	36920	256133	0.662	438078
**55**	Roadian	33380	0.092	23025	10354	0.029	477323
**56**	Kungurian	0	0.000	0	0	0.000	7926
**57**	Artinskian	0	0.000	0	0	0.000	23742
**58**	Sakmarian	831	0.002	0	831	0.002	31941
**59**	Asselian	30126	0.071	5	30121	0.071	46199
**60**	Gzhelian	14874	0.029	0	14874	0.029	14874
**61**	Kasimovian	7187	0.014	4	7183	0.014	16708
**62**	Moscovian	10	0.000	0	10	0.000	24775
**63**	Bashkirian	0	0.000	0	0	0.000	3330
**64**	Serpukhovian	0	0.000	0	0	0.000	3330
**65**	Visean	4510	0.009	0	4510	0.009	5595
**66**	Tournaisian	0	0.000	0	0	0.000	0
**67**	Famennian	0	0.000	0	0	0.000	0
**68**	Frasnian	0	0.000	0	0	0.000	0
**69**	Givetian	18670	0.026	352	18318	0.025	71871
**70**	Eifelian	60513	0.087	164	60349	0.087	71871
**71**	Emsian	0	0.000	0	0	0.000	0
**72**	Pragian	0	0.000	0	0	0.000	0
**73**	Lochkovian	0	0.000	0	0	0.000	0
**74**	Pridoli	0	0.000	0	0	0.000	0
**75**	Ludfordian	0	0.000	0	0	0.000	0

**76**	Gorstian	0	0.000	0	0	0.000	0
**77**	Homerian	0	0.000	0	0	0.000	0
**78**	Sheinwoodian	0	0.000	0	0	0.000	0
**79**	Telychian	0	0.000	0	0	0.000	0
**80**	Aeronian	0	0.000	0	0	0.000	0
**81**	Rhuddanian	0	0.000	0	0	0.000	0
**82**	Hirnantian	1444	0.002	3	1441	0.002	1444
**83**	Katian	517	0.001	0	517	0.001	1444
**84**	Sandbian	1318	0.002	0	1318	0.002	1444
**85**	Darriwilian	24020	0.043	314	23706	0.043	24820
**86**	Dapingian	126895	0.189	1130	125765	0.187	129633
**87**	Floian	89497	0.153	819	88678	0.151	106256
**88**	Tremadocian	227932	0.382	766	227167	0.381	284319
**89**	Stage 10	171138	0.287	0	171138	0.287	178063
**90**	Jiangshanian	172775	0.276	0	172775	0.276	178063
**91**	Paibian	82402	0.131	0	82402	0.131	83032
**92**	Guzhangian	63081	0.105	0	63081	0.105	83032
**93**	Drumian	99401	0.177	0	99401	0.177	130876
**94**	Wuliuan	129671	0.206	0	129671	0.206	130876
**95**	Stage 4	23402	0.045	0	23402	0.045	130876
**96**	Stage 3	0	0.000	0	0	0.000	0
**97**	Stage 2	0	0.000	0	0	0.000	0
**98**	Fortunian	0	0.000	0	0	0.000	0

Table S9.

Areas of deposits (in km²) retrieved from Macrostrat Database through the Phanerozoic, applying the moderate filter (see Methods).

Bin ID	Stage	Area shallow-water deposits	% Area of deposits of reprojected area	Area of deposits (today in the sea)	Area of deposits (today on land)	% Area of deposits on land	Area of deposits total
**1**	Chibanian-Late Pleistocene	1697776	2.420	281817	1415959	2.018	16354019
**2**	Calabrian	1844829	2.574	289089	1555740	2.171	16354019
**3**	Gelasian	2448116	4.070	563831	1884285	3.132	21460068
**4**	Piacenzian	2123761	3.818	543890	1579871	2.841	21460068
**5**	Zanclean	1725798	3.412	492067	1233731	2.439	21460068
**6**	Messinian	2223113	3.876	552271	1670843	2.913	21460068
**7**	Tortonian	1345791	2.762	526448	819344	1.682	15864581
**8**	Serravallian	1314704	2.652	515334	799370	1.613	15864581
**9**	Langhian	1833402	3.684	558253	1275149	2.562	15864581
**10**	Burdigalian	2349532	5.046	636502	1713030	3.679	17176746
**11**	Aquitanian	3114568	5.638	660765	2453802	4.442	17176746
**12**	Chattian	802289	1.716	305245	497044	1.063	3157400
**13**	Rupelian	835528	1.611	240366	595162	1.148	3230413
**14**	Priabonian	597353	1.273	80547	516807	1.102	2850808
**15**	Bartonian	661625	1.288	54664	606962	1.182	3003765
**16**	Lutetian	832861	1.381	114589	718272	1.191	2959759
**17**	Ypresian	1240113	1.583	119401	1120712	1.431	2985474
**18**	Thanetian	1205301	1.872	292386	912915	1.418	2665600
**19**	Selandian	1297028	1.988	301146	995881	1.526	2659474
**20**	Danian	1028706	1.853	274878	753829	1.358	2657317
**21**	Maastrichtian	546181	1.007	185672	360510	0.665	4729416
**22**	Campanian	1170335	1.835	176526	993809	1.558	4717130
**23**	Santonian	2031231	2.936	197776	1833456	2.65	4578496
**24**	Coniacian	1743835	2.619	194652	1549183	2.326	4578496
**25**	Turonian	1477194	2.290	191361	1285833	1.994	4513411
**26**	Cenomanian	977213	1.834	205145	772069	1.449	3842396
**27**	Albian	1281192	2.635	793449	487743	1.003	3587974
**28**	Aptian	1356621	2.567	855658	500963	0.948	3406586
**29**	Barremian	1592235	2.507	876152	716083	1.128	3405368
**30**	Hauterivian	1263926	2.589	763504	500421	1.025	3234844
**31**	Valanginian	1175853	2.814	755855	419998	1.005	3234844
**32**	Berriasian	1142439	2.462	758395	384044	0.828	3234844
**33**	Tithonian	1031451	2.435	748084	283366	0.669	3703048

**34**	Kimmeridgian	55501	0.117	6245	49256	0.104	471528
**35**	Oxfordian	139498	0.230	6255	133243	0.22	471516
**36**	Callovian	141831	0.279	22677	119154	0.234	799240
**37**	Bathonian	169432	0.331	18935	150496	0.294	497185
**38**	Bajocian	171934	0.329	18787	153147	0.293	497185
**39**	Aalenian	210818	0.407	17439	193379	0.373	698976
**40**	Toarcian	42875	0.104	4896	37979	0.092	260416
**41**	Pliensbachian	23832	0.105	7496	16335	0.072	260416
**42**	Sinemurian	58556	0.249	1372	57184	0.244	260416
**43**	Hettangian	74692	0.361	26080	48612	0.235	600009
**44**	Rhaetian	69755	0.355	18648	51107	0.26	339593
**45**	Norian	55163	0.279	26290	28873	0.146	339593
**46**	Carnian	88360	0.379	26616	61744	0.265	339593
**47**	Ladinian	10460	0.148	579	9881	0.14	219579
**48**	Anisian	47866	0.208	7215	40651	0.177	344361
**49**	Olenekian	67860	0.281	7160	60700	0.252	344361
**50**	Induan	696294	1.601	40025	656268	1.509	921247
**51**	Changhsingian	705472	1.566	39497	665975	1.479	921247
**52**	Wuchiapingia n	833753	2.017	32618	801135	1.938	1187262
**53**	Capitanian	660209	1.522	39403	620805	1.431	1177936
**54**	Wordian	639761	1.653	40463	599298	1.549	1177936
**55**	Roadian	184459	0.510	24700	159759	0.442	1491679
**56**	Kungurian	12575	0.032	761	11815	0.03	369045
**57**	Artinskian	15131	0.038	720	14411	0.036	298383
**58**	Sakmarian	19475	0.051	954	18521	0.049	306582
**59**	Asselian	56364	0.132	1544	54820	0.129	320839
**60**	Gzhelian	133804	0.257	5477	128327	0.246	392026
**61**	Kasimovian	135358	0.258	5478	129880	0.248	476657
**62**	Moscovian	82010	0.177	5477	76533	0.165	431532
**63**	Bashkirian	262383	0.601	7185	255198	0.585	976073
**64**	Serpukhovian	36691	0.083	1332	35360	0.08	367514
**65**	Visean	77959	0.154	1421	76537	0.151	367788
**66**	Tournaisian	90265	0.157	258	90007	0.157	429984
**67**	Famennian	694459	1.095	65726	628733	0.992	1194420
**68**	Frasnian	327498	0.479	77690	249809	0.366	765303
**69**	Givetian	563619	0.775	96792	466827	0.642	1134488
**70**	Eifelian	382218	0.549	92764	289454	0.416	659350
**71**	Emsian	67628	0.106	3776	63852	0.1	205228
**72**	Pragian	102774	0.147	32886	69887	0.1	205228
**73**	Lochkovian	393067	0.537	101175	291893	0.399	818648
**74**	Pridoli	275416	0.372	111609	163807	0.221	626116

**75**	Ludfordian	312774	0.424	132251	180523	0.244	626116
**76**	Gorstian	471618	0.599	144100	327518	0.416	626116
**77**	Homerian	463709	0.615	141439	322270	0.427	808560
**78**	Sheinwoodian	598218	0.727	148088	450130	0.547	808560
**79**	Telychian	345951	0.553	142650	203300	0.325	789789
**80**	Aeronian	493485	0.822	146036	347449	0.579	789789
**81**	Rhuddanian	441535	0.755	145886	295649	0.506	789789
**82**	Hirnantian	1001443	1.724	287329	714114	1.229	1508598
**83**	Katian	85304	0.173	6101	79203	0.161	718945
**84**	Sandbian	179506	0.329	85703	93803	0.172	718945
**85**	Darriwilian	418069	0.750	185485	232584	0.417	893953
**86**	Dapingian	601465	0.894	181965	419500	0.623	815922
**87**	Floian	231985	0.395	52334	179651	0.306	544827
**88**	Tremadocian	383648	0.643	43551	340097	0.57	761166
**89**	Stage 10	208125	0.349	0	208125	0.349	216339
**90**	Jiangshanian	210272	0.335	8	210263	0.335	216339
**91**	Paibian	126317	0.201	8	126308	0.201	131364
**92**	Guzhangian	106996	0.179	8	106987	0.178	131364
**93**	Drumian	137085	0.244	0	137085	0.244	196846
**94**	Wuliuan	183412	0.291	76	183336	0.291	196846
**95**	Stage 4	124345	0.240	0	124344	0.24	299163
**96**	Stage 3	32741	0.051	344	32397	0.051	129117
**97**	Stage 2	15133	0.023	398	14735	0.022	129117
**98**	Fortunian	106580	0.169	5139	101441	0.161	129117

Table S10.
Summary statistics for the binned fossil collections for the six grid types for the 49-bin approach.

Map age (Myr)	No. cells with fossil data	No. cells overlapping shallow-water regions	% cells with data in shallow-water	Grid type
**3**	497	2306	21.55	250 km, pointy-topped
**3**	284	840	33.81	500 km, pointy-topped
**3**	202	478	42.26	750 km, pointy-topped
**3**	499	2223	22.45	250 km, flat-topped
**3**	285	861	33.1	500 km, flat-topped
**3**	199	477	41.72	750 km, flat-topped
**15**	322	2119	15.2	250 km, pointy-topped
**15**	184	796	23.12	500 km, pointy-topped
**15**	132	457	28.88	750 km, pointy-topped
**15**	311	1978	15.72	250 km, flat-topped
**15**	182	810	22.47	500 km, flat-topped
**15**	131	466	28.11	750 km, flat-topped
**26**	174	2073	8.39	250 km, pointy-topped
**26**	120	791	15.17	500 km, pointy-topped
**26**	89	459	19.39	750 km, pointy-topped
**26**	175	1982	8.83	250 km, flat-topped
**26**	109	817	13.34	500 km, flat-topped
**26**	84	460	18.26	750 km, flat-topped
**36**	176	2129	8.27	250 km, pointy-topped
**36**	117	811	14.43	500 km, pointy-topped
**36**	86	471	18.26	750 km, pointy-topped
**36**	178	2060	8.64	250 km, flat-topped
**36**	117	864	13.54	500 km, flat-topped
**36**	85	465	18.28	750 km, flat-topped
**52**	191	3090	6.18	250 km, pointy-topped
**52**	117	1046	11.19	500 km, pointy-topped
**52**	92	600	15.33	750 km, pointy-topped
**52**	181	3000	6.03	250 km, flat-topped
**52**	115	1068	10.77	500 km, flat-topped
**52**	91	578	15.74	750 km, flat-topped
**61**	157	2531	6.2	250 km, pointy-topped
**61**	106	902	11.75	500 km, pointy-topped
**61**	82	506	16.21	750 km, pointy-topped
**61**	156	2432	6.41	250 km, flat-topped
**61**	98	934	10.49	500 km, flat-topped
**61**	81	498	16.27	750 km, flat-topped
**69**	183	2822	6.48	250 km, pointy-topped

**69**	123	1009	12.19	500 km, pointy-topped
**69**	91	567	16.05	750 km, pointy-topped
**69**	188	2704	6.95	250 km, flat-topped
**69**	126	1033	12.2	500 km, flat-topped
**69**	88	562	15.66	750 km, flat-topped
**75**	255	2917	8.74	250 km, pointy-topped
**75**	152	982	15.48	500 km, pointy-topped
**75**	119	545	21.83	750 km, pointy-topped
**75**	255	2795	9.12	250 km, flat-topped
**75**	151	1005	15.02	500 km, flat-topped
**75**	113	524	21.56	750 km, flat-topped
**87**	272	2773	9.81	250 km, pointy-topped
**87**	164	933	17.58	500 km, pointy-topped
**87**	117	507	23.08	750 km, pointy-topped
**87**	279	2683	10.4	250 km, flat-topped
**87**	162	961	16.86	500 km, flat-topped
**87**	121	512	23.63	750 km, flat-topped
**97**	237	2456	9.65	250 km, pointy-topped
**97**	149	851	17.51	500 km, pointy-topped
**97**	115	477	24.11	750 km, pointy-topped
**97**	230	2343	9.82	250 km, flat-topped
**97**	145	878	16.51	500 km, flat-topped
**97**	111	477	23.27	750 km, flat-topped
**107**	249	2391	10.41	250 km, pointy-topped
**107**	154	827	18.62	500 km, pointy-topped
**107**	123	462	26.62	750 km, pointy-topped
**107**	256	2296	11.15	250 km, flat-topped
**107**	155	850	18.24	500 km, flat-topped
**107**	114	459	24.84	750 km, flat-topped
**116**	165	2358	7	250 km, pointy-topped
**116**	102	812	12.56	500 km, pointy-topped
**116**	83	456	18.2	750 km, pointy-topped
**116**	158	2346	6.73	250 km, flat-topped
**116**	99	850	11.65	500 km, flat-topped
**116**	78	452	17.26	750 km, flat-topped
**127**	126	2361	5.34	250 km, pointy-topped
**127**	80	801	9.99	500 km, pointy-topped
**127**	66	454	14.54	750 km, pointy-topped
**127**	126	2300	5.48	250 km, flat-topped
**127**	85	838	10.14	500 km, flat-topped
**127**	63	447	14.09	750 km, flat-topped
**136**	143	2049	6.98	250 km, pointy-topped
**136**	97	719	13.49	500 km, pointy-topped
**136**	77	411	18.73	750 km, pointy-topped

**136**	128	1932	6.63	250 km, flat-topped
**136**	93	749	12.42	500 km, flat-topped
**136**	80	408	19.61	750 km, flat-topped
**145**	122	1964	6.21	250 km, pointy-topped
**145**	86	702	12.25	500 km, pointy-topped
**145**	67	385	17.4	750 km, pointy-topped
**145**	119	1889	6.3	250 km, flat-topped
**145**	85	723	11.76	500 km, flat-topped
**145**	62	388	15.98	750 km, flat-topped
**155**	239	2061	11.6	250 km, pointy-topped
**155**	137	689	19.88	500 km, pointy-topped
**155**	102	374	27.27	750 km, pointy-topped
**155**	232	1927	12.04	250 km, flat-topped
**155**	147	713	20.62	500 km, flat-topped
**155**	104	371	28.03	750 km, flat-topped
**168**	177	2172	8.15	250 km, pointy-topped
**168**	117	720	16.25	500 km, pointy-topped
**168**	82	387	21.19	750 km, pointy-topped
**168**	169	2045	8.26	250 km, flat-topped
**168**	113	725	15.59	500 km, flat-topped
**168**	83	387	21.45	750 km, flat-topped
**178**	142	1873	7.58	250 km, pointy-topped
**178**	86	639	13.46	500 km, pointy-topped
**178**	69	355	19.44	750 km, pointy-topped
**178**	143	1775	8.06	250 km, flat-topped
**178**	87	650	13.38	500 km, flat-topped
**178**	69	344	20.06	750 km, flat-topped
**191**	237	1642	14.43	250 km, pointy-topped
**191**	130	591	22	500 km, pointy-topped
**191**	93	337	27.6	750 km, pointy-topped
**191**	228	1530	14.9	250 km, flat-topped
**191**	133	597	22.28	500 km, flat-topped
**191**	91	319	28.53	750 km, flat-topped
**201**	50	1332	3.75	250 km, pointy-topped
**201**	37	517	7.16	500 km, pointy-topped
**201**	28	304	9.21	750 km, pointy-topped
**201**	54	1266	4.27	250 km, flat-topped
**201**	35	522	6.7	500 km, flat-topped
**201**	29	287	10.1	750 km, flat-topped
**213**	297	1459	20.36	250 km, pointy-topped
**213**	176	554	31.77	500 km, pointy-topped
**213**	127	315	40.32	750 km, pointy-topped
**213**	267	1372	19.46	250 km, flat-topped
**213**	189	556	33.99	500 km, flat-topped

**213**	123	312	39.42	750 km, flat-topped
**232**	168	1561	10.76	250 km, pointy-topped
**232**	102	554	18.41	500 km, pointy-topped
**232**	81	315	25.71	750 km, pointy-topped
**232**	157	1497	10.49	250 km, flat-topped
**232**	110	553	19.89	500 km, flat-topped
**232**	79	301	26.25	750 km, flat-topped
**245**	198	1434	13.81	250 km, pointy-topped
**245**	116	501	23.15	500 km, pointy-topped
**245**	89	286	31.12	750 km, pointy-topped
**245**	188	1417	13.27	250 km, flat-topped
**245**	113	508	22.24	500 km, flat-topped
**245**	80	288	27.78	750 km, flat-topped
**252**	159	2014	7.89	250 km, pointy-topped
**252**	96	675	14.22	500 km, pointy-topped
**252**	65	358	18.16	750 km, pointy-topped
**252**	162	2012	8.05	250 km, flat-topped
**252**	95	689	13.79	500 km, flat-topped
**252**	68	365	18.63	750 km, flat-topped
**256**	174	2024	8.6	250 km, pointy-topped
**256**	100	676	14.79	500 km, pointy-topped
**256**	72	358	20.11	750 km, pointy-topped
**256**	173	2010	8.61	250 km, flat-topped
**256**	105	689	15.24	500 km, flat-topped
**256**	80	364	21.98	750 km, flat-topped
**265**	346	2039	16.97	250 km, pointy-topped
**265**	194	677	28.66	500 km, pointy-topped
**265**	129	360	35.83	750 km, pointy-topped
**265**	343	2031	16.89	250 km, flat-topped
**265**	192	694	27.67	500 km, flat-topped
**265**	131	362	36.19	750 km, flat-topped
**280**	288	2103	13.69	250 km, pointy-topped
**280**	158	683	23.13	500 km, pointy-topped
**280**	115	363	31.68	750 km, pointy-topped
**280**	291	2110	13.79	250 km, flat-topped
**280**	162	693	23.38	500 km, flat-topped
**280**	109	361	30.19	750 km, flat-topped
**293**	237	1854	12.78	250 km, pointy-topped
**293**	133	621	21.42	500 km, pointy-topped
**293**	95	346	27.46	750 km, pointy-topped
**293**	236	1886	12.51	250 km, flat-topped
**293**	135	630	21.43	500 km, flat-topped
**293**	102	335	30.45	750 km, flat-topped
**301**	93	1931	4.82	250 km, pointy-topped

**301**	59	649	9.09	500 km, pointy-topped
**301**	45	345	13.04	750 km, pointy-topped
**301**	88	1969	4.47	250 km, flat-topped
**301**	58	668	8.68	500 km, flat-topped
**301**	45	355	12.68	750 km, flat-topped
**315**	126	1891	6.66	250 km, pointy-topped
**315**	84	609	13.79	500 km, pointy-topped
**315**	71	315	22.54	750 km, pointy-topped
**315**	130	1908	6.81	250 km, flat-topped
**315**	87	624	13.94	500 km, flat-topped
**315**	65	329	19.76	750 km, flat-topped
**327**	65	1798	3.62	250 km, pointy-topped
**327**	44	584	7.53	500 km, pointy-topped
**327**	36	305	11.8	750 km, pointy-topped
**327**	67	1812	3.7	250 km, flat-topped
**327**	44	594	7.41	500 km, flat-topped
**327**	35	315	11.11	750 km, flat-topped
**339**	175	2062	8.49	250 km, pointy-topped
**339**	105	657	15.98	500 km, pointy-topped
**339**	73	342	21.35	750 km, pointy-topped
**339**	173	2068	8.37	250 km, flat-topped
**339**	102	670	15.22	500 km, flat-topped
**339**	75	337	22.26	750 km, flat-topped
**354**	117	2141	5.46	250 km, pointy-topped
**354**	77	670	11.49	500 km, pointy-topped
**354**	57	365	15.62	750 km, pointy-topped
**354**	123	2166	5.68	250 km, flat-topped
**354**	80	688	11.63	500 km, flat-topped
**354**	55	356	15.45	750 km, flat-topped
**366**	138	2245	6.15	250 km, pointy-topped
**366**	86	689	12.48	500 km, pointy-topped
**366**	65	361	18.01	750 km, pointy-topped
**366**	147	2256	6.52	250 km, flat-topped
**366**	82	698	11.75	500 km, flat-topped
**366**	65	364	17.86	750 km, flat-topped
**375**	195	2367	8.24	250 km, pointy-topped
**375**	115	724	15.88	500 km, pointy-topped
**375**	93	370	25.14	750 km, pointy-topped
**375**	195	2402	8.12	250 km, flat-topped
**375**	117	742	15.77	500 km, flat-topped
**375**	91	386	23.58	750 km, flat-topped
**385**	225	2436	9.24	250 km, pointy-topped
**385**	148	759	19.5	500 km, pointy-topped
**385**	109	383	28.46	750 km, pointy-topped

**385**	229	2441	9.38	250 km, flat-topped
**385**	146	764	19.11	500 km, flat-topped
**385**	107	393	27.23	750 km, flat-topped
**400**	180	2217	8.12	250 km, pointy-topped
**400**	115	697	16.5	500 km, pointy-topped
**400**	89	370	24.05	750 km, pointy-topped
**400**	184	2221	8.28	250 km, flat-topped
**400**	114	699	16.31	500 km, flat-topped
**400**	96	371	25.88	750 km, flat-topped
**415**	169	2401	7.04	250 km, pointy-topped
**415**	115	745	15.44	500 km, pointy-topped
**415**	91	387	23.51	750 km, pointy-topped
**415**	174	2396	7.26	250 km, flat-topped
**415**	111	754	14.72	500 km, flat-topped
**415**	85	385	22.08	750 km, flat-topped
**425**	305	2549	11.97	250 km, pointy-topped
**425**	182	773	23.54	500 km, pointy-topped
**425**	120	402	29.85	750 km, pointy-topped
**425**	301	2545	11.83	250 km, flat-topped
**425**	177	777	22.78	500 km, flat-topped
**425**	124	405	30.62	750 km, flat-topped
**436**	264	2260	11.68	250 km, pointy-topped
**436**	153	730	20.96	500 km, pointy-topped
**436**	111	376	29.52	750 km, pointy-topped
**436**	257	2252	11.41	250 km, flat-topped
**436**	155	736	21.06	500 km, flat-topped
**436**	114	384	29.69	750 km, flat-topped
**449**	336	2124	15.82	250 km, pointy-topped
**449**	178	681	26.14	500 km, pointy-topped
**449**	125	367	34.06	750 km, pointy-topped
**449**	329	2153	15.28	250 km, flat-topped
**449**	176	689	25.54	500 km, flat-topped
**449**	119	374	31.82	750 km, flat-topped
**456**	200	2163	9.25	250 km, pointy-topped
**456**	115	680	16.91	500 km, pointy-topped
**456**	81	370	21.89	750 km, pointy-topped
**456**	215	2181	9.86	250 km, flat-topped
**456**	110	697	15.78	500 km, flat-topped
**456**	77	382	20.16	750 km, flat-topped
**465**	229	2202	10.4	250 km, pointy-topped
**465**	128	706	18.13	500 km, pointy-topped
**465**	87	370	23.51	750 km, pointy-topped
**465**	220	2231	9.86	250 km, flat-topped
**465**	124	714	17.37	500 km, flat-topped

**465**	96	377	25.46	750 km, flat-topped
**475**	164	2289	7.16	250 km, pointy-topped
**475**	103	721	14.29	500 km, pointy-topped
**475**	77	381	20.21	750 km, pointy-topped
**475**	165	2299	7.18	250 km, flat-topped
**475**	98	727	13.48	500 km, flat-topped
**475**	75	392	19.13	750 km, flat-topped
**482**	162	2366	6.85	250 km, pointy-topped
**482**	102	748	13.64	500 km, pointy-topped
**482**	72	391	18.41	750 km, pointy-topped
**482**	164	2363	6.94	250 km, flat-topped
**482**	104	752	13.83	500 km, flat-topped
**482**	77	402	19.15	750 km, flat-topped
**492**	146	2116	6.9	250 km, pointy-topped
**492**	95	648	14.66	500 km, pointy-topped
**492**	68	360	18.89	750 km, pointy-topped
**492**	138	2125	6.49	250 km, flat-topped
**492**	95	684	13.89	500 km, flat-topped
**492**	75	365	20.55	750 km, flat-topped
**505**	203	1975	10.28	250 km, pointy-topped
**505**	124	653	18.99	500 km, pointy-topped
**505**	88	347	25.36	750 km, pointy-topped
**505**	199	1976	10.07	250 km, flat-topped
**505**	133	646	20.59	500 km, flat-topped
**505**	89	349	25.5	750 km, flat-topped
**515**	210	2066	10.16	250 km, pointy-topped
**515**	121	666	18.17	500 km, pointy-topped
**515**	94	350	26.86	750 km, pointy-topped
**515**	213	2087	10.21	250 km, flat-topped
**515**	124	678	18.29	500 km, flat-topped
**515**	86	359	23.96	750 km, flat-topped
**530**	50	2037	2.45	250 km, pointy-topped
**530**	38	637	5.97	500 km, pointy-topped
**530**	32	350	9.14	750 km, pointy-topped
**530**	55	2039	2.7	250 km, flat-topped
**530**	41	658	6.23	500 km, flat-topped
**530**	32	349	9.17	750 km, flat-topped

Table S11.

Results for the logistic and logistic-like (Gompertz) models estimating fossil data coverage of shallow-marine regions based on collection history for the 49-bin approach. Predicted completeness refers to the percentage of the observed number of grid cells containing fossil data relative to the predicted value for 2100 (see Methods for details).

Map age (Myr)	Model	AIC	Predicted no. cells	Upper CI	Lower CI	Observed no. cells with fossils	Predicted complete-ness	Grid type
**3**	Gompertz Type-4	1027.82	793.84	823.91	763.77	495	62.36	250 km, pointy-topped
**3**	Gompertz Type-4	986.58	769.57	798.27	740.87	499	64.84	250 km, flat-topped
**3**	Gompertz Type-4	747.69	429.45	450.15	408.74	285	66.36	500 km, flat-topped
**3**	Gompertz Type-4	779.98	396.80	417.71	375.89	284	71.57	500 km, pointy-topped
**3**	Gompertz Type-4	565.23	274.16	290.66	257.65	201	73.32	750 km, pointy-topped
**3**	Logistic Type-5	579.56	243.76	260.23	227.28	200	82.05	750 km, flat-topped
**3**	Logistic Type-3	577.72	238.85	250.72	226.98	200	83.74	750 km, flat-topped
**3**	Logistic Type-4	579.16	236.21	249.17	223.26	200	84.67	750 km, flat-topped
**15**	Gompertz Type-4	768.97	495.71	513.81	477.62	310	62.54	250 km, flat-topped
**15**	Gompertz Type-3	767.22	489.63	507.61	471.66	310	63.31	250 km, flat-topped
**15**	Gompertz Type-3	506.51	276.50	287.65	265.34	184	66.55	500 km, pointy-topped
**15**	Gompertz Type-3	749.35	479.64	498.87	460.41	321	66.93	250 km, pointy-topped
**15**	Gompertz Type-3	436.10	195.17	206.91	183.44	131	67.12	750 km, flat-topped
**15**	Gompertz Type-4	507.83	273.36	285.12	261.59	184	67.31	500 km, pointy-topped
**15**	Gompertz Type-4	748.01	468.52	487.95	449.08	321	68.51	250 km, pointy-topped
**15**	Gompertz Type-4	436.41	188.94	201.85	176.04	131	69.33	750 km, flat-topped
**15**	Gompertz Type-3	386.73	186.39	195.46	177.31	131	70.28	750 km, pointy-topped
**15**	Gompertz Type-4	385.72	180.61	190.39	170.82	131	72.53	750 km, pointy-topped
**15**	Gompertz Type-4	557.28	223.80	237.81	209.79	181	80.88	500 km, flat-topped
**26**	Gompertz Type-4	393.88	178.22	188.92	167.51	109	61.16	500 km, flat-topped
**26**	Gompertz Type-4	423.29	189.91	202.75	177.06	120	63.19	500 km, pointy-topped
**26**	Gompertz Type-4	545.75	273.83	287.61	260.05	174	63.54	250 km, pointy-topped
**26**	Gompertz Type-4	530.21	272.36	287.16	257.56	175	64.25	250 km, flat-topped
**26**	Gompertz Type-3	547.03	264.75	279.90	249.60	174	65.72	250 km, pointy-topped
**26**	Gompertz Type-3	531.19	263.98	279.37	248.59	175	66.29	250 km, flat-topped
**26**	Gompertz Type-3	423.69	180.35	195.36	165.35	120	66.54	500 km, pointy-topped
**26**	Gompertz Type-4	290.80	120.99	128.41	113.57	84	69.43	750 km, flat-topped
**26**	Gompertz Type-4	312.21	123.98	132.69	115.27	89	71.79	750 km, pointy-topped
**26**	Gompertz Type-3	291.04	116.36	124.36	108.36	84	72.19	750 km, flat-topped
**26**	Gompertz Type-3	311.29	121.18	129.49	112.87	89	73.45	750 km, pointy-topped
**36**	Gompertz Type-4	441.59	214.13	220.86	207.39	175	81.73	250 km, pointy-topped
**36**	Gompertz Type-4	237.85	102.22	107.10	97.34	84	82.18	750 km, flat-topped
**36**	Gompertz Type-4	399.78	212.62	218.56	206.67	177	83.25	250 km, flat-topped
**36**	Gompertz Type-4	345.16	137.46	143.13	131.80	116	84.39	500 km, flat-topped

**36**	Logistic Type-5	281.80	133.42	138.15	128.69	116	86.94	500 km, pointy-topped
**36**	Gompertz Type-4	268.32	95.96	100.90	91.02	85	88.58	750 km, pointy-topped
**52**	Gompertz Type-3	332.26	151.32	159.66	142.98	116	76.66	500 km, pointy-topped
**52**	Gompertz Type-4	334.18	150.81	159.69	141.93	116	76.92	500 km, pointy-topped
**52**	Gompertz Type-4	271.89	146.43	151.70	141.17	114	77.85	500 km, flat-topped
**52**	Gompertz Type-4	532.40	243.43	257.52	229.35	190	78.05	250 km, pointy-topped
**52**	Gompertz Type-4	483.16	222.15	234.76	209.54	180	81.03	250 km, flat-topped
**52**	Logistic Type-5	534.17	226.27	239.97	212.57	190	83.97	250 km, pointy-topped
**52**	Gompertz Type-4	228.81	103.35	107.79	98.91	90	87.08	750 km, flat-topped
**52**	Logistic Type-5	250.89	101.18	107.80	94.56	91	89.94	750 km, pointy-topped
**52**	Logistic Type-4	250.29	98.42	103.33	93.51	91	92.46	750 km, pointy-topped
**52**	Logistic Type-3	248.99	97.63	102.06	93.21	91	93.21	750 km, pointy-topped
**61**	Gompertz Type-3	213.11	173.02	180.50	165.55	82	47.39	750 km, flat-topped
**61**	Gompertz Type-3	276.76	195.46	203.93	187.00	105	53.72	500 km, pointy-topped
**61**	Gompertz Type-4	277.66	189.12	198.61	179.63	105	55.52	500 km, pointy-topped
**61**	Logistic Type-4	214.23	143.68	165.65	121.70	82	57.07	750 km, flat-topped
**61**	Gompertz Type-3	385.83	269.73	282.98	256.48	156	57.84	250 km, pointy-topped
**61**	Gompertz Type-4	387.10	264.32	278.49	250.15	156	59.02	250 km, pointy-topped
**61**	Gompertz Type-3	245.72	163.64	171.76	155.52	97	59.28	500 km, flat-topped
**61**	Gompertz Type-4	247.14	159.79	168.44	151.14	97	60.70	500 km, flat-topped
**61**	Logistic Type-3	214.31	129.65	145.03	114.28	82	63.25	750 km, flat-topped
**61**	Gompertz Type-4	392.13	243.60	261.06	226.14	156	64.04	250 km, flat-topped
**61**	Logistic Type-4	195.07	117.55	128.16	106.93	82	69.76	750 km, pointy-topped
**61**	Logistic Type-5	197.02	114.89	130.27	99.51	82	71.37	750 km, pointy-topped
**61**	Logistic Type-3	195.24	113.22	120.92	105.52	82	72.43	750 km, pointy-topped
**61**	Logistic Type-4	246.60	133.85	146.19	121.51	97	72.47	500 km, flat-topped
**69**	Logistic Type-3	403.79	241.15	251.75	230.56	187	77.54	250 km, flat-topped
**69**	Logistic Type-4	403.76	237.37	248.78	225.96	187	78.78	250 km, flat-topped
**69**	Logistic Type-3	308.97	152.99	160.21	145.77	122	79.74	500 km, pointy-topped
**69**	Logistic Type-4	308.53	150.35	158.08	142.62	122	81.14	500 km, pointy-topped
**69**	Logistic Type-5	404.49	229.45	250.99	208.05	187	81.50	250 km, flat-topped
**69**	Logistic Type-4	216.43	108.81	113.93	103.70	91	83.63	750 km, pointy-topped
**69**	Logistic Type-5	309.88	145.87	157.77	133.99	122	83.63	500 km, pointy-topped
**69**	Logistic Type-5	218.32	107.43	115.42	99.43	91	84.71	750 km, pointy-topped
**69**	Logistic Type-5	292.80	132.56	139.57	125.63	125	94.30	500 km, flat-topped
**69**	Logistic Type-5	400.06	189.57	198.63	180.58	182	96.01	250 km, pointy-topped
**69**	Logistic Type-5	211.92	90.18	94.64	85.75	88	97.58	750 km, flat-topped
**75**	Logistic Type-4	317.69	226.30	253.13	199.47	119	52.59	750 km, pointy-topped
**75**	Logistic Type-3	319.17	197.59	216.57	178.61	119	60.23	750 km, pointy-topped
**75**	Logistic Type-4	398.12	240.26	268.34	212.19	150	62.43	500 km, flat-topped
**75**	Logistic Type-4	391.23	232.53	258.44	206.62	151	64.94	500 km, pointy-topped
**75**	Logistic Type-5	317.85	183.04	212.26	153.82	119	65.01	750 km, pointy-topped
**75**	Logistic Type-4	319.16	174.28	194.52	154.04	114	65.41	750 km, flat-topped
**75**	Logistic Type-5	392.79	228.47	262.14	194.80	151	66.09	500 km, pointy-topped

**75**	Logistic Type-3	398.08	223.52	240.49	206.55	150	67.11	500 km, flat-topped
**75**	Logistic Type-3	317.77	167.72	181.30	154.14	114	67.97	750 km, flat-topped
**75**	Logistic Type-3	392.43	218.92	235.52	202.32	151	68.98	500 km, pointy-topped
**75**	Logistic Type-5	318.99	158.69	185.57	132.89	114	71.84	750 km, flat-topped
**75**	Logistic Type-4	579.44	340.92	362.54	319.30	254	74.50	250 km, pointy-topped
**75**	Logistic Type-3	577.88	335.97	352.45	319.49	254	75.60	250 km, pointy-topped
**75**	Logistic Type-5	577.97	306.52	332.55	280.52	254	82.87	250 km, pointy-topped
**75**	Logistic Type-5	559.69	295.54	318.91	272.34	255	86.28	250 km, flat-topped
**87**	Logistic Type-5	553.63	290.45	301.47	279.44	279	96.06	250 km, flat-topped
**87**	Logistic Type-5	596.42	274.47	284.89	264.06	271	98.73	250 km, pointy-topped
**87**	Logistic Type-5	323.21	118.49	123.67	113.31	117	98.75	750 km, pointy-topped
**87**	Logistic Type-5	448.54	164.48	172.25	156.70	164	99.71	500 km, pointy-topped
**87**	Logistic Type-5	410.73	161.89	169.16	154.62	162	100.00	500 km, flat-topped
**87**	Logistic Type-5	306.46	120.75	125.00	116.50	121	100.00	750 km, flat-topped
**97**	Logistic Type-5	489.49	244.99	254.83	235.15	234	95.51	250 km, pointy-topped
**97**	Logistic Type-5	341.80	152.56	161.22	143.98	146	95.70	500 km, pointy-topped
**97**	Logistic Type-5	283.37	113.61	119.71	107.52	109	95.94	750 km, flat-topped
**97**	Logistic Type-5	489.92	231.42	240.43	222.40	227	98.09	250 km, flat-topped
**97**	Logistic Type-5	281.73	114.45	120.43	108.49	113	98.73	750 km, pointy-topped
**97**	Logistic Type-5	352.42	141.36	146.91	135.81	143	100.00	500 km, flat-topped
**107**	Logistic Type-5	466.65	261.71	271.87	251.55	248	94.76	250 km, pointy-topped
**107**	Logistic Type-5	482.95	266.63	276.52	256.74	255	95.64	250 km, flat-topped
**107**	Logistic Type-5	357.35	157.89	164.74	151.05	154	97.53	500 km, flat-topped
**107**	Logistic Type-5	343.14	155.49	160.92	150.06	153	98.40	500 km, pointy-topped
**107**	Logistic Type-5	318.38	122.92	128.13	117.70	122	99.25	750 km, pointy-topped
**107**	Logistic Type-5	304.49	112.67	117.87	107.47	113	100.00	750 km, flat-topped
**116**	Logistic Type-4	172.01	98.33	105.67	90.98	78	79.33	750 km, flat-topped
**116**	Logistic Type-4	256.90	118.66	126.43	110.88	102	85.96	500 km, pointy-topped
**116**	Logistic Type-5	255.61	111.91	121.42	102.59	102	91.14	500 km, pointy-topped
**116**	Logistic Type-4	402.74	178.20	187.02	169.37	165	92.59	250 km, pointy-topped
**116**	Logistic Type-5	225.76	105.15	111.23	99.09	99	94.15	500 km, flat-topped
**116**	Logistic Type-5	193.95	88.02	93.71	82.41	83	94.30	750 km, pointy-topped
**116**	Logistic Type-5	323.88	165.74	175.13	156.36	158	95.33	250 km, flat-topped
**116**	Logistic Type-5	401.03	172.91	182.83	162.99	165	95.42	250 km, pointy-topped
**127**	Gompertz Type-4	211.36	108.05	117.83	98.26	84	77.74	500 km, flat-topped
**127**	Gompertz Type-4	291.77	142.12	152.05	132.19	123	86.55	250 km, pointy-topped
**127**	Gompertz Type-4	160.33	76.10	81.30	70.90	66	86.73	750 km, pointy-topped
**127**	Gompertz Type-4	186.92	89.76	95.44	84.08	79	88.01	500 km, pointy-topped
**127**	Logistic Type-5	161.61	73.63	78.98	68.28	66	89.63	750 km, pointy-topped
**127**	Gompertz Type-4	302.92	138.23	148.22	128.24	125	90.43	250 km, flat-topped
**127**	Logistic Type-5	188.35	86.44	92.29	80.60	79	91.39	500 km, pointy-topped
**127**	Logistic Type-5	290.37	134.10	144.43	123.77	123	91.72	250 km, pointy-topped
**127**	Logistic Type-5	304.55	134.63	144.75	124.52	125	92.84	250 km, flat-topped
**127**	Logistic Type-5	149.63	66.76	72.22	61.37	62	92.87	750 km, flat-topped

**127**	Logistic Type-4	149.68	64.32	68.01	60.63	62	96.39	750 km, flat-topped
**136**	Logistic Type-4	247.61	109.39	117.38	101.40	98	89.58	500 km, pointy-topped
**136**	Logistic Type-4	224.79	101.35	107.63	95.08	93	91.76	500 km, flat-topped
**136**	Logistic Type-4	227.26	83.08	90.34	75.82	77	92.68	750 km, pointy-topped
**136**	Logistic Type-5	248.16	105.54	115.26	96.06	98	92.86	500 km, pointy-topped
**136**	Logistic Type-4	318.50	137.32	145.68	128.96	129	93.94	250 km, flat-topped
**136**	Logistic Type-5	228.67	81.91	91.22	73.03	77	94.00	750 km, pointy-topped
**136**	Logistic Type-4	331.93	151.98	160.01	143.95	143	94.09	250 km, pointy-topped
**136**	Logistic Type-4	226.62	85.90	90.84	80.96	81	94.30	750 km, flat-topped
**136**	Logistic Type-5	224.15	97.90	105.30	90.61	93	95.00	500 km, flat-topped
**136**	Logistic Type-5	227.37	84.60	90.48	78.75	81	95.74	750 km, flat-topped
**136**	Logistic Type-5	319.13	134.52	143.83	125.21	129	95.90	250 km, flat-topped
**136**	Logistic Type-5	330.82	148.07	156.96	139.18	143	96.58	250 km, pointy-topped
**145**	Gompertz Type-4	321.99	133.37	141.00	125.74	122	91.48	250 km, pointy-topped
**145**	Gompertz Type-4	249.22	91.28	98.17	84.40	85	93.12	500 km, flat-topped
**145**	Gompertz Type-4	197.12	70.77	76.77	64.77	67	94.67	750 km, pointy-topped
**145**	Logistic Type-5	170.09	65.96	71.10	60.89	63	95.51	750 km, flat-topped
**145**	Logistic Type-4	223.87	90.02	94.90	85.15	86	95.53	500 km, pointy-topped
**145**	Logistic Type-4	310.65	123.97	130.71	117.22	119	95.99	250 km, flat-topped
**145**	Logistic Type-5	312.50	123.55	131.68	115.44	119	96.32	250 km, flat-topped
**145**	Logistic Type-4	168.23	65.33	69.19	61.47	63	96.43	750 km, flat-topped
**145**	Logistic Type-5	322.69	125.25	133.13	117.38	122	97.40	250 km, pointy-topped
**145**	Logistic Type-4	321.29	124.78	131.46	118.10	122	97.77	250 km, pointy-topped
**145**	Logistic Type-5	197.61	67.85	74.38	61.45	67	98.75	750 km, pointy-topped
**145**	Logistic Type-4	195.78	67.29	72.63	61.95	67	99.57	750 km, pointy-topped
**155**	Logistic Type-5	507.39	237.76	248.93	226.60	233	98.00	250 km, flat-topped
**155**	Logistic Type-5	495.64	242.25	253.44	231.06	238	98.25	250 km, pointy-topped
**155**	Logistic Type-5	402.14	147.36	156.19	138.52	147	99.76	500 km, flat-topped
**155**	Logistic Type-5	371.37	137.09	145.71	128.47	137	99.94	500 km, pointy-topped
**155**	Logistic Type-5	277.02	103.68	110.95	96.41	105	100.00	750 km, flat-topped
**155**	Logistic Type-5	275.56	100.15	107.38	92.91	102	100.00	750 km, pointy-topped
**168**	Logistic Type-5	276.82	121.77	127.71	115.83	118	96.91	500 km, pointy-topped
**168**	Logistic Type-5	412.27	183.44	192.22	174.66	178	97.04	250 km, pointy-topped
**168**	Logistic Type-5	257.33	116.58	121.70	111.46	114	97.79	500 km, flat-topped
**168**	Logistic Type-5	398.01	171.50	180.45	162.54	169	98.54	250 km, flat-topped
**168**	Logistic Type-5	177.28	82.23	86.13	78.33	82	99.72	750 km, pointy-topped
**168**	Logistic Type-5	210.63	82.93	87.45	78.41	84	100.00	750 km, flat-topped
**178**	Logistic Type-5	212.00	100.93	111.92	90.25	86	85.21	500 km, pointy-topped
**178**	Logistic Type-4	346.85	163.17	175.27	151.06	140	85.80	250 km, pointy-topped
**178**	Logistic Type-4	173.94	79.33	85.74	72.91	69	86.98	750 km, flat-topped
**178**	Logistic Type-4	210.24	98.39	104.80	91.98	86	87.41	500 km, flat-topped
**178**	Logistic Type-4	210.10	97.68	104.40	90.95	86	88.05	500 km, pointy-topped
**178**	Logistic Type-5	173.22	74.21	81.28	67.41	69	92.99	750 km, flat-topped
**178**	Logistic Type-5	161.66	70.21	75.59	65.03	68	96.86	750 km, pointy-topped

**178**	Logistic Type-5	310.88	140.07	147.27	132.89	142	100.00	250 km, flat-topped
**191**	Gompertz Type-4	227.68	107.02	115.60	98.44	91	85.03	750 km, flat-topped
**191**	Logistic Type-5	227.35	100.12	109.80	90.64	91	90.89	750 km, flat-topped
**191**	Logistic Type-4	226.56	96.65	103.32	89.99	91	94.15	750 km, flat-topped
**191**	Logistic Type-4	199.93	95.76	101.11	90.41	92	96.07	750 km, pointy-topped
**191**	Logistic Type-5	200.02	93.93	99.76	88.10	92	97.95	750 km, pointy-topped
**191**	Logistic Type-5	290.54	131.67	140.17	123.18	130	98.73	500 km, pointy-topped
**191**	Logistic Type-5	300.83	133.17	141.18	125.17	133	99.87	500 km, flat-topped
**191**	Logistic Type-5	439.60	226.16	236.72	215.59	227	100.00	250 km, flat-topped
**191**	Logistic Type-5	464.78	230.54	242.99	218.09	235	100.00	250 km, pointy-topped
**201**	Gompertz Type-4	138.78	97.91	132.23	65.06	50	51.07	250 km, pointy-topped
**201**	Gompertz Type-4	111.60	66.09	92.86	42.28	37	55.98	500 km, pointy-topped
**201**	Gompertz Type-4	82.87	49.22	70.55	31.61	30	60.95	750 km, flat-topped
**201**	Gompertz Type-4	158.47	86.54	106.50	66.73	55	63.55	250 km, flat-topped
**201**	Logistic Type-3	113.02	55.21	72.09	39.70	37	67.02	500 km, pointy-topped
**201**	Logistic Type-4	113.19	50.97	64.51	38.45	37	72.59	500 km, pointy-topped
**201**	Logistic Type-4	137.27	67.89	80.28	55.66	50	73.64	250 km, pointy-topped
**201**	Logistic Type-3	158.77	72.68	84.32	61.06	55	75.67	250 km, flat-topped
**201**	Logistic Type-4	84.79	38.64	49.22	29.17	30	77.65	750 km, flat-topped
**201**	Logistic Type-4	156.83	68.91	78.76	59.08	55	79.81	250 km, flat-topped
**201**	Logistic Type-5	158.33	60.79	84.92	50.58	55	90.48	250 km, flat-topped
**201**	Logistic Type-5	137.58	51.94	64.29	45.36	50	96.26	250 km, pointy-topped
**201**	Logistic Type-5	89.53	35.18	38.35	32.18	35	99.48	500 km, flat-topped
**201**	Logistic Type-5	67.35	27.92	30.96	25.01	28	100.00	750 km, pointy-topped
**213**	Logistic Type-5	287.92	126.29	135.62	117.02	127	100.00	750 km, pointy-topped
**213**	Logistic Type-5	391.71	175.38	187.37	163.38	177	100.00	500 km, pointy-topped
**213**	Logistic Type-5	406.24	188.06	200.72	175.41	190	100.00	500 km, flat-topped
**213**	Logistic Type-5	284.06	122.12	131.25	112.98	124	100.00	750 km, flat-topped
**213**	Logistic Type-5	551.87	292.20	308.79	275.62	298	100.00	250 km, pointy-topped
**213**	Logistic Type-5	518.33	261.98	276.28	247.68	268	100.00	250 km, flat-topped
**232**	Logistic Type-3	253.56	182.14	336.15	85.45	81	44.47	750 km, pointy-topped
**232**	Logistic Type-4	254.30	105.07	157.21	71.27	81	77.09	750 km, pointy-topped
**232**	Logistic Type-5	253.47	88.15	114.82	68.89	81	91.89	750 km, pointy-topped
**232**	Logistic Type-5	271.21	105.05	115.77	94.54	103	98.05	500 km, pointy-topped
**232**	Logistic Type-5	198.05	80.77	87.67	73.92	80	99.04	750 km, flat-topped
**232**	Logistic Type-5	317.17	111.76	126.47	99.05	111	99.32	500 km, flat-topped
**232**	Logistic Type-5	388.24	166.76	181.35	152.25	169	100.00	250 km, pointy-topped
**232**	Logistic Type-5	364.15	155.80	167.44	144.17	158	100.00	250 km, flat-topped
**245**	Logistic Type-5	358.83	123.29	134.77	111.82	116	94.08	500 km, pointy-topped
**245**	Logistic Type-5	513.81	195.42	208.17	182.66	198	100.00	250 km, pointy-topped
**245**	Logistic Type-5	316.89	111.30	119.43	103.19	113	100.00	500 km, flat-topped
**245**	Logistic Type-5	252.39	79.65	86.05	73.25	81	100.00	750 km, flat-topped
**245**	Logistic Type-5	450.91	184.31	195.31	173.32	189	100.00	250 km, flat-topped
**245**	Logistic Type-5	258.57	85.72	92.31	79.14	89	100.00	750 km, pointy-topped

**252**	Logistic Type-5	232.65	68.50	76.33	61.38	69	100.00	750 km, flat-topped
**252**	Logistic Type-5	214.58	64.41	72.26	57.22	65	100.00	750 km, pointy-topped
**252**	Logistic Type-5	292.35	94.42	101.86	87.05	96	100.00	500 km, pointy-topped
**252**	Logistic Type-5	409.37	159.53	169.19	149.88	163	100.00	250 km, flat-topped
**252**	Logistic Type-5	288.43	91.96	100.34	83.61	95	100.00	500 km, flat-topped
**252**	Logistic Type-5	427.59	153.58	164.95	142.22	159	100.00	250 km, pointy-topped
**256**	Logistic Type-5	301.90	103.23	110.31	96.16	105	100.00	500 km, flat-topped
**256**	Logistic Type-5	250.00	78.38	84.65	72.11	81	100.00	750 km, flat-topped
**256**	Logistic Type-5	216.97	69.40	74.28	64.51	72	100.00	750 km, pointy-topped
**256**	Logistic Type-5	304.11	95.60	102.71	88.50	100	100.00	500 km, pointy-topped
**256**	Logistic Type-5	466.27	164.46	177.49	151.44	173	100.00	250 km, flat-topped
**256**	Logistic Type-5	490.50	163.86	177.34	150.39	174	100.00	250 km, pointy-topped
**265**	Logistic Type-4	612.87	347.71	367.42	328.01	346	99.51	250 km, pointy-topped
**265**	Logistic Type-5	324.49	131.43	141.10	121.76	132	100.00	750 km, flat-topped
**265**	Logistic Type-5	439.28	190.47	199.78	181.17	194	100.00	500 km, pointy-topped
**265**	Logistic Type-5	318.87	126.39	135.21	117.59	129	100.00	750 km, pointy-topped
**265**	Logistic Type-5	412.70	187.66	197.94	177.37	192	100.00	500 km, flat-topped
**265**	Logistic Type-5	618.31	333.19	349.49	316.89	343	100.00	250 km, flat-topped
**280**	Logistic Type-5	321.75	112.22	123.36	101.44	108	96.24	750 km, flat-topped
**280**	Logistic Type-5	377.27	159.54	166.54	152.53	161	100.00	500 km, flat-topped
**280**	Logistic Type-5	559.74	281.91	294.80	269.02	287	100.00	250 km, pointy-topped
**280**	Logistic Type-5	576.90	281.31	294.14	268.49	289	100.00	250 km, flat-topped
**280**	Logistic Type-5	316.53	110.70	117.18	104.21	114	100.00	750 km, pointy-topped
**280**	Logistic Type-5	419.71	152.43	160.53	144.33	157	100.00	500 km, pointy-topped
**293**	Logistic Type-5	488.76	231.36	242.11	220.61	228	98.55	250 km, flat-topped
**293**	Logistic Type-5	493.20	229.80	240.20	219.39	230	100.00	250 km, pointy-topped
**293**	Logistic Type-5	338.94	128.51	135.65	121.37	130	100.00	500 km, flat-topped
**293**	Logistic Type-4	324.14	96.83	104.51	89.14	98	100.00	750 km, flat-topped
**293**	Logistic Type-5	380.91	127.77	136.31	119.23	130	100.00	500 km, pointy-topped
**293**	Logistic Type-5	322.19	94.65	102.16	87.15	98	100.00	750 km, flat-topped
**293**	Logistic Type-5	307.00	85.80	93.33	78.27	90	100.00	750 km, pointy-topped
**301**	Logistic Type-4	145.70	90.85	108.53	73.19	59	64.94	500 km, pointy-topped
**301**	Logistic Type-3	204.28	132.26	143.65	120.86	87	65.78	250 km, flat-topped
**301**	Logistic Type-4	205.90	129.99	145.96	114.01	87	66.93	250 km, flat-topped
**301**	Logistic Type-5	205.97	122.01	144.45	100.95	87	71.31	250 km, flat-topped
**301**	Logistic Type-5	119.92	68.37	77.91	59.91	58	84.83	500 km, flat-topped
**301**	Logistic Type-5	210.50	106.70	120.52	94.52	92	86.23	250 km, pointy-topped
**301**	Logistic Type-5	96.34	51.87	60.26	44.89	45	86.76	750 km, pointy-topped
**301**	Logistic Type-5	92.32	48.70	53.91	43.99	45	92.41	750 km, flat-topped
**315**	Logistic Type-4	199.27	73.11	81.04	65.18	65	88.91	750 km, flat-topped
**315**	Logistic Type-4	238.18	89.65	99.38	79.94	84	93.69	500 km, pointy-topped
**315**	Logistic Type-5	198.88	68.96	76.85	61.37	65	94.26	750 km, flat-topped
**315**	Logistic Type-5	239.30	87.37	97.61	77.42	84	96.14	500 km, pointy-topped
**315**	Logistic Type-4	347.50	131.76	143.71	119.80	130	98.67	250 km, flat-topped

**315**	Logistic Type-5	225.86	70.99	78.57	63.47	71	100.00	750 km, pointy-topped
**315**	Logistic Type-5	331.57	125.63	135.68	115.60	126	100.00	250 km, pointy-topped
**315**	Logistic Type-5	251.92	86.24	93.48	79.00	87	100.00	500 km, flat-topped
**315**	Logistic Type-5	345.63	127.41	139.04	115.80	130	100.00	250 km, flat-topped
**327**	Gompertz Type-3	94.78	45.99	49.59	42.39	43	93.50	500 km, pointy-topped
**327**	Gompertz Type-3	77.74	34.40	37.13	31.66	34	98.85	750 km, flat-topped
**327**	Gompertz Type-4	153.51	64.56	69.94	59.19	64	99.13	250 km, pointy-topped
**327**	Gompertz Type-4	79.13	34.15	36.96	31.34	34	99.56	750 km, flat-topped
**327**	Gompertz Type-3	152.17	64.23	69.43	59.03	64	99.64	250 km, pointy-topped
**327**	Gompertz Type-4	81.45	34.40	37.65	31.15	35	100.00	750 km, pointy-topped
**327**	Gompertz Type-3	147.35	64.70	69.27	60.13	66	100.00	250 km, flat-topped
**327**	Gompertz Type-4	149.15	64.57	69.24	59.90	66	100.00	250 km, flat-topped
**327**	Gompertz Type-3	107.05	41.75	45.71	37.79	43	100.00	500 km, flat-topped
**327**	Gompertz Type-4	105.89	41.16	44.95	37.38	43	100.00	500 km, flat-topped
**339**	Gompertz Type-4	474.86	210.83	231.23	190.43	172	81.58	250 km, flat-topped
**339**	Gompertz Type-4	470.08	198.97	214.52	183.43	173	86.95	250 km, pointy-topped
**339**	Logistic Type-5	473.52	196.08	215.88	176.30	172	87.72	250 km, flat-topped
**339**	Logistic Type-5	289.78	118.39	132.84	104.41	104	87.84	500 km, pointy-topped
**339**	Logistic Type-4	288.14	115.59	125.64	105.55	104	89.97	500 km, pointy-topped
**339**	Gompertz Type-4	206.51	79.57	87.35	71.79	72	90.49	750 km, pointy-topped
**339**	Gompertz Type-4	296.93	111.60	122.52	100.69	101	90.50	500 km, flat-topped
**339**	Logistic Type-4	473.72	186.77	201.21	172.33	172	92.09	250 km, flat-topped
**339**	Logistic Type-5	206.03	76.47	84.14	68.90	72	94.15	750 km, pointy-topped
**339**	Logistic Type-4	204.72	74.96	81.12	68.79	72	96.06	750 km, pointy-topped
**339**	Gompertz Type-4	229.28	76.48	83.99	68.97	74	96.76	750 km, flat-topped
**354**	Logistic Type-4	161.36	72.02	85.29	58.87	55	76.37	750 km, flat-topped
**354**	Logistic Type-5	162.64	66.21	87.57	52.49	55	83.07	750 km, flat-topped
**354**	Logistic Type-4	219.57	88.09	96.60	79.57	77	87.41	500 km, pointy-topped
**354**	Logistic Type-4	224.43	91.06	99.29	82.84	80	87.85	500 km, flat-topped
**354**	Logistic Type-5	226.13	88.14	101.63	76.58	80	90.77	500 km, flat-topped
**354**	Logistic Type-5	218.19	78.45	87.46	70.41	77	98.15	500 km, pointy-topped
**354**	Logistic Type-5	316.81	122.61	134.42	111.69	122	99.50	250 km, flat-topped
**354**	Logistic Type-5	152.71	56.90	61.37	52.56	57	100.00	750 km, pointy-topped
**354**	Logistic Type-5	318.88	114.58	123.49	105.82	117	100.00	250 km, pointy-topped
**366**	Logistic Type-4	193.80	91.48	97.56	85.39	81	88.55	500 km, flat-topped
**366**	Logistic Type-5	195.64	89.77	98.79	81.12	81	90.23	500 km, flat-topped
**366**	Logistic Type-5	176.42	93.51	102.37	84.86	85	90.90	500 km, pointy-topped
**366**	Logistic Type-4	174.86	92.15	98.08	86.21	85	92.25	500 km, pointy-topped
**366**	Logistic Type-5	152.10	70.31	77.28	63.54	65	92.44	750 km, pointy-topped
**366**	Logistic Type-4	152.59	69.99	74.86	65.13	65	92.86	750 km, flat-topped
**366**	Logistic Type-4	150.18	69.94	75.06	64.82	65	92.94	750 km, pointy-topped
**366**	Logistic Type-5	285.24	146.05	156.91	135.42	137	93.80	250 km, pointy-topped
**366**	Logistic Type-5	154.49	69.25	75.83	62.89	65	93.86	750 km, flat-topped
**366**	Logistic Type-5	276.24	152.46	160.77	144.18	146	95.76	250 km, flat-topped

**375**	Gompertz Type-4	447.05	230.85	248.38	213.33	192	83.17	250 km, flat-topped
**375**	Gompertz Type-4	406.26	230.91	245.28	216.54	195	84.45	250 km, pointy-topped
**375**	Gompertz Type-4	255.30	103.57	112.40	94.74	93	89.79	750 km, pointy-topped
**375**	Gompertz Type-4	289.84	128.10	136.12	120.09	116	90.55	500 km, flat-topped
**375**	Gompertz Type-4	309.76	123.91	133.92	113.90	114	92.00	500 km, pointy-topped
**375**	Gompertz Type-4	258.91	97.72	104.68	90.76	90	92.10	750 km, flat-topped
**385**	Gompertz Type-4	363.46	155.80	165.88	145.72	145	93.07	500 km, flat-topped
**385**	Logistic Type-5	373.06	151.76	163.23	140.32	147	96.86	500 km, pointy-topped
**385**	Logistic Type-4	372.22	148.67	157.86	139.48	147	98.88	500 km, pointy-topped
**385**	Logistic Type-5	364.86	146.47	156.61	136.33	145	99.00	500 km, flat-topped
**385**	Logistic Type-4	364.19	145.12	154.14	136.10	145	99.92	500 km, flat-topped
**385**	Logistic Type-5	272.51	105.63	112.93	98.35	106	100.00	750 km, flat-topped
**385**	Logistic Type-5	508.25	227.00	239.47	214.54	228	100.00	250 km, flat-topped
**385**	Logistic Type-5	284.51	107.31	115.05	99.58	108	100.00	750 km, pointy-topped
**385**	Logistic Type-4	282.72	106.90	113.61	100.19	108	100.00	750 km, pointy-topped
**385**	Logistic Type-5	509.02	221.16	234.88	207.44	224	100.00	250 km, pointy-topped
**385**	Logistic Type-4	271.24	104.22	110.20	98.24	106	100.00	750 km, flat-topped
**400**	Logistic Type-4	262.87	113.64	120.30	106.99	114	100.00	500 km, flat-topped
**400**	Logistic Type-5	341.18	178.81	187.22	170.39	180	100.00	250 km, pointy-topped
**400**	Logistic Type-5	386.20	182.70	191.97	173.44	184	100.00	250 km, flat-topped
**400**	Logistic Type-5	257.18	114.18	119.68	108.69	115	100.00	500 km, pointy-topped
**400**	Logistic Type-4	227.96	95.09	100.84	89.34	96	100.00	750 km, flat-topped
**400**	Logistic Type-4	339.85	177.82	185.59	170.06	180	100.00	250 km, pointy-topped
**400**	Logistic Type-4	217.08	87.89	93.49	82.28	89	100.00	750 km, pointy-topped
**400**	Logistic Type-5	263.08	111.93	118.85	105.01	114	100.00	500 km, flat-topped
**400**	Logistic Type-5	218.61	87.36	93.51	81.22	89	100.00	750 km, pointy-topped
**400**	Logistic Type-4	386.33	180.46	188.98	171.94	184	100.00	250 km, flat-topped
**400**	Logistic Type-5	228.73	93.73	99.72	87.73	96	100.00	750 km, flat-topped
**415**	Gompertz Type-4	379.48	180.44	189.09	171.80	173	95.88	250 km, flat-topped
**415**	Gompertz Type-4	385.37	174.31	184.25	164.38	168	96.38	250 km, pointy-topped
**415**	Logistic Type-5	379.40	177.62	186.96	168.27	173	97.40	250 km, flat-topped
**415**	Gompertz Type-4	215.90	93.31	98.78	87.84	91	97.52	750 km, pointy-topped
**415**	Logistic Type-4	268.19	116.82	123.32	110.32	114	97.59	500 km, pointy-topped
**415**	Logistic Type-5	383.49	170.36	180.56	160.16	168	98.61	250 km, pointy-topped
**415**	Logistic Type-5	218.36	86.19	92.50	79.90	85	98.62	750 km, flat-topped
**415**	Logistic Type-5	215.76	91.66	97.31	86.02	91	99.27	750 km, pointy-topped
**415**	Logistic Type-4	217.46	84.90	90.31	79.49	85	100.00	750 km, flat-topped
**415**	Logistic Type-4	270.46	110.16	116.76	103.56	111	100.00	500 km, flat-topped
**415**	Logistic Type-5	272.24	109.92	116.83	103.01	111	100.00	500 km, flat-topped
**425**	Logistic Type-4	431.91	187.01	199.88	174.14	182	97.32	500 km, pointy-topped
**425**	Logistic Type-4	555.94	312.77	328.50	297.04	305	97.52	250 km, pointy-topped
**425**	Logistic Type-4	565.35	308.38	325.22	291.55	301	97.61	250 km, flat-topped
**425**	Logistic Type-5	567.07	306.34	324.07	288.62	301	98.26	250 km, flat-topped
**425**	Logistic Type-5	557.00	310.05	326.46	293.64	305	98.37	250 km, pointy-topped

**425**	Logistic Type-4	392.75	179.42	190.44	168.39	177	98.65	500 km, flat-topped
**425**	Logistic Type-5	432.53	184.05	197.48	170.62	182	98.89	500 km, pointy-topped
**425**	Logistic Type-5	306.21	120.58	131.20	109.96	120	99.52	750 km, pointy-topped
**425**	Logistic Type-5	391.34	176.22	187.24	165.20	177	100.00	500 km, flat-topped
**425**	Logistic Type-5	318.82	123.17	132.40	113.95	124	100.00	750 km, flat-topped
**436**	Logistic Type-4	396.81	158.67	167.78	149.56	152	95.80	500 km, pointy-topped
**436**	Logistic Type-4	514.02	272.34	283.60	261.08	263	96.57	250 km, pointy-topped
**436**	Logistic Type-5	396.93	155.65	165.32	145.97	152	97.66	500 km, pointy-topped
**436**	Logistic Type-5	514.63	269.29	281.40	257.18	263	97.66	250 km, pointy-topped
**436**	Logistic Type-5	510.50	260.96	272.22	249.70	256	98.10	250 km, flat-topped
**436**	Logistic Type-5	295.70	111.90	119.59	104.22	110	98.30	750 km, pointy-topped
**436**	Logistic Type-4	418.67	154.78	164.80	144.75	154	99.50	500 km, flat-topped
**436**	Logistic Type-4	294.59	110.36	116.97	103.75	110	99.68	750 km, pointy-topped
**436**	Logistic Type-5	420.60	154.24	165.08	143.40	154	99.84	500 km, flat-topped
**436**	Logistic Type-4	316.81	112.49	120.06	104.92	113	100.00	750 km, flat-topped
**436**	Logistic Type-5	317.30	110.99	118.76	103.22	113	100.00	750 km, flat-topped
**449**	Logistic Type-5	398.69	115.14	124.66	105.62	119	100.00	750 km, flat-topped
**449**	Logistic Type-5	418.53	120.69	129.77	111.62	125	100.00	750 km, pointy-topped
**449**	Logistic Type-5	540.52	169.80	182.47	157.14	176	100.00	500 km, flat-topped
**449**	Logistic Type-5	523.72	171.59	183.93	159.25	178	100.00	500 km, pointy-topped
**449**	Logistic Type-5	806.02	315.86	336.16	295.56	328	100.00	250 km, flat-topped
**449**	Logistic Type-5	794.50	321.01	342.25	299.78	335	100.00	250 km, pointy-topped
**456**	Logistic Type-5	246.83	73.98	79.11	68.85	77	100.00	750 km, flat-topped
**456**	Logistic Type-5	490.77	205.80	217.69	193.91	215	100.00	250 km, flat-topped
**456**	Logistic Type-5	271.30	77.12	83.65	70.60	81	100.00	750 km, pointy-topped
**456**	Logistic Type-5	324.32	104.52	112.20	96.84	110	100.00	500 km, flat-topped
**456**	Logistic Type-5	319.24	108.62	116.03	101.21	115	100.00	500 km, pointy-topped
**456**	Logistic Type-5	461.34	188.63	200.16	177.09	200	100.00	250 km, pointy-topped
**465**	Logistic Type-5	504.64	209.24	225.68	192.80	220	100.00	250 km, flat-topped
**465**	Logistic Type-5	521.53	216.39	232.11	200.68	228	100.00	250 km, pointy-topped
**465**	Logistic Type-5	335.11	117.38	126.09	108.67	124	100.00	500 km, flat-topped
**465**	Logistic Type-5	248.76	82.13	88.32	75.93	87	100.00	750 km, pointy-topped
**465**	Logistic Type-5	370.14	120.69	129.45	111.93	128	100.00	500 km, pointy-topped
**465**	Logistic Type-5	266.60	90.35	97.02	83.68	96	100.00	750 km, flat-topped
**475**	Logistic Type-3	193.25	195.07	213.31	176.82	77	39.47	750 km, pointy-topped
**475**	Logistic Type-4	189.00	187.79	213.17	162.41	75	39.94	750 km, flat-topped
**475**	Logistic Type-5	189.62	173.58	NA	NA	75	43.21	750 km, flat-topped
**475**	Logistic Type-3	189.67	166.66	188.47	144.84	75	45.00	750 km, flat-topped
**475**	Logistic Type-4	192.44	166.65	188.07	145.23	77	46.20	750 km, pointy-topped
**475**	Gompertz Type-4	226.54	197.16	203.80	190.52	103	52.24	500 km, pointy-topped
**475**	Logistic Type-5	191.28	133.80	160.03	107.58	77	57.55	750 km, pointy-topped
**475**	Gompertz Type-4	382.24	266.99	287.01	246.97	165	61.80	250 km, flat-topped
**475**	Gompertz Type-4	408.21	255.78	272.16	239.40	164	64.12	250 km, pointy-topped
**475**	Logistic Type-5	216.86	141.51	162.63	120.67	98	69.25	500 km, flat-topped

**475**	Logistic Type-3	383.93	223.03	238.87	207.19	165	73.98	250 km, flat-topped
**482**	Logistic Type-3	354.32	176.29	185.34	167.25	161	91.33	250 km, pointy-topped
**482**	Logistic Type-4	355.00	174.90	184.17	165.64	161	92.05	250 km, pointy-topped
**482**	Logistic Type-5	356.07	171.71	184.13	159.37	161	93.76	250 km, pointy-topped
**482**	Logistic Type-3	252.09	107.74	112.76	102.72	102	94.67	500 km, pointy-topped
**482**	Logistic Type-4	346.05	171.86	179.53	164.19	163	94.84	250 km, flat-topped
**482**	Logistic Type-4	250.34	106.56	111.53	101.59	102	95.72	500 km, pointy-topped
**482**	Logistic Type-5	346.53	169.50	179.07	159.94	163	96.16	250 km, flat-topped
**482**	Logistic Type-5	252.00	105.59	111.83	99.36	102	96.60	500 km, pointy-topped
**482**	Logistic Type-5	185.20	77.71	81.97	73.46	77	99.09	750 km, flat-topped
**482**	Logistic Type-5	172.09	71.00	75.00	67.01	71	100.00	750 km, pointy-topped
**482**	Logistic Type-5	266.66	103.86	110.71	97.04	104	100.00	500 km, flat-topped
**492**	Logistic Type-5	142.38	72.83	78.15	67.68	68	93.37	750 km, pointy-topped
**492**	Logistic Type-5	303.94	139.19	145.94	132.44	137	98.43	250 km, flat-topped
**492**	Logistic Type-5	237.64	94.80	100.60	89.05	94	99.15	500 km, pointy-topped
**492**	Logistic Type-5	141.69	74.44	77.12	71.76	74	99.41	750 km, flat-topped
**492**	Logistic Type-5	311.74	145.78	151.85	139.72	145	99.46	250 km, pointy-topped
**492**	Logistic Type-5	216.96	93.45	97.40	89.50	94	100.00	500 km, flat-topped
**505**	Logistic Type-5	375.02	203.14	212.31	193.97	200	98.46	250 km, pointy-topped
**505**	Logistic Type-5	213.23	89.44	93.69	85.20	89	99.50	750 km, flat-topped
**505**	Logistic Type-5	200.91	87.43	90.90	83.96	88	100.00	750 km, pointy-topped
**505**	Logistic Type-5	375.49	194.67	202.69	186.66	197	100.00	250 km, flat-topped
**505**	Logistic Type-5	308.75	122.00	127.15	116.85	124	100.00	500 km, pointy-topped
**505**	Logistic Type-5	320.22	128.62	134.87	122.37	132	100.00	500 km, flat-topped
**515**	Logistic Type-4	214.53	86.58	92.17	81.00	86	99.33	750 km, flat-topped
**515**	Logistic Type-5	345.62	205.93	213.91	197.95	208	100.00	250 km, flat-topped
**515**	Logistic Type-5	235.11	118.75	124.65	112.84	121	100.00	500 km, pointy-topped
**515**	Logistic Type-4	345.73	203.97	211.36	196.58	208	100.00	250 km, flat-topped
**515**	Logistic Type-4	359.06	203.89	212.13	195.65	209	100.00	250 km, pointy-topped
**515**	Logistic Type-5	360.86	203.47	212.23	194.71	209	100.00	250 km, pointy-topped
**515**	Logistic Type-5	203.57	91.32	96.42	86.22	94	100.00	750 km, pointy-topped
**515**	Logistic Type-5	262.34	120.47	126.31	114.63	124	100.00	500 km, flat-topped
**530**	Gompertz Type-4	98.95	42.58	61.93	32.31	38	89.24	500 km, pointy-topped
**530**	Logistic Type-4	99.15	39.73	46.45	33.29	38	95.64	500 km, pointy-topped
**530**	Gompertz Type-3	97.92	39.44	44.99	33.88	38	96.36	500 km, pointy-topped
**530**	Gompertz Type-3	104.26	42.13	47.55	36.72	41	97.31	500 km, flat-topped
**530**	Gompertz Type-3	133.07	56.28	63.69	48.87	55	97.73	250 km, flat-topped
**530**	Gompertz Type-4	105.68	40.98	46.94	35.11	41	100.00	500 km, flat-topped
**530**	Logistic Type-4	105.96	39.87	45.61	34.26	41	100.00	500 km, flat-topped
**530**	Logistic Type-3	81.01	31.07	34.19	27.95	32	100.00	750 km, flat-topped
**530**	Gompertz Type-4	132.39	53.27	60.42	46.15	55	100.00	250 km, flat-topped
**530**	Logistic Type-3	80.67	30.80	34.12	27.48	32	100.00	750 km, pointy-topped
**530**	Logistic Type-4	124.06	48.11	53.31	42.92	50	100.00	250 km, pointy-topped
**530**	Logistic Type-3	122.20	47.81	52.48	43.14	50	100.00	250 km, pointy-topped

**530**	Logistic Type-4	82.37	30.54	34.10	26.99	32	100.00	750 km, pointy-topped
**530**	Logistic Type-3	104.91	38.88	43.62	34.13	41	100.00	500 km, flat-topped
**530**	Logistic Type-4	133.92	51.77	58.68	44.89	55	100.00	250 km, flat-topped
**530**	Logistic Type-3	132.06	51.32	57.46	45.17	55	100.00	250 km, flat-topped

Table S12.

Results for the logistic and logistic-like (Gompertz) models estimating fossil data coverage of marine regions (not shallow-water only) based on collection history for the 49-bin approach. Predicted completeness refers to the percentage of the observed number of grid cells containing fossil data relative to the predicted value for 2100 (see Methods for details).

Map age (Myr)	Model	AIC	Predicted no. cells	Upper CI	Lower CI	Observed no. cells with fossil data	Predicted completeness	Grid type
**3**	Gompertz Type-4	1055.24	830.86	853.06	808.67	528	63.55	250 km, pointy-topped
**3**	Gompertz Type-4	986.80	816.64	838.97	794.31	532	65.14	250 km, flat-topped
**3**	Gompertz Type-4	768.84	454.64	471.89	437.39	302	66.43	500 km, flat-topped
**3**	Gompertz Type-4	807.52	427.21	444.40	410.01	303	70.93	500 km, pointy-topped
**3**	Gompertz Type-4	603.57	294.23	307.51	280.95	215	73.07	750 km, pointy-topped
**3**	Logistic Type-5	769.28	398.45	427.28	369.61	302	75.79	500 km, flat-topped
**3**	Logistic Type-4	769.11	388.39	407.82	368.96	302	77.76	500 km, flat-topped
**3**	Logistic Type-5	806.48	388.68	408.60	368.76	303	77.96	500 km, pointy-topped
**3**	Logistic Type-5	604.57	272.56	287.05	258.08	215	78.88	750 km, pointy-topped
**3**	Logistic Type-3	768.70	381.31	396.24	366.38	302	79.20	500 km, flat-topped
**3**	Logistic Type-3	607.26	259.85	269.06	250.64	214	82.36	750 km, flat-topped
**3**	Logistic Type-4	608.60	257.23	268.25	246.21	214	83.19	750 km, flat-topped
**15**	Gompertz Type-3	880.85	581.41	597.58	565.24	362	62.26	250 km, flat-topped
**15**	Gompertz Type-4	882.81	581.02	597.75	564.30	362	62.30	250 km, flat-topped
**15**	Gompertz Type-3	873.85	565.28	582.91	547.65	371	65.63	250 km, pointy-topped
**15**	Gompertz Type-4	872.90	551.51	570.47	532.56	371	67.27	250 km, pointy-topped
**15**	Gompertz Type-3	610.30	304.93	316.26	293.60	208	68.21	500 km, pointy-topped
**15**	Gompertz Type-4	609.22	298.00	310.93	285.07	208	69.80	500 km, pointy-topped
**15**	Gompertz Type-4	498.70	198.56	210.63	186.49	147	74.03	750 km, flat-topped
**15**	Gompertz Type-4	448.41	196.11	205.24	186.98	148	75.47	750 km, pointy-topped
**15**	Logistic Type-5	448.92	184.26	193.45	175.06	148	80.32	750 km, pointy-topped
**15**	Gompertz Type-4	646.85	253.63	265.74	241.53	206	81.22	500 km, flat-topped
**26**	Logistic Type-4	325.22	183.69	NA	NA	95	51.72	750 km, flat-topped
**26**	Gompertz Type-4	326.48	177.11	NA	NA	95	53.64	750 km, flat-topped
**26**	Gompertz Type-4	459.79	239.29	253.32	225.26	130	54.33	500 km, pointy-topped
**26**	Gompertz Type-4	433.86	220.35	NA	NA	121	54.91	500 km, flat-topped
**26**	Gompertz Type-4	616.78	352.71	364.31	341.11	200	56.70	250 km, pointy-topped
**26**	Gompertz Type-3	459.85	226.10	234.68	217.53	130	57.50	500 km, pointy-topped
**26**	Gompertz Type-4	619.91	349.67	361.80	337.54	205	58.63	250 km, flat-topped
**26**	Gompertz Type-3	616.86	335.38	347.54	323.22	200	59.63	250 km, pointy-topped
**26**	Gompertz Type-3	619.30	340.28	353.31	327.25	205	60.24	250 km, flat-topped
**26**	Gompertz Type-3	332.76	134.18	142.17	126.19	94	70.05	750 km, pointy-topped
**26**	Gompertz Type-4	334.75	133.87	142.33	125.41	94	70.22	750 km, pointy-topped
**36**	Gompertz Type-4	546.19	281.96	289.10	274.81	219	77.67	250 km, pointy-topped

**36**	Gompertz Type-4	292.12	129.54	134.44	124.64	101	77.97	750 km, flat-topped
**36**	Gompertz Type-4	517.25	275.82	282.52	269.11	216	78.31	250 km, flat-topped
**36**	Gompertz Type-3	303.61	125.53	130.17	120.89	102	81.25	750 km, pointy-topped
**36**	Gompertz Type-4	390.90	164.90	169.47	160.33	135	81.87	500 km, flat-topped
**36**	Gompertz Type-4	301.69	123.30	128.10	118.50	102	82.73	750 km, pointy-topped
**36**	Logistic Type-5	351.75	160.74	167.24	154.25	136	84.61	500 km, pointy-topped
**36**	Logistic Type-4	352.73	156.38	160.09	152.68	136	86.97	500 km, pointy-topped
**36**	Logistic Type-3	351.39	155.62	158.65	152.59	136	87.39	500 km, pointy-topped
**52**	Gompertz Type-3	284.51	162.28	166.49	158.08	123	75.79	500 km, flat-topped
**52**	Gompertz Type-3	348.18	164.92	171.59	158.25	126	76.40	500 km, pointy-topped
**52**	Gompertz Type-4	350.17	164.69	171.96	157.41	126	76.51	500 km, pointy-topped
**52**	Gompertz Type-4	284.22	160.56	165.10	156.03	123	76.61	500 km, flat-topped
**52**	Gompertz Type-3	511.44	252.56	262.70	242.42	195	77.21	250 km, flat-topped
**52**	Gompertz Type-4	541.27	262.32	273.47	251.18	204	77.77	250 km, pointy-topped
**52**	Gompertz Type-4	510.34	247.67	258.23	237.11	195	78.73	250 km, flat-topped
**52**	Gompertz Type-4	258.37	109.99	113.56	106.41	96	87.28	750 km, flat-topped
**52**	Logistic Type-5	271.53	109.79	115.51	104.07	98	89.26	750 km, pointy-topped
**52**	Logistic Type-4	271.43	106.03	109.77	102.29	98	92.42	750 km, pointy-topped
**52**	Logistic Type-3	270.98	104.76	107.69	101.84	98	93.54	750 km, pointy-topped
**61**	Gompertz Type-4	260.87	199.18	NA	NA	95	47.70	750 km, flat-topped
**61**	Gompertz Type-3	260.16	190.80	196.95	184.65	95	49.79	750 km, flat-topped
**61**	Logistic Type-4	261.77	176.22	196.21	156.22	95	53.91	750 km, flat-topped
**61**	Gompertz Type-3	341.20	243.25	250.37	236.13	133	54.68	500 km, pointy-topped
**61**	Gompertz Type-4	343.01	239.34	247.07	231.61	133	55.57	500 km, pointy-topped
**61**	Gompertz Type-3	242.55	174.09	179.12	169.06	100	57.44	750 km, pointy-topped
**61**	Gompertz Type-3	451.94	325.00	337.38	312.61	190	58.46	250 km, flat-topped
**61**	Gompertz Type-3	459.98	327.53	338.96	316.09	192	58.62	250 km, pointy-topped
**61**	Gompertz Type-4	461.52	323.67	336.65	310.70	192	59.32	250 km, pointy-topped
**61**	Gompertz Type-4	452.54	319.01	332.42	305.61	190	59.56	250 km, flat-topped
**61**	Gompertz Type-4	284.50	192.15	198.09	186.22	119	61.93	500 km, flat-topped
**61**	Gompertz Type-3	282.59	190.29	196.31	184.28	119	62.54	500 km, flat-topped
**61**	Logistic Type-4	341.21	194.46	209.87	179.04	133	68.40	500 km, pointy-topped
**61**	Logistic Type-4	244.12	145.74	156.96	134.52	100	68.62	750 km, pointy-topped
**69**	Logistic Type-5	509.50	274.86	295.96	255.59	249	90.59	250 km, flat-topped
**69**	Logistic Type-5	382.50	168.74	181.44	157.34	154	91.26	500 km, pointy-topped
**69**	Logistic Type-5	286.14	120.11	128.01	112.61	110	91.58	750 km, pointy-topped
**69**	Logistic Type-5	386.75	164.00	172.09	156.31	155	94.51	500 km, flat-topped
**69**	Logistic Type-5	253.37	110.22	114.38	106.21	107	97.08	750 km, flat-topped
**69**	Logistic Type-5	508.76	243.36	251.66	235.42	237	97.39	250 km, pointy-topped
**75**	Gompertz Type-3	321.63	330.32	NA	NA	125	37.84	750 km, pointy-topped
**75**	Gompertz Type-3	391.62	361.65	370.41	352.89	160	44.24	500 km, flat-topped
**75**	Logistic Type-4	320.81	261.98	286.54	237.42	125	47.71	750 km, pointy-topped
**75**	Gompertz Type-3	421.94	340.73	349.83	331.62	165	48.43	500 km, pointy-topped
**75**	Logistic Type-5	320.05	233.93	308.63	160.85	125	53.43	750 km, pointy-topped

**75**	Logistic Type-4	391.07	298.63	330.04	267.22	160	53.58	500 km, flat-topped
**75**	Logistic Type-5	392.75	277.02	327.99	226.05	160	57.76	500 km, flat-topped
**75**	Logistic Type-4	332.07	192.64	214.33	170.94	122	63.33	750 km, flat-topped
**75**	Logistic Type-5	420.23	255.17	290.99	219.36	165	64.66	500 km, pointy-topped
**75**	Logistic Type-5	333.59	185.04	209.69	160.39	122	65.93	750 km, flat-topped
**75**	Logistic Type-3	332.14	180.85	194.10	167.59	122	67.46	750 km, flat-topped
**75**	Logistic Type-3	420.83	242.28	259.80	224.75	165	68.10	500 km, pointy-topped
**75**	Logistic Type-4	613.77	382.35	405.64	359.06	272	71.14	250 km, pointy-topped
**75**	Logistic Type-5	613.03	347.77	378.80	316.75	272	78.21	250 km, pointy-topped
**75**	Logistic Type-5	601.97	320.99	344.62	297.83	277	86.30	250 km, flat-topped
**87**	Logistic Type-5	627.67	344.42	352.78	336.07	333	96.69	250 km, flat-topped
**87**	Logistic Type-5	664.24	330.19	338.03	322.37	323	97.82	250 km, pointy-topped
**87**	Logistic Type-5	373.64	141.32	145.42	137.25	139	98.36	750 km, pointy-topped
**87**	Logistic Type-5	491.47	192.93	197.90	187.99	191	99.00	500 km, pointy-topped
**87**	Logistic Type-5	354.60	135.06	139.00	131.28	134	99.22	750 km, flat-topped
**87**	Logistic Type-5	446.17	190.57	194.50	186.66	191	100.00	500 km, flat-topped
**97**	Logistic Type-5	397.68	187.41	195.30	179.58	179	95.51	500 km, pointy-topped
**97**	Logistic Type-5	306.66	130.50	134.38	126.63	127	97.32	750 km, flat-topped
**97**	Logistic Type-5	589.31	294.68	301.94	287.43	294	99.77	250 km, pointy-topped
**97**	Logistic Type-5	294.75	137.21	140.35	134.08	137	99.84	750 km, pointy-topped
**97**	Logistic Type-5	571.34	283.46	291.25	275.72	284	100.00	250 km, flat-topped
**97**	Logistic Type-5	380.36	164.77	167.78	161.77	167	100.00	500 km, flat-topped
**107**	Logistic Type-5	394.52	188.82	196.68	180.96	176	93.21	500 km, flat-topped
**107**	Logistic Type-5	515.49	312.90	322.38	303.42	292	93.32	250 km, pointy-topped
**107**	Logistic Type-5	552.88	320.86	331.91	309.82	301	93.81	250 km, flat-topped
**107**	Logistic Type-5	356.55	141.93	146.54	137.35	138	97.23	750 km, pointy-topped
**107**	Logistic Type-5	372.87	182.78	187.20	178.38	178	97.38	500 km, pointy-topped
**107**	Logistic Type-5	345.40	130.21	133.39	127.05	131	100.00	750 km, flat-topped
**116**	Logistic Type-3	188.74	125.45	136.24	114.66	87	69.35	750 km, flat-topped
**116**	Logistic Type-4	189.18	120.72	132.58	108.87	87	72.07	750 km, flat-topped
**116**	Logistic Type-3	260.50	142.27	148.50	136.04	114	80.13	500 km, flat-topped
**116**	Logistic Type-4	260.48	139.48	146.39	132.56	114	81.73	500 km, flat-topped
**116**	Logistic Type-4	280.21	139.48	146.77	132.19	116	83.17	500 km, pointy-topped
**116**	Logistic Type-5	260.58	132.07	142.81	121.48	114	86.32	500 km, flat-topped
**116**	Logistic Type-5	280.15	132.52	144.38	121.17	116	87.53	500 km, pointy-topped
**116**	Logistic Type-5	222.71	103.14	113.16	94.17	92	89.20	750 km, pointy-topped
**116**	Logistic Type-4	444.02	208.38	215.66	201.11	186	89.26	250 km, pointy-topped
**116**	Logistic Type-5	443.60	203.87	213.81	193.92	186	91.24	250 km, pointy-topped
**116**	Logistic Type-5	365.58	187.72	196.32	179.16	176	93.76	250 km, flat-topped
**127**	Gompertz Type-4	223.71	108.65	115.39	101.90	89	81.92	500 km, flat-topped
**127**	Gompertz Type-4	165.85	81.45	86.36	76.54	69	84.72	750 km, pointy-topped
**127**	Logistic Type-5	222.22	100.51	107.62	93.44	89	88.55	500 km, flat-topped
**127**	Logistic Type-5	165.83	77.30	83.04	71.65	69	89.27	750 km, pointy-topped
**127**	Gompertz Type-4	323.93	149.17	155.82	142.53	134	89.83	250 km, flat-topped

**127**	Logistic Type-4	222.54	96.72	100.93	92.51	89	92.02	500 km, flat-topped
**127**	Logistic Type-5	323.09	143.28	150.37	136.20	134	93.52	250 km, flat-topped
**127**	Logistic Type-5	300.82	141.87	148.82	134.92	134	94.46	250 km, pointy-topped
**127**	Logistic Type-5	187.94	89.92	94.22	85.64	85	94.53	500 km, pointy-topped
**127**	Logistic Type-5	158.48	68.36	71.48	65.27	66	96.55	750 km, flat-topped
**127**	Logistic Type-4	300.48	138.52	142.69	134.35	134	96.74	250 km, pointy-topped
**127**	Logistic Type-4	187.95	87.84	90.39	85.28	85	96.77	500 km, pointy-topped
**127**	Logistic Type-4	156.70	67.90	70.05	65.74	66	97.21	750 km, flat-topped
**136**	Logistic Type-4	241.36	120.65	125.05	116.24	110	91.18	500 km, flat-topped
**136**	Logistic Type-5	240.42	117.46	124.21	110.94	110	93.65	500 km, flat-topped
**136**	Logistic Type-4	325.16	157.93	162.69	153.18	148	93.71	250 km, flat-topped
**136**	Logistic Type-5	260.17	116.49	122.45	110.70	111	95.29	500 km, pointy-topped
**136**	Logistic Type-4	236.45	89.17	93.42	84.91	85	95.33	750 km, pointy-topped
**136**	Logistic Type-5	324.87	154.62	161.09	148.22	148	95.72	250 km, flat-topped
**136**	Logistic Type-4	234.69	95.64	98.51	92.78	92	96.19	750 km, flat-topped
**136**	Logistic Type-5	342.84	169.96	176.23	163.73	164	96.49	250 km, pointy-topped
**136**	Logistic Type-5	236.33	95.21	99.78	90.72	92	96.63	750 km, flat-topped
**145**	Logistic Type-5	380.24	158.74	166.65	150.85	146	91.97	250 km, pointy-topped
**145**	Logistic Type-5	385.76	154.74	162.39	147.10	144	93.06	250 km, flat-topped
**145**	Logistic Type-4	384.28	152.23	157.21	147.25	144	94.59	250 km, flat-topped
**145**	Logistic Type-5	297.86	104.48	111.37	97.74	99	94.76	500 km, flat-topped
**145**	Logistic Type-4	380.05	153.97	158.67	149.28	146	94.82	250 km, pointy-topped
**145**	Logistic Type-4	264.78	106.41	109.63	103.20	101	94.91	500 km, pointy-topped
**145**	Logistic Type-4	296.05	103.37	108.28	98.46	99	95.78	500 km, flat-topped
**145**	Logistic Type-5	228.30	78.52	82.26	74.80	76	96.79	750 km, flat-topped
**145**	Logistic Type-4	229.18	77.59	80.91	74.27	76	97.95	750 km, pointy-topped
**145**	Logistic Type-4	226.88	77.55	80.20	74.89	76	98.00	750 km, flat-topped
**145**	Logistic Type-5	230.52	76.89	80.74	73.11	76	98.84	750 km, pointy-topped
**155**	Logistic Type-5	561.23	296.12	303.28	288.97	290	97.93	250 km, pointy-topped
**155**	Logistic Type-5	567.32	287.84	294.10	281.58	284	98.67	250 km, flat-topped
**155**	Logistic Type-5	427.98	174.21	178.97	169.45	172	98.73	500 km, flat-topped
**155**	Logistic Type-5	410.84	167.16	171.44	162.89	166	99.31	500 km, pointy-topped
**155**	Logistic Type-5	281.71	120.62	124.44	116.82	120	99.49	750 km, flat-topped
**155**	Logistic Type-5	311.01	121.51	125.55	117.57	122	100.00	750 km, pointy-topped
**168**	Logistic Type-5	286.33	133.79	137.61	129.99	131	97.92	500 km, pointy-topped
**168**	Logistic Type-5	413.29	219.14	223.98	214.30	215	98.11	250 km, pointy-topped
**168**	Logistic Type-5	428.35	208.27	213.28	203.27	205	98.43	250 km, flat-topped
**168**	Logistic Type-5	205.65	91.89	94.44	89.36	92	100.00	750 km, pointy-topped
**168**	Logistic Type-5	262.86	128.38	130.89	125.87	129	100.00	500 km, flat-topped
**168**	Logistic Type-5	228.71	93.21	95.37	91.12	95	100.00	750 km, flat-topped
**178**	Logistic Type-4	237.33	114.23	118.71	109.74	102	89.30	500 km, flat-topped
**178**	Logistic Type-4	227.60	109.03	112.93	105.13	100	91.72	500 km, pointy-topped
**178**	Logistic Type-5	238.34	110.90	119.03	103.19	102	91.98	500 km, flat-topped
**178**	Logistic Type-5	182.67	83.26	89.85	77.14	77	92.48	750 km, flat-topped

**178**	Logistic Type-5	167.46	79.86	82.42	77.34	80	100.00	750 km, pointy-topped
**178**	Logistic Type-5	330.80	160.94	165.04	156.93	162	100.00	250 km, pointy-topped
**178**	Logistic Type-5	323.71	159.57	163.69	155.55	161	100.00	250 km, flat-topped
**191**	Gompertz Type-4	264.44	124.99	131.34	118.64	107	85.61	750 km, flat-topped
**191**	Logistic Type-5	226.55	118.81	125.29	112.40	111	93.43	750 km, pointy-topped
**191**	Logistic Type-4	343.63	174.33	178.96	169.70	166	95.22	500 km, flat-topped
**191**	Logistic Type-4	226.08	115.59	119.28	111.91	111	96.03	750 km, pointy-topped
**191**	Logistic Type-5	342.68	169.86	176.37	163.47	166	97.73	500 km, flat-topped
**191**	Logistic Type-5	338.36	157.51	162.48	152.72	157	99.67	500 km, pointy-topped
**191**	Logistic Type-5	483.21	275.50	279.46	271.53	281	100.00	250 km, flat-topped
**191**	Logistic Type-5	514.15	286.65	291.62	281.71	293	100.00	250 km, pointy-topped
**201**	Logistic Type-5	111.33	43.57	46.85	40.74	42	96.41	750 km, flat-topped
**201**	Logistic Type-5	174.45	68.03	71.17	65.13	68	99.95	250 km, pointy-topped
**201**	Logistic Type-5	92.50	37.84	39.67	36.25	38	100.00	750 km, pointy-topped
**201**	Logistic Type-5	143.47	48.58	51.24	46.25	49	100.00	500 km, pointy-topped
**201**	Logistic Type-5	113.30	47.41	49.14	45.79	48	100.00	500 km, flat-topped
**201**	Logistic Type-5	198.45	74.39	77.07	71.92	76	100.00	250 km, flat-topped
**213**	Logistic Type-5	316.96	142.53	147.33	137.99	144	100.00	750 km, pointy-topped
**213**	Logistic Type-5	411.86	199.59	205.07	194.25	202	100.00	500 km, pointy-topped
**213**	Logistic Type-5	307.13	141.93	146.03	138.03	144	100.00	750 km, flat-topped
**213**	Logistic Type-5	581.48	337.53	344.74	330.41	344	100.00	250 km, pointy-topped
**213**	Logistic Type-5	439.11	207.52	212.86	202.33	212	100.00	500 km, flat-topped
**213**	Logistic Type-5	569.81	305.29	310.57	300.07	312	100.00	250 km, flat-topped
**232**	Logistic Type-3	315.57	277.56	376.33	180.75	125	45.03	500 km, pointy-topped
**232**	Logistic Type-4	317.03	247.44	352.75	156.30	125	50.52	500 km, pointy-topped
**232**	Logistic Type-5	317.52	222.78	407.64	131.99	125	56.11	500 km, pointy-topped
**232**	Logistic Type-3	270.08	152.98	194.58	112.38	94	61.45	750 km, pointy-topped
**232**	Logistic Type-5	268.15	98.93	113.90	88.34	94	95.02	750 km, pointy-topped
**232**	Logistic Type-5	350.46	137.66	149.04	129.74	136	98.79	500 km, flat-topped
**232**	Logistic Type-5	250.55	95.98	100.16	92.11	97	100.00	750 km, flat-topped
**232**	Logistic Type-5	458.73	210.47	217.18	204.24	213	100.00	250 km, pointy-topped
**232**	Logistic Type-5	432.60	191.81	196.93	186.93	195	100.00	250 km, flat-topped
**245**	Logistic Type-4	399.62	145.07	151.82	138.33	138	95.12	500 km, pointy-topped
**245**	Logistic Type-5	543.17	231.70	237.73	225.79	235	100.00	250 km, flat-topped
**245**	Logistic Type-5	284.05	95.59	99.77	91.62	97	100.00	750 km, flat-topped
**245**	Logistic Type-5	376.83	135.85	140.16	131.63	138	100.00	500 km, flat-topped
**245**	Logistic Type-5	595.93	240.09	246.93	233.37	244	100.00	250 km, pointy-topped
**245**	Logistic Type-5	282.04	98.61	102.29	95.16	101	100.00	750 km, pointy-topped
**252**	Logistic Type-5	285.08	88.91	94.90	83.92	88	98.98	750 km, flat-topped
**252**	Logistic Type-5	282.10	86.71	93.51	80.75	86	99.18	750 km, pointy-topped
**252**	Logistic Type-5	469.57	201.07	206.25	195.92	203	100.00	250 km, flat-topped
**252**	Logistic Type-5	346.43	115.80	120.92	110.89	117	100.00	500 km, flat-topped
**252**	Logistic Type-5	336.81	117.71	122.19	113.51	119	100.00	500 km, pointy-topped
**252**	Logistic Type-5	489.52	189.43	195.06	183.90	193	100.00	250 km, pointy-topped

**256**	Logistic Type-5	435.02	138.83	144.04	133.83	141	100.00	500 km, flat-topped
**256**	Logistic Type-5	334.13	100.69	105.15	96.53	103	100.00	750 km, flat-topped
**256**	Logistic Type-5	406.35	132.61	137.72	127.77	136	100.00	500 km, pointy-topped
**256**	Logistic Type-5	334.43	96.68	100.57	93.12	100	100.00	750 km, pointy-topped
**256**	Logistic Type-5	646.87	233.37	239.24	227.56	242	100.00	250 km, flat-topped
**256**	Logistic Type-5	650.14	228.28	234.77	221.92	237	100.00	250 km, pointy-topped
**265**	Logistic Type-5	341.06	137.31	142.90	131.95	138	100.00	750 km, flat-topped
**265**	Logistic Type-5	448.32	200.95	204.23	197.66	204	100.00	500 km, pointy-topped
**265**	Logistic Type-5	318.91	128.44	132.99	124.17	131	100.00	750 km, pointy-topped
**265**	Logistic Type-5	630.61	359.09	364.48	353.71	367	100.00	250 km, pointy-topped
**265**	Logistic Type-5	429.39	192.67	196.38	188.97	197	100.00	500 km, flat-topped
**265**	Logistic Type-5	657.65	357.74	362.66	352.82	368	100.00	250 km, flat-topped
**280**	Logistic Type-5	397.91	165.47	168.88	162.07	168	100.00	500 km, flat-topped
**280**	Logistic Type-5	578.72	293.33	299.07	287.59	300	100.00	250 km, pointy-topped
**280**	Logistic Type-5	609.42	294.16	299.15	289.17	303	100.00	250 km, flat-topped
**280**	Logistic Type-5	302.18	109.87	112.72	107.06	114	100.00	750 km, flat-topped
**280**	Logistic Type-5	427.13	157.02	160.44	153.64	163	100.00	500 km, pointy-topped
**280**	Logistic Type-5	320.15	113.65	116.48	110.88	118	100.00	750 km, pointy-topped
**293**	Logistic Type-5	536.50	263.12	270.27	255.98	258	98.05	250 km, flat-topped
**293**	Logistic Type-5	510.20	255.53	261.18	249.89	255	99.79	250 km, pointy-topped
**293**	Logistic Type-5	389.25	148.16	152.56	143.81	149	100.00	500 km, flat-topped
**293**	Logistic Type-5	422.63	145.57	150.49	140.74	148	100.00	500 km, pointy-topped
**293**	Logistic Type-5	364.86	110.28	114.16	106.48	114	100.00	750 km, flat-topped
**293**	Logistic Type-5	335.92	102.24	105.58	98.99	107	100.00	750 km, pointy-topped
**301**	Logistic Type-4	134.16	86.19	92.71	79.67	65	75.41	500 km, flat-topped
**301**	Logistic Type-5	134.77	83.36	97.07	71.05	65	77.97	500 km, flat-topped
**301**	Logistic Type-5	95.10	60.15	NA	NA	49	81.46	750 km, flat-topped
**301**	Logistic Type-5	227.17	111.20	119.17	103.32	97	87.23	250 km, flat-topped
**301**	Logistic Type-5	100.53	54.11	61.81	48.27	49	90.56	750 km, pointy-topped
**301**	Logistic Type-5	151.40	65.07	69.38	61.25	63	96.82	500 km, pointy-topped
**301**	Logistic Type-5	228.51	104.06	107.32	100.94	103	98.98	250 km, pointy-topped
**315**	Logistic Type-5	442.40	176.59	185.93	167.36	166	94.00	250 km, pointy-topped
**315**	Logistic Type-5	274.34	92.79	101.44	85.27	88	94.84	750 km, pointy-topped
**315**	Logistic Type-5	460.28	176.23	186.22	166.49	169	95.90	250 km, flat-topped
**315**	Logistic Type-5	337.65	114.10	121.54	107.29	110	96.41	500 km, pointy-topped
**315**	Logistic Type-5	263.05	82.23	88.26	77.22	80	97.29	750 km, flat-topped
**315**	Logistic Type-5	326.45	113.64	119.34	108.26	111	97.68	500 km, flat-topped
**327**	Gompertz Type-4	129.65	66.00	68.82	63.19	61	92.42	500 km, pointy-topped
**327**	Gompertz Type-3	116.89	50.32	52.96	47.69	48	95.38	750 km, flat-topped
**327**	Gompertz Type-4	117.66	50.92	53.86	47.99	49	96.22	750 km, pointy-topped
**327**	Gompertz Type-3	203.17	89.28	93.26	85.29	86	96.33	250 km, pointy-topped
**327**	Gompertz Type-4	204.32	88.83	92.85	84.80	86	96.82	250 km, pointy-topped
**327**	Gompertz Type-4	115.00	49.44	51.94	46.94	48	97.09	750 km, flat-topped
**327**	Gompertz Type-3	229.09	90.65	94.52	86.77	90	99.29	250 km, flat-topped

**327**	Gompertz Type-4	173.82	62.22	66.33	58.12	62	99.65	500 km, flat-topped
**327**	Gompertz Type-4	227.17	89.68	93.37	86.00	90	100.00	250 km, flat-topped
**339**	Gompertz Type-4	491.47	236.75	249.61	223.88	202	85.32	250 km, pointy-topped
**339**	Logistic Type-5	493.07	232.31	243.79	220.84	202	86.95	250 km, pointy-topped
**339**	Logistic Type-4	505.02	219.18	227.87	210.50	200	91.25	250 km, flat-topped
**339**	Logistic Type-4	306.91	124.95	130.57	119.33	116	92.84	500 km, pointy-topped
**339**	Gompertz Type-4	270.56	89.44	95.21	83.67	84	93.92	750 km, flat-topped
**339**	Logistic Type-4	316.99	122.71	128.03	117.40	116	94.53	500 km, flat-topped
**339**	Logistic Type-5	308.09	122.40	129.91	115.18	116	94.77	500 km, pointy-topped
**339**	Logistic Type-4	248.00	87.56	91.39	83.73	84	95.93	750 km, pointy-topped
**339**	Logistic Type-5	249.29	86.40	91.00	81.91	84	97.23	750 km, pointy-topped
**339**	Logistic Type-4	272.26	85.85	90.33	81.38	84	97.84	750 km, flat-topped
**354**	Gompertz Type-4	178.37	89.56	108.25	70.87	59	65.88	750 km, flat-topped
**354**	Gompertz Type-4	248.09	111.02	120.50	101.53	88	79.27	500 km, flat-topped
**354**	Gompertz Type-4	239.49	105.89	114.96	96.83	84	79.33	500 km, pointy-topped
**354**	Logistic Type-4	176.72	71.52	80.40	62.66	59	82.49	750 km, flat-topped
**354**	Logistic Type-5	248.87	102.39	112.88	92.18	88	85.95	500 km, flat-topped
**354**	Logistic Type-4	170.84	73.16	78.19	68.13	63	86.11	750 km, pointy-topped
**354**	Logistic Type-4	344.52	151.80	159.20	144.41	136	89.59	250 km, flat-topped
**354**	Logistic Type-4	248.06	97.28	102.88	91.67	88	90.46	500 km, flat-topped
**354**	Logistic Type-5	240.14	92.75	106.42	82.22	84	90.57	500 km, pointy-topped
**354**	Logistic Type-4	238.27	92.08	97.21	86.95	84	91.22	500 km, pointy-topped
**354**	Logistic Type-4	355.32	139.01	144.65	133.38	130	93.52	250 km, pointy-topped
**354**	Logistic Type-5	168.89	66.60	72.44	61.59	63	94.59	750 km, pointy-topped
**354**	Logistic Type-5	345.13	143.61	156.14	132.93	136	94.70	250 km, flat-topped
**354**	Logistic Type-5	355.72	132.75	142.42	124.50	130	97.93	250 km, pointy-topped
**366**	Gompertz Type-4	161.29	82.83	90.43	75.23	69	83.30	750 km, pointy-topped
**366**	Gompertz Type-4	183.51	108.01	114.35	101.68	91	84.25	500 km, pointy-topped
**366**	Logistic Type-5	162.07	76.85	84.71	69.52	69	89.79	750 km, pointy-topped
**366**	Logistic Type-5	184.65	100.63	107.68	93.69	91	90.43	500 km, pointy-topped
**366**	Logistic Type-5	161.33	74.93	82.53	67.96	68	90.75	750 km, flat-topped
**366**	Logistic Type-4	204.44	94.57	98.80	90.35	86	90.94	500 km, flat-topped
**366**	Logistic Type-4	291.19	171.42	176.66	166.17	156	91.00	250 km, flat-topped
**366**	Logistic Type-4	306.98	158.11	163.49	152.73	144	91.08	250 km, pointy-topped
**366**	Logistic Type-4	159.46	74.35	78.78	69.92	68	91.46	750 km, flat-topped
**366**	Logistic Type-5	206.40	94.00	101.42	87.01	86	91.49	500 km, flat-topped
**366**	Logistic Type-4	161.04	74.68	79.32	70.03	69	92.40	750 km, pointy-topped
**366**	Logistic Type-4	185.31	96.89	100.88	92.91	91	93.92	500 km, pointy-topped
**366**	Logistic Type-5	306.56	152.55	160.37	144.96	144	94.39	250 km, pointy-topped
**366**	Logistic Type-5	289.76	164.65	172.13	157.29	156	94.75	250 km, flat-topped
**375**	Gompertz Type-4	493.27	251.82	269.11	234.53	205	81.41	250 km, flat-topped
**375**	Gompertz Type-4	447.41	251.00	265.68	236.32	206	82.07	250 km, pointy-topped
**375**	Gompertz Type-4	280.33	111.92	120.25	103.60	98	87.56	750 km, pointy-topped
**375**	Gompertz Type-4	350.86	136.87	145.12	128.63	122	89.13	500 km, pointy-topped

**375**	Logistic Type-5	281.46	107.76	117.43	98.60	98	90.95	750 km, pointy-topped
**375**	Gompertz Type-4	335.83	136.17	141.92	130.42	124	91.06	500 km, flat-topped
**375**	Gompertz Type-4	282.87	103.01	108.09	97.93	95	92.23	750 km, flat-topped
**375**	Logistic Type-4	281.38	103.34	108.80	97.88	98	94.83	750 km, pointy-topped
**385**	Gompertz Type-4	372.42	165.23	171.50	158.96	154	93.20	500 km, flat-topped
**385**	Logistic Type-5	372.85	159.34	168.66	150.44	154	96.65	500 km, flat-topped
**385**	Logistic Type-5	381.27	160.20	168.10	152.50	156	97.38	500 km, pointy-topped
**385**	Logistic Type-4	380.28	157.22	161.44	153.01	156	99.22	500 km, pointy-topped
**385**	Logistic Type-5	515.10	242.67	249.52	235.86	243	100.00	250 km, flat-topped
**385**	Logistic Type-4	373.75	153.73	157.94	149.53	154	100.00	500 km, flat-topped
**385**	Logistic Type-5	291.00	112.38	117.55	107.45	113	100.00	750 km, pointy-topped
**385**	Logistic Type-5	514.37	233.01	239.76	226.34	236	100.00	250 km, pointy-topped
**385**	Logistic Type-5	280.33	110.24	114.65	105.95	112	100.00	750 km, flat-topped
**385**	Logistic Type-4	289.64	111.00	113.82	108.18	113	100.00	750 km, pointy-topped
**385**	Logistic Type-4	278.91	109.27	111.77	106.76	112	100.00	750 km, flat-topped
**400**	Logistic Type-4	297.20	125.38	128.24	122.52	126	100.00	500 km, flat-topped
**400**	Logistic Type-4	237.44	94.30	96.78	91.81	95	100.00	750 km, pointy-topped
**400**	Logistic Type-4	372.13	195.34	198.84	191.84	197	100.00	250 km, pointy-topped
**400**	Logistic Type-5	414.86	199.15	204.38	193.93	201	100.00	250 km, flat-topped
**400**	Logistic Type-5	281.57	126.80	129.89	123.73	128	100.00	500 km, pointy-topped
**400**	Logistic Type-5	374.12	195.00	199.98	190.04	197	100.00	250 km, pointy-topped
**400**	Logistic Type-4	256.99	102.57	105.01	100.14	104	100.00	750 km, flat-topped
**400**	Logistic Type-5	298.72	124.27	128.39	120.27	126	100.00	500 km, flat-topped
**400**	Logistic Type-4	413.68	197.71	201.19	194.22	201	100.00	250 km, flat-topped
**400**	Logistic Type-5	237.84	92.93	96.24	89.76	95	100.00	750 km, pointy-topped
**400**	Logistic Type-5	258.42	101.62	105.00	98.35	104	100.00	750 km, flat-topped
**415**	Gompertz Type-4	392.50	196.54	201.18	191.89	186	94.64	250 km, flat-topped
**415**	Logistic Type-5	391.73	192.65	198.82	186.49	186	96.55	250 km, flat-topped
**415**	Logistic Type-4	282.11	128.25	131.34	125.16	125	97.47	500 km, pointy-topped
**415**	Logistic Type-5	283.05	127.46	131.42	123.52	125	98.07	500 km, pointy-topped
**415**	Logistic Type-5	221.52	91.53	95.77	87.39	90	98.33	750 km, flat-topped
**415**	Logistic Type-5	217.32	98.64	102.44	94.88	97	98.34	750 km, pointy-topped
**415**	Logistic Type-5	399.53	185.05	190.41	179.70	182	98.35	250 km, pointy-topped
**415**	Logistic Type-4	219.57	91.29	94.25	88.33	90	98.58	750 km, flat-topped
**415**	Logistic Type-4	277.92	121.67	124.65	118.68	121	99.45	500 km, flat-topped
**415**	Logistic Type-4	218.33	96.81	99.32	94.29	97	100.00	750 km, pointy-topped
**425**	Logistic Type-4	438.38	198.34	204.76	191.92	194	97.81	500 km, pointy-topped
**425**	Logistic Type-4	572.68	324.69	331.55	317.83	318	97.94	250 km, flat-topped
**425**	Logistic Type-4	566.63	327.69	334.02	321.35	321	97.96	250 km, pointy-topped
**425**	Logistic Type-5	568.22	326.02	333.90	318.14	321	98.46	250 km, pointy-topped
**425**	Logistic Type-5	439.68	196.18	203.74	188.66	194	98.89	500 km, pointy-topped
**425**	Logistic Type-4	406.32	188.69	193.29	184.09	187	99.10	500 km, flat-topped
**425**	Logistic Type-5	320.23	125.55	130.69	120.53	126	100.00	750 km, pointy-topped
**425**	Logistic Type-5	405.11	185.84	190.85	180.84	187	100.00	500 km, flat-topped

**425**	Logistic Type-5	326.88	128.21	132.87	123.65	130	100.00	750 km, flat-topped
**436**	Logistic Type-4	571.14	301.28	306.37	296.19	293	97.25	250 km, pointy-topped
**436**	Logistic Type-4	445.03	177.16	181.44	172.89	173	97.65	500 km, pointy-topped
**436**	Logistic Type-5	571.01	297.61	304.15	291.08	293	98.45	250 km, pointy-topped
**436**	Logistic Type-5	445.36	174.84	180.03	169.66	173	98.95	500 km, pointy-topped
**436**	Logistic Type-5	569.84	289.88	296.02	283.73	287	99.01	250 km, flat-topped
**436**	Logistic Type-5	328.37	125.22	129.01	121.43	125	99.83	750 km, pointy-topped
**436**	Logistic Type-4	474.17	175.30	179.35	171.24	176	100.00	500 km, flat-topped
**436**	Logistic Type-4	373.54	133.33	136.66	130.00	134	100.00	750 km, flat-topped
**436**	Logistic Type-4	326.71	124.33	127.26	121.39	125	100.00	750 km, pointy-topped
**436**	Logistic Type-5	476.11	174.86	180.13	169.61	176	100.00	500 km, flat-topped
**436**	Logistic Type-5	371.68	131.00	134.64	127.39	134	100.00	750 km, flat-topped
**449**	Logistic Type-5	860.49	368.25	376.62	359.93	379	100.00	250 km, flat-topped
**449**	Logistic Type-5	574.14	194.69	200.77	188.74	201	100.00	500 km, flat-topped
**449**	Logistic Type-5	847.70	366.24	374.79	357.74	379	100.00	250 km, pointy-topped
**449**	Logistic Type-5	447.17	133.01	137.93	128.23	138	100.00	750 km, flat-topped
**449**	Logistic Type-5	459.88	135.68	140.44	131.08	141	100.00	750 km, pointy-topped
**449**	Logistic Type-5	582.32	195.31	200.80	189.96	203	100.00	500 km, pointy-topped
**456**	Logistic Type-5	289.11	83.18	85.62	80.84	87	100.00	750 km, flat-topped
**456**	Logistic Type-5	545.21	226.84	230.90	222.80	238	100.00	250 km, flat-topped
**456**	Logistic Type-5	364.74	118.81	122.07	115.79	125	100.00	500 km, flat-topped
**456**	Logistic Type-5	374.65	124.85	128.06	121.72	132	100.00	500 km, pointy-topped
**456**	Logistic Type-5	307.68	84.94	87.79	82.30	90	100.00	750 km, pointy-topped
**456**	Logistic Type-5	516.22	210.38	214.31	206.49	223	100.00	250 km, pointy-topped
**465**	Logistic Type-5	363.49	133.78	136.91	130.71	140	100.00	500 km, flat-topped
**465**	Logistic Type-5	538.24	230.90	236.43	225.44	242	100.00	250 km, flat-topped
**465**	Logistic Type-5	563.16	239.02	244.25	233.84	251	100.00	250 km, pointy-topped
**465**	Logistic Type-5	405.27	135.02	137.79	132.31	142	100.00	500 km, pointy-topped
**465**	Logistic Type-5	267.84	90.18	92.49	87.97	95	100.00	750 km, pointy-topped
**465**	Logistic Type-5	298.73	100.20	102.63	97.81	106	100.00	750 km, flat-topped
**475**	Logistic Type-3	206.29	170.04	195.69	144.38	79	46.46	750 km, flat-topped
**475**	Logistic Type-4	207.30	161.78	188.70	134.86	79	48.83	750 km, flat-topped
**475**	Logistic Type-5	207.53	138.06	172.61	104.26	79	57.22	750 km, flat-topped
**475**	Gompertz Type-4	426.07	272.59	288.07	257.12	173	63.46	250 km, pointy-topped
**475**	Logistic Type-5	213.34	110.13	131.03	91.67	81	73.55	750 km, pointy-topped
**475**	Logistic Type-3	415.74	237.02	250.53	223.50	175	73.83	250 km, flat-topped
**475**	Logistic Type-4	415.78	230.89	245.53	216.24	175	75.79	250 km, flat-topped
**475**	Logistic Type-3	425.26	228.15	238.96	217.35	173	75.83	250 km, pointy-topped
**475**	Logistic Type-4	426.71	225.56	238.12	213.00	173	76.70	250 km, pointy-topped
**475**	Logistic Type-5	416.64	207.94	236.78	183.48	175	84.16	250 km, flat-topped
**475**	Logistic Type-5	250.93	116.55	125.79	108.70	108	92.66	500 km, pointy-topped
**475**	Logistic Type-5	213.31	104.53	107.67	101.49	102	97.58	500 km, flat-topped
**482**	Logistic Type-3	399.34	185.49	191.25	179.74	173	93.26	250 km, pointy-topped
**482**	Logistic Type-4	400.78	184.60	190.75	178.46	173	93.71	250 km, pointy-topped

**482**	Logistic Type-4	222.03	89.39	92.33	86.45	84	93.97	750 km, flat-topped
**482**	Logistic Type-3	285.13	113.79	117.16	110.42	108	94.91	500 km, pointy-topped
**482**	Logistic Type-3	402.94	185.38	189.77	180.98	176	94.94	250 km, flat-topped
**482**	Logistic Type-4	316.63	118.66	122.95	114.37	113	95.23	500 km, flat-topped
**482**	Logistic Type-4	403.73	184.33	189.00	179.65	176	95.48	250 km, flat-topped
**482**	Logistic Type-4	285.15	112.86	116.37	109.35	108	95.69	500 km, pointy-topped
**482**	Logistic Type-5	222.18	86.76	91.20	82.50	84	96.82	750 km, flat-topped
**482**	Logistic Type-5	314.76	113.48	119.28	108.14	113	99.58	500 km, flat-topped
**482**	Logistic Type-5	204.13	77.93	80.94	74.96	78	100.00	750 km, pointy-topped
**492**	Logistic Type-5	147.86	75.12	79.44	70.97	71	94.52	750 km, pointy-topped
**492**	Logistic Type-5	324.13	153.28	157.21	149.36	150	97.86	250 km, pointy-topped
**492**	Logistic Type-5	241.60	98.49	102.78	94.32	97	98.48	500 km, pointy-topped
**492**	Logistic Type-5	311.18	142.49	146.71	138.30	141	98.96	250 km, flat-topped
**492**	Logistic Type-5	159.16	78.53	80.67	76.40	78	99.33	750 km, flat-topped
**492**	Logistic Type-5	218.53	96.72	99.14	94.31	97	100.00	500 km, flat-topped
**505**	Logistic Type-5	399.28	231.73	238.75	224.72	225	97.10	250 km, pointy-topped
**505**	Logistic Type-5	410.36	221.18	226.89	215.49	221	99.92	250 km, flat-topped
**505**	Logistic Type-5	243.19	98.91	102.50	95.38	99	100.00	750 km, flat-topped
**505**	Logistic Type-5	225.98	95.88	98.32	93.44	97	100.00	750 km, pointy-topped
**505**	Logistic Type-5	327.25	133.50	136.77	130.24	136	100.00	500 km, pointy-topped
**505**	Logistic Type-5	349.31	140.45	143.90	137.01	144	100.00	500 km, flat-topped
**515**	Logistic Type-4	225.80	100.30	102.71	97.89	100	99.70	750 km, pointy-topped
**515**	Logistic Type-5	380.37	220.85	225.47	216.24	224	100.00	250 km, flat-topped
**515**	Logistic Type-5	211.61	89.20	91.60	86.82	91	100.00	750 km, flat-topped
**515**	Logistic Type-5	252.61	124.48	127.17	121.80	127	100.00	500 km, pointy-topped
**515**	Logistic Type-4	373.91	216.38	219.44	213.32	221	100.00	250 km, pointy-topped
**515**	Logistic Type-5	261.29	127.82	130.10	125.55	131	100.00	500 km, flat-topped
**530**	Gompertz Type-4	98.95	42.58	61.54	34.14	38	89.24	500 km, pointy-topped
**530**	Logistic Type-4	99.15	39.73	45.23	34.90	38	95.64	500 km, pointy-topped
**530**	Gompertz Type-3	97.92	39.44	43.46	35.45	38	96.36	500 km, pointy-topped
**530**	Gompertz Type-3	104.26	42.13	45.79	38.50	41	97.31	500 km, flat-topped
**530**	Gompertz Type-3	133.07	56.28	61.39	51.21	55	97.73	250 km, flat-topped
**530**	Gompertz Type-4	105.68	40.98	45.34	36.99	41	100.00	500 km, flat-topped
**530**	Logistic Type-4	105.96	39.87	43.90	36.36	41	100.00	500 km, flat-topped
**530**	Logistic Type-3	81.01	31.07	32.72	29.41	32	100.00	750 km, flat-topped
**530**	Gompertz Type-4	132.39	53.27	58.15	48.64	55	100.00	250 km, flat-topped
**530**	Logistic Type-3	80.67	30.80	32.52	29.08	32	100.00	750 km, pointy-topped
**530**	Logistic Type-4	124.06	48.11	51.26	45.08	50	100.00	250 km, pointy-topped
**530**	Logistic Type-3	122.20	47.81	50.13	45.49	50	100.00	250 km, pointy-topped
**530**	Logistic Type-4	82.37	30.54	32.58	28.58	32	100.00	750 km, pointy-topped
**530**	Logistic Type-3	104.91	38.88	41.34	36.41	41	100.00	500 km, flat-topped
**530**	Logistic Type-4	133.92	51.77	56.11	47.73	55	100.00	250 km, flat-topped
**530**	Logistic Type-3	132.06	51.32	54.48	48.16	55	100.00	250 km, flat-topped

Table S13.

Shallow-water areas through the Phanerozoic for the 49-bin approach. All values in km².

Map age (Myr)	Shallow-water area (on paleo-map)	Shallow-water area (re-projected)	Shallow-water area with fossil data (re-projected)
**3**	37381009	45562380	30845542
**15**	29791849	49527261	18332505
**26**	30231773	56625716	12120581
**36**	30371347	57193541	10440182
**52**	62861103	86883764	14087765
**61**	49574328	70549172	11536458
**69**	51015193	65097671	13301881
**75**	67074021	78174454	20154491
**87**	66165265	72230448	21680712
**97**	58236532	65082490	17596846
**107**	53746727	59948771	17254067
**116**	56535746	61621174	12645794
**127**	55733619	60532961	9633416
**136**	40714078	47147172	10469307
**145**	36296354	51264884	8047182
**155**	41747547	45100971	19142304
**168**	54124580	56562591	12830881
**178**	43840101	47019673	10058649
**191**	30789717	30940971	12112388
**201**	15959354	27500752	3395313
**213**	21132037	17865770	15362567
**232**	40069786	26271552	8729607
**245**	37043499	23617311	9843165
**252**	62981208	46050770	10247742
**256**	62976599	45309527	10921527
**265**	62980243	34909583	20566516
**280**	68589864	36352049	21726505
**293**	58645837	37737776	18196567
**301**	62350481	56931051	8821031
**315**	65600934	44353036	12448784
**327**	62094073	49450627	6277598
**339**	72358314	53202222	15112889
**354**	77702243	63388647	10509078
**366**	82721260	67466489	12115551
**375**	89351292	71999935	16980182

**385**	90092774	72351702	19606210
**400**	81808027	66765332	17235215
**415**	90832434	72862177	14851544
**425**	95156457	63939653	22594105
**436**	78658276	50988761	25723925
**449**	76587123	49218828	23654040
**456**	79013434	56624275	17304018
**465**	81165030	54708318	21425304
**475**	85715370	64350440	13588608
**482**	88424911	63055755	15669607
**492**	77585145	58536631	13729727
**505**	73273303	51588350	18219000
**515**	77775772	55109290	17288490
**530**	76090504	62874653	7349695

Table S14.

Areas of deposits (in km²) retrieved from Macrostrat Database through the Phanerozoic, applying the strict filter (see Methods) for the 49-bin approach.

Map age (Myr)	Area shallow-water deposits	% Area of deposits of reprojected area	Area of deposits (today in the sea)	Area of deposits (today on land)	% Area of deposits on land	Area of deposits total
**3**	221786	0.487	5136	216649	0.476	473299
**15**	200702	0.405	4555	196147	0.396	483652
**26**	1115	0.002	0	1115	0.002	40192
**36**	4726	0.008	899	3827	0.007	282128
**52**	77734	0.089	922	76812	0.088	279667
**61**	5117	0.007	103	5014	0.007	87993
**69**	16581	0.025	5	16576	0.025	971523
**75**	60463	0.077	36	60427	0.077	986085
**87**	187090	0.259	0	187090	0.259	945300
**97**	24073	0.037	0	24073	0.037	178874
**107**	202419	0.338	135392	67028	0.112	462820
**116**	260962	0.423	136244	124718	0.202	342181
**127**	256694	0.424	119978	136716	0.226	340963
**136**	0	0.000	0	0	0.000	0
**145**	43876	0.086	1622	42254	0.082	102315
**155**	71982	0.160	1962	70020	0.155	234434
**168**	50488	0.089	359	50129	0.089	132119
**178**	22324	0.047	210	22114	0.047	132119
**191**	0	0.000	0	0	0.000	0
**201**	0	0.000	0	0	0.000	0
**213**	0	0.000	0	0	0.000	0
**232**	0	0.000	0	0	0.000	0
**245**	30348	0.128	4180	26168	0.111	140643
**252**	36609	0.079	2233	34376	0.075	210082
**256**	357915	0.790	29846	328069	0.724	438078
**265**	29000	0.083	21844	7156	0.020	477323
**280**	0	0.000	0	0	0.000	23742
**293**	6587	0.017	0	6587	0.017	46199
**301**	18014	0.032	75	17939	0.032	57520
**315**	8423	0.019	11	8412	0.019	54797
**327**	0	0.000	0	0	0.000	4084
**339**	6348	0.012	0	6348	0.012	6348
**354**	0	0.000	0	0	0.000	0
**366**	127479	0.189	277	127202	0.189	143676

**375**	39405	0.055	268	39137	0.054	143676
**385**	78233	0.108	10892	67342	0.093	259170
**400**	20212	0.030	1555	18658	0.028	43738
**415**	203341	0.279	82613	120728	0.166	333316
**425**	180224	0.282	128326	51898	0.081	289579
**436**	147909	0.290	94733	53176	0.104	289579
**449**	28094	0.057	4566	23527	0.048	291022
**456**	1444	0.003	3	1441	0.003	1444
**465**	23348	0.043	314	23034	0.042	24820
**475**	117544	0.183	1130	116414	0.181	129633
**482**	249335	0.395	742	248593	0.394	321298
**492**	202428	0.346	0	202428	0.346	215042
**505**	53538	0.104	0	53538	0.104	167855
**515**	46119	0.084	0	46119	0.084	167855
**530**	0	0.000	0	0	0.000	0

Table S15.

Areas of deposits (in km²) retrieved from Macrostrat Database through the Phanerozoic, applying the moderate filter (see Methods) for the 49-bin approach.

Map age (Myr)	Area shallow-water deposits	% Area of deposits of reprojected area	Area of deposits (today in the sea)	Area of deposits (today on land)	% Area of deposits on land	Area of deposits total
**3**	2839297	6.232	850880	1988417	4.364	34473953
**15**	2084149	4.208	565355	1518793	3.067	17533700
**26**	959697	1.695	287964	671734	1.186	3230413
**36**	737685	1.290	64748	672937	1.177	3003765
**52**	1339017	1.541	87117	1251900	1.441	3001304
**61**	1313137	1.861	266559	1046578	1.483	2731414
**69**	651828	1.001	187114	464714	0.714	4729416
**75**	1364767	1.746	181575	1183192	1.514	4717130
**87**	1354089	1.875	171661	1182428	1.637	4593264
**97**	1134463	1.743	210175	924288	1.420	3842396
**107**	1434329	2.393	811278	623051	1.039	3587974
**116**	1520320	2.467	863856	656463	1.065	3406586
**127**	1511400	2.497	837356	674043	1.114	3405368
**136**	1164404	2.470	742994	421409	0.894	3234844
**145**	1225267	2.390	752962	472305	0.921	3703048
**155**	173868	0.386	16447	157422	0.349	799252
**168**	220473	0.390	19656	200816	0.355	497185
**178**	206752	0.440	10933	195819	0.416	698976
**191**	49916	0.161	6773	43143	0.139	260416
**201**	124434	0.452	23098	101336	0.368	600009
**213**	42543	0.238	13043	29500	0.165	339593
**232**	84210	0.321	24539	59671	0.227	339593
**245**	33444	0.142	5726	27719	0.117	219579
**252**	237085	0.515	3946	233139	0.506	574669
**256**	792706	1.750	31226	761480	1.681	1187262
**265**	51276	0.147	23185	28090	0.080	1491679
**280**	3641	0.010	41	3601	0.010	384862
**293**	16766	0.044	711	16055	0.043	320839
**301**	81947	0.144	5478	76469	0.134	570400
**315**	140102	0.316	1831	138271	0.312	1019860
**327**	38259	0.077	1332	36927	0.075	367514
**339**	75312	0.142	1379	73934	0.139	367788
**354**	118262	0.187	1096	117166	0.185	429984
**366**	713150	1.057	67203	645946	0.957	1194420

**375**	288447	0.401	77313	211134	0.293	765303
**385**	584773	0.808	70736	514037	0.710	1278950
**400**	66077	0.099	3774	62303	0.093	205228
**415**	368385	0.506	104505	263880	0.362	818648
**425**	372688	0.583	129057	243630	0.381	833346
**436**	189828	0.372	95014	94814	0.186	789789
**449**	186639	0.379	7099	179540	0.365	1508598
**456**	255981	0.452	150592	105390	0.186	718945
**465**	425561	0.778	195892	229669	0.420	893953
**475**	425595	0.661	184699	240896	0.374	815922
**482**	378950	0.601	48934	330016	0.523	761166
**492**	202436	0.346	0	202436	0.346	216339
**505**	55317	0.107	0	55317	0.107	196933
**515**	61958	0.112	0	61958	0.112	299163
**530**	43211	0.069	1012	42199	0.067	129117

Table S16.
Results of the test for the impact of the chosen patch size threshold to determine inland depressions.

Map age (Myr)	Threshold (km²)	Total shallow water area (km²)	Area of all patches (km²)	Area of isolated non-marine patches (km²)	Number of patches	Number of isolated non-marine patches
**0**	1000	43443285.1	1215471.1	11391.5	127	5
**0**	2500	43321160.7	1084830.5	3994.7	43	1
**0**	5000	43246117.0	1009984.3	0.0	21	0
**0**	10000	43149102.3	926687.8	0.0	10	0
**0**	25000	43014423.1	819510.7	0.0	4	0
**3**	1000	37590380.4	1787440.7	1207.2	124	1
**3**	2500	37475740.7	1675939.6	0.0	56	0
**3**	5000	37381008.7	1587068.0	0.0	30	0
**3**	10000	37293059.0	1498437.9	0.0	17	0
**3**	25000	37144398.2	1376555.0	0.0	9	0
**11**	1000	33339622.8	1310949.3	2151.0	163	1
**11**	2500	33197524.8	1180220.9	0.0	79	0
**11**	5000	33073271.4	1071353.8	0.0	47	0
**11**	10000	32858061.2	867337.6	0.0	18	0
**11**	25000	32701784.2	729344.1	0.0	10	0
**15**	1000	29931334.6	741833.1	0.0	88	0
**15**	2500	29862963.4	676359.2	0.0	46	0
**15**	5000	29791848.5	604146.9	0.0	25	0
**15**	10000	29670865.5	501140.1	0.0	11	0
**15**	25000	29574531.2	421135.9	0.0	6	0
**20**	1000	35237489.0	569840.5	0.0	132	0
**20**	2500	35120061.8	455815.4	0.0	60	0
**20**	5000	34971181.5	337971.1	0.0	25	0
**20**	10000	34837336.4	250175.0	0.0	13	0
**20**	25000	34607617.9	97014.7	0.0	2	0
**26**	1000	30411030.5	1655132.0	172387.6	130	6
**26**	2500	30321083.4	1555198.1	166176.1	60	2
**26**	5000	30231772.8	1462331.7	163651.9	35	1
**26**	10000	30080873.3	1320606.9	163651.9	15	1
**26**	25000	29978283.5	1232327.9	163651.9	8	1
**31**	1000	33462230.1	2851178.1	157703.7	180	10
**31**	2500	33334684.3	2729909.8	156474.6	100	9
**31**	5000	33181699.6	2604229.7	147826.7	63	6
**31**	10000	32951564.1	2418540.9	132506.3	35	4
**31**	25000	32743070.3	2210244.7	92142.0	22	1
**36**	1000	30642746.3	1526664.3	120961.1	189	10
**36**	2500	30494700.1	1372560.2	118590.6	89	8
**36**	5000	30371346.6	1243390.2	104762.7	51	4

**36**	10000	30191891.4	1062376.6	89222.8	24	2
**36**	25000	30004424.8	912552.6	73068.3	14	1
**40**	1000	33417827.2	1782568.0	59350.2	270	7
**40**	2500	33227573.1	1590843.3	56343.1	143	5
**40**	5000	32999610.5	1371023.8	53577.6	83	4
**40**	10000	32642082.9	1037969.3	37642.6	35	2
**40**	25000	32289844.9	746025.4	25790.1	17	1
**45**	1000	41877332.0	1293894.9	0.0	91	0
**45**	2500	41807578.0	1225962.7	0.0	46	0
**45**	5000	41731810.0	1147407.2	0.0	25	0
**45**	10000	41668794.3	1089571.5	0.0	16	0
**45**	25000	41502201.3	958908.9	0.0	8	0
**52**	1000	63117797.1	2145604.8	965.8	163	1
**52**	2500	62986893.1	2021131.9	0.0	83	0
**52**	5000	62861103.3	1905153.3	0.0	48	0
**52**	10000	62671112.5	1714482.1	0.0	21	0
**52**	25000	62509172.6	1552542.2	0.0	11	0
**56**	1000	49770771.5	1420848.1	1207.2	147	1
**56**	2500	49644784.1	1292665.8	0.0	61	0
**56**	5000	49534951.2	1190668.7	0.0	31	0
**56**	10000	49424767.1	1092710.2	0.0	16	0
**56**	25000	49259930.0	941942.3	0.0	7	0
**61**	1000	49801368.5	1614438.5	0.0	142	0
**61**	2500	49675468.9	1496286.9	0.0	63	0
**61**	5000	49574327.8	1394970.2	0.0	32	0
**61**	10000	49449130.5	1290251.4	0.0	15	0
**61**	25000	49308042.2	1176204.3	0.0	6	0
**66**	1000	45864459.9	1426401.2	0.0	149	0
**66**	2500	45735794.7	1304891.4	0.0	70	0
**66**	5000	45601796.0	1175062.9	0.0	31	0
**66**	10000	45507788.3	1080484.6	0.0	18	0
**66**	25000	45378025.7	975282.9	0.0	11	0
**69**	1000	51302242.7	1343368.0	18349.4	170	1
**69**	2500	51144626.7	1193478.2	18349.4	71	1
**69**	5000	51015193.4	1077082.5	18349.4	37	1
**69**	10000	50898073.4	958908.9	18349.4	21	1
**69**	25000	50665677.1	793786.4	0.0	10	0
**75**	1000	67274261.7	1601927.5	0.0	131	0
**75**	2500	67185082.8	1511804.8	0.0	71	0
**75**	5000	67074020.8	1400479.4	0.0	37	0
**75**	10000	66949701.5	1276160.1	0.0	19	0
**75**	25000	66737388.6	1092007.8	0.0	7	0
**87**	1000	66402753.8	1235598.3	0.0	148	0
**87**	2500	66272596.1	1110905.9	0.0	68	0

**87**	5000	66165265.4	1007833.3	0.0	39	0
**87**	10000	66056859.1	899361.2	0.0	24	0
**87**	25000	65719634.3	667886.8	0.0	10	0
**92**	1000	66396805.6	1236125.1	0.0	148	0
**92**	2500	66266691.8	1111476.6	0.0	68	0
**92**	5000	66159185.5	1008228.4	0.0	39	0
**92**	10000	66050779.3	899756.3	0.0	24	0
**92**	25000	65713400.8	667864.9	0.0	10	0
**97**	1000	58396101.2	871507.9	0.0	101	0
**97**	2500	58313507.0	791964.7	0.0	48	0
**97**	5000	58236531.8	714791.9	0.0	27	0
**97**	10000	58133898.2	621859.7	0.0	14	0
**97**	25000	58067260.9	555222.5	0.0	9	0
**107**	1000	53868500.6	906143.5	0.0	86	0
**107**	2500	53801446.3	843896.1	0.0	46	0
**107**	5000	53746727.4	794313.2	0.0	32	0
**107**	10000	53646135.0	693720.8	0.0	17	0
**107**	25000	53515296.9	562882.7	0.0	9	0
**116**	1000	56705894.7	952192.5	0.0	126	0
**116**	2500	56618976.6	860994.3	0.0	66	0
**116**	5000	56535745.9	777083.2	0.0	43	0
**116**	10000	56379776.1	620894.0	0.0	21	0
**116**	25000	56111295.7	375174.7	0.0	6	0
**122**	1000	67610498.8	1517533.5	1777.9	136	1
**122**	2500	67504177.7	1411651.4	0.0	69	0
**122**	5000	67405385.2	1312332.1	0.0	42	0
**122**	10000	67287123.8	1193917.1	0.0	25	0
**122**	25000	67001610.9	975370.7	0.0	11	0
**131**	1000	46823851.7	1945232.2	36457.3	176	4
**131**	2500	46710309.4	1837747.8	36457.3	106	4
**131**	5000	46567553.0	1702102.9	36457.3	69	4
**131**	10000	46303747.7	1420628.6	14442.5	28	1
**131**	25000	46002672.9	1159654.7	0.0	10	0
**136**	1000	40874327.5	1479166.6	20785.7	114	6
**136**	2500	40782295.2	1385707.7	18525.0	56	4
**136**	5000	40714077.7	1318038.9	12115.9	36	2
**136**	10000	40581637.3	1181537.9	0.0	17	0
**136**	25000	40393995.1	1013584.0	0.0	7	0
**142**	1000	42895503.3	3087503.3	401074.5	158	7
**142**	2500	42784880.2	2974685.3	399296.6	81	6
**142**	5000	42675157.1	2863842.7	396355.5	48	5
**142**	10000	42496887.1	2704273.3	386610.1	25	4
**142**	25000	42360100.8	2589238.5	345082.5	17	2
**145**	1000	36546024.0	3057740.4	357703.2	171	8

**145**	2500	36428333.3	2936252.6	353401.2	93	5
**145**	5000	36296353.8	2803526.8	350108.9	54	4
**145**	10000	36164396.3	2677693.1	350108.9	36	4
**145**	25000	35944489.0	2460024.6	328335.4	22	3
**149**	1000	41369519.4	2216149.0	341417.1	174	9
**149**	2500	41205735.8	2042971.2	334064.1	68	5
**149**	5000	41119059.1	1971066.2	334064.1	46	5
**149**	10000	40943927.8	1812791.8	323967.6	24	4
**149**	25000	40714385.0	1569882.0	277479.6	10	2
**160**	1000	65297115.6	1620342.8	181737.9	188	5
**160**	2500	65127076.5	1459346.7	179850.3	80	4
**160**	5000	64980435.1	1324513.8	179850.3	41	4
**160**	10000	64824509.3	1210093.6	179850.3	25	4
**160**	25000	64592837.3	984216.2	151184.9	11	2
**165**	1000	54366831.2	1748700.6	30136.0	167	3
**165**	2500	54240119.5	1624161.9	30136.0	86	3
**165**	5000	54134105.7	1519223.6	27172.9	55	2
**165**	10000	53927214.2	1353113.4	21597.8	31	1
**165**	25000	53623286.1	1058930.6	0.0	13	0
**168**	1000	54358293.0	1774139.6	30136.0	169	3
**168**	2500	54230549.7	1643828.2	30136.0	87	3
**168**	5000	54124579.8	1538911.9	27172.9	56	2
**168**	10000	53917424.9	1372713.9	21597.8	32	1
**168**	25000	53595345.0	1060203.7	0.0	13	0
**172**	1000	51250245.4	1948985.5	18810.3	200	6
**172**	2500	51113503.0	1805043.8	12818.2	107	2
**172**	5000	50999192.5	1688604.2	8187.0	71	1
**172**	10000	50751563.6	1429693.5	0.0	34	0
**172**	25000	50487012.1	1182810.9	0.0	18	0
**178**	1000	44130311.5	2926616.9	0.0	183	0
**178**	2500	43987423.3	2790862.2	0.0	98	0
**178**	5000	43840101.5	2645011.0	0.0	55	0
**178**	10000	43662270.5	2467026.3	0.0	30	0
**178**	25000	43306213.4	2189919.9	0.0	11	0
**187**	1000	25458760.1	1522406.2	183867.0	132	4
**187**	2500	25187536.0	1428881.4	356166.8	73	5
**187**	5000	25074959.5	1306910.7	347343.3	40	3
**187**	10000	24947984.4	1189790.7	347343.3	23	3
**187**	25000	24813941.7	1055638.3	347343.3	15	3
**196**	1000	25234133.8	2459344.2	617140.7	148	20
**196**	2500	25126320.2	2338141.7	605024.8	72	12
**196**	5000	25046447.7	2256118.2	592974.8	48	9
**196**	10000	24954239.9	2152277.3	581605.2	33	7
**196**	25000	24681128.2	1873678.4	552632.5	17	5

**201**	1000	16152725.3	1839306.2	407286.1	173	34
**201**	2500	16038305.1	1697735.0	379849.8	84	17
**201**	5000	15959354.4	1580351.6	341724.3	51	7
**201**	10000	15813547.1	1428552.2	327325.8	29	5
**201**	25000	15556984.9	1234720.4	312993.1	17	4
**205**	1000	17849845.8	1673964.2	568765.0	155	37
**205**	2500	17764398.2	1558622.1	541372.7	81	19
**205**	5000	17665232.6	1438407.3	517997.0	47	12
**205**	10000	17567998.4	1321814.1	486258.7	31	8
**205**	25000	17386611.6	1115493.3	441746.0	18	5
**218**	1000	24658345.1	2325894.1	596903.7	202	33
**218**	2500	24522107.5	2178704.0	583185.6	108	24
**218**	5000	24417915.5	2038340.0	547628.1	69	15
**218**	10000	24204000.3	1816654.8	488278.0	39	7
**218**	25000	23871933.5	1460158.8	445587.1	16	4
**232**	1000	40325426.3	2086320.5	423133.3	164	14
**232**	2500	40189364.3	1937023.3	415407.2	72	9
**232**	5000	40069786.0	1802497.7	402545.1	33	5
**232**	10000	39985677.4	1717335.5	394928.8	20	4
**232**	25000	39843491.6	1562507.1	382791.0	10	3
**240**	1000	7899014.6	2554032.3	781955.9	85	28
**240**	2500	7877548.4	2521789.2	771178.9	65	21
**240**	5000	7845876.0	2474730.4	755792.7	52	17
**240**	10000	7751275.7	2345362.9	721025.4	33	12
**240**	25000	7541816.2	2036013.4	621135.4	15	6
**245**	1000	37218893.2	1232327.9	295543.6	132	12
**245**	2500	37115842.6	1112617.9	284700.8	55	6
**245**	5000	37043498.6	1035862.2	279016.0	32	4
**245**	10000	36958073.0	956494.5	279016.0	21	4
**245**	25000	36831536.9	814550.2	263607.8	11	3
**252**	1000	63019575.2	582944.1	40934.9	44	7
**252**	2500	62990339.1	550832.7	38103.5	24	5
**252**	5000	62981208.3	534744.1	31145.7	20	3
**252**	10000	62948262.8	493326.3	22673.3	14	2
**252**	25000	62854233.2	376579.4	0.0	6	0
**256**	1000	63017182.8	582263.7	40759.3	44	7
**256**	2500	62985729.8	550349.8	38059.6	24	5
**256**	5000	62976599.0	534261.2	31101.8	20	3
**256**	10000	62943675.5	492865.3	22629.4	14	2
**256**	25000	62849580.0	376096.5	0.0	6	0
**263**	1000	70826360.0	892118.1	191966.2	58	8
**263**	2500	70803620.8	867447.4	190166.3	41	7
**263**	5000	70775438.2	835709.1	186654.5	31	6
**263**	10000	70706913.4	748944.6	168436.8	19	3

**263**	25000	70544139.4	574164.5	156452.6	7	2
**265**	1000	63020892.2	588914.2	40913.0	44	7
**265**	2500	62989373.4	556802.8	38081.6	24	5
**265**	5000	62980242.6	540714.2	31123.7	20	3
**265**	10000	62947319.0	499318.3	22651.4	14	2
**265**	25000	62853223.6	382527.6	0.0	6	0
**269**	1000	60879413.7	976994.9	0.0	29	0
**269**	2500	60861327.7	958733.3	0.0	18	0
**269**	5000	60834264.6	931626.3	0.0	10	0
**269**	10000	60814378.8	911740.5	0.0	7	0
**269**	25000	60761130.4	858492.2	0.0	4	0
**280**	1000	68613086.3	42866.4	0.0	17	0
**280**	2500	68595241.7	24626.8	0.0	3	0
**280**	5000	68589864.2	19117.6	0.0	1	0
**280**	10000	68589864.2	19117.6	0.0	1	0
**280**	25000	68570746.6	0.0	0.0	0	0
**287**	1000	64626463.2	241219.8	1426.7	32	1
**287**	2500	64591783.8	204630.7	0.0	9	0
**287**	5000	64568254.4	180530.7	0.0	2	0
**287**	10000	64568254.4	180530.7	0.0	2	0
**287**	25000	64568254.4	180530.7	0.0	2	0
**293**	1000	58675775.7	4495094.2	1009.7	20	1
**293**	2500	58660652.8	4479334.8	0.0	9	0
**293**	5000	58645837.2	4464233.8	0.0	5	0
**293**	10000	58622527.3	4449967.0	0.0	3	0
**293**	25000	58607382.5	4434822.1	0.0	2	0
**297**	1000	58265526.5	326952.7	0.0	24	0
**297**	2500	58240504.6	302479.5	0.0	8	0
**297**	5000	58234710.0	296224.0	0.0	6	0
**297**	10000	58212300.1	273462.9	0.0	3	0
**297**	25000	58197769.8	258932.6	0.0	2	0
**301**	1000	62374339.8	165737.1	0.0	15	0
**301**	2500	62361850.8	151623.9	0.0	6	0
**301**	5000	62350481.2	140012.8	0.0	3	0
**301**	10000	62342623.4	132155.1	0.0	2	0
**301**	25000	62342623.4	132155.1	0.0	2	0
**305**	1000	61630641.0	135184.0	2260.7	22	1
**305**	2500	61612620.9	116329.8	2260.7	9	1
**305**	5000	61601997.5	105311.4	2260.7	5	1
**305**	10000	61583801.8	87071.8	2260.7	3	1
**305**	25000	61583801.8	84811.0	0.0	2	0
**311**	1000	62632701.7	5079135.7	14508.3	14	2
**311**	2500	62618917.7	5064737.2	14508.3	5	2
**311**	5000	62618917.7	5061686.2	11457.4	4	1

**311**	10000	62610862.4	5053521.2	11457.4	3	1
**311**	25000	62610862.4	5042063.8	0.0	2	0
**319**	1000	67145069.8	4606924.4	23990.3	7	2
**319**	2500	67143818.7	4604553.9	22870.9	5	1
**319**	5000	67143818.7	4604553.9	22870.9	5	1
**319**	10000	67137716.8	4598452.1	22870.9	4	1
**319**	25000	67137716.8	4575581.2	0.0	3	0
**327**	1000	62126337.8	4584887.6	0.0	19	0
**327**	2500	62107132.4	4566362.6	0.0	7	0
**327**	5000	62094072.7	4553039.6	0.0	3	0
**327**	10000	62094072.7	4553039.6	0.0	3	0
**327**	25000	62094072.7	4553039.6	0.0	3	0
**339**	1000	72361299.1	246487.5	0.0	4	0
**339**	2500	72358314.0	243370.8	0.0	2	0
**339**	5000	72358314.0	243370.8	0.0	2	0
**339**	10000	72350588.0	235644.7	0.0	1	0
**339**	25000	72350588.0	235644.7	0.0	1	0
**354**	1000	77715236.6	2273699.3	0.0	12	0
**354**	2500	77702242.8	2260595.8	0.0	3	0
**354**	5000	77702242.8	2260595.8	0.0	3	0
**354**	10000	77702242.8	2260595.8	0.0	3	0
**354**	25000	77695131.3	2253484.3	0.0	2	0
**366**	1000	82730193.4	19293.2	0.0	6	0
**366**	2500	82725320.7	14069.3	0.0	2	0
**366**	5000	82721260.1	9833.2	0.0	1	0
**366**	10000	82711426.9	0.0	0.0	0	0
**366**	25000	82711426.9	0.0	0.0	0	0
**375**	1000	89383886.2	173463.1	13432.8	23	1
**375**	2500	89372297.1	161654.6	13432.8	13	1
**375**	5000	89351291.9	140386.0	13432.8	7	1
**375**	10000	89344926.7	134020.7	13432.8	6	1
**375**	25000	89290910.1	66571.4	0.0	1	0
**385**	1000	90125982.7	189771.3	0.0	22	0
**385**	2500	90111935.3	177040.8	0.0	13	0
**385**	5000	90092773.8	157835.4	0.0	8	0
**385**	10000	90073875.7	138695.9	0.0	5	0
**385**	25000	90014086.5	78906.7	0.0	2	0
**391**	1000	82945842.5	639243.4	34218.5	34	1
**391**	2500	82694504.2	617140.7	261829.9	20	2
**391**	5000	82672335.7	594840.5	261829.9	13	2
**391**	10000	82646062.7	568391.9	261829.9	10	2
**391**	25000	82562876.0	485205.1	261829.9	5	2
**400**	1000	81827034.9	19205.4	0.0	8	0
**400**	2500	81819199.1	11325.7	0.0	3	0

**400**	5000	81808027.1	0.0	0.0	0	0
**400**	10000	81808027.1	0.0	0.0	0	0
**400**	25000	81808027.1	0.0	0.0	0	0
**410**	1000	83988926.0	391109.6	0.0	18	0
**410**	2500	83972003.3	373243.1	0.0	7	0
**410**	5000	83964145.5	365363.4	0.0	5	0
**410**	10000	83935392.3	336610.2	0.0	1	0
**410**	25000	83935392.3	336610.2	0.0	1	0
**415**	1000	90837438.3	12225.6	7023.7	5	2
**415**	2500	90834848.3	7550.5	4938.5	2	1
**415**	5000	90832433.9	4938.5	4938.5	1	1
**415**	10000	90832433.9	0.0	0.0	0	0
**415**	25000	90832433.9	0.0	0.0	0	0
**421**	1000	97152238.2	200372.6	145390.3	13	3
**421**	2500	97146399.8	194534.2	145390.3	9	3
**421**	5000	97138849.3	179433.3	137839.9	5	1
**421**	10000	97125043.4	165627.3	137839.9	3	1
**421**	25000	97098375.3	137839.9	137839.9	1	1
**425**	1000	95183059.6	845959.3	0.0	16	0
**425**	2500	95165302.8	827785.5	0.0	5	0
**425**	5000	95156457.4	818940.0	0.0	2	0
**425**	10000	95146931.5	809414.1	0.0	1	0
**425**	25000	95146931.5	809414.1	0.0	1	0
**430**	1000	96543789.3	166900.4	126711.7	14	2
**430**	2500	96536195.0	159196.3	126711.7	8	2
**430**	5000	96523706.0	143349.1	123419.4	4	1
**430**	10000	96503776.2	123419.4	123419.4	1	1
**430**	25000	96503776.2	123419.4	123419.4	1	1
**436**	1000	78679325.5	885994.3	0.0	26	0
**436**	2500	78658276.4	864594.0	0.0	14	0
**436**	5000	78658276.4	864594.0	0.0	14	0
**436**	10000	78628469.6	834787.2	0.0	9	0
**436**	25000	78569382.8	775700.4	0.0	5	0
**441**	1000	72944111.5	22168.5	0.0	8	0
**441**	2500	72937680.4	15518.0	0.0	3	0
**441**	5000	72931666.4	9503.9	0.0	1	0
**441**	10000	72922162.5	0.0	0.0	0	0
**441**	25000	72922162.5	0.0	0.0	0	0
**445**	1000	73726440.5	21356.4	0.0	8	0
**445**	2500	73720382.6	15013.1	0.0	4	0
**445**	5000	73711273.8	5706.7	0.0	1	0
**445**	10000	73705610.9	0.0	0.0	0	0
**445**	25000	73705610.9	0.0	0.0	0	0
**449**	1000	76597131.4	7682.2	0.0	5	0

**449**	2500	76589734.6	2765.6	0.0	1	0
**449**	5000	76587122.6	0.0	0.0	0	0
**449**	10000	76587122.6	0.0	0.0	0	0
**449**	25000	76587122.6	0.0	0.0	0	0
**456**	1000	79022257.0	39354.6	0.0	8	0
**456**	2500	79016638.1	33691.8	0.0	4	0
**456**	5000	79013433.5	30487.2	0.0	3	0
**456**	10000	78998113.1	15166.8	0.0	1	0
**456**	25000	78982946.3	0.0	0.0	0	0
**465**	1000	81174863.6	345850.8	0.0	13	0
**465**	2500	81165030.4	336017.6	0.0	6	0
**465**	5000	81165030.4	336017.6	0.0	6	0
**465**	10000	81145078.8	315846.4	0.0	3	0
**465**	25000	81130263.2	300943.1	0.0	2	0
**470**	1000	83520336.3	293107.3	0.0	9	0
**470**	2500	83509559.3	282330.3	0.0	2	0
**470**	5000	83506705.9	279476.9	0.0	1	0
**470**	10000	83506705.9	279476.9	0.0	1	0
**470**	25000	83506705.9	279476.9	0.0	1	0
**475**	1000	85743201.7	32199.2	4126.4	13	2
**475**	2500	85733851.4	21312.5	2590.0	6	1
**475**	5000	85715370.3	0.0	0.0	0	0
**475**	10000	85715370.3	0.0	0.0	0	0
**475**	25000	85715370.3	0.0	0.0	0	0
**482**	1000	88464880.6	231671.9	9174.7	30	4
**482**	2500	88442843.7	202567.5	3863.0	12	1
**482**	5000	88424911.4	180135.6	0.0	5	0
**482**	10000	88413497.9	168722.1	0.0	3	0
**482**	25000	88379213.5	134437.8	0.0	1	0
**492**	1000	77605315.9	20171.2	0.0	6	0
**492**	2500	77601430.9	16286.2	0.0	4	0
**492**	5000	77585144.7	0.0	0.0	0	0
**492**	10000	77585144.7	0.0	0.0	0	0
**492**	25000	77585144.7	0.0	0.0	0	0
**496**	1000	76189955.0	4565.4	0.0	2	0
**496**	2500	76188221.1	2611.9	0.0	1	0
**496**	5000	76185609.1	0.0	0.0	0	0
**496**	10000	76185609.1	0.0	0.0	0	0
**496**	25000	76185609.1	0.0	0.0	0	0
**505**	1000	73283882.4	70193.0	0.0	8	0
**505**	2500	73279317.0	65583.7	0.0	5	0
**505**	5000	73273303.0	59569.6	0.0	3	0
**505**	10000	73258289.8	44556.5	0.0	1	0
**505**	25000	73258289.8	44556.5	0.0	1	0

**510**	1000	73572402.3	358098.3	4302.0	25	2
**510**	2500	73550189.9	334217.8	3270.4	9	1
**510**	5000	73547687.7	328379.3	0.0	7	0
**510**	10000	73533552.5	314244.2	0.0	5	0
**510**	25000	73508772.1	288783.3	0.0	3	0
**520**	1000	81675498.9	548484.1	493260.4	23	2
**520**	2500	81650762.3	525437.7	493260.4	9	2
**520**	5000	81633422.6	507878.5	493260.4	4	2
**520**	10000	81618804.6	493260.4	493260.4	2	2
**520**	25000	81618804.6	480508.0	480508.0	1	1
**525**	1000	79351272.9	337532.1	215012.6	13	3
**525**	2500	79341659.2	328159.8	215012.6	7	3
**525**	5000	79333911.2	316702.5	211522.7	4	2
**525**	10000	79333911.2	316702.5	211522.7	4	2
**525**	25000	79323946.4	296070.4	201009.2	2	1
**535**	1000	73433091.8	395718.9	0.0	17	0
**535**	2500	73423763.5	386346.7	0.0	11	0
**535**	5000	73413096.3	375679.5	0.0	8	0
**535**	10000	73403350.9	365934.1	0.0	7	0
**535**	25000	73329997.3	292580.5	0.0	2	0

Data S1. (separate file)

Macrostrat data originally retrieved from Ye and Peters (*30*), filtered for sedimentary Phanerozoic deposits, and supplemented with details on lithology and environment, as far as available through Macrostrat. The column "is_marine" is based on a keyword search to detect marine environments; non-marine strata were removed.

REFERENCES

1. M. J. Benton, D. A. T. Harper, *Introduction to Paleobiology and the Fossil Record* (Wiley-Blackwell, Oxford, 2009).

2. D. Jablonski, Evolutionary innovations in the fossil record: The intersection of ecology, development, and macroevolution. *J. Exp. Zool. B Mol. Dev. Evol.* **304**, 504–519 (2005).

3. J. J. Sepkoski, A factor analytic description of the Phanerozoic marine fossil record. *Paleobiology* **7**, 36–53 (1981).

4. J. J. Sepkoski, R. K. Bambach, D. M. Raup, J. W. Valentine, Phanerozoic marine diversity and the fossil record. *Nature* **293**, 435–437 (1981).

5. J. Alroy, M. Aberhan, D. J. Bottjer, M. Foote, F. T. Fürsich, P. J. Harries, A. J. W. Hendy, S. M. Holland, L. C. Ivany, W. Kiessling, M. A. Kosnik, C. R. Marshall, A. J. McGowan, A. I. Miller, T. D. Olszewski, M. E. Patzkowsky, S. E. Peters, L. Villier, P. J. Wagner, N. Bonuso, P. S. Borkow, B. Brenneis, M. E. Clapham, L. M. Fall, C. A. Ferguson, V. L. Hanson, A. Z. Krug, K. M. Layou, E. H. Leckey, S. Nürnberg, C. M. Powers, J. A. Sessa, C. Simpson, A. Tomašových, C. C. Visaggi, Phanerozoic trends in the global diversity of marine invertebrates. *Science* **321**, 97–100 (2008).

6. R. A. Close, R. B. J. Benson, E. E. Saupe, M. E. Clapham, R. J. Butler, The spatial structure of Phanerozoic marine animal diversity. *Science* **368**, 420–424 (2020).

7. A. M. Bush, J. L. Payne, Biotic and abiotic controls on the phanerozoic history of marine animal biodiversity. *Annu. Rev. Ecol. Evol.* **52**, 269–289 (2021).

8. R. B. J. Benson, R. A. Close, G. T. Antell, R. J. Whittaker, P. Valdes, A. Farnsworth, D. J. Lunt, S. Shen, J. Fan, E. E. Saupe, Marine animal diversity across latitudinal and temperature gradients during the Phanerozoic. *Palaeontology* **68**, e70006 (2025).

9. G. H. Mathes, J. Van Dijk, W. Kiessling, M. J. Steinbauer, Extinction risk controlled by interaction of long-term and short-term climate change. *Nat. Ecol. Evol.* **5**, 304–310 (2021).

10. P. Cermeño, C. García-Comas, A. Pohl, S. Williams, M. J. Benton, C. Chaudhary, G. Le Gland, R. D. Müller, A. Ridgwell, S. M. Vallina, Post-extinction recovery of the Phanerozoic oceans and biodiversity hotspots. *Nature* **607**, 507–511 (2022).

11. V. Rineau, J. Smyčka, D. Storch, Diversity dependence is a ubiquitous phenomenon across Phanerozoic oceans. *Sci. Adv.* **8**, eadd9620 (2022).

12. A. M. Bush, G. Hunt, R. K. Bambach, Sex and the shifting biodiversity dynamics of marine animals in deep time. *Proc. Natl. Acad. Sci. U.S.A.* **113**, 14073–14078 (2016).

13. C. J. Wilson, T. Reitan, L. H. Liow, Unveiling the underlying drivers of Phanerozoic marine diversification. *Proc. Biol. Sci.* **291**, 20240165 (2024).

14. A. Balembois, A. Pohl, B. Lefebvre, T. Servais, D. J. Lunt, P. J. Valdes, G. Beaugrand, Unravelling the drivers of marine biodiversity across the Phanerozoic. *Nat. Commun.* **16**, 8498 (2025).

15. J. T. Flannery-Sutherland, D. Silvestro, M. J. Benton, Global diversity dynamics in the fossil record are regionally heterogeneous. *Nat. Commun.* **13**, 2751 (2022).

16. D. M. Raup, Taxonomic diversity during the Phanerozoic: The increase in the number of marine species since the Paleozoic may be more apparent than real. *Science* **177**, 1065–1071 (1972).

17. P. J. Lu, M. Yogo, C. R. Marshall, Phanerozoic marine biodiversity dynamics in light of the incompleteness of the fossil record. *Proc. Natl. Acad. Sci. U.S.A.* **103**, 2736–2739 (2006).

18. M. Foote, J. J. Sepkoski, Absolute measures of the completeness of the fossil record. *Nature* **398**, 415–417 (1999).

19. S. M. Kidwell, S. M. Holland, The quality of the fossil record: Implications for evolutionary analyses. *Annu. Rev. Ecol. Syst.* **33**, 561–588 (2002).

20. K. Nanglu, T. M. Cullen, Across space and time: A review of sampling, preservational, analytical, and anthropogenic biases in fossil data across macroecological scales. *Earth Sci. Rev.* **244**, 104537 (2023).

21. A. B. Smith, Large-scale heterogeneity of the fossil record: Implications for Phanerozoic biodiversity studies. *Philos Trans. R Soc. Lond. B Biol. Sci.* **356**, 351–367 (2001).

22. J. S. Crampton, A. G. Beu, R. A. Cooper, C. M. Jones, B. Marshall, P. A. Maxwell, Estimating the rock volume bias in paleobiodiversity studies. *Science* **301**, 358–360 (2003).

23. S. E. Peters, Geologic constraints on the macroevolutionary history of marine animals. *Proc. Natl. Acad. Sci. U.S.A.* **102**, 12326–12331 (2005).

24. A. B. Smith, G. T. Lloyd, A. J. McGowan, Phanerozoic marine diversity: Rock record modelling provides an independent test of large-scale trends. *Proc. Biol. Sci.* **279**, 4489–4495 (2012).

25. D. A. Vilhena, A. B. Smith, Spatial bias in the marine fossil record. *PLOS ONE* **8**, e74470 (2013).

26. A. M. Dunhill, B. Hannisdal, M. J. Benton, Disentangling rock record bias and common-cause from redundancy in the British fossil record. *Nat. Commun.* **5**, 4818 (2014).

27. A. M. Bush, R. K. Bambach, Sustained Mesozoic–Cenozoic diversification of marine Metazoa: A consistent signal from the fossil record. *Geology* **43**, 979–982 (2015).

28. B. Hannisdal, S. E. Peters, Phanerozoic Earth system evolution and marine biodiversity. *Science* **334**, 1121–1124 (2011).

29. S. E. Peters, N. A. Heim, The geological completeness of paleontological sampling in North America. *Paleobiology* **36**, 61–79 (2010).

30. S. Ye, S. E. Peters, Bedrock geological map predictions for Phanerozoic fossil occurrences. *Paleobiology* **49**, 394–413 (2023).

31. S. E. Peters, J. M. Husson, J. Czaplewski, Macrostrat: A platform for geological data integration and deep-time earth crust research. *Geochem. Geophys. Geosyst.* **19**, 1393–1409 (2018).

32. T. Salles, L. Husson, M. Lorcery, B. Hadler Boggiani, Landscape dynamics and the Phanerozoic diversification of the biosphere. *Nature* **624**, 115–121 (2023).

33. J. D. Schueth, A. Wroblewski, K. P. Minor, Revised paleogeographic maps of the North American Western Interior Seaway: Applying integrated approaches for predictive reconstructions and recognizing bias from lack of data. *AAPG Bull.* **109**, 991–1025 (2025).

34. L. Buffan, L. A. Jones, M. Domeier, C. R. Scotese, S. Zahirovic, S. Varela, Mind the uncertainty: Global plate model choice impacts deep-time palaeobiological studies. *Methods Ecol. Evol.* **14**, 3007–3019 (2023).

35. B. H. Wilkinson, B. J. McElroy, S. E. Kesler, S. E. Peters, E. D. Rothman, Global geologic maps are tectonic speedometers—Rates of rock cycling from area-age frequencies. *Geol. Soc. Am. Bull.* **121**, 760–779 (2009).

36. A. T. Kocsis, C. R. Scotese, Mapping paleocoastlines and continental flooding during the Phanerozoic. *Earth Sci. Rev.* **213**, 103463 (2021).

37. H. D. Klemme, G. F. Ulmishek, Effective petroleum source rocks of the world: Stratigraphic distribution and controlling depositional factors. *AAPG Bull.* **75**, 1809–1851 (1991).

38. P. A. Allison, D. E. G. Briggs, Paleolatitudinal sampling bias, Phanerozoic species diversity, and the end-Permian extinction. *Geology* **21**, 65–68 (1993).

39. N. B. Raja, E. M. Dunne, A. Matiwane, T. M. Khan, P. S. Nätscher, A. M. Ghilardi, D. Chattopadhyay, Colonial history and global economics distort our understanding of deep-time biodiversity. *Nat. Ecol. Evol.* **6**, 145–154 (2022).

40. E. M. Dunne, D. Chattopadhyay, C. D. Dean, E. M. Dillon, E. M. Dowding, P. L. Godoy, J. A. Smith, N. B. Raja, Data equity in paleobiology: Progress, challenges, and future outlook. *Paleobiology* **51**, 237–249 (2025).

41. C. D. Dean, J. R. Thompson, Museum 'dark data' show variable impacts on deep-time biogeographic and evolutionary history. *Proc. Biol. Sci.* **292**, 20242481 (2025).

42. C. Wang, R. M. Hazen, Q. Cheng, M. H. Stephenson, C. Zhou, P. Fox, S. Shen, R. Oberhänsli, Z. Hou, X. Ma, Z. Feng, J. Fan, C. Ma, X. Hu, B. Luo, J. Wang, C. M. Schiffries, The Deep-Time Digital Earth program: Data-driven discovery in geosciences. *Nat. Sci. Rev.* **8**, nwab027 (2021).

43. M. D. Wilkinson, M. Dumontier, I. J. J. Aalbersberg, G. Appleton, M. Axton, A. Baak, N. Blomberg, J.-W. Boiten, L. B. da Silva Santos, P. E. Bourne, J. Bouwman, A. J. Brookes, T. Clark, M. Crosas, I. Dillo, O. Dumon, S. Edmunds, C. T. Evelo, R. Finkers, A. Gonzalez-Beltran, A. J. G. Gray, P. Groth, C. Goble, J. S. Grethe, J. Heringa, P. A. C. Hoen, R. Hooft, T. Kuhn, R. Kok, J. Kok, S. J. Lusher, M. E. Martone, A. Mons, A. L. Packer, B. Persson, P. Rocca-Serra, M. Roos, R. van Schaik, S.-A. Sansone, E. Schultes, T. Sengstag, T. Slater, G. Strawn, M. A. Swertz, M. Thompson, J. van der Lei, E. van Mulligen, J. Velterop, A. Waagmeester, P. Wittenburg, K. Wolstencroft, J. Zhao, B. Mons, The FAIR Guiding Principles for scientific data management and stewardship. *Sci. Data* **3**, 160018 (2016).

44. R Core Team, R: A language and environment for statistical computing. Version 4.4.2, R Foundation for Statistical Computing (2024); https://www.r-project.org/.

45. T. A. Neubauer, The fossil record of freshwater Gastropoda—A global review. *Biol. Rev.* **99**, 177–199 (2024).

46. MolluscaBase, Eds. MolluscaBase (2026); https://doi.org/10.14284/448.

47. J. T. Flannery-Sutherland, N. B. Raja, Á. T. Kocsis, W. Kiessling, fossilbrush: An R package for automated detection and resolution of anomalies in palaeontological occurrence data. *Methods Ecol. Evol.* **13**, 2404–2418 (2022).

48. W. Kiessling, Sampling-standardized expansion and collapse of reef building in the Phanerozoic. *Foss. Rec.* **11**, 7–18 (2008).

49. L. A. Jones, W. Gearty, B. J. Allen, K. Eichenseer, C. D. Dean, S. Galván, M. Kouvari, P. L. Godoy, C. S. C. Nicholl, L. Buffan, E. M. Dillon, J. T. Flannery-Sutherland, A. A. Chiarenza, palaeoverse: A community-driven R package to support palaeobiological analysis. *Methods Ecol. Evol.* **14**, 2205–2215 (2023).

50. S. Zahirovic, R. D. Müller, M. Seton, N. Flament, Tectonic speed limits from plate kinematic reconstructions. *Earth Planet. Sci. Lett.* **418**, 40–52 (2015).

51. X. Li, D. Yang, L. Wang, J. J. Wiens, The past and future of known biodiversity: Rates, patterns, and projections of new species over time. *Sci. Adv.* **11**, eadz3071 (2025).

52. S. N. Wood, *Generalized Additive Models: An Introduction with R* (Chapman and Hall/CRC, Boca Raton, ed. 2, 2017).

53. S. Wood, mgcv: Mixed GAM Computation Vehicle with Automatic Smoothness Estimation, R package version 1.9-4 (2025); http://CRAN.R-project.org/package=mgcv.

54. M. Strimas-Mackey, smoothr: Smooth and Tidy Spatial Features, R package version 1.2.1 (2025); http://CRAN.R-project.org/package=smoothr.

55. L. A. Jones, C. D. Dean, W. Gearty, B. J. Allen, rmacrostrat: An R package for accessing and retrieving data from the Macrostrat geological database. *Geosphere* **20**, 1456–1467 (2024).

56. GEBCO Bathymetric Compilation Group 2023, The GEBCO_2023 Grid—A continuous terrain model of the global oceans and land, version 1, NERC EDS British Oceanographic Data Centre NOC (2023); https://doi.org/10.5285/F98B053B-0CBC-6C23-E053-6C86ABC0AF7B.

57. A. Zizka, CoordinateCleaner: Automated Cleaning of Occurrence Records from Biological Collections, package version 3.0.1 (2023); http://CRAN.R-project.org/package=CoordinateCleaner.

58. W. Gearty, deeptime: an R package that facilitates highly customizable and reproducible visualizations of data over geological time intervals. *Big Earth Data* **10**, 1–17 (2025).

59. H. Wickham, R. François, L. Henry, K. Müller, D. Vaughan, dplyr: A Grammar of Data Manipulation, R package version 1.1.4 (2023); https://CRAN.R-project.org/package=dplyr.

60. C. Ritz, F. Baty, J. C. Streibig, D. Gerhard, Dose-response analysis using R. *PLOS ONE* **10**, e0146021 (2015).

61. D. Vaughan, M. Dancho, furrr: Apply Mapping Functions in Parallel using Futures, R package version 0.3.1 (2022); https://CRAN.R-project.org/package=furrr.

62. H. Bengtsson, A unifying framework for parallel and distributed processing in R using futures. *R J.* **13**, 208 (2021).

63. H. Wickham, *Ggplot2: Elegant Graphics for Data Analysis* (Springer-Verlag, New York, 2016).

64. B. Auguie, gridExtra: Miscellaneous Functions for "Grid" Graphics, R package version 2.3.1 (2017); http://CRAN.R-project.org/package=gridExtra.

65. H. Wickham, httr2: Perform HTTP Requests and Process the Responses, R package version 1.2.2 (2025); http://CRAN.R-project.org/package=httr2.

66. W. N. Venables, B. D. Ripley, *Modern Applied Statistics with S* (Springer, New York, ed. 4, 2002).

67. J. Pinheiro, D. Bates, R Core Team, nlme: Linear and Nonlinear Mixed Effects Models, R package version 3.1-166 (2024). https://CRAN.R-project.org/package=nlme.

68. J. Pinheiro, D. M. Bates, *Mixed-Effects Models in S and S-PLUS* (Springer-Verlag, New York, 2000).

69. T. L. Pedersen, patchwork: The Composer of Plots, R package version 1.3.2 (2025); https://CRAN.R-project.org/package=patchwork.

70. S. Varela, J. González-Hernández, L. F. Sgarbi, C. Marshall, M. D. Uhen, S. Peters, M. McClennen, paleobioDB: an R package for downloading, visualizing and processing data from the Paleobiology Database. *Ecography* **38**, 419–425 (2015).

71. G. L. Simpson, permute: Functions for Generating Restricted Permutations of Data, R package version 0.9-8 (2025). https://CRAN.R-project.org/package=permute.

72. H. Wickham, L. Henry, purrr: Functional Programming Tools, R package version 1.2.0 (2025); http://CRAN.R-project.org/package=purrr.

73. D. Eddelbuettel, R. Francois, J. J. Allaire, K. Ushey, Q. Kou, N. Russell, I. Ucar, D. Bates, J. Chambers, Rcpp: Seamless R and C++ Integration, R package version 1.1.1 (2025); https://CRAN.R-project.org/package=Rcpp.

74. R. D. Müller, J. Cannon, X. Qin, R. J. Watson, M. Gurnis, S. Williams, T. Pfaffelmoser, M. Seton, S. H. J. Russell, S. Zahirovic, GPlates: Building a virtual Earth through deep time. *Geochem. Geophys. Geosyst.* **19**, 2243–2261 (2018).

75. Á. T. Kocsis, N. B. Raja, S. Williams, E. M. Dowding, rgplates: R Interface for the GPlates Web Service and Desktop Application, version 0.6.1, Zenodo (2025); https://doi.org/10.5281/ZENODO.8093990.

76. P. Massicotte, A. South, rnaturalearth: World Map Data from Natural Earth, R package version 1.1.0 (2025); https://CRAN.R-project.org/package=rnaturalearth.

77. E. Pebesma, Simple features for R: Standardized support for spatial vector data. *R J.* **10**, 439–446 (2018).

78. E. Pebesma, R. Bivand, *Spatial Data Science: With Applications in R* (Chapman and Hall/CRC, New York, ed. 1, 2023).

79. D. Cooley, sfheaders: Converts Between R Objects and Simple Feature Objects, R package version 0.4.5 (2025); https://CRAN.R-project.org/package=sfheaders.

80. A. Onofri, statforbiology: Data Analyses in Agriculture and Biology, R package version 1.0.2 (2025); http://CRAN.R-project.org/package=statforbiology.

81. H. Wickham, stringr: Simple, Consistent Wrappers for Common String Operations, R package version 1.6.0 (2025); https://CRAN.R-project.org/package=stringr.

82. R. J. Hijmans, terra: Spatial Data Analysis, R package version 1.7-83 (2024); https://cran.r-project.org/package=terra.

83. H. Wickham, D. Vaughan, M. Girlich, tidyr: Tidy Messy Data, R package version 1.3.2 (2025); https://CRAN.R-project.org/package=tidyr.

84. J. Oksanen, G. L. Simpson, F. G. Blanchet, R. Kindt, P. Legendre, P. R. Minchin, R. B. O'Hara, P. Solymos, M. H. H. Stevens, E. Szoecs, H. Wagner, M. Barbour, M. Bedward, B. Bolker, D. Borcard, T. Bormann, G. Carvalho, M. Chirico, M. De Caceres, S. Durand, H. B. A. Evangelista, R. FitzJohn, M. Friendly, B. Furneaux, G. Hannigan, M. O. Hill, L. Lahti, C. Martino, D. McGlinn, M.-H. Ouellette, E. R. Cunha, T. Smith, A. Stier, C. J. F. Ter Braak, J. Weedon, vegan: Community Ecology Package, R package version 2.7-2 (2025); http://CRAN.R-project.org/package=vegan.
